# Kinetic Resolution of β-Branched Aldehydes through Peptide-Catalyzed Conjugate Addition Reactions

**DOI:** 10.1021/jacs.4c03617

**Published:** 2024-07-03

**Authors:** Greta Vastakaite, Alena Budinská, Claude L. Bögli, Linus B. Boll, Helma Wennemers

**Affiliations:** Laboratorium für Organische Chemie, ETH Zürich, D-CHAB, Vladimir-Prelog-Weg 3, Zürich 8093, Switzerland

## Abstract

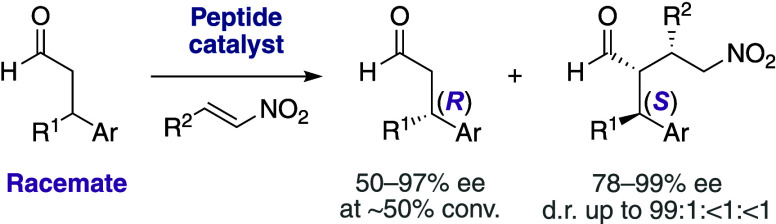

The catalytic kinetic resolution of racemic β-branched aldehydes offers a straightforward stereoselective entry to aldehydes and addition products. Yet, control over stereoselectivity is difficult due to the conformational flexibility of β-branched aldehydes. Here, we show that the peptide catalyst H-dPro-αMePro-Glu-NH_2_ resolves β-branched aldehydes through reaction with nitroolefins and provides γ-nitroaldehydes with three consecutive stereogenic centers in high yields and stereoselectivities. Kinetic, NMR spectroscopic, and computational studies provided insights into the selectivity-determining step and origins of the kinetic resolution.

## Introduction

**Scheme 1:**
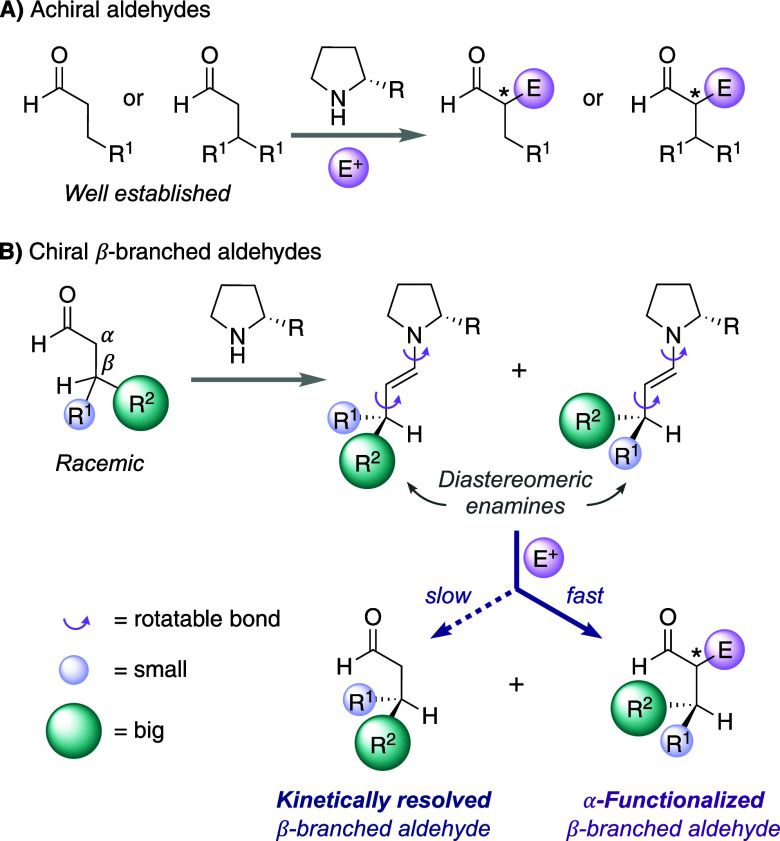
Organocatalytic α-Functionalization of (A) Achiral Aldehydes and (B) Racemic β-Branched Aldehydes through Kinetic Resolution

Over the past two decades, proline and proline-based peptides have been established as versatile catalysts to access, via an enamine intermediate, α-functionalized carbonyl compounds.^1−5^ Achiral linear and symmetric β-branched aldehydes are widely used substrates and react readily with a variety of electrophiles (Scheme 1A). In contrast, β-branched aldehydes with two different substituents at C^β^ have rarely been used. Addition reactions with control over the stereochemistry of all stereogenic centers require either the use of enantiomerically enriched starting aldehydes or the kinetic resolution of racemic aldehydes by the catalyst. The latter approach is convenient but challenging since the catalyst needs to distinguish the stereochemistry at a remote center that is connected to the enamine intermediate by a rotatable bond (Scheme 1B). In fact, previous examples of kinetic resolutions with racemic β-branched aldehydes proceeded with moderate selectivity^6,7^ or required a specific aldehyde with an additional stereogenic center.^8,9^

We reasoned that an amine-based catalyst with properly positioned reactive sites could provide a suitable chiral environment for the kinetic resolution of racemic β-branched aldehydes. Stereocontrol and kinetic resolution would arise either during enamine formation or upon the reaction of the diastereomeric enamines with a suitable electrophile. This method would complement enantioselective syntheses of β-branched aldehydes by reduction of α,β-unsaturated aldehydes^10−12^ or carboxylic acids,^13^ conjugate addition to enals,^14−18^ and enantioselective isomerization of allylic alcohols.^19,20^ A unique further benefit would be access to α-functionalized addition products with two or more consecutive stereogenic centers (Scheme 1B).

Herein, we present the kinetic resolution of racemic β-branched aldehydes through peptide-catalyzed addition reactions with nitrostyrenes as electrophiles. The method yields enantiomerically enriched β-branched aldehydes and γ-nitroaldehydes bearing three consecutive stereogenic centers with high stereoselectivity. Mechanistic studies revealed the reaction of the diastereoisomeric enamines with the electrophile as the key step of the kinetic resolution.

## Results and Discussion

### Access to Enantioenriched β-Branched Aldehydes

**Scheme 2:**
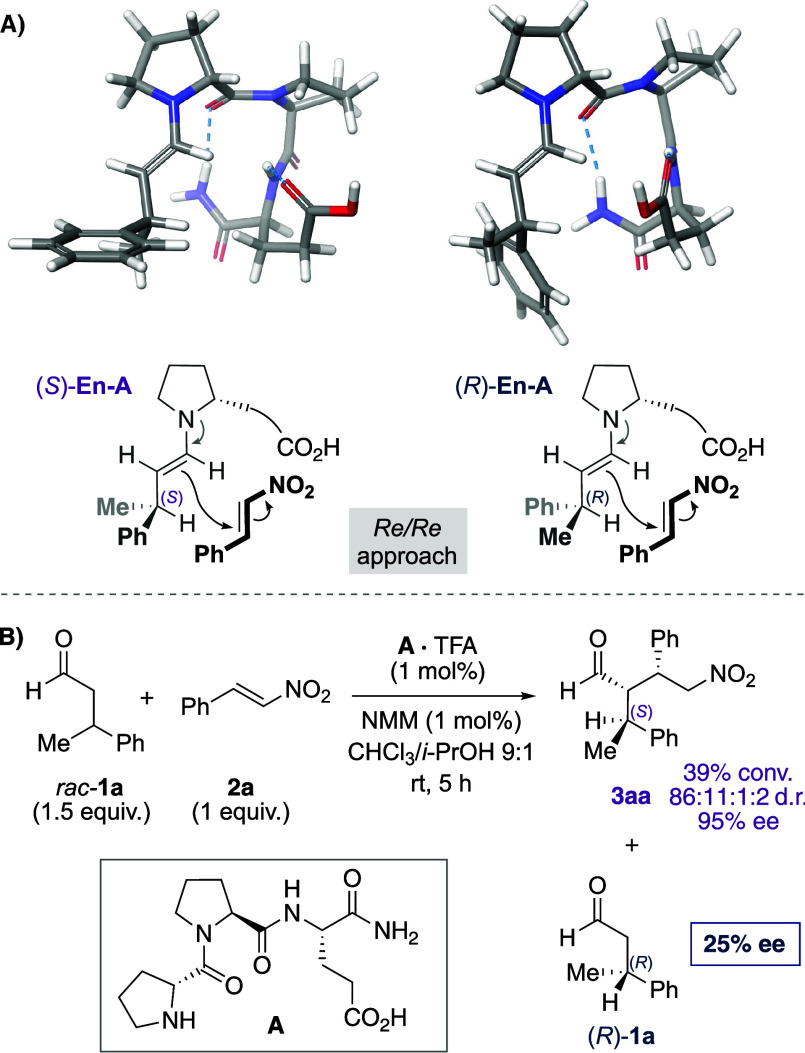
(A) Calculated Lowest-Energy Conformers of s-*trans* Enamines Formed between Peptide Catalyst A and (*S*)- and (*R*)-Configured 3-Phenylbutanal; (B) Initial Kinetic Resolution Results Conversion of nitrostyrene (**2a**) into **3aa** was determined by ^1^H NMR spectroscopy of the crude reaction mixture. Diastereoselectivity was determined by ^1^H NMR spectroscopy, enantioselectivity by chiral stationary phase SFC and HPLC analysis.

We reasoned that the kinetic resolution of β-branched aldehydes would require a catalyst that creates diastereomeric enamines with different environments around C^β^ of the former aldehyde. We envisioned the catalyst H-dPro-Pro-Glu-NH_2_ (**A**) and related peptides to fulfill this requirement. In contrast to proline derivatives with a sterically bulky substituent at C^α^, this highly chemo- and stereoselective peptide catalyst forms a β-turn with a far reach of the glutamic acid (Glu) residue.^21^ To probe this reasoning, we started with a computational analysis of diastereoisomeric enamines derived from **A** and (*S*)- and (*R*)-configured 3-phenylbutanal. Conformational searches using CREST^22,23^ followed by reranking of the conformer-rotamer ensemble using CENSO^24^ predicted the lowest-energy s-*trans* enamines, which were further optimized at the PBE0-D3BJ/def2-TZVP level of theory using ORCA^25^ (Scheme 2A). Both enamines adopt a conformation with minimal allylic strain.^26^ The diastereoisomeric enamines (*S*)-**En-A** and (*R*)-**En-A** differ by the position of the methyl and phenyl groups, which supports the expectation of different reactivities with electrophiles.

Since peptide **A** is a powerful catalyst for conjugate additions with nitroolefins,^27−30^ we envisioned that (*E*)-β-nitrostyrene would be an appropriate electrophile to achieve the kinetic resolution. The reaction between enamines and nitroolefins takes place by a *Re*/*Re* approach.^31,32^ This approach positions the resulting nitronate intermediate close to the COOH group of Glu, which serves as an intramolecular proton donor.^33^

We initiated our experimental studies by reacting racemic 3-phenylbutanal (*rac*-**1a**) and nitrostyrene (**2a**) in the presence of 1 mol % of peptide **A** (Scheme 2B). Under previously established conditions,^34,35^ only 39% of nitrostyrene converted into addition product **3aa** within 5 h. In contrast, nitrostyrene is quickly consumed in reactions with aldehydes bearing only one substituent at C^β^ under the same reaction conditions (full conv. after 5 h with *n*-butanal). The four possible diastereoisomers of γ-nitroaldehyde **3aa** that features three consecutive stereogenic centers formed in a ratio of 86:11:1:2 and an enantioselectivity of 95% ee of the major stereoisomer.^36^ This diastereoisomeric ratio of the products implied stereodifferentiation at C^β^ and, hence, the kinetic resolution of the aldehyde. Indeed, the remaining aldehyde **1a** was obtained with an enantiomeric excess of 25% of the (*R*)-configured enantiomer.

**Scheme 3:**
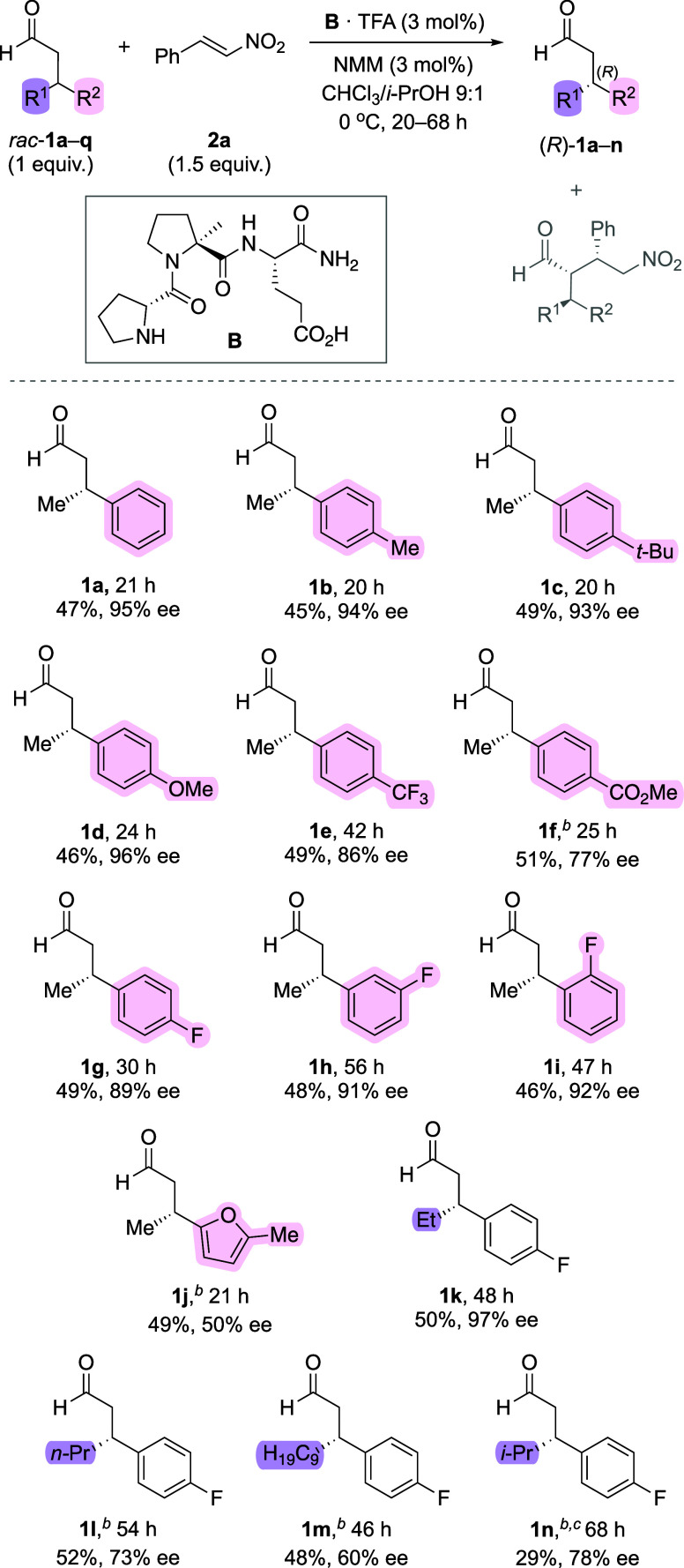
Enantioenriched Aldehydes Obtained by Peptide-Catalyzed Kinetic Resolution The amount of remaining unreacted aldehyde **1** is listed as determined by ^1^H NMR spectroscopy. Enantioselectivities were determined by chiral stationary phase SFC or HPLC analyses. Enantioselectivity was determined after reduction to the corresponding alcohol. 20 mol % of **B**·TFA/NMM was used.

We reasoned that the conversion and enantiomeric excess of the aldehyde could be improved by optimizing the reaction parameters. Indeed, variations of the catalyst, stoichiometry, solvent, and temperature improved the kinetic resolution (Tables S1–S6). An excess of nitrostyrene (1.5 equiv), optimized peptide H-dPro-αMePro-Glu-NH_2_ (**B**) bearing an α-methylproline residue,^37^ and a reaction temperature of 0 °C enhanced the enantioselectivity. Under these conditions, the kinetic resolution afforded (*R*)-configured 3-phenylbutanal (**1a**) with an enantiomeric excess of 95% at a ∼50% conversion of *rac*-**1a** (Scheme 3).

With established reaction conditions, we examined the scope of the kinetic resolution of racemic β-branched aldehydes (Scheme 3). In each reaction, the reaction progress was monitored by ^1^H NMR spectroscopy and the enantioselectivity was determined at a conversion close to 50%, the maximum possible in a kinetic resolution. For aldehydes bearing a methyl and an aryl moiety, highly enantiomerically enriched aldehydes (77–96% ee) were obtained, regardless of the substituent at the aromatic moiety. Both electron-donating Me (**1b**), *t*-Bu (**1c**), OMe (**1d**) and electron-withdrawing CF_3_ (**1e**), CO_2_Me (**1f**) groups in the *para* position as well as fluorine in the *meta* and *ortho* position (**1g**–**1i**) of the aryl moiety were tolerated. An aldehyde with a methylfuran group (**1j**) was resolved with lower enantioselectivity (50% ee). Aldehydes bearing ethyl or other aliphatic substituents instead of the methyl group at C^β^ (**1k**–**1n**) were resolved with high (Et; 97% ee) to moderate (*n*-Pr, *i*-Pr, C_9_H_19_; 60–78% ee) enantioselectivity. Overall, the selectivity was highest for aldehydes with Me/Ar substituents at C^β^ and lower for aldehydes with longer alkyl chains or a heteroaromatic moiety. Of note, we refrain from using the selectivity factor (*s*)^38^ to describe the efficiency of the kinetic resolution since the reaction is not first order with respect to the aldehyde, and the order changes over the course of the reaction.^33,39−41^ As a result, the *s*-value increases with higher conversion (Tables S7–S9).

Aldehydes bearing two alkyl substituents at C^β^ were resolved with significantly lower stereoselectivity (*vide infra*), implying that the aromatic moiety is crucial for efficient kinetic resolution, possibly through engaging in a noncovalent interaction with the approaching nitroolefin. The kinetic resolution proceeded with only 3 mol % of the peptide catalyst, except for an aldehyde with a bulky *i*-Pr group that required a catalyst loading of 20 mol %. Importantly, since the enantiomer of the peptide catalyst is readily accessible, the methodology enables access to either enantiomer of the aldehyde.

### Stereoselective Access to γ-Nitroaldehydes with Three Consecutive Stereogenic Centers

In addition to enantioenriched β-branched aldehydes, the kinetic resolution provides γ-nitroaldehydes with three consecutive stereogenic centers. These compounds are difficult to access by other means and are valuable for downstream derivatization into, e.g., chiral pyrrolidine or butyrolactones.^5,42−44^ We, therefore, also explored the scope of these addition products.

**Scheme 4:**
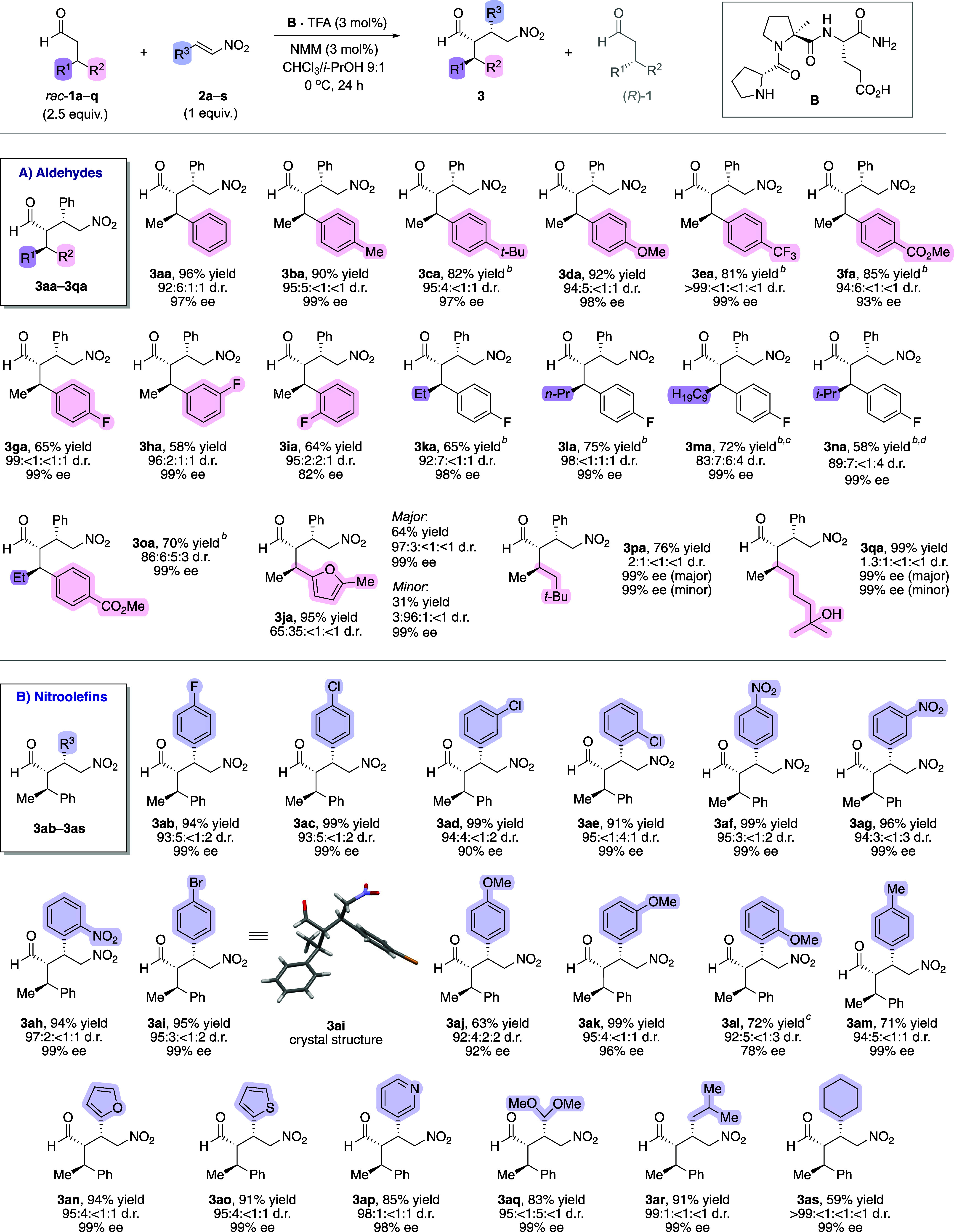
Scope of the Conjugate Addition Reactions between β-Branched Aldehydes and Nitroolefins Diastereoselectivities were determined by ^1^H NMR spectroscopy of the isolated products; enantioselectivities were determined by chiral stationary phase HPLC or SFC analyses. 10 mol % of **B**·TFA/NMM used. Reaction time was 48 h. Reaction time was 4 days.

Here, an excess of the racemic aldehyde (2.5 equiv) under otherwise identical reaction conditions was beneficial for high product yields and stereoselectivities (Scheme 4). Different β-branched aldehydes (*rac*-**1a**–**1q**) yielded upon reaction with β-nitrostyrene γ-nitroaldehydes (**3aa**–**3qa**) (Scheme 4A). Aldehydes with a methyl and electron-rich aryl substituent at C^β^ provided products **3aa**–**3da** with uniformly high diastereoselectivities (92:6:1:1 to 95:5:<1:<1 d.r.)^36^ and excellent enantioselectivities (97–99% ee). Aldehydes bearing electron-withdrawing substituents at the aryl moiety, e.g., CF_3_ (**3ea**), CO_2_Me (**3fa**), or alkyl groups at C^β^ other than methyl (**3ka**–**3oa**), required 10 mol % of the catalyst for high conversions, possibly due to lower enamine reactivity. A substrate with a heteroaromatic substituent provided the product (**3ja**), albeit with lower diastereoselectivity (65:35:<1:<1). Importantly, the diastereoisomers could be separated easily by column chromatography and were isolated as essentially optically pure compounds (≥99% ee). Aldehydes with a combination of two alkyl substituents at C^β^ also reacted to the γ-nitroaldehydes (**3pa** and **3qa**), but with low diastereoselectivity (2:1:<1:<1 and 1.3:1:<1:<1), indicating that the differentiation between two alkyl groups is challenging. Nevertheless, both diastereomers formed with excellent enantioselectivity (>99% ee), implying that a high *Re*-facial selectivity of the C–C bond-forming step is maintained. Overall, the selectivity trends of the kinetic resolution (Scheme 3) are reflected in the γ-nitroaldehyde products and show that the peptide catalyst resolves β-branched aldehydes with alkyl and aryl substituents at C^β^ particularly efficiently.

The scope of nitroolefins was also broad (Scheme 4B). Product yields of reactions of racemic 3-phenylbutanal (*rac*-**1a**) with nitrostyrene derivatives bearing electron-withdrawing (**3ab**–**3ai**) and electron-donating (**3aj**–**3am**) substituents were typically higher than 90%, with diastereoselectivities greater than 92:4:2:2 and for most products an enantioselectivity of 99% ee of the major diastereomer. Products from nitroolefins with *O*-, *S*-, and even *N*-heteroaromatic substituents (**3an**, **3ao**, **3ap**) were also obtained in high yields and stereoselectivities (85–94% yield, 95:4:<1:1–98:1:<1:1 d.r. and 98–99% ee). Even nitroolefins bearing aliphatic substituents (**3aq**–**3as**) underwent the conjugate addition reaction with excellent selectivity, highlighting that the catalytic system is highly tolerant to variations on the nitroolefin.

Crystal structures of several γ-nitroaldehyde products were obtained (**3ai** in Scheme 4B; **3aa**–**3ad**, **3al** in Figures S3–S8) and revealed the absolute configuration of the major product. These structures corroborated that the stereogenic centers of the major diastereomer form through a *Re*/*Re* facial approach between the nitroolefin and enamine from the (*S*)-configured aldehyde.

### Mechanism of the Kinetic Resolution

**Scheme 5:**
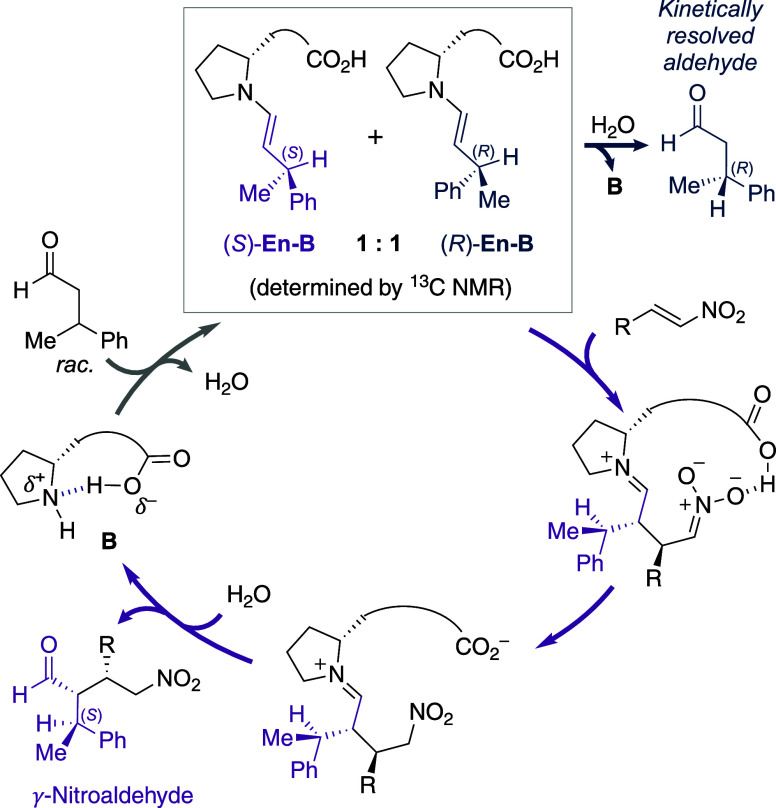
Proposed Catalytic Cycle of the Peptide-Catalyzed Kinetic Resolution of Racemic β-Branched Aldehydes

The kinetic resolution of the β-branched aldehydes can either take place upon formation of the diastereoisomeric enamine intermediates or during their reaction with the nitroolefin. To elucidate the origin of the kinetic resolution, we first monitored the formation of the enamines by NMR spectroscopy (Scheme 5, top). Enamines formed by amine-based catalysts that bear an acidic moiety are highly reactive and, therefore, difficult to characterize, especially in protic solvents.^45^ We, therefore, used anhydrous DMSO-*d*_6_ in the presence of molecular sieves to monitor enamine formation between catalyst **B** and racemic aldehyde **1a**. In this solvent, the diastereomeric enamines (*S*)-**En-B** and (*R*)-**En-B** formed in a 1:1 ratio (Schemes S2–S4).^46^ This result suggests similar stabilities of the diastereomers and implies that the kinetic resolution does not take place during enamine formation but rather originates from different reactivity of the diastereomeric enamines with the nitroolefin.

**Scheme 6:**
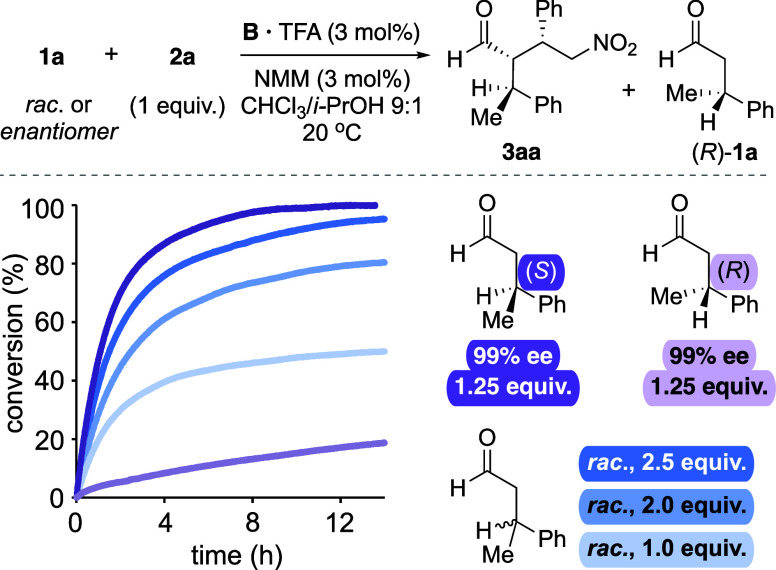
Peptide-Catalyzed Conjugate Addition Reaction with Racemic, (*S*)- or (*R*)-3-Phenylbutanal Monitored by *In Situ* IR Spectroscopy

For further analysis, we compared the reaction progress of the racemate and the (*R*)- and (*S*)-enantiomers of 3-phenylbutanal (**1a**) with nitrostyrene in the presence of 3 mol % of peptide catalyst **B** (Scheme 6). *In situ* infrared (IR) spectroscopy served as a convenient tool to monitor the formation of γ-nitroaldehyde **3aa**. The reaction progress curves show that the reaction proceeds significantly faster with (*S*)-**1a** than with (*R*)-**1a** (Scheme 6, dark and light purple).^47^ With 1.25 equiv of (*S*)-**1a** relative to nitrostyrene, 50% conversion was reached after ∼1 h at 20 °C, whereas the reaction with (*R*)-**1a** proceeded only to 20% conversion after 14 h. With racemic **1a**, a similar reaction progress as with (*S*)-**1a** required twice as many equivalents (2.5 equiv of *rac*-**1a** instead of 1.25 equiv of (*S*)-**1a**; Scheme 6, dark blue). These findings corroborate that the (*S*)-configured β-branched aldehyde reacts significantly faster compared to the (*R*)-enantiomer, a result that is in agreement with the observed stereochemical outcome of the reaction.

Considering the predicted lowest-energy structures of (*S*)-**En-B** and (*R*)-**En-B** and the preferred *Re*/*Re* facial approach, a phenyl substituent at the *Re*-face of the enamine allows for faster reaction with the nitroolefin compared to a methyl group (Scheme 2). This implies that the *Re*-face of (*S*)-**En-B** (Ph at *Re*-face) is less sterically hindered compared to that of (*R*)-**En-B** (Me at *Re*-face). In addition, the aryl substituent in (*S*)-**En-B** might interact with the incoming nitroolefin electrophile, e.g., by π–π stacking or CH−π interactions. Such an interaction is supported by the low selectivity observed for aldehydes bearing two alkyl substituents at C^β^ but the high selectivity of reactions with nitroolefins bearing either aryl or alkyl substituents.

## Conclusions

In conclusion, this study presents an organocatalytic kinetic resolution to yield enantiomerically enriched β-branched aldehydes and γ-nitroaldehydes with three consecutive stereogenic centers in high yields and stereoselectivities. The key to the kinetic resolution is the peptide catalyst H-dPro-αMePro-Glu-NH_2_. This chiral secondary amine forms diastereomeric enamines with distinctly different chiral environments, reminiscent of the chiral pockets of enzymes, that react at different rates with the electrophile.

Of note, contemporary peptide catalysis started with seminal work by Miller et al. on the kinetic resolution of α-amido alcohols through acylation reactions.^48,49^ Further examples include the resolution of (thio)formamides, sulfinyl chlorides, and planar-chiral metallocenes.^50−54^ Here, we show the power of peptide catalysts for kinetic resolution via a C–C bond formation that yields, in addition to the resolved aldehyde substrate, a synthetically versatile addition product. Combined, the work highlights the power of peptide catalysts to facilitate kinetic resolutions that are difficult to achieve by enzymes or other synthetic catalysts.

## Supporting Information Available

Experimental details on the reactions, mechanistic studies, and computational analyses, including the analytical data of the presented compounds (PDF)

## Abbreviations

TFA: trifluoroacetic acid
NMM: *N*-methylmorpholine
Pro: proline
Glu: glutamic acid
αMePro: α-methylproline
NMR: nuclear magnetic resonance
SFC: supercritical fluid chromatography
HPLC: high-performance liquid chromatography
DMSO: dimethyl sulfoxide
IR: infrared
