# Supplementary material for: Kinetic Resolution of β-Branched Aldehydes through Peptide-Catalyzed Conjugate Addition Reactions
Source: J Am Chem Soc. 2024 Jul 3;146(28):19101–7. doi: 10.1021/jacs.4c03617 (PMC11258695; doi:10.1021/jacs.4c03617)
Supplement: Supplementary file 1 — ja4c03617_si_001.pdf [file ja4c03617_si_001.pdf]

Supporting Information

Kinetic Resolution of β-Branched Aldehydes Through Peptide-Catalyzed Conjugate Addition Reactions

Greta Vastakaite,[§] Alena Budinská,[§] Claude L. Bögli, Linus B. Boll, Helma Wennemers*

Laboratory of Organic Chemistry, ETH Zurich, Vladimir-Prelog-Weg 3, CH-8093 Zurich, Switzerland.

email: helma.wennemers@org.chem.ethz.ch

Table of Contents

1 General Aspects and Materials 3

2 Synthesis and Analytical Data of Peptides 4

2.1 General Protocols for Solid Phase Peptide Synthesis 4

2.2 Analytical Data of Peptides 5

3 Optimization of the Reaction Conditions 11

4 Synthesis and Analytical Data of Racemic β-Branched Aldehydes 15

5 Kinetic Resolution of Racemic β-Branched Aldehydes 23

6 Synthesis and Analytical Data of γ-Nitroaldehydes 41

6.1 General Procedures 41

6.2 Comparison of Racemic Samples 42

6.3 Assignment of Diastereoisomers 43

6.4 Epimerization Test 44

6.5 Analytical Data 45

7 ^{1}H and ^{13}C NMR Spectra of the Peptide Catalysts 83

8 NMR Spectra of β-Branched Aldehydes 84

9 NMR Spectra of Alcohols 105

10 ^{1}H and ^{13}C NMR Spectra of γ-Nitroaldehydes 113

11 NMR Spectroscopic Analysis of Enamine Intermediates 149

12 Kinetic Experiments 163

13 X-Ray Crystal Structures 164

14 Computational Details 170

15 References 175

1 General Aspects and Materials

Reagents and materials were of the highest commercially available grade and used without further purification if not stated otherwise. 4-Methylmorpholine was freshly distilled before use. Reactions were monitored by **thin layer chromatography** using Merck silica gel 60 F254 glass plates. Visualization of the compounds was achieved by UV-Vis, 2,4-dinitrophenylhydrazine (DNP) or $KMnO_4$ staining. **Flash column chromatography** was performed using a Teledyne Isco CombiFlash system with prepacked silica columns. **^{1}H and ^{13}C NMR** spectra were recorded on a Bruker DRX 400, a Bruker AV III 400 (400 MHz/100 MHz) or a Bruker AV III 600 (600 MHz/150 MHz). All spectra were recorded at 25 °C, unless stated otherwise. Chemical shifts (δ) are reported in parts per million (ppm) relative to the signal of tetramethylsilane (TMS) using the residual solvent signals. **SFC** analyses were performed on an analytical SFC with a diode array detector ACQUITY-UPLC-PDA from Waters using chiral stationary phase columns (Trefoil, AS, AD, IA, Whelk, IC, OD, OJ) (150 mm x 30 mm) from Daicel or Waters under the reported conditions. **HPLC** analyses were performed on an analytical Ultimate 3000 HPLC system from Dionex with a diode array detector and chiral stationary phase columns (Daicel AD-H, AS-H, OD-H or Daicel IA, IB N-5, IC, ID, IE, IF, IG or IH). **High-resolution electron ionization** (HR-EI) mass spectra were measured on a Thermo Scientific Q Exactive GC Orbitrap with a direct probe. A Bruker Daltonics maXis (ESI-QTOF) was used for high-resolution electrospray ionization (HR-ESI) mass spectrometry. **IR spectra** were recorded on an Agilent Technologies Cary 630 FTIR. ***In-situ* FT-IR spectroscopy** was carried out on a ReactIR R4000 (SiComb probe) with a spectral range of 4000–650 cm^{-1}. All measurements were performed at 20 °C and spectra were recorded every minute. **X-ray crystal structure analyses** were carried out on an XtaLAB Synergy, Dualflex, Pilatus 300K diffractometer.

2 Synthesis and Analytical Data of Peptides

2.1 General Protocols for Solid Phase Peptide Synthesis

All peptides were prepared using general procedures for Fmoc/*t*-Bu solid-phase peptide synthesis (SPPS) on Rink Amide resin (Novabiochem® Rink amide AM resin, 200-400 mesh) described below.

General procedure for peptide couplings: *i*-Pr_2NEt (6 equiv.) was added to a solution of Fmoc-Xaa-OH (3 equiv.) and HATU (3 equiv.) in DMF. The solution of the activated amino acid (≈ 100 mM) was added to the amino-functionalized resin (swollen in CH_2Cl_2), and the mixture was agitated for 1 h before washing with DMF (3x) and CH_2Cl_2 (3x).

General procedure for Fmoc-deprotections: A solution of 20% piperidine in DMF was added to the resin (swollen in CH_2Cl_2). The reaction mixture was agitated for 10 min, drained, and the piperidine treatment was repeated twice for 10 min. Finally, the resin was washed with DMF (3x) and CH_2Cl_2 (3x).

General procedure for side chain deprotection and cleavage of the peptides from the solid support: The peptides were deprotected and cleaved from the resin by stirring in a mixture of TFA/TIS/H_2O (95:2.5:2.5) for 1 h and a second time for 30 min. Pooling of the filtrates and removal of all volatiles under reduced pressure followed by precipitation and thorough washing with Et_2O afforded the peptides as their TFA-salts. The peptides were redissolved in MeCN/H_2O (1:1), dried by lyophilisation and used without further purification.

HATU: Hexafluorophosphate Azabenzotriazole Tetramethyl Uronium; DMF: *N*,*N*-Dimethylformamide; TFA: trifluoroacetic acid; TIS: Triisopropylsilane)

2.2 Analytical Data of Peptides

TFA·H-DPro-Pro-Glu-NH$_2$ (A-DLL)

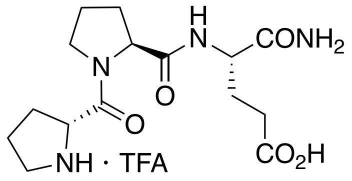

The analytical data are in agreement with previously published data.[1] **^{1}H NMR** (400 MHz, D_2O) δ = 4.66 (dd, J = 8.9, 6.8 Hz, 1H), 4.48 (dd, J = 9.3, 3.6 Hz, 1H), 4.36 (dd, J = 9.4, 5.1 Hz, 1H), 3.79 – 3.71 (m, 1H), 3.66 – 3.59 (m, 1H), 3.51 – 3.37 (m, 2H), 2.62 – 2.55 (m, 1H), 2.55 – 2.49 (m, 2H), 2.37 – 2.28 (m, 1H), 2.17 – 1.98 (m, 8H). **^{13}C NMR** (101 MHz, D_2O) δ = 176.7, 175.1, 173.3, 167.5, 60.1, 58.5, 52.2, 46.9, 45.9, 29.5, 28.7, 27.3, 25.4, 23.5, 23.2. **HRMS** (ESI): *m/z* calcd for $C_{15}H_{25}N_4O_5^+$: 341.1819 [*M* + H]$^+$; found: 341.1819.

TFA·H-Pro-DPro-Glu-NH$_2$ (A-LDL)

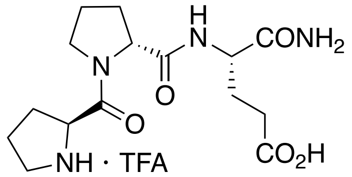

The analytical data are in agreement with previously published data.[2] **^{1}H NMR** (400 MHz, D_2O) δ = 4.69 – 4.60 (m, 1H), 4.48 (dd, J = 8.8, 4.3 Hz, 1H), 4.36 (dd, J = 9.8, 4.8 Hz, 1H), 3.75 (dt, J = 10.1, 6.3 Hz, 1H), 3.63 (dt, J = 10.4, 7.0 Hz, 1H), 3.50 – 3.36 (m, 2H), 2.62 – 2.53 (m, 1H), 2.48 (t, J = 7.2 Hz, 2H), 2.37 – 2.29 (m, 1H), 2.17 – 1.93 (m, 8H). **^{13}C NMR** (101 MHz, D_2O) δ = 176.8, 175.2, 173.5, 167.3, 60.2, 58.5, 52.2, 46.9, 45.8, 29.7, 28.7, 27.3, 25.3, 23.6, 23.2. **HRMS** (ESI): *m/z* calcd for $C_{15}H_{25}N_4O_5^+$: 341.1819 [*M* + H]$^+$; found: 341.1821.

TFA·H-DPro-DPro-Glu-NH$_2$ (A-DDL)

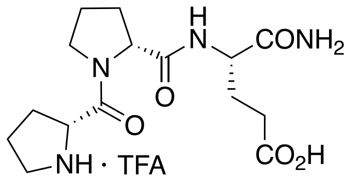

The analytical data are in agreement with previously published data.[2] **^{1}H NMR** (400 MHz, D_2O) δ = 4.65 (dd, J = 8.8, 6.3 Hz, 1H), 4.50 (dd, J = 8.1, 6.6 Hz, 1H), 4.38 (dd, J = 9.8, 4.7 Hz, 1H), 3.74 (ddd, J = 10.3, 7.2, 5.0 Hz, 1H), 3.61 (dt, J = 10.1, 7.2 Hz, 1H), 3.47 – 3.36 (m, 2H), 2.63 – 2.53 (m, 1H), 2.51 – 2.45 (m, 2H), 2.42 – 2.33 (m, 1H), 2.20 – 1.90 (m, 8H). **^{13}C NMR** (101 MHz, D_2O) δ = 176.5, 175.2, 173.3, 167.2, 60.0, 58.3, 51.9, 47.0, 45.8, 29.6, 28.6, 27.5, 25.4, 24.0, 23.1. **HRMS** (ESI): *m/z* calcd for $C_{15}H_{25}N_4O_5^+$: 341.1819 [*M* + H]$^+$; found: 341.1822.

TFA·H-Pro-Pro-Glu-NH$_2$ (A-LLL)

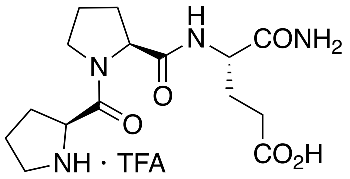

The analytical data are in agreement with previously published data.[1] **^{1}H NMR** (400 MHz, D_2O) δ = 4.65 (dd, J = 8.6, 6.2 Hz, 1H), 4.50 (dd, J = 8.3, 6.1 Hz, 1H), 4.34 (dd, J = 9.0, 5.5 Hz, 1H), 3.77 – 3.69 (m, 1H), 3.61 (dt, J = 10.4, 7.2 Hz, 1H), 3.49 – 3.37 (m, 2H), 2.64 – 2.55 (m, 1H), 2.53 (td, J = 7.1, 1.8 Hz, 2H), 2.41 – 2.31 (m, 1H), 2.14 – 1.91 (m, 8H). **^{13}C NMR** (101 MHz, D_2O) δ = 176.6, 175.1, 173.0, 167.3, 59.7, 58.4, 52.1, 46.9, 45.8, 29.3, 28.5, 27.6, 25.5, 23.9, 23.1. **HRMS** (ESI): *m/z* calcd for $C_{15}H_{25}N_4O_5^+$: 341.1819 [M + H]$^+$; found: 341.1824.

TFA·H-DPro-αMePro-Glu-NH$_2$ (B)

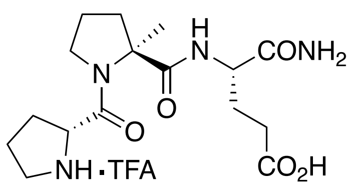

The analytical data are in agreement with previously published data.[3] **^{1}H NMR** (400 MHz, $CDCl_3/CD_3OH$ 9:1) δ = 8.83 (d, J = 7.0 Hz, 1H), 7.14 (s, 1H), 6.28 (s, 1H), 4.71 (dd, J = 8.9, 6.7 Hz, 1H), 4.35 (td, J = 7.2, 2.8 Hz, 1H), 3.92 (dt, J = 10.2, 6.2 Hz, 1H), 3.60 (dt, J = 10.0, 7.2 Hz, 1H), 3.54 (dt, J = 11.2, 6.8 Hz, 1H), 3.41 – 3.32 (m, 1H), 2.68 – 2.44 (m, 3H), 2.30 (q, J = 5.8 Hz, 1H), 2.24 – 2.14 (m, 2H), 2.13 – 1.82 (m, 6H), 1.59 (s, 3H). **^{13}C NMR** (101 MHz, $CDCl_3/CD_3OH$ 9:1) δ = 178.7, 174.8, 173.0, 168.5, 68.8, 59.4, 53.6, 48.7, 46.8, 39.4, 30.2, 29.1, 25.0, 24.8, 23.7, 21.2.

^{1}H NMR (400 MHz, D_2O) δ = 4.71 – 4.65 (m, 1H), 4.37 (dd, J = 10.0, 4.6 Hz, 1H), 3.82 (dt, J = 10.2, 6.5 Hz, 1H), 3.77 – 3.67 (m, 1H), 3.44 (qt, J = 11.5, 7.1 Hz, 2H), 2.64 – 2.45 (m, 3H), 2.29 – 1.90 (m, 10H), 1.57 (s, 3H). **^{13}C NMR** (101 MHz, D_2O) δ = 177.7, 176.3, 176.1, 168.2, 68.0, 59.6, 53.4, 48.4, 46.7, 38.8, 30.5, 28.2, 25.5, 23.9, 23.3, 19.9. **HRMS** (MALDI) m/z calcd for $C_{16}H_{27}N_4O_5^+$ 355.1976 [M + H]$^+$; found 355.1976.

TFA·H-DPro-Pip-Glu-NH$_2$ (C)

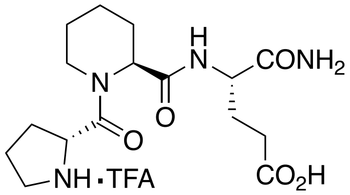

The analytical data are in agreement with previously published data.[4] **^{1}H NMR** (400 MHz, D_2O) δ = 5.08 (dd, J = 5.9, 2.8 Hz, 1H), 4.90 – 4.76 (m, 1H), 4.36 (dd, J = 9.2, 5.2 Hz, 1H), 3.84 – 3.69 (m, 1H), 3.55 – 3.35 (m, 2H), 3.35 – 3.18 (m, 1H), 2.56 – 2.46 (m, 3H), 2.29 – 1.93 (m, 6H), 1.93 – 1.63 (m, 4H), 1.63 – 1.28 (m, 2H). **^{13}C NMR** (101 MHz, D_2O) δ = 177.2, 175.9, 172.7, 169.8, 58.9, 54.2, 53.3, 46.5, 43.8, 30.1, 29.0, 25.82, 25.75, 23.95, 23.93, 19.5. **HRMS** (ESI): *m/z* calcd for $C_{16}H_{26}N_4O_5Na^+$: 377.1795 [M + Na]$^+$; found: 377.1798.

TFA·H-DPro-Pro-Asn-NH$_2$ (D)

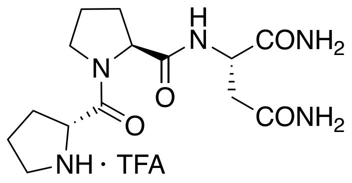

The analytical data are in agreement with previously published data.[5] **^{1}H NMR** (400 MHz, D_2O) δ = 4.70 (dd, J = 8.6, 5.5 Hz, 1H), 4.67 (dd, J = 8.6, 6.7 Hz, 1H), 4.48 (dd, J = 9.0, 3.8 Hz, 1H), 3.81 – 3.71 (m, 1H), 3.68 – 3.58 (m, 1H), 3.53 – 3.37 (m, 2H), 2.87 (dd, J = 15.5, 5.5 Hz, 1H), 2.77 (dd, J = 15.5, 8.6 Hz, 1H), 2.63 – 2.53 (m, 1H), 2.40 – 2.28 (m, 1H), 2.16 – 1.98 (m, 6H). **^{13}C NMR** (101 MHz, D_2O) δ = 174.1, 173.7, 173.1, 167.6, 60.2, 58.5, 49.6, 46.9, 45.9, 35.4, 28.7, 27.3, 23.4, 23.1. **HRMS** (ESI): *m/z* calcd for $C_{14}H_{24}N_5O_4^+$: 326.1823 [M + H]$^+$; found: 326.1824.

TFA·H-DPro-Pro-Gln-NH$_2$ (E)

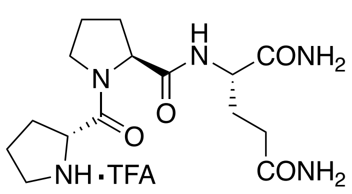

The analytical data are in agreement with previously published data.[5] **^{1}H NMR** (400 MHz, D_2O) δ = 4.67 (dd, J = 8.7, 7.1 Hz, 1H), 4.50 (dd, J = 8.9, 3.7 Hz, 1H), 4.32 (dd, J = 9.3, 5.2 Hz, 1H), 3.81 – 3.70 (m, 1H), 3.68 – 3.59 (m, 1H), 3.53 – 3.37 (m, 2H), 2.64 – 2.51 (m, 1H), 2.43 (t, J = 7.5 Hz, 2H), 2.39 – 2.30 (m, 1H), 2.21 – 1.98 (m, 8H). **^{13}C NMR** (101 MHz, D_2O) δ = 177.9, 175.8, 174.1, 168.3, 60.8, 59.3, 53.0, 47.7, 46.6, 31.2, 29.5, 28.1, 26.7, 24.3, 23.9. **HRMS** (ESI): *m/z* calcd for $C_{15}H_{25}N_5NaO_4^+$: 362.1799 [M + Na]$^+$; found: 362.1794.

TFA·H-DPro-Pro-Ala-NH$_2$ (F)

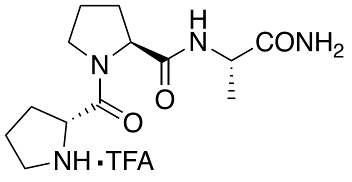

The analytical data are in agreement with previously published data.[5] **^{1}H NMR** (400 MHz, D_2O) δ = 4.63 (dd, J = 8.9, 6.8 Hz, 1H), 4.46 (dd, J = 8.7, 4.0 Hz, 1H), 4.28 (q, J = 7.3 Hz, 1H), 3.72 (dt, J = 10.1, 6.2 Hz, 1H), 3.61 (dt, J = 10.0, 6.9 Hz, 1H), 3.50 – 3.35 (m, 2H), 2.64 – 2.48 (m, 1H), 2.40 – 2.28 (m, 1H), 2.18 – 1.91 (m, 6H), 1.41 (d, J = 7.3 Hz, 3H). **^{13}C NMR** (101 MHz, D_2O δ = 177.7, 173.7, 168.1, 60.6, 59.2, 49.5, 47.7, 46.6, 29.5, 28.0, 24.2, 23.9, 16.5. **HRMS** (ESI): *m/z* calcd for $C_{13}H_{23}N_4O_3^+$: 283.1765 [M + H]$^+$; found: 283.1768.

TFA·H-DPro-Pro-βAla-NH₂ (G)

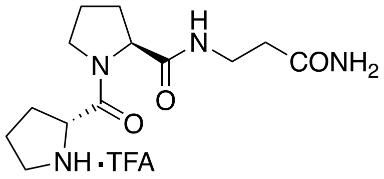

The analytical data are in agreement with previously published data.[5] **¹H NMR** (400 MHz, D_2O) δ = 4.63 (dd, J = 8.9, 6.8 Hz, 1H), 4.39 (dd, J = 8.9, 3.9 Hz, 1H), 3.72 (dt, J = 9.8, 6.1 Hz, 1H), 3.60 (dt, J = 10.3, 7.3 Hz, 1H), 3.54 – 3.34 (m, 4H), 2.59 – 2.51 (m, 1H), 2.49 (t, J = 6.6 Hz, 2H), 2.32 – 2.24 (m, 1H), 2.14 – 1.91 (m, 6H). **^{13}C NMR** (101 MHz, D_2O) δ = 176.8, 173.8, 168.0, 61.0, 59.2, 47.6, 46.6, 35.7, 34.5, 29.7, 28.0, 24.1, 23.9. **HRMS** (ESI): *m/z* calcd for $C_{13}H_{23}N_4O_3^+$: 283.1765 [M + H]$^+$; found: 283.1767.

TFA·H-DPro-Pro-Leu-NH₂ (H)

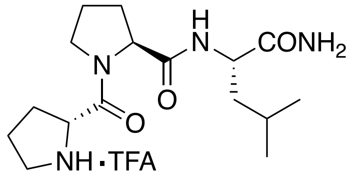

The analytical data are in agreement with previously published data.[5] **¹H NMR** (400 MHz, D_2O) δ = 4.64 (dd, J = 8.9, 6.7 Hz, 1H), 4.46 (dd, J = 9.1, 3.6 Hz, 1H), 4.31 (dd, J = 9.9, 4.9 Hz, 1H), 3.78 – 3.68 (m, 1H), 3.61 (dt, J = 10.3, 6.8 Hz, 1H), 3.50 – 3.35 (m, 2H), 2.61 – 2.50 (m, 1H), 2.40 – 2.27 (m, 1H), 2.20 – 1.95 (m, 6H), 1.75 – 1.63 (m, 2H), 1.63 – 1.54 (m, 1H), 0.92 (dd, J = 18.9, 6.1 Hz, 6H). **^{13}C NMR** (101 MHz, D_2O) δ = 177.4, 174.0, 168.3, 60.8, 59.3, 52.2, 47.6, 46.7, 39.6, 29.5, 28.1, 24.3, 24.2, 23.9, 22.1, 20.5. **HRMS** (ESI): *m/z* calcd for $C_{16}H_{29}N_4O_3^+$: 325.2234 [M + H]$^+$; found: 325.2237.

TFA·H-DPro-Pro-Phe-NH₂ (I)

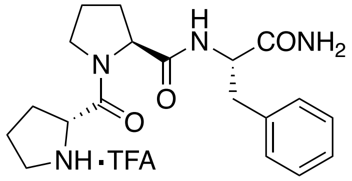

The analytical data are in agreement with previously published data.[5] **¹H NMR** (400 MHz, D_2O) δ = 7.44 – 7.27 (m, 5H), 4.62 (ddd, J = 9.0, 6.9, 3.5 Hz, 2H), 4.39 (dd, J = 9.0, 3.8 Hz, 1H), 3.72 – 3.61 (m, 1H), 3.61 – 3.50 (m, 1H), 3.50 – 3.30 (m, 2H), 3.20 (dd, J = 14.0, 6.5 Hz, 1H), 3.04 (dd, J = 13.9, 9.0 Hz, 1H), 2.54 (ddt, J = 13.0, 9.0, 6.4 Hz, 1H), 2.23 – 2.14 (m, 1H), 2.12 – 2.02 (m, 2H), 2.02 – 1.88 (m, 2H), 1.85 – 1.69 (m, 2H). **^{13}C NMR** (101 MHz, D_2O) δ = 175.6, 173.6, 168.5, 136.5, 129.1, 128.7, 127.1, 60.9, 59.3, 54.6, 47.6, 46.7, 36.6, 29.3, 28.0, 24.0, 23.9. **HRMS** (ESI): *m/z* calcd for $C_{19}H_{27}N_4O_3^+$: 359.2078 [M + H]$^+$; found: 359.2077.

2 TFA·H-DPro-Pro-His-NH$_2$ (J)

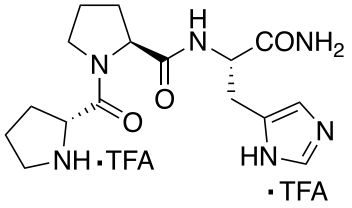

^{1}H NMR (400 MHz, D_2O) δ = 8.63 (s, 1H), 7.34 (s, 1H), 4.70 (dd, J = 8.7, 5.8 Hz, 1H), 4.62 (t, J = 8.0 Hz, 1H), 4.43 (dd, J = 8.8, 3.9 Hz, 1H), 3.70 (dt, J = 11.7, 4.7 Hz, 1H), 3.64 – 3.52 (m, 1H), 3.53 – 3.10 (m, 4H), 2.63 – 2.49 (m, 1H), 2.27 (dt, J = 13.0, 8.2 Hz, 1H), 2.20 – 1.82 (m, 6H). **^{13}C NMR** (101 MHz, D_2O) δ = 174.3, 174.3, 168.5, 133.9, 128.8, 117.6, 61.1, 59.6, 52.6, 48.0, 47.0, 29.9, 28.4, 26.6, 24.6, 24.2. **HRMS** (MALDI): *m/z* calcd. for [M + TFA + H]$^+$ $C_{18}H_{24}F_3N_6O_5^+$: 461.1763; found: 461.1763.

2 TFA·H-DPro-Pro-Arg-NH$_2$ (K)

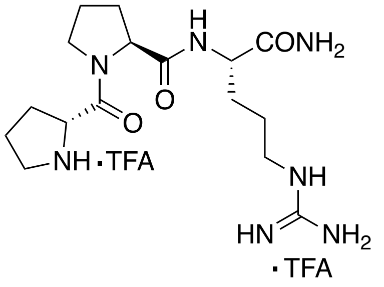

^{1}H NMR (400 MHz, D_2O) δ = 4.67 (dd, J = 8.9, 6.8 Hz, 1H), 4.49 (dd, J = 9.0, 3.7 Hz, 1H), 4.29 (dd, J = 8.9, 5.5 Hz, 1H), 3.76 (ddd, J = 12.3, 6.7, 3.9 Hz, 1H), 3.62 (dt, J = 10.0, 6.9 Hz, 1H), 3.53 – 3.33 (m, 2H), 3.32 – 3.19 (m, 2H), 2.66 – 2.50 (m, 1H), 2.47 – 2.27 (m, 1H), 2.21 – 1.57 (m, 10H). **^{13}C NMR** (101 MHz, D_2O) δ = 176.6, 174.4, 168.5, 157.0, 61.0, 59.5, 53.6, 48.0, 46.9, 40.7, 29.8, 28.3, 28.2, 24.7, 24.5, 24.2. **HRMS** (MALDI): *m/z* calcd. for [M + H]$^+$ $C_{16}H_{30}N_7O_3^+$: 368.2405; found: 368.2404.

TFA·H-DPro-Pro-Ser-NH$_2$ (L)

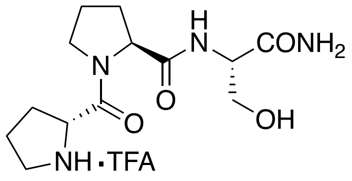

^{1}H NMR (400 MHz, D_2O) δ = 4.62 (dd, J = 8.9, 6.9 Hz, 1H), 4.50 (dd, J = 8.6, 3.8 Hz, 1H), 4.41 (t, J = 5.2 Hz, 1H), 3.87 (dd, J = 5.3, 1.7 Hz, 2H), 3.80 – 3.66 (m, 1H), 3.67 – 3.54 (m, 1H), 3.51 – 3.28 (m, 2H), 2.64 – 2.42 (m, 1H), 2.42 – 2.24 (m, 1H), 2.05 (m, 6H). **^{13}C NMR** (101 MHz, D_2O) δ = 174.1, 174.1, 168.2, 61.0, 60.8, 59.2, 55.4, 47.7, 46.6, 29.5, 28.0, 24.2, 23.8. **HRMS** (MALDI): *m/z* calcd. for [M + H]$^+$ $C_{13}H_{23}N_4O_4^+$: 299.1714; found: 299.1714.

TFA·H-DPro-Pro-Tyr-NH₂ (M)

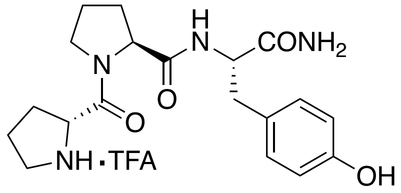

^{1}H NMR (400 MHz, D_2O) δ = 7.21 – 7.13 (m, 2H), 6.91 – 6.82 (m, 2H), 4.67 – 4.60 (m, 1H), 4.57 (dd, J = 9.0, 6.5 Hz, 1H), 4.40 (dd, J = 9.0, 3.7 Hz, 1H), 3.67 (ddd, J = 10.0, 7.3, 4.5 Hz, 1H), 3.62 – 3.51 (m, 1H), 3.51 – 3.31 (m, 2H), 3.19 – 3.06 (m, 1H), 2.96 (dd, J = 14.0, 9.1 Hz, 1H), 2.55 (ddt, J = 13.0, 9.1, 6.5 Hz, 1H), 2.35 – 1.66 (m, 1H). **^{13}C NMR** (101 MHz, D_2O) δ = 175.7, 173.6, 168.5, 154.4, 130.4, 128.3, 115.4, 60.9, 59.3, 54.7, 47.6, 46.7, 35.8, 29.3, 28.0, 23.9, 23.9. **HRMS** (MALDI): *m/z* calcd. for $[M + H]^+$ $C_{19}H_{27}N_4O_4^+$: 375.2027; found: 375.2026.

3 Optimization of the Reaction Conditions

Table S1: Initial screening of H-Pro-Xaa-Glu-NH2 catalysts and the catalyst loading in the conjugate addition reaction between racemic 3-phenylbutanal (*rac*-**1a**) and nitrostyrene (**2a**).

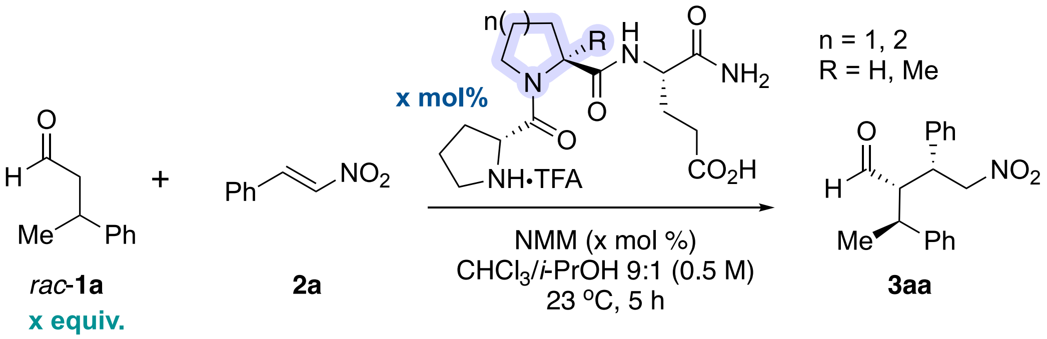

Entry	Peptide	mol %	1.5 equiv. Conv. (%)[a]	1.5 equiv. d.r.[a]	1.5 equiv. ee (%)[b]	3 equiv. Conv. (%)[a]	3 equiv. d.r.[a]	3 equiv. ee (%)[b]
1	pPE (**A**)	1	39	86:11:1:2	95	50	90:6:1:3	94
2	pPE (**A**)	2	64	88:8:1:3	93	80	87:8:1:3	93
3	pPE (**A**)	3	78	88:8:1:3	92	85	90:6:1:3	93
4	pαMePE (**B**)	1	4	93:3:1:3	94	28	93:2:2:3	97
5	pαMePE (**B**)	2	24	89:7:1:3	96	63	90:6:1:3	95
6	pαMePE (**B**)	3	48	90:5:1:4	96	71	92:4:1:3	96
7	pPipE (**C**)	1	66	80:18:1:1	96	53	85:14:<1:<1	96
8	pPipE (**C**)	2	70	75:23:1:1	95	89	79:20:<1:<1	96
9	pPipE (**C**)	3	95	75:23:1:1	94	>95	81:18:<1:<1	95

[a] Determined by ^1H NMR spectroscopy of the crude reaction mixture. [b] Determined by chiral stationary phase HPLC analysis.

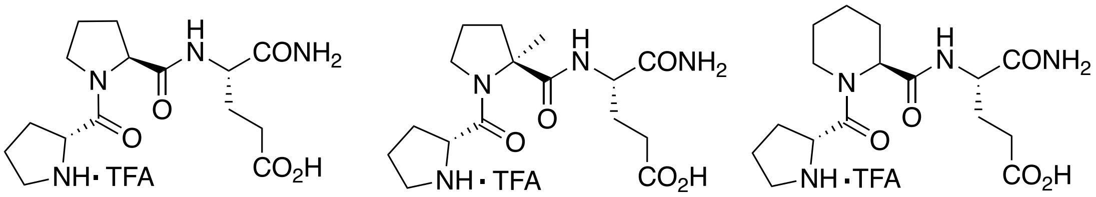

A **B** **C**

Table S2: Catalyst screening for the conjugate addition reaction between racemic 3-phenylbutanal (*rac*-**1a**) and nitrostyrene (**2a**).

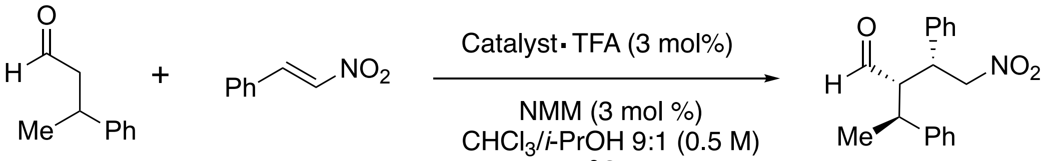

rac-**1a**
3 equiv.

2a

3aa

**Entry**	**Catalyst**	**Conv. (%)**[a]	**d.r.**[a]	**ee (%)**[b]
1	H-DPro-Pro-Glu-NH2 (**A-DLL**)	85	90:6:1:3	93
2	H-Pro-DPro-Glu-NH2 (**A-LDL**)	52	83:14:2:1	63[c]
3	H-DPro-DPro-Glu-NH2 (**A-DDL**)	14	89:9:2:<1	60
4	H-Pro-Pro-Glu-NH2 (**A-LLL**)	49	81:16:2:1	50[c]
5	H-DPro-αMePro-Glu-NH2 (**B**)	71	92:4:3:1	96
6	H-DPro-Pip-Glu-NH2 (**C**)	>95	81:18:<1:<1	95
7	H-DPro-Pro-Asn-NH2 (**D**)	20	81:11:2:6	78
8	H-DPro-Pro-Gln-NH2 (**E**)	21	83:6:9:2	85
9	H-DPro-Pro-Ala-NH2 (**F**)	10	76:18:4:2	80
10	H-DPro-Pro-βAla-NH2 (**G**)	20	77:14:7:2	83
11	H-DPro-Pro-Leu-NH2 (**H**)	<5	n.d.	n.d.
12	H-DPro-Pro-Phe-NH2 (**I**)	<5	n.d.	n.d.
13	H-DPro-Pro-His-NH2 (**J**)	<5	n.d.	n.d.
14	H-DPro-Pro-Arg-NH2 (**K**)	<5	n.d.	n.d.
15	H-DPro-Pro-Ser-NH2 (**L**)	<5	n.d.	n.d.
16	H-DPro-Pro-Tyr-NH2 (**M**)	32	77:16:5:2	78

[a] Determined by ^1H NMR spectroscopy of the crude reaction mixture. [b] Determined by chiral stationary phase HPLC analysis. [c] *ent*-**3aa** was the major enantiomer.

Table S3: Solvent screening for the conjugate addition reaction between 3-phenylbutanal (*rac*-**1a**) and nitrostyrene (**2a**) catalyzed by H-DPro-Pro-Glu-NH_2 (**A**).

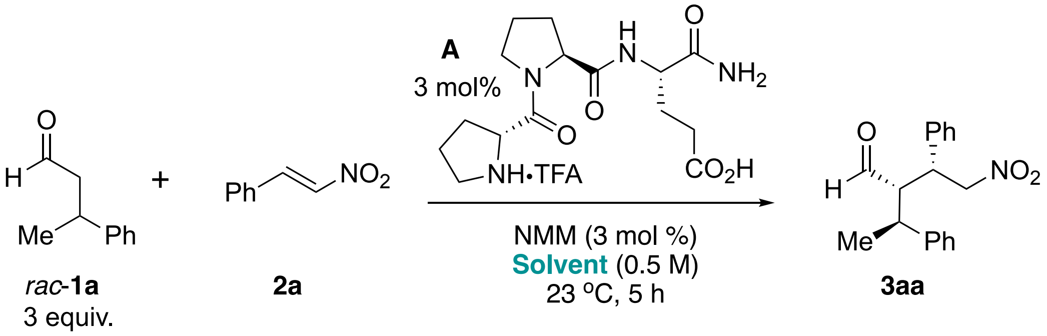

Entry	Solvent	Conv. (%)[a]	d.r.[a]	ee (%)[b]
1	$CHCl_3$/*i*-PrOH 9:1	85	90:6:1:3	93
2	$CHCl_3$	65	90:6:1:3	91
3	*i*-PrOH	>95	78:15:3:4	83
4	MeOH	53	72:19:4:5	67
5	MeCN	>95	81:12:2:5	77
6	DMSO	19	74:15:8:3	55
7	1,4-Dioxane	33	84:10:3:3	90
8	THF	20	86:20:2:2	83
9	Toluene	62	90:6:2:2	84

[a] Determined by ^{1}H NMR spectroscopy of the crude reaction mixture. [b] Determined by chiral stationary phase HPLC analysis.

Table S4: Optimization of aldehyde stoichiometry and temperature in the conjugate addition reaction between 3-phenylbutanal (*rac*-**1a**) and nitrostyrene (**2a**) catalyzed by H-DPro-αMePro-Glu-NH_2 (**B**).

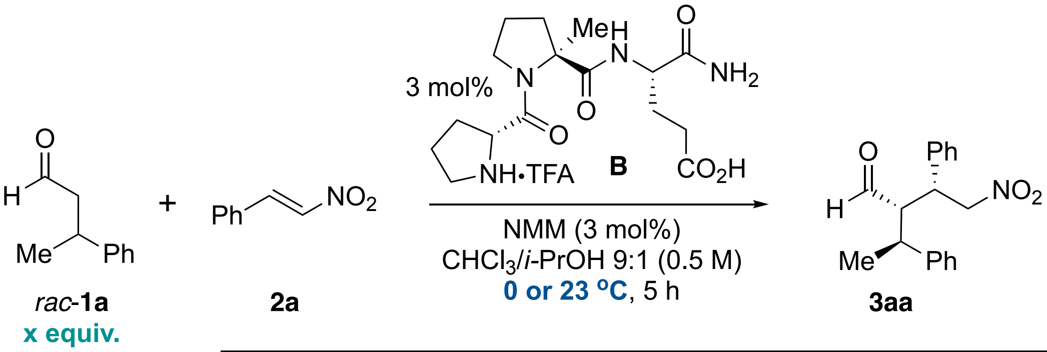

		0 °C			23 °C		
**Entry**	**x equiv.**	**Conv. (%)[a]**	**d.r.[a]**	**ee (%)[b]**	**Conv. (%)[a]**	**d.r.[a]**	**ee (%)[b]**
1	**2**	55	93:5:1:1	97	50	87:9:1:3	96
2	**2.5**	60	95:3:1:1	97	59	89:7:1:3	96
3	**3**	55	95:3:1:1	97	71	92:4:1:3	96
4[c]	**2.5**	>95	92:6:1:1	97	>95	90:6:1:3	95

[a] Determined by ^{1}H NMR spectroscopy of the crude reaction mixture. [b] Determined by chiral stationary phase HPLC analysis. [c] Reaction time 24 h.

Table S5: Optimization of the kinetic resolution of 3-phenylbutanal (*rac*-**1a**) to (*R*)-3-phenylbutanal.

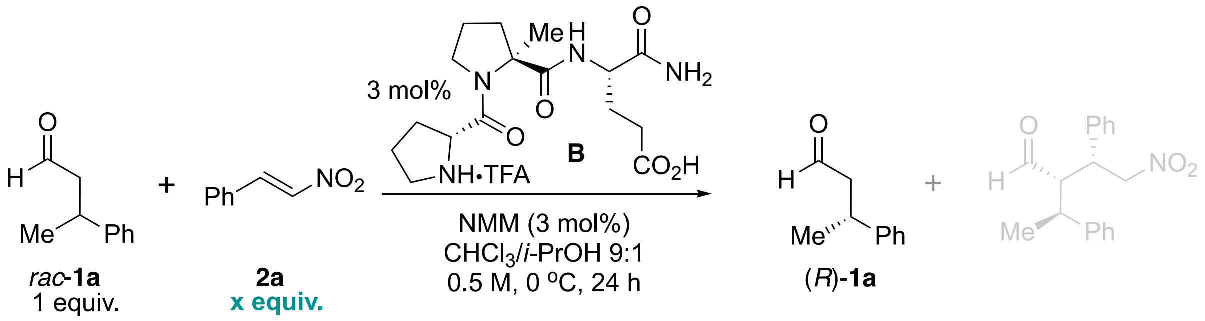

Entry	x equiv.	Remaining **1a** (%)[a]	ee (%)[b]
1	1	51	85
2	1.2	48	92
3	1.5	45	99
4	2	30	94

[a] The amount of remaining enantioenriched aldehyde **1a** was determined by ^{1}H NMR spectroscopy analysis of the crude reaction mixture. [b] Enantioselectivity was determined by chiral stationary phase SFC analysis.

Table S6: Catalyst loading screening in the kinetic resolution of 3-phenylbutanal (*rac*-**1a**).

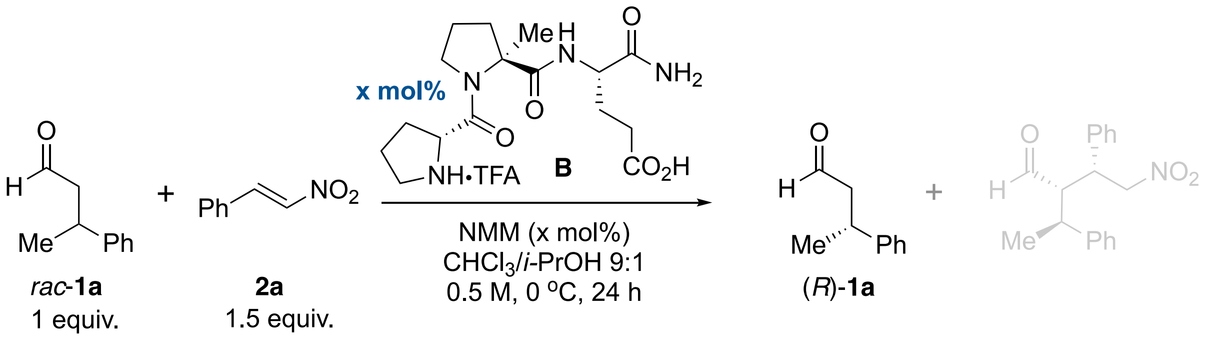

Entry	x mol%	Remaining **1a** (%)[a]	ee (%)[b]
1	3	45	99
2	2	46	98
3	1	50	90

[a] The amount of remaining enantioenriched aldehyde **1a** was determined by ^{1}H NMR spectroscopy analysis of the crude reaction mixture. [b] Enantioselectivity was determined by chiral stationary phase SFC analysis.

4 Synthesis and Analytical Data of Racemic β-Branched Aldehydes

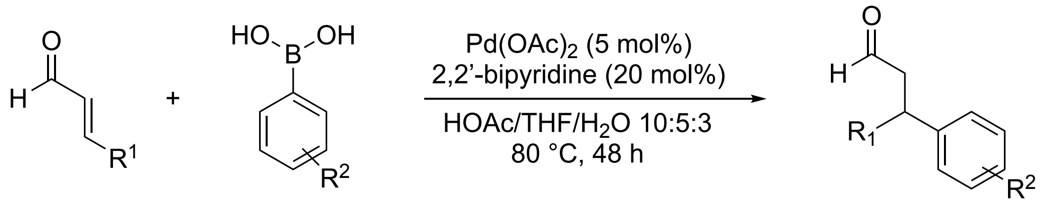

4 1 equiv. **5** 3 equiv. **1** racemic

General procedure I[6]**:** The α,β-unsaturated aldehyde **4** (1 equiv., 10 mmol, 0.6 mM) was added to a solution of boronic acid **5** (3 equiv., 30 mmol) and 2,2'-bipyridine (20 mol%, 2 mmol) in HOAc/THF/H_2O 10:5:3 under a nitrogen atmosphere. Palladium(II) acetate (5 mol%, 0.5 mmol) was added, and the reaction mixture was stirred for 48 h at 80 °C. After cooling to room temperature, a saturated aqueous solution of $NaHCO_3$ (ca. 200 mL) was added until pH 7. The solution was extracted with Et_2O (3 x ca. 300 mL). The combined organic layers were dried over $MgSO_4$, filtered and carefully concentrated in vacuo at 25 °C (note that some of the aldehydes are volatile). The crude orange oil was purified by column flash chromatography using *n*-hexane/EtOAc or hexane/CH_2Cl_2.

3-Phenylbutanal (1a):

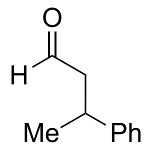

Aldehyde **1a** (CAS 16251-77-7) was purchased from a commercial supplier (Sigma Aldrich) and used as received.

3-(*p*-Tolyl)butanal (1b)

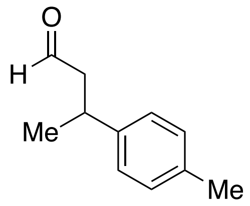

Following general procedure **I**, **1b** was obtained as a colorless liquid (0.56 g, 35% yield). **R*f*** = 0.25 (*n*-hexane/CH_2Cl_2 50:50). **^{1}H NMR** (400 MHz, $CDCl_3$) δ = 9.70 (t, J = 2.1 Hz, 1H), 7.12 (s, 4H), 3.33 (dt, J = 7.7, 6.9 Hz, 1H), 2.79 – 2.59 (m, 2H), 2.32 (s, 3H), 1.30 (d, J = 7.0 Hz, 3H). **^{13}C NMR** (101 MHz, $CDCl_3$) δ = 202.2, 142.6, 136.2, 129.5, 126.8, 51.9, 34.1, 22.4, 21.1. **IR** (ATR): 3019, 2961, 2923. 2873, 2820, 2719, 1720, 1513, 1452, 1410, 1376, 1108, 814 cm^{-1}. **HRMS** (ESI) m/z calcd for $C_{11}H_{18}NO^+$ 180.1383 $[M + NH_4]^+$; found: 180.1379.

3-(4-(*tert*-Butyl)phenyl)butanal (1c)

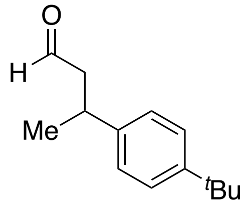

Following general procedure **I**, **1c** was obtained as a colorless liquid (1.41 g, 69% yield). $\mathbf{R_f}$ = 0.50 (*n*-hexane/EtOAc, 90:10). ^{1}**H NMR** (400 MHz, $CDCl_3$) δ = 9.72 (dd, J = 1.9 Hz, 1H), 7.36 – 7.30 (m, 2H), 7.18 – 7.12 (m, 2H), 3.34 (h, J = 7.0 Hz, 1H), 2.80 – 2.58 (m, 2H), 1.35 – 1.25 (m, 12H). ^{13}**C NMR** (101 MHz, $CDCl_3$) δ = 202.3, 149.5, 142.5, 126.5, 125.7, 51.9, 34.5, 33.9, 31.5, 22.3. **IR** (ATR): 2959, 2903, 2869, 2821, 2716, 1722, 1509, 1458, 1406, 1394, 1363, 1268, 1241, 1202, 1115, 1050, 1016, 829 cm^{-1}. **HRMS** (EI) m/z calcd for $C_{14}H_{20}O^+$ 204.1509 [*M*]$^+$; found: 204.1505.

3-(4-Methoxyphenyl)butanal (1d)

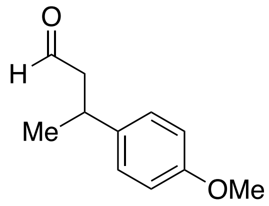

Following general procedure **I**, **1d** was obtained as a colorless liquid (1.64 g, 94% yield). $\mathbf{R_f}$ = 0.28 (*n*-hexane/EtOAc, 90:10). ^{1}**H NMR** (400 MHz, $CDCl_3$) δ = 9.70 (t, J = 2.1 Hz, 1H), 7.17 – 7.11 (m, 2H), 6.88 – 6.82 (m, 2H), 3.79 (s, 3H), 3.32 (h, J = 7.1 Hz, 1H), 2.76 – 2.55 (m, 2H), 1.29 (d, J = 7.0 Hz, 3H). ^{13}**C NMR** (101 MHz, $CDCl_3$) δ = 202.2, 158.3, 137.7, 127.8, 114.2, 55.4, 52.1, 33.7, 22.5. **IR** (ATR): 2959, 2834, 2720, 1719, 1610, 1510, 1458, 1243, 1177, 1029, 828 cm^{-1}. **HRMS** (ESI) m/z calcd for $C_{11}H_{14}NaO_2^+$ 201.0886 [M + Na]$^+$; found: 201.0889.

3-(4-(Trifluoromethyl)phenyl)butanal (1e)

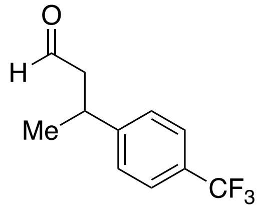

Following general procedure **I** (3 mmol scale), **1e** was obtained as a colorless liquid (0.65 g, 71% yield). $\mathbf{R_f}$ = 0.24 (*n*-hexane/EtOAc, 90:10). ^{1}**H NMR** (400 MHz, $CDCl_3$) δ = 9.72 (t, J = 1.7 Hz, 1H), 7.59 – 7.53 (m, 2H), 7.37 – 7.31 (m, 2H), 3.44 (h, J = 7.1 Hz, 1H), 2.84 – 2.65 (m, 2H), 1.33 (d, J = 7.0 Hz, 3H). ^{13}**C NMR** (101 MHz, $CDCl_3$) δ = 201.0, 149.7 (q, J = 1.4 Hz), 129.0 (q, J = 32.3 Hz), 127.3, 125.8 (q, J = 3.9 Hz), 124.3 (q, J = 123.0 Hz), 51.6, 34.1, 22.1. ^{19}**F NMR** (376 MHz, $CDCl_3$) δ = –62.45 (s). **IR** (ATR): 2968, 2825, 2726, 1724, 1618, 1420, 1322, 1161, 1114, 1066, 1015, 837 cm^{-1}. **HRMS** (EI) m/z calcd for $C_{11}H_{11}F_3O^+$ 216.0757 [*M*]$^+$; found: 216.07585.

Methyl 4-(4-oxobutan-2-yl)benzoate (1f)

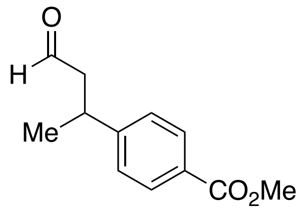

Following general procedure **I** (5 mmol scale), **1f** was obtained as a light-yellow liquid (0.98 g, 95% yield). $\mathbf{R_f}$ = 0.42 (*n*-hexane/EtOAc, 70:30). ^{1}**H NMR** (400 MHz, $CDCl_3$) δ = 9.71 (t, *J* = 1.8 Hz, 1H), 7.98 (d, *J* = 8.4 Hz, 2H), 7.31 – 7.27 (m, 2H), 3.90 (s, 3H), 3.43 (h, *J* = 7.0 Hz, 1H), 2.82 – 2.63 (m, 2H), 1.33 (d, *J* = 7.0 Hz, 3H). ^{13}**C NMR** (101 MHz, $CDCl_3$) δ = 201.2, 167.0, 151.0, 130.2, 128.7, 127.0, 52.2, 51.6, 34.3, 22.0. **IR** (ATR): 2955, 2835, 2723, 1713, 1609, 1434, 1274, 1181, 1108, 1017, 964, 856, 772, 706 cm^{-1}. **HRMS** (ESI) m/z calcd for $C_{12}H_{14}NaO_3^+$ 229.0835 [*M* + Na]$^+$; found: 229.0834.

3-(4-Fluorophenyl)butanal (1g)

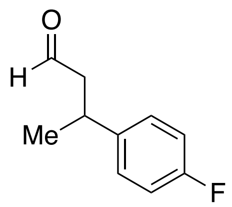

Following general procedure **I** (50 mmol scale), **1g** was obtained as a light-yellow liquid (5.7 g, 69% yield). $\mathbf{R_f}$ = 0.48 (*n*-hexane/EtOAc, 90:10). ^{1}**H NMR** (400 MHz, $CDCl_3$) δ = 9.70 (t, *J* = 1.9 Hz, 1H), 7.22 – 7.14 (m, 2H), 7.03 – 6.95 (m, 2H), 3.36 (h, *J* = 7.1 Hz, 1H), 2.78 – 2.53 (m, 2H), 1.30 (d, *J* = 7.0 Hz, 3H). ^{13}**C NMR** (101 MHz, $CDCl_3$) δ = 201.7, 161.6 (d, *J* = 244.5 Hz), 141.3 (d, *J* = 3.2 Hz), 128.3 (d, *J* = 7.9 Hz), 115.6 (d, *J* = 21.2 Hz), 52.0, 33.7, 22.5. ^{19}**F NMR** (376 MHz, $CDCl_3$) δ = –116.62 (tt, *J* = 8.7, 5.4 Hz). **IR** (ATR): 2964, 2877, 2823, 2723, 1720, 1602, 1508, 1455, 1220, 1159, 1096, 832 cm^{-1}. **HRMS** (ESI) m/z calcd for $C_{10}H_{11}FNaO^+$ 189.0686 [*M* + Na]$^+$; found: 189.0682.

3-(3-Fluorophenyl)butanal (1h)

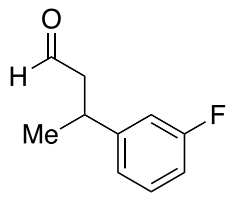

Following general procedure **I**, **1h** was obtained as a colorless liquid (1.46 g, 88% yield). $\mathbf{R_f}$ = 0.48 (*n*-hexane/EtOAc, 90:10). ^{1}**H NMR** (400 MHz, $CDCl_3$) δ = 9.71 (t, *J* = 1.8 Hz, 1H), 7.30 – 7.23 (m, 1H), 7.00 (dddd, *J* = 7.7, 1.6, 1.0, 0.5 Hz, 1H), 6.95 – 6.87 (m, 2H), 3.37 (h, *J* = 7.1 Hz, 1H), 2.80 – 2.61 (m, 2H), 1.31 (d, *J* = 7.0 Hz, 3H). ^{13}**C NMR** (101 MHz, $CDCl_3$) δ = 201.4, 163.2 (d, *J* = 245.8 Hz), 148.3 (d, *J* = 6.8 Hz), 130.3 (d, *J* = 8.4 Hz), 122.6 (d, *J* = 2.9 Hz), 113.8 (d, *J* = 21.2 Hz), 113.5 (d, *J* = 21.1 Hz), 51.7, 34.1 (d, *J* = 1.8 Hz), 22.1. ^{19}**F NMR** (376 MHz, $CDCl_3$) δ = –112.92 (ddd, *J* = 10.1, 8.7, 6.0 Hz). **IR** (ATR): 2966, 2878, 2824, 2723, 1721, 1588, 1486, 1447, 1241, 1149, 910, 870, 783 cm^{-1}. **HRMS** (ESI) m/z calcd for $C_{10}H_{11}FNaO^+$ 189.0686 [*M* + Na]$^+$; found: 189.0684.

3-(2-Fluorophenyl)butanal (1i)

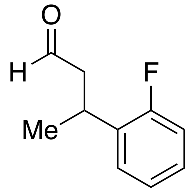

Following general procedure **I** (5 mmol scale), **1i** was obtained as a yellow liquid (0.33 g, 39% yield). **R_f** = 0.24 (*n*-hexane/EtOAc, 90:10). **^{1}H NMR** (400 MHz, $CDCl_3$) δ = 9.71 (t, *J* = 1.8 Hz, 1H), 7.30 – 7.22 (m, 1H), 7.00 (dddd, *J* = 7.7, 1.5, 1.0, 0.5 Hz, 1H), 6.95 – 6.86 (m, 2H), 3.44 – 3.22 (m, 1H), 2.81 – 2.52 (m, 2H), 1.31 (d, *J* = 7.0 Hz, 3H). **^{13}C NMR** (101 MHz, $CDCl_3$) δ = 201.4, 163.2 (d, *J* = 245.9 Hz), 148.3 (d, *J* = 6.7 Hz), 130.3 (d, *J* = 8.4 Hz), 122.6 (d, *J* = 2.7 Hz), 113.8 (d, *J* = 21.3 Hz), 113.6 (d, *J* = 21.1 Hz), 51.7, 34.1 (d, *J* = 1.7 Hz), 22.1. **^{19}F NMR** (376 MHz, $CDCl_3$) δ = –118.18 (ddd, *J* = 10.1, 8.7, 6.1 Hz). **IR** (ATR): 2968, 2878, 2822, 2722, 1721, 1490, 1451, 1224, 1114, 1083, 1030, 940, 824, 753 cm^{-1}. **HRMS** (ESI) m/z calcd for $C_{10}H_{11}FNaO^+$ 189.0686 $[M + Na]^+$; found: 189.0686.

3-(5-Methyl-2-furyl)butyraldehyde (1j)

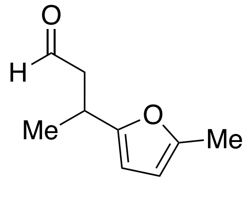

Aldehyde **1j** (CAS 31704-80-0) was purchased from a commercial supplier (ABCR) and used as received.

3-(4-Fluorophenyl)pentanal (1k)

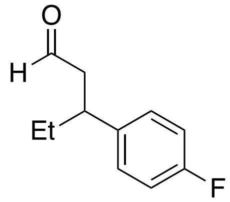

Following general procedure **I** (5 mmol scale), **1k** was obtained as a light-yellow liquid (0.62 g, 69% yield). **R_f** = 0.37 (*n*-hexane/EtOAc, 90:10). **^{1}H NMR** (400 MHz, $CDCl_3$) δ = 9.66 (t, *J* = 2.0 Hz, 1H), 7.17 – 7.11 (m, 2H), 7.02 – 6.95 (m, 2H), 3.08 (tt, J = 8.9, 6.2 Hz, 1H), 2.73 – 2.68 (m, 2H), 1.78 – 1.51 (m, 2H), 0.79 (t, *J* = 7.4 Hz, 3H). **^{13}C NMR** (101 MHz, $CDCl_3$) δ = 201.9, 161.7 (d, *J* = 245 Hz), 139.4 (d, *J* = 3.5 Hz), 129.0 (d, *J* = 7.8 Hz), 115.5 (d, *J* = 21.2 Hz), 50.5, 41.1, 29.7, 12.0. **^{19}F NMR** (377 MHz, $CDCl_3$) δ = –116.56 (tt, *J* = 8.7, 5.4 Hz). **IR** (ATR): 2963, 2928, 2876, 2824, 2722, 1721, 1602, 1508, 1460, 1220, 1159, 1096, 1014, 830 cm^{-1}. **HRMS** (ESI) m/z calcd for $C_{11}H_{13}FNaO^+$ 203.0843 $[M + Na]^+$; found: 203.0840.

3-(4-Fluorophenyl)hexanal (1l)

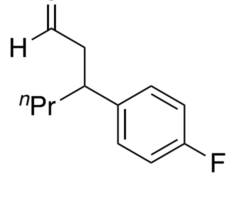

Following general procedure **I**, **1l** was obtained as a yellow liquid (1.2 g, 60% yield). **R_f** = 0.52 (*n*-hexane/EtOAc, 90:10). **^{1}H NMR** (400 MHz, $CDCl_3$) δ = 9.66 (t, *J* = 2.0 Hz, 1H), 7.18 – 7.10 (m, 2H), 7.02 – 6.94 (m, 2H), 3.18 (dtd, *J* = 9.0, 7.3, 5.8 Hz, 1H), 2.73 – 2.51 (m, 2H), 1.69 – 1.49 (m, 2H), 1.27 – 1.08 (m, 2H), 0.89 – 0.81 (m, 3H). **^{13}C NMR** (101 MHz, $CDCl_3$) δ = 201.9, 161.6 (d, *J* = 244.5 Hz), 139.7 (d, *J* = 3.3 Hz), 129.0 (d, *J* = 7.8 Hz), 115.5 (d, *J* = 21.2 Hz), 50.9, 39.2, 39.0, 20.5, 14.0. **^{19}F NMR** (377 MHz, $CDCl_3$) δ = –116.56 (tt, J = 8.7, 5.3 Hz).

IR (ATR): 2957, 2929, 2872, 2722, 1721, 1602, 1508, 1461, 1409, 1220, 1159, 1093, 831, 737 cm^{-1}. **HRMS** (EI) m/z calcd for $C_{11}H_{15}FO^+$ 194.1101 [*M*]$^+$; found: 194.1101.

3-(4-Fluorophenyl)dodecanal (1m)

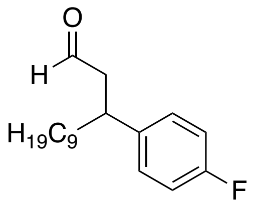

Following general procedure **I**, **1n** was obtained as a yellow liquid (1.5 g, 54% yield). **R*f*** = 0.51 (*n*-hexane/CH_2Cl_2 50:50). **^{1}H NMR** (400 MHz, $CDCl_3$) *δ* = 9.65 (t, *J* = 2.0 Hz, 1H), 7.18 – 7.10 (m, 2H), 7.02 – 6.94 (m, 2H), 3.15 (dtd, *J* = 9.1, 7.2, 5.6 Hz, 1H), 2.71 – 2.66 (m, 2H), 1.70 – 1.49 (m, 2H), 1.42 – 1.03 (m, 14H), 0.91 – 0.80 (m, 3H). **^{13}C NMR** (101 MHz, $CDCl_3$) *δ* = 201.8, 161.6 (d, *J* = 244 Hz), 139.8 (d, *J* = 3.2 Hz), 129.0 (d, *J* = 8.7 Hz), 115.5 (d, *J* = 21.5 Hz), 50.9, 39.5, 36.8, 32.0, 29.7, 29.6, 29.6, 29.4, 27.4, 22.8, 14.2. **^{19}F NMR** (376 MHz, $CDCl_3$) *δ* = –116.57 (tt, *J* = 8.7, 5.4 Hz). **IR** (ATR) 2922, 2852, 2717, 1723, 1603, 1509, 1463, 1415, 1223, 1158, 832, 723 cm^{-1}. **HRMS** (EI) m/z calcd for $C_{18}H_{27}FO^+$ 278.2040 [*M*]$^+$; found: 278.2038.

3-(4-Fluorophenyl)-4-methylpentanal (1n)

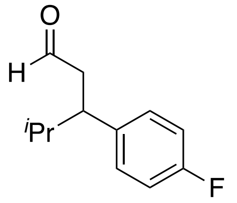

Following general procedure **I**, **1m** was obtained as a colorless liquid. (0.96 g, 49% yield). **R*f*** = 0.57 (*n*-hexane/EtOAc, 90:10). **^{1}H NMR** (400 MHz, $CDCl_3$) *δ* = 9.60 (dd, *J* = 2.5, 1.7 Hz, 1H), 7.15 – 7.07 (m, 2H), 7.02 – 6.93 (m, 2H), 2.96 (ddd, *J* = 9.8, 7.5, 5.2 Hz, 1H), 2.85 – 2.66 (m, 2H), 1.83 (dp, *J* = 7.4, 6.6 Hz, 1H), 0.94 (d, *J* = 6.7 Hz, 3H), 0.76 (d, *J* = 6.7 Hz, 3H). **^{13}C NMR** (101 MHz, $CDCl_3$) *δ* = 202.2, 161.7 (d, *J* = 245 Hz), 138.4 (d, *J* = 3.8 Hz), 129.7 (d, *J* = 7.7 Hz), 115.3 (d, *J* = 22.1 Hz), 47.5, 46.2, 33.6, 20.7, 20.3. **^{19}F NMR** (376 MHz, $CDCl_3$) *δ* = –116.57 (tt, *J* = 8.7, 5.4 Hz). **IR** (ATR): 2961, 2932, 2898, 2873, 2822, 2722, 1721, 1602, 1508, 1467, 1387, 1221, 1159, 1125, 1031, 825, 728 cm^{-1}. **HRMS** (ESI) m/z calcd for $C_{11}H_{15}FO^+$ 194.1101 [*M*]$^+$; found: 194.1105.

Methyl 4-(1-oxopentan-3-yl)benzoate (1o)

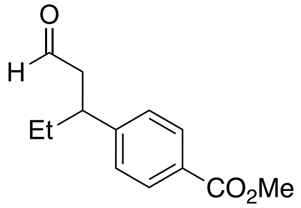

Following general procedure **I**, **1o** was obtained as a light yellow liquid (1.0 g, 45% yield). **R*f*** = 0.40 (*n*-hexane/EtOAc, 70:30). **^{1}H NMR** (400 MHz, $CDCl_3$) *δ* = 9.67 (dt, *J* = 2.9, 1.4 Hz, 1H), 8.29 – 7.85 (m, 2H), 7.40 – 7.20 (m, 2H), 3.90 (s, 3H), 3.26 – 3.07 (m, 1H), 2.78 – 2.72 (m, 2H), 1.83 – 1.54 (m, 2H), 0.80 (td, *J* = 7.4, 1.0 Hz, 3H). **^{13}C NMR** (101 MHz, $CDCl_3$) *δ* = 201.3, 167.1, 149.3, 130.1, 128.8, 127.8, 52.2, 50.1, 41.7, 29.4, 12.0. **IR** (ATR): 2959, 2930, 2723, 1714, 1608, 1434, 1310, 1274, 1180, 1109, 1017, 771, 707 cm^{-1}. **HRMS** (ESI) m/z calcd for $C_{13}H_{16}NaO_3^+$ 243.0992 [*M* + Na]$^+$; found: 243.0988.

3,5,5-Trimethylhexanal (1p)

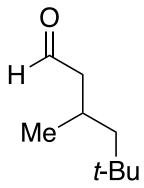

Aldehyde **1p** (CAS 5435-64-3) was purchased from a commercial supplier (Sigma Aldrich) and used as received.

7-Hydroxy-3,7-dimethyl-octanal (1q)

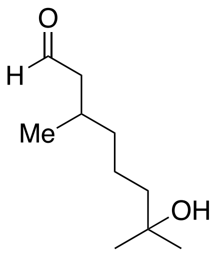

Aldehyde **1q** (CAS 107-75-5) was purchased from a commercial supplier (Acros Organics) and used as received.

Some enantiomeric aldehydes could not be separated by chiral stationary phase SFC or HPLC and were therefore reduced to the respective alcohol using the following procedure:

General Procedure II – reduction of β-branched aldehyde: The β-branched aldehyde (50 mg, 1 equiv.) was dissolved in MeOH (1 mL) and cooled to 0 °C. $NaBH_4$ (2 equiv.) was added, the reaction was allowed to warm up to room temperature and stirred for 2 h. The reaction was quenched with a saturated solution of NH_4Cl, the solution was extracted with EtOAc (3x) and the combined organic layers were dried over $MgSO_4$, filtered and concentrated in vacuo. The crude product was purified by flash chromatography (*n*-hexane/EtOAc or *n*-hexane/CH_2Cl_2).

Methyl 4-(4-hydroxybutan-2-yl)benzoate (1f')

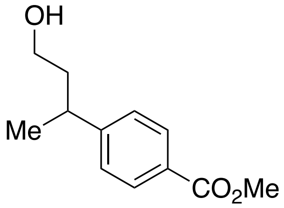

Following general procedure **II**, **1f'** was obtained as a yellow oil (35 mg, 69% yield). **R*f*** = 0.15 (*n*-hexane/EtOAc, 50:50). **^{1}H NMR** (400 MHz, $CDCl_3$) *δ* = 7.99 – 7.93 (m, 2H), 7.28 – 7.26 (m, 1H), 7.26 – 7.24 (m, 1H), 3.89 (s, 3H), 3.61 – 3.43 (m, 2H), 2.96 (dt, *J* = 7.9, 6.8 Hz, 1H), 1.94 – 1.76 (m, 2H), 1.59 (br s, 1H), 1.27 (d, *J* = 7.0 Hz, 3H). **^{13}C NMR** (101 MHz, $CDCl_3$) *δ* = 167.3, 152.6, 130.0, 128.2, 127.2, 60.9, 52.1, 40.7, 36.5, 22.2. **IR** (ATR): 3475, 2954, 2926, 2874, 2853, 1717, 1609, 1435, 1311, 1274, 1179, 1109, 1041, 1017, 993, 855, 773, 707 cm^{-1}. **HRMS** (ESI) m/z calcd for $C_{12}H_{16}NaO_3^+$ 231.0992 [*M* + Na]$^+$; found: 231.0992.

3-(5-methylfuran-2-yl)butan-1-ol (1j')

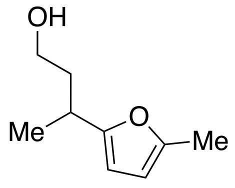

Following general procedure **II**, **1j'** was obtained as a yellow oil (34 mg, 68% yield). **R*f*** = 0.38 (CH_2Cl_2/MeOH, 95:5). **^{1}H NMR** (400 MHz, $CDCl_3$) δ = 5.88 – 5.81 (m, 2H), 3.71 – 3.60 (m, 2H), 2.94 (h, *J* = 7.0 Hz, 1H), 2.25 (d, *J* = 1.0 Hz, 3H), 1.89 (ddt, *J* = 14.2, 8.0, 6.2 Hz, 1H), 1.82 – 1.72 (m, 1H), 1.43 (br s, 1H), 1.25 (d, *J* = 7.0 Hz, 3H). **^{13}C NMR** (101 MHz, $CDCl_3$) δ 158.3, 150.4, 105.8, 104.5, 61.2, 38.9, 30.1, 19.5, 13.7. **IR** (ATR) 3320, 2964, 2926, 2875, 1565, 1452, 1378, 1219, 1043, 1018, 938, 776 cm^{-1}. **HRMS** (EI) m/z calcd. for $C_9H_{14}O_2^+$ 154.0988 [M]$^+$; found 154.0989.

3-(4-Fluorophenyl)hexan-1-ol (1l')

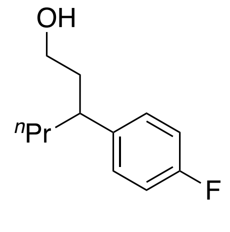

Following general procedure **II**, **1l'** was obtained as a yellow oil (32 mg, 62% yield). **R*f*** = 0.60 (*n*-hexane/EtOAc, 50:50). **^{1}H NMR** (400 MHz, $CDCl_3$) *δ* = 7.17 – 7.08 (m, 2H), 7.01 – 6.94 (m, 2H), 3.53 (ddd, *J* = 10.6, 7.0, 5.3 Hz, 1H), 3.44 (ddd, *J* = 10.6, 7.7, 6.4 Hz, 1H), 2.70 (tt, *J* = 8.1, 4.3 Hz, 1H), 1.92 (dddd, *J* = 13.7, 7.6, 7.0, 5.0 Hz, 1H), 1.75 (dddd, *J* = 13.7, 10.0, 6.4, 5.3 Hz, 1H), 1.67 – 1.47 (m, 2H), 1.23 – 1.07 (m, 2H), 0.84 (t, *J* = 7.3 Hz, 3H). **^{13}C NMR** (101 MHz, $CDCl_3$) *δ* = 161.5 (d, *J* = 243.5 Hz), 140.9 (d, *J* = 3.2 Hz), 129.0 (d, *J* = 7.6 Hz), 115.3 (d, *J* = 21.0 Hz), 61.2, 41.5, 39.8, 39.4, 20.7, 14.2. **^{19}F NMR** (377 MHz, $CDCl_3$) *δ* = –117.34 (m). **IR** (ATR): 3329, 2956, 2928, 2871, 1707, 1602, 1507, 1459, 1221, 1158, 1092, 1038, 1014, 829 cm^{-1}. **HRMS** (EI) m/z calcd for $C_{12}H_{17}FO^+$ 196.1258 [*M*]$^+$; found: 196.1258.

(*R*)-3-(4-Fluorophenyl)dodecan-1-ol (1m')

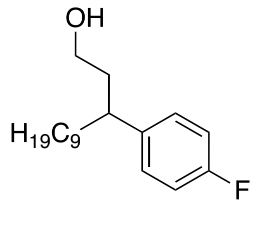

Following general procedure **II**, **1m'** was obtained as a yellow oil (39 mg, 78% yield). **R*f*** = 0.73 (*n*-hexane/EtOAc, 50:50). **^{1}H NMR** (400 MHz, $CDCl_3$) *δ* = 7.13 (ddt, *J* = 8.5, 5.2, 2.6 Hz, 2H), 7.04 – 6.96 (m, 2H), 3.55 (ddd, *J* = 10.5, 6.9, 5.2 Hz, 1H), 3.46 (ddd, *J* = 10.6, 7.7, 6.4 Hz, 1H), 2.70 (tt, *J* = 9.9, 5.2 Hz, 1H), 2.00 – 1.90 (m, 1H), 1.77 (dddd, *J* = 13.8, 10.0, 6.4, 5.3 Hz, 1H), 1.59 – 1.54 (m, 2H), 1.30 – 1.19 (m, 14H), 0.90 (m, 3H). **^{13}C NMR** (101 MHz, $CDCl_3$) *δ* = 161.5 (d, *J* = 243.6 Hz), 141.0 (d, *J* = 3.1 Hz), 129.0 (d, *J* = 7.7 Hz), 115.3 (d, *J* = 21.0 Hz), 61.2, 41.8, 39.9, 37.2, 32.0, 29.8, 29.7, 29.7, 29.4, 27.6, 22.8, 14.3. **^{19}F NMR** (376 MHz, $CDCl_3$) *δ* = –117.36 (tt, *J* = 8.7, 5.3 Hz). **IR** (ATR): 3332, 2922, 2852, 1603, 1508, 1461, 1223, 1157, 1045, 1015, 832 cm^{-1}. **HRMS** (EI) m/z calcd for $C_{18}H_{29}O^+$ 280.2197 [*M*]$^+$; found: 280.2202.

3-(4-Fluorophenyl)-4-methylpentan-1-ol (1n')

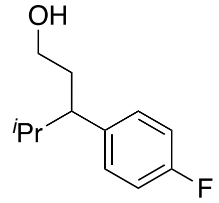

Following general procedure **II**, **1n'** was obtained as a yellow oil (28 mg, 56% yield). **R*f*** = 0.63 (*n*-hexane/EtOAc 50:50). **^{1}H NMR** (500 MHz, $CDCl_3$) *δ* = 7.14 – 7.08 (m, 2H), 7.03 – 6.96 (m, 2H), 3.50 (ddd, *J* = 10.5, 7.5, 4.8 Hz, 1H), 3.39 (ddd, *J* = 10.5, 7.9, 6.7 Hz, 1H), 2.45 (ddd, *J* = 11.5, 7.7, 3.9 Hz, 1H), 2.10 (dtd, *J* = 13.6, 7.7, 3.9 Hz, 1H), 1.85 – 1.76 (m, 1H), 0.98 (d, *J* = 6.6 Hz, 3H), 0.74 (d, *J* = 6.7 Hz, 3H). **^{13}C NMR** (126 MHz, $CDCl_3$) *δ* = 161.4 (d, *J* = 243.6 Hz), 139.3 (d, *J* = 3.2 Hz), 129.6 (d, *J* = 7.6 Hz), 115.0 (d, *J* = 20.9 Hz), 61.5, 48.7, 36.0, 33.6 (d, *J* = 1.0 Hz), 20.9, 20.5. **^{19}F NMR** (471 MHz, $CDCl_3$) *δ* = –117.36 (tt, *J* = 8.6, 5.4 Hz). **IR** (ATR): 3321, 2955, 2927, 2871, 1602, 1507, 1466, 1384, 1221, 1158, 1041, 1014, 825 cm^{-1}. **HRMS** (EI) m/z calcd. for $C_{12}H_{17}FO^+$ 196.1258 [*M*]$^+$; found: 196.1258.

5 Kinetic Resolution of Racemic β-Branched Aldehydes

General Procedure III – β-branched aldehyde substrate scope: A solution of the racemic β-branched aldehyde (**1**, 1 equiv., 0.75 mmol, 0.5 M) in $CHCl_3$/*i*-PrOH 9:1 (1.5 mL) was cooled to 0 °C. Nitrostyrene **2a** (1.5 equiv., 1.125 mmol), the TFA salt of peptide **B** (3 mol%, 22.5 μmol) and *N*-methylmorpholine (3 mol%, 22.5 μmol) were added and stirred at 0 °C. The reaction progress was followed by taking samples at different time points. The amount of unreacted enantioenriched β-branched aldehyde was determined by ^{1}H NMR spectroscopy by comparison of the signals of the starting aldehyde and the γ-nitroaldehyde product (50 μL of the reaction mixture were taken and dissolved in $CDCl_3$). The enantiomeric excess of the unreacted (enantiomerically enriched) aldehyde **1** was determined by chiral stationary phase SFC or HPLC after a preparative TLC purification.

(*R*)-3-Phenylbutanal ((*R*)-1a)

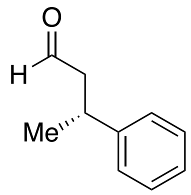

Following general procedure **III**, 47% of aldehyde **1a** remained after 21 h, 95% ee. The enantiomeric excess was determined by chiral stationary phase SFC with a Whelk column (CO_2/MeOH 0.1%, 40 °C) at 2 ml/min, 10 min, UV detection at λ = 214 nm t_R(*R*) at 6.8 min, t_R(*S*) at 7.3 min.

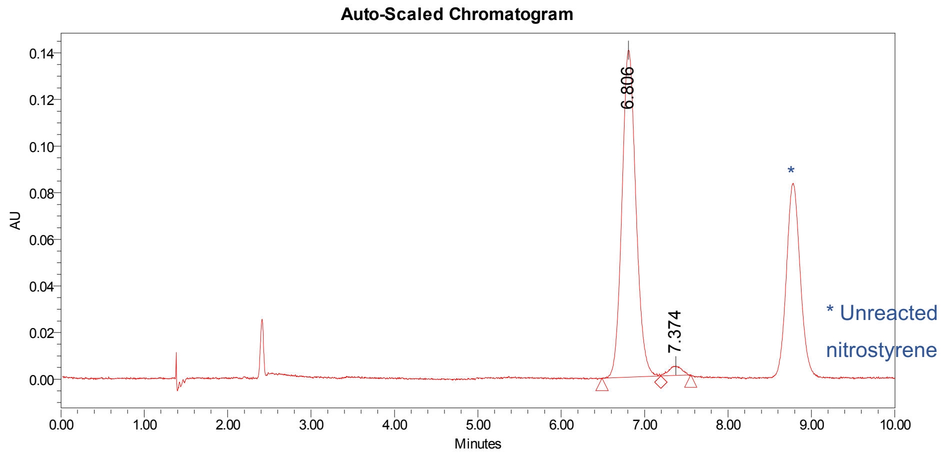

Processed Channel: PDA 214.0 nm (190-400)nm

	Processed Channel	Retention Time (min)	Area	% Area	Height
1	PDA 214.0 nm (190-400)nm	6.806	1630971	97.37	140279
2	PDA 214.0 nm (190-400)nm	7.374	44085	2.63	4182

Racemic:

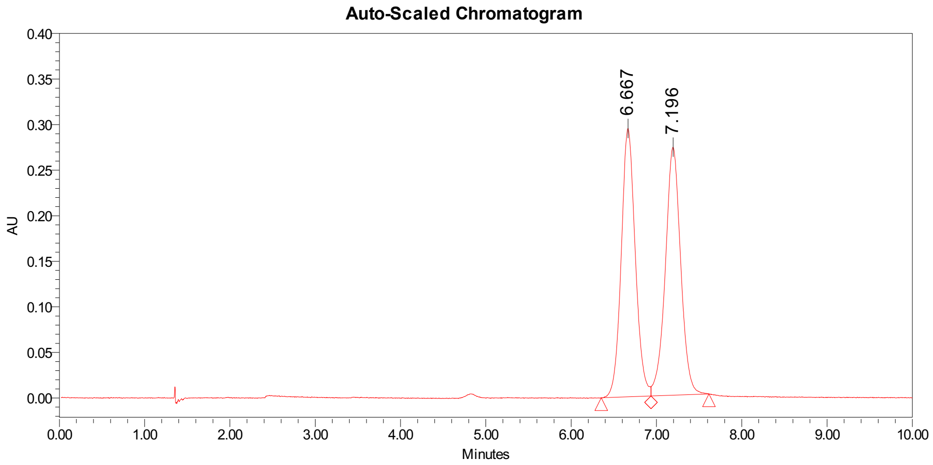

Processed Channel: PDA Ch2 214nm@1.2nm

	Processed Channel	Retention Time (min)	Area	% Area	Height
1	PDA Ch2 214nm@1.2nm	6.667	3372719	49.72	294475
2	PDA Ch2 214nm@1.2nm	7.196	3410774	50.28	272188

(*R*)-3-(*p*-Tolyl)butanal ((*R*)-1b)

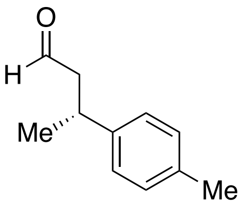

Following general procedure **III**, 45% of aldehyde **1b** remained after 20 h, 94% ee. The enantiomeric excess was determined by chiral stationary phase SFC with a Whelk column (CO_2/MeOH 0.5%, 40 °C) at 2 ml/min, 15 min, UV detection at λ = 214 nm t_R(*R*) at 6.5 min, t_R(*S*) at 7.1 min.

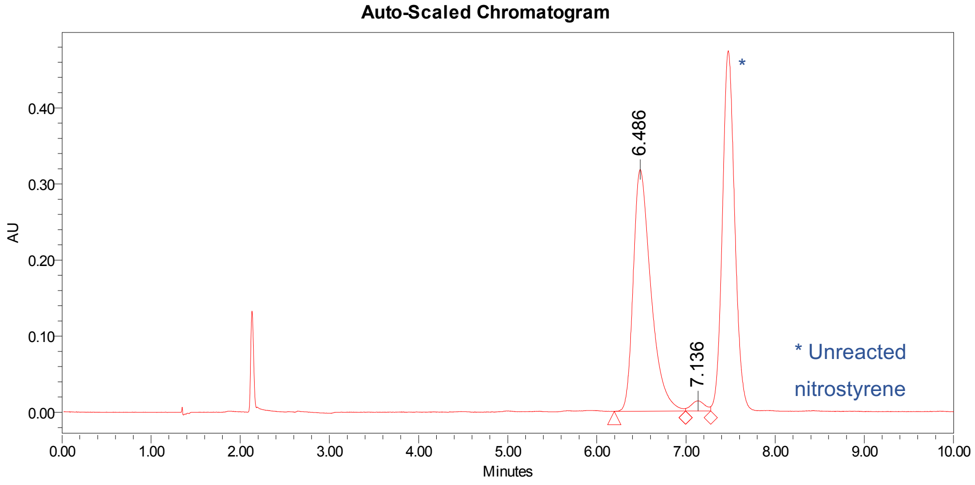

Processed Channel: PDA 214.0 nm (190-400)nm

	Processed Channel	Retention Time (min)	Area	% Area	Height
1	PDA 214.0 nm (190-400)nm	6.486	4391569	96.82	317765
2	PDA 214.0 nm (190-400)nm	7.136	144213	3.18	13357

Racemic:

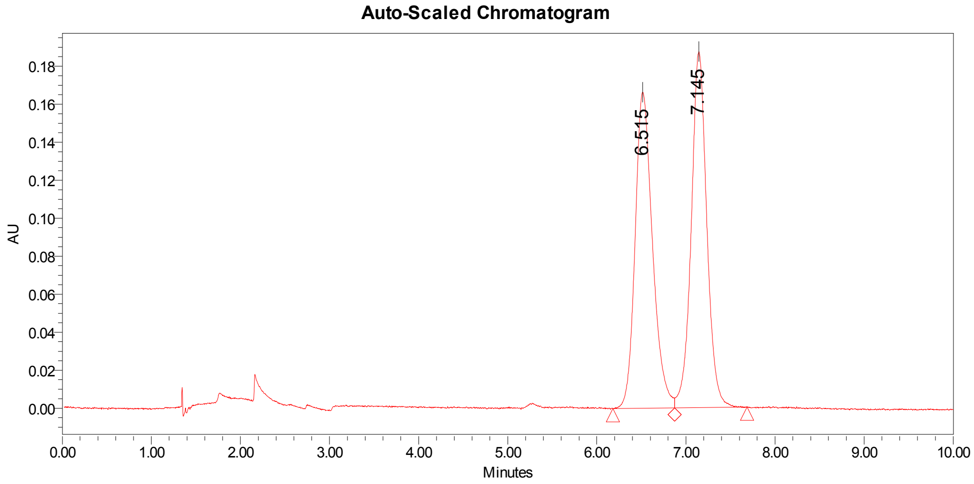

Processed Channel: PDA 214.0 nm (190-400)nm

	Processed Channel	Retention Time (min)	Area	% Area	Height
1	PDA 214.0 nm (190-400)nm	6.515	2260770	49.89	166339
2	PDA 214.0 nm (190-400)nm	7.145	2270716	50.11	187317

Table S7: Reaction progress over time with aldehyde **1b**

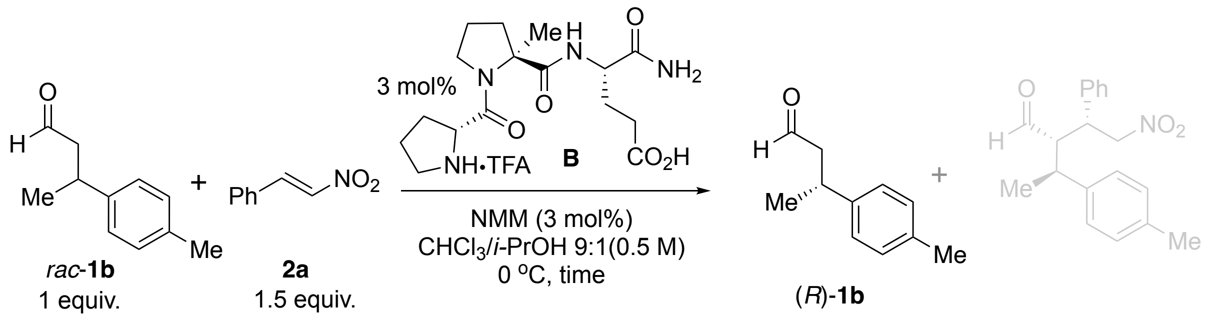

Entry	Time (h)	Remaining 1b (%)[a]	ee (%)[b]	Selectivity factor (*s*)[c]
1	1	88	12	18
2	2	81	20	15
3	3	75	29	19
4	4	71	37	29
5	6	65	50	28
6	20	45	94	27
7	22	44	95	25
8	24	43	96	24

[a] The amount of remaining enantioenriched aldehyde **1b** was determined by ^{1}H NMR spectroscopy analysis of the crude reaction mixture. [b] Enantioselectivity was determined by chiral stationary phase SFC analysis. [c] The selectivity factor was calculated according to *Kagan*[7] assuming a first order dependence on substrate **1b**; see further discussion below.

Discussion on the Suitability of Using Selectivity Factor:

The commonly used metrics for depicting the efficiency of a kinetic resolution – selectivity factor (*s*)[7] – assumes that the reaction is first order with respect to the substrate. However, our previous studies revealed a less than 1st order at low and zero-order at higher aldehyde concentration.[8] Here, monitoring the reaction over time showed increasing selectivity factors at higher conversion (Tables S7–S9), corroborating that the reaction obeys a more complex rate law. Therefore, we refrained from using the *s* values to describe this kinetic resolution. For further discussions on the suitability of using the selectivity factor, see references 9 and 10.

(*R*)-3-(4-(*tert*-Butyl)phenyl)butanal ((*R*)-1c)

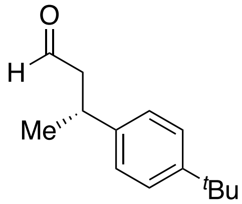

Following general procedure **III**, 49% of aldehyde **1c** remained after 20 h, 93% ee. The enantiomeric excess was determined by chiral stationary phase SFC with a Whelk column (CO_2/MeOH 0.5%, 40 °C) at 2 ml/min, 15 min, UV detection at λ = 214 nm t_R(*R*) at 6.6 min, t_R(*S*) at 7.0 min.

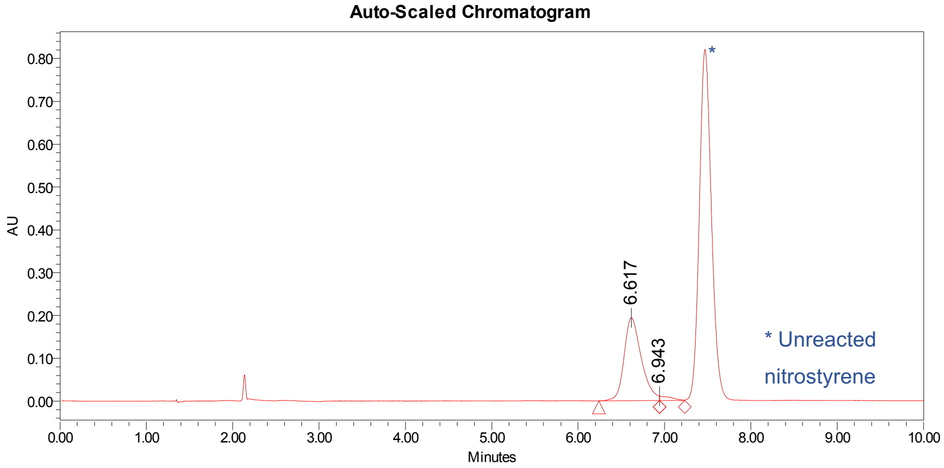

Processed Channel: PDA 214.0 nm (190-400)nm

	Processed Channel	Retention Time (min)	Area	% Area	Height
1	PDA 214.0 nm (190-400)nm	6.617	2687879	96.44	193921
2	PDA 214.0 nm (190-400)nm	6.943	99277	3.56	10460

Racemic:

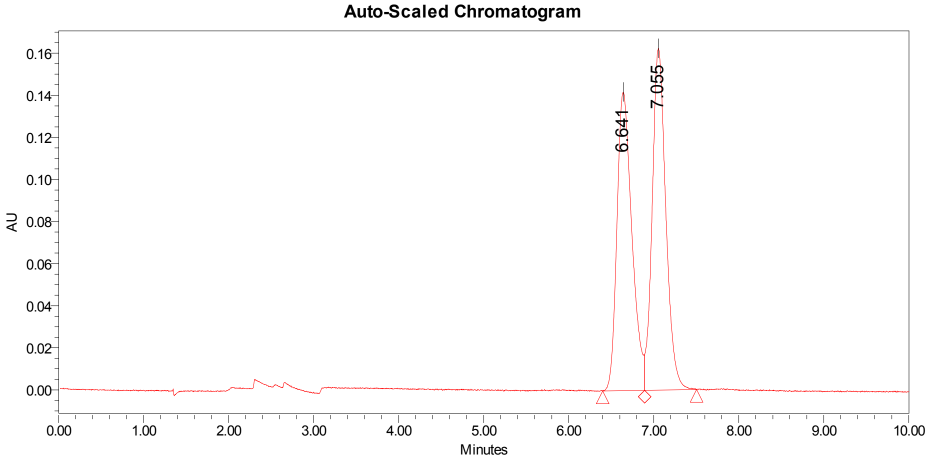

Processed Channel: PDA 214.0 nm (190-400)nm

	Processed Channel	Retention Time (min)	Area	% Area	Height
1	PDA 214.0 nm (190-400)nm	6.641	1737489	49.02	141966
2	PDA 214.0 nm (190-400)nm	7.055	1807237	50.98	162364

Table S8: Reaction progress over time with aldehyde **1c**

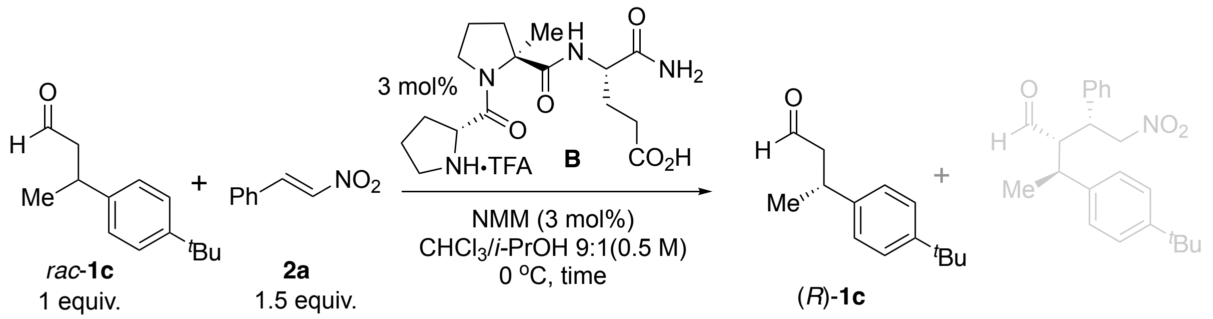

Entry	Time (h)	Remaining 1c (%)[a]	ee (%)[b]	Selectivity factor (*s*)[c]
1	1	88	9	5
2	2	81	19	11
3	3	77	26	19
4	4	72	33	17
5	6	65	47	23
6	20	49	93	60
7	22	46	96	39
8	24	46	96	39

[a] The amount of remaining enantioenriched aldehyde **1c** was determined by ^{1}H NMR spectroscopy analysis of the crude reaction mixture. [b] Enantioselectivity was determined by chiral stationary phase SFC analysis. [c] The selectivity factor was calculated according to Kagan[7] assuming a first order dependence on substrate **1c**. For a discussion on the reasons for the fluctuating selectivity factor, see page 26.

(*R*)-3-(4-Methoxyphenyl)butanal ((*R*)-1d)

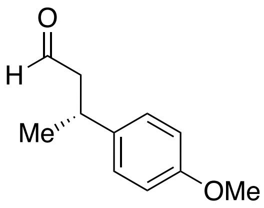

Following general procedure **III**, 46% of aldehyde **1d** remained after 24 h, 96% ee. The enantiomeric excess was determined by chiral stationary phase SFC with a Whelk column (CO_2/MeOH 0.5%, 40 °C) at 2 ml/min, 15 min, UV detection at λ = 214 nm t_R(*R*) at 12.3 min, t_R(*S*) at 13.1 min.

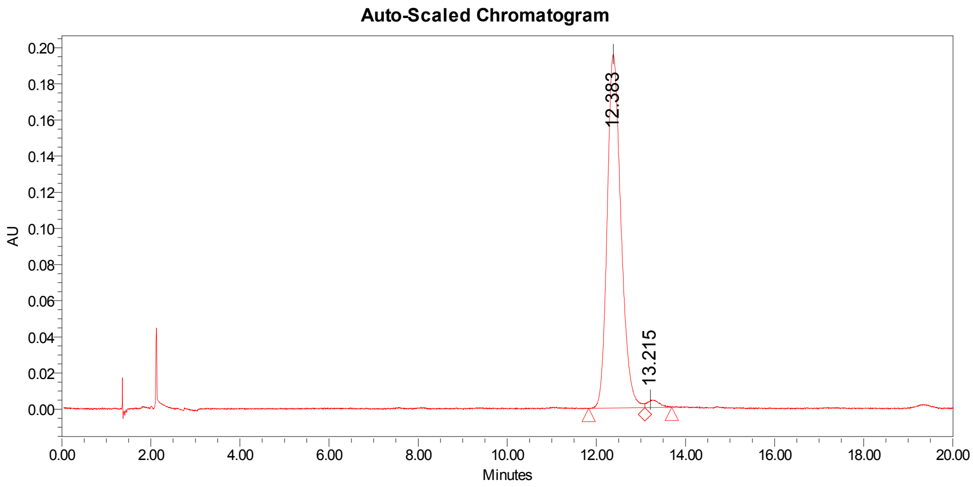

Processed Channel: PDA 214.0 nm (190-400)nm

	Processed Channel	Retention Time (min)	Area	% Area	Height
1	PDA 214.0 nm (190-400)nm	12.383	4194874	98.15	195790
2	PDA 214.0 nm (190-400)nm	13.215	79275	1.85	4362

Racemic:

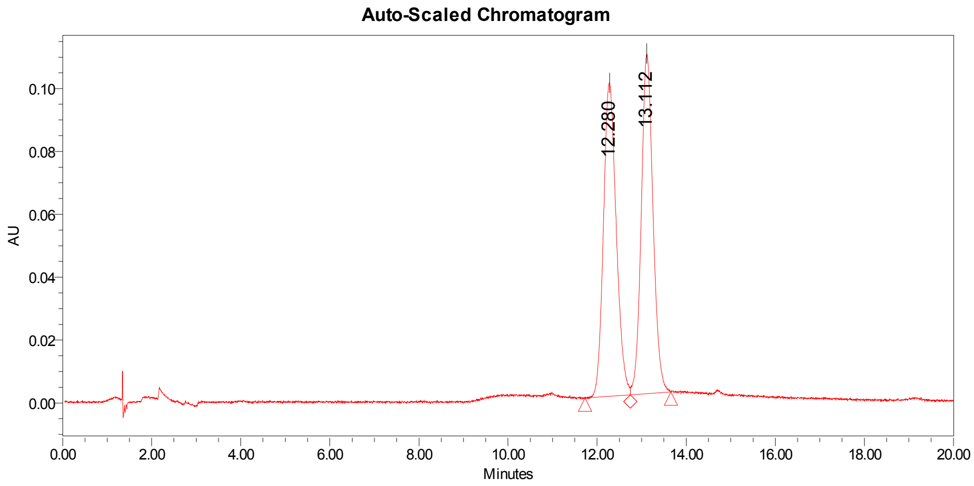

Processed Channel: PDA 214.0 nm (190-400)nm

	Processed Channel	Retention Time (min)	Area	% Area	Height
1	PDA 214.0 nm (190-400)nm	12.280	1985254	49.99	99675
2	PDA 214.0 nm (190-400)nm	13.112	1985979	50.01	108166

(*R*)-3-(4-(Trifluoromethyl)phenyl)butanal ((*R*)-1e)

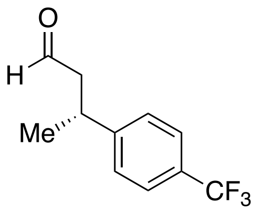

Following general procedure **III**, 49% of aldehyde **1e** remained after 42 h, 86% ee. The enantiomeric excess was determined by chiral stationary phase SFC with a Whelk column (CO_2/MeOH 0.5%, 40 °C) at 2 ml/min, 15 min, UV detection at λ = 214 nm t_R(*R*) at 4.5 min, t_R(*S*) at 5.1 min.

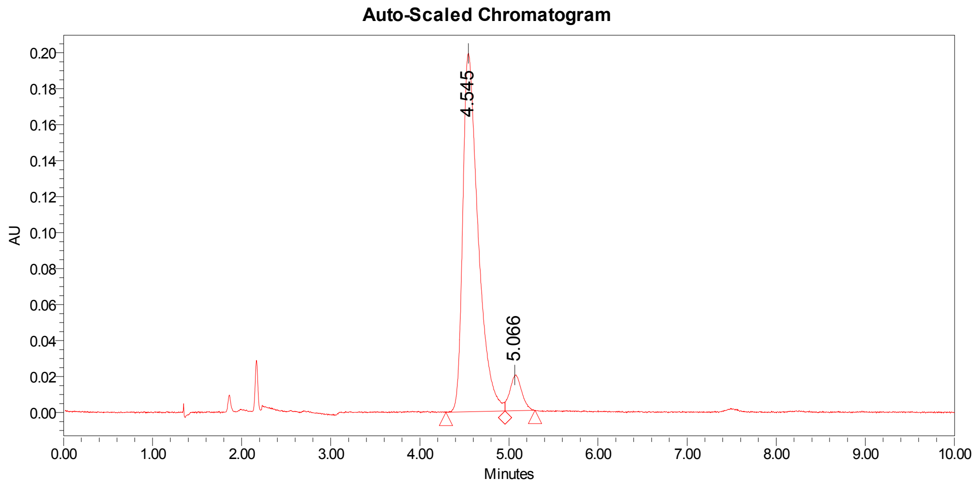

Processed Channel: PDA 214.0 nm (190-400)nm

	Processed Channel	Retention Time (min)	Area	% Area	Height
1	PDA 214.0 nm (190-400)nm	4.545	2382686	92.84	199089
2	PDA 214.0 nm (190-400)nm	5.066	183798	7.16	19982

Racemic:

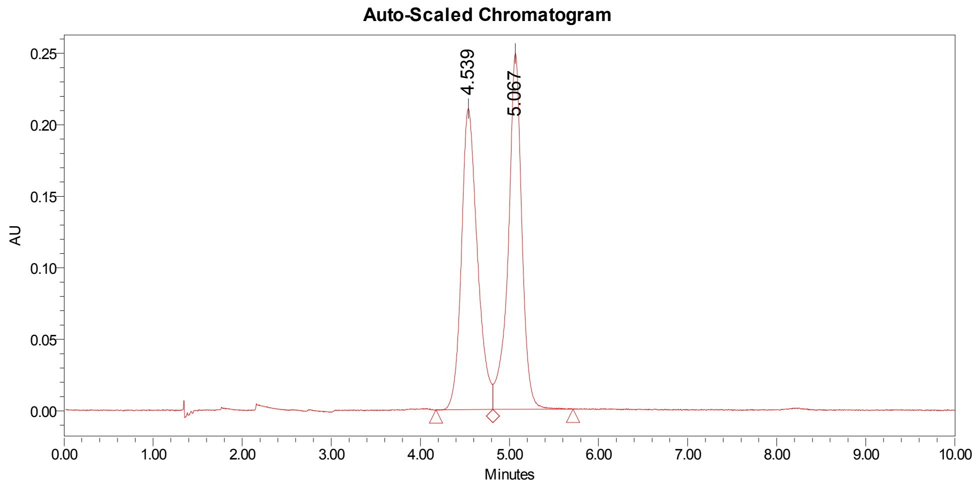

Processed Channel: PDA 214.0 nm (190-400)nm

	Processed Channel	Retention Time (min)	Area	% Area	Height
1	PDA 214.0 nm (190-400)nm	4.539	2626479	49.70	210475
2	PDA 214.0 nm (190-400)nm	5.067	2657728	50.30	248956

Methyl (*R*)-4-(4-oxobutan-2-yl)benzoate ((*R*)-1f)

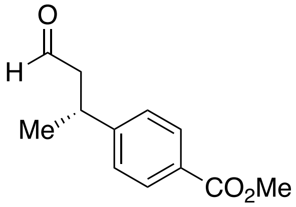

Following general procedure **III**, 51% of aldehyde **1f** remained after 25 h, 77% ee (alcohol **1f'**). The enantiomeric excess was determined after reduction to the alcohol **1f'** by chiral stationary phase SFC with a Trefoil column (CO_2/MeOH 5%, 40 °C) at 2 ml/min, 15 min, UV detection at λ = 214 nm t_R(*R*) at 3.6 min, t_R(*S*) at 3.2 min.

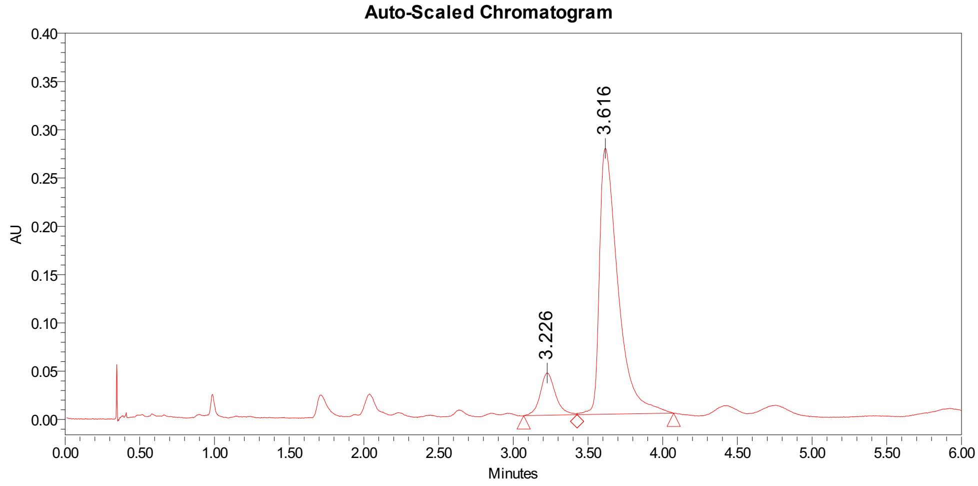

Processed Channel: PDA Ch2 214nm@1.2nm

	Processed Channel	Retention Time (min)	Area	% Area	Height
1	PDA Ch2 214nm@1.2nm	3.226	310155	11.53	43812
2	PDA Ch2 214nm@1.2nm	3.616	2380747	88.47	275429

Racemic:

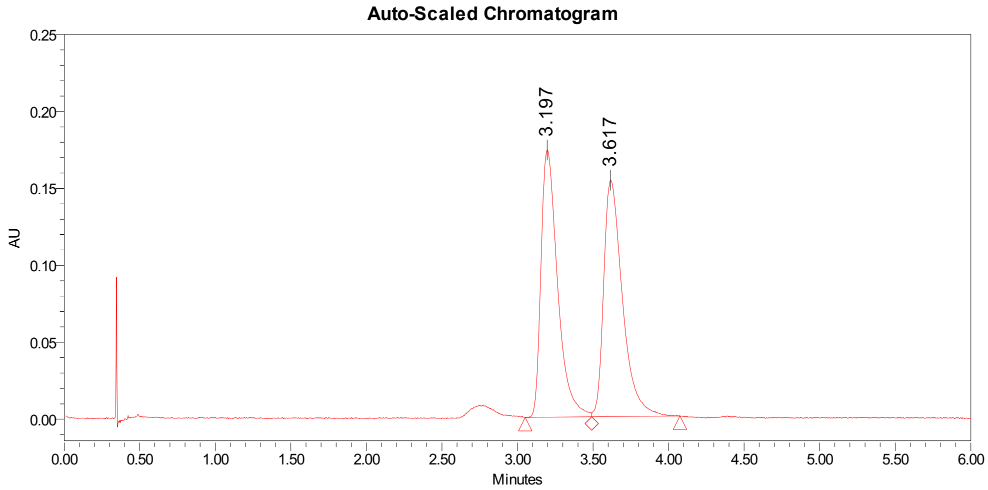

Processed Channel: PDA Ch2 214nm@1.2nm

	Processed Channel	Retention Time (min)	Area	% Area	Height
1	PDA Ch2 214nm@1.2nm	3.197	1327413	49.97	173623
2	PDA Ch2 214nm@1.2nm	3.617	1329260	50.03	153646

(*R*)-3-(4-Fluorophenyl)butanal ((*R*)-1g)

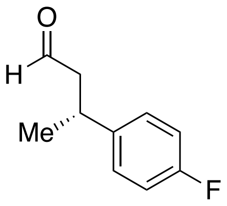

Following general procedure **III**, 49% of aldehyde **1g** remained after 30 h, 89% ee. The enantiomeric excess was determined by chiral stationary phase SFC with a Whelk column (CO_2/MeOH 0.5%, 40 °C) at 2 ml/min, 10 min, UV detection at λ = 214 nm t_R(*R*) at 5.5 min, t_R(*S*) at 5.9 min.

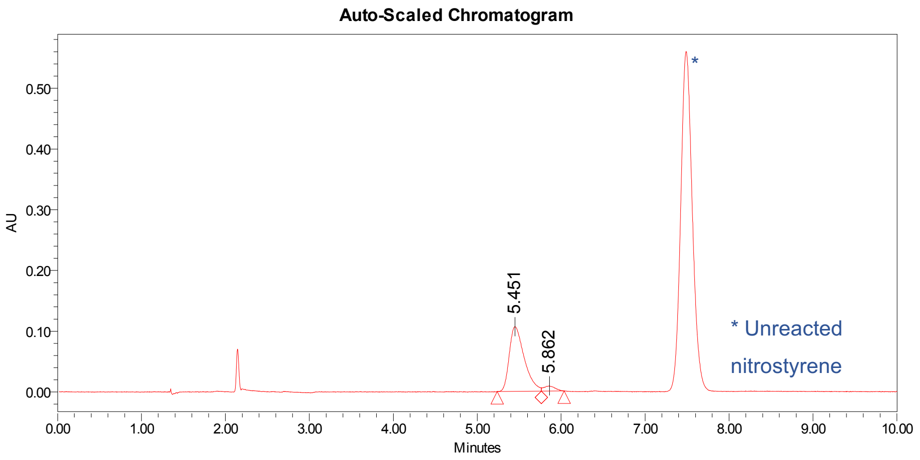

Processed Channel: PDA 214.0 nm (190-400)nm

	Processed Channel	Retention Time (min)	Area	% Area	Height
1	PDA 214.0 nm (190-400)nm	5.451	1320357	94.36	106694
2	PDA 214.0 nm (190-400)nm	5.862	78930	5.64	8465

Racemic:

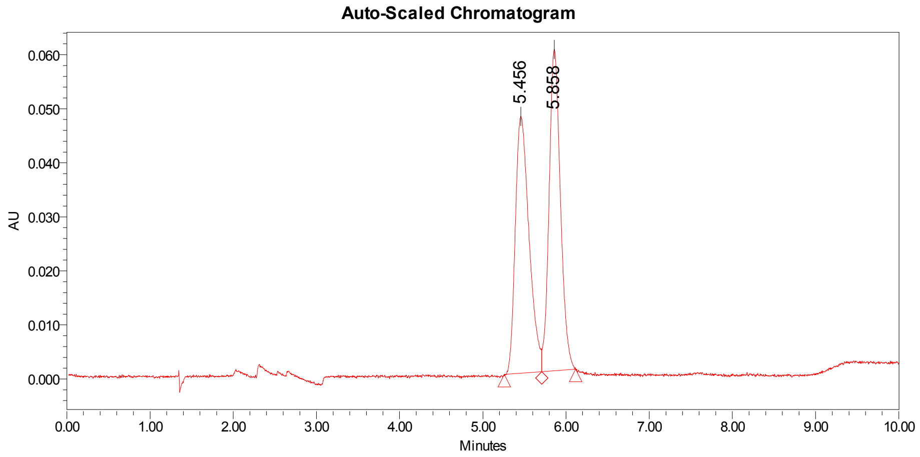

Processed Channel: PDA 214.0 nm (190-400)nm

	Processed Channel	Retention Time (min)	Area	% Area	Height
1	PDA 214.0 nm (190-400)nm	5.456	546449	49.20	47559
2	PDA 214.0 nm (190-400)nm	5.858	564280	50.80	59439

Table S9: Reaction progress over time with aldehyde **1g**

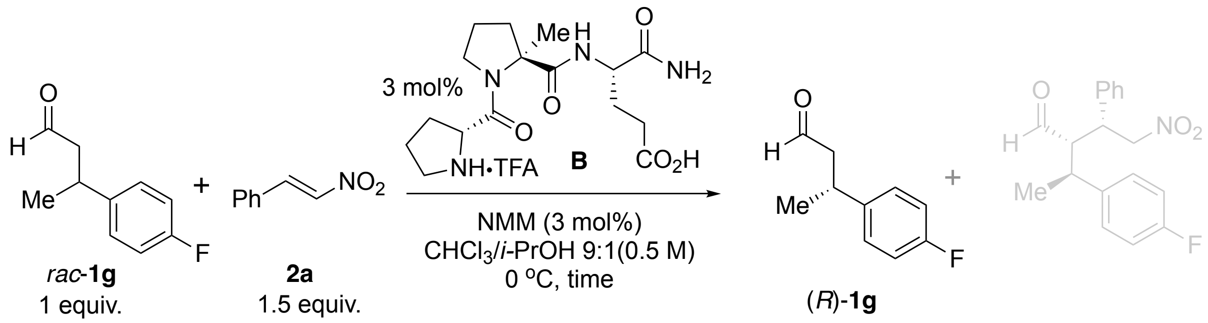

Entry	Time (h)	Remaining 1g (%)[a]	ee (%)[b]	Selectivity factor (*s*)[c]
1	1	93	4	3
2	2	89	8	5
3	3	86	13	10
4	4	82	19	17
5	6	77	26	19
6	20	55	73	38
7	22	54	77	46
8	24	53	80	48
9	26	52	85	66
10	28	51	86	50
11	30	49	89	38

[a] The amount of remaining enantioenriched aldehyde **1e** was determined by ^{1}H NMR spectroscopy analysis of the crude reaction mixture. [b] Enantioselectivity was determined by chiral stationary phase SFC analysis. [c] The selectivity factor was calculated according to Kagan[7] assuming a first order dependence on substrate **1e**. For a discussion on the reasons for the fluctuating selectivity factor, see page 26.

(*R*)-3-(3-Fluorophenyl)butanal ((*R*)-1h)

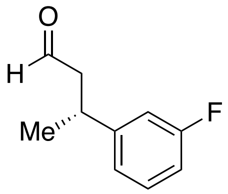

Following general procedure **III**, 48% of aldehyde **1h** remained after 56 h, 91% ee. The enantiomeric excess was determined by chiral stationary phase SFC with a Whelk column (CO_2/MeOH 0.5%, 40 °C) at 2 ml/min, 10 min, UV detection at λ = 214 nm t_R(*R*) at 5.0 min, t_R(*S*) at 5.5 min.

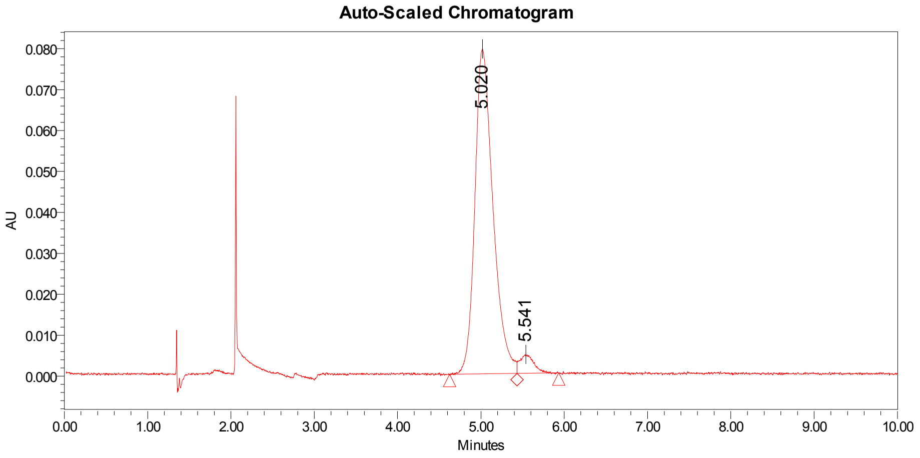

Processed Channel: PDA 214.0 nm (190-400)nm

	Processed Channel	Retention Time (min)	Area	% Area	Height
1	PDA 214.0 nm (190-400)nm	5.020	1201208	95.70	79373
2	PDA 214.0 nm (190-400)nm	5.541	53955	4.30	4615

Racemic:

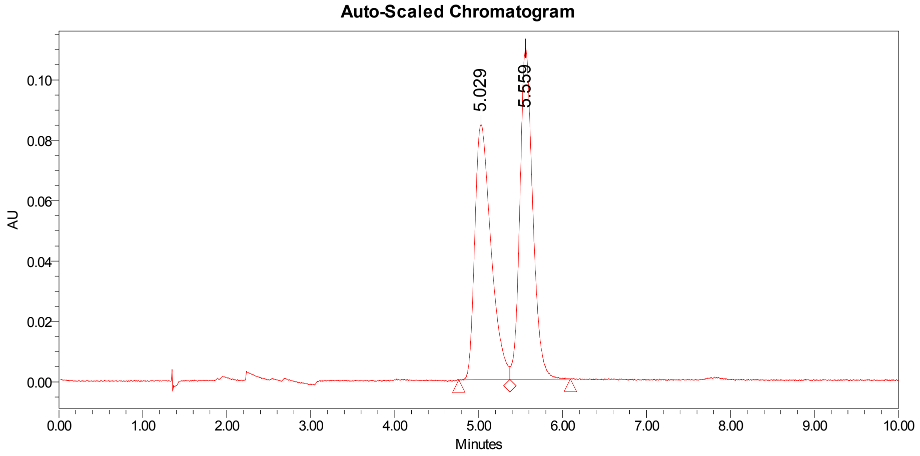

Processed Channel: PDA 214.0 nm (190-400)nm

	Processed Channel	Retention Time (min)	Area	% Area	Height
1	PDA 214.0 nm (190-400)nm	5.029	1132855	49.23	84476
2	PDA 214.0 nm (190-400)nm	5.559	1168117	50.77	109615

(*R*)-3-(2-Fluorophenyl)butanal ((*R*)-1i)

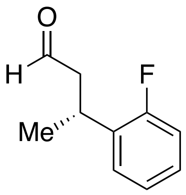

Following general procedure **III**, 46% of aldehyde **1i** remained after 47 h, 92% ee. The enantiomeric excess was determined by chiral stationary phase SFC with a Whelk column (CO_2/MeOH 0.5%, 40 °C) at 2 ml/min, 10 min, UV detection at λ = 214 nm t_R(*R*) at 5.1 min, t_R(*S*) at 5.6 min.

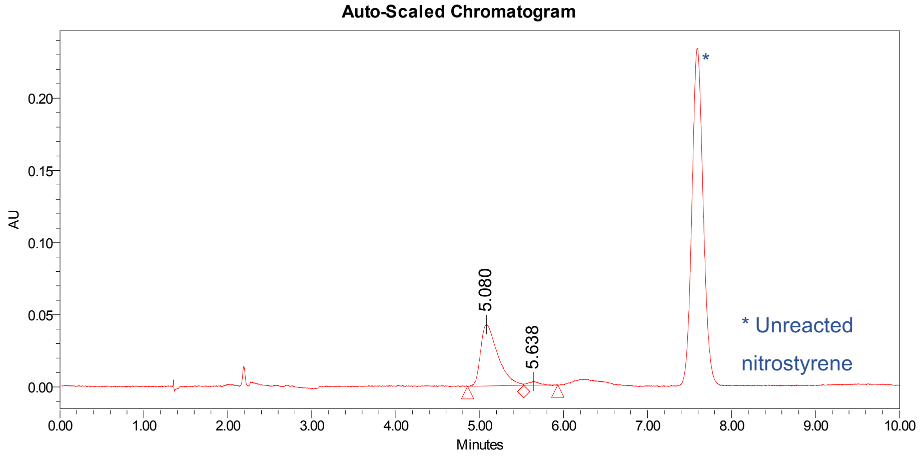

Processed Channel: PDA 214.0 nm (190-400)nm

	Processed Channel	Retention Time (min)	Area	% Area	Height
1	PDA 214.0 nm (190-400)nm	5.080	604220	96.09	42558
2	PDA 214.0 nm (190-400)nm	5.638	24611	3.91	2540

Racemic:

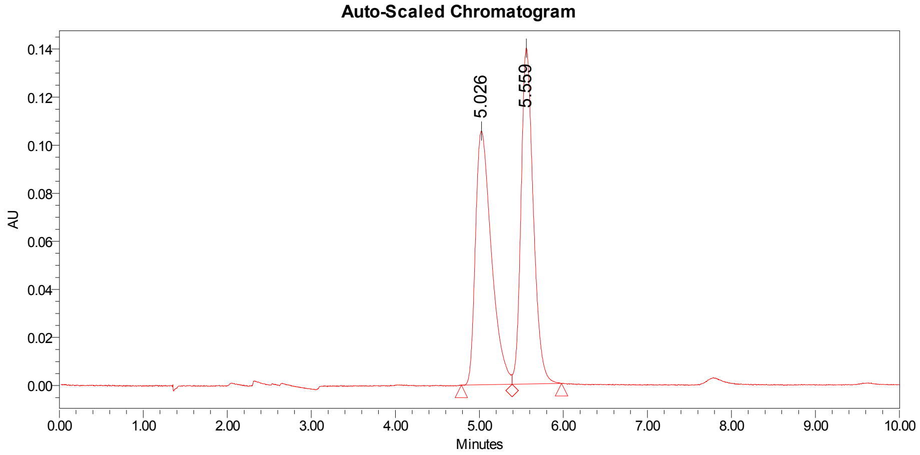

Processed Channel: PDA 214.0 nm (190-400)nm

	Processed Channel	Retention Time (min)	Area	% Area	Height
1	PDA 214.0 nm (190-400)nm	5.026	1393275	49.62	105593
2	PDA 214.0 nm (190-400)nm	5.559	1414333	50.38	139772

(*R*)-3-(5-Methylfuran-2-yl)butanal ((*R*)-1j)

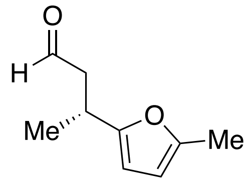

Following general procedure **III**, 49% of aldehyde **1f** remained after 21 h, 50% ee (alcohol **1j'**). The enantiomeric excess was determined after reduction to the alcohol **1j'** by chiral stationary phase HPLC with an OD-H column (*n*-hexane/*i*-PrOH 98:2, 25 °C) at 0.5 ml/min, UV detection at λ = 214 nm $t_R(R)$ at 37.5 min, $t_R(S)$ at 40.1 min.

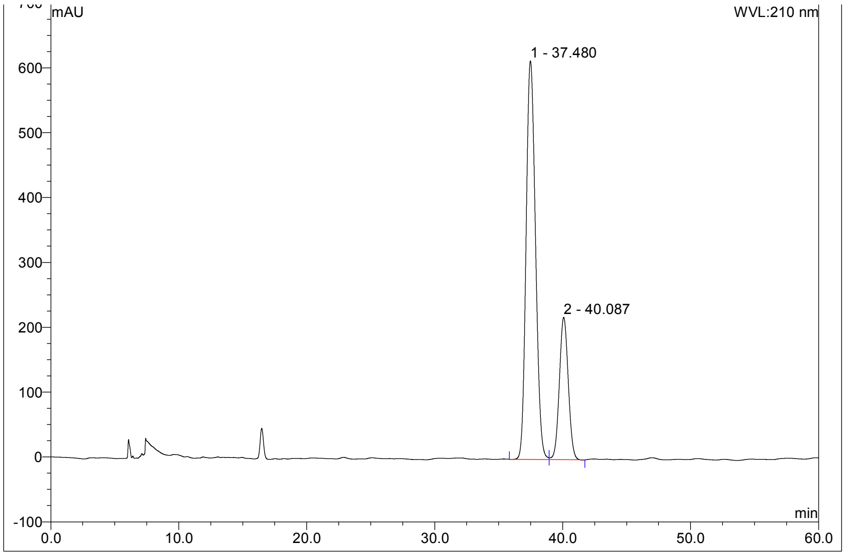

No.	Ret.Time min	Peak Name	Height mAU	Area mAU*min	Rel.Area %	Amount	Type
1	37.48	n.a.	614.821	521.502	74.67	n.a.	BM *
2	40.09	n.a.	219.568	176.920	25.33	n.a.	MB*

Racemic:

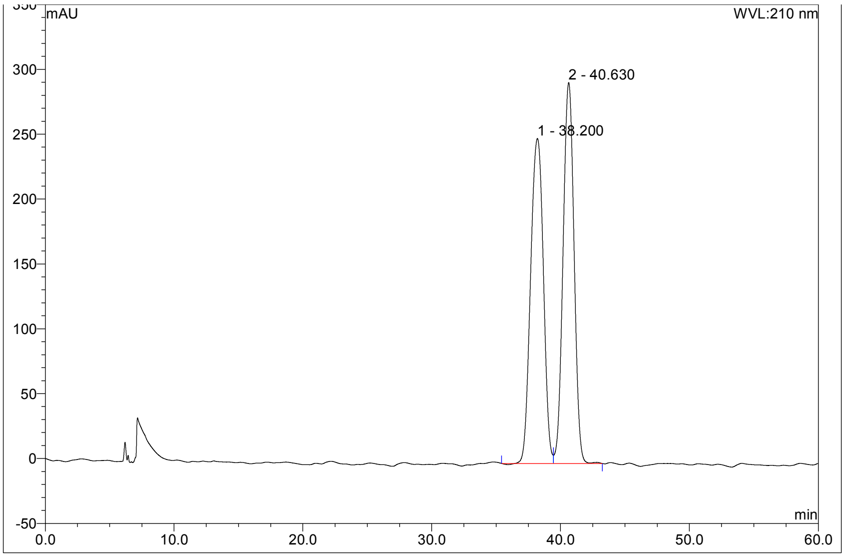

No.	Ret.Time min	Peak Name	Height mAU	Area mAU*min	Rel.Area %	Amount	Type
1	38.20	n.a.	250.538	288.579	49.85	n.a.	BM *
2	40.63	n.a.	293.704	290.374	50.15	n.a.	MB*

(*R*)-3-(4-Fluorophenyl)pentanal ((*R*)-1k)

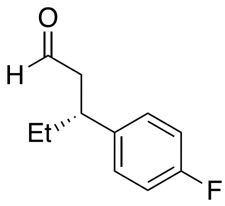

Following general procedure **III**, 50% of aldehyde **1k** remained after 48 h, 97% ee. The enantiomeric excess was determined by chiral stationary phase SFC with a Whelk column (CO_2/MeOH 0.1%, 40 °C) at 2 ml/min, 15 min, UV detection at λ = 214 nm t_R(*R*) at 7.3 min, t_R(*S*) at 7.8 min.

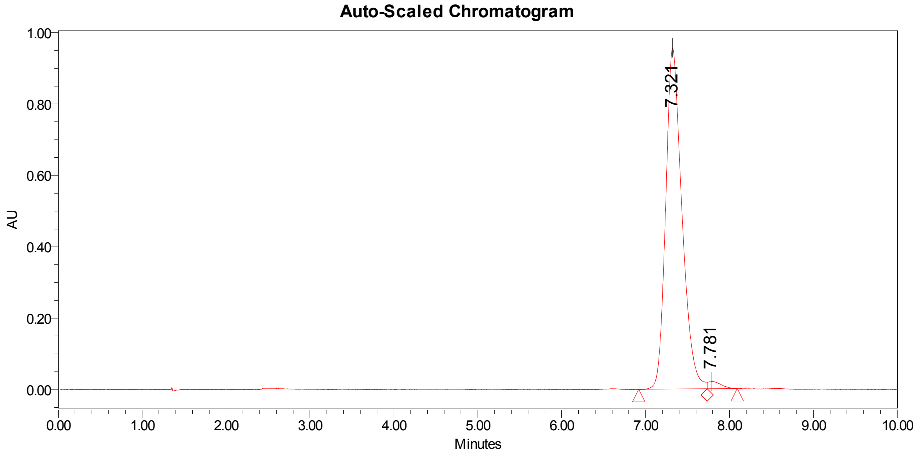

Processed Channel: PDA 214.0 nm (190-400)nm

	Processed Channel	Retention Time (min)	Area	% Area	Height
1	PDA 214.0 nm (190-400)nm	7.321	12868345	98.31	955244
2	PDA 214.0 nm (190-400)nm	7.781	221361	1.69	19954

Racemic:

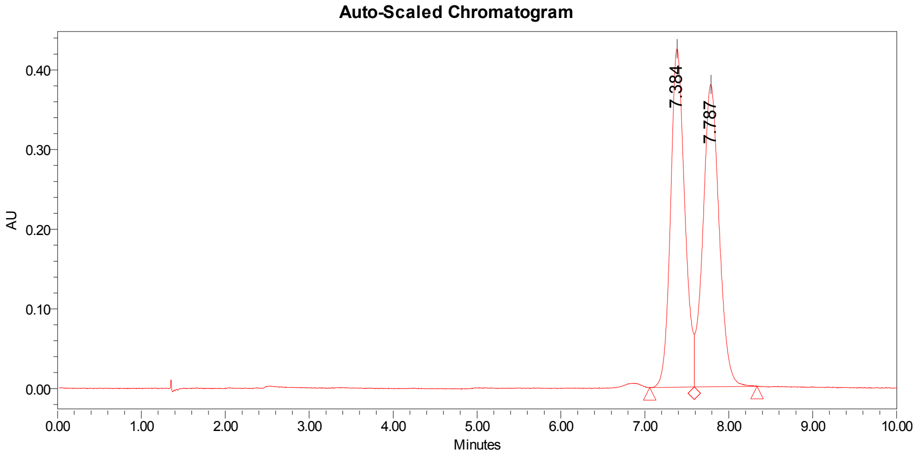

Processed Channel: PDA 214.0 nm (190-400)nm

	Processed Channel	Retention Time (min)	Area	% Area	Height
1	PDA 214.0 nm (190-400)nm	7.384	4990858	49.46	424979
2	PDA 214.0 nm (190-400)nm	7.787	5098924	50.54	379609

(*R*)-3-(4-Fluorophenyl)hexanal ((*R*)-1l)

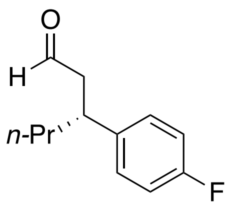

Following general procedure **III**, 52% of aldehyde **1l** remained after 54 h, 73% ee (alcohol **1l'**). The enantiomeric excess was determined after reduction to the alcohol **1l'** by chiral stationary phase SFC with a Whelk column (CO_2/MeOH 0.1%, 40 °C) at 2 ml/min, 10 min, UV detection at λ = 214 nm $t_R(R)$ at 12.5 min, $t_R(S)$ at 13.1 min.

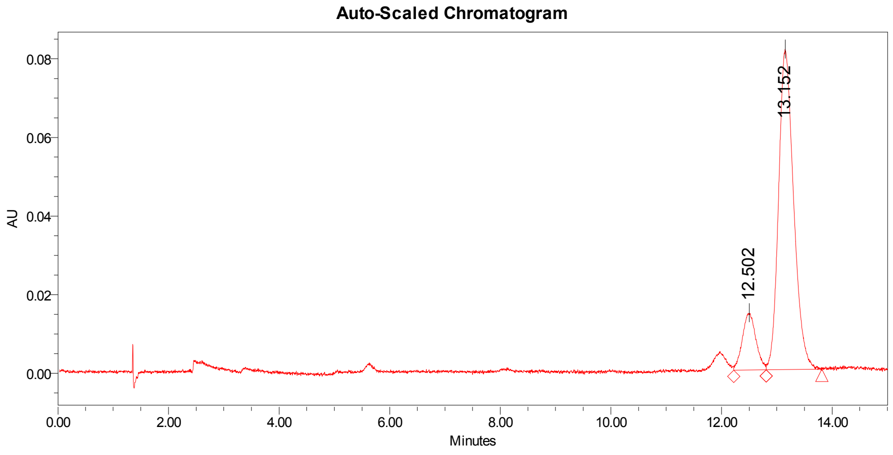

Processed Channel: PDA 214.0 nm (190-400)nm

	Processed Channel	Retention Time (min)	Area	% Area	Height
1	PDA 214.0 nm (190-400)nm	12.502	244333	13.70	14545
2	PDA 214.0 nm (190-400)nm	13.152	1539175	86.30	81501

Racemic:

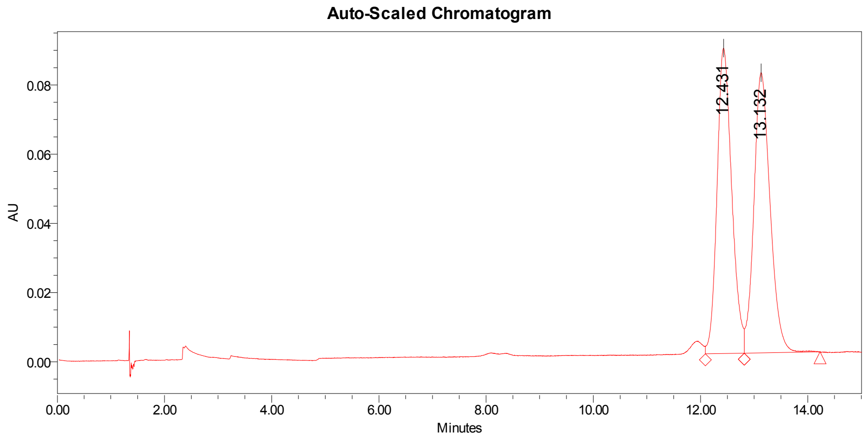

Processed Channel: PDA 214.0 nm (190-400)nm

	Processed Channel	Retention Time (min)	Area	% Area	Height
1	PDA 214.0 nm (190-400)nm	12.431	1676122	49.65	88265
2	PDA 214.0 nm (190-400)nm	13.132	1699731	50.35	80995

(*R*)-3-(4-Fluorophenyl)dodecanal ((*R*)-1m)

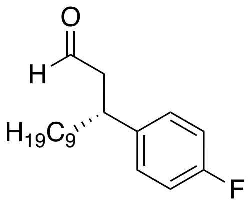

Following general procedure **III**, 48% of aldehyde **1m** remained after 46 h, 60% ee (alcohol **1m'**). The enantiomeric excess was determined after reduction to the alcohol **1m'** by chiral stationary phase SFC with a Whelk column (CO_2/MeOH 2%, 40 °C) at 2 ml/min, 15 min, UV detection at λ = 214 nm t_R(*R*) at 10.7 min, t_R(*S*) at 11.3 min.

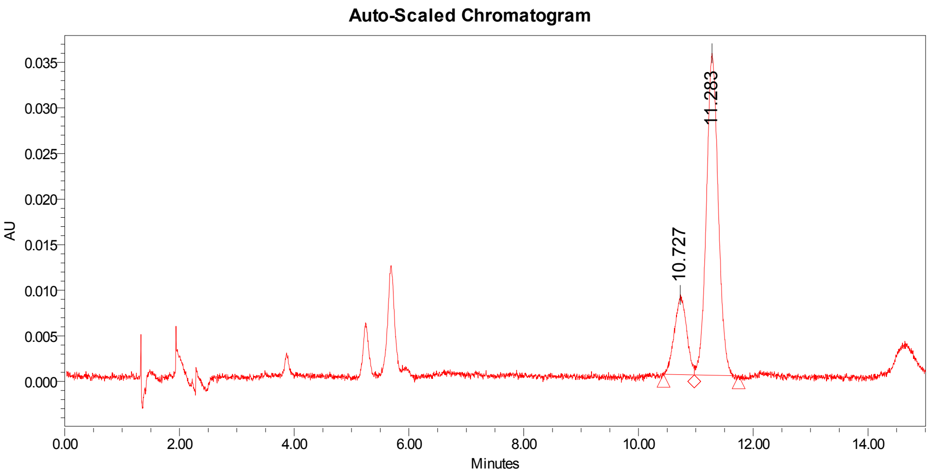

Processed Channel: PDA 214.0 nm (190-400)nm

	Processed Channel	Retention Time (min)	Area	% Area	Height
1	PDA 214.0 nm (190-400)nm	10.727	127063	19.95	8792
2	PDA 214.0 nm (190-400)nm	11.283	509689	80.05	35302

Racemic:

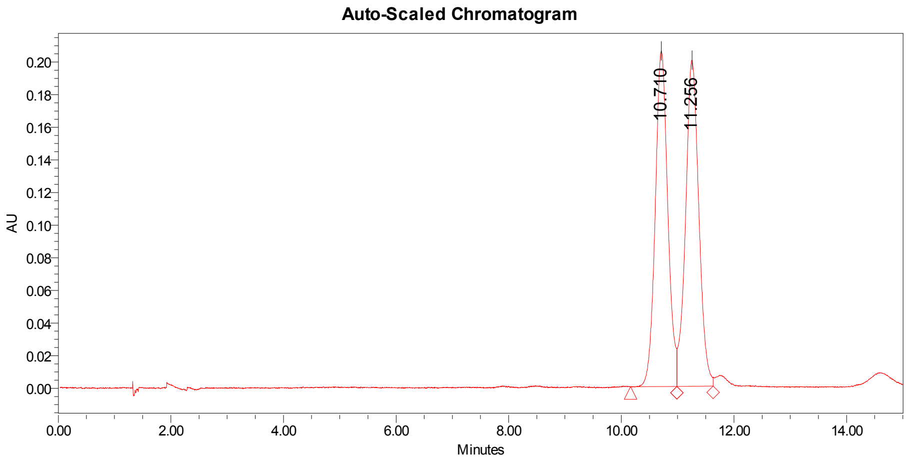

Processed Channel: PDA 214.0 nm (190-400)nm

	Processed Channel	Retention Time (min)	Area	% Area	Height
1	PDA 214.0 nm (190-400)nm	10.710	3172958	49.17	205816
2	PDA 214.0 nm (190-400)nm	11.256	3280262	50.83	199947

(*R*)-3-(4-Fluorophenyl)-4-methylpentanal ((*R*)-1n)

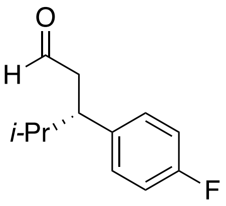

Following general procedure **III** (with 20 mol% of TFA·**B**/NMM), 29% of aldehyde **1n** remained after 68 h, 78% ee (alcohol **1n'**). The enantiomeric excess was determined after reduction to the alcohol **1n'** by chiral stationary phase SFC with a Whelk column (CO_2/MeOH 1%, 40 °C) at 2 ml/min, 10 min, UV detection at λ = 214 nm t_R(*R*) at 21.4 min, t_R(*S*) at 20.3 min.

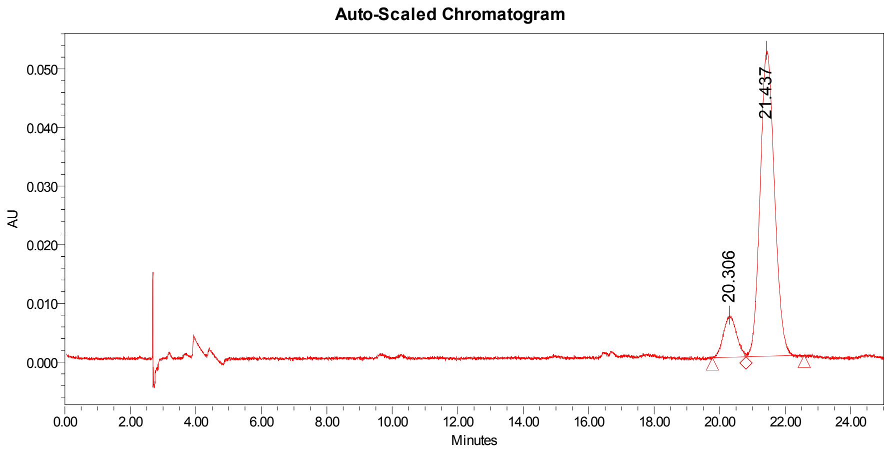

Processed Channel: PDA 214.0 nm (190-400)nm

	Processed Channel	Retention Time (min)	Area	% Area	Height
1	PDA 214.0 nm (190-400)nm	20.306	197257	11.01	7199
2	PDA 214.0 nm (190-400)nm	21.437	1594513	88.99	52191

Racemic:

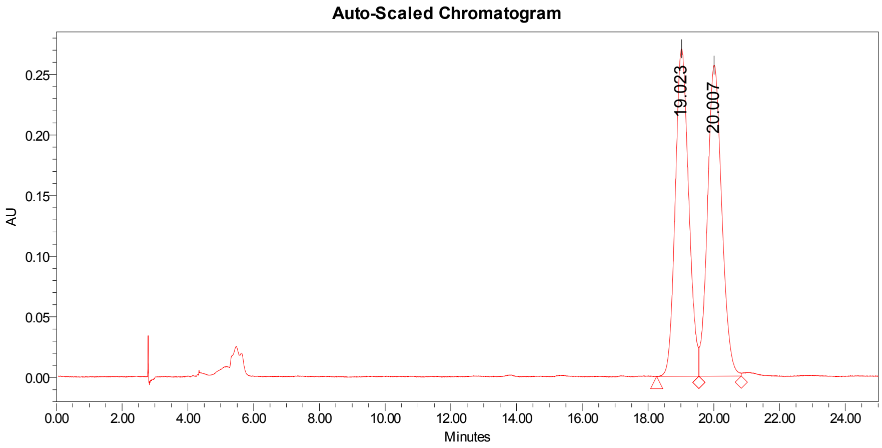

Processed Channel: PDA 214.0 nm (190-400)nm

	Processed Channel	Retention Time (min)	Area	% Area	Height
1	PDA 214.0 nm (190-400)nm	19.023	7683361	49.76	270202
2	PDA 214.0 nm (190-400)nm	20.007	7758817	50.24	256676

6 Synthesis and Analytical Data of γ-Nitroaldehydes

6.1 General Procedures

General procedure IV – γ-nitroaldehyde substrate scope: The peptide TFA salt **B** (3 mol%, 0.015 mmol) was placed in a vial and dissolved in $CHCl_3$/*i*-PrOH 9:1 (1 mL). Nitroolefin (1 equiv., 0.5 mmol, 0.5 M) and *N*-methylmorpholine (3 mol%, 0.015 mmol) were added and the solution was stirred at 0 °C for 5 min. Racemic β-branched aldehyde **1** (2.5 equiv., 1.25 mmol) was added and the solution was stirred for 24 h (unless otherwise stated). The solvent was removed under reduced pressure, and the crude mixture was subjected to flash column chromatography (*n*-hexane or cyclohexane/EtOAc 100:0 to 90:10) to yield the product after removal of all volatiles. Diastereoisomers of the γ-nitroaldehyde product were only separated by column chromatography in selected examples, the yield refers to the mixture of all diastereoisomers (unless otherwise stated). The diastereomeric ratio of the isolated product was determined by ^{1}H NMR spectroscopy by comparing the aldehyde R-CHO signals. Only signals of the major diastereoisomer are reported. The enantiomeric excess was determined by chiral stationary phase SFC or HPLC.

General procedure V – racemic γ-nitroaldehydes as reference compounds: The racemic γ-nitroaldehydes were synthesized using 20-30 mol% of pyrrolidine or 5 mol% of a 1:1 mixture of **A-DLL** and **A-LDD** using general procedure **IV**. ^{1}H NMR spectra of the crude mixture were recorded and used as a reference for the chemical shifts of the C*H*O signals of the four diastereomers of product **3**. A sample was purified by column chromatography or preparative TLC and used as a reference for chiral stationary phase SFC or HPLC. Reactions using racemic peptide catalyst **A** afforded the product in high diastereomeric ratios (typically >90:<5:<5:<1), and therefore not all stereoisomers were detected by HPLC in some cases.

Of note, reactions with racemic peptide catalyst **A** proceeded cleanly to the γ-nitroaldehydes. Pyrrolidine also catalyzed the formation of aldol side-products, which complicated the purification of the racemic conjugate addition products and in some cases did not allow to obtain a reference with all 8 possible stereoisomers separated by HPLC/SFC. In these cases, the HPLC/SFC conditions were optimized to separate the two enantiomers of the major diastereomer.

6.2 Comparison of Racemic Samples

a) Racemic **3aa** obtained using 5 mol% rac. peptide **A** (d.r. 90:6:1:3)

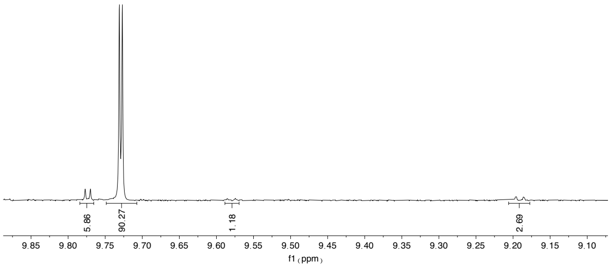

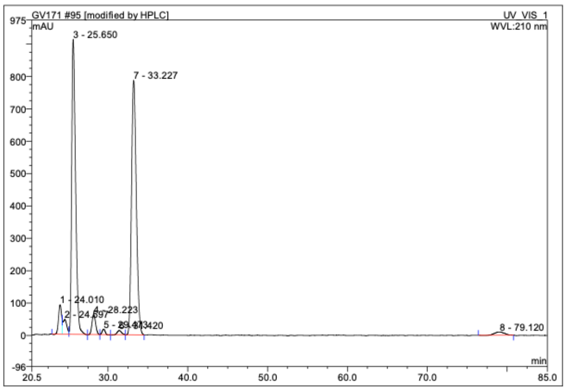

No.	Ret.Time min	Peak Name	Height mAU	Area mAU*min	Rel.Area %	Amount	Type
1	24.01	n.a.	91.100	39.922	3.37	n.a.	BM *
2	24.60	n.a.	45.570	26.697	2.25	n.a.	M *
3	25.65	n.a.	910.892	508.778	42.90	n.a.	M *
4	28.22	n.a.	61.463	32.059	2.70	n.a.	M *
5	29.47	n.a.	16.651	6.516	0.55	n.a.	M *
6	31.42	n.a.	12.774	7.065	0.60	n.a.	M *
7	33.23	n.a.	785.971	551.248	46.48	n.a.	MB*
8	79.12	n.a.	9.724	13.700	1.16	n.a.	BMB*
Total:			1934.145	1185.985	100.00	0.000	

b) Racemic **3aa** obtained using 20 mol% pyrrolidine (d.r. 61:14:7:18)

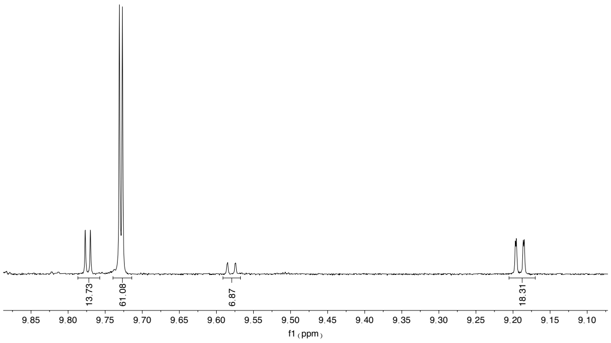

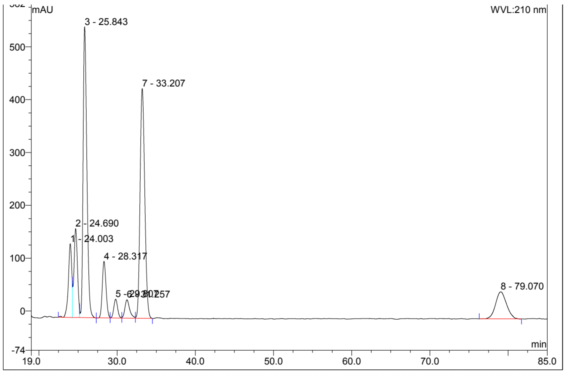

No.	Ret.Time min	Peak Name	Height mAU	Area mAU*min	Rel.Area %	Amount	Type
1	24.00	n.a.	139.468	68.678	7.20	n.a.	BM *
2	24.69	n.a.	168.306	89.835	9.42	n.a.	M *
3	25.84	n.a.	550.234	305.282	32.01	n.a.	M *
4	28.32	n.a.	106.977	59.562	6.25	n.a.	M *
5	29.81	n.a.	35.564	19.943	2.09	n.a.	M *
6	31.26	n.a.	34.723	24.422	2.56	n.a.	M *
7	33.21	n.a.	435.052	302.751	31.75	n.a.	MB*
8	79.07	n.a.	51.179	83.149	8.72	n.a.	BMB*
Total:			1521.503	953.623	100.00	0.000	

6.3 Assignment of Diastereoisomers

The aldehyde signals of all 4 diastereoisomers of compound **3aa** were assigned by analyzing the ^{1}H NMR spectra of the reaction mixtures of reactions with (*S*)-**1a** or (*R*)-**1a** (Figure S1).

Starting aldehyde **1a**	Possible diastereoisomers of product **3aa**	
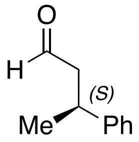 (*S*)-**1a**:	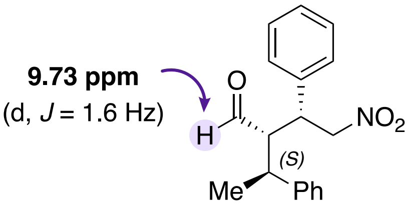 major diastereisomer (**3aa-A**)	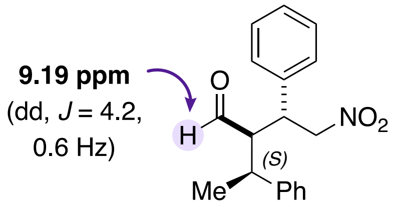 minor diastereoisomer (**3aa-B**)
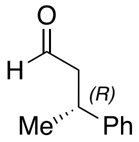 (*R*)-**1a**:	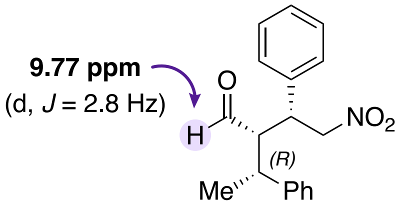 major diastereoisomer (**3aa-C**)	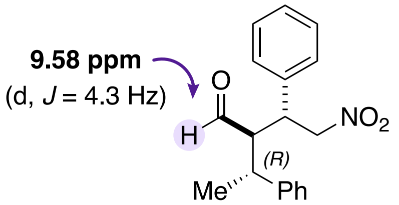 minor diastereoisomer (**3aa-D**)

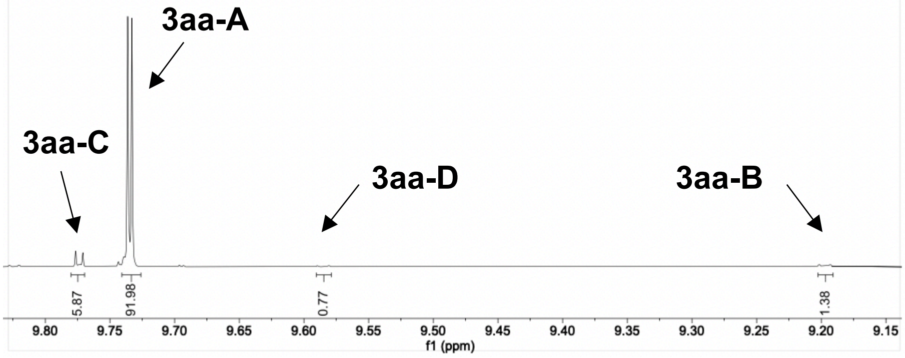

Figure S1: Assignment of aldehyde ^{1}H NMR signals of diastereomers of compound **3aa**.

The diastereomeric ratio for all γ-nitroaldehydes **3** is reported in the order of **A**:**C**:**D**:**B**.

6.4 Epimerization Test

No decomposition or epimerization of compound **3aa** was observed after >1 year when stored at 6 °C. No loss of d.r. was observed after stirring **3aa** in the presence of 1 equiv. NMM in $CDCl_3$ for 12 h.

In the presence of 1 equiv. of pyrrolidine, epimerization at the α-carbon to the aldehyde was observed, leading to the enrichment of the diastereomer **3aa-B** (Scheme S1).

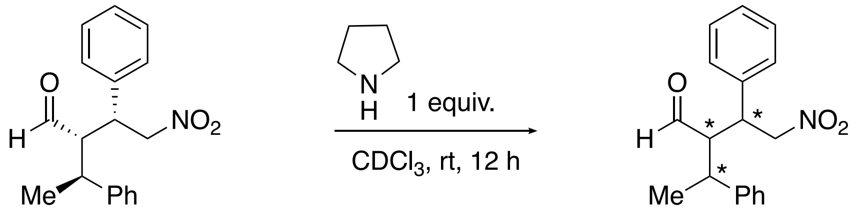

95:3:1:1 **A**:**C**:**D**:**B** → 65:2:2:30 **A**:**C**:**D**:**B**

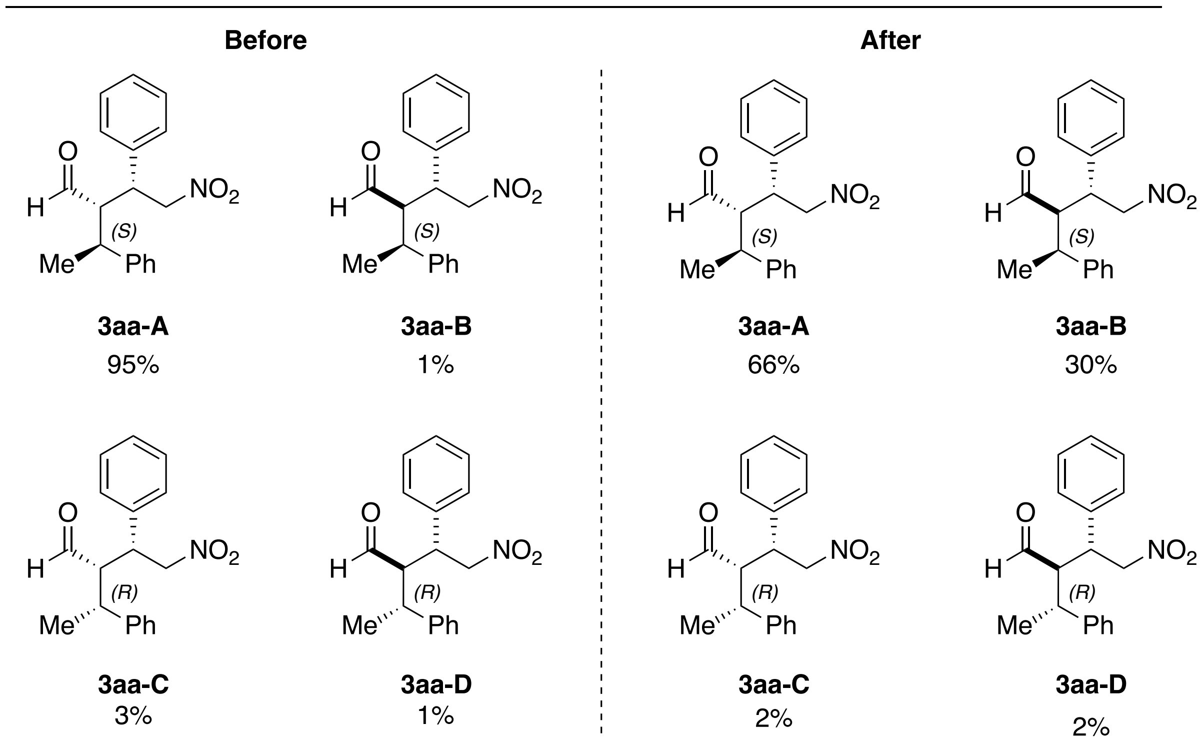

Scheme S1: Epimerization of **3aa** in the presence of pyrrolidine.

6.5 Analytical Data

(2*S*,3*R*)-4-Nitro-3-phenyl-2-((*S*)-1-phenylethyl)butanal (3aa)

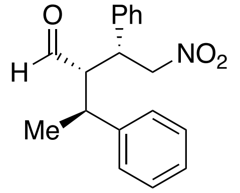

Following general procedure **IV**, **3aa** was obtained as a colorless oil (96% yield, 92:6:1:1 d.r., 97% ee). **^{1}H NMR** (500 MHz, $CDCl_3$, 25 °C) δ = 9.73 (d, *J* = 1.6 Hz, 1H), 7.45 – 7.31 (m, 5H), 7.31 – 7.23 (m, 3H), 7.22 – 7.16 (m, 2H), 4.74 (dd, *J* = 12.7, 4.3 Hz, 1H), 4.64 (dd, *J* = 12.7, 10.1 Hz, 1H), 3.86 (td, *J* = 9.8, 4.3 Hz, 1H), 3.26 (ddd, *J* = 9.5, 5.6, 1.7 Hz, 1H), 2.97 (qd, *J* = 7.1, 5.4 Hz, 1H), 1.27 (d, *J* = 7.2 Hz, 3H). **^{13}C NMR** (126 MHz, $CDCl_3$) δ = 204.0, 142.9, 137.3, 129.3, 128.9, 128.2, 127.9, 127.2, 127.2, 78.2, 59.6, 42.3, 38.1, 14.9. **IR** (ATR): 3025, 2924, 2849, 2741, 1716, 1547, 1493, 1450, 1377, 1202, 1084, 1029, 975, 909, 842, 762. **HRMS** (EI) m/z calcd. for $C_{18}H_{19}NO_3^+$ 298.1438 [*M*]$^+$; found: 298.1439.

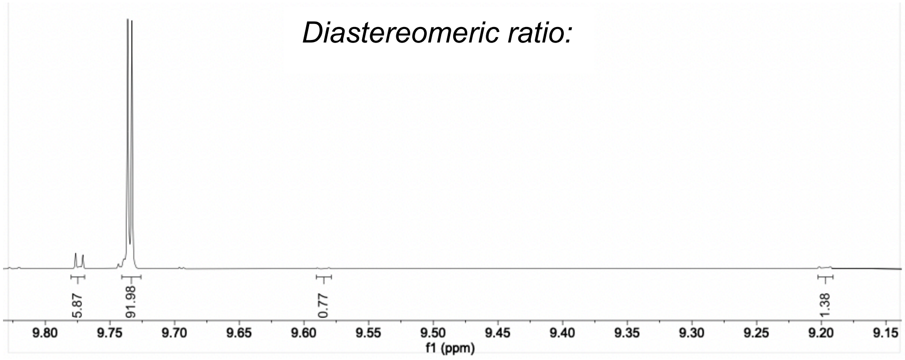

The enantiomeric excess was determined by chiral stationary phase HPLC with an IB-N5 column (*n*-hexane/*i*-PrOH 96:4, 25 °C) at 1 mL/min, UV detection at λ = 210 nm: t_R (major) = 32.5 min, t_R (minor) = 25.9 min.

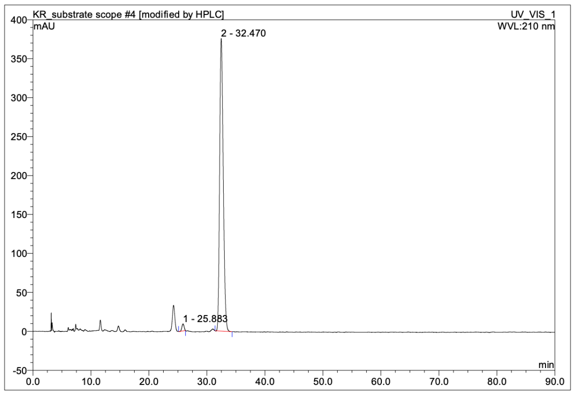

No.	Ret.Time min	Peak Name	Height mAU	Area mAU*min	Rel.Area %	Amount	Type
1	25.88	n.a.	8.953	3.893	1.41	n.a.	BMB*
2	32.47	n.a.	375.679	272.338	98.59	n.a.	BMB*
Total:			384.632	276.232	100.00	0.000	

Isolated product

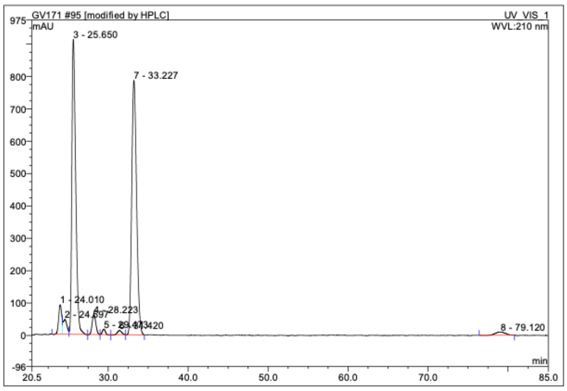

No.	Ret.Time min	Peak Name	Height mAU	Area mAU*min	Rel.Area %	Amount	Type
1	24.01	n.a.	91.100	39.922	3.37	n.a.	BM *
2	24.60	n.a.	45.570	26.697	2.25	n.a.	M *
3	25.65	n.a.	910.892	508.778	42.90	n.a.	M *
4	28.22	n.a.	61.463	32.059	2.70	n.a.	M *
5	29.47	n.a.	16.651	6.516	0.55	n.a.	M *
6	31.42	n.a.	12.774	7.065	0.60	n.a.	M *
7	33.23	n.a.	785.971	551.248	46.48	n.a.	MB*
8	79.12	n.a.	9.724	13.700	1.16	n.a.	BMB*
Total:			1934.145	1185.985	100.00	0.000	

Racemic sample

*(Using rac. peptide **A**)*

(2*S*,3*R*)-4-Nitro-3-phenyl-2-((*S*)-1-(*p*-tolyl)ethyl)butanal (3ba)

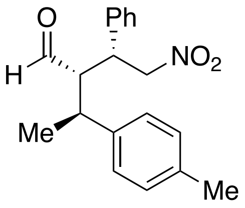

Following general procedure **IV**, **3ba** was obtained as a colorless oil (90% yield, 95:5:<1:<1 d.r., 99% ee). **^{1}H NMR** (500 MHz, $CDCl_3$) δ = 9.74 (d, *J* = 1.7 Hz, 1H), 7.46 – 7.37 (m, 2H), 7.37 – 7.31 (m, 1H), 7.27 – 7.22 (m, 2H), 7.17 (dt, *J* = 7.8, 0.8 Hz, 2H), 7.07 (d, *J* = 8.1 Hz, 2H), 4.73 (dd, *J* = 12.7, 4.3 Hz, 1H), 4.64 (dd, *J* = 12.7, 10.1 Hz, 1H), 3.85 (td, *J* = 9.8, 4.3 Hz, 1H), 3.23 (ddd, *J* = 9.4, 5.7, 1.7 Hz, 1H), 2.99 – 2.88 (m, 1H), 2.36 (s, 3H), 1.25 (d, *J* = 7.2 Hz, 3H). **^{13}C NMR** (126 MHz, $CDCl_3$) δ = 204.3, 139.8, 137.3, 136.8, 129.6, 129.3, 128.2, 127.9, 127.0, 78.1, 59.7, 42.3, 37.7, 21.0, 15.1. **IR** (ATR): 3024, 2968, 2921, 2832, 2734, 1718, 1547, 1511, 1493, 1453, 1427, 1379, 1194, 1101, 1049, 1031, 822, 761, 734, 703. **HRMS** (ESI) m/z calcd for $C_{19}H_{21}NNaO_3^+$ 334.1414 [*M* + Na]$^+$; found: 344.1413.

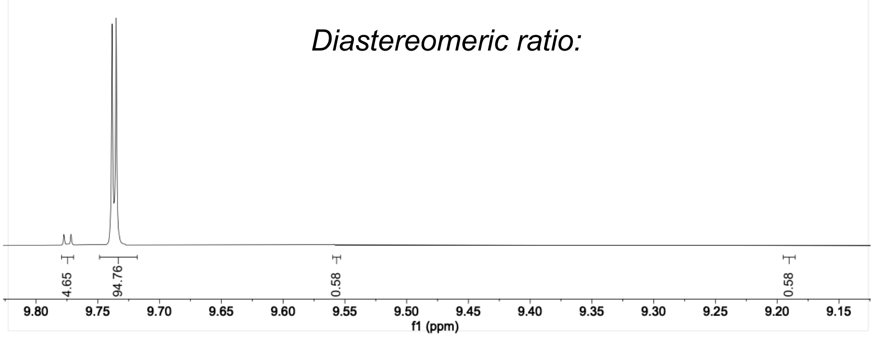

The enantiomeric excess was determined by chiral stationary phase HPLC with an IC column (*n*-hexane/*i*-PrOH 96:4, 25 °C) at 1 mL/min, UV detection at λ = 210 nm: t_R (major) = 28.0 min, t_R (minor) = 33.3 min.

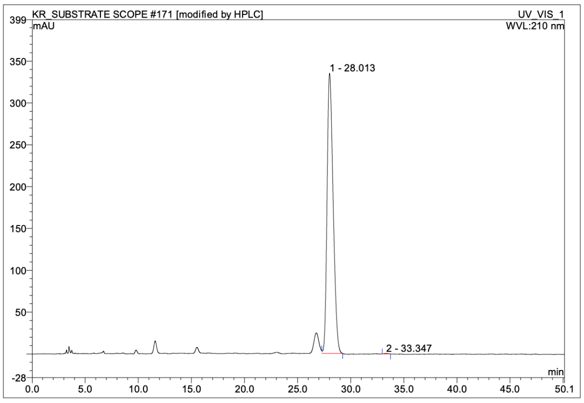

No.	Ret.Time min	Peak Name	Height mAU	Area mAU*min	Rel.Area %	Amount	Type
1	28.01	n.a.	334.861	221.154	99.90	n.a.	MB*
2	33.35	n.a.	0.532	0.215	0.10	n.a.	BMB*
Total:			335.393	221.370	100.00	0.000	

Isolated product

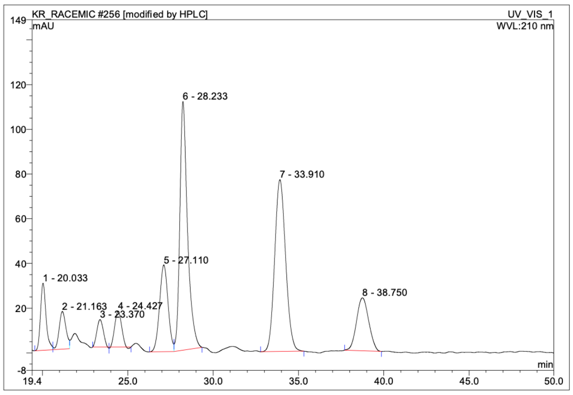

No.	Ret.Time min	Peak Name	Height mAU	Area mAU*min	Rel.Area %	Amount	Type
1	20.03	n.a.	30.047	11.318	5.98	n.a.	BM *
2	21.16	n.a.	16.854	7.841	4.14	n.a.	M *
3	23.37	n.a.	12.246	5.337	2.82	n.a.	BM *
4	24.43	n.a.	15.985	7.340	3.88	n.a.	MB*
5	27.11	n.a.	38.787	22.430	11.85	n.a.	BM *
6	28.23	n.a.	111.259	58.168	30.73	n.a.	MB*
7	33.91	n.a.	76.848	57.219	30.23	n.a.	BMB*
8	38.75	n.a.	23.684	19.615	10.36	n.a.	BMB*
Total:			325.710	189.268	100.00	0.000	

Racemic sample

(Using pyrrolidine)

(2*S*,3*R*)-2-((*S*)-1-(4-(*tert*-Butyl)phenyl)ethyl)-4-nitro-3-phenylbutanal (3ca)

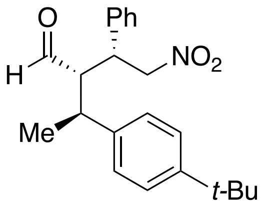

Following general procedure **IV** (10 mol% catalyst), **3ca** was obtained as a colorless oil (82% yield, 95:4:<1:1 d.r., 97% ee). **^{1}H NMR** (400 MHz, $CDCl_3$) δ = 9.74 (d, J = 1.6 Hz, 1H), 7.44 – 7.30 (m, 5H), 7.28 – 7.23 (m, 2H), 7.13 – 7.07 (m, 2H), 4.74 (dd, J = 12.6, 4.3 Hz, 1H), 4.62 (dd, J = 12.6, 10.0 Hz, 1H), 3.87 (td, J = 9.9, 4.3 Hz, 1H), 3.25 (ddd, J = 9.7, 5.3, 1.6 Hz, 1H), 2.98 – 2.88 (m, 1H), 1.33 (s, 9H), 1.25 (d, J = 7.2 Hz, 3H). **^{13}C NMR** (101 MHz, $CDCl_3$) δ = 204.3, 150.1, 139.8, 137.3, 129.3, 128.2, 127.9, 126.8, 125.7, 78.3, 59.5, 42.2, 37.5, 34.5, 31.3, 14.8. **IR** (ATR): 3060, 2962, 2847, 2746, 1714, 1551, 1511, 1494, 1456, 1436, 1410, 1386, 1269, 1204, 1099, 1083, 1017, 828, 761, 700. **HRMS** (ESI) m/z calcd for $C_{22}H_{27}NNaO_3^+$ 376.1883 [*M* + Na]$^+$; found: 376.1883.

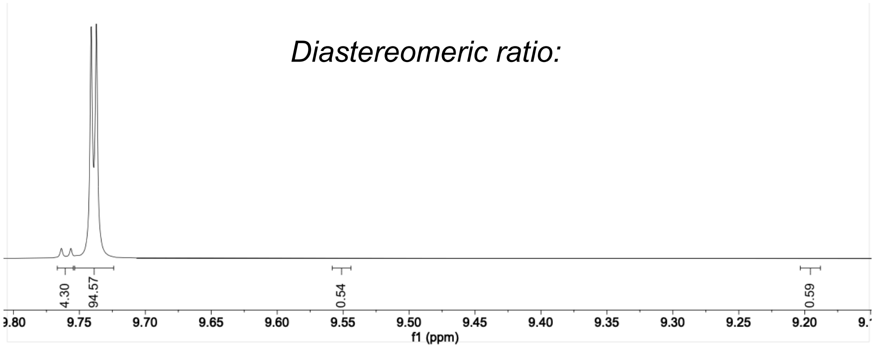

The enantiomeric excess was determined by chiral stationary phase HPLC with an IC column (*n*-hexane/*i*-PrOH 96:4, 40 °C) at 1 mL/min, UV detection at λ = 210 nm: t_R (major) = 15.6 min, t_R (minor) = 17.6 min.

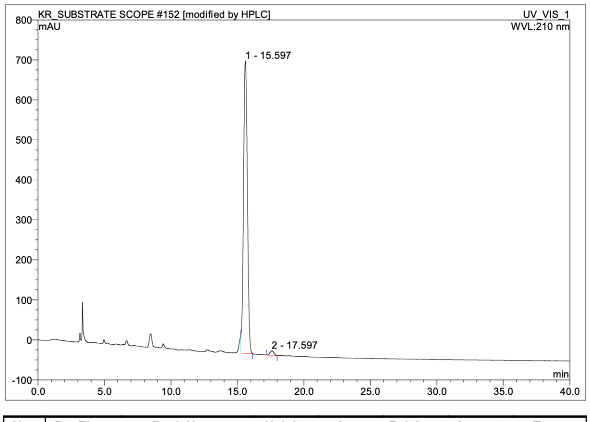

No.	Ret.Time min	Peak Name	Height mAU	Area mAU*min	Rel.Area %	Amount	Type
1	15.60	n.a.	730.937	248.172	98.53	n.a.	MB*
2	17.60	n.a.	11.383	3.700	1.47	n.a.	BMB*
Total:			742.320	251.872	100.00	0.000	

Isolated product

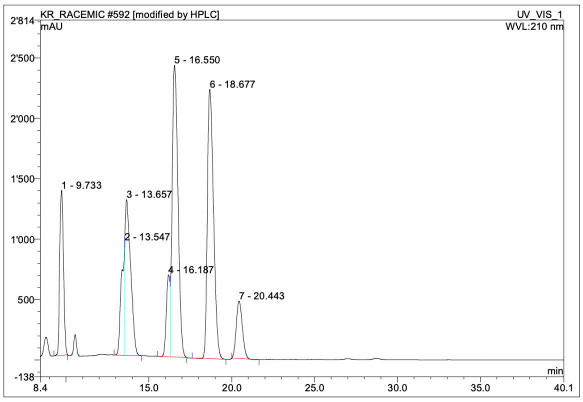

No.	Ret.Time min	Peak Name	Height mAU	Area mAU*min	Rel.Area %	Amount	Type
1	9.73	n.a.	1364.666	360.543	10.64	n.a.	BMB*
2	13.55	n.a.	938.156	216.521	6.39	n.a.	BM *
3	13.66	n.a.	1289.344	495.413	14.62	n.a.	MB*
4	16.19	n.a.	678.086	169.809	5.01	n.a.	BM *
5	16.55	n.a.	2414.158	981.956	28.98	n.a.	MB*
6	18.68	n.a.	2229.296	961.587	28.38	n.a.	BMB*
7	20.44	n.a.	476.437	202.707	5.98	n.a.	BMB*
Total:			9390.143	3388.537	100.00	0.000	

Racemic sample

(*Using pyrrolidine*)

(2*S*,3*R*)-2-((*S*)-1-(4-Methoxyphenyl)ethyl)-4-nitro-3-phenylbutanal (3da)

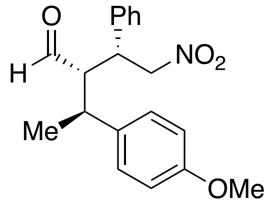

Following general procedure **IV**, **3da** was obtained as a light-yellow oil (92% yield, 94:5:<1:1 d.r., 98% ee). **^{1}H NMR** (400 MHz, $CDCl_3$) δ = 9.74 (d, *J* = 1.7 Hz, 1H), 7.45 – 7.30 (m, 3H), 7.28 – 7.23 (m, 2H), 7.14 – 7.06 (m, 2H), 6.94 – 6.87 (m, 2H), 4.73 (dd, *J* = 12.6, 4.3 Hz, 1H), 4.64 (dd, *J* = 12.6, 10.1 Hz, 1H), 3.88 – 3.81 (m, 1H), 3.83 (s, 3H), 3.21 (ddd, *J* = 9.3, 5.7, 1.7 Hz, 1H), 2.99 – 2.87 (m, 1H), 1.24 (d, *J* = 7.2 Hz, 3H). **^{13}C NMR** (101 MHz, $CDCl_3$) δ = 204.3, 158.6, 137.3, 134.8, 129.3, 128.2, 128.1, 127.9, 114.3, 78.1, 59.9, 55.3, 42.3, 37.3, 15.2. **IR** (ATR): 3022, 2965, 2922, 2842, 2744, 1712, 1607, 1549, 1509, 1452, 1381, 1304, 1242, 1180, 1124, 1102, 1029, 834, 761, 736, 701. **HRMS** (ESI) m/z calcd for $C_{19}H_{21}NNaO_4^+$ 350.1363 $[M + Na]^+$; found: 350.1359.

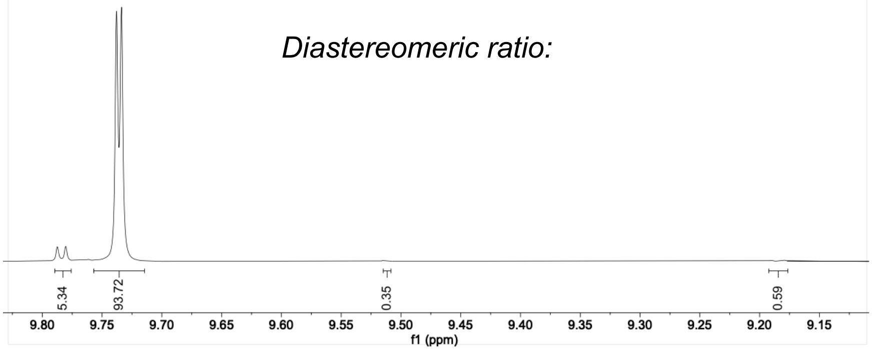

The enantiomeric excess was determined by chiral stationary phase HPLC with an AD-H column (*n*-hexane/*i*-PrOH 96:4, 25 °C) at 1 mL/min, UV detection at λ = 210 nm: t_R (major) = 17.6 min, t_R (minor) = 20.0 min.

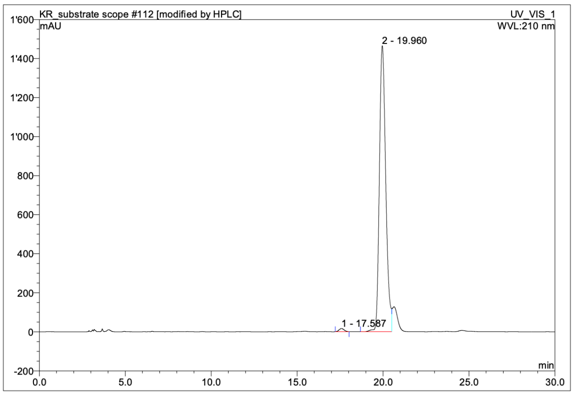

No.	Ret.Time min	Peak Name	Height mAU	Area mAU*min	Rel.Area %	Amount	Type
1	17.59	n.a.	16.234	5.539	0.87	n.a.	BMB*
2	19.96	n.a.	1465.023	632.063	99.13	n.a.	BM *
Total:			1481.257	637.601	100.00	0.000	

Isolated product

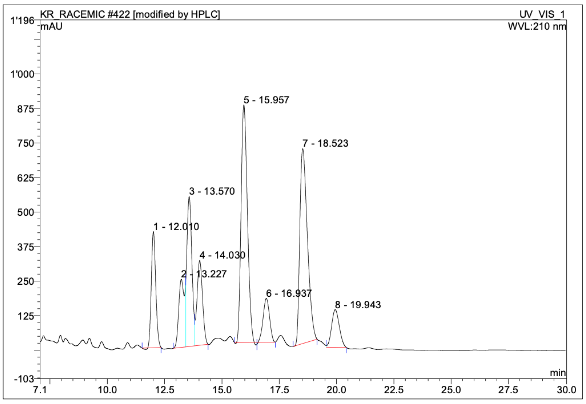

No.	Ret.Time min	Peak Name	Height mAU	Area mAU*min	Rel.Area %	Amount	Type
1	12.01	n.a.	420.629	93.880	9.03	n.a.	BMB*
2	13.23	n.a.	246.582	73.700	7.09	n.a.	BM *
3	13.57	n.a.	542.425	137.141	13.19	n.a.	M *
4	14.03	n.a.	306.618	86.500	8.32	n.a.	MB*
5	15.96	n.a.	859.820	272.869	26.25	n.a.	BM *
6	16.94	n.a.	158.638	51.526	4.96	n.a.	MB*
7	18.52	n.a.	705.724	270.868	26.05	n.a.	BMB*
8	19.94	n.a.	136.201	53.165	5.11	n.a.	MB*
Total:			3376.637	1039.648	100.00	0.000	

Racemic sample

(Using pyrrolidine)

(2*S*,3*R*)-4-Nitro-3-phenyl-2-((*S*)-1-(4-(trifluoromethyl)phenyl)ethyl)butanal (3ea)

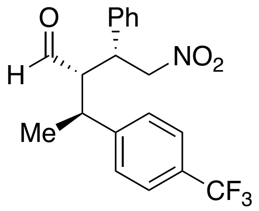

Following general procedure **IV** (10 mol% catalyst), **3ea** was obtained as a colorless oil (81% yield, >99:<1:<1:<1 d.r., 99% ee). **^{1}H NMR** (400 MHz, $CDCl_3$) δ = 9.72 (d, *J* = 1.8 Hz, 1H), 7.65 – 7.58 (m, 2H), 7.46 – 7.39 (m, 2H), 7.39 – 7.33 (m, 1H), 7.29 – 7.20 (m, 4H), 4.73 (dd, *J* = 12.6, 4.4 Hz, 1H), 4.62 (dd, *J* = 12.6, 9.8 Hz, 1H), 3.87 (td, *J* = 9.8, 4.4 Hz, 1H), 3.25 (ddd, *J* = 9.9, 5.4, 1.8 Hz, 1H), 3.01 (p, *J* = 7.0 Hz, 1H), 1.29 (d, *J* = 7.2 Hz, 3H). **^{13}C NMR** (101 MHz, $CDCl_3$) δ = 203.1, 147.0, 136.8, 129.5, 128.5, 127.8, 127.6, 125.9 (q, *J* = 3.8 Hz), 78.2, 59.2, 42.3, 38.1, 14.6 (due to heteronuclear ^{19}F-^{13}C coupling resulting in a distribution of the intensity over multiple signals for the $C_{Ar}CF_3$ group, some ^{13}C resonances could not be detected in ^{13}C{^{1}H} spectra even with increased number of scans and are thus not reported). **^{19}F NMR** (376 MHz, $CDCl_3$) δ = –62.5. **IR** (ATR): 2970, 2922, 2847, 2746, 1713, 1618, 1551, 1457, 1435, 1420, 1322, 1116, 1067, 1015, 945, 835, 761, 700. **HRMS** (ESI) m/z calcd for $C_{19}H_{18}F_3NNaO_3^+$ 388.1131 [*M* + Na]$^+$; found 388.1132.

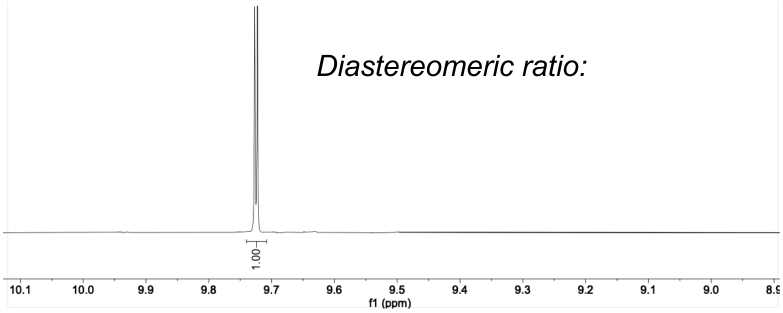

The enantiomeric excess was determined by chiral stationary phase HPLC with an IC column (*n*-hexane/*i*-PrOH 96:4, 25 °C) at 1 mL/min, UV detection at λ = 210 nm: t_R (major) = 32.7 min, t_R (minor) = 36.4 min.

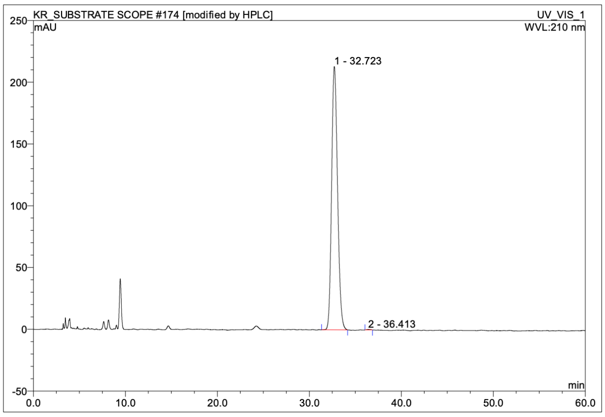

No.	Ret.Time min	Peak Name	Height mAU	Area mAU*min	Rel.Area %	Amount	Type
1	32.72	n.a.	213.167	159.275	99.88	n.a.	BMB*
2	36.41	n.a.	0.424	0.192	0.12	n.a.	BMB*
Total:			213.592	159.467	100.00	0.000	

Isolated product

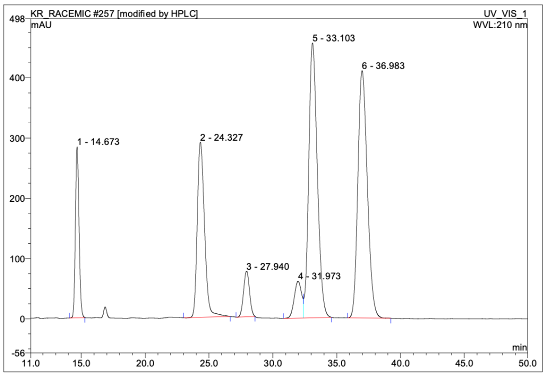

No.	Ret.Time min	Peak Name	Height mAU	Area mAU*min	Rel.Area %	Amount	Type
1	14.67	n.a.	283.655	93.446	8.70	n.a.	BMB*
2	24.33	n.a.	290.790	190.933	17.77	n.a.	BMB*
3	27.94	n.a.	75.858	40.875	3.80	n.a.	BMB*
4	31.97	n.a.	61.276	41.017	3.82	n.a.	BM *
5	33.10	n.a.	456.132	355.155	33.06	n.a.	MB*
6	36.98	n.a.	410.618	352.877	32.85	n.a.	BMB*
Total:			1578.329	1074.304	100.00	0.000	

Racemic sample

(Using pyrrolidine)

Methyl 4-((2*S*,3*S*,4*R*)-3-formyl-5-nitro-4-phenylpentan-2-yl)benzoate (3fa)

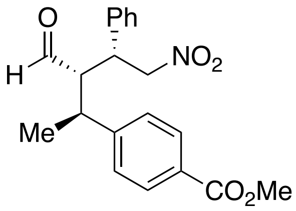

Following general procedure **IV** (10 mol% catalyst), **3fa** was obtained as a light-yellow oil (85% yield, 94:6:<1:<1 d.r., 93% ee). **^{1}H NMR** (400 MHz, $CDCl_3$) δ = 9.70 (d, J = 1.7 Hz, 1H), 8.07 – 8.01 (m, 2H), 7.46 – 7.39 (m, 2H), 7.38 – 7.34 (m, 1H), 7.27 – 7.21 (m, 4H), 4.73 (dd, J = 12.6, 4.4 Hz, 1H), 4.63 (dd, J = 12.7, 9.9 Hz, 1H), 3.94 (s, 3H), 3.86 (td, J = 9.8, 4.4 Hz, 1H), 3.26 (ddd, J = 9.7, 5.5, 1.7 Hz, 1H), 3.05 – 2.97 (m, 1H), 1.28 (d, J = 7.2 Hz, 3H). **^{13}C NMR** (101 MHz, $CDCl_3$) δ = 203.3, 166.7, 148.2, 136.9, 130.2, 129.4, 129.1, 128.4, 127.8, 127.3, 78.2, 59.2, 52.2, 42.3, 38.2, 14.6. **IR** (ATR): 2924, 2848, 2738, 1714, 1609, 1551, 1434, 1378, 1277, 1184, 1111, 1017, 965, 918, 859, 767, 702. **HRMS** (ESI) m/z calcd for $C_{20}H_{22}NNaO_5^+$ 378.1312 [M + Na]$^+$; found: 378.1310.

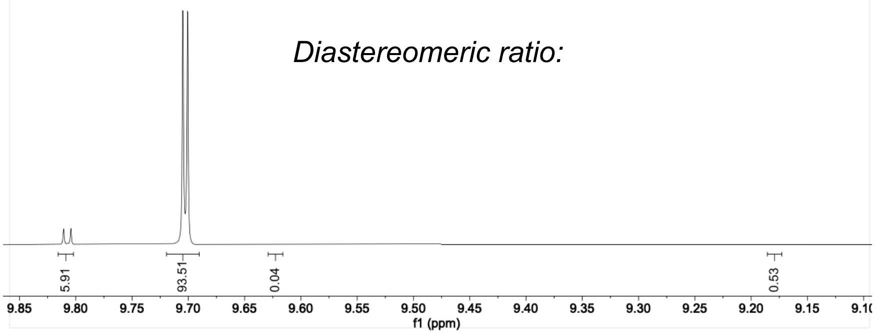

The enantiomeric excess was determined by chiral stationary phase HPLC with an AD-H column (*n*-hexane/*i*-PrOH 96:4, 25 °C) at 1 mL/min, UV detection at λ = 210 nm: t_R (major) = 43.8 min, t_R (minor) = 40.4 min.

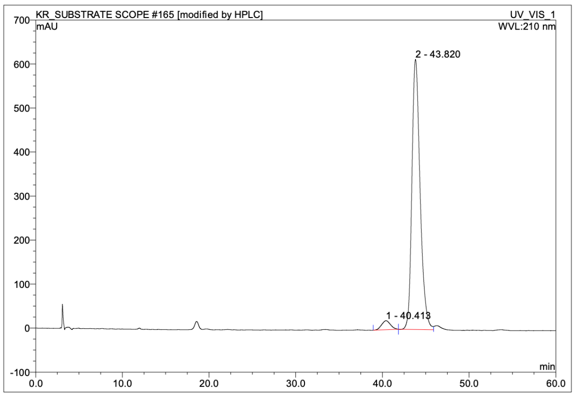

No.	Ret.Time min	Peak Name	Height mAU	Area mAU*min	Rel.Area %	Amount	Type
1	40.41	n.a.	20.445	24.148	3.47	n.a.	BMB*
2	43.82	n.a.	613.831	672.133	96.53	n.a.	BM *
Total:			634.276	696.281	100.00	0.000	

Isolated product

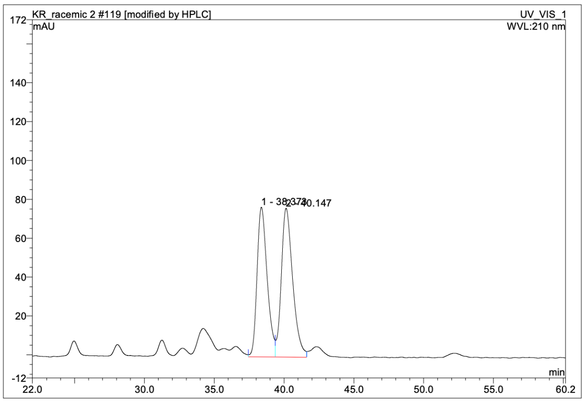

No.	Ret.Time min	Peak Name	Height mAU	Area mAU*min	Rel.Area %	Amount	Type
1	38.37	n.a.	77.058	64.582	47.07	n.a.	M *
2	40.15	n.a.	76.622	72.631	52.93	n.a.	M *
Total:			153.680	137.213	100.00	0.000	

Racemic sample

(Using rac. peptide ***A****)*

(2*S*,3*R*)-2-((*S*)-1-(4-Fluorophenyl)ethyl)-4-nitro-3-phenylbutanal (3ga)

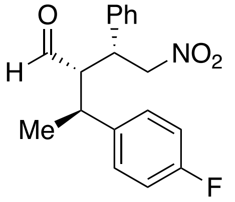

Following general procedure **IV**, **3ga** was obtained as a colorless oil (65% yield, 99:<1:<1:1 d.r., 99% ee). **^{1}H NMR** (400 MHz, $CDCl_3$) δ = 9.72 (d, J = 1.7 Hz, 1H), 7.45 – 7.38 (m, 2H), 7.38 – 7.32 (m, 1H), 7.27 – 7.22 (m, 2H), 7.19 – 7.10 (m, 2H), 7.09 – 7.00 (m, 2H), 4.73 (dd, J = 12.6, 4.4 Hz, 1H), 4.62 (dd, J = 12.6, 9.9 Hz, 1H), 3.85 (td, J = 9.8, 4.4 Hz, 1H), 3.21 (ddd, J = 9.7, 5.5, 1.7 Hz, 1H), 3.03 – 2.88 (m, 1H), 1.25 (d, J = 7.2 Hz, 3H). **^{13}C NMR** (101 MHz, $CDCl_3$) δ = 203.8, 161.7 (d, J = 246.0 Hz), 138.6 (d, J = 3.4 Hz), 137.1, 129.4, 128.7 (d, J = 7.9 Hz), 128.3, 127.8, 115.8 (d, J = 21.3 Hz), 78.2, 59.6 (d, J = 1.0 Hz), 42.3, 37.5, 15.0. **^{19}F NMR** (376 MHz, $CDCl_3$) δ = –115.3 (tt, J = 8.3, 5.2 Hz). **IR** (ATR): 3026, 2971, 2919, 2835, 2737, 1720, 1600, 1549, 1505, 1454, 1429, 1379, 1218, 1160, 1084, 838, 761. **HRMS** (ESI) m/z calcd for $C_{18}H_{18}FNNaO_3^+$ 338.1163 [M + Na]$^+$; found: 338.1160.

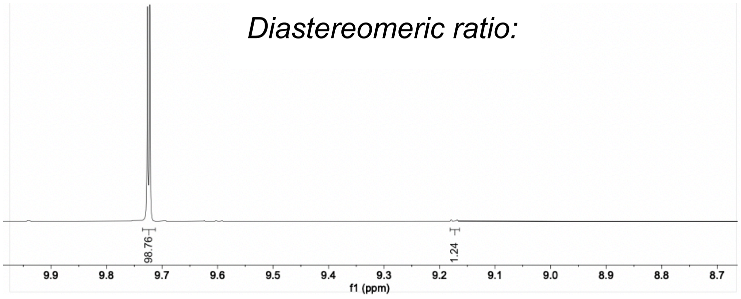

The enantiomeric excess was determined by chiral stationary phase HPLC with an IC column (*n*-hexane/*i*-PrOH 95:5, 25 °C) at 1 mL/min, UV detection at λ = 210 nm: t_R (major) = 35.8 min, t_R (minor) = 37.8 min.

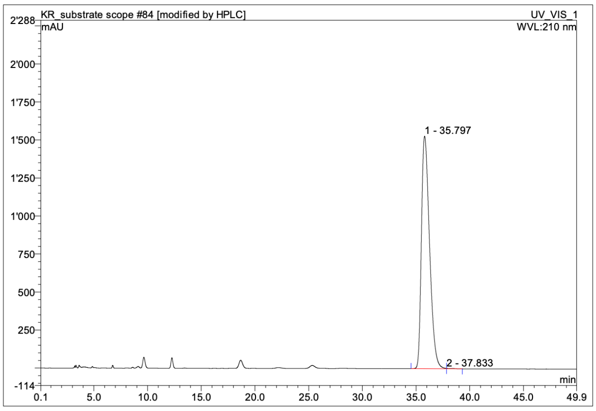

No.	Ret.Time min	Peak Name	Height mAU	Area mAU*min	Rel.Area %	Amount	Type
1	35.80	n.a.	1529.206	1338.075	99.84	n.a.	BM *
2	37.83	n.a.	2.934	2.195	0.16	n.a.	MB*
Total:			1532.140	1340.270	100.00	0.000	

Isolated product

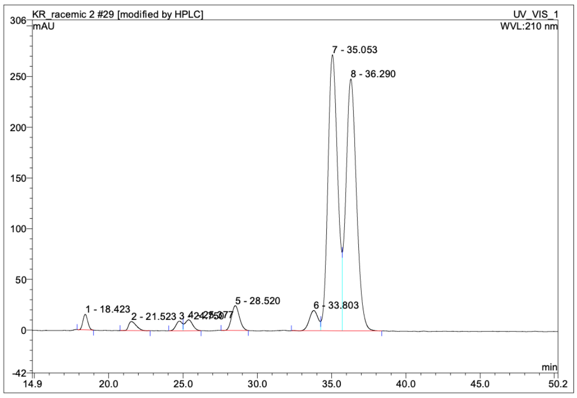

No.	Ret.Time min	Peak Name	Height mAU	Area mAU*min	Rel.Area %	Amount	Type
1	18.42	n.a.	15.317	5.655	1.22	n.a.	BMB*
2	21.52	n.a.	8.988	5.332	1.15	n.a.	BMB*
3	24.75	n.a.	9.523	4.646	1.00	n.a.	BM *
4	25.38	n.a.	10.774	6.613	1.43	n.a.	MB*
5	28.52	n.a.	24.441	14.888	3.22	n.a.	BMB*
6	33.80	n.a.	19.916	13.534	2.92	n.a.	BM *
7	35.05	n.a.	272.123	207.481	44.83	n.a.	M *
8	36.29	n.a.	248.344	204.711	44.23	n.a.	MB*
Total:			609.426	462.860	100.00	0.000	

Racemic sample

(Using rac. peptide **A***)*

(2*S*,3*R*)-2-((*S*)-1-(3-Fluorophenyl)ethyl)-4-nitro-3-phenylbutanal (3ha)

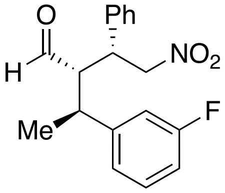

Following general procedure **IV**, **3ha** was obtained as a colorless oil (58% yield, 96:2:1:1 d.r., 99% ee). **^{1}H NMR** (400 MHz, $CDCl_3$) δ = 9.71 (d, J = 1.7 Hz, 1H), 7.46 – 7.39 (m, 2H), 7.38 – 7.29 (m, 2H), 7.28 – 7.24 (m, 2H), 7.02 – 6.94 (m, 2H), 6.91 – 6.85 (m, 1H), 4.74 (dd, J = 12.6, 4.3 Hz, 1H), 4.62 (dd, J = 12.6, 10.0 Hz, 1H), 3.87 (td, J = 9.9, 4.3 Hz, 1H), 3.24 (ddd, J = 9.8, 5.3, 1.7 Hz, 1H), 3.00 – 2.90 (m, 1H), 1.25 (d, J = 7.2 Hz, 3H). **^{13}C NMR** (101 MHz, $CDCl_3$) δ = 203.5, 163.1 (d, J = 247.0 Hz), 145.6 (d, J = 6.7 Hz), 137.0, 130.5 (d, J = 8.4 Hz), 129.4, 128.4, 127.8, 122.9 (d, J = 2.9 Hz), 114.3 (d, J = 4.0 Hz), 114.1 (d, J = 3.6 Hz), 78.2, 59.3, 42.2, 37.8 (d, J = 1.7 Hz), 14.6. **^{19}F NMR** (376 MHz, $CDCl_3$) δ = –112.1 (ddd, J = 10.0, 8.3, 6.1 Hz). **IR** (ATR): 3029, 2915, 2846, 2742, 1716, 1587, 1544, 1484, 1443, 1375, 1271, 1220, 1148, 1101, 972, 930, 873, 834. **HRMS** (ESI) m/z calcd for $C_{18}H_{18}FNNaO_3^+$ 338.1163 [M + Na]$^+$; found: 338.1160.

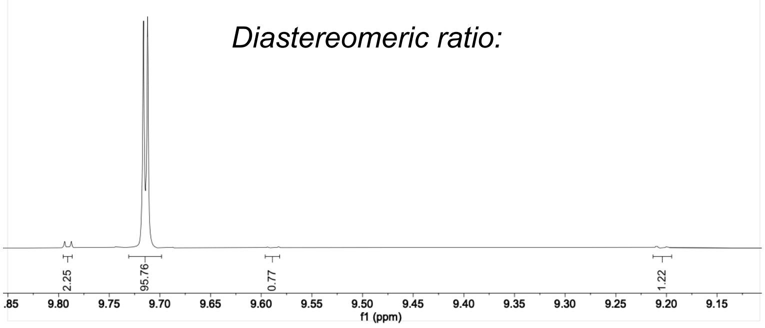

The enantiomeric excess was determined by chiral stationary phase HPLC with an IC column (*n*-hexane/*i*-PrOH 96:4, 40 °C) at 1 mL/min, UV detection at λ = 210 nm: t_R (major) = 26.6 min, t_R (minor) = 23.1 min.

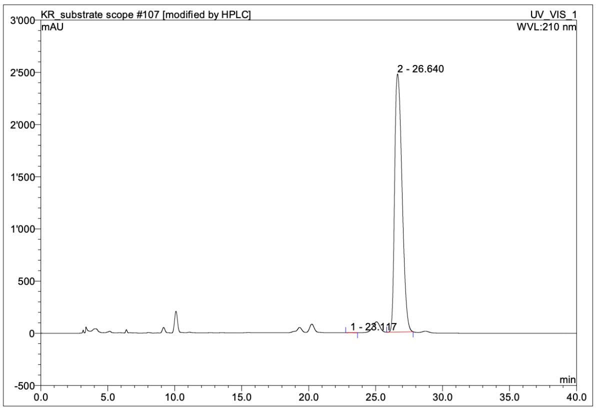

No.	Ret.Time min	Peak Name	Height mAU	Area mAU*min	Rel.Area %	Amount	Type
1	23.12	n.a.	1.992	0.738	0.05	n.a.	BMB*
2	26.64	n.a.	2472.281	1581.815	99.95	n.a.	BMB*
Total:			2474.273	1582.553	100.00	0.000	

Isolated product

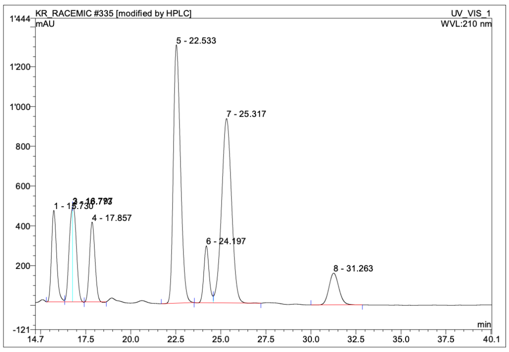

No.	Ret.Time min	Peak Name	Height mAU	Area mAU*min	Rel.Area %	Amount	Type
1	15.73	n.a.	459.796	149.798	7.88	n.a.	BM *
2	16.77	n.a.	480.893	102.530	5.39	n.a.	M *
3	16.80	n.a.	483.719	117.156	6.16	n.a.	M *
4	17.86	n.a.	400.766	139.883	7.36	n.a.	MB*
5	22.53	n.a.	1298.902	596.620	31.37	n.a.	BMB*
6	24.20	n.a.	285.860	96.146	5.06	n.a.	BM *
7	25.32	n.a.	927.071	597.494	31.42	n.a.	MB*
8	31.26	n.a.	160.128	102.252	5.38	n.a.	BMB*
Total:			4497.136	1901.880	100.00	0.000	

Racemic sample

(Using pyrrolidine)

(2*S*,3*R*)-2-((*S*)-1-(2-Fluorophenyl)ethyl)-4-nitro-3-phenylbutanal (3ia)

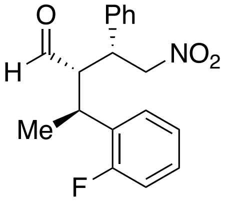

Following general procedure **IV**, **3ia** was obtained as a colorless oil (64% yield, 95:2:2:1 d.r., 82% ee). **^{1}H NMR** (400 MHz, $CDCl_3$) δ = 9.70 (d, *J* = 1.5 Hz, 1H), 7.45 – 7.37 (m, 2H), 7.37 – 7.24 (m, 4H), 7.23 – 7.05 (m, 3H), 4.74 (dd, *J* = 12.6, 4.2 Hz, 1H), 4.62 (dd, *J* = 12.6, 10.1 Hz, 1H), 3.88 (td, *J* = 9.9, 4.2 Hz, 1H), 3.34 (ddd, *J* = 9.6, 5.5, 1.5 Hz, 1H), 3.31 – 3.21 (m, 1H), 1.25 (d, *J* = 7.2 Hz, 3H). **^{13}C NMR** (101 MHz, $CDCl_3$) δ = 203.7, 160.6 (d, *J* = 245.5 Hz, C_{Ar}–F), 136.7, 129.5 (d, *J* = 13.9 Hz, C_{Ar}), 129.2, 128.8 (d, *J* = 8.5 Hz, C_{Ar}–H), 128.2, 128.2 (d, *J* = 4.4 Hz, C_{Ar}–H), 128.1, 124.5 (d, *J* = 3.4 Hz, C_{Ar}–H), 115.9 (d, *J* = 22.4 Hz, C_{Ar}–H), 78.4, 56.9 (d, *J* = 1.5 Hz, *C*–H), 42.3, 31.6 (d, *J* = 1.7 Hz, *C*–H), 13.8. **^{19}F NMR** (376 MHz, $CDCl_3$) δ = –116.6 (ddd, *J* = 12.0, 7.1, 5.4 Hz). **IR** (ATR): 3408, 3038, 2969, 2917, 2854, 2755, 1715, 1553, 1487, 1453, 1382, 1288, 1213, 1121, 1083, 1032, 974, 939, 845, 753, 703. **HRMS** (ESI) m/z calcd for $C_{18}H_{18}FNNaO_3^+$ 338.1163 [*M* + Na]$^+$; found: 338.1160.

Diastereomeric ratio:

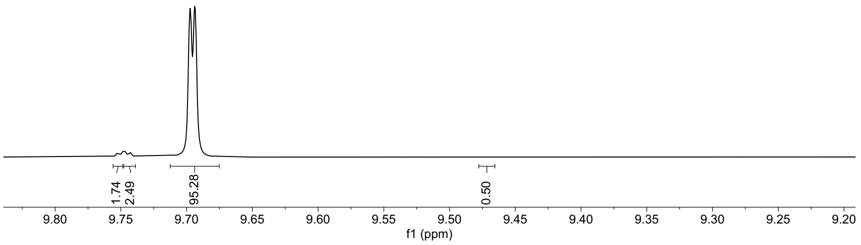

The enantiomeric excess was determined by chiral stationary phase HPLC with an IC column (*n*-hexane/*i*-PrOH 98:2, 25 °C) at 1 mL/min, UV detection at λ = 210 nm: t_R (major) = 49.8 min, t_R (minor) = 57.9 min.

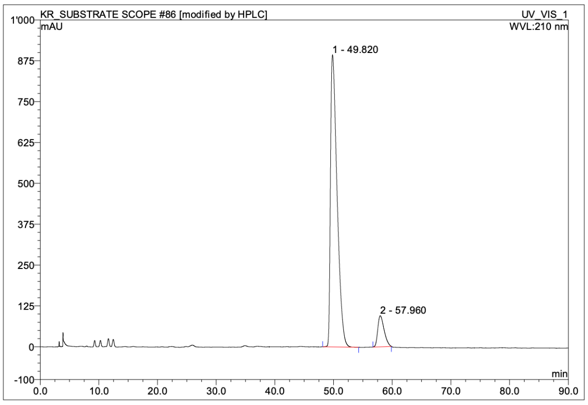

No.	Ret.Time min	Peak Name	Height mAU	Area mAU*min	Rel.Area %	Amount	Type
1	49.82	n.a.	893.631	1162.317	90.72	n.a.	BMB*
2	57.96	n.a.	95.068	118.839	9.28	n.a.	BMB*
Total:			988.699	1281.156	100.00	0.000	

Isolated product

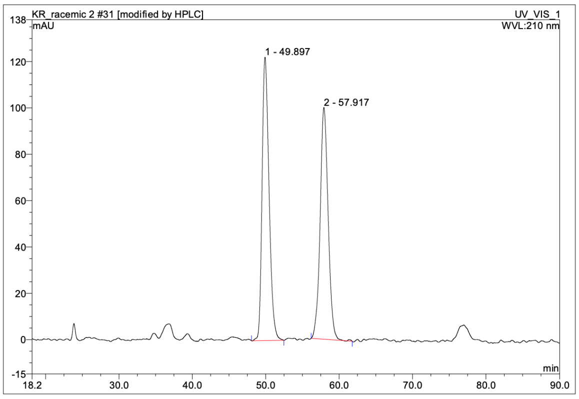

No.	Ret.Time min	Peak Name	Height mAU	Area mAU*min	Rel.Area %	Amount	Type
1	49.90	n.a.	122.347	134.327	51.50	n.a.	BMB*
2	57.92	n.a.	100.031	126.508	48.50	n.a.	BMB*
Total:			222.378	260.835	100.00	0.000	

Racemic sample

(Using rac. peptide **A***)*

(2*S*,3*S*)-3-(4-Fluorophenyl)-2-((*R*)-2-nitro-1-phenylethyl)pentanal (3ka)

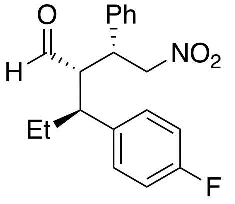

Following general procedure **IV** (10 mol% catalyst), **3ka** was obtained as a colorless oil (65% yield, 92:7:<1:1 d.r., 98% ee). **^{1}H NMR** (500 MHz, $CDCl_3$) δ = 9.88 (d, J = 2.1 Hz, 1H), 7.43 – 7.35 (m, 3H), 7.35 – 7.30 (m, 1H), 7.20 – 7.14 (m, 2H), 7.12 – 7.03 (m, 5H), 4.69 (d, J = 4.1 Hz, 1H), 4.67 (d, J = 0.8 Hz, 1H), 3.80 (td, J = 8.6, 5.7 Hz, 1H), 3.15 (ddd, J = 8.2, 6.6, 2.1 Hz, 1H), 2.70 (ddd, J = 11.7, 6.5, 3.4 Hz, 1H), 1.77 (dqd, J = 13.7, 7.4, 3.4 Hz, 1H), 1.61 (ddq, J = 13.9, 11.5, 7.0 Hz, 1H), 0.66 (t, J = 7.3 Hz, 3H). **^{13}C NMR** (126 MHz, $CDCl_3$) δ = 204.0, 161.8 (d, J = 246.2 Hz), 137.4, 136.5 (d, J = 3.4 Hz), 129.4 (d, J = 7.9 Hz), 129.3, 128.2, 127.7, 115.9 (d, J = 21.3 Hz), 77.3, 60.2 (d, J = 1.0 Hz), 46.0, 42.5, 23.8, 12.1. **^{19}F NMR** (471 MHz, $CDCl_3$) δ = –115.1 (tt, J = 8.4, 5.6 Hz). **IR** (ATR): 3034, 2967, 2930, 2876, 2843, 2740, 1715, 1549, 1506, 1457, 1433, 1384, 1289, 1222, 1201, 1159, 1121, 1093, 1032, 1013, 987, 914, 863, 837, 762, 699. **HRMS** (ESI) m/z calcd for $C_{19}H_{20}FNNaO_3^+$ 352.1319 [M + Na]$^+$; found: 352.1322.

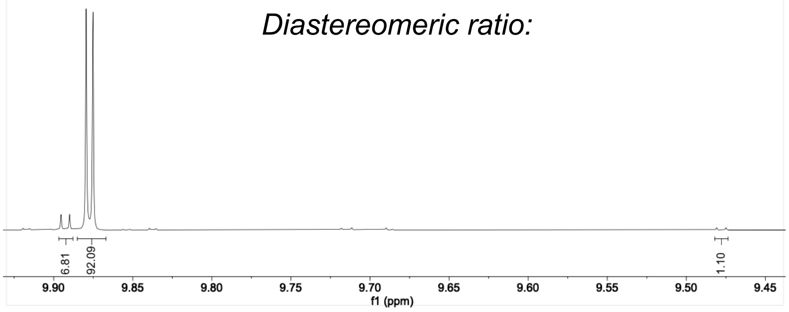

The enantiomeric excess was determined by chiral stationary phase HPLC with an IC column (*n*-hexane/*i*-PrOH 96:4, 25 °C) at 1 mL/min, UV detection at λ = 210 nm: t_R (major) = 35.4 min, t_R (minor) = 38.8 min.

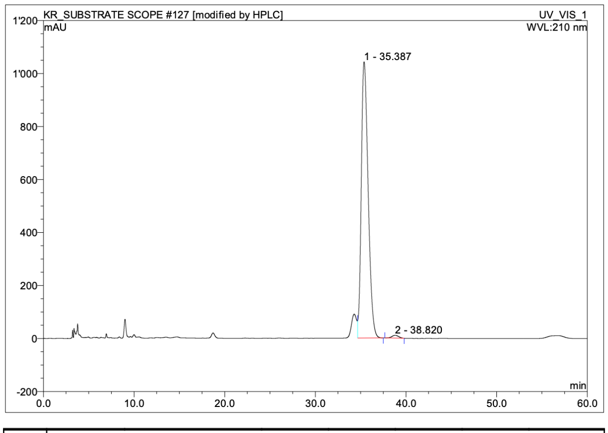

No.	Ret.Time min	Peak Name	Height mAU	Area mAU*min	Rel.Area %	Amount	Type
1	35.39	n.a.	1042.799	915.620	99.01	n.a.	MB*
2	38.82	n.a.	10.535	9.120	0.99	n.a.	BMB*
Total:			1053.333	924.740	100.00	0.000	

Isolated product

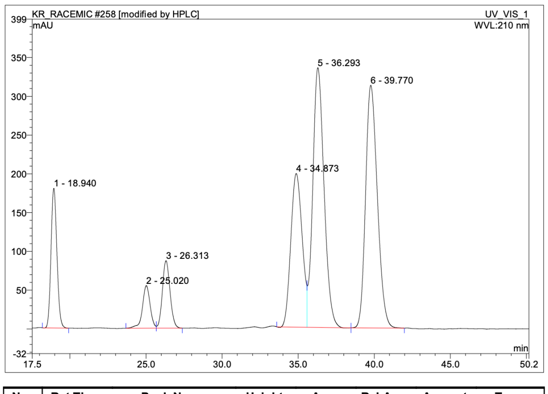

No.	Ret.Time min	Peak Name	Height mAU	Area mAU*min	Rel.Area %	Amount	Type
1	18.94	n.a.	180.196	80.783	8.93	n.a.	BMB*
2	25.02	n.a.	54.915	32.134	3.55	n.a.	BM *
3	26.31	n.a.	87.030	50.721	5.61	n.a.	MB*
4	34.87	n.a.	198.091	171.082	18.92	n.a.	BM *
5	36.29	n.a.	334.999	289.214	31.98	n.a.	Mb*
6	39.77	n.a.	313.040	280.480	31.01	n.a.	bMB*
Total:			1168.271	904.413	100.00	0.000	

Racemic sample

(Using pyrrolidine)

((2*S*,3*S*)-3-(4-Fluorophenyl)-2-((*R*)-2-nitro-1-phenylethyl)hexanal (3la)

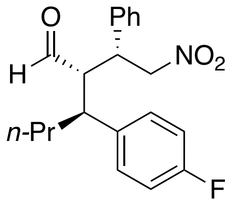

Following general procedure **IV** (10 mol% catalyst), **3la** was obtained as a colorless oil (75% yield, 98:<1:1:1 d.r., 99% ee). **^{1}H NMR** (400 MHz, $CDCl_3$) δ = 9.88 (d, J = 2.1 Hz, 1H), 7.46 – 7.30 (m, 3H), 7.22 – 7.15 (m, 2H), 7.13 – 7.00 (m, 4H), 4.68 (s, 1H), 4.67 (d, J = 1.0 Hz, 1H), 3.81 (td, J = 8.1, 6.9 Hz, 1H), 3.13 (ddd, J = 8.4, 6.3, 2.1 Hz, 1H), 2.81 (dt, J = 7.8, 6.2 Hz, 1H), 1.68 – 1.59 (m, 2H), 1.18 – 1.03 (m, 1H), 0.99 – 0.83 (m, 1H), 0.74 (t, J = 7.3 Hz, 3H). **^{13}C NMR** (101 MHz, $CDCl_3$) δ = 204.0, 161.8 (d, J = 246.1 Hz), 137.3, 136.8 (d, J = 3.4 Hz), 129.4 (d, overlapping), 129.3, 128.2, 127.8, 115.9 (d, J = 21.3 Hz), 77.4, 60.0, 43.7, 42.5, 32.5, 20.4, 13.6. **IR** (ATR): 2959, 2931, 2872, 2828, 2730, 1711, 1602, 1551, 1507, 1431, 1379, 1226, 1191, 1159, 1120, 1092, 834, 764, 743, 702. **HRMS** (ESI) m/z calcd for $C_{20}H_{22}FNNaO_3^+$ 366.1476 [*M* + Na]$^+$; found: 366.1478.

Diastereomeric ratio:

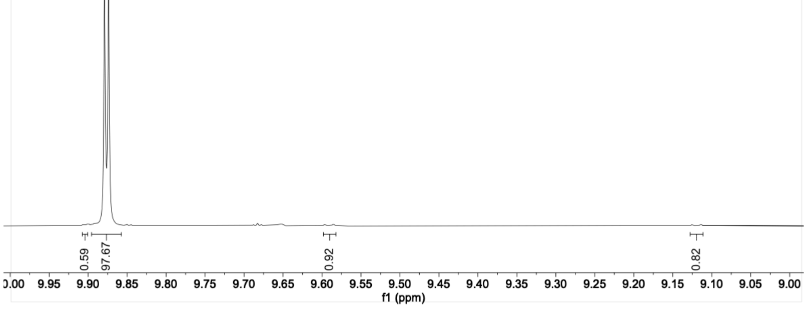

The enantiomeric excess was determined by chiral stationary phase HPLC with an IC column (*n*-hexane/*i*-PrOH 98:2, 40 °C) at 1 mL/min, UV detection at λ = 210 nm: t_R (major) = 48.9 min, t_R (minor) = 55.6 min.

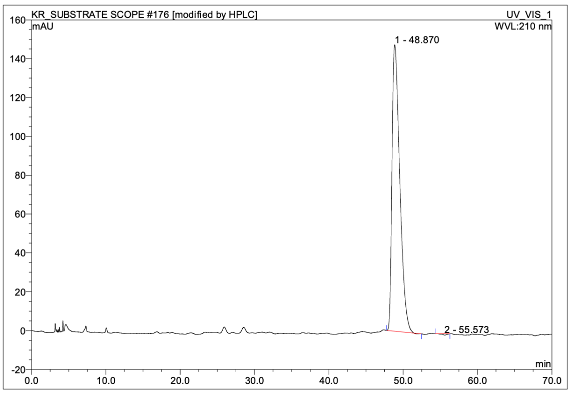

No.	Ret.Time min	Peak Name	Height mAU	Area mAU*min	Rel.Area %	Amount	Type
1	48.87	n.a.	147.523	178.709	99.73	n.a.	BMB*
2	55.57	n.a.	0.677	0.489	0.27	n.a.	BMB*
Total:			148.200	179.198	100.00	0.000	

Isolated product

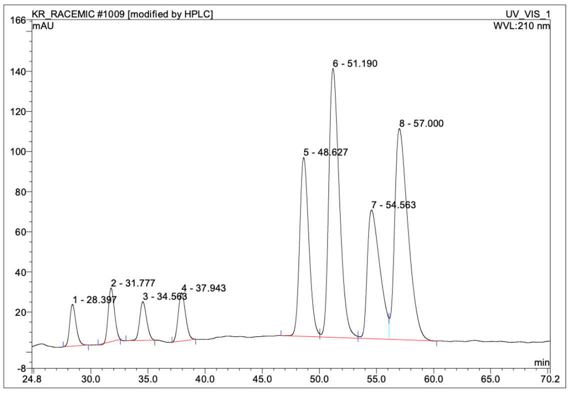

No.	Ret.Time min	Peak Name	Height mAU	Area mAU*min	Rel.Area %	Amount	Type
1	28.40	n.a.	20.795	13.440	2.62	n.a.	BMB*
2	31.78	n.a.	26.659	16.739	3.26	n.a.	BMB*
3	34.56	n.a.	19.323	13.636	2.66	n.a.	BMB*
4	37.94	n.a.	24.056	17.914	3.49	n.a.	BMB*
5	48.63	n.a.	89.206	83.378	16.24	n.a.	BM *
6	51.19	n.a.	134.141	143.641	27.97	n.a.	M *
7	54.56	n.a.	64.270	81.821	15.93	n.a.	M *
8	57.00	n.a.	105.292	142.951	27.84	n.a.	MB*
Total:			483.743	513.521	100.00	0.000	

Racemic sample

(Using pyrrolidine)

(2*S*,3*S*)-3-(4-Fluorophenyl)-2-((*R*)-2-nitro-1-phenylethyl)dodecanal (3ma)

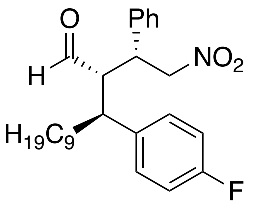

Following general procedure **IV** (10 mol% catalyst, 48 h), **3ma** was obtained as a colorless oil (72% yield, 83:7:6:4 d.r., 99% ee). **^{1}H NMR** (500 MHz, $CDCl_3$) δ = 9.87 (d, J = 2.1 Hz, 1H), 7.44 – 7.31 (m, 4H), 7.22 – 7.13 (m, 2H), 7.12 – 6.94 (m, 3H), 4.68 (s, 1H), 4.66 (d, J = 1.5 Hz, 1H), 3.81 (td, J = 8.2, 6.5 Hz, 1H), 3.12 (ddd, J = 8.4, 6.3, 2.1 Hz, 1H), 2.78 (ddd, J = 10.5, 6.2, 3.7 Hz, 1H), 1.71 – 1.55 (m, 2H), 1.33 – 1.09 (m, 14H), 0.89 (t, J = 7.1 Hz, 3H). **^{13}C NMR** (126 MHz, $CDCl_3$) δ = 204.0, 161.8 (d, J = 246.1 Hz), 137.4, 136.8 (d, J = 3.3 Hz), 129.4 (d, overlapping), 129.3, 128.2, 127.8, 115.9 (d, J = 21.2 Hz), 77.5, 60.1, 44.1, 42.5, 31.8, 30.3, 29.4, 29.3, 29.2, 29.1, 27.3, 22.7, 14.1. **^{19}F NMR** (471 MHz, $CDCl_3$) δ = –115.10 (tt, J = 8.3, 5.5 Hz). **IR** (ATR): 2923, 2853, 2733, 1718, 1603, 1552, 1508, 1456, 1433, 1378, 1223, 1159, 833, 759, 700. **HRMS** (ESI) m/z calcd for $C_{26}H_{34}FNNaO_3^+$ 450.2415 [M + Na]$^+$; found: 450.2411.

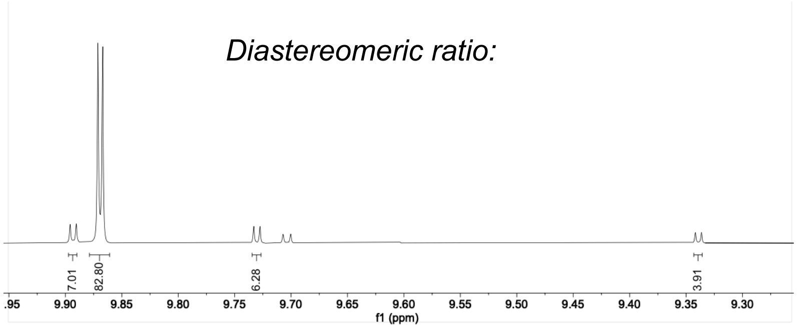

The enantiomeric excess was determined by chiral stationary phase HPLC with an IC column (*n*-hexane/*i*-PrOH 98.5:1.5, 40 °C) at 1 mL/min, UV detection at λ = 210 nm: t_R (major) = 57.2 min, t_R (minor) = 45.1 min.

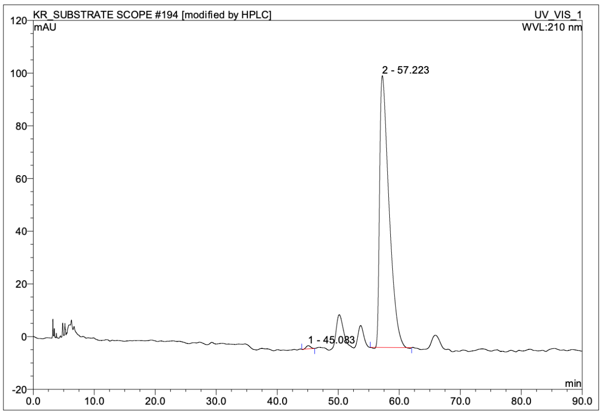

No.	Ret.Time min	Peak Name	Height mAU	Area mAU*min	Rel.Area %	Amount	Type
1	45.08	n.a.	1.247	0.982	0.53	n.a.	BMB*
2	57.22	n.a.	103.180	184.035	99.47	n.a.	BMB*
Total:			104.427	185.017	100.00	0.000	

Isolated product

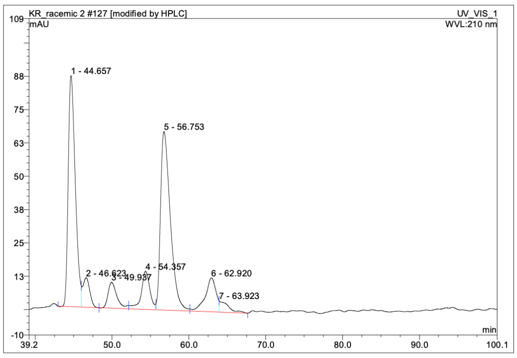

No.	Ret.Time min	Peak Name	Height mAU	Area mAU*min	Rel.Area %	Amount	Type
1	44.66	n.a.	86.705	92.211	35.22	n.a.	BM *
2	46.62	n.a.	11.086	11.977	4.57	n.a.	M *
3	49.94	n.a.	9.723	12.926	4.94	n.a.	M *
4	54.36	n.a.	14.338	18.759	7.17	n.a.	M *
5	56.75	n.a.	66.975	99.301	37.93	n.a.	M *
6	62.92	n.a.	12.758	20.661	7.89	n.a.	M *
7	63.92	n.a.	4.311	5.958	2.28	n.a.	MB*
Total:			205.895	261.794	100.00	0.000	

Racemic sample

(Using rac. peptide **A***)*

(2*S*,3*S*)-3-(4-Fluorophenyl)-4-methyl-2-((*R*)-2-nitro-1-phenylethyl)pentanal (3na)

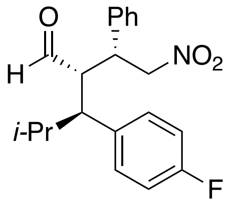

Following general procedure **IV** (10 mol% catalyst, 4 d), **3na** was obtained as a colorless oil (58% yield, 89:7:<1:4 d.r., 99% ee). **^{1}H NMR** (500 MHz, $CDCl_3$) δ = 9.96 (dd, J = 2.6, 0.5 Hz, 1H), 7.35 – 7.27 (m, 3H), 7.15 (ddt, J = 8.1, 5.1, 2.5 Hz, 2H), 7.13 – 7.05 (m, 4H), 4.85 (dd, J = 13.4, 11.0 Hz, 1H), 4.57 (dd, J = 13.4, 4.1 Hz, 1H), 3.64 (dt, J = 11.0, 4.3 Hz, 1H), 3.44 (ddd, J = 9.7, 4.5, 2.5 Hz, 1H), 2.99 (dd, J = 9.7, 5.9 Hz, 1H), 1.98 (pd, J = 6.7, 5.8 Hz, 1H), 0.82 (t, J = 6.7 Hz, 6H). **^{13}C NMR** (126 MHz, $CDCl_3$) δ = 204.3, 162.0 (d, J = 246.5 Hz), 138.2, 133.9 (d, J = 3.4 Hz), 130.5 (d, J = 7.9 Hz), 129.2, 128.0, 127.6, 115.7 (d, J = 21.3 Hz), 75.8, 57.7, 50.9, 43.2, 30.3 (d, J = 0.9 Hz), 22.2, 18.1. **^{19}F NMR** (471 MHz, $CDCl_3$) δ = –114.80 (tt, J = 8.5, 5.3 Hz). **IR** (ATR): 3033, 2961, 2928, 2874, 2731, 1718, 1603, 1551, 1508, 1455, 1433, 1378, 1223, 1160, 1128, 1099, 1080, 1014, 909, 837, 814, 753, 731, 700. **HRMS** (ESI) m/z calcd for $C_{20}H_{22}FNNaO_3^+$ 366.1476 [*M* + Na]$^+$; found: 366.1473.

Diastereomeric ratio:

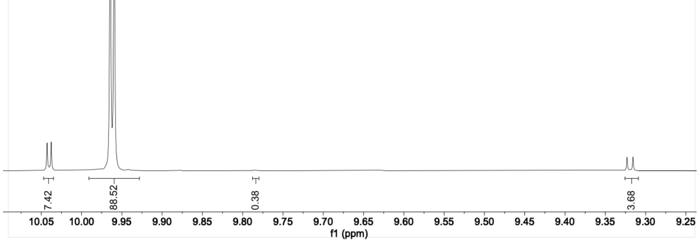

The enantiomeric excess was determined by chiral stationary phase HPLC with an IC column (*n*-hexane/*i*-PrOH 98:2, 40 °C) at 1 mL/min, UV detection at λ = 210 nm: t_R (major) = 32.0 min, t_R (minor) = 28.6 min.

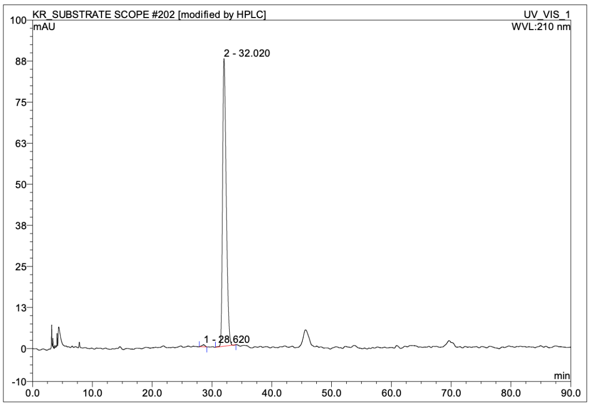

No.	Ret.Time min	Peak Name	Height mAU	Area mAU*min	Rel.Area %	Amount	Type
1	28.62	n.a.	0.645	0.400	0.64	n.a.	BMB*
2	32.02	n.a.	87.513	62.036	99.36	n.a.	BMB*
Total:			88.158	62.436	100.00	0.000	

Isolated product

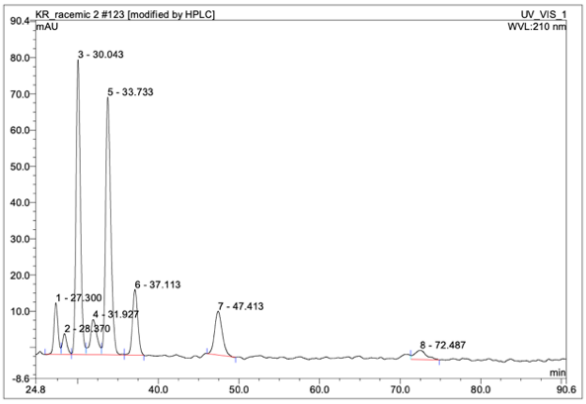

No.	Ret.Time min	Peak Name	Height mAU	Area mAU*min	Rel.Area %	Amount	Type
1	27.30	n.a.	14.211	8.285	5.30	n.a.	BM *
2	28.37	n.a.	5.724	3.632	2.32	n.a.	M *
3	30.04	n.a.	81.420	50.754	32.48	n.a.	M *
4	31.93	n.a.	9.688	9.349	5.98	n.a.	M *
5	33.73	n.a.	71.178	53.000	33.92	n.a.	M *
6	37.11	n.a.	18.067	13.744	8.80	n.a.	MB*
7	47.41	n.a.	12.093	13.210	8.45	n.a.	BMB*
8	72.49	n.a.	2.505	4.288	2.74	n.a.	MB*
Total:			214.885	156.264	100.00	0.000	

Racemic sample

(Using rac. peptide **A***)*

Methyl 4-((3*S*,4*S*,5*R*)-4-formyl-6-nitro-5-phenylhexan-3-yl)benzoate (3oa)

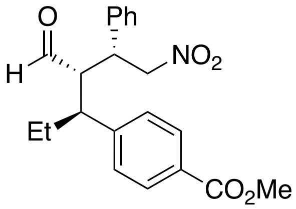

Following general procedure **IV** (10 mol% catalyst), **3oa** was obtained as a light-yellow oil (70% yield, 86:6:5:3 d.r., 99% ee). **^{1}H NMR** (500 MHz, $CDCl_3$) δ = 9.87 (d, *J* = 2.1 Hz, 1H), 8.07 – 8.03 (m, 2H), 7.41 – 7.36 (m, 2H), 7.35 – 7.32 (m, 1H), 7.22 – 7.19 (m, 2H), 7.19 – 7.15 (m, 2H), 4.69 (d, *J* = 1.8 Hz, 1H), 4.68 (s, 1H), 3.95 (s, 3H), 3.80 (td, *J* = 8.2, 6.3 Hz, 1H), 3.19 (ddd, *J* = 8.4, 6.5, 2.1 Hz, 1H), 2.78 (ddd, *J* = 11.6, 6.5, 3.4 Hz, 1H), 1.80 (dqd, *J* = 13.6, 7.4, 3.4 Hz, 1H), 1.68 (ddq, *J* = 14.2, 11.7, 7.2 Hz, 1H), 0.66 (t, *J* = 7.3 Hz, 3H). **^{13}C NMR** (126 MHz, $CDCl_3$) δ = 203.1, 166.2, 145.7, 136.7, 129.8, 128.8, 128.8, 127.8, 127.5, 127.2, 76.7, 59.3, 51.7, 46.2, 42.1, 23.0, 11.6. **IR** (ATR): 2960, 2928, 2875, 2847, 2735, 1712, 1609, 1550, 1454, 1434, 1378, 1311, 1278, 1182, 1111, 1017, 965, 911, 858, 820, 775, 730, 702. **HRMS** (ESI) m/z calcd for $C_{21}H_{23}NNaO_5^+$ 392.1468 [*M* + Na]$^+$; found: 392.1468.

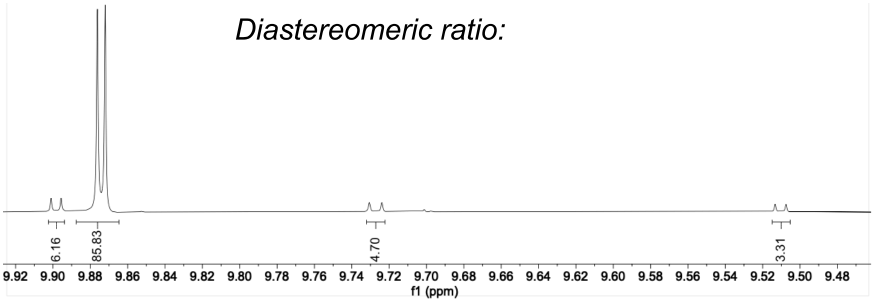

The enantiomeric excess was determined by chiral stationary phase HPLC with an OD-H column (*n*-hexane/*i*-PrOH 95:5, 40 °C) at 1 mL/min, UV detection at λ = 210 nm: t_R (major) = 35.8 min, t_R (minor) = 46.5 min.

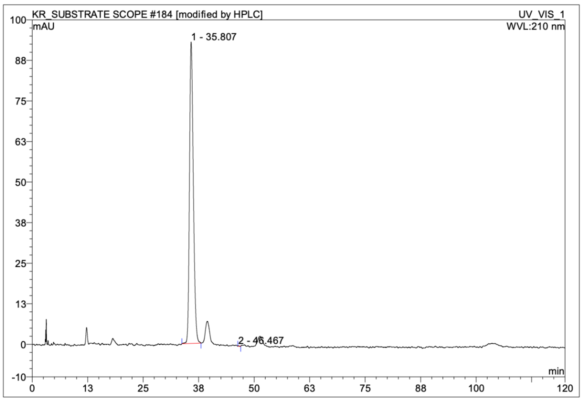

No.	Ret.Time min	Peak Name	Height mAU	Area mAU*min	Rel.Area %	Amount	Type
1	35.81	n.a.	92.876	93.416	99.96	n.a.	BMB*
2	46.47	n.a.	0.160	0.033	0.04	n.a.	BMB*
Total:			93.036	93.449	100.00	0.000	

Isolated product

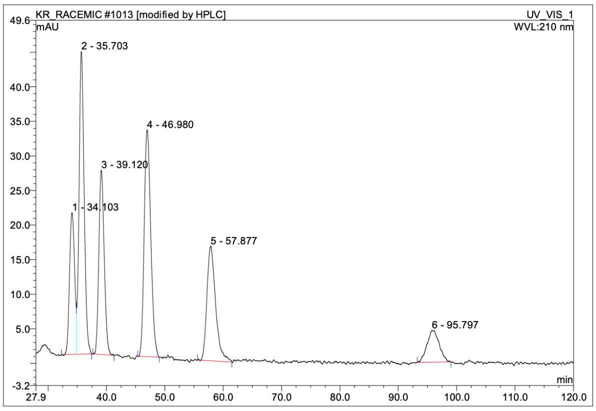

No.	Ret.Time min	Peak Name	Height mAU	Area mAU*min	Rel.Area %	Amount	Type
1	34.10	n.a.	20.496	20.579	11.73	n.a.	BM *
2	35.70	n.a.	43.810	43.978	25.08	n.a.	MB*
3	39.12	n.a.	26.681	28.367	16.17	n.a.	BMB*
4	46.98	n.a.	32.803	42.731	24.37	n.a.	BMB*
5	57.88	n.a.	16.590	28.544	16.28	n.a.	BMB*
6	95.80	n.a.	4.600	11.179	6.37	n.a.	BMB*
Total:			144.980	175.377	100.00	0.000	

Racemic sample

(Using pyrrolidine)

(2*S*,3*R*)-2-(1-(5-Methylfuran-2-yl)ethyl)-4-nitro-3-phenylbutanal (3ja)

Following general procedure **IV 3ja**, was obtained as a colorless oil with a d.r. of 2:1. The two major diastereoisomers **3ja**-diastA and **3ja**-diastB were separated by column chromatography:

(2*R*,3*R*)-2-((*S*)-1-(5-Methylfuran-2-yl)ethyl)-4-nitro-3-phenylbutanal (3ja-diastA**)**

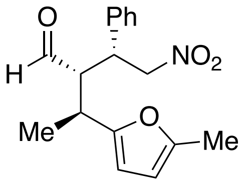

Diastereoisomer 1, **3ja**-diastA: 64% yield, 97:3:<1:<1, 99% ee. **^{1}H NMR** (500 MHz, $CDCl_3$) δ = 9.69 (d, J = 1.2 Hz, 1H), 7.43 – 7.37 (m, 2H), 7.35 – 7.30 (m, 1H), 7.30 – 7.25 (m, 2H), 5.92 (dd, J = 3.2, 1.0 Hz, 1H), 5.89 (dt, J = 3.1, 1.1 Hz, 1H), 4.76 (dd, J = 12.6, 4.2 Hz, 1H), 4.64 (dd, J = 12.6, 10.1 Hz, 1H), 3.89 (td, J = 10.1, 4.2 Hz, 1H), 3.42 (ddd, J = 10.1, 4.7, 1.3 Hz, 1H), 2.94 (qd, J = 7.1, 4.4 Hz, 1H), 2.37 – 2.26 (m, 3H), 1.17 (d, J = 7.2 Hz, 3H). **^{13}C NMR** (126 MHz, $CDCl_3$) δ = 203.6, 154.1, 151.4, 137.0, 129.3, 128.2, 128.0, 106.6, 106.2, 78.7, 56.0, 41.5, 31.8, 13.6, 13.0. **IR** (ATR): 2978, 2920, 2835, 2737, 1718, 1543, 1435, 1378, 1322, 1218, 1199, 1185, 1088, 1023, 953, 940, 842, 788, 756, 700. **HRMS** (ESI) m/z calcd for $C_{17}H_{19}NNaO_4^+$ 324.1206 [M + Na]$^+$; found: 324.1208.

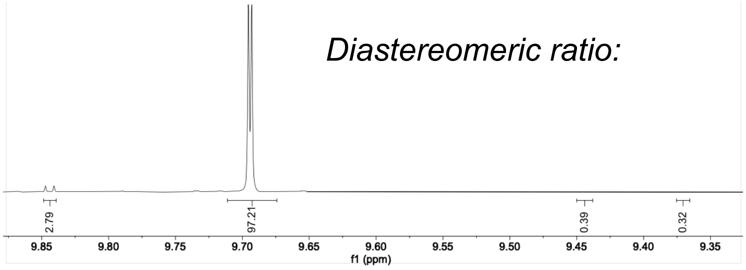

The enantiomeric excess was determined by chiral stationary phase HPLC with an IB N-5 column (*n*-hexane/*i*-PrOH 96:4, 25 °C) at 1 mL/min, UV detection at λ = 210 nm: t_R (major) = 15.5 min, t_R (minor) = 17.1 min.

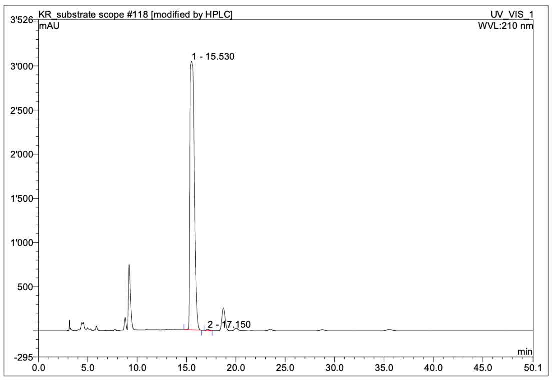

No.	Ret.Time min	Peak Name	Height mAU	Area mAU*min	Rel.Area %	Amount	Type
1	15.53	n.a.	3041.495	1576.466	99.74	n.a.	BMB*
2	17.15	n.a.	12.869	4.166	0.26	n.a.	BMB*
Total:			3054.365	1580.631	100.00	0.000	

Isolated product

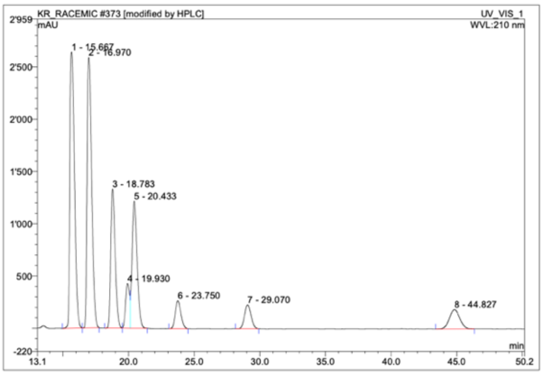

No.	Ret.Time min	Peak Name	Height mAU	Area mAU*min	Rel.Area %	Amount	Type
1	15.67	n.a.	2644.178	1080.841	28.47	n.a.	BM *
2	16.97	n.a.	2588.073	1062.688	27.99	n.a.	MB*
3	18.78	n.a.	1333.426	524.546	13.82	n.a.	BM *
4	19.93	n.a.	427.022	149.511	3.94	n.a.	M *
5	20.43	n.a.	1217.137	546.505	14.39	n.a.	MB*
6	23.75	n.a.	267.301	131.559	3.46	n.a.	BMB*
7	29.07	n.a.	227.954	131.185	3.46	n.a.	BMB*
8	44.83	n.a.	184.731	170.031	4.48	n.a.	BMB*
Total:			8889.823	3796.867	100.00	0.000	

Racemic sample

(Using pyrrolidine)

(2*R*,3*R*)-2-((*R*)-1-(5-Methylfuran-2-yl)ethyl)-4-nitro-3-phenylbutanal (3ja-diastB**)**

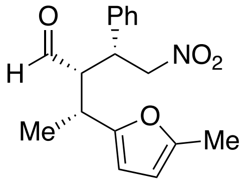

Diastereoisomer 2, **3ja**-diastB: 31% yield, 3:96:1:<1, 99% ee.

^{1}H NMR (500 MHz, $CDCl_3$) δ = 9.84 (d, J = 3.2 Hz, 1H), 7.42 – 7.35 (m, 2H), 7.35 – 7.30 (m, 1H), 7.24 – 7.20 (m, 2H), 5.87 (dq, J = 3.1, 1.0 Hz, 1H), 5.79 (dt, J = 3.1, 0.6 Hz, 1H), 4.65 (s, 1H), 4.63 (d, J = 2.9 Hz, 1H), 3.88 (ddd, J = 10.7, 8.5, 6.0 Hz, 1H), 2.92 (qd, J = 7.3, 3.7 Hz, 1H), 2.86 (dt, J = 10.7, 3.4 Hz, 1H), 2.31 (d, J = 1.0 Hz, 3H), 1.34 (d, J = 7.3 Hz, 3H). **^{13}C NMR** (126 MHz, $CDCl_3$) δ = 203.2, 152.2, 151.6, 137.0, 129.1, 128.3, 128.2, 108.0, 106.0, 79.0, 58.5, 42.5, 32.6, 18.3, 13.6. **IR** (ATR): 2971, 2921, 2877, 2740, 1717, 1550, 1494, 1453, 1377, 1326, 1216, 1184, 1079, 1020, 939, 838, 784, 765, 700. **HRMS** (ESI) m/z calcd for $C_{17}H_{19}NNaO_4^+$ 324.1206 [M + Na]$^+$; found 324.1205.

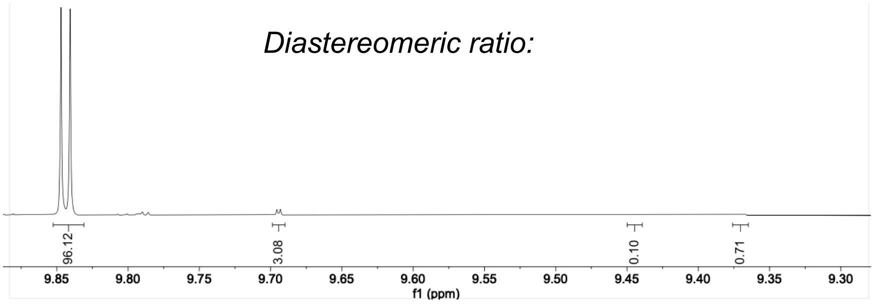

The enantiomeric excess was determined by chiral stationary phase HPLC with an IB N-5 column (*n*-hexane/*i*-PrOH 96:4, 25 °C) at 1 mL/min, UV detection at λ = 210 nm: t_R (major) = 18.4 min, t_R (minor) = 20.3 min.

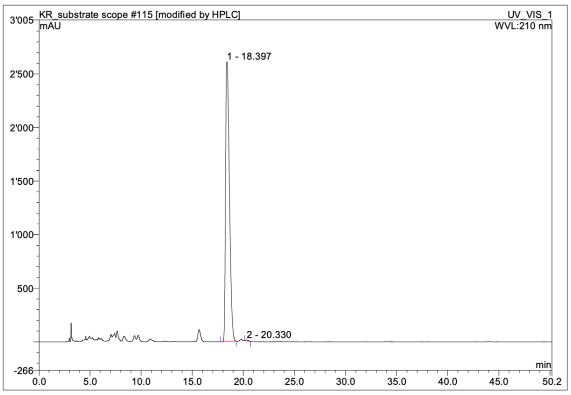

No.	Ret.Time min	Peak Name	Height mAU	Area mAU*min	Rel.Area %	Amount	Type
1	18.40	n.a.	2614.047	1140.319	99.74	n.a.	BMB*
2	20.33	n.a.	9.859	2.978	0.26	n.a.	BMB*
Total:			2623.905	1143.297	100.00	0.000	

Isolated product

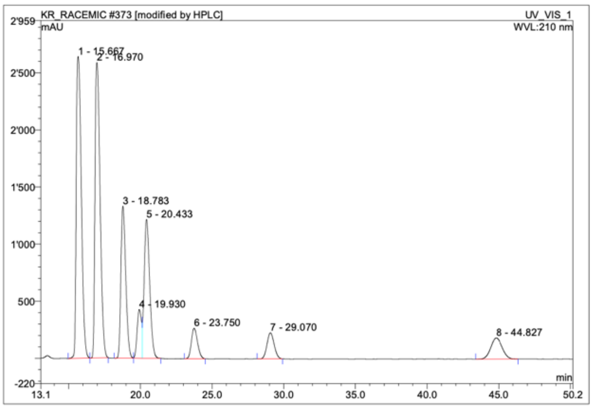

No.	Ret.Time min	Peak Name	Height mAU	Area mAU*min	Rel.Area %	Amount	Type
1	15.67	n.a.	2644.178	1080.841	28.47	n.a.	BM *
2	16.97	n.a.	2588.073	1062.688	27.99	n.a.	MB*
3	18.78	n.a.	1333.426	524.546	13.82	n.a.	BM *
4	19.93	n.a.	427.022	149.511	3.94	n.a.	M *
5	20.43	n.a.	1217.137	546.505	14.39	n.a.	MB*
6	23.75	n.a.	267.301	131.559	3.46	n.a.	BMB*
7	29.07	n.a.	227.954	131.185	3.46	n.a.	BMB*
8	44.83	n.a.	184.731	170.031	4.48	n.a.	BMB*
Total:			8889.823	3796.867	100.00	0.000	

Racemic sample

(Using pyrrolidine)

(2*S*,3*R*)-3,5,5-Trimethyl-2-((*R*)-2-nitro-1-phenylethyl)hexanal (3pa)

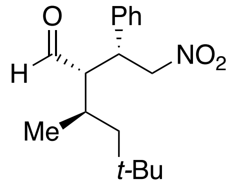

Following general procedure **IV**, **3pa** was obtained as a colorless oil (76% yield, ~2:1 d.r., 99% ee major diast. and 99% ee for minor diast.). **^{1}H NMR** (400 MHz, $CDCl_3$) δ = 9.92 – 9.92 (m, 1H, *maj*+*min*), 7.40 – 7.29 (m, 3H, *maj*+*min*), 7.23–7.20 (m, 2H, *maj*+*min*), 4.78 – 4.70 (m, 1H, *maj*+*min*), 4.61 – 4.51 (m, 1H, *maj*+*min*), 3.94 – 3.82 (m, 1H, *maj*+*min)*, 2.86 – 2.74 (m, 1H, *maj*+*min*), 1.84 – 1.72 (m, 1H, *minor*), 1.62 – 1.51 (m, 1H, *maj*+*min*), 1.40 (dd, *J* = 14.0, 1.7 Hz, 1H, *minor*), 1.22 – 1.19 (m, 1H, *minor*), 0.97 (dd, *J* = 14.0, 10.5 Hz, 1H, *minor*), 0.90 (d, *J* = 7.1 Hz, 2H, *major*), 0.76 (s, 9H, *major*), 0.73 (s, 9H, *minor*). **^{13}C NMR** (101 MHz, $CDCl_3$) *major diast*: δ = 204.5, 136.9, 129.1, 128.2, 128.1, 79.2, 59.8, 50.0, 41.9, 31.6, 29.4, 29.0, 17.3; *minor diast:* δ = 204.1, 137.1, 129.1, 128.3, 128.1, 79.4, 60.3, 45.4, 41.8, 30.9, 30.2, 29.6, 22.0. **IR** (ATR): 2950, 2909, 2865, 2746, 1712, 1543, 1493, 1472, 1430, 1381, 1364, 1234, 1199, 832, 760, 701. **HRMS** (ESI) m/z calcd for $C_{17}H_{25}NNaO_3^+$ 314.1727 [M + Na]$^+$; found: 314.1730.

Diastereomeric ratio

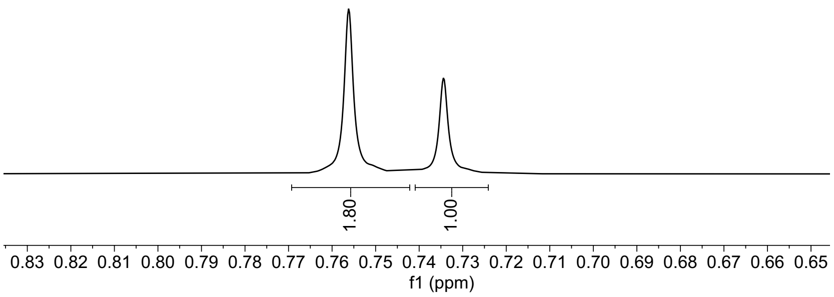

The enantiomeric excess was determined by chiral stationary phase HPLC with an IC column (*n*-hexane/*i*-PrOH 96:4, 25 °C) at 1 mL/min, UV detection at λ = 210 nm: a) *major diast.:* t_R (major enantiomer) = 33.5 min, t_R (minor enantiomer) = 31.1 min; b) *minor diast.:* t_R (major enantiomer) = 23.0 min, t_R (minor enantiomer) = 26.1 min.

Major diastereoisomer:

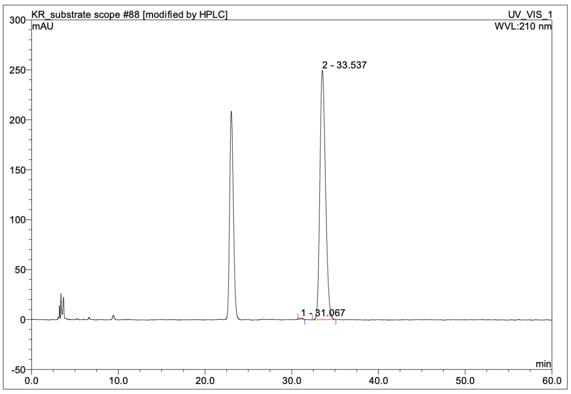

No.	Ret.Time min	Peak Name	Height mAU	Area mAU*min	Rel.Area %	Amount	Type
1	31.07	n.a.	1.027	0.485	0.26	n.a.	BMB*
2	33.54	n.a.	249.822	188.219	99.74	n.a.	BMB*
Total:			250.849	188.704	100.00	0.000	

Minor diastereoisomer:

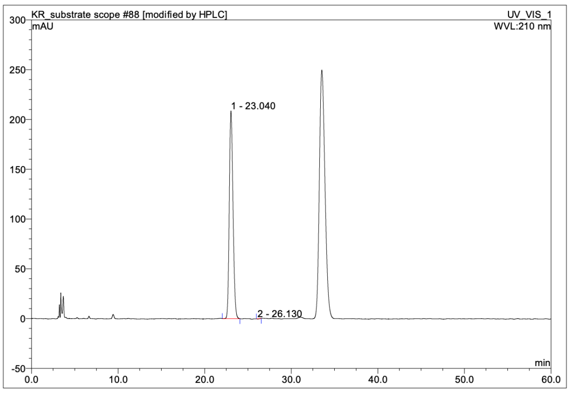

No.	Ret.Time min	Peak Name	Height mAU	Area mAU*min	Rel.Area %	Amount	Type
1	23.04	n.a.	208.560	103.713	99.91	n.a.	BMB*
2	26.13	n.a.	0.216	0.089	0.09	n.a.	BMB*
Total:			208.776	103.802	100.00	0.000	

Racemic sample (Using pyrrolidine):

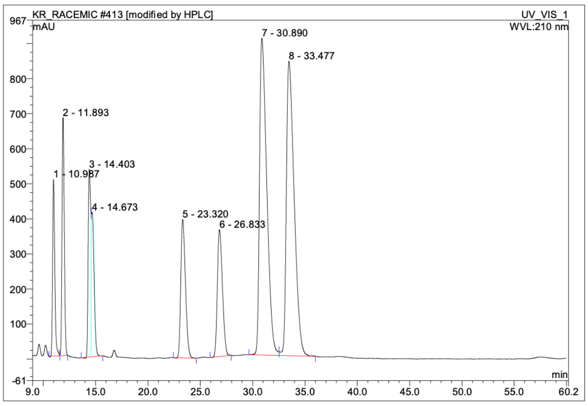

No.	Ret.Time min	Peak Name	Height mAU	Area mAU*min	Rel.Area %	Amount	Type
1	10.99	n.a.	504.141	122.209	5.03	n.a.	BM *
2	11.89	n.a.	678.665	168.867	6.96	n.a.	MB*
3	14.40	n.a.	535.375	150.536	6.20	n.a.	BM *
4	14.67	n.a.	412.100	129.171	5.32	n.a.	MB*
5	23.32	n.a.	395.220	203.016	8.36	n.a.	BMB*
6	26.83	n.a.	361.806	203.363	8.38	n.a.	BMB*
7	30.89	n.a.	903.644	723.724	29.82	n.a.	BM *
8	33.48	n.a.	840.178	726.311	29.92	n.a.	MB*
Total:			4631.129	2427.197	100.00	0.000	

(2*S*,3*R*)-7-Hydroxy-3,7-dimethyl-2-((*R*)-2-nitro-1-phenylethyl)octanal (3qa)

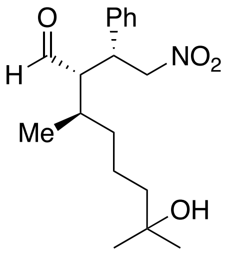

Following general procedure **IV**, **3qa** was obtained as a colorless oil (99% yield, 1.3:1 d.r., 99% ee major diast. and 99% ee for minor diast.). **^{1}H NMR** (400 MHz, $CDCl_3$) δ = 9.92 (d, *J* = 2.4 Hz, 1H, *maj*), 9.91 (d, *J* = 2.2 Hz, 1H, *min*), 7.40 – 7.29 (m, 3H, *maj+min*), 7.22 – 7.18 (m, 2H, *maj+min*), 4.74 – 4.66 (m, 1H, *maj+min*), 4.65 – 4.62 (m, 1H, *maj+min*), 4.02 – 3.88 (m, 1H, *maj+min*), 2.97 – 2.85 (m, 1H, *maj*), 2.80 (ddd, *J* = 10.5, 3.8, 2.2 Hz, 1H, *min*), 1.64 – 1.21 (m, 7H, *maj+min*), 1.19 (s, 3H, *maj+min*), 1.15 (d, *J* = 3.1 Hz, 3H, *maj+min*), 1.11 (d, *J* = 7.1 Hz, 3H, *min*), 0.90 (d, *J* = 7.0 Hz, 3H, *maj*). **^{13}C NMR** (101 MHz, $CDCl_3$) *Major diast*: δ = 204.2, 136.9, 129.2, 128.2, 128.01, 79.3, 70.8, 56.6, 43.40, 41.7, 36.1, 32.9, 29.3, 29.1, 22.0, 14.9. *Minor diast*: δ = 204.0, 137.1, 129.1, 128.2, 128.03, 79.0, 70.7, 59.0, 43.39, 41.6, 32.6, 32.0, 29.4, 29.1, 22.1, 18.6. **IR** (ATR): 2966, 2935, 2870, 2736, 1715, 1551, 1495, 1456, 1432, 1375, 1242, 1209, 1154, 1044, 936, 909, 845, 761, 737, 701. **HRMS** (ESI) m/z calcd for $C_{18}H_{27}NNaO_4^+$ 344.1832 [*M* + Na]$^+$; found: 344.1834.

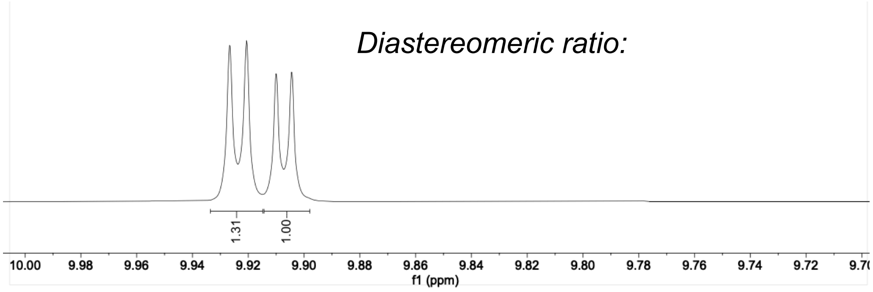

The enantiomeric excess was determined by chiral stationary phase HPLC with an IC column (*n*-hexane/*i*-PrOH 90:10, 40 °C) at 1 mL/min, UV detection at λ = 210 nm:
major diast.: t_R (major enantiomer) = 35.3 min, t_R (minor enantiomer) = 48.6 min;
minor diast.: t_R (major enantiomer) = 51.1 min, t_R (minor enantiomer) = 44.0 min.

Major diastereoisomer:

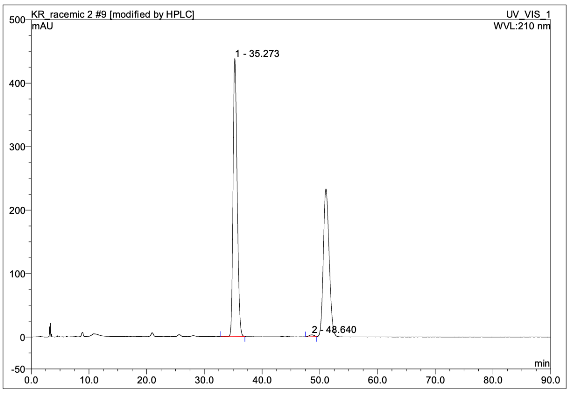

No.	Ret.Time min	Peak Name	Height mAU	Area mAU*min	Rel.Area %	Amount	Type
1	35.27	n.a.	437.800	356.972	99.20	n.a.	BMB*
2	48.64	n.a.	2.909	2.869	0.80	n.a.	BMB*
Total:			440.709	359.840	100.00	0.000	

Minor diastereoisomer:

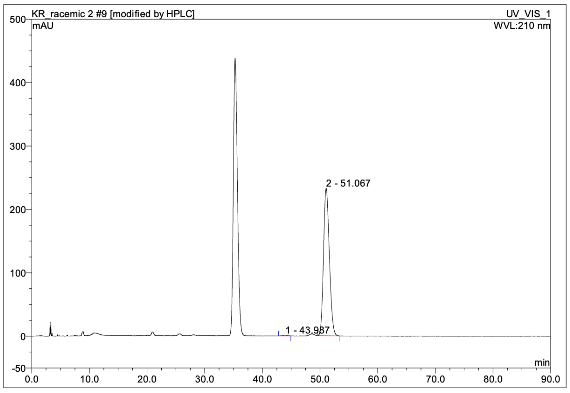

No.	Ret.Time min	Peak Name	Height mAU	Area mAU*min	Rel.Area %	Amount	Type
1	43.99	n.a.	1.013	0.905	0.33	n.a.	BMB*
2	51.07	n.a.	232.717	272.161	99.67	n.a.	BMB*
Total:			233.730	273.067	100.00	0.000	

Racemic sample (Using rac. peptide **A***):*

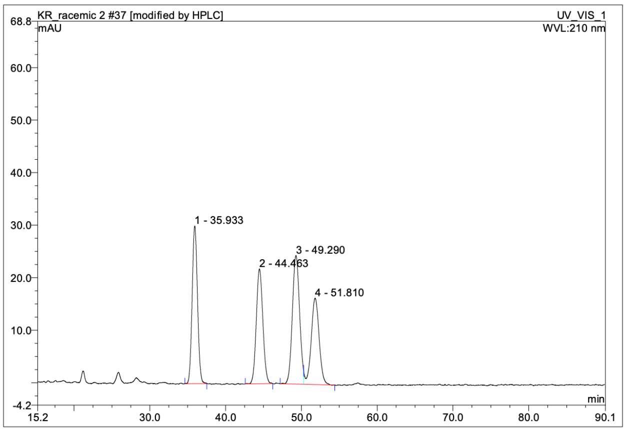

No.	Ret.Time min	Peak Name	Height mAU	Area mAU*min	Rel.Area %	Amount	Type
1	35.93	n.a.	30.025	24.433	26.11	n.a.	BMB*
2	44.46	n.a.	21.847	21.969	23.48	n.a.	BMB*
3	49.29	n.a.	24.483	26.989	28.84	n.a.	BM *
4	51.81	n.a.	16.483	20.183	21.57	n.a.	MB*
Total:			92.838	93.573	100.00	0.000	

(2*S*,3*R*)-3-(4-Fluorophenyl)-4-nitro-2-((*S*)-1-phenylethyl)butanal (3ab)

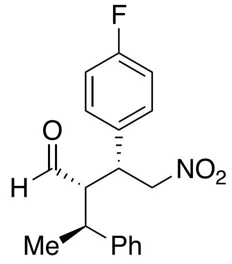

Following general procedure **IV**, **3ab** was obtained as a colorless oil (94% yield, 93:5:<1:2 d.r., 99% ee). **^{1}H NMR** (500 MHz, $CDCl_3$) δ = 9.73 (d, *J* = 1.6 Hz, 1H), 7.39 – 7.34 (m, 2H), 7.32 – 7.26 (m, 1H), 7.25 – 7.21 (m, 2H), 7.19 – 7.16 (m, 2H), 7.13 – 7.07 (m, 2H), 4.73 (dd, *J* = 12.7, 4.2 Hz, 1H), 4.60 (dd, *J* = 12.7, 10.2 Hz, 1H), 3.85 (td, *J* = 9.8, 4.2 Hz, 1H), 3.22 (ddd, *J* = 9.4, 5.7, 1.6 Hz, 1H), 2.99 – 2.91 (m, 1H), 1.27 (d, *J* = 7.2 Hz, 3H). **^{13}C NMR** (126 MHz, $CDCl_3$) δ = 203.8, 162.4 (d, *J* = 247.6 Hz), 142.7, 133.0 (d, *J* = 3.4 Hz), 129.5, 129.5 (d, *J* = 8.1 Hz), 129.0, 127.3, 127.1, 116.3 (d, *J* = 21.6 Hz), 78.1, 59.7, 41.6, 38.2, 15.0. **^{19}F NMR** (471 MHz, $CDCl_3$) δ = –113.43 (tt, *J* = 8.4, 5.1 Hz). **IR** (ATR): 3024, 2970, 2919, 2856, 2749, 1716, 1600, 1546, 1507, 1494, 1450, 1378, 1219, 1158, 1107, 1031, 968, 849, 767, 778, 699. **HRMS** (ESI) m/z calcd for $C_{18}H_{18}FNNaO_3^+$ 338.1163 [*M* + Na]$^+$; found: 338.1164.

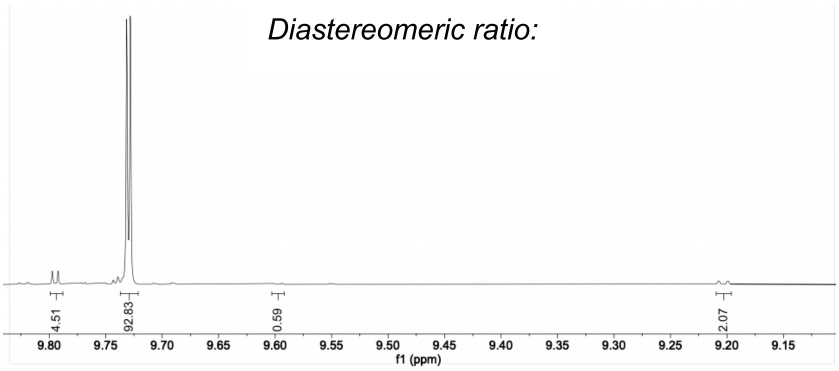

The enantiomeric excess was determined by chiral stationary phase HPLC with an IC column (*n*-hexane/*i*-PrOH 95:5, 25 °C) at 1 mL/min, UV detection at λ = 210 nm: t_R (major) = 28.7 min, t_R (minor) = 22.3 min.

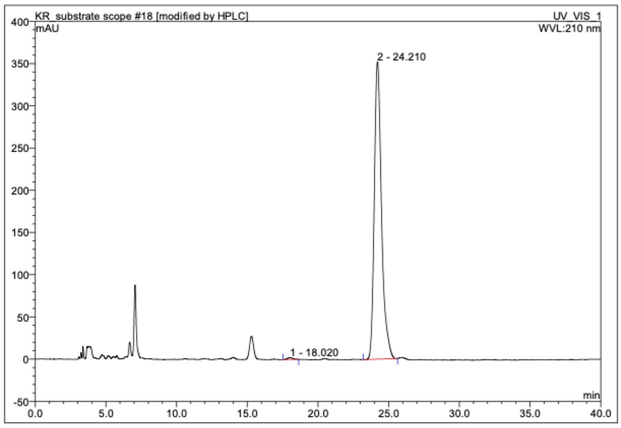

No.	Ret.Time min	Peak Name	Height mAU	Area mAU*min	Rel.Area %	Amount	Type
1	18.02	n.a.	2.076	0.977	0.47	n.a.	BMB*
2	24.21	n.a.	352.292	208.091	99.53	n.a.	BMB*
Total:			354.368	209.068	100.00	0.000	

Isolated product

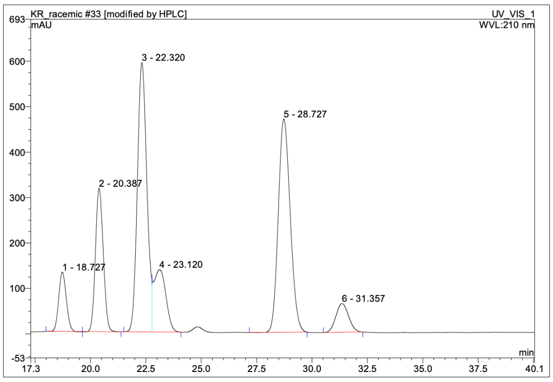

No.	Ret.Time min	Peak Name	Height mAU	Area mAU*min	Rel.Area %	Amount	Type
1	18.73	n.a.	130.873	51.895	5.76	n.a.	BM *
2	20.39	n.a.	315.503	129.868	14.42	n.a.	MB*
3	22.32	n.a.	592.821	293.929	32.64	n.a.	BM *
4	23.12	n.a.	137.283	88.510	9.83	n.a.	MB*
5	28.73	n.a.	469.449	294.037	32.66	n.a.	BMB*
6	31.36	n.a.	63.346	42.168	4.68	n.a.	BMB*
Total:			1709.275	900.407	100.00	0.000	

Racemic sample

(Using pyrrolidine)

(2*S*,3*R*)-3-(4-Chlorophenyl)-4-nitro-2-((*S*)-1-phenylethyl)butanal (3ac)

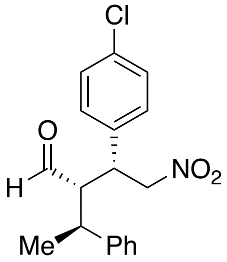

Following general procedure **IV**, **3ac** was obtained as a colorless oil (99% yield, 93:5:<1:2 d.r., 99% ee). **^{1}H NMR** (500 MHz, $CDCl_3$) δ = 9.72 (d, J = 1.6 Hz, 1H), 7.46 – 7.33 (m, 4H), 7.31 – 7.25 (m, 1H), 7.21 – 7.13 (m, 4H), 4.73 (dd, J = 12.8, 4.2 Hz, 1H), 4.61 (dd, J = 12.8, 10.2 Hz, 1H), 3.83 (td, J = 9.8, 4.2 Hz, 1H), 3.21 (ddd, J = 9.3, 5.7, 1.6 Hz, 1H), 2.99 – 2.90 (m, 1H), 1.26 (d, J = 7.2 Hz, 3H). **^{13}C NMR** (126 MHz, $CDCl_3$) δ = 203.7, 142.6, 135.8, 134.1, 129.5, 129.2, 129.0, 127.3, 127.1, 77.9, 59.5, 41.7, 38.2, 15.1. **IR** (ATR): 3066, 3024, 2969, 2916, 2855, 2747, 1716, 1546, 1491, 1448, 1377, 1200, 1091, 1014, 849, 819, 776. **HRMS** (ESI) m/z calcd. for $C_{18}H_{18}ClNNaO_3^+$ 354.0867 [M + Na]$^+$; found: 354.0866.

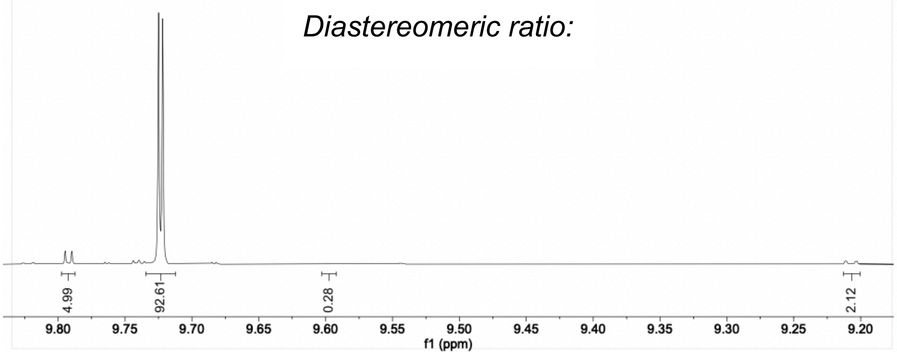

The enantiomeric excess was determined by chiral stationary phase HPLC with an IB-N5 column (*n*-hexane/*i*-PrOH 95:5, 25 °C) at 1 mL/min, UV detection at λ = 210 nm: t_R (major) = 49.5 min, t_R (minor) = 29.0 min.

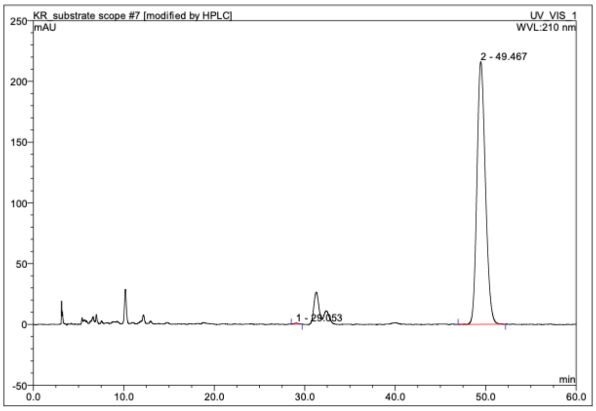

No.	Ret.Time min	Peak Name	Height mAU	Area mAU*min	Rel.Area %	Amount	Type
1	29.05	n.a.	0.791	0.454	0.19	n.a.	BMB*
2	49.47	n.a.	216.170	234.600	99.81	n.a.	BMB*
Total:			216.961	235.055	100.00	0.000	

Isolated product

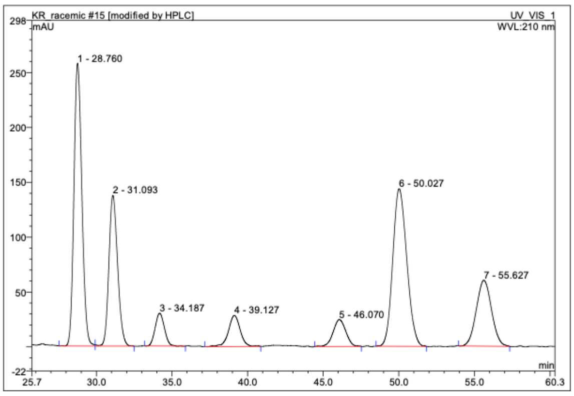

No.	Ret.Time min	Peak Name	Height mAU	Area mAU*min	Rel.Area %	Amount	Type
1	28.76	n.a.	257.447	155.896	28.80	n.a.	BM *
2	31.09	n.a.	137.411	91.679	16.94	n.a.	MB*
3	34.19	n.a.	29.876	21.030	3.89	n.a.	BMB*
4	39.13	n.a.	28.311	25.369	4.69	n.a.	BMB*
5	46.07	n.a.	24.458	23.828	4.40	n.a.	BMB*
6	50.03	n.a.	143.541	153.692	28.39	n.a.	BMB*
7	55.63	n.a.	60.011	69.794	12.89	n.a.	BMB*
Total:			681.054	541.288	100.00	0.000	

Racemic sample

(Using pyrrolidine)

(2*S*,3*R*)-3-(3-Chlorophenyl)-4-nitro-2-((*S*)-1-phenylethyl)butanal (3ad)

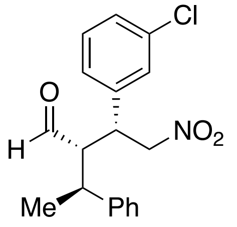

Following general procedure **IV**, **3ad** was obtained as a colorless oil (99% yield, 94:4:<1:2 d.r., 90% ee). ^{1}**H NMR** (500 MHz, $CDCl_3$) δ = 9.73 (d, *J* = 1.6 Hz, 1H), 7.42 – 7.27 (m, 5H), 7.23 (td, *J* = 1.9, 0.6 Hz, 1H), 7.20 – 7.17 (m, 2H), 7.16 – 7.12 (m, 1H), 4.72 (dd, *J* = 13.0, 4.2 Hz, 1H), 4.63 (dd, *J* = 13.0, 10.1 Hz, 1H), 3.82 (ddd, *J* = 10.1, 9.1, 4.2 Hz, 1H), 3.23 (ddd, *J* = 9.2, 5.9, 1.6 Hz, 1H), 3.04 – 2.93 (m, 1H), 1.28 (d, *J* = 7.1 Hz, 3H). ^{13}**C NMR** (126 MHz, $CDCl_3$) δ = 203.6, 142.5, 139.5, 135.2, 130.6, 129.0, 128.5, 128.2, 127.4, 127.1, 125.9, 77.6, 59.5, 42.0, 38.3, 15.3. **IR** (ATR): 3024, 2971, 2918, 2850, 2744, 1717, 1546, 1492, 1435, 1379, 1273, 1234, 1080, 853, 779, 699. **HRMS** (ESI) m/z calcd for $C_{18}H_{18}ClNNaO_3^+$ 354.0867 [*M* + Na]$^+$; found: 354.0867.

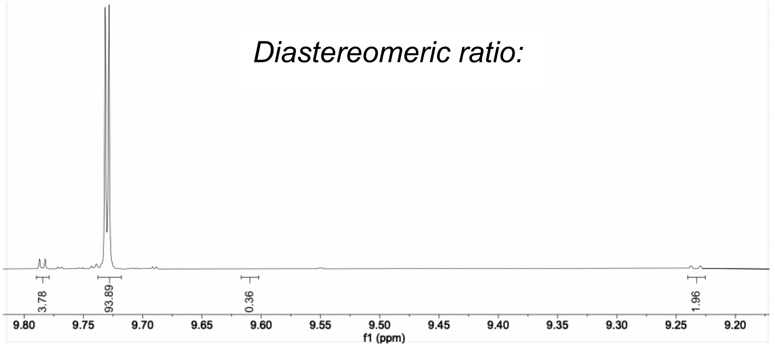

The enantiomeric excess was determined by chiral stationary phase HPLC with an IB-N5 column (*n*-hexane/*i*-PrOH 96:4, 25 °C) at 1 mL/min, UV detection at λ = 210 nm: t_R (major) = 39.1 min, t_R (minor) = 31.1 min.

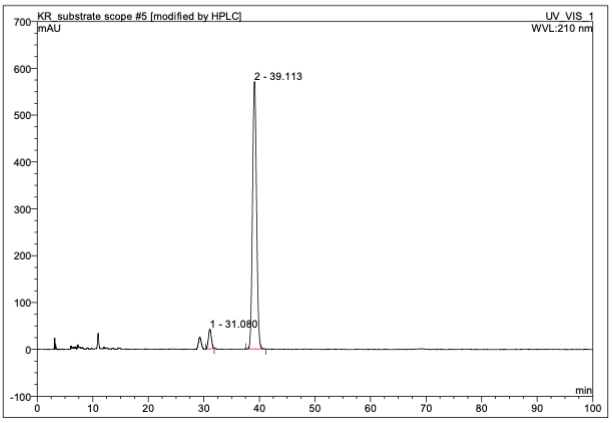

No.	Ret.Time min	Peak Name	Height mAU	Area mAU*min	Rel.Area %	Amount	Type
1	31.08	n.a.	41.433	25.673	5.08	n.a.	BMB*
2	39.11	n.a.	570.662	479.886	94.92	n.a.	BMB*
Total:			612.094	505.559	100.00	0.000	

Isolated product

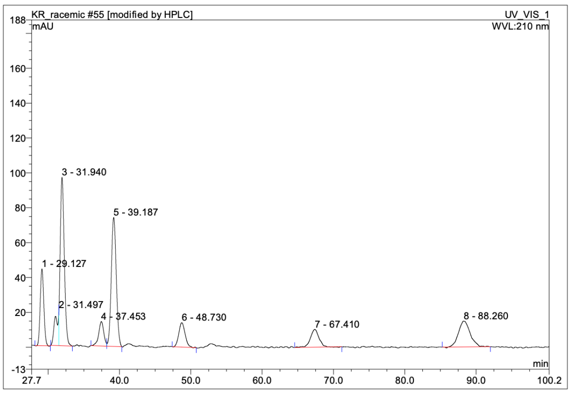

No.	Ret.Time min	Peak Name	Height mAU	Area mAU*min	Rel.Area %	Amount	Type
1	29.13	n.a.	44.050	25.608	11.15	n.a.	BMB*
2	31.50	n.a.	20.451	11.606	5.05	n.a.	BM *
3	31.94	n.a.	96.571	64.351	28.02	n.a.	MB*
4	37.45	n.a.	13.976	11.583	5.04	n.a.	BM *
5	39.19	n.a.	73.958	59.941	26.10	n.a.	MB*
6	48.73	n.a.	13.986	14.359	6.25	n.a.	BMB*
7	67.41	n.a.	10.224	14.209	6.19	n.a.	BMB*
8	88.26	n.a.	14.769	28.003	12.19	n.a.	BMB*
Total:			287.985	229.660	100.00	0.000	

Racemic sample

(Using pyrrolidine)

(2*S*,3*R*)-3-(2-Chlorophenyl)-4-nitro-2-((*S*)-1-phenylethyl)butanal (3ae)

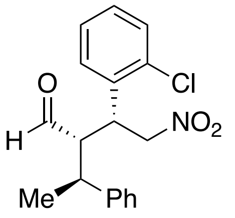

Following general procedure **IV**, **3ae** was obtained as a colorless oil (91% yield, 95:<1:4:1 d.r., 99% ee). **^{1}H NMR** (500 MHz, $CDCl_3$) δ = 9.72 (d, *J* = 1.5 Hz, 1H), 7.48 – 7.44 (m, 1H), 7.40 – 7.35 (m, 2H), 7.33 – 7.24 (m, 6H), 4.91 (dd, *J* = 12.9, 9.6 Hz, 1H), 4.71 (dd, *J* = 12.9, 4.1 Hz, 1H), 4.33 (td, *J* = 9.4, 4.1 Hz, 1H), 3.62 – 3.51 (m, 1H), 2.98 (p, *J* = 6.9 Hz, 1H), 1.28 (d, *J* = 7.2 Hz, 3H). **^{13}C NMR** (126 MHz, $CDCl_3$) δ = 203.9, 142.6, 134.4, 134.2, 130.9, 129.4, 128.9, 128.9, 127.5, 127.3, 127.3, 76.2, 57.7, 38.5, 15.5. **IR** (ATR): 3029, 2964, 2920, 2834, 2735, 1716, 1546, 1493, 1428, 1379, 1284, 1200, 1129, 1039, 966, 917, 846, 765, 699. **HRMS** (ESI) m/z calcd for $C_{18}H_{18}ClNNaO_3^+$ 354.0867 [*M* + Na]$^+$; found: 354.0867.

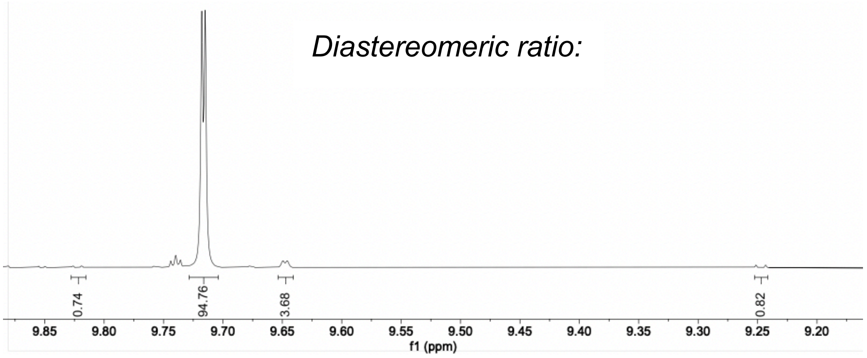

The enantiomeric excess was determined by chiral stationary phase HPLC with an IB N-5 column (*n*-hexane/*i*-PrOH 97:3, 25 °C) at 1 mL/min, UV detection at λ = 210 nm: t_R (major) = 30.2 min, t_R (minor) = 25.8 min.

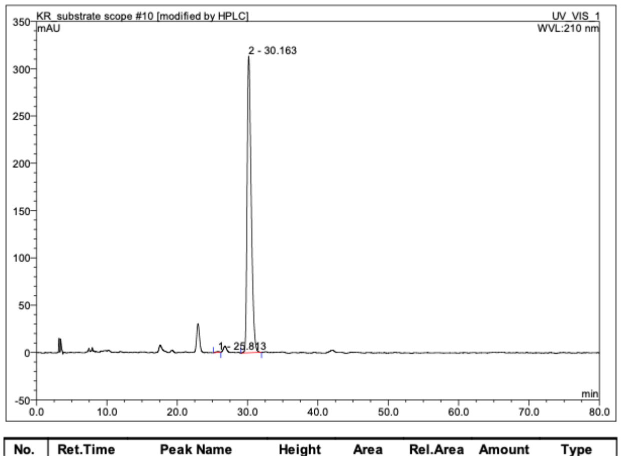

No.	Ret.Time min	Peak Name	Height mAU	Area mAU*min	Rel.Area %	Amount	Type
1	25.81	n.a.	0.866	0.509	0.24	n.a.	BMB*
2	30.16	n.a.	313.557	211.087	99.76	n.a.	BMB*
Total:			314.423	211.596	100.00	0.000	

Isolated product

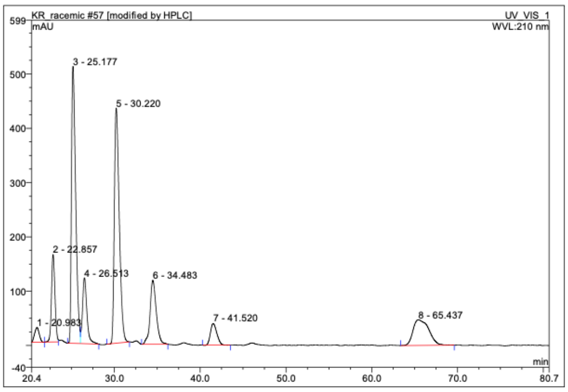

No.	Ret.Time min	Peak Name	Height mAU	Area mAU*min	Rel.Area %	Amount	Type
1	20.98	n.a.	27.098	16.756	1.72	n.a.	BMb*
2	22.86	n.a.	161.142	75.367	7.73	n.a.	bM *
3	25.18	n.a.	509.925	289.947	29.73	n.a.	BM *
4	26.51	n.a.	121.360	72.516	7.44	n.a.	MB*
5	30.22	n.a.	432.648	287.771	29.51	n.a.	BMB*
6	34.48	n.a.	117.671	98.466	10.10	n.a.	BMB*
7	41.52	n.a.	39.749	33.605	3.45	n.a.	BMB*
8	65.44	n.a.	47.120	100.686	10.33	n.a.	BMB*
Total:			1456.713	975.113	100.00	0.000	

Racemic sample

(Using pyrrolidine)

(2*S*,3*R*)-4-Nitro-3-(4-nitrophenyl)-2-((*S*)-1-phenylethyl)butanal (3af)

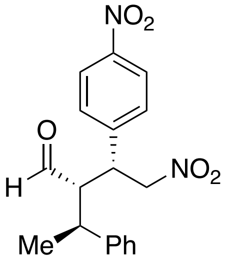

Following general procedure **IV**, **3af** was obtained as a yellow oil (99% yield, 95:3:<1:2 d.r., 99% ee). **^{1}H NMR** (500 MHz, $CDCl_3$) δ = 9.75 (d, J = 1.5 Hz, 1H), 8.30 – 8.23 (m, 2H), 7.46 – 7.41 (m, 2H), 7.41 – 7.36 (m, 2H), 7.33 – 7.30 (m, 1H), 7.19 – 7.16 (m, 2H), 4.81 – 4.67 (m, 2H), 3.95 (ddd, J = 9.9, 8.7, 4.5 Hz, 1H), 3.27 (ddd, J = 8.7, 6.2, 1.5 Hz, 1H), 2.94 (p, J = 6.9 Hz, 1H), 1.30 (d, J = 7.1 Hz, 3H). **^{13}C NMR** (126 MHz, $CDCl_3$) δ = 203.0, 147.7, 145.1, 142.1, 129.2, 129.0, 127.6, 127.0, 124.5, 77.2, 59.5, 42.1, 38.7, 15.7. **IR** (ATR): 2971, 2918, 2852, 2745, 1714, 1552, 1519, 1448, 1377, 1345, 1182, 1087, 1046, 857, 759, 697. **HRMS** (ESI) m/z calcd for $C_{18}H_{18}N_2NaO_5^+$ 365.1108 [M + Na]$^+$; found: 365.1105.

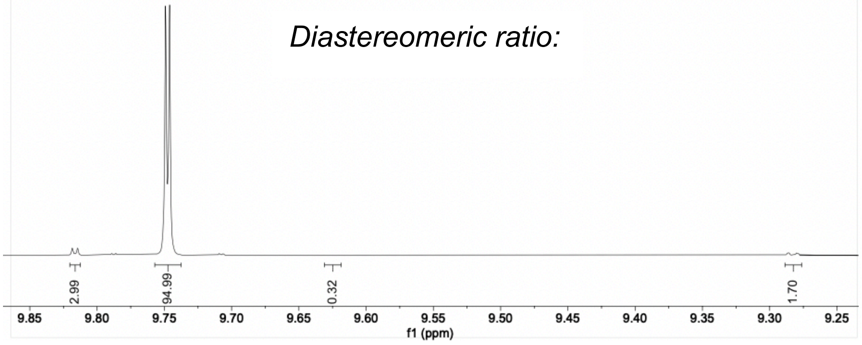

The enantiomeric excess was determined by chiral stationary phase HPLC with an AD-H column (n-hexane/i-PrOH 95:5, 25 °C) at 1 mL/min, UV detection at λ = 210 nm: t_R (major) = 63.2 min, t_R (minor) = 50.3 min.

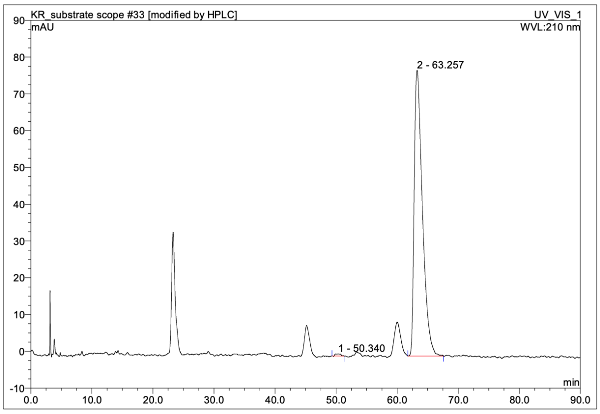

No.	Ret.Time min	Peak Name	Height mAU	Area mAU*min	Rel.Area %	Amount	Type
1	50.34	n.a.	0.580	0.600	0.49	n.a.	BMB*
2	63.26	n.a.	77.709	121.094	99.51	n.a.	BMB*
Total:			78.288	121.694	100.00	0.000	

Isolated product

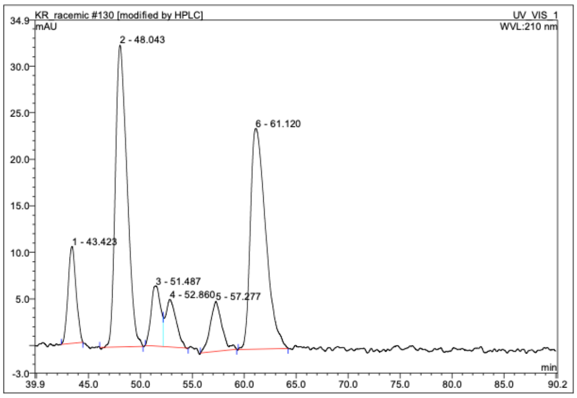

No.	Ret.Time min	Peak Name	Height mAU	Area mAU*min	Rel.Area %	Amount	Type
1	43.42	n.a.	10.409	9.328	8.36	n.a.	BMB*
2	48.04	n.a.	32.414	41.981	37.60	n.a.	BMB*
3	51.49	n.a.	6.468	6.843	6.13	n.a.	BM *
4	52.86	n.a.	5.117	6.151	5.51	n.a.	MB*
5	57.28	n.a.	5.353	6.942	6.22	n.a.	BMB*
6	61.12	n.a.	23.700	40.394	36.18	n.a.	BMB*
Total:			83.460	111.639	100.00	0.000	

Racemic sample

(Using pyrrolidine)

(2*S*,3*R*)-4-Nitro-3-(3-nitrophenyl)-2-((*S*)-1-phenylethyl)butanal (3ag)

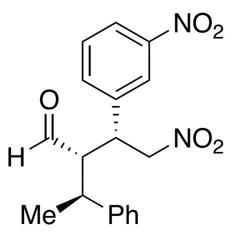

Following general procedure **IV**, **3ag** was obtained as a yellow oil (96% yield, 94:3:<1:3 d.r., 99% ee). ^{1}**H NMR** (500 MHz, $CDCl_3$) δ = 9.75 (d, *J* = 1.5 Hz, 1H), 8.23 – 8.17 (m, 1H), 8.12 (qd, *J* = 1.2, 0.5 Hz, 1H), 7.62 – 7.57 (m, 2H), 7.41 – 7.34 (m, 2H), 7.32 – 7.26 (m, 1H), 7.17 (ddt, *J* = 7.8, 1.2, 0.6 Hz, 2H), 4.84 – 4.63 (m, 2H), 3.97 (ddd, *J* = 10.1, 8.9, 4.4 Hz, 1H), 3.29 (ddd, *J* = 8.9, 6.3, 1.6 Hz, 1H), 2.95 (p, *J* = 7.0 Hz, 1H), 1.30 (d, *J* = 7.1 Hz, 3H). ^{13}**C NMR** (126 MHz, $CDCl_3$) δ = 203.0, 148.7, 142.1, 139.8, 134.2, 130.3, 129.2, 127.5, 127.1, 123.3, 122.8, 77.4, 59.4, 42.0, 38.7, 15.8. **IR** (ATR): 3025, 2971, 2918, 2852, 2745, 1714, 1537, 1526, 1441, 1347, 1101, 901, 807, 759, 736. **HRMS** (ESI) m/z calcd for $C_{18}H_{18}N_2NaO_5^+$ 365.1108 [*M* + Na]$^+$; found 365.1108.

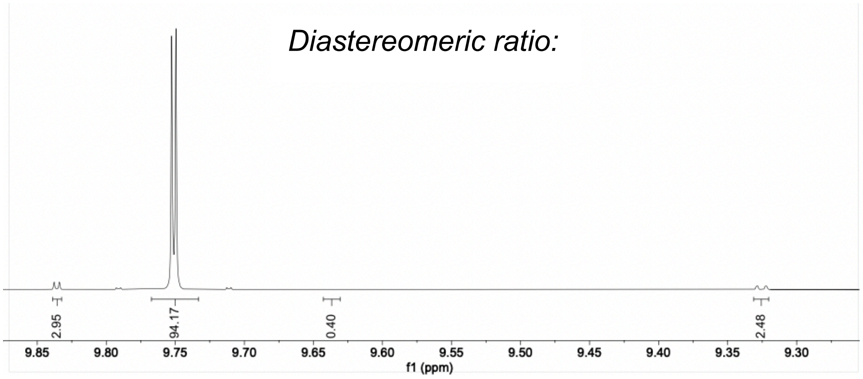

The enantiomeric excess was determined by chiral stationary phase HPLC with an IB N-5 column (*n*-hexane/*i*-PrOH 95:5, 25 °C) at 1 mL/min, UV detection at λ = 210 nm: t_R (major) = 89.6 min, t_R (minor) = 78.2 min.

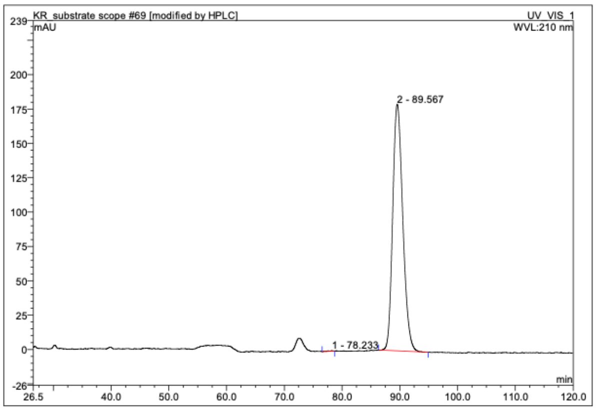

No.	Ret.Time min	Peak Name	Height mAU	Area mAU*min	Rel.Area %	Amount	Type
1	78.23	n.a.	0.357	0.390	0.11	n.a.	BMB*
2	89.57	n.a.	179.522	363.528	99.89	n.a.	BMB*
Total:			179.879	363.919	100.00	0.000	

Isolated product

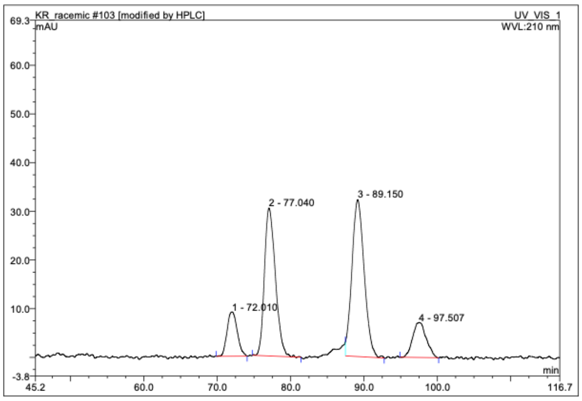

No.	Ret.Time min	Peak Name	Height mAU	Area mAU*min	Rel.Area %	Amount	Type
1	72.01	n.a.	9.110	14.708	10.11	n.a.	BMB*
2	77.04	n.a.	30.391	52.690	36.23	n.a.	BMB*
3	89.15	n.a.	32.234	62.618	43.05	n.a.	MB*
4	97.51	n.a.	7.201	15.422	10.60	n.a.	BMB*
Total:			78.935	145.438	100.00	0.000	

Racemic sample

(Using pyrrolidine)

(2*S*,3*R*)-4-Nitro-3-(2-nitrophenyl)-2-((*S*)-1-phenylethyl)butanal (3ah)

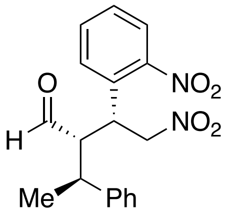

Following general procedure **IV**, **3ah** was obtained as a yellow oil (94% yield, 97:2:<1:1 d.r., 99% ee). ^{1}**H NMR** (500 MHz, $CDCl_3$) δ = 9.78 (d, *J* = 1.7 Hz, 1H), 7.90 (dd, *J* = 8.1, 1.4 Hz, 1H), 7.63 (td, *J* = 7.7, 1.4 Hz, 1H), 7.49 (ddd, *J* = 8.1, 7.4, 1.4 Hz, 1H), 7.44 (dd, *J* = 7.9, 1.4 Hz, 1H), 7.38 – 7.32 (m, 2H), 7.31 – 7.24 (m, 1H), 7.21 – 7.17 (m, 2H), 4.94 (dd, *J* = 13.5, 8.7 Hz, 1H), 4.76 (dd, *J* = 13.4, 4.0 Hz, 1H), 4.38 (td, *J* = 8.8, 4.0 Hz, 1H), 3.58 (ddd, *J* = 8.8, 6.2, 1.7 Hz, 1H), 3.01 (p, *J* = 6.9 Hz, 1H), 1.27 (d, *J* = 7.1 Hz, 3H). ^{13}**C NMR** (126 MHz, $CDCl_3$) δ = 203.5, 150.3, 142.1, 133.2, 131.7, 129.1, 129.1, 129.0, 127.4, 127.2, 125.5, 76.9, 58.0, 39.2, 26.9, 16.4. **IR** (ATR): 2968, 2924, 2849, 2739, 1716, 1531, 1523, 1449, 1347, 1200, 855, 755, 697. **HRMS** (ESI) m/z calcd for $C_{18}H_{18}N_2NaO_5^+$ 365.1108 [*M* + Na]$^+$; found: 365.1108.

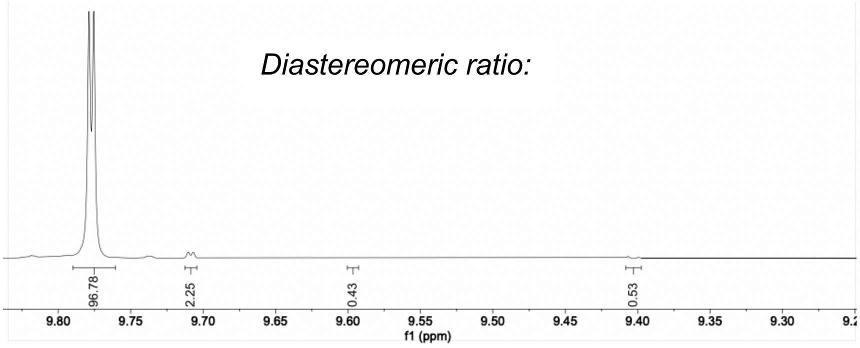

The enantiomeric excess was determined by chiral stationary phase HPLC with an IB N-5 column (*n*-hexane/*i*-PrOH 95:5, 25 °C) at 1 mL/min, UV detection at λ = 210 nm: t_R (major) = 53.0 min, t_R (minor) = 42.1 min.

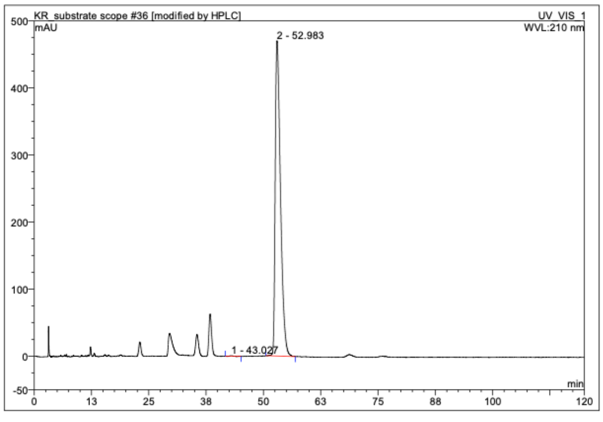

No.	Ret.Time min	Peak Name	Height mAU	Area mAU*min	Rel.Area %	Amount	Type
1	43.03	n.a.	0.799	0.279	0.04	n.a.	BMB*
2	52.98	n.a.	470.009	628.463	99.96	n.a.	BMB*
Total:			470.808	628.742	100.00	0.000	

Isolated product

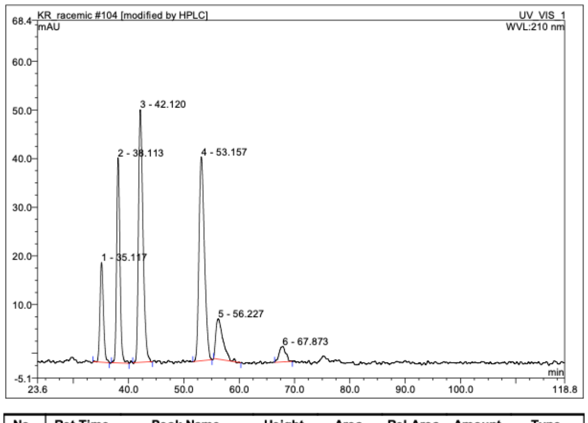

No.	Ret.Time min	Peak Name	Height mAU	Area mAU*min	Rel.Area %	Amount	Type
1	35.12	n.a.	20.475	15.039	9.52	n.a.	BMB*
2	38.11	n.a.	42.125	29.132	18.43	n.a.	BMB*
3	42.12	n.a.	51.913	48.939	30.97	n.a.	BMB*
4	53.16	n.a.	41.880	48.066	30.42	n.a.	BMB*
5	56.23	n.a.	8.329	12.452	7.88	n.a.	BMB*
6	67.87	n.a.	3.175	4.401	2.79	n.a.	BMB*
Total:			167.896	158.029	100.00	0.000	

Racemic sample

(*Using pyrrolidine*)

(2*S*,3*R*)-3-(4-Bromophenyl)-4-nitro-2-((*S*)-1-phenylethyl)butanal (3ai)

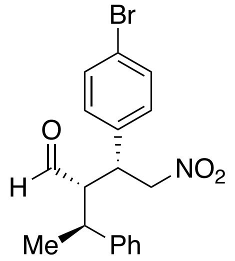

Following general procedure **IV**, **3ai** was obtained as a yellow oil (95% yield, 95:3:<1:2 d.r., 99% ee). **^{1}H NMR** (500 MHz, $CDCl_3$) δ = 9.72 (d, J = 1.6 Hz, 1H), 7.56 – 7.52 (m, 2H), 7.40 – 7.34 (m, 2H), 7.32 – 7.27 (m, 1H), 7.19 – 7.16 (m, 2H), 7.15 – 7.10 (m, 2H), 4.72 (dd, J = 12.8, 4.2 Hz, 1H), 4.61 (dd, J = 12.8, 10.2 Hz, 1H), 3.82 (td, J = 9.8, 4.2 Hz, 1H), 3.21 (ddd, J = 9.3, 5.7, 1.6 Hz, 1H), 2.99 – 2.90 (m, 1H), 1.26 (d, J = 7.1 Hz, 3H). **^{13}C NMR** (126 MHz, $CDCl_3$) δ = 203.6, 142.6, 136.4, 132.5, 129.6, 129.0, 127.3, 127.1, 122.2, 77.8, 59.4, 41.8, 38.2, 15.1. **IR** (ATR): 3025, 2969, 2913, 2853, 2746, 1714, 1546, 1489, 1439, 1377, 1236, 1200, 1071, 1009, 849, 816, 775. **HRMS** (ESI) m/z calcd for $C_{18}H_{18}BrNNaO_3^+$ 398.0362 [M + Na]$^+$; found: 398.0362.

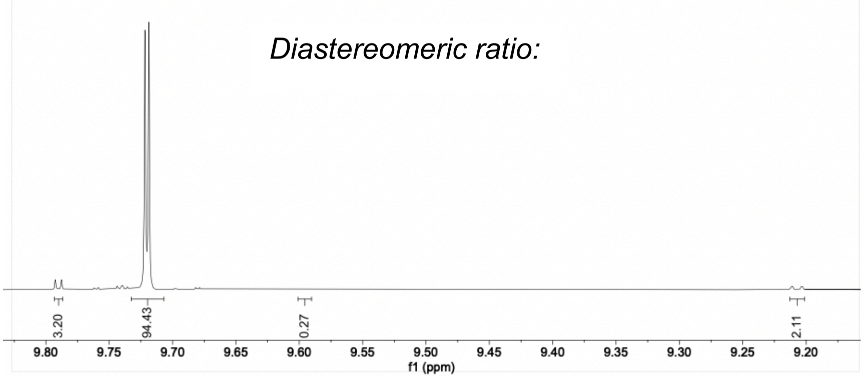

The enantiomeric excess was determined by chiral stationary phase HPLC with an IB N-5 column (*n*-hexane/*i*-PrOH 95:5, 25 °C) at 1 mL/min, UV detection at λ = 210 nm: t_R (major) = 53.5 min, t_R (minor) = 32.0 min.

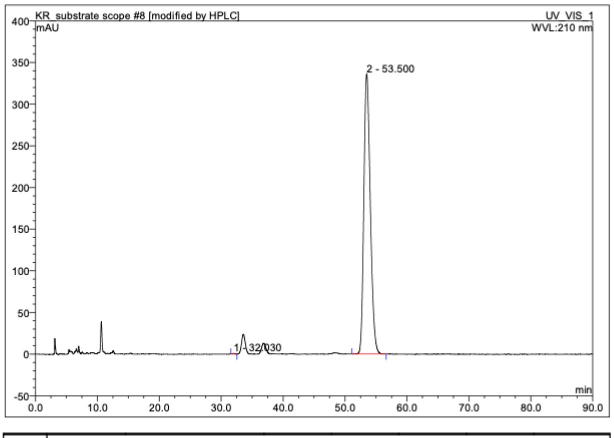

No.	Ret.Time min	Peak Name	Height mAU	Area mAU*min	Rel.Area %	Amount	Type
1	32.03	n.a.	0.644	0.269	0.07	n.a.	BMB*
2	53.50	n.a.	336.232	406.218	99.93	n.a.	BMB*
Total:			336.876	406.486	100.00	0.000	

Isolated product

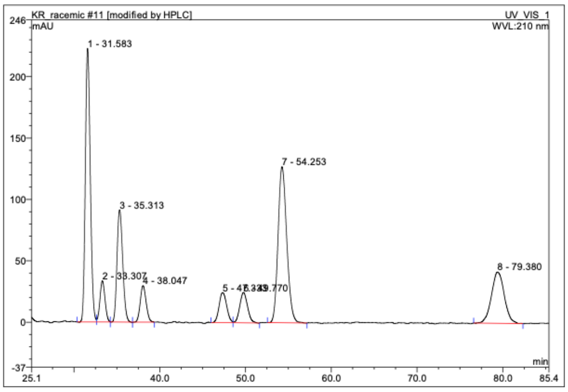

No.	Ret.Time min	Peak Name	Height mAU	Area mAU*min	Rel.Area %	Amount	Type
1	31.58	n.a.	222.728	148.592	27.63	n.a.	BM *
2	33.31	n.a.	33.562	22.913	4.26	n.a.	M *
3	35.31	n.a.	91.389	70.324	13.07	n.a.	M *
4	38.05	n.a.	29.641	23.608	4.39	n.a.	MB*
5	47.33	n.a.	24.549	25.070	4.66	n.a.	BM *
6	49.77	n.a.	24.671	25.845	4.81	n.a.	MB*
7	54.25	n.a.	127.073	149.296	27.76	n.a.	BMB*
8	79.38	n.a.	41.735	72.213	13.43	n.a.	BMB*
Total:			595.347	537.862	100.00	0.000	

Racemic sample

(Using pyrrolidine)

(2*S*,3*R*)-3-(4-Methoxyphenyl)-4-nitro-2-((*S*)-1-phenylethyl)butanal (3aj)

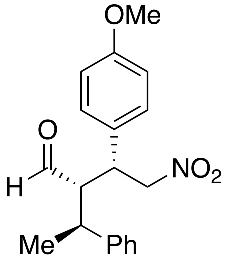

Following general procedure **IV**, **3aj** was obtained as a colorless oil (63% yield, 92:4:2:2 d.r., 92% ee). **^{1}H NMR** (500 MHz, $CDCl_3$) δ = 9.73 (d, J = 1.7 Hz, 1H), 7.39 – 7.33 (m, 2H), 7.28 (m, 1H), 7.21 – 7.16 (m, 4H), 6.95 – 6.92 (m, 2H), 4.71 (dd, J = 12.5, 4.3 Hz, 1H), 4.57 (dd, J = 12.5, 10.2 Hz, 1H), 3.83 (s, 3H), 3.84 – 3.79 (m, 1H), 3.21 (ddd, J = 9.7, 5.4, 1.7 Hz, 1H), 2.98 (qd, J = 7.1, 5.2 Hz, 1H), 1.25 (d, J = 7.2 Hz, 3H). **^{13}C NMR** (126 MHz, $CDCl_3$) δ = 204.2, 159.3, 143.0, 128.9, 128.9, 127.2, 127.1, 114.7, 78.5, 59.5, 55.3, 41.6, 39.5, 14.7. **IR** (ATR): 2965, 2926, 2837, 2736, 1715, 1610, 1549, 1512, 1452, 1378, 1297, 1250, 1179, 1117, 1031, 830, 764. **HRMS** (ESI) m/z calcd. for $C_{19}H_{21}NNaO_4^+$ 350.1363 [M + Na]$^+$; found: 350.1361.

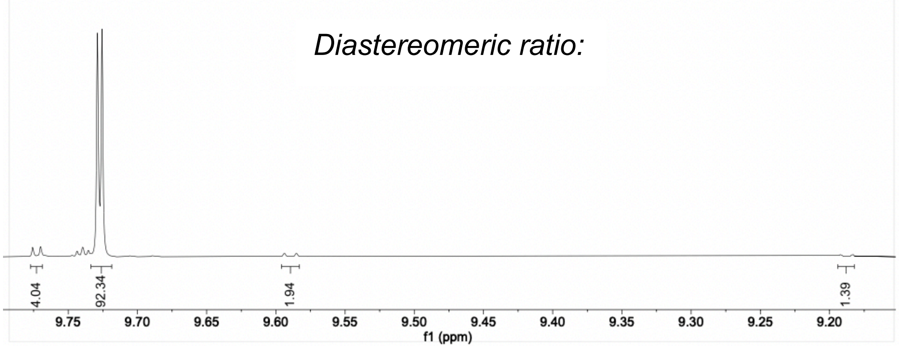

The enantiomeric excess was determined by chiral stationary phase HPLC with an IB-N5 column (n-hexane/i-PrOH 97:3, 25 °C) at 1 mL/min, UV detection at λ = 210 nm: t_R (major) = 53.7 min, t_R (minor) = 39.2 min.

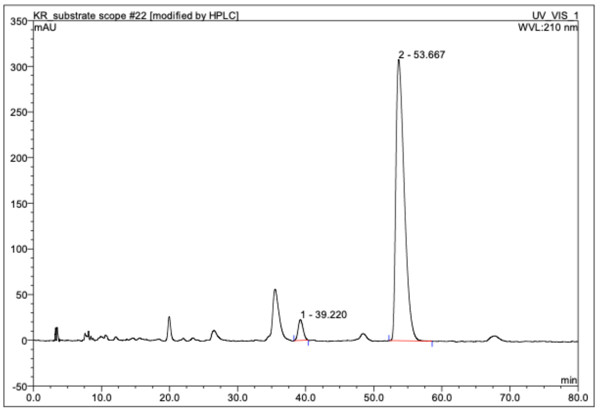

No.	Ret.Time min	Peak Name	Height mAU	Area mAU*min	Rel.Area %	Amount	Type
1	39.22	n.a.	22.879	19.301	4.19	n.a.	BMB*
2	53.67	n.a.	307.979	441.530	95.81	n.a.	BMB*
Total:			330.858	460.831	100.00	0.000	

Isolated product

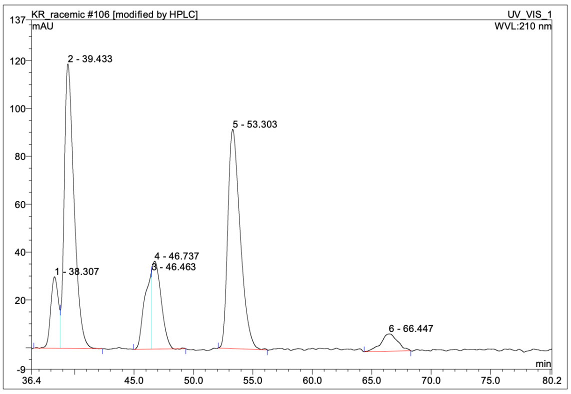

No.	Ret.Time min	Peak Name	Height mAU	Area mAU*min	Rel.Area %	Amount	Type
1	38.31	n.a.	29.888	22.530	7.17	n.a.	BM *
2	39.43	n.a.	118.849	114.018	36.30	n.a.	MB*
3	46.46	n.a.	32.314	21.642	6.89	n.a.	BM *
4	46.74	n.a.	36.727	33.351	10.62	n.a.	MB*
5	53.30	n.a.	91.601	110.452	35.17	n.a.	BMB*
6	66.45	n.a.	7.267	12.083	3.85	n.a.	BMB*
Total:			316.644	314.076	100.00	0.000	

Racemic sample

(Using pyrrolidine)

(2*S*,3*R*)-3-(3-Methoxyphenyl)-4-nitro-2-((*S*)-1-phenylethyl)butanal (3ak)

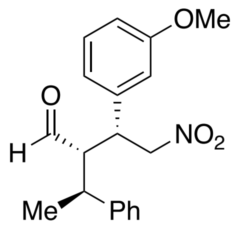

Following general procedure **IV**, **3ak** was obtained as a colorless oil (99% yield, 95:4:<1:1 d.r., 96% ee). **^{1}H NMR** (500 MHz, $CDCl_3$) δ = 9.73 (d, *J* = 1.6 Hz, 1H), 7.40 – 7.31 (m, 3H), 7.30 – 7.26 (m, 1H), 7.21 – 7.16 (m, 2H), 6.86 (dddd, *J* = 13.7, 7.6, 2.2, 0.9 Hz, 2H), 6.80 – 6.77 (m, 1H), 4.72 (dd, *J* = 12.7, 4.3 Hz, 1H), 4.62 (dd, *J* = 12.7, 10.1 Hz, 1H), 3.85 – 3.79 (m, 1H), 3.84 (s, 3H), 3.24 (ddd, *J* = 9.5, 5.6, 1.7 Hz, 1H), 3.00 (qd, *J* = 7.1, 5.5 Hz, 1H), 1.27 (d, *J* = 7.1 Hz, 3H). **^{13}C NMR** (126 MHz, $CDCl_3$) δ = 204.0, 160.1, 142.9, 138.9, 130.4, 128.9, 128.9, 127.2, 119.9, 114.3, 113.0, 78.5, 59.6, 55.3, 42.3, 38.0, 14.7. **IR** (ATR): 2924, 2837, 2737, 1715, 1600, 1550, 1490, 1452, 1378, 1320, 1260, 1156, 1042, 874, 779. **HRMS** (ESI) m/z calcd for $C_{19}H_{21}NNaO_4^+$ 350.1363 $[M + Na]^+$; found: 350.1362.

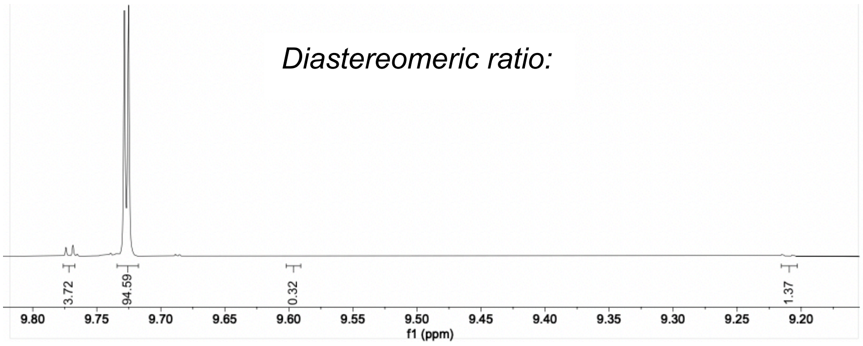

The enantiomeric excess was determined by chiral stationary phase HPLC with an AD-H column (*n*-hexane/*i*-PrOH 97:3, 25 °C) at 1 mL/min, UV detection at λ = 210 nm: t_R (major) = 21.9 min, t_R (minor) = 18.4 min.

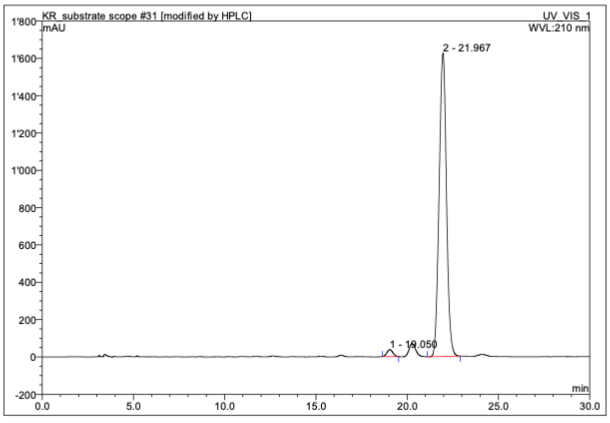

No.	Ret.Time min	Peak Name	Height mAU	Area mAU*min	Rel.Area %	Amount	Type
1	19.05	n.a.	37.344	14.380	1.87	n.a.	BMB*
2	21.97	n.a.	1626.020	754.937	98.13	n.a.	BMB*
Total:			1663.364	769.317	100.00	0.000	

Isolated product

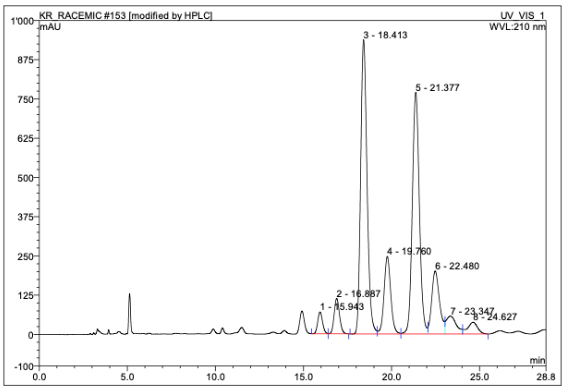

No.	Ret.Time min	Peak Name	Height mAU	Area mAU*min	Rel.Area %	Amount	Type
1	15.94	n.a.	69.849	22.321	2.06	n.a.	BM *
2	16.89	n.a.	112.922	39.855	3.67	n.a.	MB*
3	18.41	n.a.	936.913	392.612	36.15	n.a.	BM *
4	19.76	n.a.	247.102	108.433	9.99	n.a.	M *
5	21.38	n.a.	769.883	357.467	32.92	n.a.	M *
6	22.48	n.a.	200.655	103.184	9.50	n.a.	M *
7	23.35	n.a.	56.849	37.377	3.44	n.a.	M *
8	24.63	n.a.	37.462	24.683	2.27	n.a.	MB*
Total:			2431.635	1085.931	100.00	0.000	

Racemic sample

(Using pyrrolidine)

(2*S*,3*R*)-3-(2-Methoxyphenyl)-4-nitro-2-((*S*)-1-phenylethyl)butanal (3al)

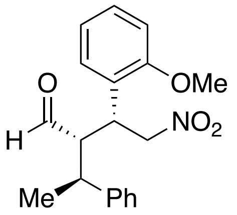

Following general procedure **IV** (48 h), **3al** was obtained as a colorless oil (72% yield, 92:5:<1:3 d.r., 78% ee). **^{1}H NMR** (500 MHz, $CDCl_3$) δ = 9.68 (d, J = 1.5 Hz, 1H), 7.40 – 7.23 (m, 5H), 7.23 – 7.14 (m, 2H), 7.00 – 6.93 (m, 2H), 4.84 (dd, J = 12.4, 10.1 Hz, 1H), 4.65 (dd, J = 12.4, 4.2 Hz, 1H), 4.07 (td, J = 9.9, 4.2 Hz, 1H), 3.95 (s, 2H), 3.53 (ddd, J = 9.7, 5.3, 1.6 Hz, 1H), 2.92 (qd, J = 7.1, 5.1 Hz, 1H), 1.21 (d, J = 7.2 Hz, 3H). **^{13}C NMR** (126 MHz, $CDCl_3$) δ = 204.3, 157.4, 143.4, 130.9, 129.5, 128.7, 127.3, 126.9, 124.5, 121.2, 111.2, 76.7, 57.6, 55.3, 39.8, 38.2, 14.6. **IR** (ATR): 2960, 2926, 2831, 2736, 1713, 1599, 1545, 1490, 1455, 1379, 1284, 1243, 1199, 1120, 1080, 1024, 968, 915, 763, 700. **HRMS** (ESI) m/z calcd for $C_{19}H_{21}NNaO_4^+$ 350.1363 [*M* + Na]$^+$; found: 350.1363.

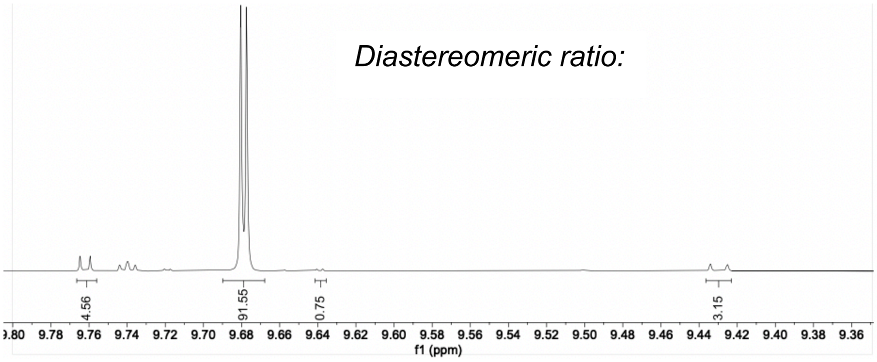

The enantiomeric excess was determined by chiral stationary phase HPLC with an IB N-5 column (*n*-hexane/*i*-PrOH 90:10, 25 °C) at 1 mL/min, UV detection at λ = 210 nm: t_R (major) = 30.4 min, t_R (minor) = 28.5 min.

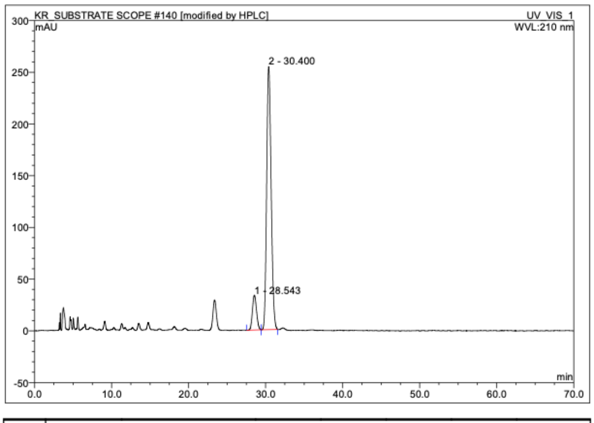

No.	Ret.Time min	Peak Name	Height mAU	Area mAU*min	Rel.Area %	Amount	Type
1	28.54	n.a.	33.675	20.827	10.80	n.a.	BMB*
2	30.40	n.a.	254.396	171.932	89.20	n.a.	BMB*
Total:			288.071	192.759	100.00	0.000	

Isolated product

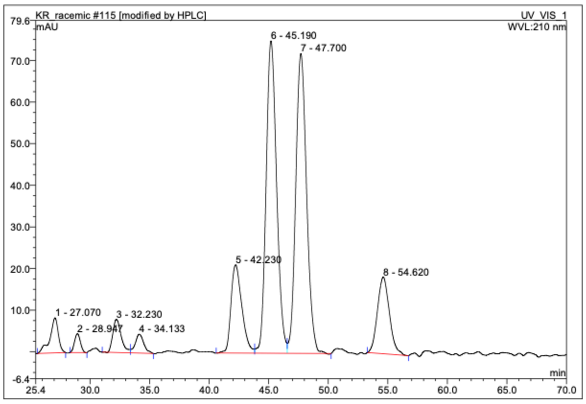

No.	Ret.Time min	Peak Name	Height mAU	Area mAU*min	Rel.Area %	Amount	Type
1	27.07	n.a.	8.446	6.937	3.20	n.a.	BMB*
2	28.95	n.a.	4.526	2.575	1.19	n.a.	BMB*
3	32.23	n.a.	7.985	6.569	3.03	n.a.	BM *
4	34.13	n.a.	4.544	3.825	1.76	n.a.	MB*
5	42.23	n.a.	21.238	22.620	10.44	n.a.	BM *
6	45.19	n.a.	75.069	76.631	35.35	n.a.	M *
7	47.70	n.a.	72.075	76.219	35.16	n.a.	MB*
8	54.62	n.a.	18.452	21.388	9.87	n.a.	BMB*
Total:			212.336	216.764	100.00	0.000	

Racemic sample

(Using pyrrolidine)

(2*S*,3*R*)-4-Nitro-2-((*S*)-1-phenylethyl)-3-(*p*-tolyl)butanal (3am)

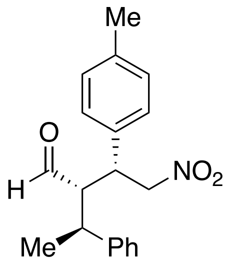

Following general procedure **IV**, **3am** was obtained as a colorless oil (71% yield, 94:5:<1:1 d.r., 99% ee). **^{1}H NMR** (400 MHz, $CDCl_3$) δ = 9.72 (d, *J* = 1.7 Hz, 1H), 7.42 – 7.32 (m, 2H), 7.31 – 7.25 (m, 1H), 7.24 – 7.10 (m, 6H), 4.72 (dd, *J* = 12.5, 4.3 Hz, 1H), 4.60 (dd, *J* = 12.5, 10.1 Hz, 1H), 3.82 (td, *J* = 9.9, 4.3 Hz, 1H), 3.23 (ddd, *J* = 9.6, 5.5, 1.7 Hz, 1H), 3.02 – 2.93 (m, 1H), 2.37 (s, 3H), 1.25 (d, *J* = 7.2 Hz, 3H). **^{13}C NMR** (101 MHz, $CDCl_3$) δ = 204.2, 143.0, 138.0, 134.0, 130.0, 128.9, 127.7, 127.2, 127.1, 78.3, 59.6, 41.9, 38.0, 21.1, 14.7. **IR** (ATR): 3025, 2969, 2917, 2851, 2744, 1716, 1544, 1513, 1432, 1378, 1308, 1237, 1200, 1117, 1077, 1044, 847, 807, 764, 697. **HRMS** (ESI) m/z calcd for $C_{19}H_{21}NNaO_3^+$ 334.1414 [*M* + Na]$^+$; found: 334.1411.

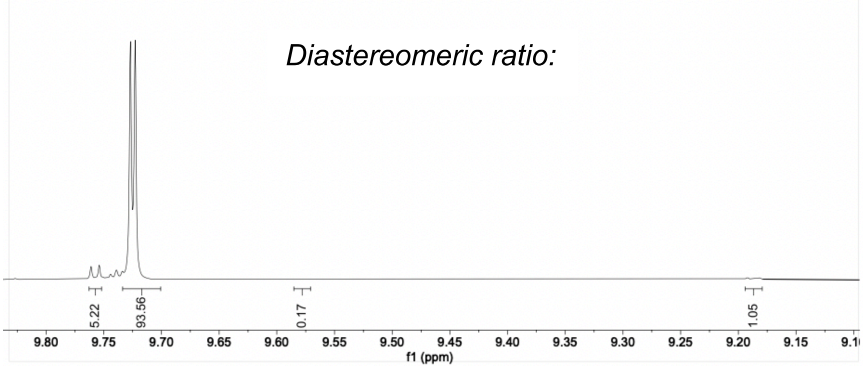

The enantiomeric excess was determined by chiral stationary phase HPLC with an IB N-5 column (*n*-hexane/*i*-PrOH 96:4, 25 °C) at 1 mL/min, UV detection at λ = 210 nm: t_R (major) = 28.1 min, t_R (minor) = 20.8 min.

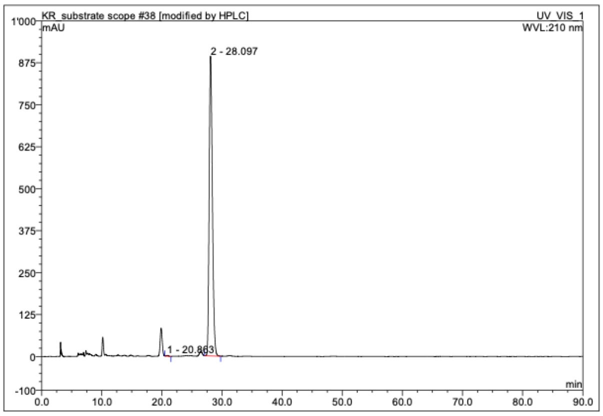

No.	Ret.Time min	Peak Name	Height mAU	Area mAU*min	Rel.Area %	Amount	Type
1	20.86	n.a.	2.070	0.697	0.13	n.a.	BMB*
2	28.10	n.a.	892.787	555.762	99.87	n.a.	BMB*
Total:			894.858	556.459	100.00	0.000	

Isolated product

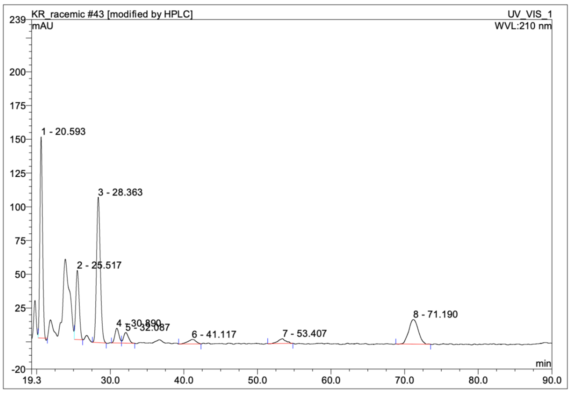

No.	Ret.Time min	Peak Name	Height mAU	Area mAU*min	Rel.Area %	Amount	Type
1	20.59	n.a.	149.007	61.104	30.36	n.a.	M *
2	25.52	n.a.	51.273	27.134	13.48	n.a.	MB*
3	28.36	n.a.	107.740	64.299	31.94	n.a.	BMB*
4	30.89	n.a.	10.933	6.962	3.46	n.a.	BM *
5	32.09	n.a.	7.850	6.035	3.00	n.a.	MB*
6	41.12	n.a.	3.359	4.359	2.17	n.a.	BMB*
7	53.41	n.a.	3.454	4.073	2.02	n.a.	BMB*
8	71.19	n.a.	18.199	27.329	13.58	n.a.	BMB*
Total:			351.816	201.295	100.00	0.000	

Racemic sample

(Using pyrrolidine)

(2*S*,3*S*)-3-(Furan-2-yl)-4-nitro-2-((*S*)-1-phenylethyl)butanal (3an)

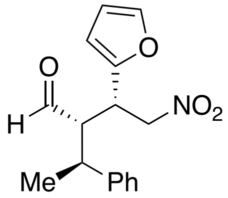

Following general procedure **IV**, **3an** was obtained as a colorless oil (94% yield, 95:4:<1:1 d.r., 99% ee). **^{1}H NMR** (400 MHz, $CDCl_3$) δ = 9.72 (d, J = 1.5 Hz, 1H), 7.45 (dd, J = 1.9, 0.8 Hz, 1H), 7.42 – 7.34 (m, 2H), 7.33 – 7.27 (m, 1H), 7.26 – 7.21 (m, 2H), 6.35 (dd, J = 3.3, 1.9 Hz, 1H), 6.24 (dt, J = 3.2, 0.6 Hz, 1H), 4.61 (s, 1H), 4.60 (d, J = 1.5 Hz, 1H), 3.97 (td, J = 8.0, 6.2 Hz, 1H), 3.33 (ddd, J = 8.2, 6.4, 1.5 Hz, 1H), 2.95 (p, J = 7.0 Hz, 1H), 1.29 (d, J = 7.1 Hz, 3H). **^{13}C NMR** (101 MHz, $CDCl_3$) δ = 203.2, 150.4, 142.8, 142.7, 129.0, 127.3, 127.2, 110.6, 108.9, 76.0, 58.1, 38.2, 36.4, 16.0. **IR** (ATR): 3027, 2970, 2739, 1717, 1552, 1494, 1452, 1376, 1237, 1188, 1147, 1083, 1014, 969, 914, 883, 811, 737, 701. **HRMS** (ESI) m/z calcd for $C_{16}H_{17}NNaO_4^+$ 310.1050 [M + Na]$^+$; found: 310.1052.

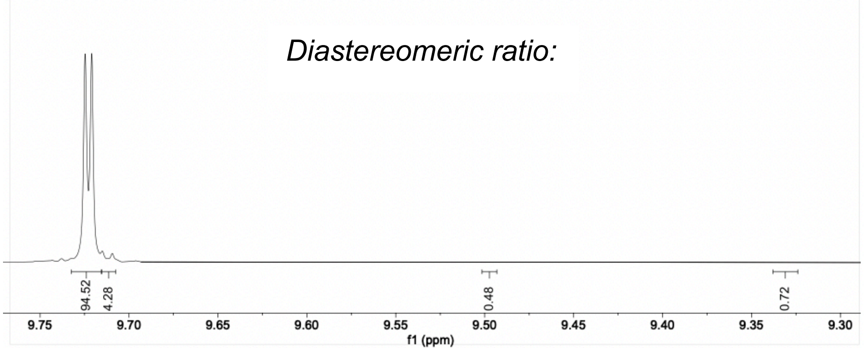

The enantiomeric excess was determined by chiral stationary phase HPLC with an IC column (*n*-hexane/*i*-PrOH 96:4, 25 °C) at 1 mL/min, UV detection at λ = 210 nm: t_R (major) = 21.4 min, t_R (minor) = 26.5 min.

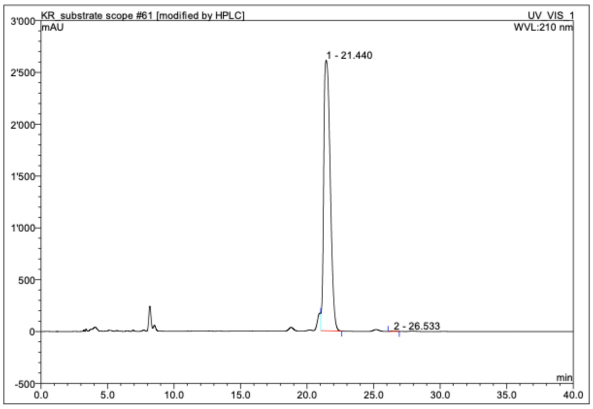

No.	Ret.Time min	Peak Name	Height mAU	Area mAU*min	Rel.Area %	Amount	Type
1	21.44	n.a.	2610.369	1485.347	99.86	n.a.	MB*
2	26.53	n.a.	4.712	2.095	0.14	n.a.	BMB*
Total:			2615.080	1487.442	100.00	0.000	

Isolated product

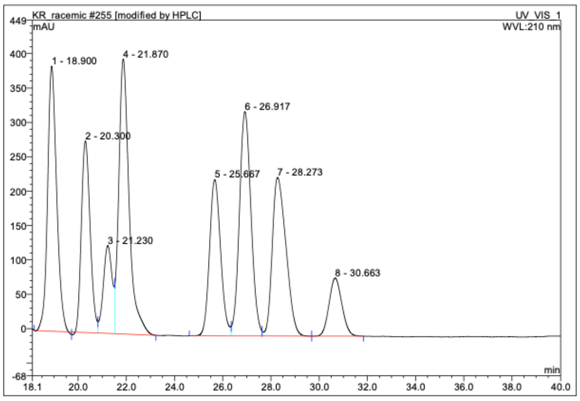

No.	Ret.Time min	Peak Name	Height mAU	Area mAU*min	Rel.Area %	Amount	Type
1	18.90	n.a.	385.385	166.979	15.97	n.a.	BM *
2	20.30	n.a.	278.500	116.955	11.19	n.a.	M *
3	21.23	n.a.	127.790	55.663	5.32	n.a.	M *
4	21.87	n.a.	399.997	197.471	18.89	n.a.	MB*
5	25.67	n.a.	227.492	121.874	11.66	n.a.	BM *
6	26.92	n.a.	326.280	181.675	17.38	n.a.	M *
7	28.27	n.a.	230.765	152.582	14.59	n.a.	M *
8	30.66	n.a.	84.484	52.258	5.00	n.a.	MB*
Total:			2060.691	1045.458	100.00	0.000	

Racemic sample

(Using pyrrolidine)

(2*S*,3*S*)-4-Nitro-2-((*S*)-1-phenylethyl)-3-(thiophen-2-yl)butanal (3ao)

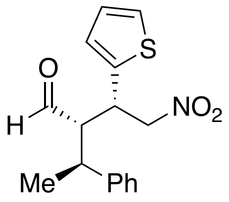

Following general procedure **IV**, **3ao** was obtained as a colorless oil (91% yield, 95:4:<1:1 d.r., 99% ee). **^{1}H NMR** (500 MHz, $CDCl_3$) δ = 9.71 (d, J = 1.4 Hz, 1H), 7.41 – 7.36 (m, 2H), 7.34 – 7.27 (m, 2H), 7.26 – 7.22 (m, 2H), 7.00 (dd, J = 5.1, 3.5 Hz, 1H), 6.97 (ddd, J = 3.5, 1.2, 0.5 Hz, 1H), 4.73 (dd, J = 12.8, 4.0 Hz, 1H), 4.60 (dd, J = 12.7, 9.9 Hz, 1H), 4.25 – 4.14 (m, 1H), 3.27 (ddd, J = 9.2, 5.7, 1.4 Hz, 1H), 3.16 – 3.08 (m, 1H), 1.31 (d, J = 7.1 Hz, 3H). **^{13}C NMR** (126 MHz, $CDCl_3$) δ = 203.6, 142.7, 140.2, 129.0, 127.3, 127.2, 127.2, 127.0, 125.4, 78.8, 60.5, 38.1, 38.0, 15.0. **IR** (ATR): 3403, 3029, 2968, 2915, 2871, 2747, 1709, 1546, 1428, 1376, 1341, 1318, 1253, 1121, 1081, 1039, 912, 852, 782, 700. **HRMS** (ESI) m/z calcd for $C_{16}H_{17}NNaO_3S^+$ 326.0821 [M + Na]$^+$; found: 326.0819.

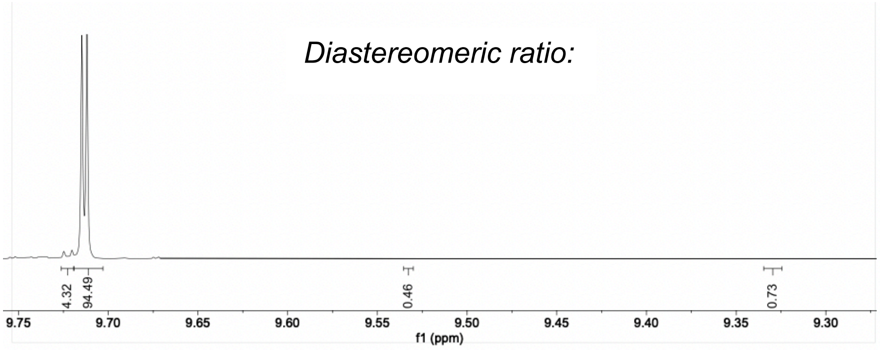

The enantiomeric excess was determined by chiral stationary phase HPLC with an IB N-5 column (n-hexane/i-PrOH 90:10, 25 °C) at 1 mL/min, UV detection at λ = 210 nm: t_R (major) = 19.9 min, t_R (minor) = 17.4 min.

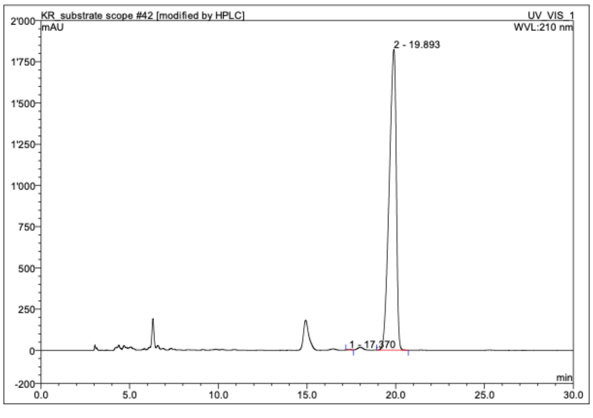

No.	Ret.Time min	Peak Name	Height mAU	Area mAU*min	Rel.Area %	Amount	Type
1	17.37	n.a.	2.602	0.597	0.07	n.a.	BMB*
2	19.89	n.a.	1824.624	837.223	99.93	n.a.	BMB*
Total:			1827.226	837.819	100.00	0.000	

Isolated product

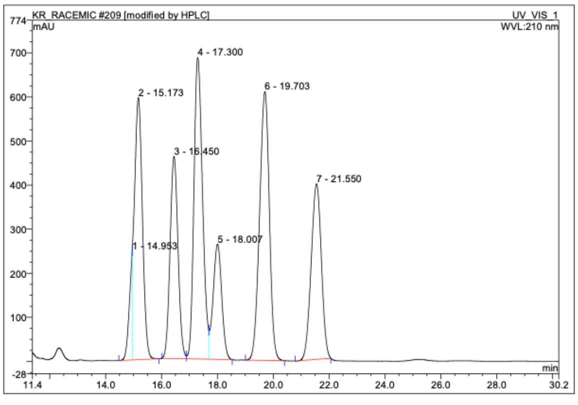

No.	Ret.Time min	Peak Name	Height mAU	Area mAU*min	Rel.Area %	Amount	Type
1	14.95	n.a.	248.182	28.815	2.56	n.a.	BM *
2	15.17	n.a.	594.054	196.505	17.43	n.a.	MB*
3	16.45	n.a.	458.314	150.281	13.33	n.a.	BM *
4	17.30	n.a.	683.754	242.771	21.53	n.a.	M *
5	18.01	n.a.	261.608	94.527	8.38	n.a.	MB*
6	19.70	n.a.	609.771	246.206	21.84	n.a.	BMB*
7	21.55	n.a.	398.148	168.377	14.93	n.a.	BMB*
Total:			3253.832	1127.482	100.00	0.000	

Racemic sample

(Using pyrrolidine)

(2*S*,3*R*)-4-nitro-2-((*S*)-1-phenylethyl)-3-(pyridin-3-yl)butanal (3ap)

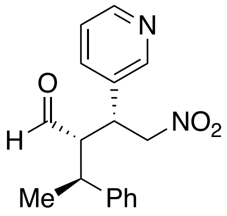

Following general procedure **IV**, **3ap** was obtained as a colorless oil (85% yield, 98:1:<1:1 d.r., 98% ee). Note that the major diastereomer could be separated from the minor diastereomers during column chromatography (crude d.r. was 89:6:2:3 after 24 h). **^{1}H NMR** (500 MHz, $CDCl_3$) δ = 9.71 (d, J = 1.5 Hz, 1H), 8.57 (dd, J = 4.9, 1.6 Hz, 1H), 8.51 (dd, J = 2.5, 0.9 Hz, 1H), 7.60 – 7.49 (m, 1H), 7.37 – 7.32 (m, 3H), 7.32 – 7.23 (m, 1H), 7.18 – 7.11 (m, 2H), 4.75 – 4.60 (m, 2H), 3.83 (td, J = 9.3, 4.6 Hz, 1H), 3.25 (ddd, J = 9.0, 6.1, 1.5 Hz, 1H), 2.92 (p, J = 7.0 Hz, 1H), 1.25 (d, J = 7.1 Hz, 3H). **^{13}C NMR** (126 MHz, $CDCl_3$) δ = 203.4, 149.74, 149.72, 142.4, 135.3, 133.4, 129.2, 127.6, 127.2, 124.1, 77.5, 59.4, 40.0, 38.6, 15.5. **IR** (ATR): 3027, 2970, 2918, 2839, 1725, 1550, 1426, 1377, 1182, 1024, 909. **HRMS** (ESI) m/z calcd for $C_{17}H_{19}N_2O_3^+$ 299.1390 [M + H]$^+$; found: 299.1388.

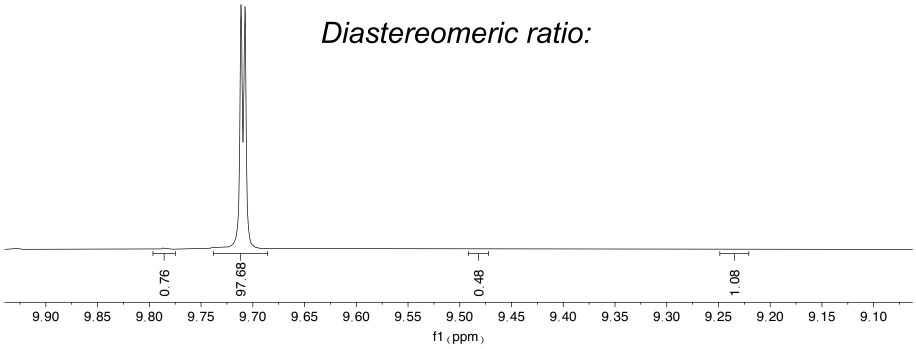

The enantiomeric excess was determined by chiral stationary phase SFC with an AD column (CO_2/MeOH 95:5, 40 °C) at 2 mL/min, UV detection at λ = 214 nm: t_R (major) = 5.3 min, t_R (minor) = 7.8 min.

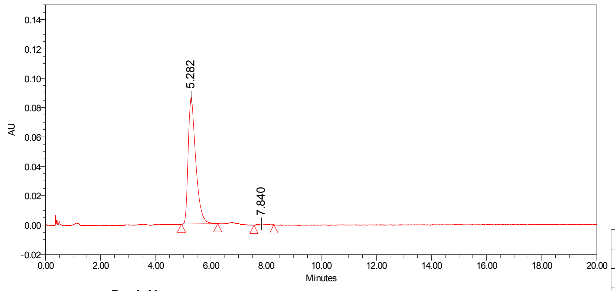

Peak Name:

	Injection	RT	Area	% Area	Height
1	1	5.282	1634200	99.19	86511
2	1	7.840	13317	0.81	746

Isolated product

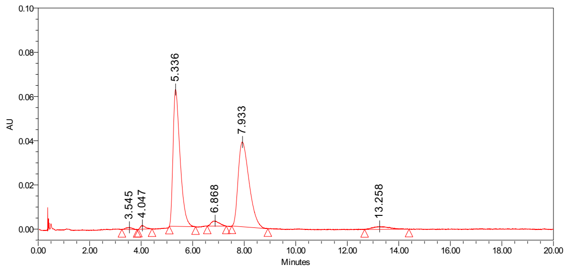

Peak Name:

	Injection	RT	Area	% Area	Height
1	1	3.545	15394	0.63	982
2	1	4.047	21199	0.87	1688
3	1	5.336	1166404	47.72	61921
4	1	6.868	51013	2.09	2409
5	1	7.933	1131711	46.30	38366
6	1	13.258	58625	2.40	1368

Racemic sample

(Using rac. peptide ***A****)*

(2*S*,3*S*)-4,4-Dimethoxy-3-(nitromethyl)-2-((*S*)-1-phenylethyl)butanal (3aq)

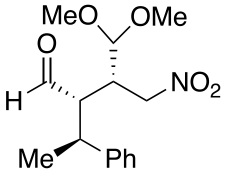

Following general procedure **IV**, **3aq** was obtained as a colourless oil (83% yield, 95:<1:5:<1 d.r., 99% ee). **^{1}H NMR** (500 MHz, $CDCl_3$) δ = 9.91 (dd, J = 2.4, 0.6 Hz, 1H), 7.40 – 7.35 (m, 2H), 7.31 – 7.26 (m, 1H), 7.25 – 7.22 (m, 2H), 4.51 – 4.37 (m, 2H), 4.21 (d, J = 3.8 Hz, 1H), 3.32 (s, 3H), 3.28 – 3.23 (m, 1H), 3.23 (s, 3H), 3.18 – 3.09 (m, 1H), 2.58 (dtdd, J = 9.2, 3.6, 2.8, 0.7 Hz, 1H), 1.33 (d, J = 6.7 Hz, 3H). **^{13}C NMR** (126 MHz, $CDCl_3$) δ = 204.1, 143.2, 129.1, 127.3, 127.2, 105.0, 72.6, 55.8, 55.6, 54.1, 41.0, 39.1, 20.8. **IR** (ATR): 2934, 2836, 2737, 1715, 1552, 1493, 1452, 1377, 1187, 1061, 972, 815, 763, 701. **HRMS** (ESI) m/z calcd for $C_{15}H_{21}NNaO_5^+$ 318.1312 [*M* + Na]$^+$; found: 318.1316.

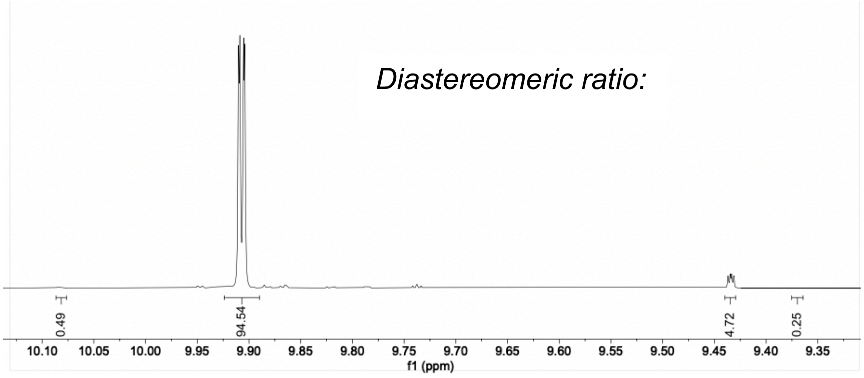

The enantiomeric excess was determined by chiral stationary phase HPLC with an IH column (*n*-hexane/*i*-PrOH 96:4, 25 °C) at 1 mL/min, UV detection at λ = 210 nm: t_R (major) = 11.0 min, t_R (minor) = 12.3 min.

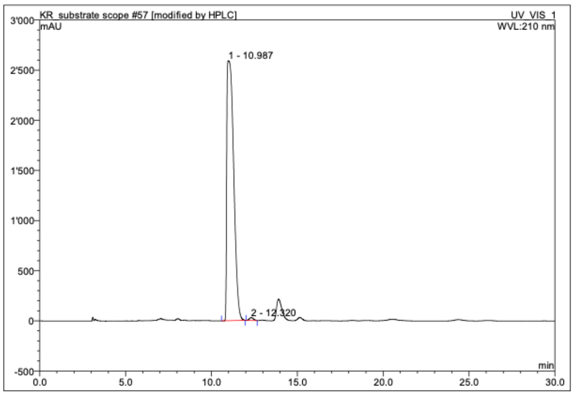

No.	Ret.Time min	Peak Name	Height mAU	Area mAU*min	Rel.Area %	Amount	Type
1	10.99	n.a.	2592.244	1202.455	99.36	n.a.	BMB*
2	12.32	n.a.	28.075	7.710	0.64	n.a.	BMB*
Total:			2620.319	1210.165	100.00	0.000	

Isolated product

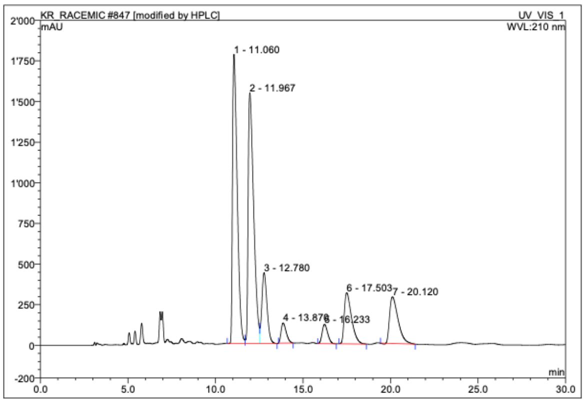

No.	Ret.Time min	Peak Name	Height mAU	Area mAU*min	Rel.Area %	Amount	Type
1	11.06	n.a.	1778.541	556.085	33.37	n.a.	BM *
2	11.97	n.a.	1540.811	560.911	33.66	n.a.	M *
3	12.78	n.a.	435.896	150.212	9.01	n.a.	MB*
4	13.87	n.a.	123.549	42.255	2.54	n.a.	BMB*
5	16.23	n.a.	119.031	43.971	2.64	n.a.	BMB*
6	17.50	n.a.	314.662	151.489	9.09	n.a.	BMB*
7	20.12	n.a.	287.500	161.646	9.70	n.a.	BMB*
Total:			4599.991	1666.570	100.00	0.000	

Racemic sample

(Using pyrrolidine)

(2*S*,3*S*)-5-Methyl-3-(nitromethyl)-2-((*S*)-1-phenylethyl)hexanal (3ar)

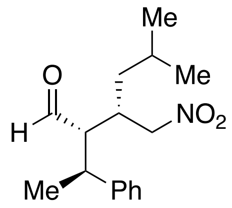

Following general procedure **IV**, **3ar** was obtained as a colorless oil (91% yield, 99:1:<1:<1 d.r., 99% ee). **^{1}H NMR** (400 MHz, $CDCl_3$) δ = 9.89 (dd, J = 2.9, 0.5 Hz, 1H), 7.42 – 7.35 (m, 2H), 7.34 – 7.29 (m, 1H), 7.26 – 7.21 (m, 2H), 4.44 (dd, J = 12.6, 4.1 Hz, 1H), 4.29 (dd, J = 12.6, 8.8 Hz, 1H), 3.18 (dq, J = 10.1, 6.8 Hz, 1H), 2.93 (ddd, J = 10.1, 3.9, 2.8 Hz, 1H), 2.33 (dddd, J = 12.9, 9.1, 5.7, 4.0 Hz, 1H), 1.72 – 1.58 (m, 1H), 1.43 – 1.36 (m, 1H), 1.35 (d, J = 6.8 Hz, 3H), 1.14 (ddd, J = 14.2, 8.6, 5.7 Hz, 1H), 0.86 (d, J = 6.6 Hz, 3H), 0.68 (d, J = 6.5 Hz, 3H). **^{13}C NMR** (101 MHz, $CDCl_3$) δ = 204.2, 142.9, 129.1, 127.4, 127.2, 76.8, 58.2, 39.7, 38.3, 34.8, 24.8, 22.8, 21.4, 20.4. **IR** (ATR): 3028, 2958, 2871, 2730, 1718, 1548, 1453, 1380, 1209, 1170, 1080, 1043, 931, 762, 700. **HRMS** (ESI) m/z calcd for $C_{16}H_{23}NNaO_3^+$ 300.1576 [M + Na]$^+$; found: 300.1565.

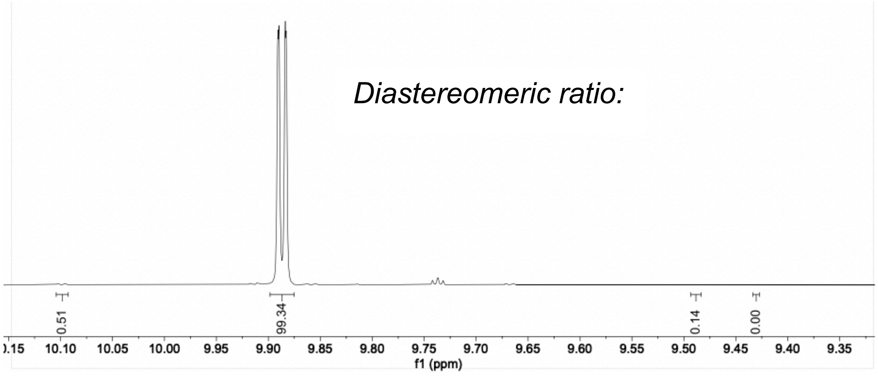

The enantiomeric excess was determined by chiral stationary phase HPLC with an IC column (*n*-hexane/*i*-PrOH 95:5, 25 °C) at 1 mL/min, UV detection at λ = 210 nm: t_R (major) = 11.1 min, t_R (minor) = 16.1 min.

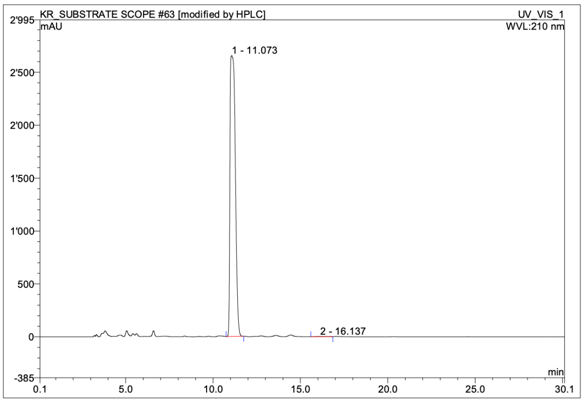

No.	Ret.Time min	Peak Name	Height mAU	Area mAU*min	Rel.Area %	Amount	Type
1	11.07	n.a.	2654.117	937.989	99.86	n.a.	BMB*
2	16.14	n.a.	2.330	1.342	0.14	n.a.	BMB*
Total:			2656.447	939.331	100.00	0.000	

Isolated product

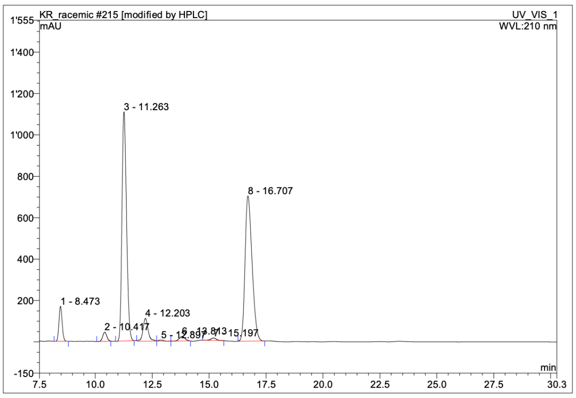

No.	Ret.Time min	Peak Name	Height mAU	Area mAU*min	Rel.Area %	Amount	Type
1	8.47	n.a.	169.123	27.422	4.70	n.a.	BMB*
2	10.42	n.a.	44.097	9.036	1.55	n.a.	BMB*
3	11.26	n.a.	1107.932	256.053	43.92	n.a.	BMB*
4	12.20	n.a.	110.120	28.846	4.95	n.a.	M *
5	12.90	n.a.	5.354	1.056	0.18	n.a.	Mb*
6	13.81	n.a.	21.832	6.527	1.12	n.a.	bMB*
7	15.20	n.a.	11.824	3.429	0.59	n.a.	BMB*
8	16.71	n.a.	701.447	250.591	42.99	n.a.	BMB*
Total:			2171.729	582.960	100.00	0.000	

Racemic sample

(Using rac. peptide **A***)*

(2*S*,3*S*)-3-Cyclohexyl-4-nitro-2-((*S*)-1-phenylethyl)butanal (3as)

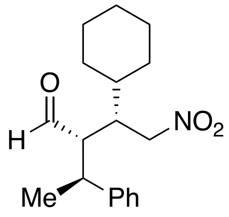

Following general procedure **IV**, **3as** was obtained as a colorless oil (59% yield, >99:<1:<1:<1 d.r., 99% ee). **^{1}H NMR** (400 MHz, $CDCl_3$) δ = 9.96 (dd, *J* = 2.7, 0.7 Hz, 1H), 7.41 – 7.34 (m, 2H), 7.31 – 7.27 (m, 1H), 7.24 – 7.20 (m, 2H), 4.53 – 4.40 (m, 2H), 3.19 (dq, *J* = 9.7, 6.8 Hz, 1H), 3.03 (dt, *J* = 9.7, 2.8 Hz, 1H), 2.31 – 2.18 (m, 1H), 1.81 – 1.60 (m, 4H), 1.37 (d, *J* = 6.9 Hz, 3H), 1.35 – 1.26 (m, 2H), 1.25 – 1.06 (m, 3H), 1.05 – 0.90 (m, 1H), 0.78 (qd, *J* = 11.9, 3.1 Hz, 1H). **^{13}C NMR** (101 MHz, $CDCl_3$) δ = 204.5, 143.0, 129.0, 127.3, 127.3, 75.0, 56.6, 42.5, 39.7, 39.7, 30.3, 30.3, 26.4, 26.3, 26.1, 20.4. **IR** (ATR): 3027, 2924, 2851, 2728, 1716, 1547, 1493, 1449, 1377, 1260, 1218, 1080, 1029, 998, 908, 830, 762, 700. **HRMS** (ESI) m/z calcd for $C_{18}H_{25}NNaO_3^+$ 326.1727 [*M* + Na]$^+$; found: 326. 1726.

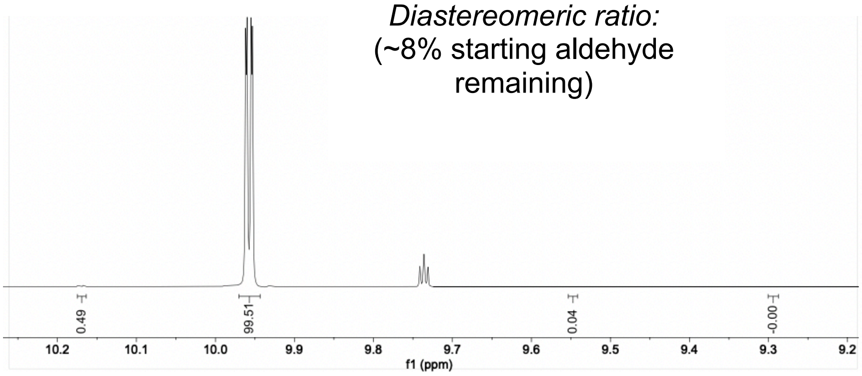

The enantiomeric excess was determined by chiral stationary phase HPLC with an IB N-5 column (*n*-hexane/*i*-PrOH 96:4, 25 °C) at 1 mL/min, UV detection at λ = 210 nm: t_R (major) = 11.5 min, t_R (minor) = 10.7 min.

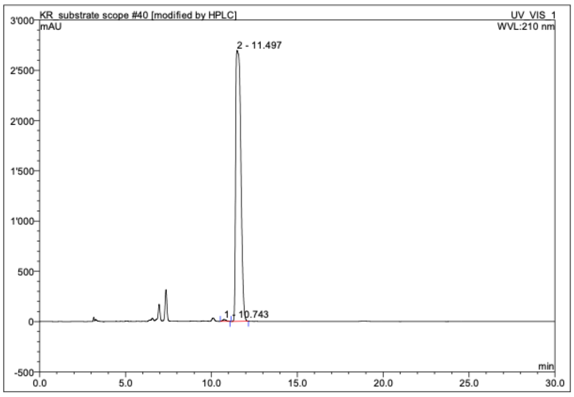

No.	Ret.Time min	Peak Name	Height mAU	Area mAU*min	Rel.Area %	Amount	Type
1	10.74	n.a.	18.147	4.315	0.45	n.a.	BMB*
2	11.50	n.a.	2695.837	945.628	99.55	n.a.	BMB*
Total:			2713.984	949.943	100.00	0.000	

Isolated product

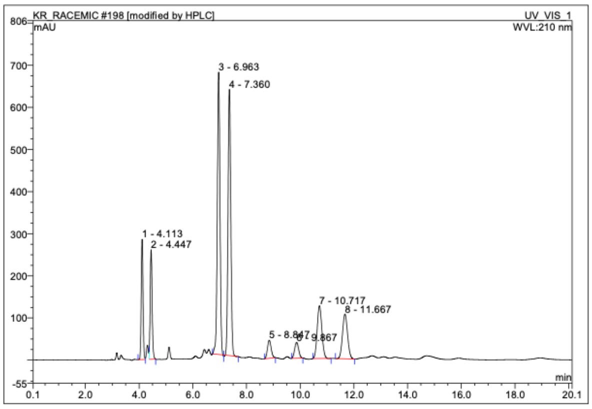

No.	Ret.Time min	Peak Name	Height mAU	Area mAU*min	Rel.Area %	Amount	Type
1	4.11	n.a.	285.665	22.049	8.57	n.a.	BM *
2	4.45	n.a.	260.239	21.767	8.46	n.a.	MB*
3	6.96	n.a.	670.568	76.635	29.77	n.a.	BM *
4	7.36	n.a.	632.210	75.135	29.19	n.a.	MB*
5	8.85	n.a.	43.112	6.866	2.67	n.a.	BMB*
6	9.87	n.a.	37.614	6.535	2.54	n.a.	BMB*
7	10.72	n.a.	125.050	25.182	9.78	n.a.	BMB*
8	11.67	n.a.	106.020	23.218	9.02	n.a.	BMB*
Total:			2160.476	257.388	100.00	0.000	

Racemic sample
(*Using pyrrolidine*)

7 ^{1}H and ^{13}C NMR Spectra of the Peptide Catalysts

TFA ·H-DPro-αMePro-Glu-NH$_2$ (B)

^{1}H NMR (400 MHz, D_2O)

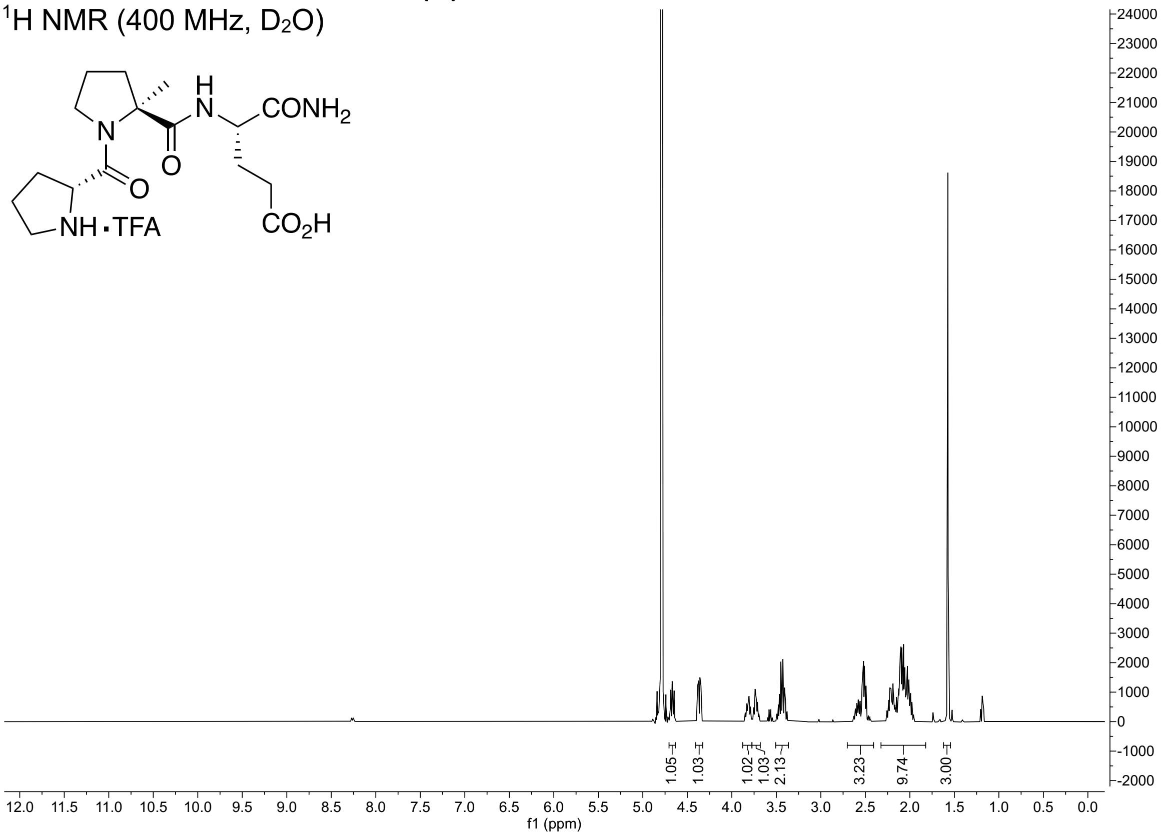

^{13}C NMR (101 MHz, D_2O)

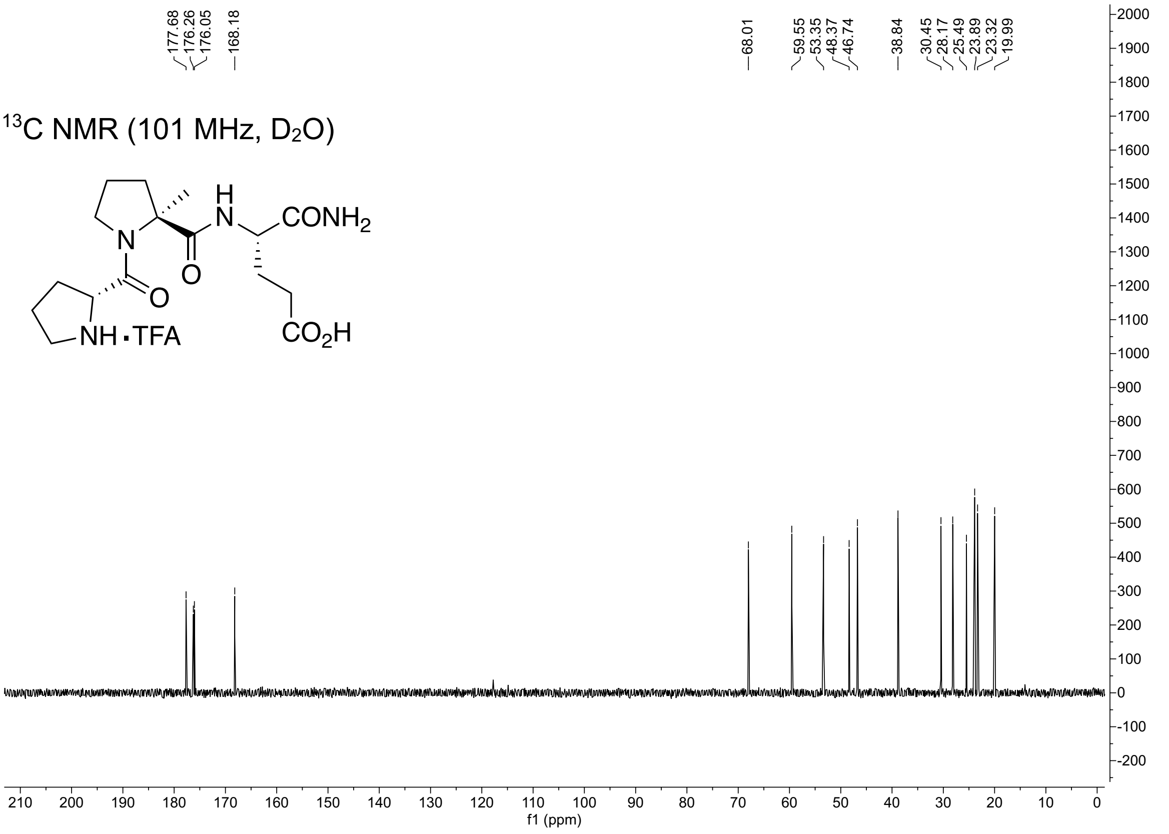

8 NMR Spectra of β-Branched Aldehydes

3-(*p*-Tolyl)butanal (1b)

^{1}H NMR (400 MHz, $CDCl_3$)

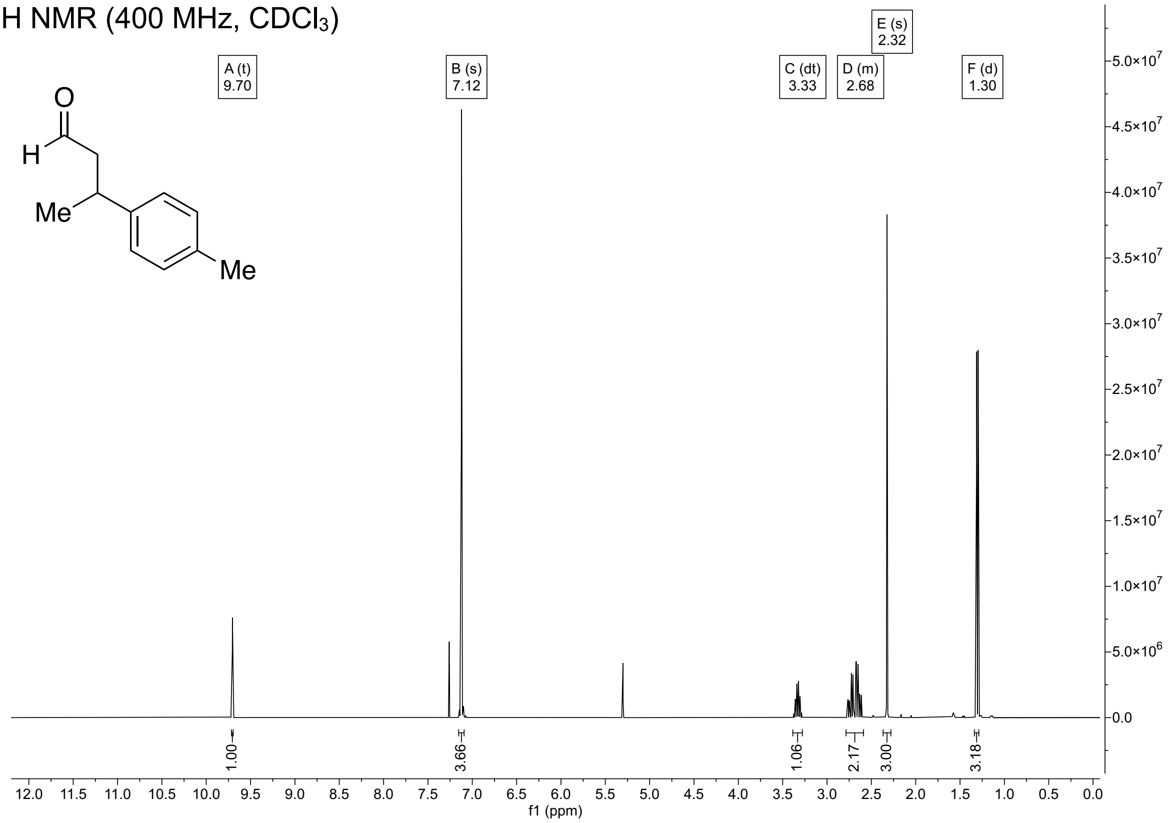

^{13}C NMR (101 MHz, $CDCl_3$)

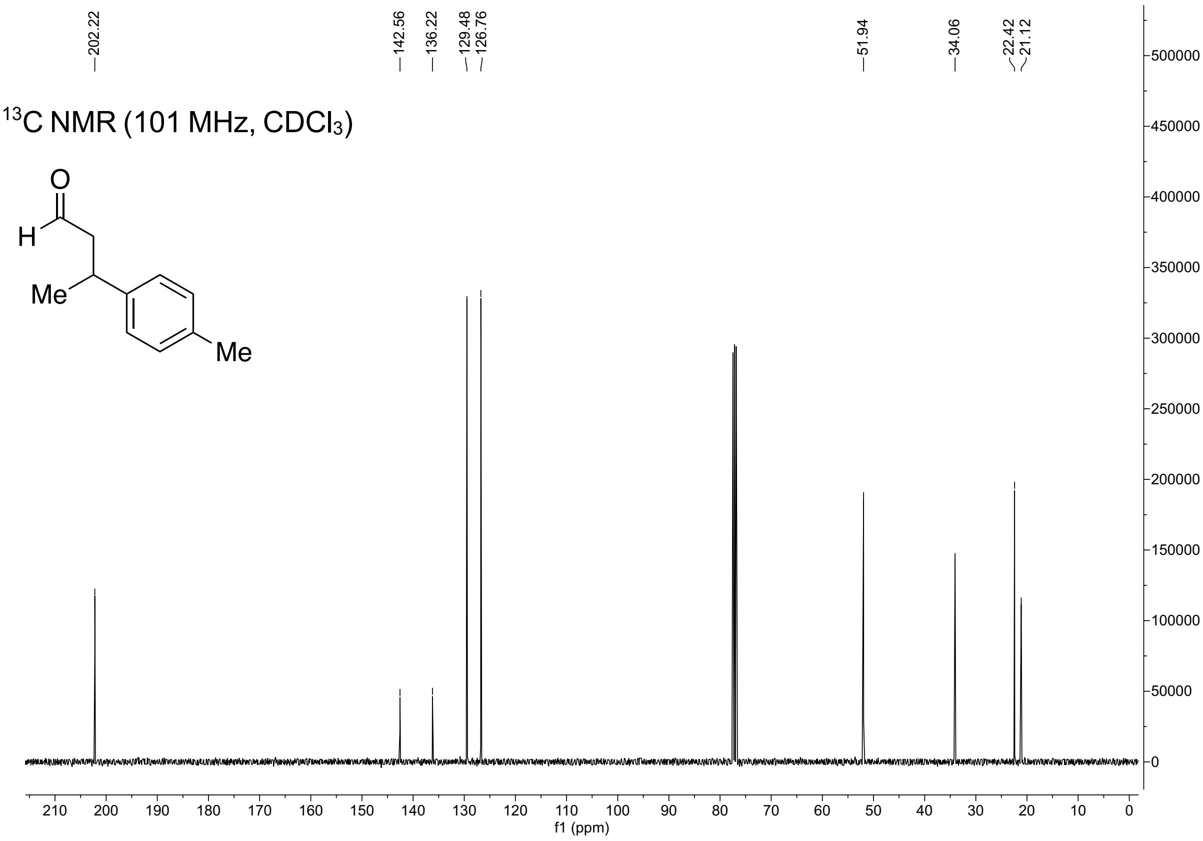

3-(4-(*tert*-Butyl)phenyl)butanal (1c)

^{1}H NMR (400 MHz, $CDCl_3$)

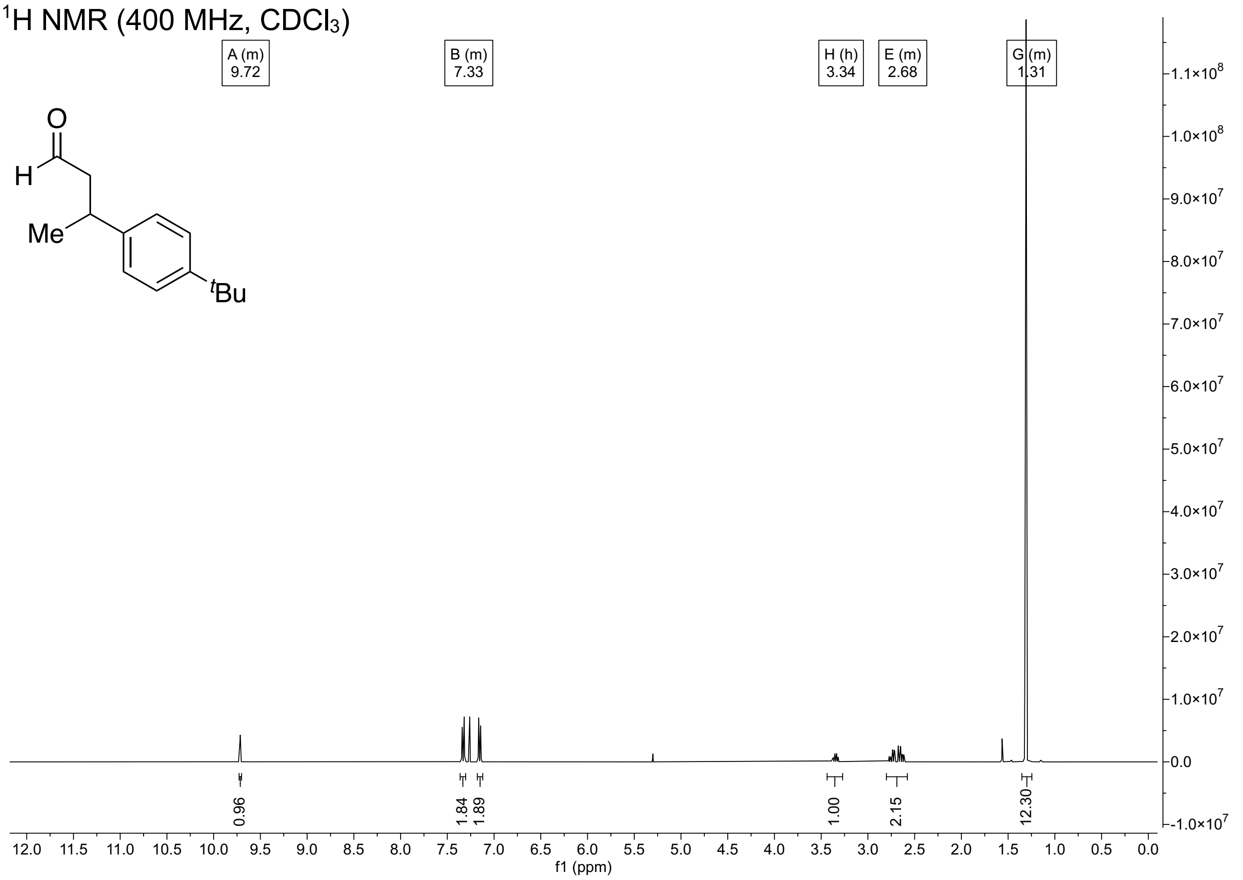

^{13}C NMR (101 MHz, $CDCl_3$)

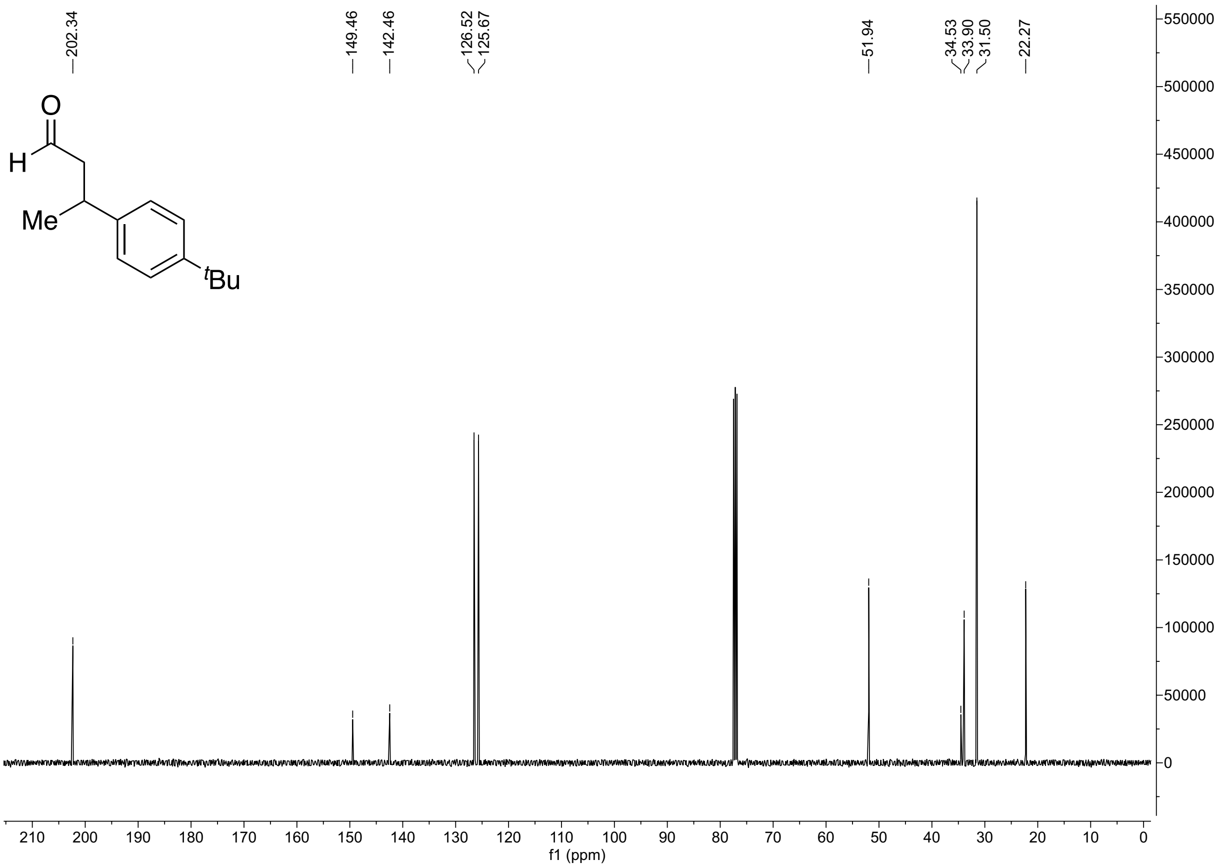

3-(4-Methoxyphenyl)butanal (1d)

^{1}H NMR (400 MHz, $CDCl_3$)

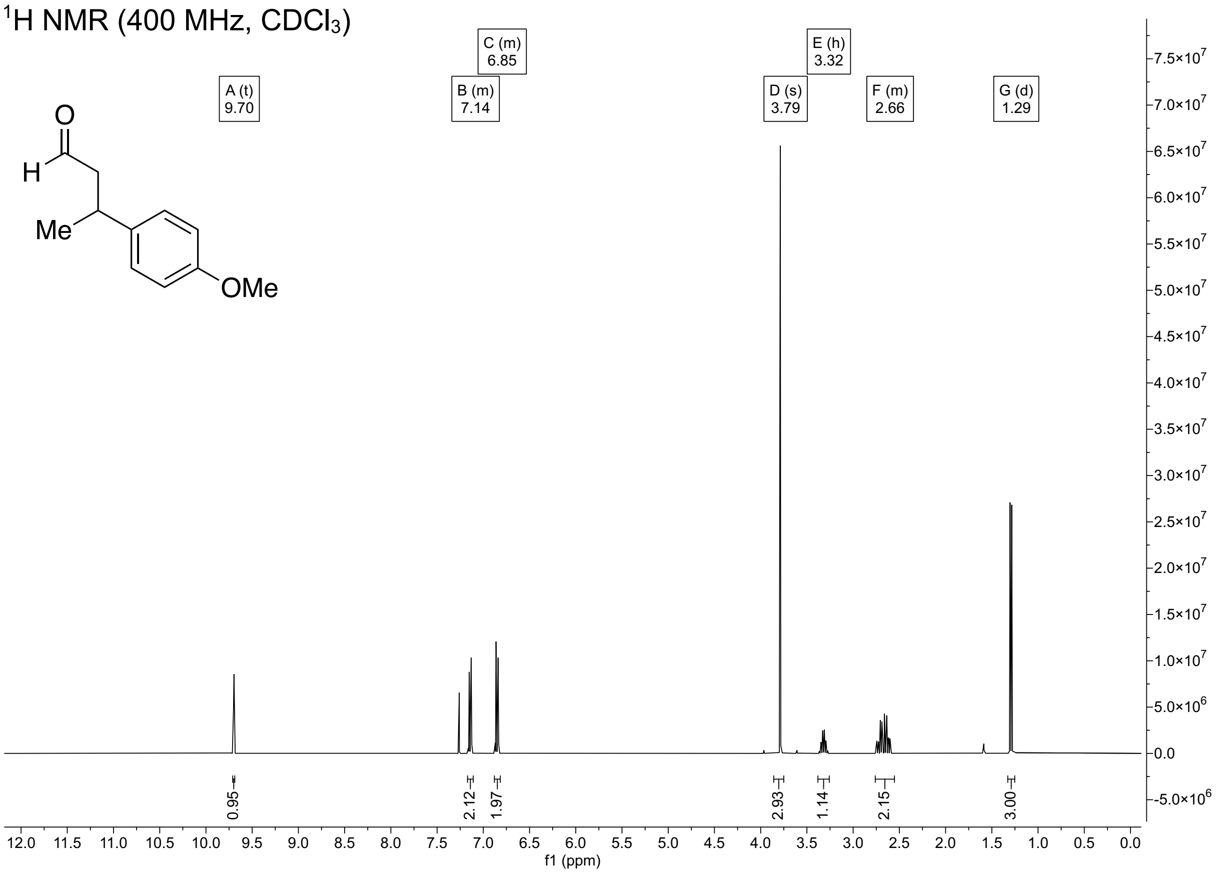

^{13}C NMR (101 MHz, $CDCl_3$)

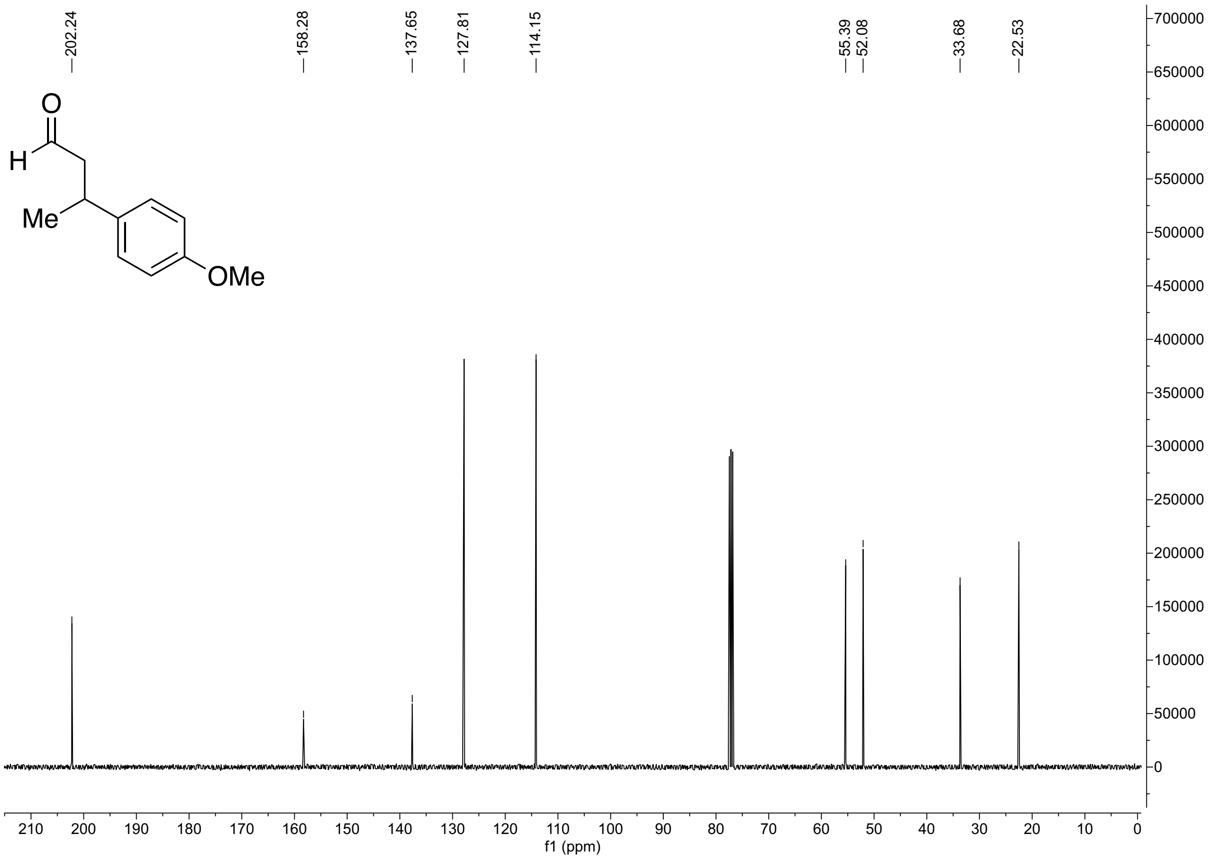

3-(4-(Trifluoromethyl)phenyl)butanal (1e)

^{1}H NMR (400 MHz, $CDCl_3$)

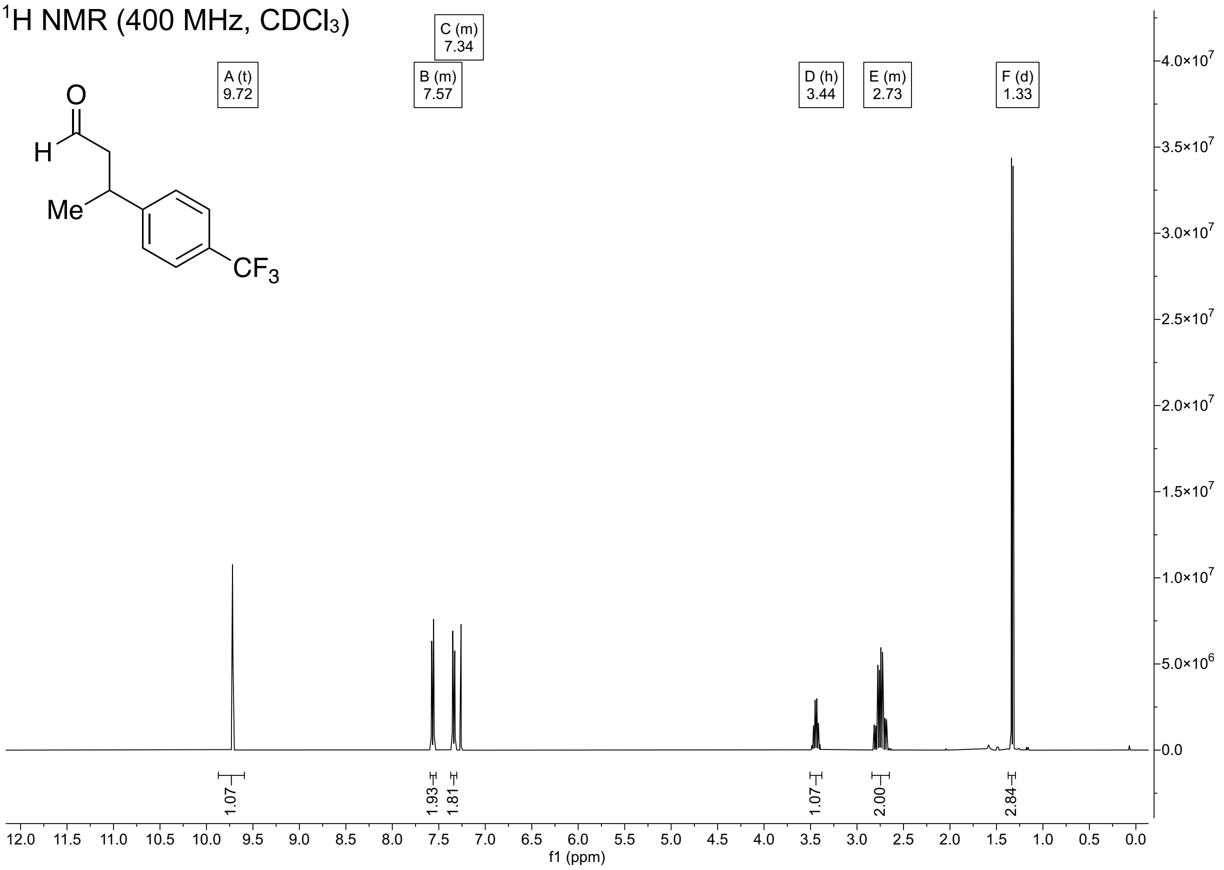

^{13}C NMR (101 MHz, $CDCl_3$)

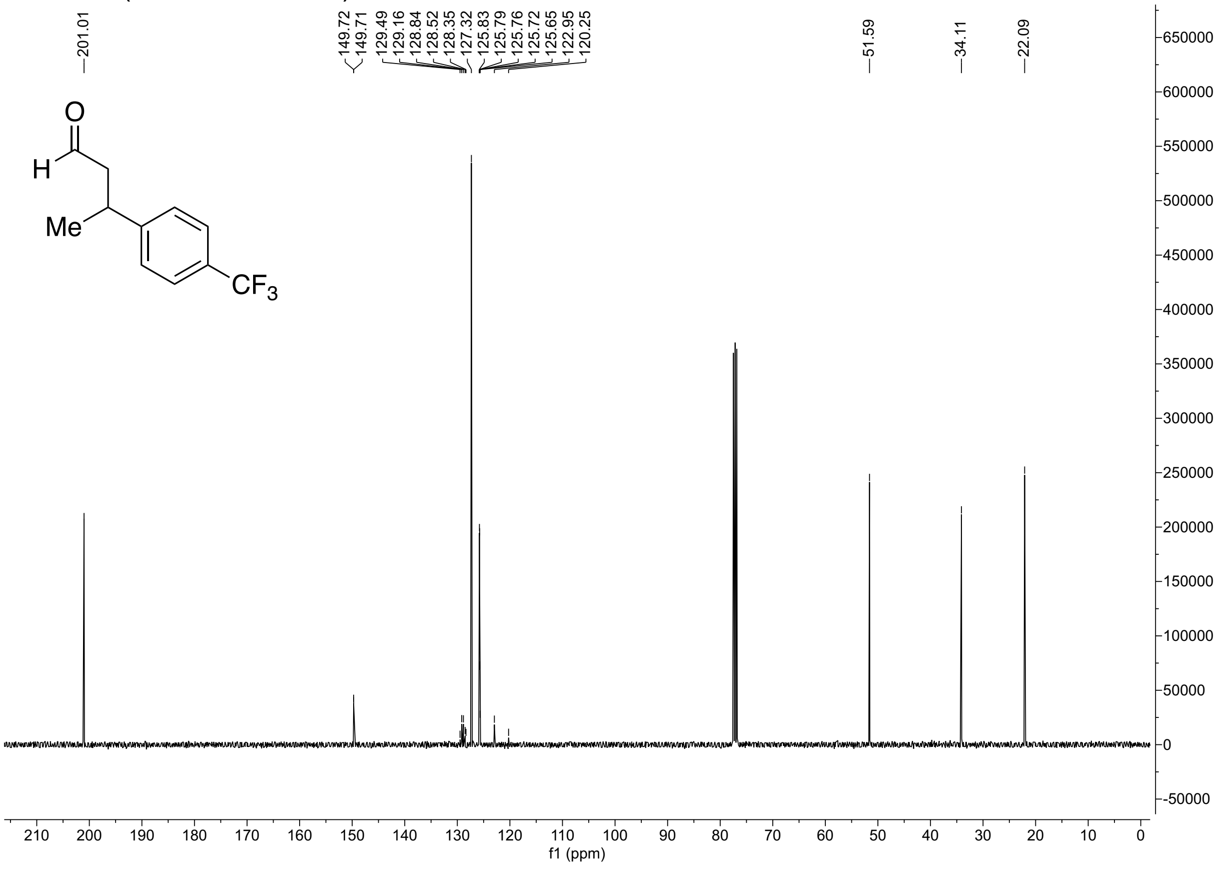

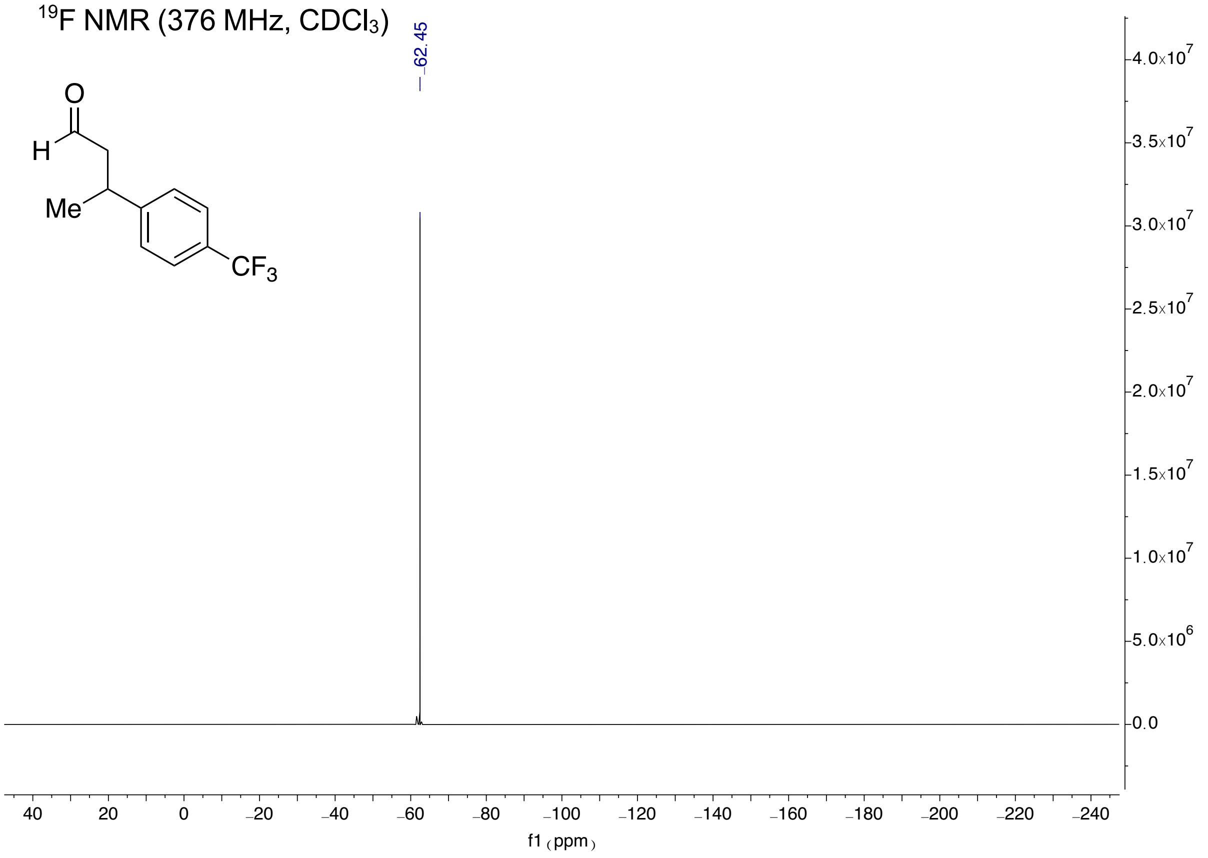

19F NMR (376 MHz, CDCl3)
O
H
Me
CF3
−62.45
4.0×10^7
3.5×10^7
3.0×10^7
2.5×10^7
2.0×10^7
1.5×10^7
1.0×10^7
5.0×10^6
0.0
40
20
0
−20
−40
−60
−80
−100
−120
−140
−160
−180
−200
−220
−240
f1 (ppm)

Methyl 4-(4-oxobutan-2-yl)benzoate (1f)

^{1}H NMR (400 MHz, $CDCl_3$)

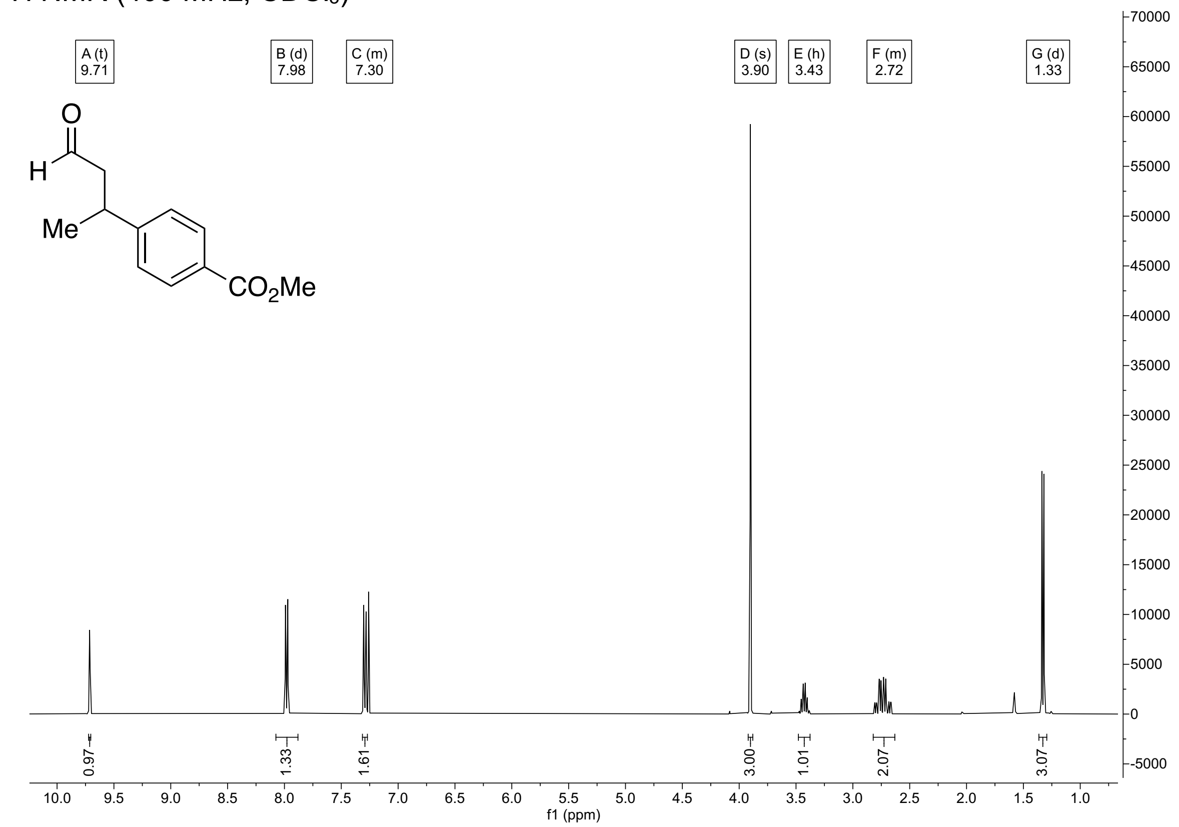

^{13}C NMR (101 MHz, $CDCl_3$)

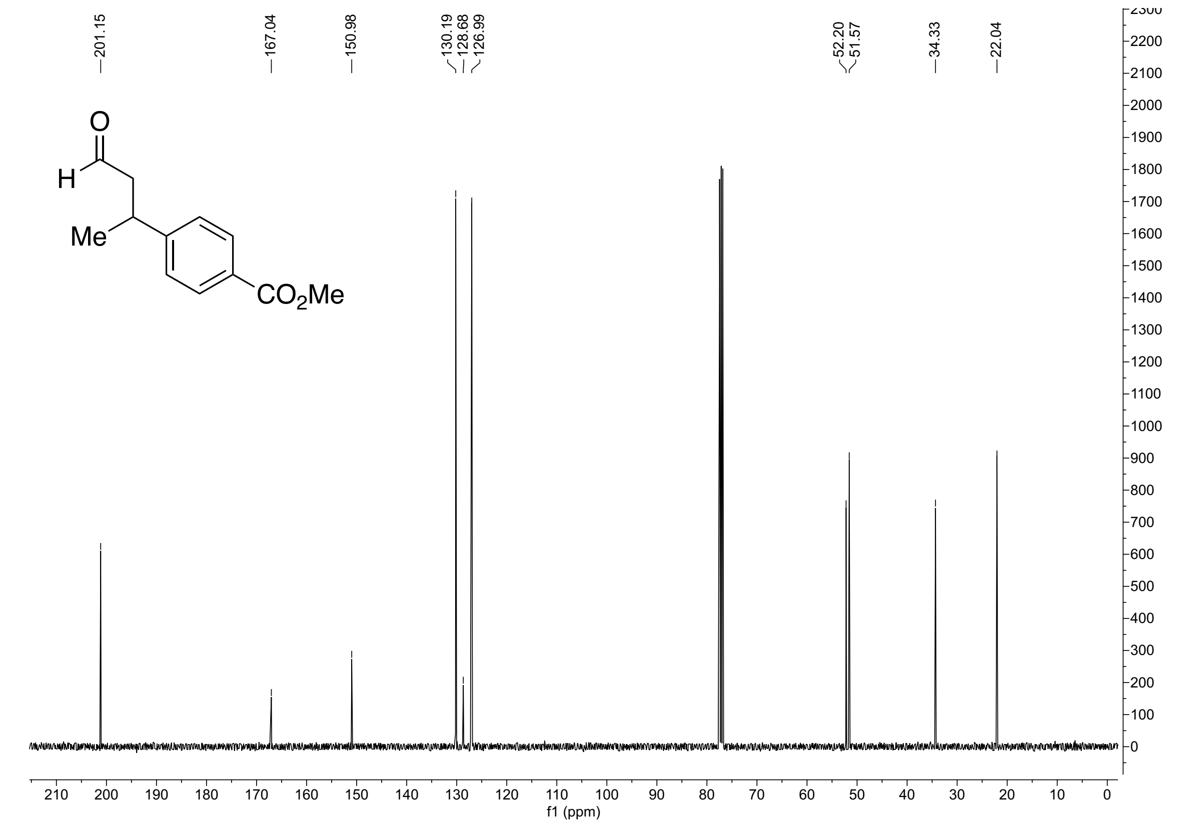

3-(4-Fluorophenyl)butanal (1g)

^{1}H NMR (400 MHz, $CDCl_3$)

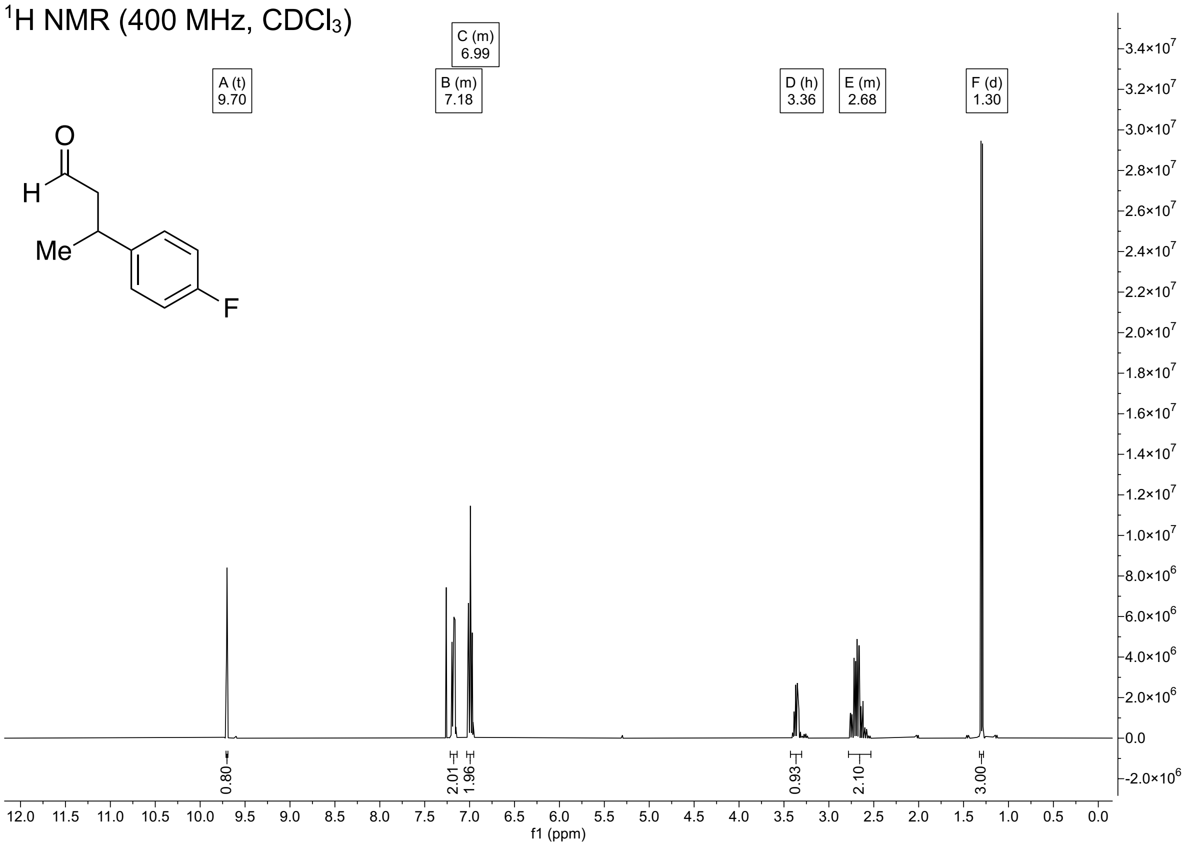

^{13}C NMR (101 MHz, $CDCl_3$)

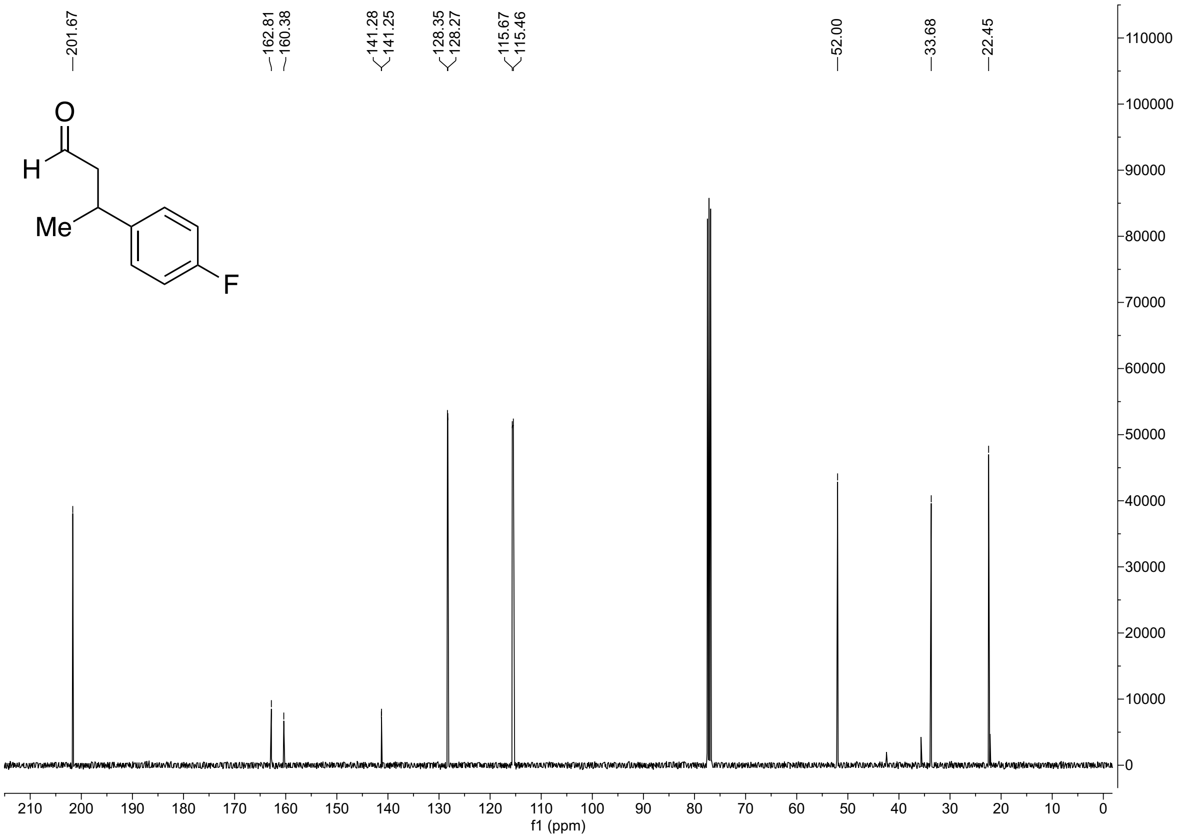

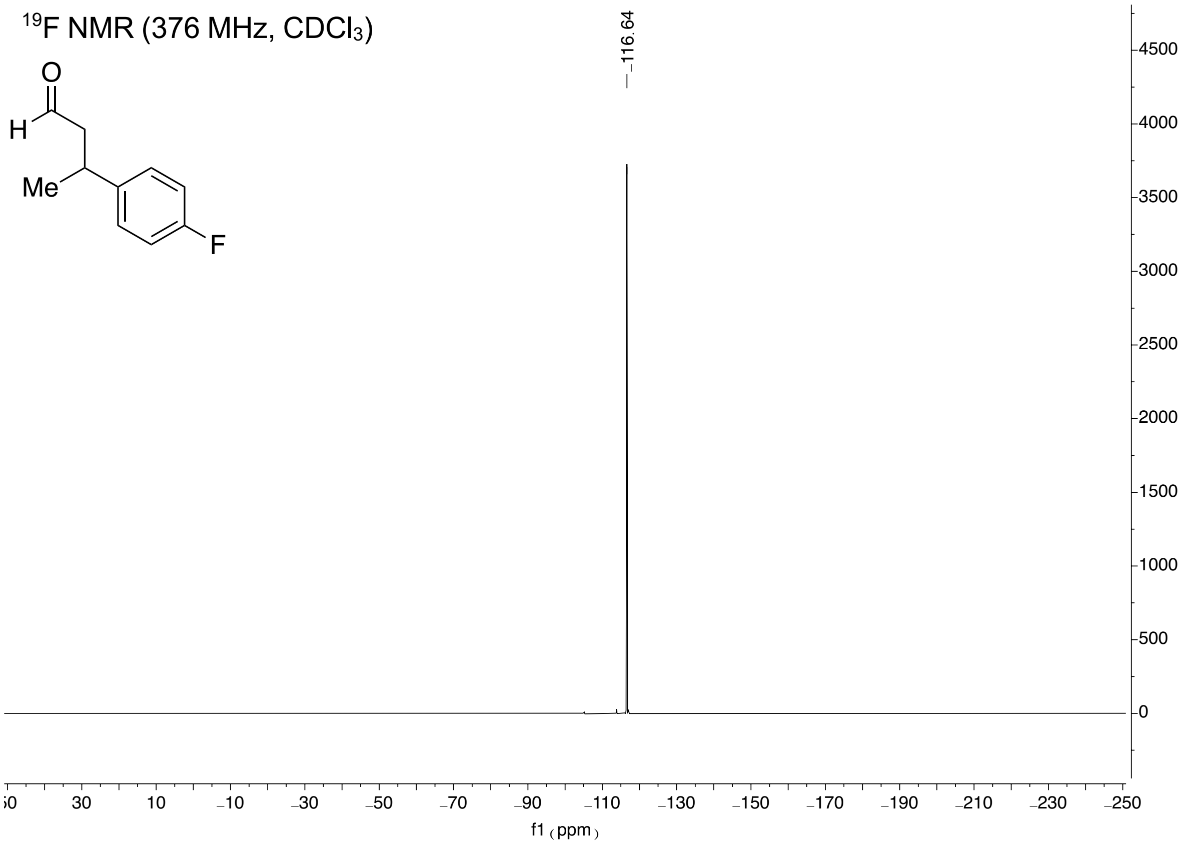

19F NMR (376 MHz, CDCl3)
O
H
Me
F
-116.64
4500
4000
3500
3000
2500
2000
1500
1000
500
0
30
10
-10
-30
-50
-70
-90
-110
-130
-150
-170
-190
-210
-230
-250
f1 (ppm)

3-(3-Fluorophenyl)butanal (1h)

^{1}H NMR (400 MHz, $CDCl_3$)

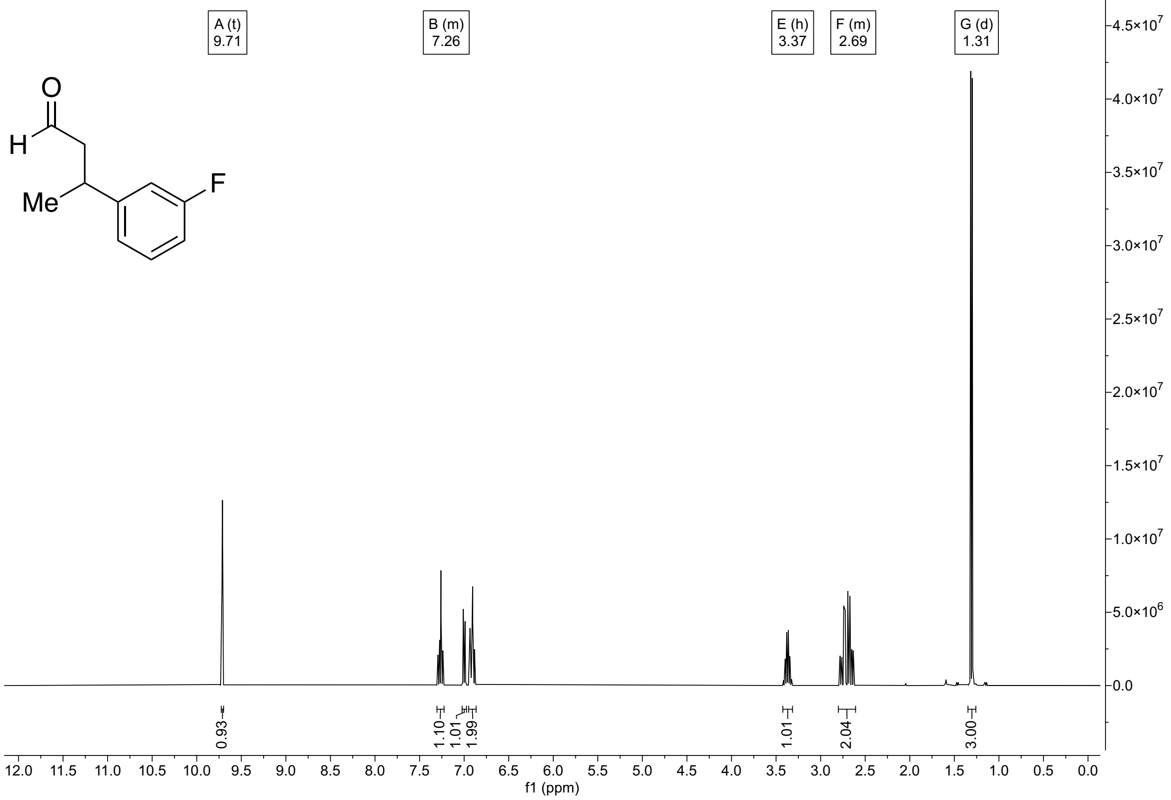

^{13}C NMR (101 MHz, $CDCl_3$)

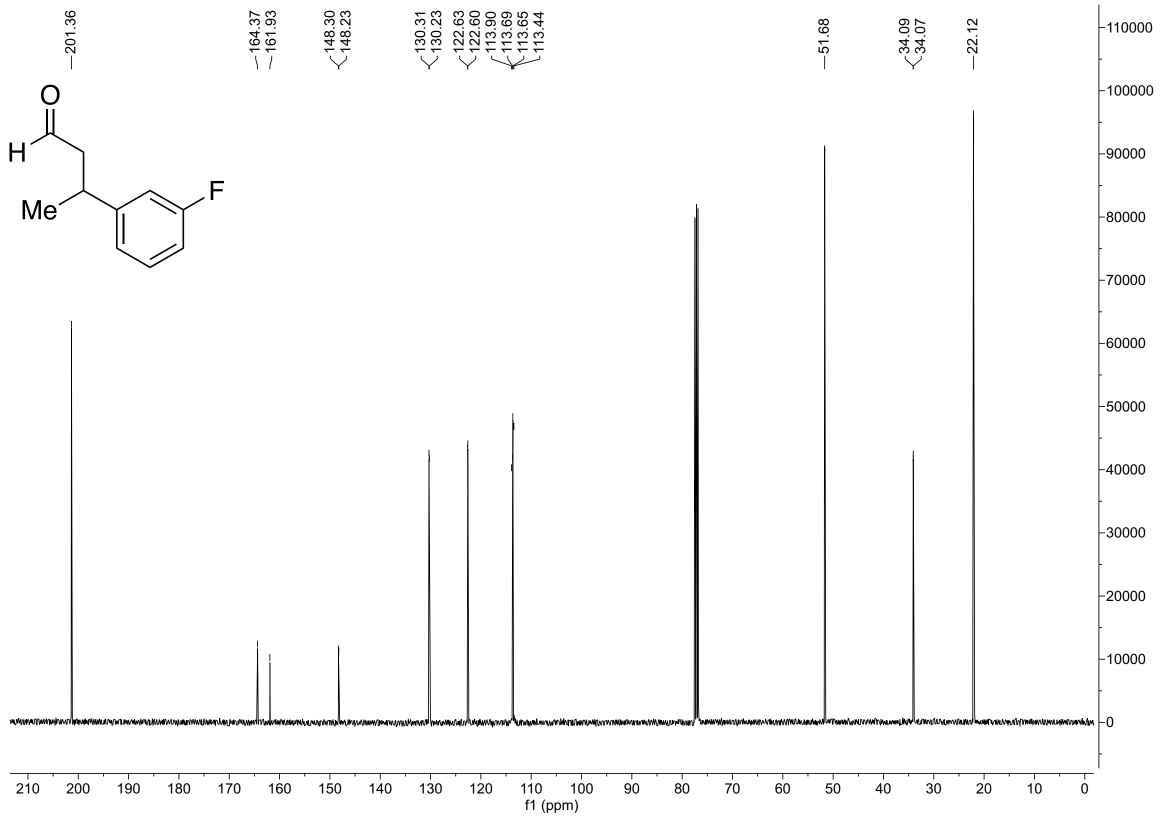

^{19}F NMR (376 MHz, $CDCl_3$)

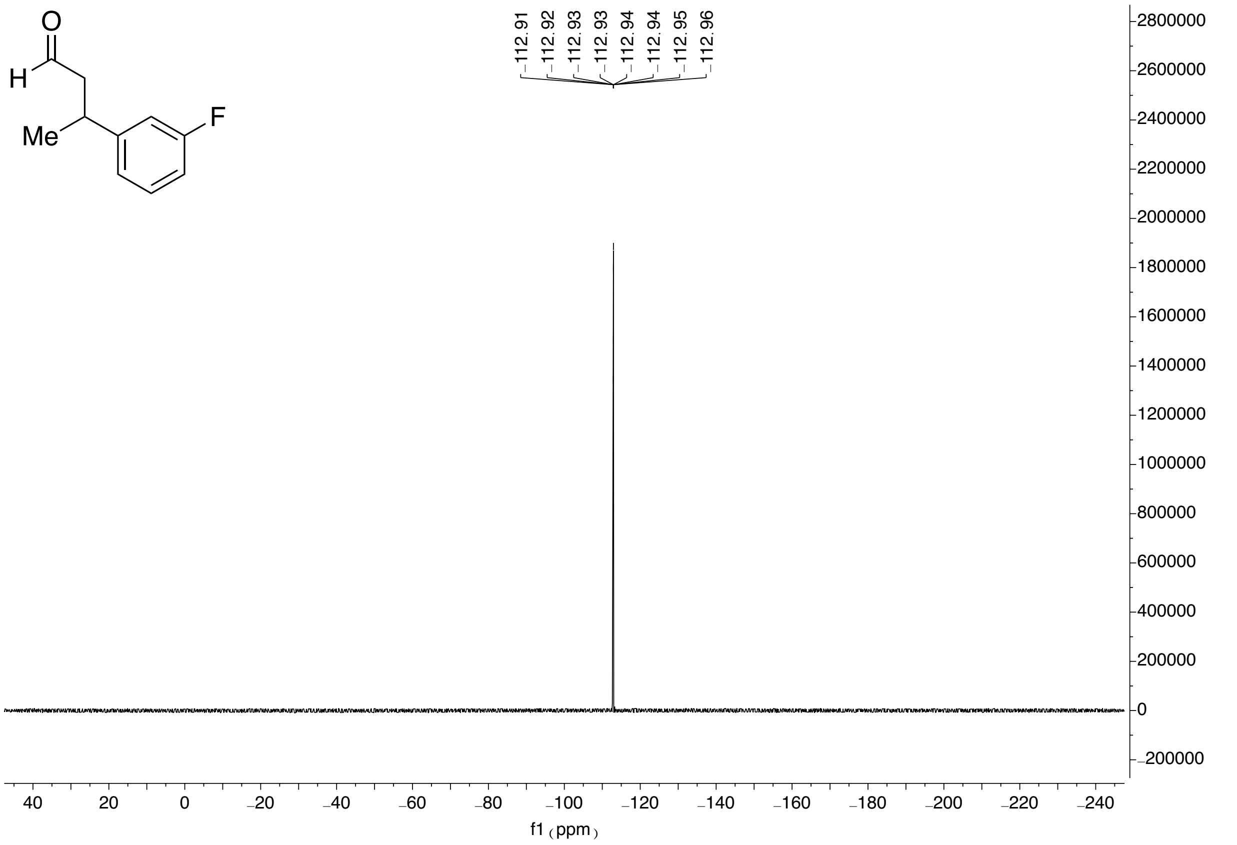

3-(2-Fluorophenyl)butanal (1i)

^{1}H NMR (400 MHz, $CDCl_3$)

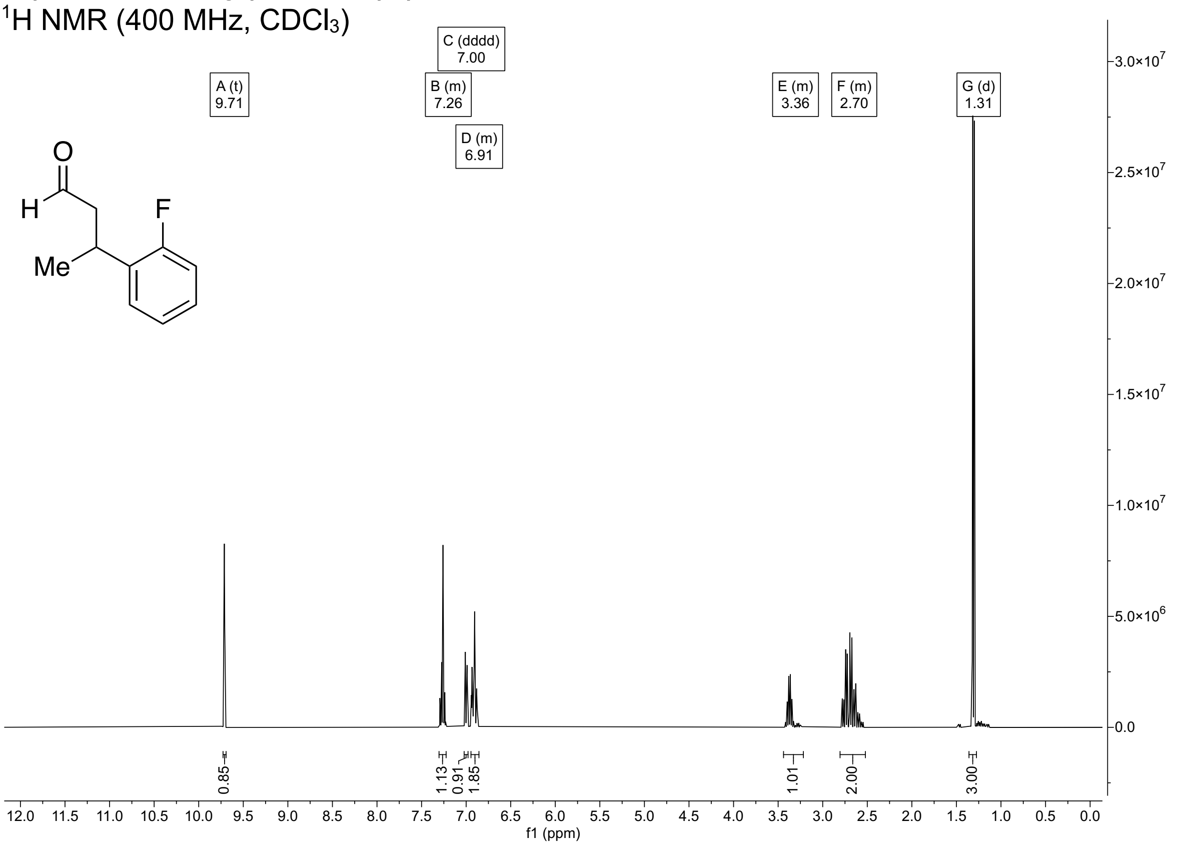

^{13}C NMR (101 MHz, $CDCl_3$)

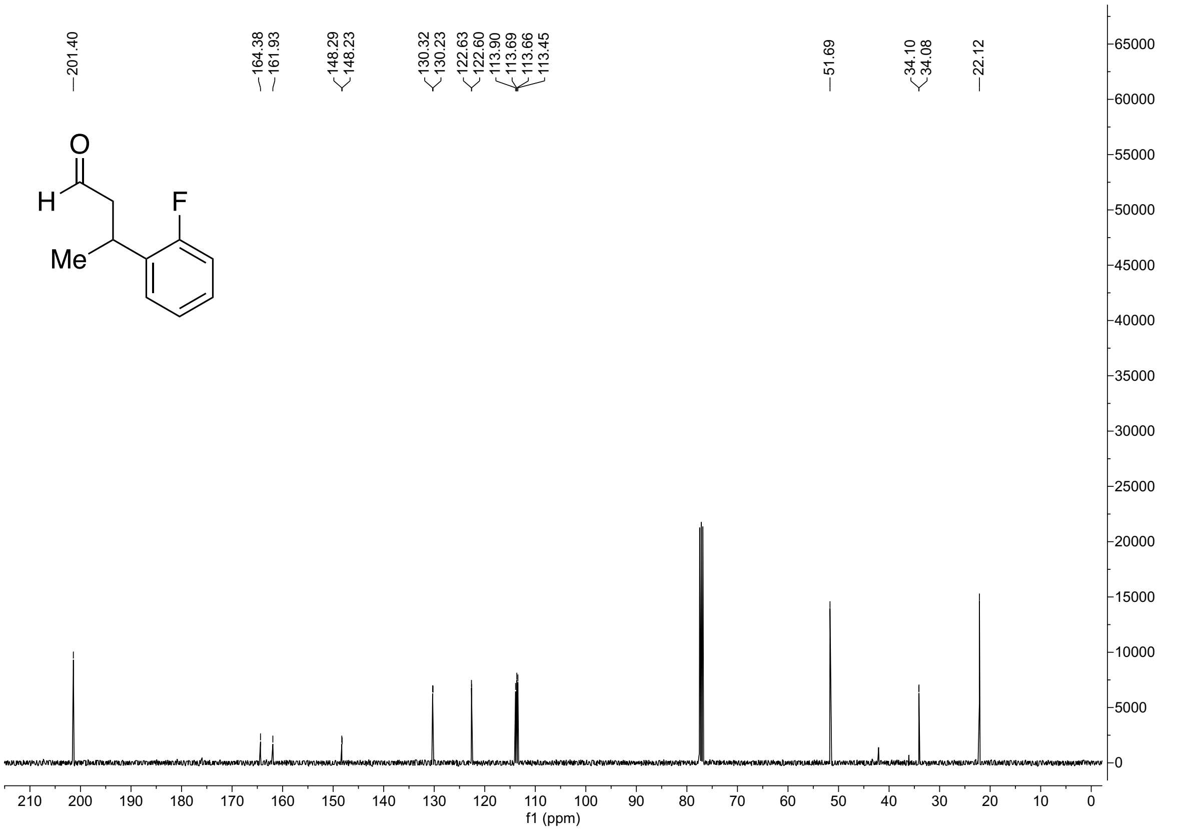

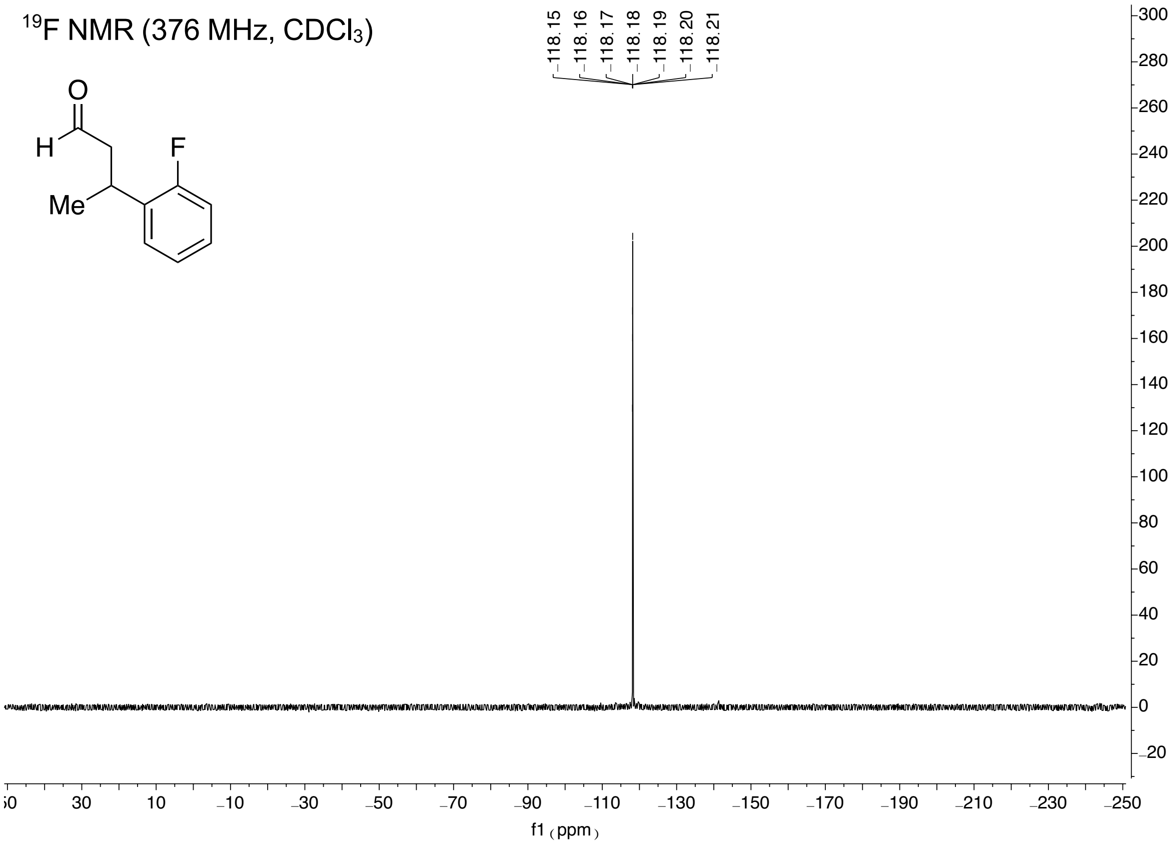

^{19}F NMR (376 MHz, CDCl$_3$)
O
H
F
Me
-118.15
-118.16
-118.17
-118.18
-118.19
-118.20
-118.21
f1 (ppm)

3-(4-Fluorophenyl)pentanal (1k)

^{1}H NMR (400 MHz, $CDCl_3$)

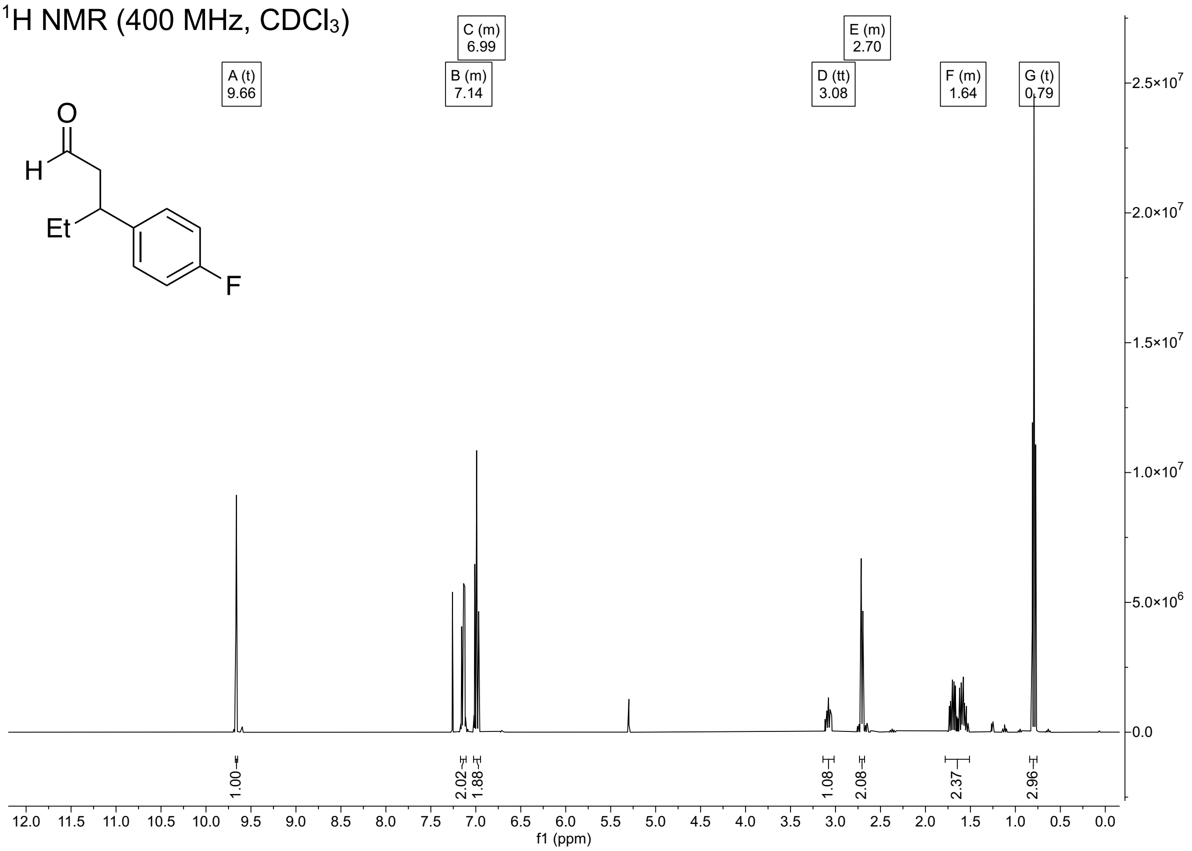

^{13}C NMR (101 MHz, $CDCl_3$)

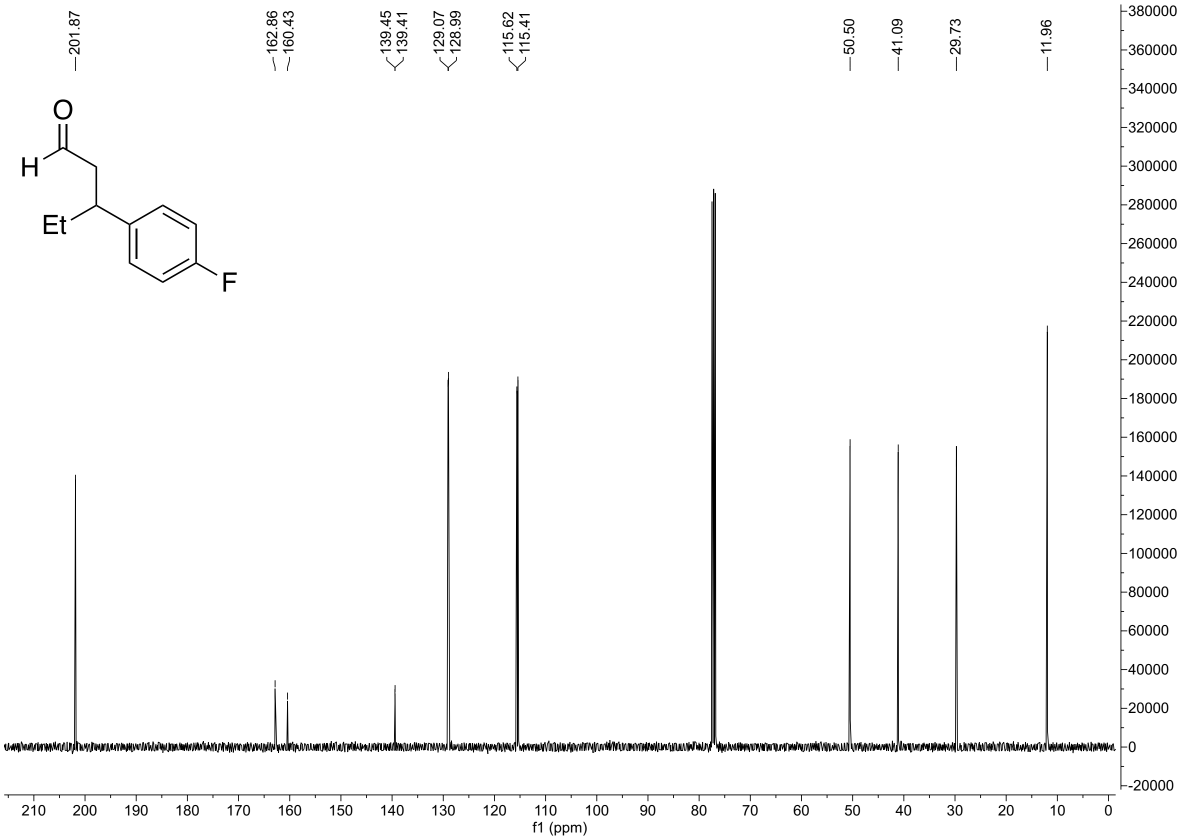

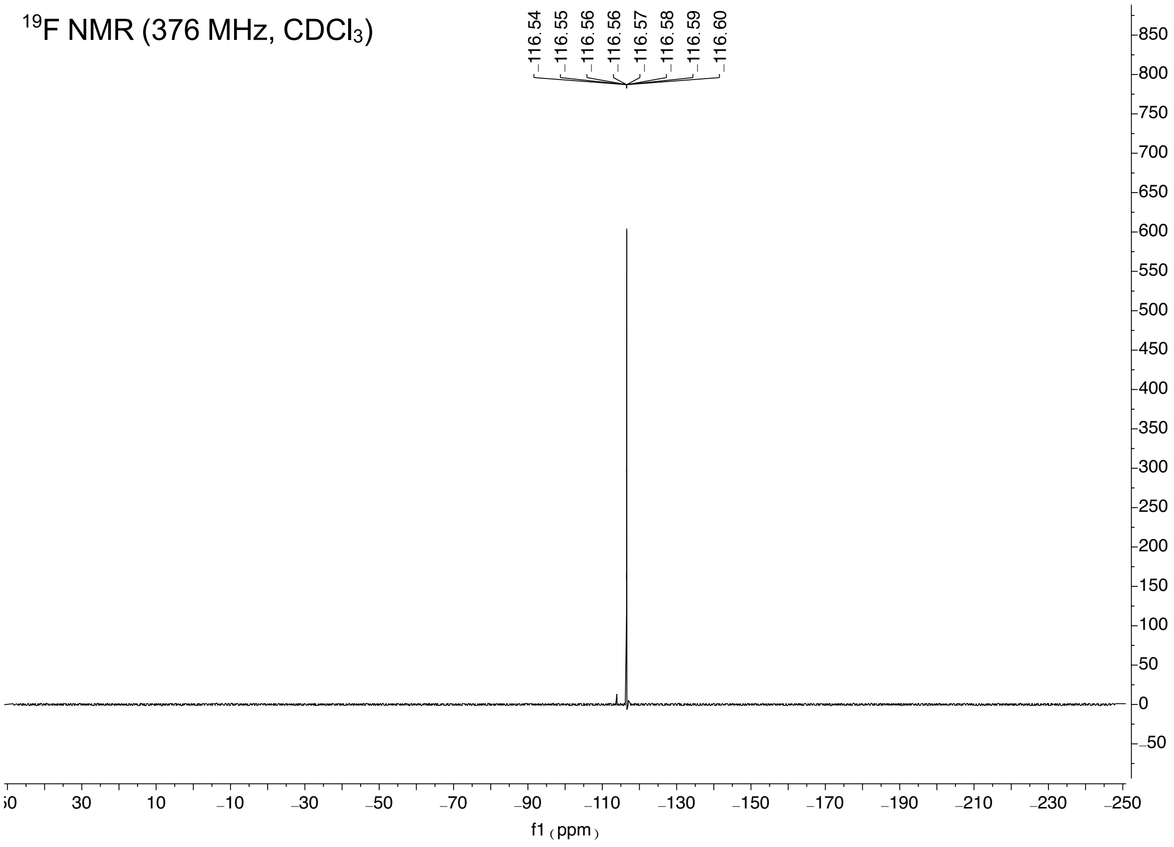

^{19}F NMR (376 MHz, CDCl$_3$)
-116.54
-116.55
-116.56
-116.56
-116.57
-116.58
-116.59
-116.60
f1 (ppm)

3-(4-Fluorophenyl)hexanal (1l)

^{1}H NMR (400 MHz, $CDCl_3$)

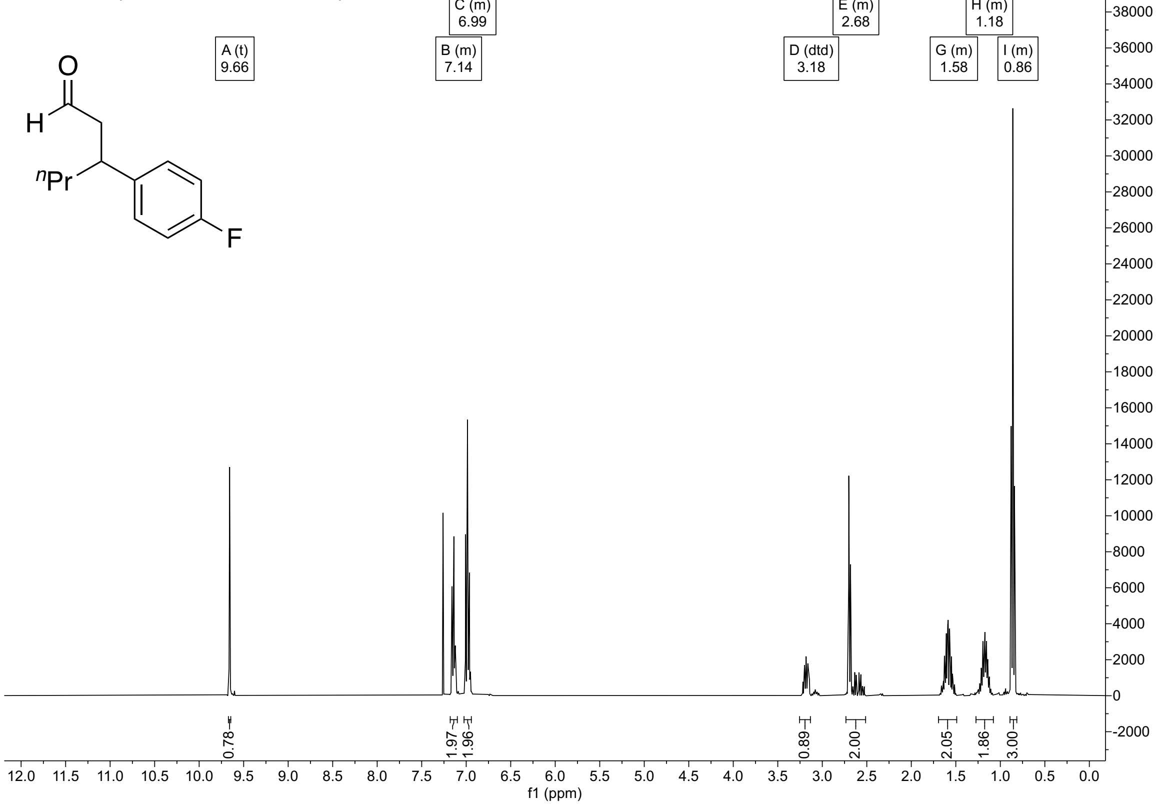

^{13}C NMR (101 MHz, $CDCl_3$)

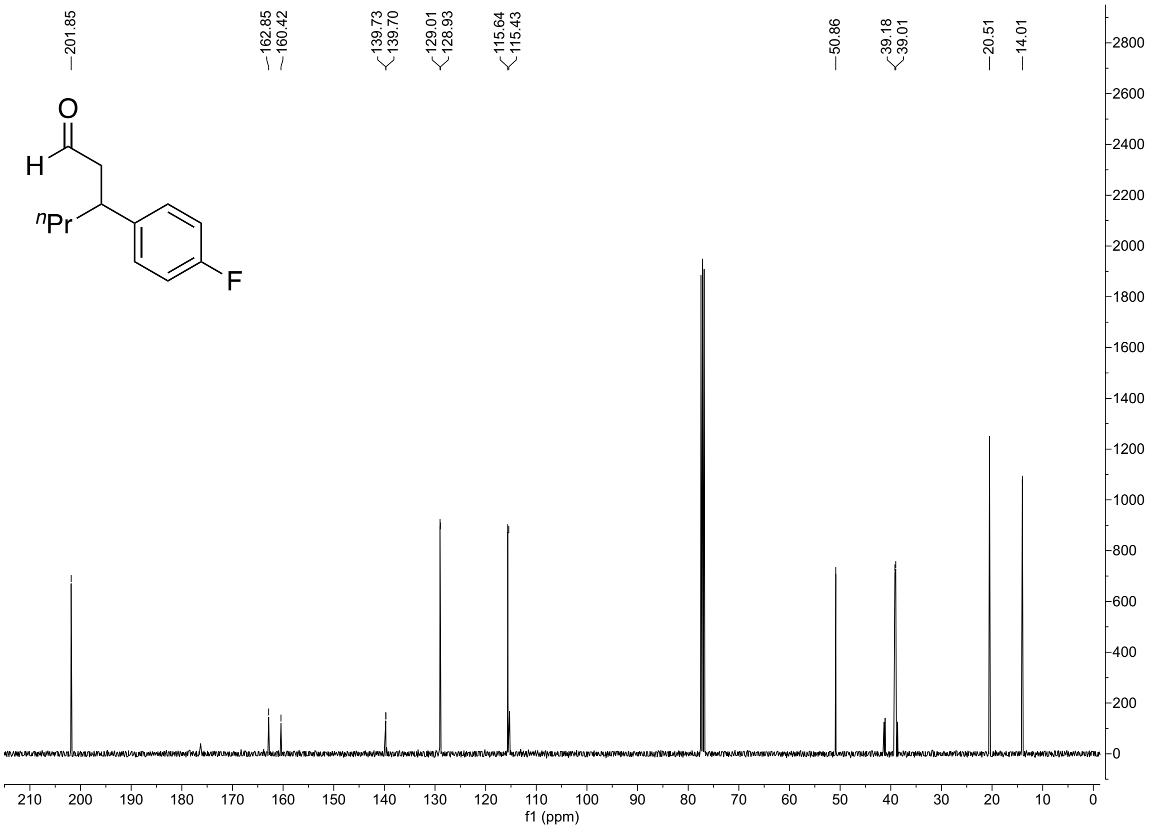

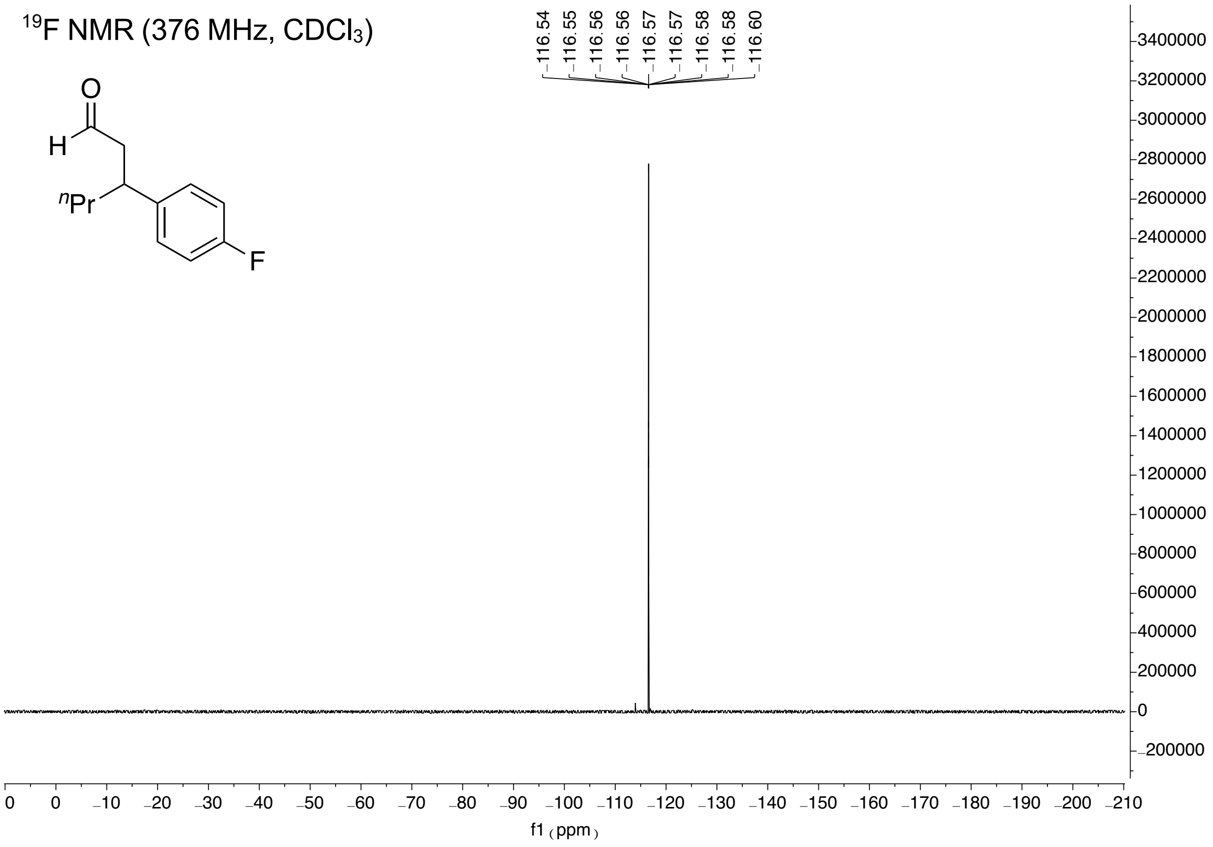

^{19}F NMR (376 MHz, CDCl$_3$)
O
H
^{n}Pr
F
-116.54
-116.55
-116.56
-116.56
-116.57
-116.57
-116.58
-116.58
-116.60
3400000
3200000
3000000
2800000
2600000
2400000
2200000
2000000
1800000
1600000
1400000
1200000
1000000
800000
600000
400000
200000
0
-200000
0 0 -10 -20 -30 -40 -50 -60 -70 -80 -90 -100 -110 -120 -130 -140 -150 -160 -170 -180 -190 -200 -210
f1 (ppm)

3-(4-Fluorophenyl)dodecanal (1m)

^{1}H NMR (400 MHz, $CDCl_3$)

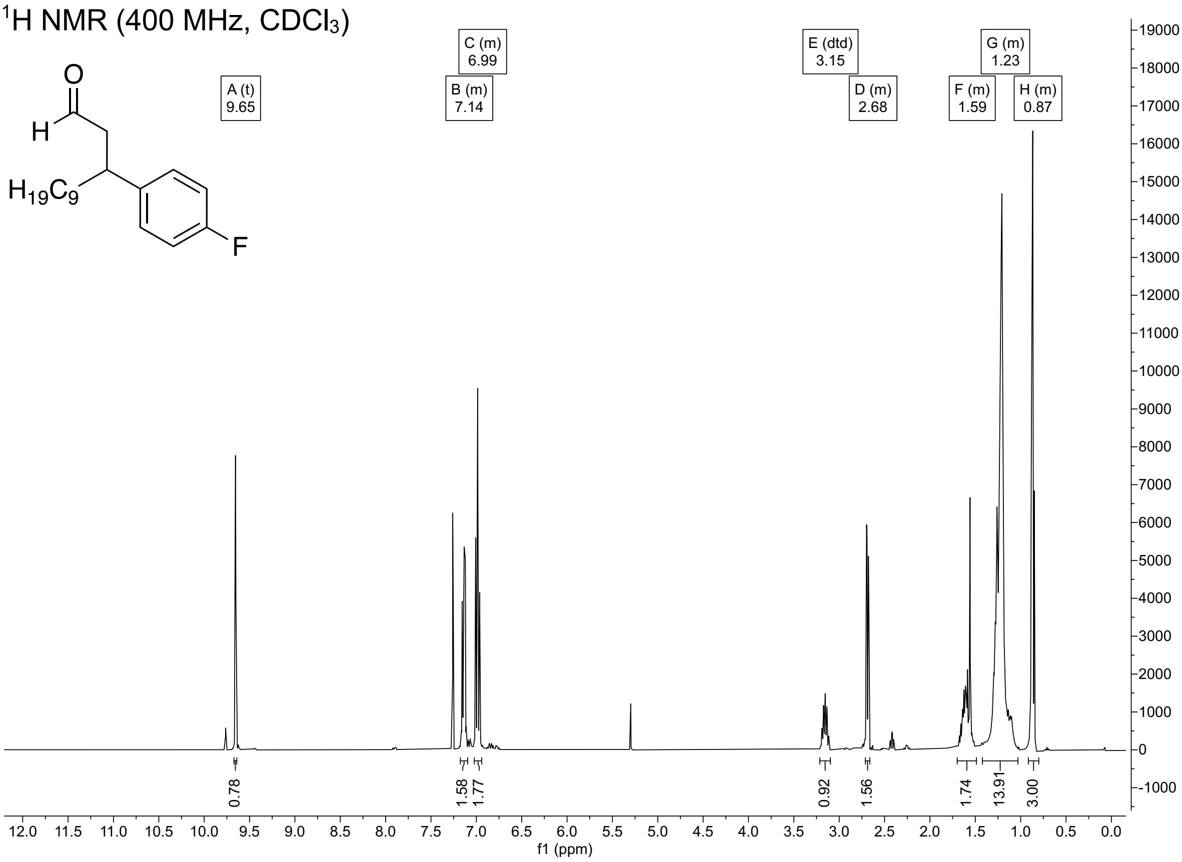

^{13}C NMR (101 MHz, $CDCl_3$)

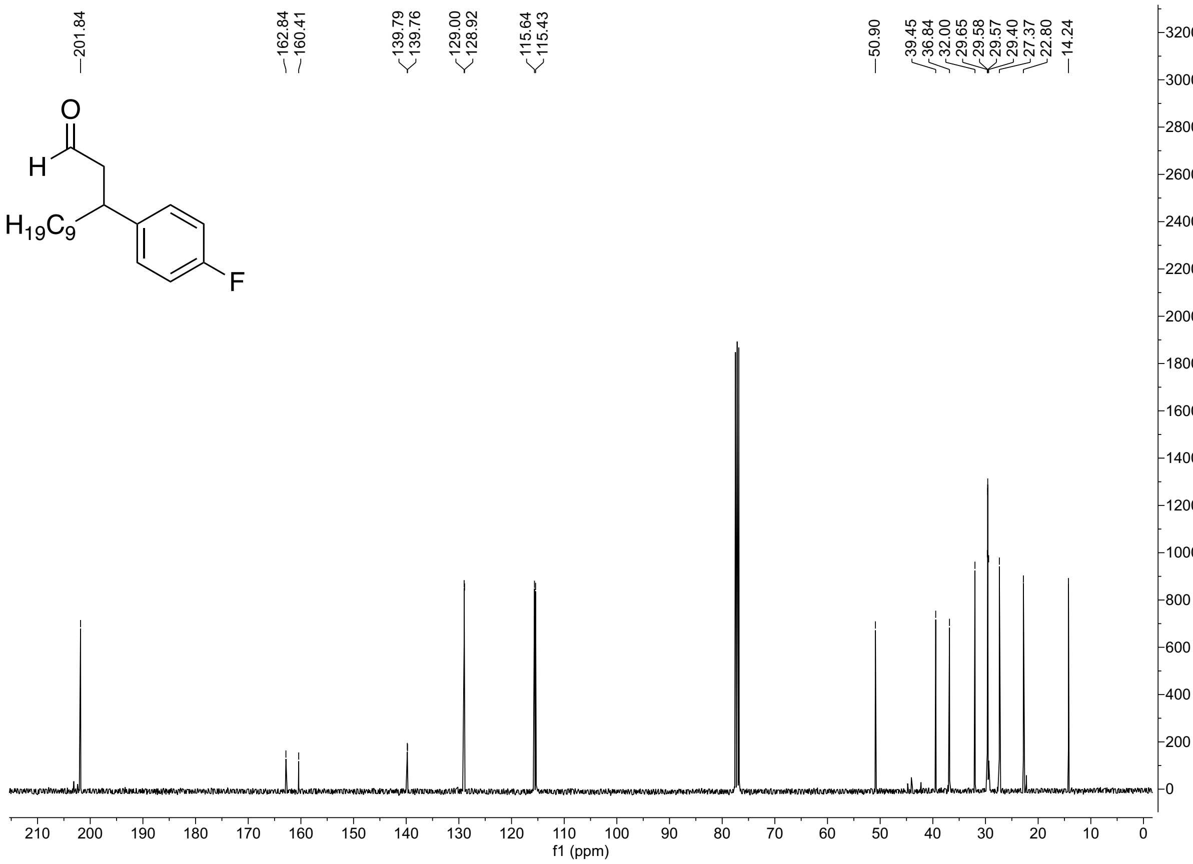

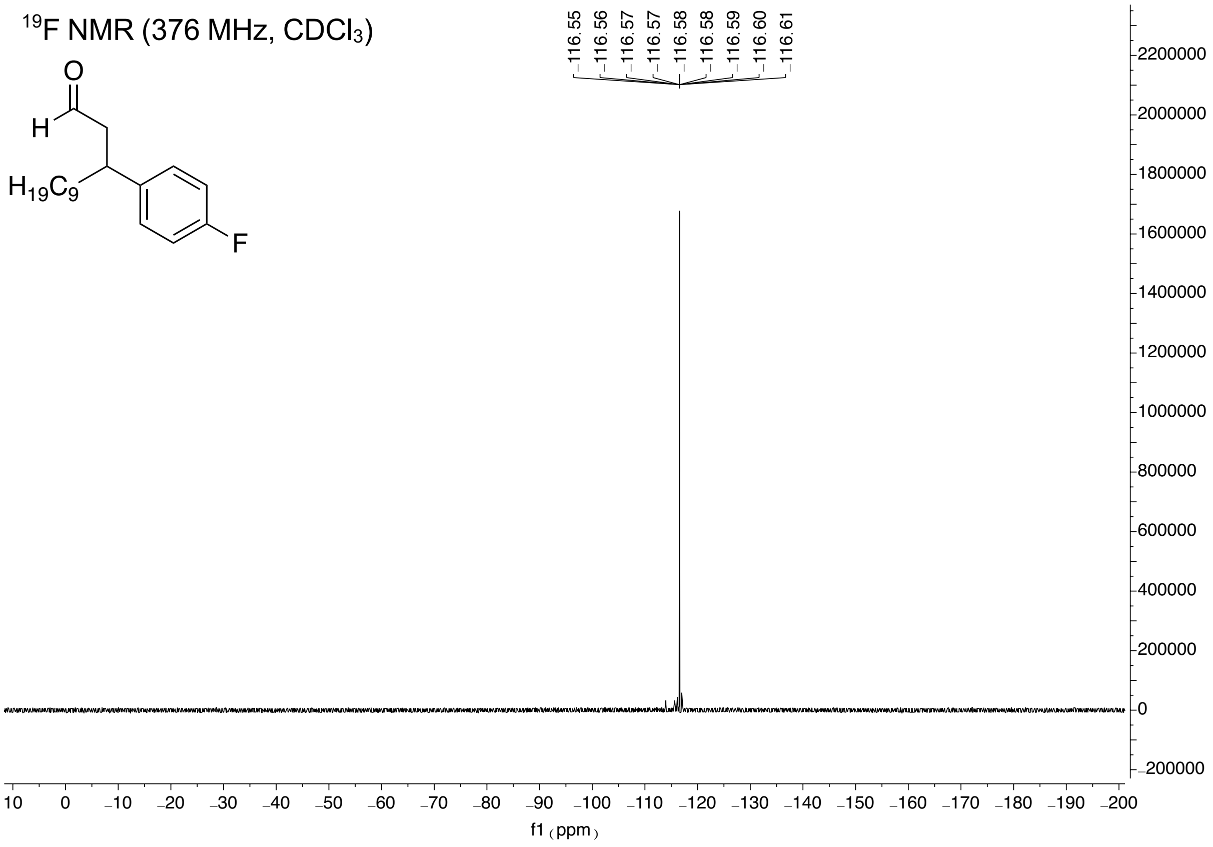

19F NMR (376 MHz, CDCl3)
-116.55
-116.56
-116.57
-116.57
-116.58
-116.58
-116.59
-116.60
-116.61
H19C9
F
f1 (ppm)

3-(4-Fluorophenyl)-4-methylpentanal (1n)

^{1}H NMR (400 MHz, $CDCl_3$)

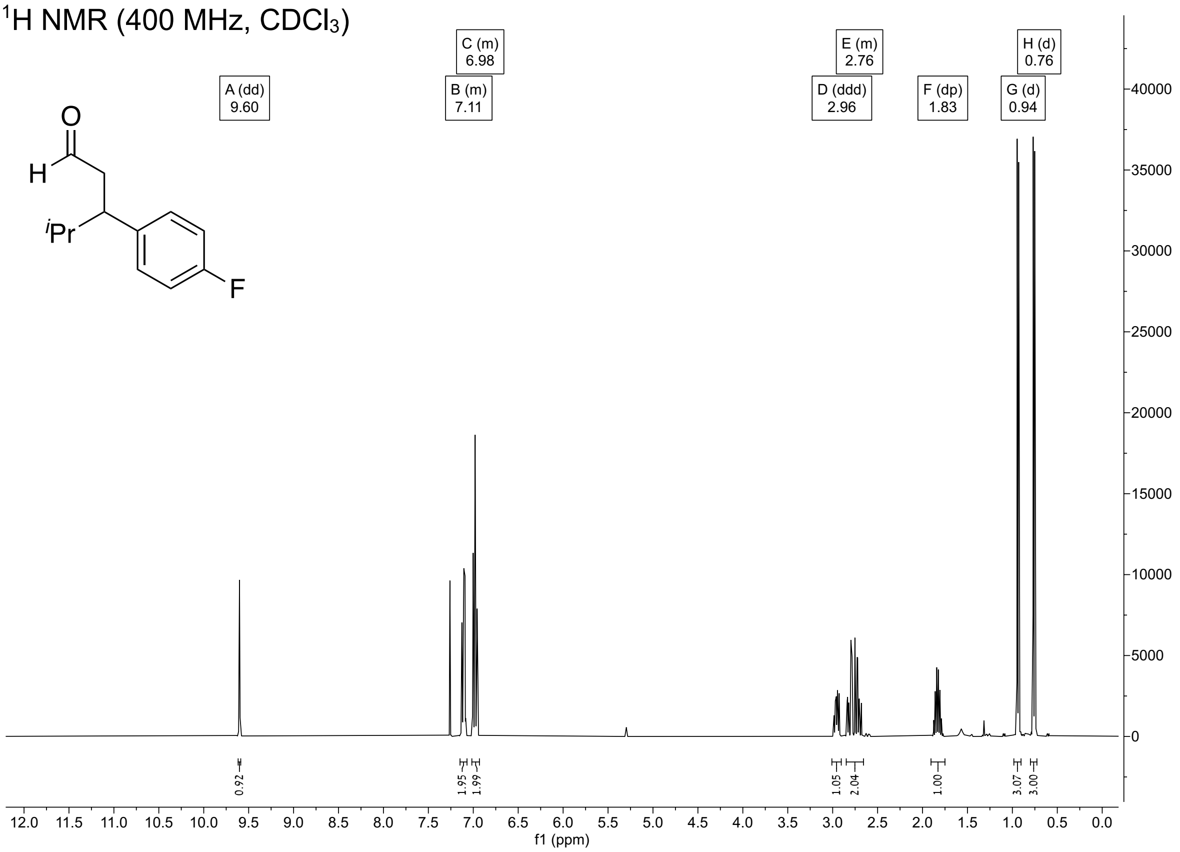

^{13}C NMR (101 MHz, $CDCl_3$)

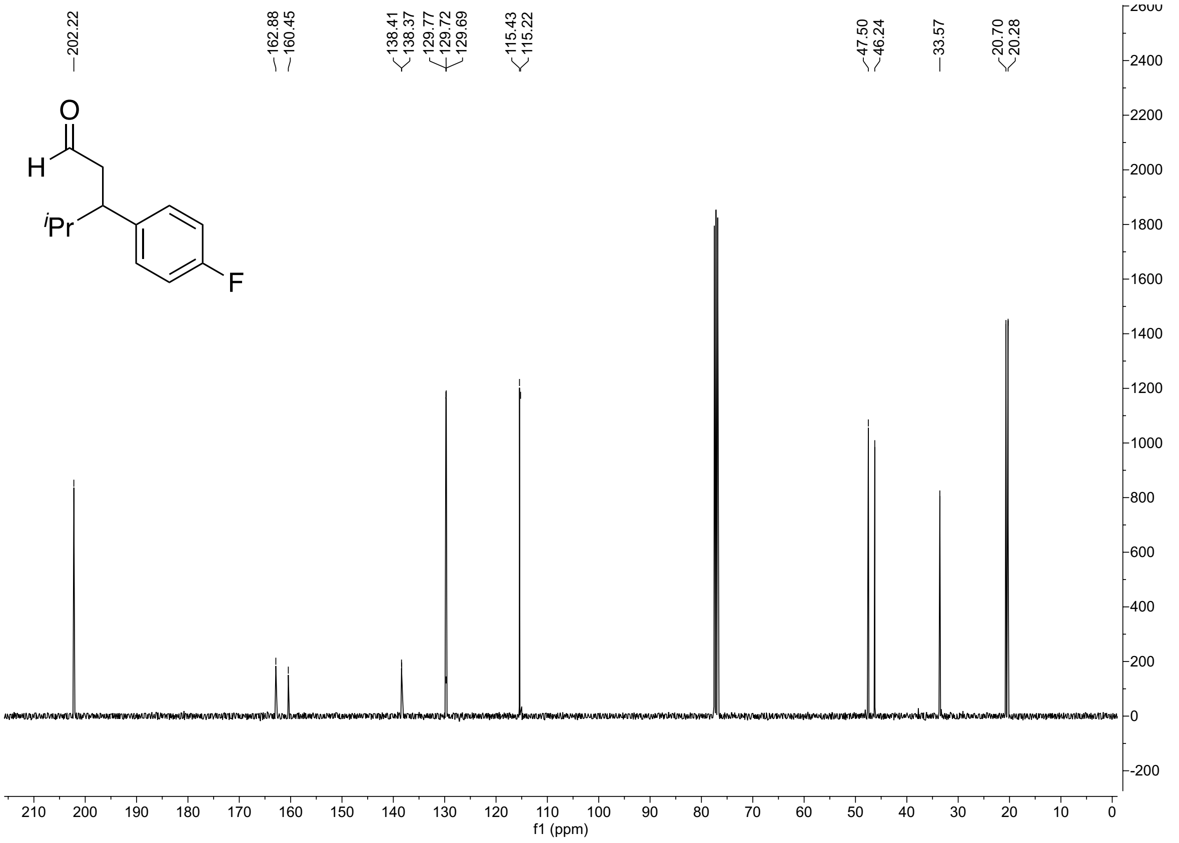

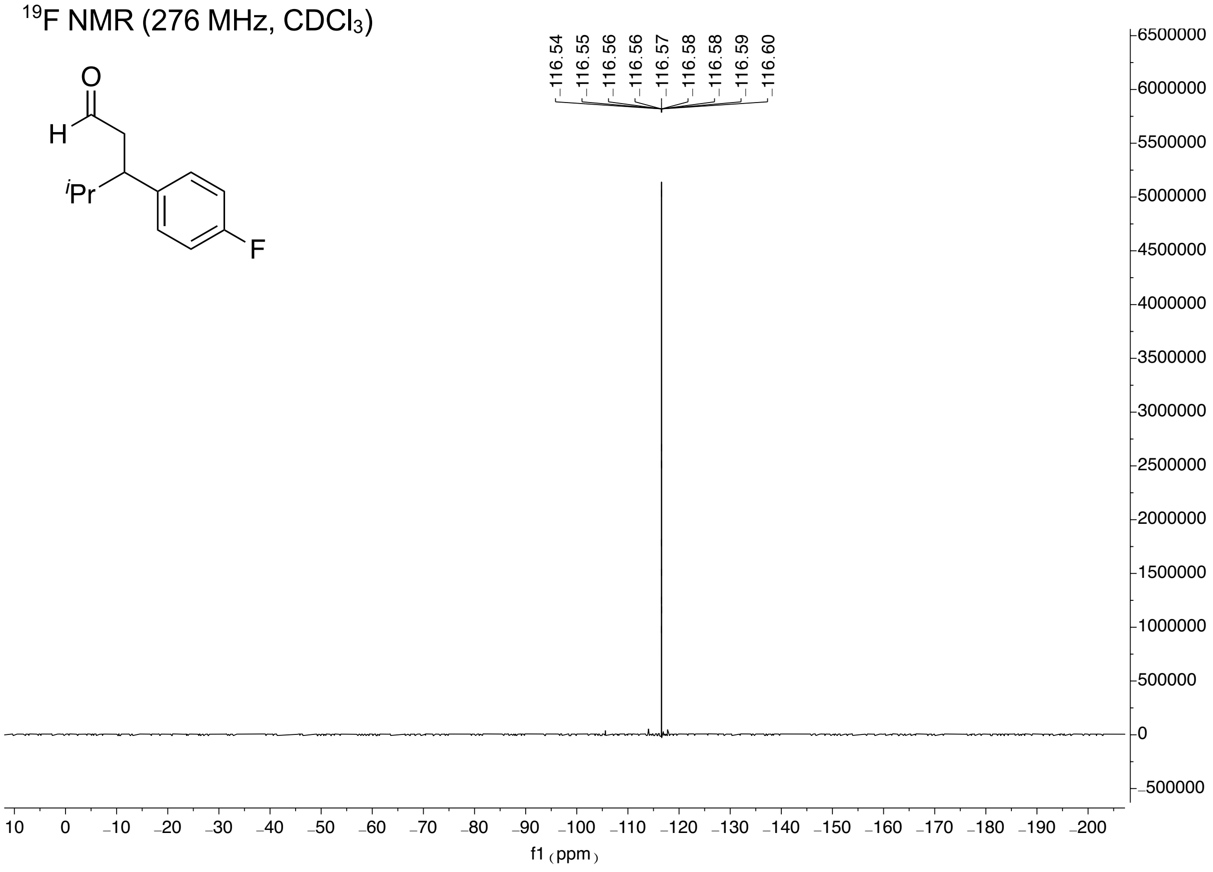

19F NMR (276 MHz, CDCl3)
O
H
iPr
F
-116.54
-116.55
-116.56
-116.56
-116.57
-116.58
-116.58
-116.59
-116.60
6500000
6000000
5500000
5000000
4500000
4000000
3500000
3000000
2500000
2000000
1500000
1000000
500000
0
-500000
10 0 -10 -20 -30 -40 -50 -60 -70 -80 -90 -100 -110 -120 -130 -140 -150 -160 -170 -180 -190 -200
f1 (ppm)

Methyl 4-(1-oxopentan-3-yl)benzoate (1o)

^{1}H NMR (400 MHz, $CDCl_3$)

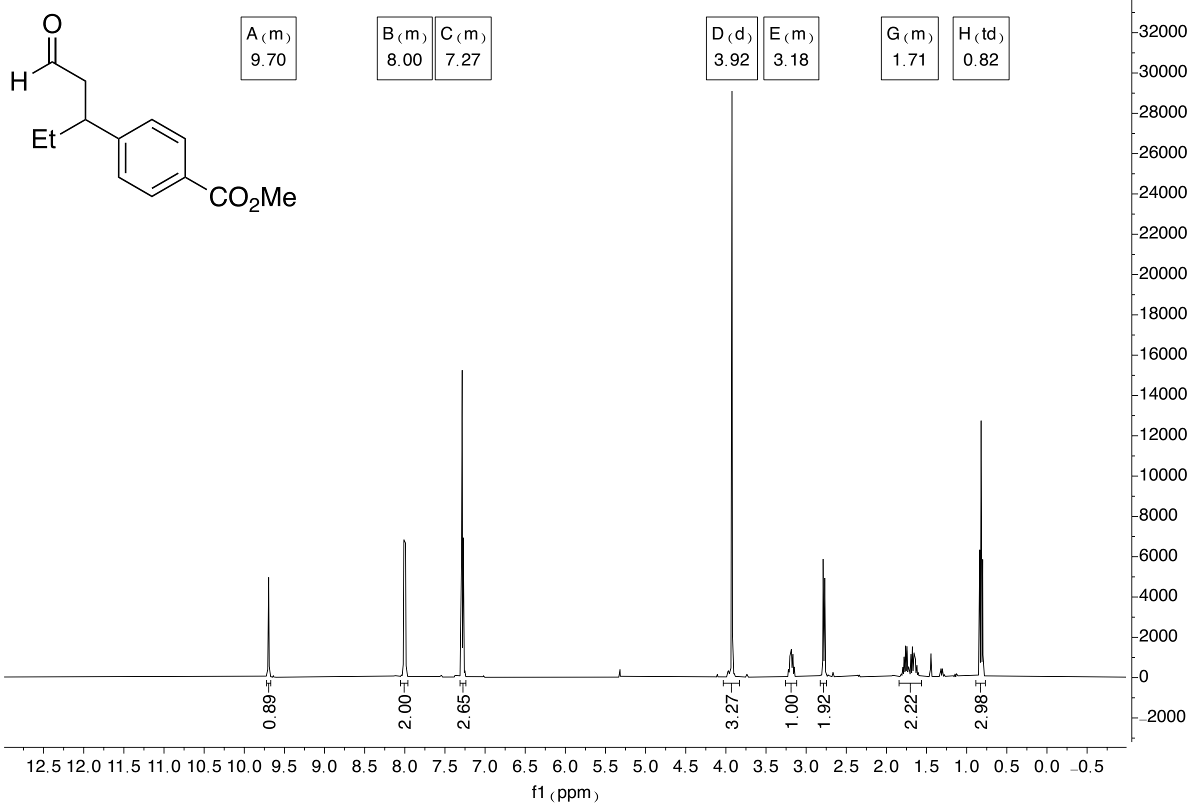

^{13}C NMR (101 MHz, $CDCl_3$)

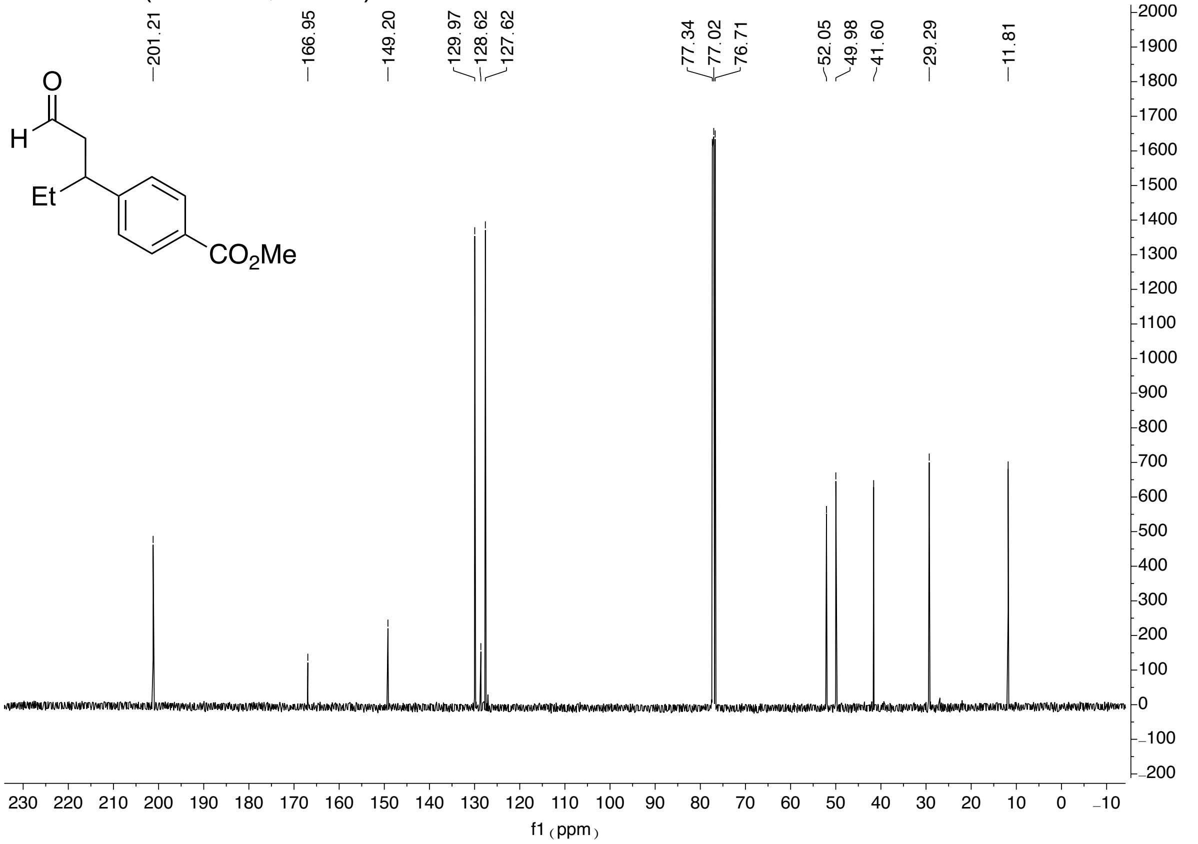

9 NMR Spectra of Alcohols

Methyl 4-(4-hydroxybutan-2-yl)benzoate (1f')

^{1}H NMR (400 MHz, $CDCl_3$)

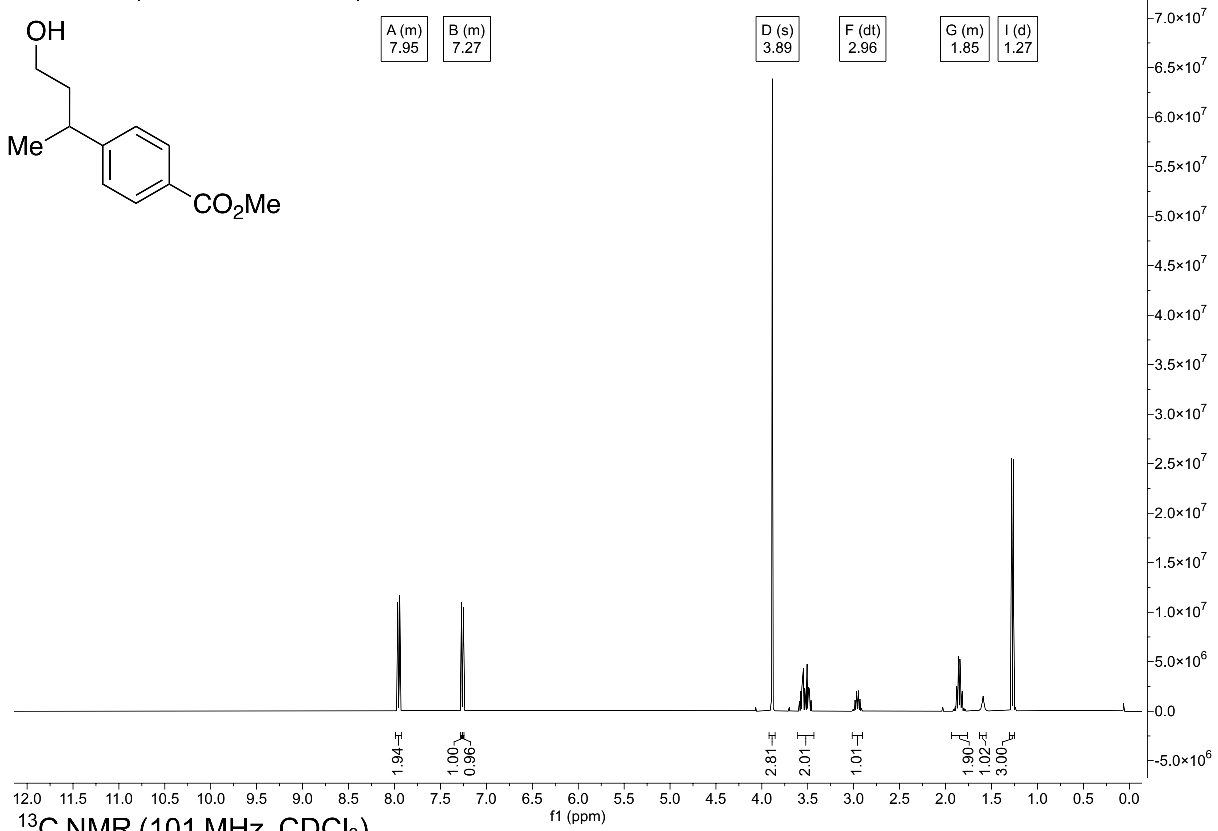

^{13}C NMR (101 MHz, $CDCl_3$)

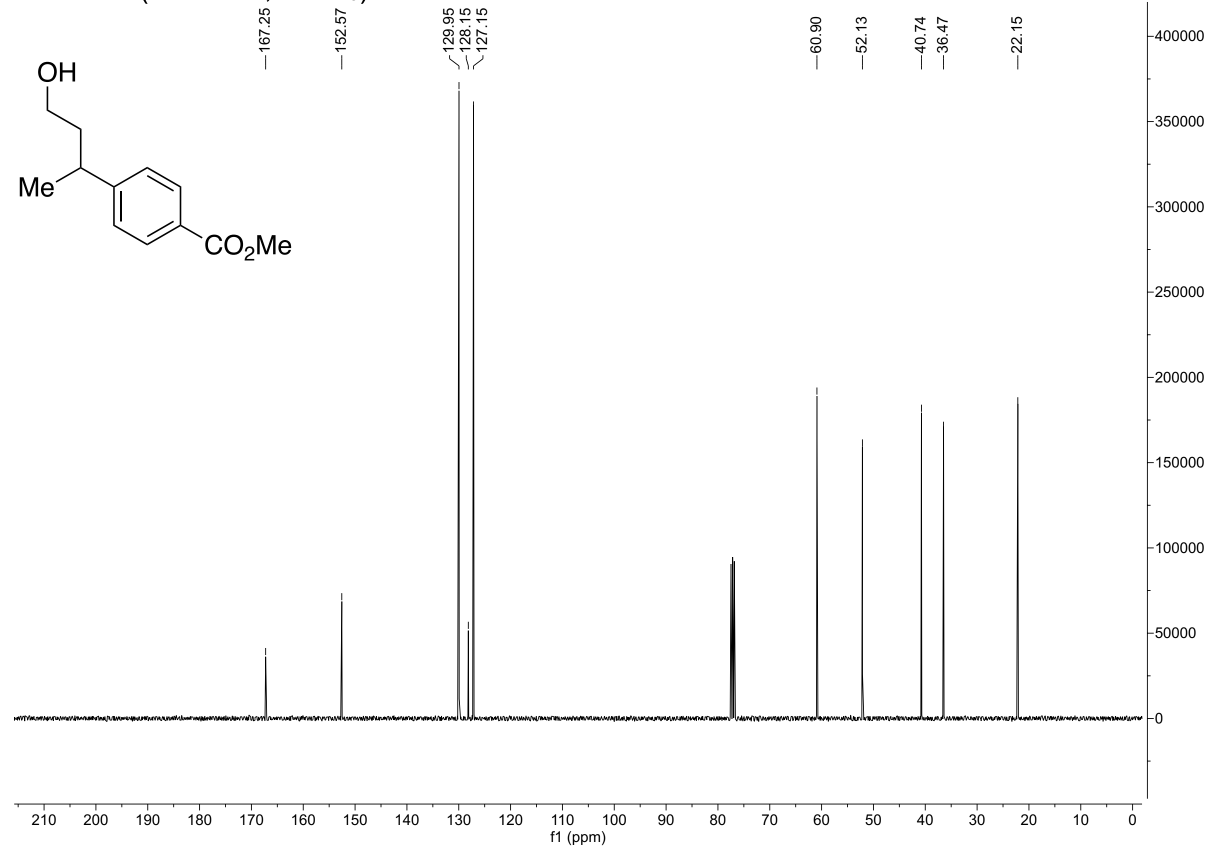

3-(5-Methylfuran-2-yl)butan-1-ol (1j’)

^{1}H NMR (400 MHz, $CDCl_3$)

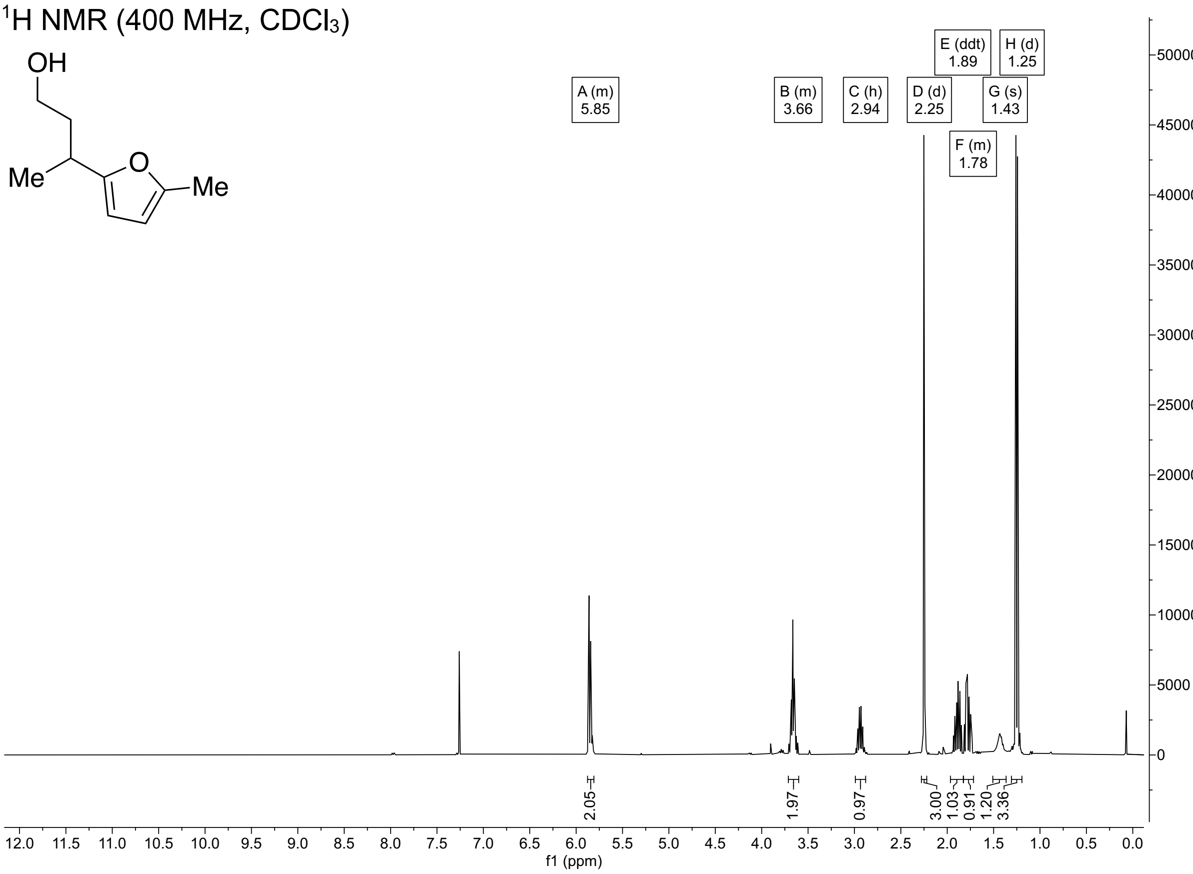

^{13}C NMR (101 MHz, $CDCl_3$)

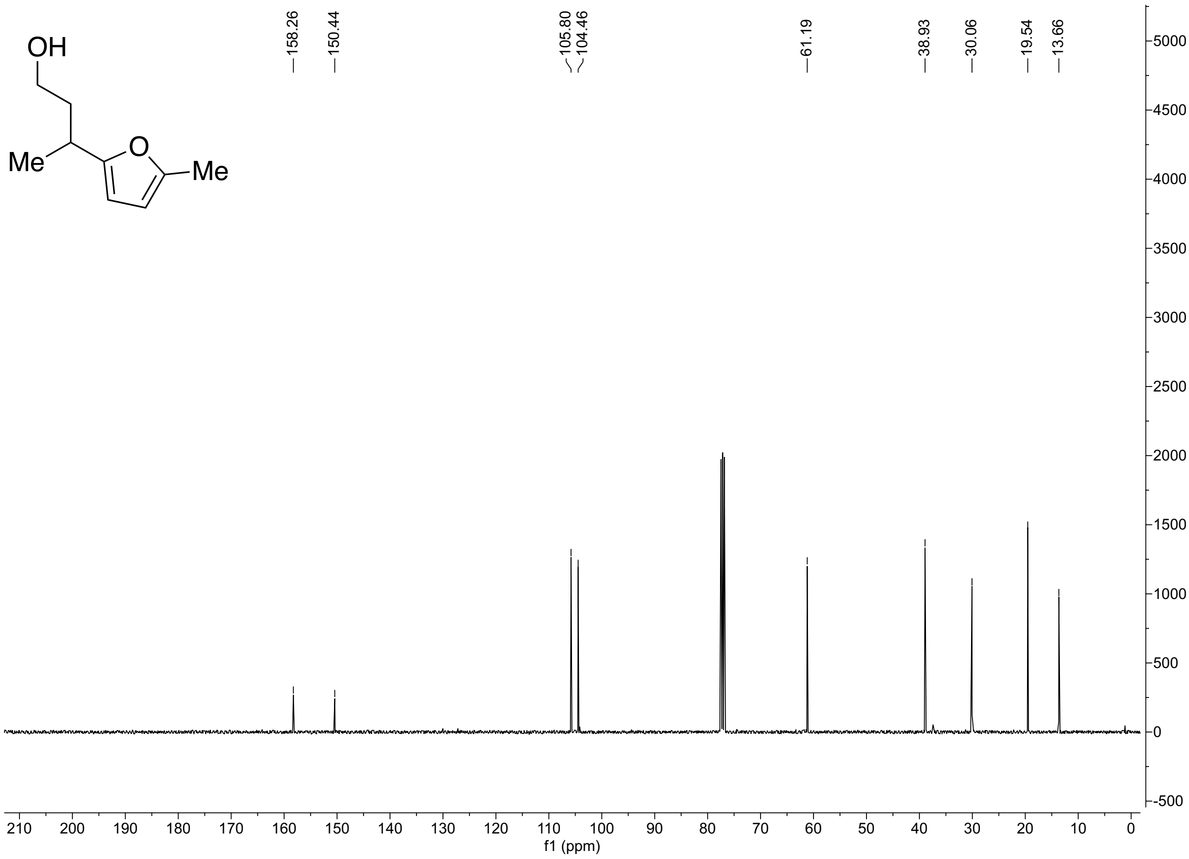

3-(4-Fluorophenyl)hexan-1-ol (1l')

^{1}H NMR (400 MHz, $CDCl_3$)

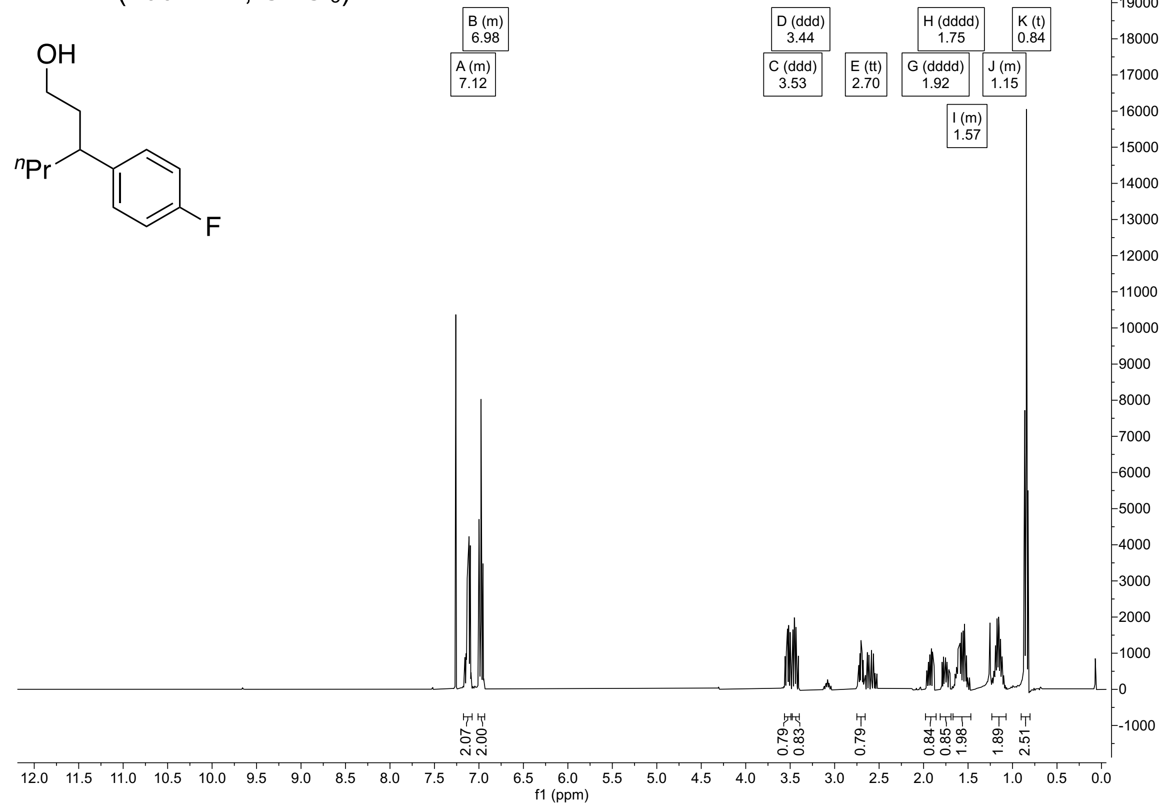

^{13}C NMR (101 MHz, $CDCl_3$)

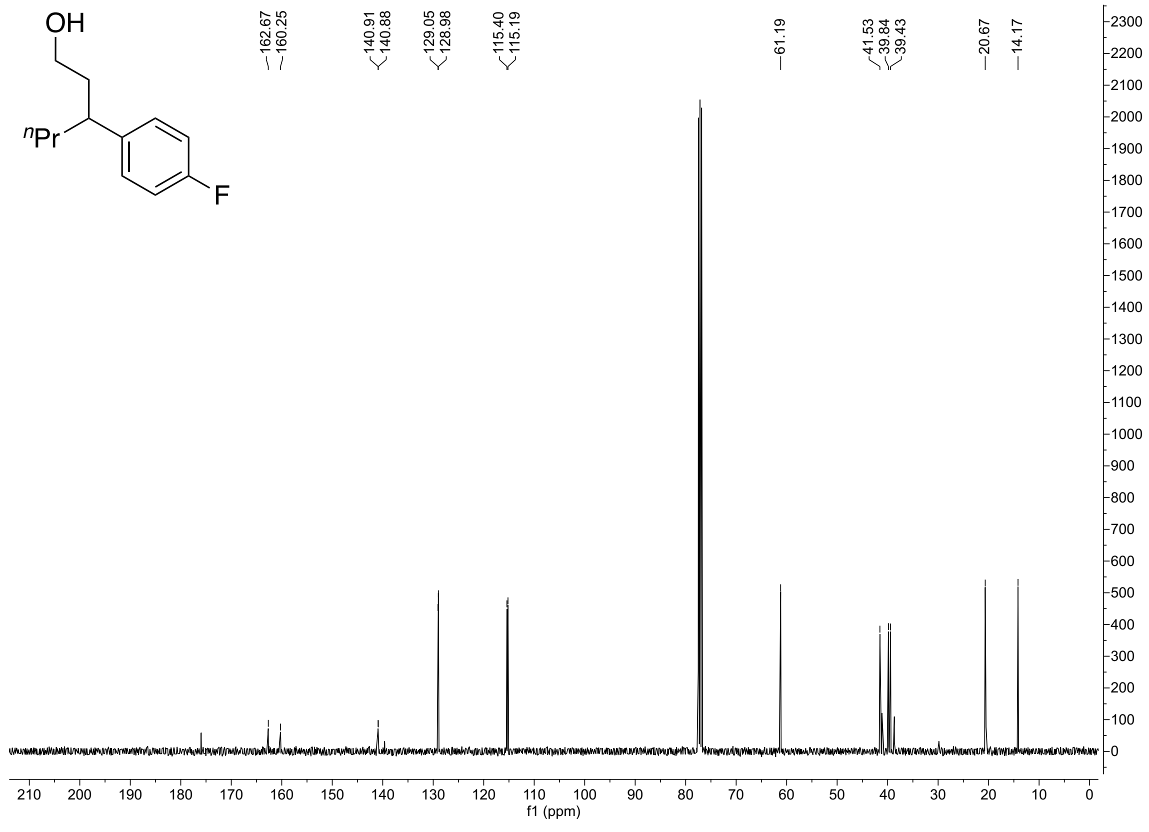

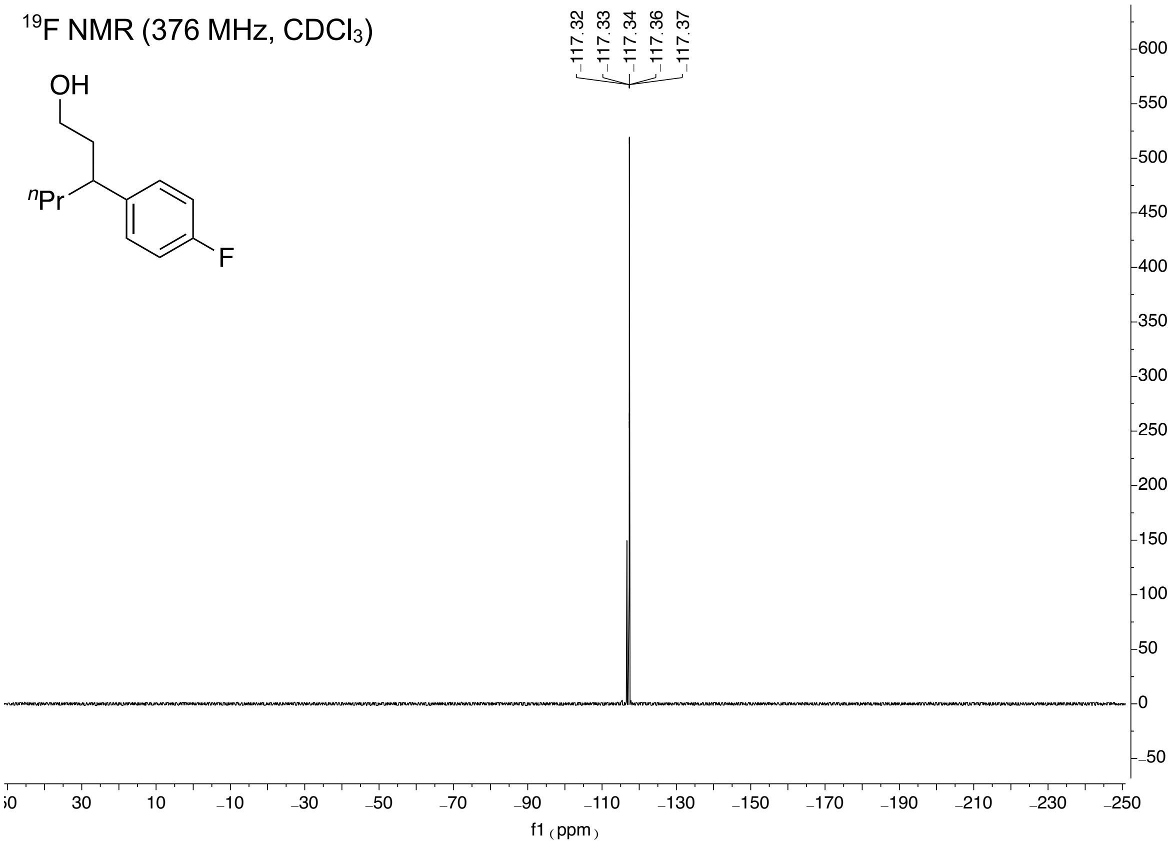

^{19}F NMR (376 MHz, CDCl$_3$)
OH
^{n}Pr
F
-117.32
-117.33
-117.34
-117.36
-117.37
f1 (ppm)

3-(4-Fluorophenyl)dodecan-1-ol (1m’)

^{1}H NMR (400 MHz, $CDCl_3$)

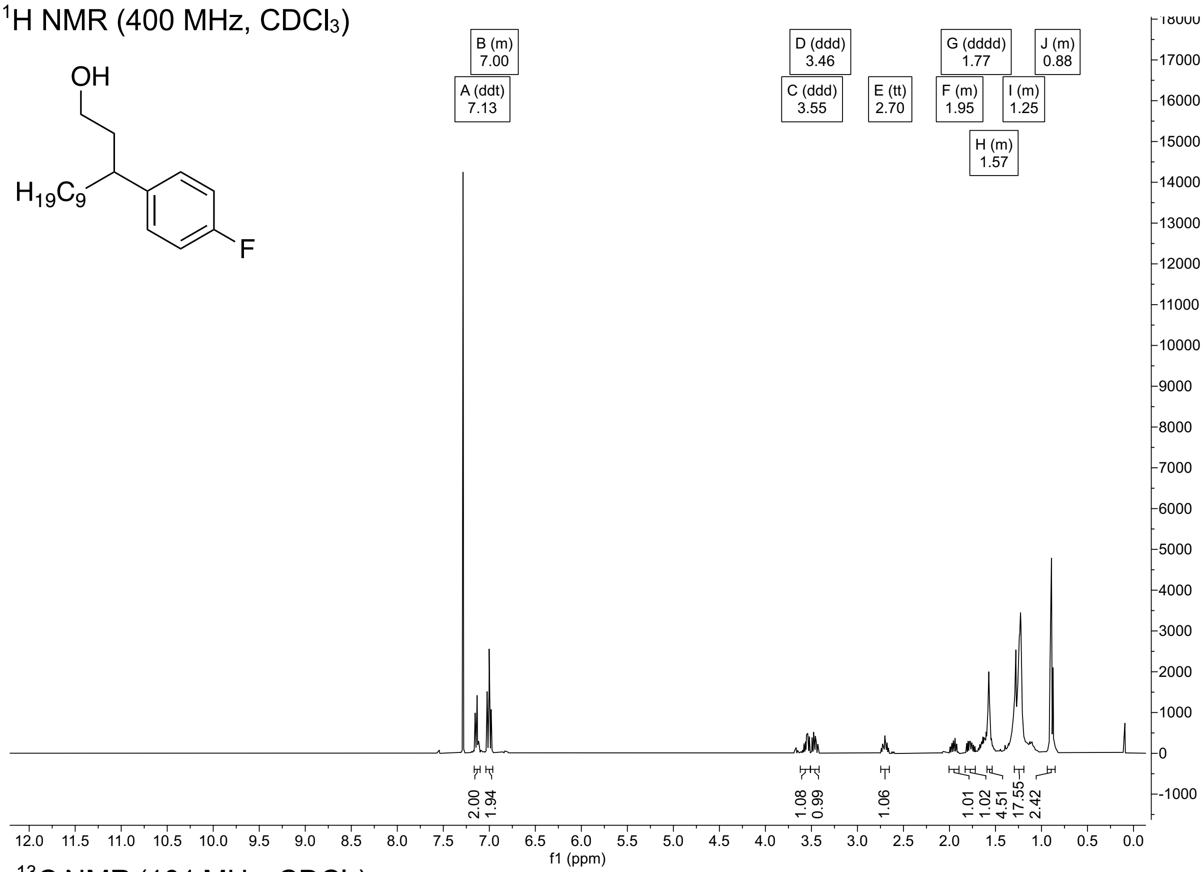

^{13}C NMR (101 MHz, $CDCl_3$)

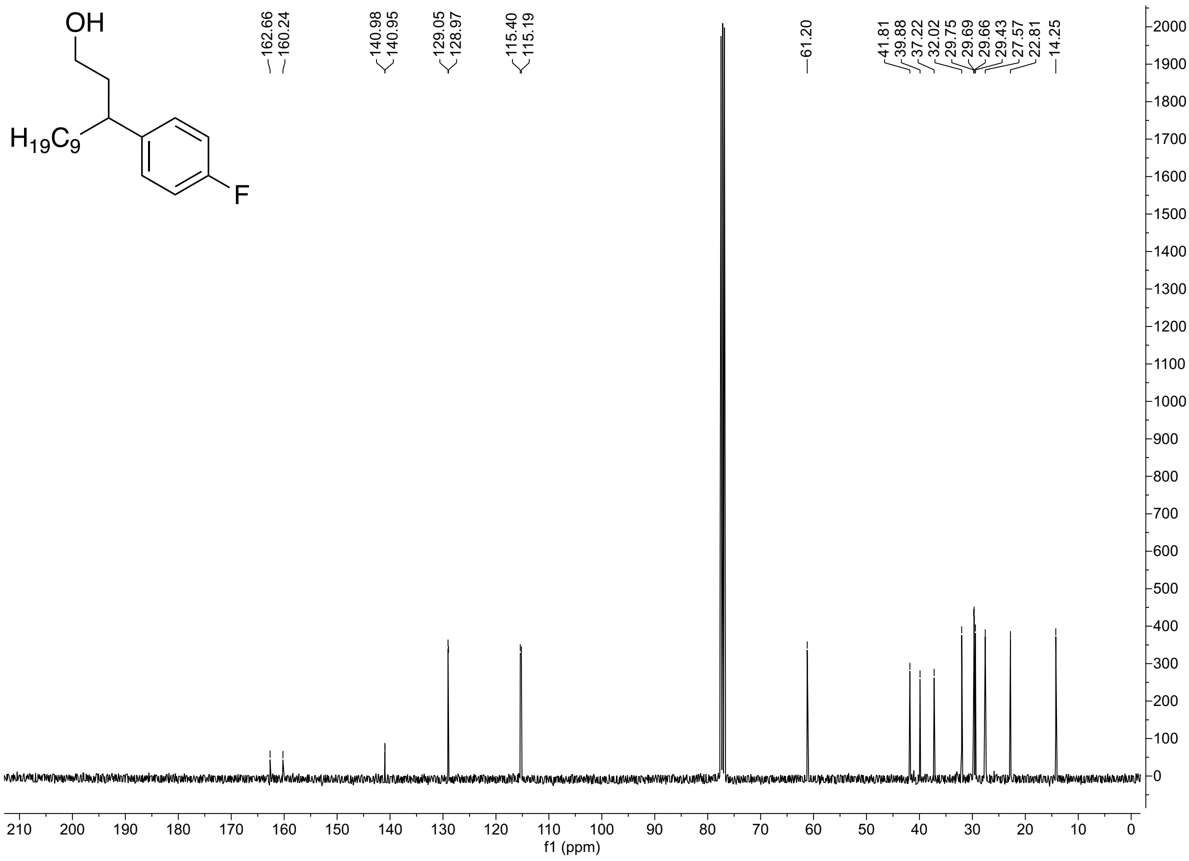

^{19}F NMR (376 MHz, $CDCl_3$)

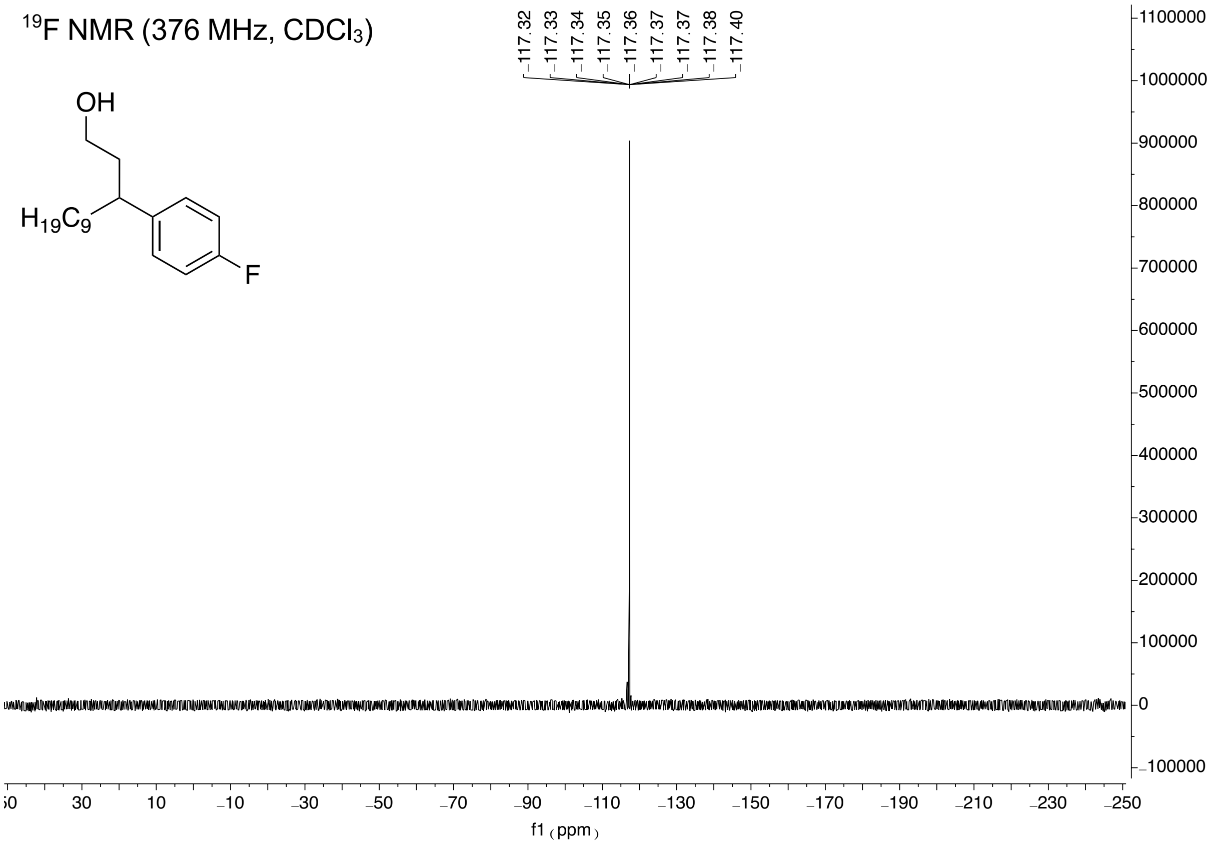

3-(4-Fluorophenyl)-4-methylpentan-1-ol (1n’)

^{1}H NMR (500 MHz, $CDCl_3$)

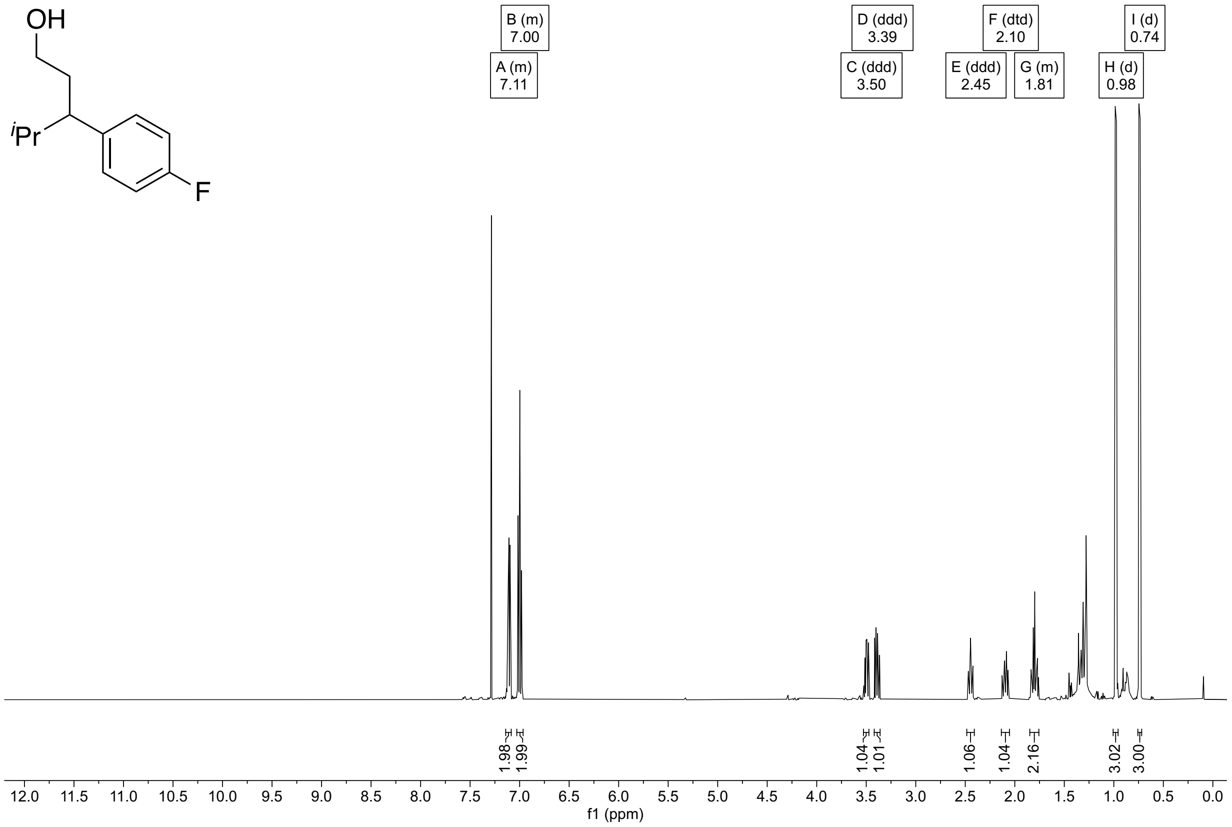

^{13}C NMR (126 MHz, $CDCl_3$)

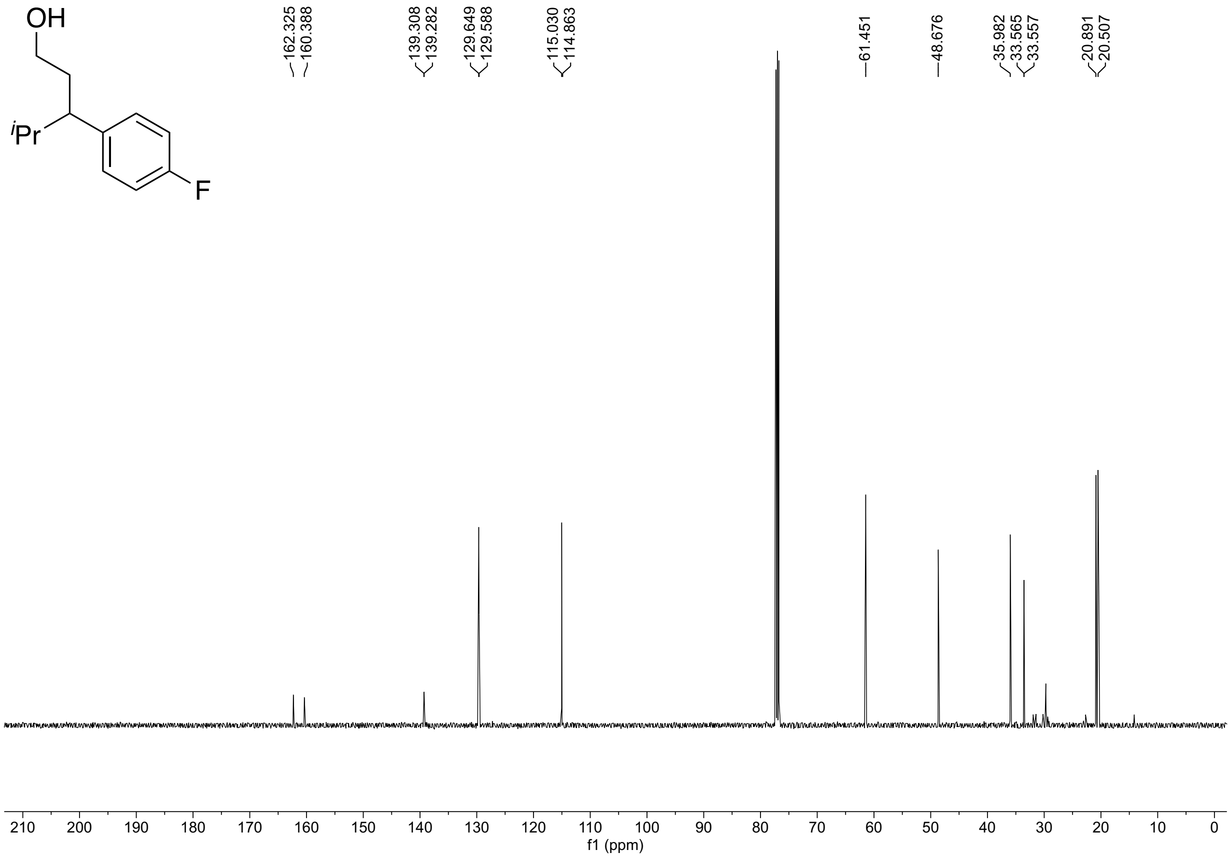

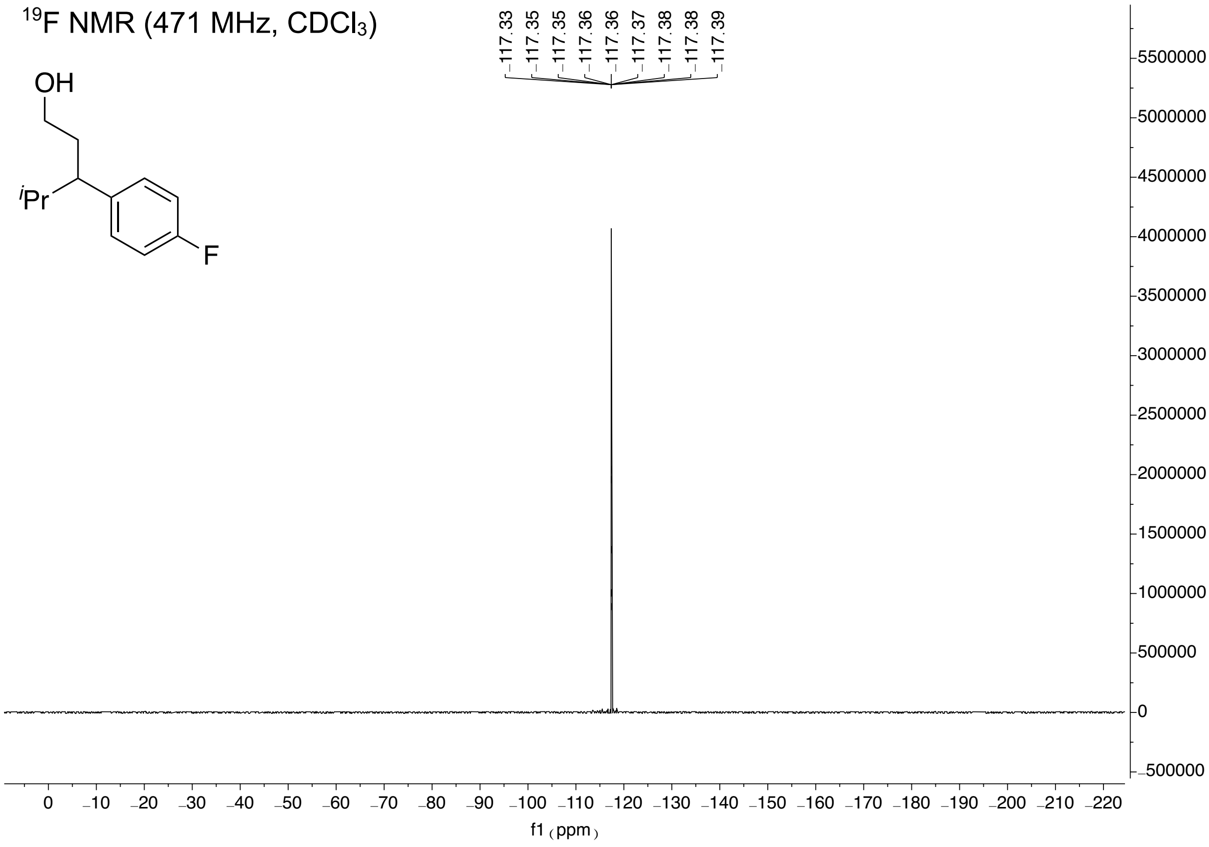

^{19}F NMR (471 MHz, $CDCl_3$)
OH
^{i}Pr
F
-117.33
-117.35
-117.35
-117.36
-117.36
-117.37
-117.38
-117.38
-117.39
f1 (ppm)

10 ^{1}H and ^{13}C NMR Spectra of γ-Nitroaldehydes

(2*S*,3*R*)-4-Nitro-3-phenyl-2-((*S*)-1-phenylethyl)butanal (3aa)

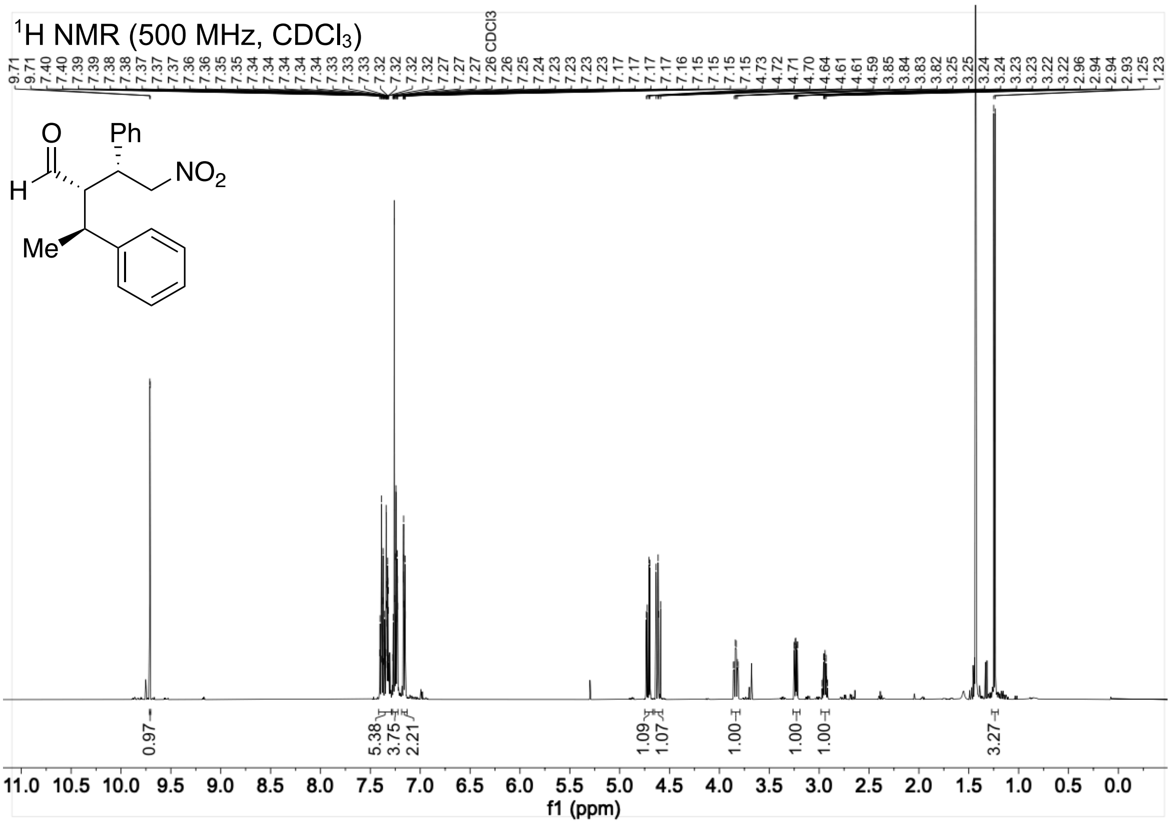

^{13}C NMR (126 MHz, $CDCl_3$)

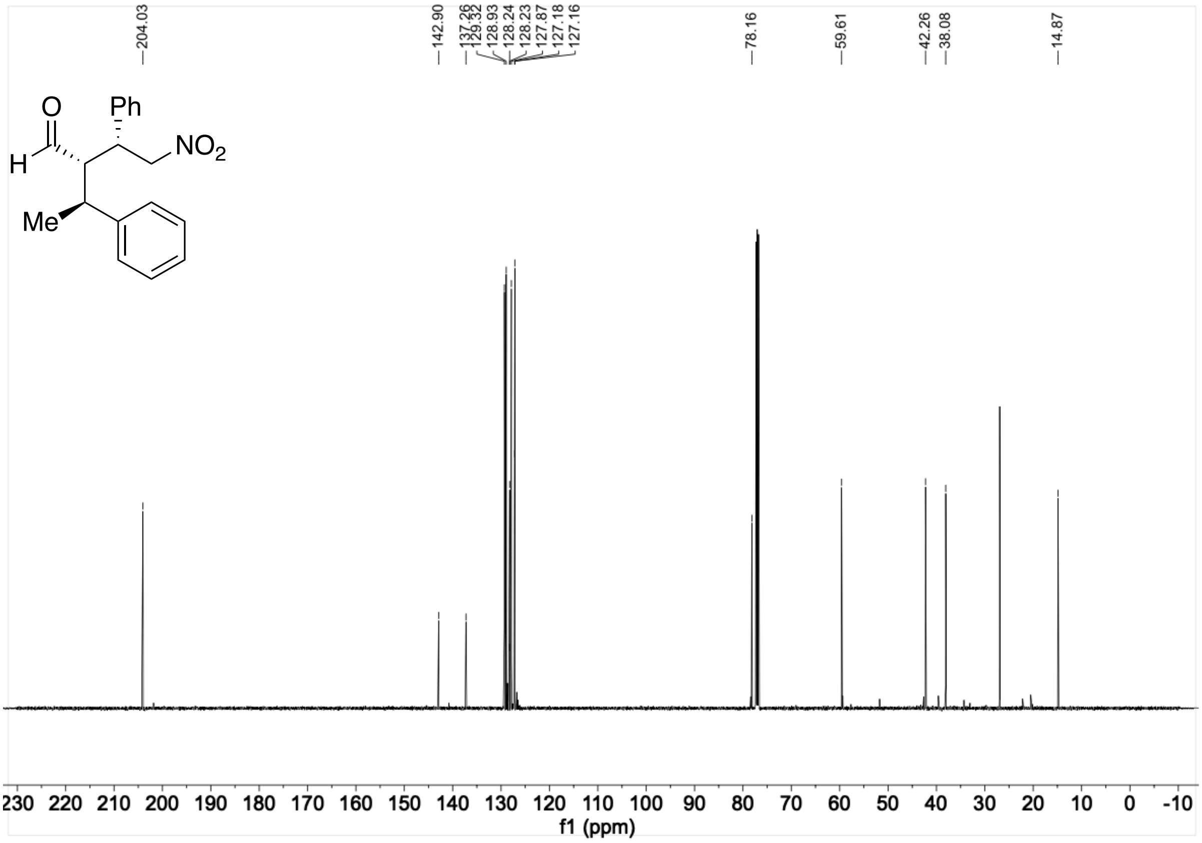

(2*S*,3*R*)-4-Nitro-3-phenyl-2-((*S*)-1-(*p*-tolyl)ethyl)butanal (3ba)

^{1}H NMR (500 MHz, $CDCl_3$)

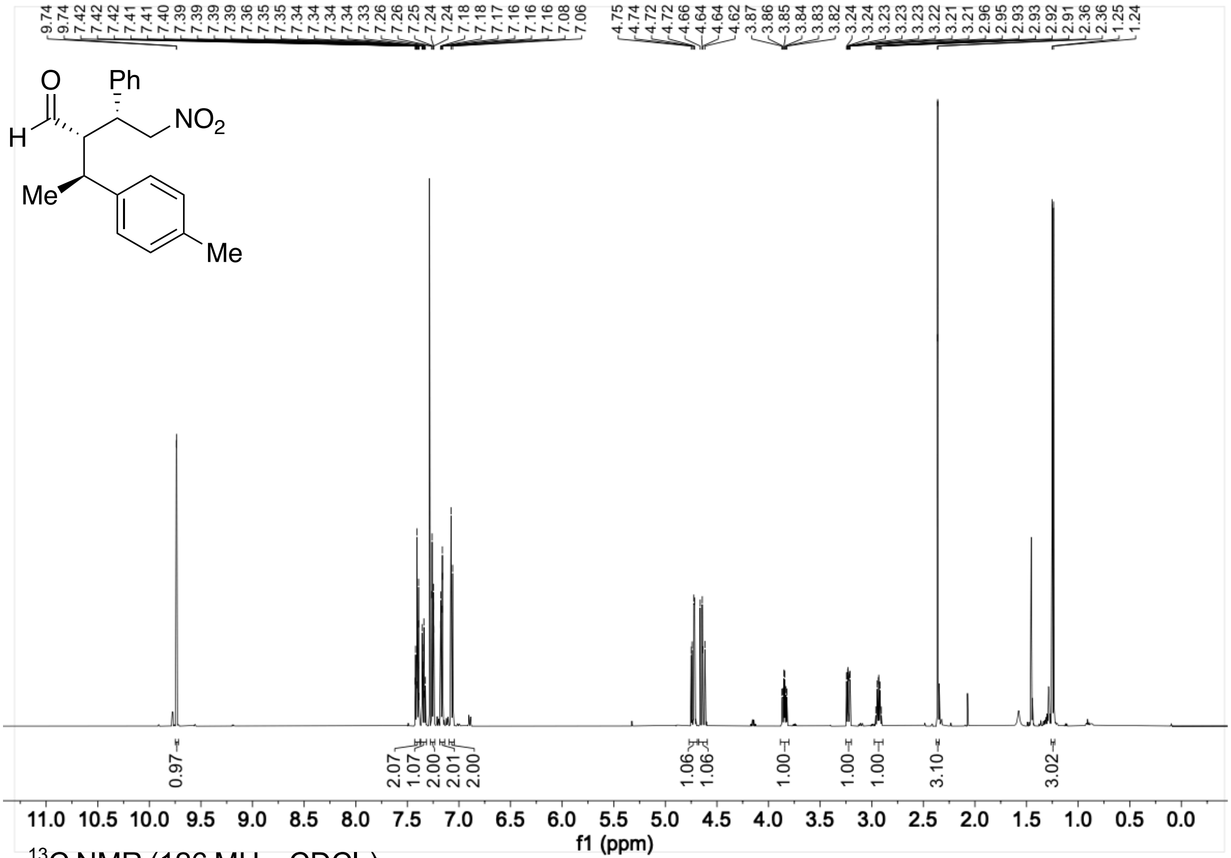

^{13}C NMR (126 MHz, $CDCl_3$)

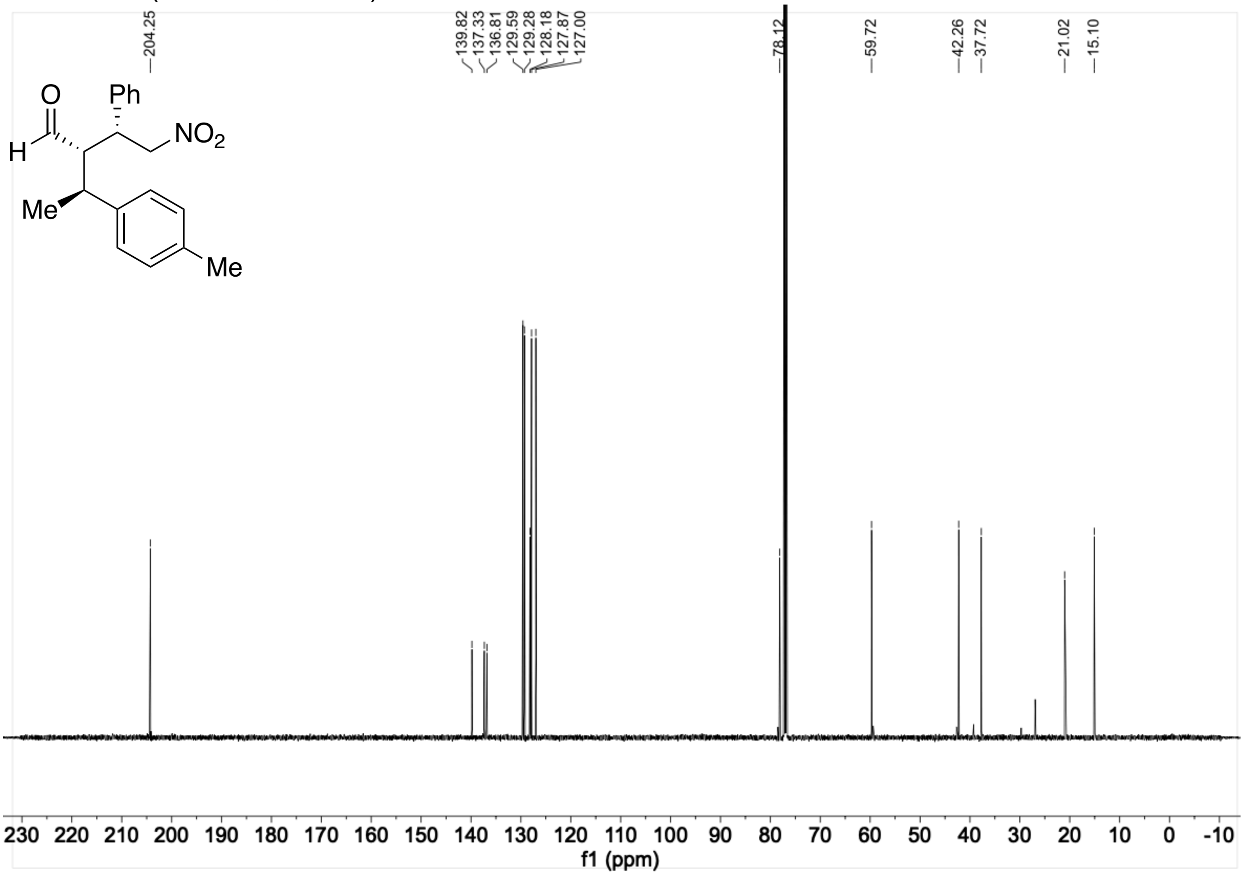

(2*S*,3*R*)-2-((*S*)-1-(4-(*tert*-Butyl)phenyl)ethyl)-4-nitro-3-phenylbutanal (3ca)

^{1}H NMR (400 MHz, $CDCl_3$)

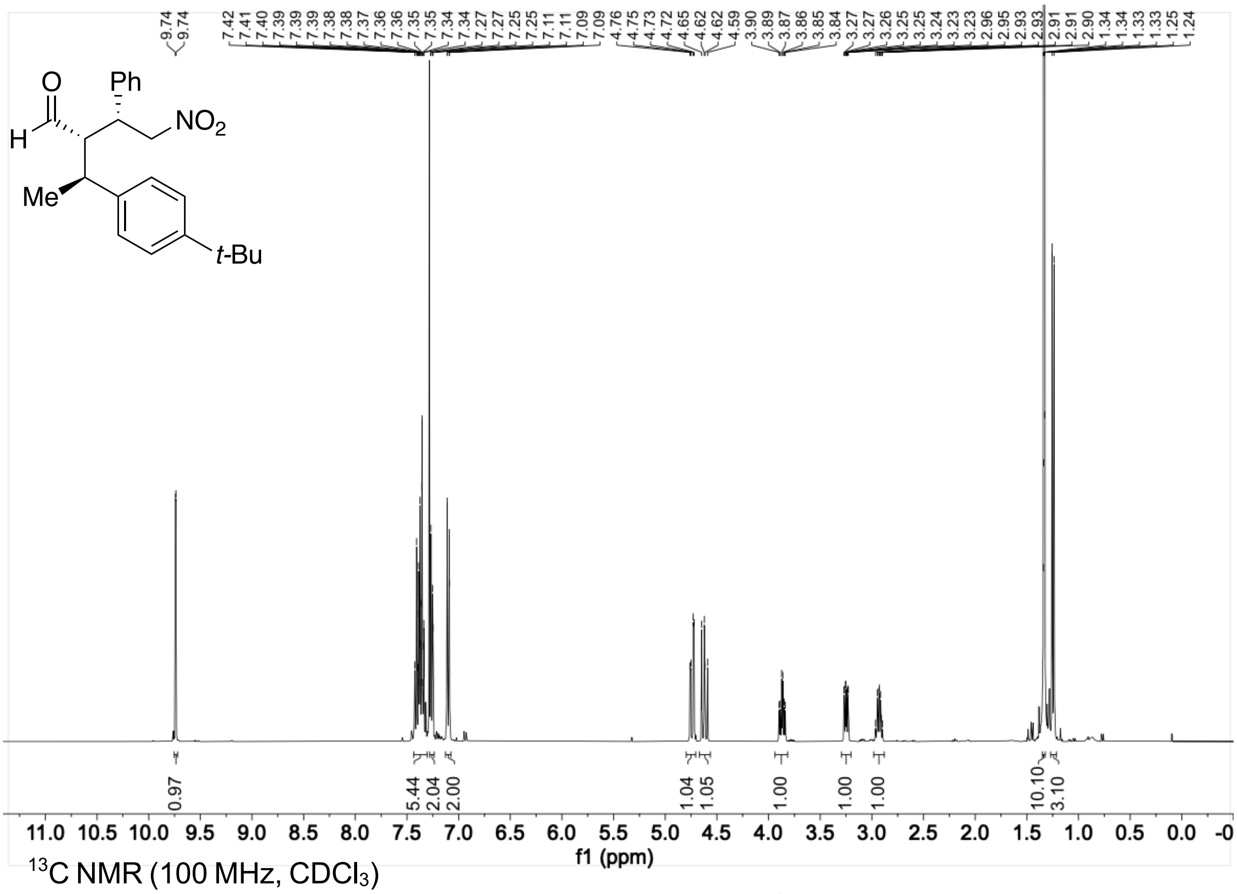

^{13}C NMR (100 MHz, $CDCl_3$)

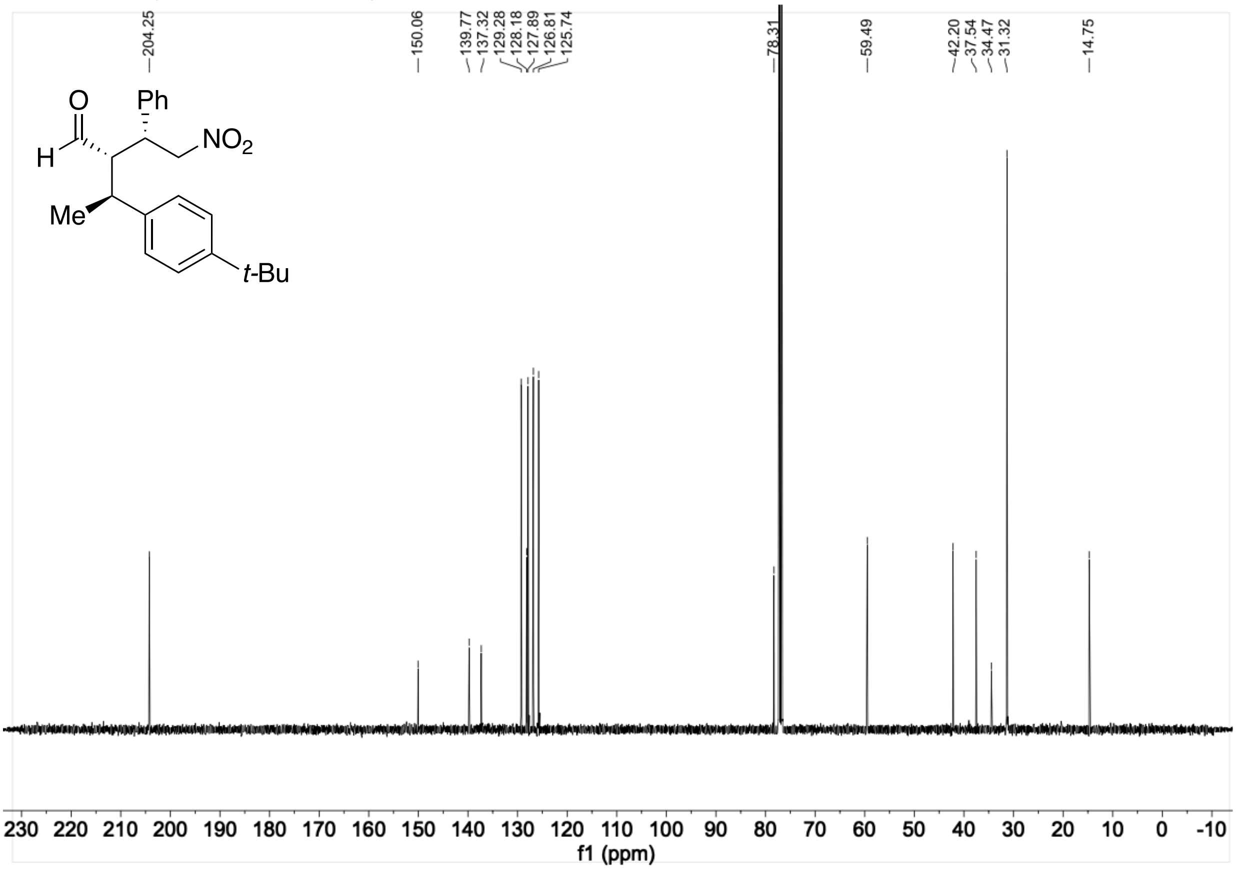

(2*S*,3*R*)-2-((*S*)-1-(4-Methoxyphenyl)ethyl)-4-nitro-3-phenylbutanal (3da)

^{1}H NMR (400 MHz, $CDCl_3$)

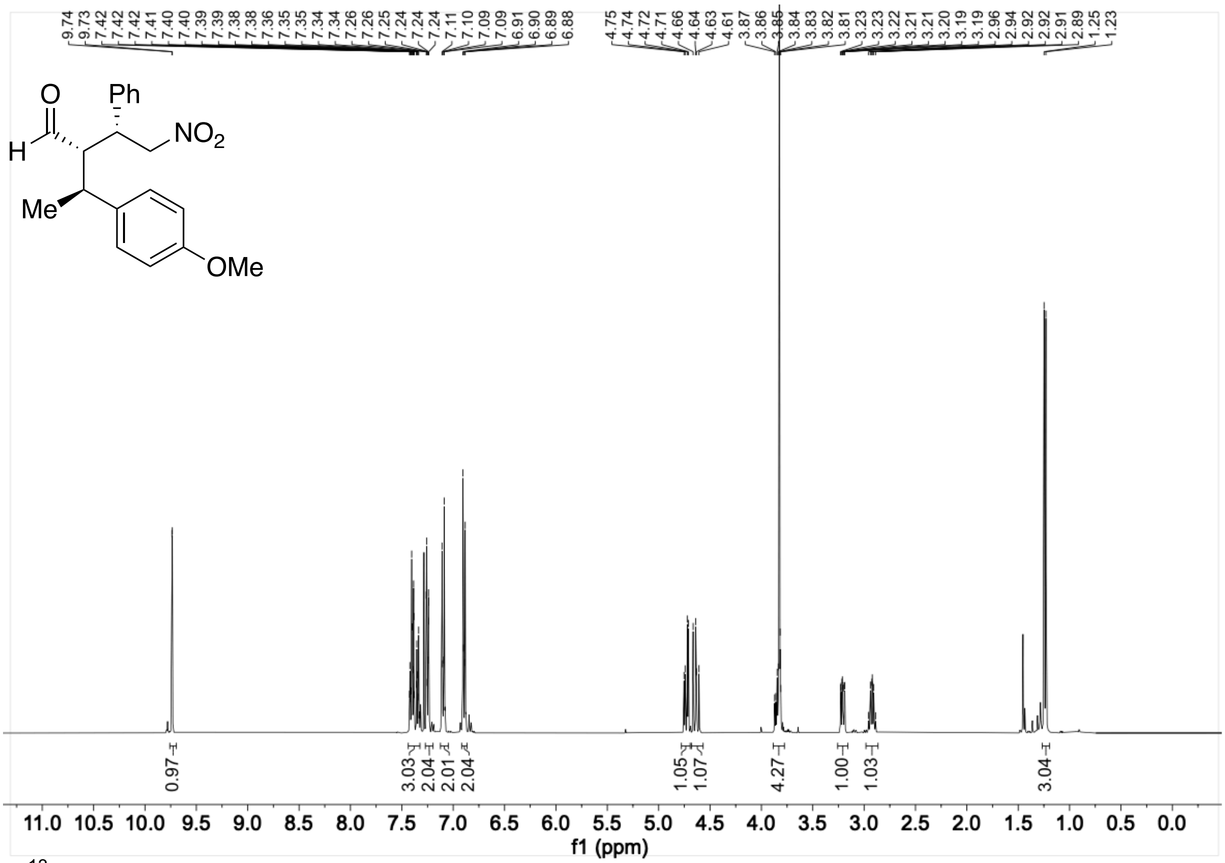

^{13}C NMR (100 MHz, $CDCl_3$)

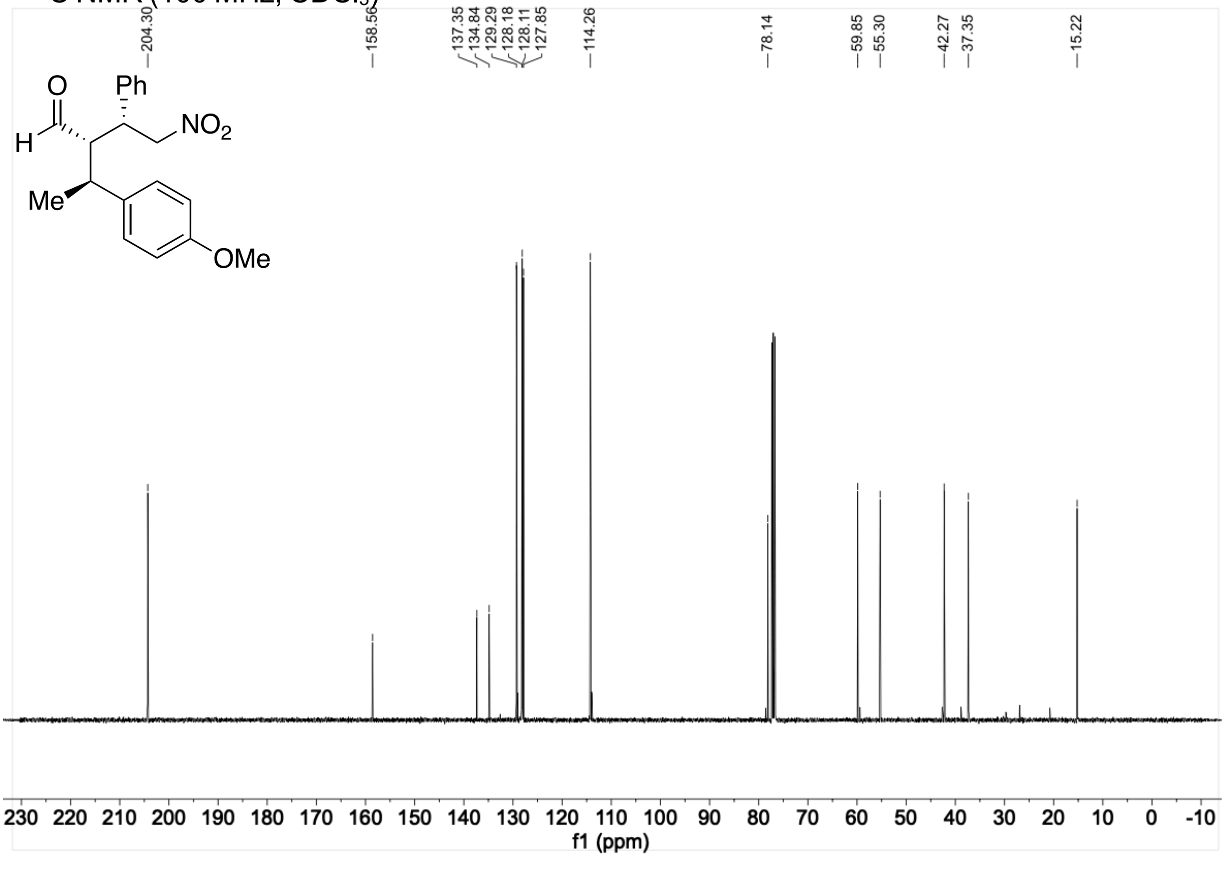

(2*S*,3*R*)-4-Nitro-3-phenyl-2-((*S*)-1-(4-(trifluoromethyl)phenyl)ethyl)butanal (3ea)

^{1}H NMR (400 MHz, $CDCl_3$)

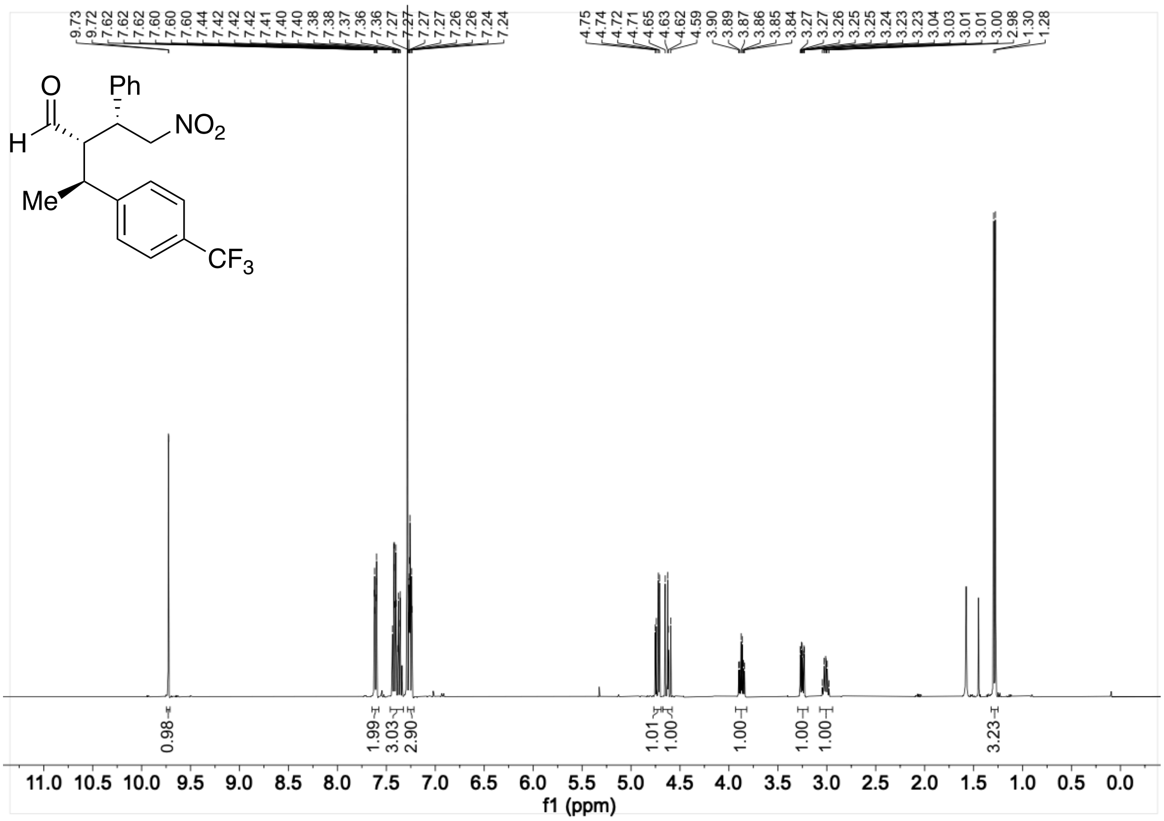

^{13}C NMR (100 MHz, $CDCl_3$)

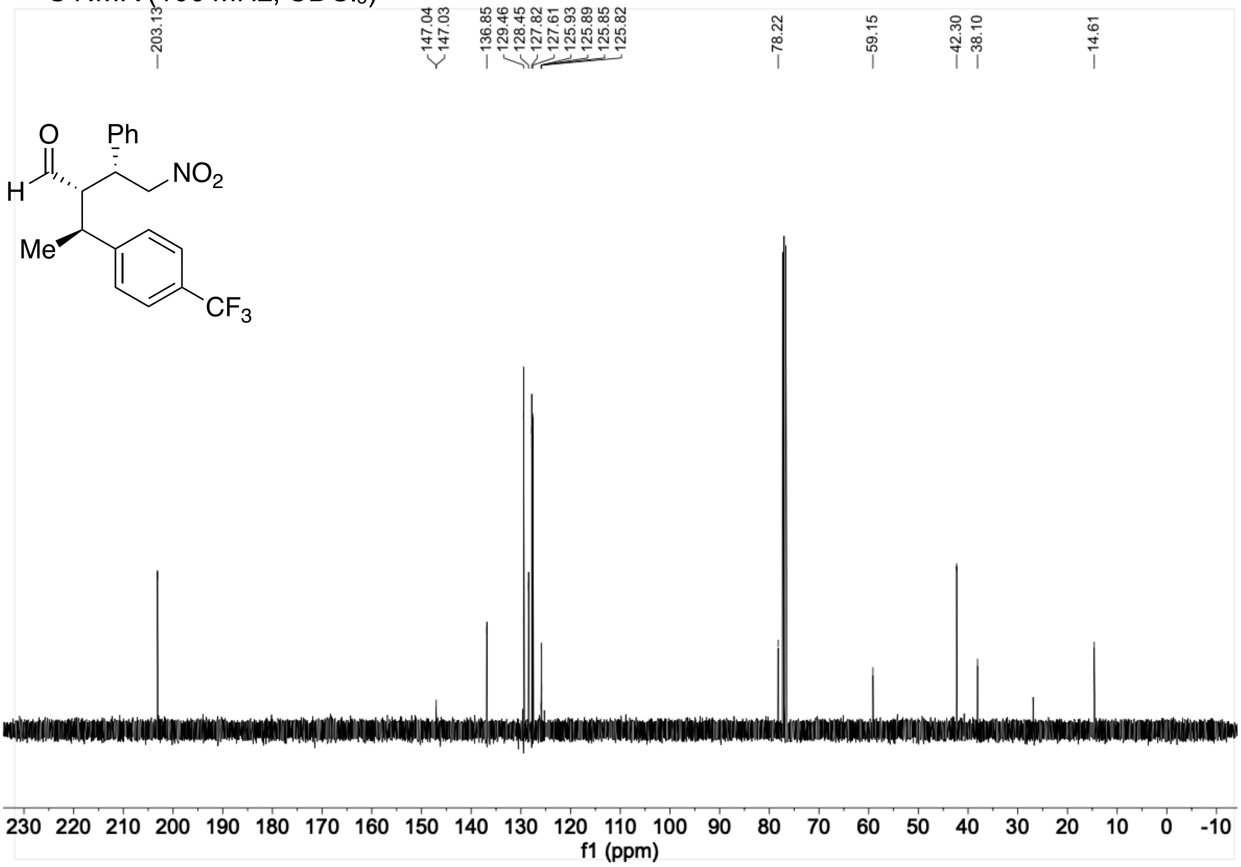

Methyl 4-((2*S*,3*S*,4*R*)-3-formyl-5-nitro-4-phenylpentan-2-yl)benzoate (3fa)

^{1}H NMR (400 MHz, $CDCl_3$)

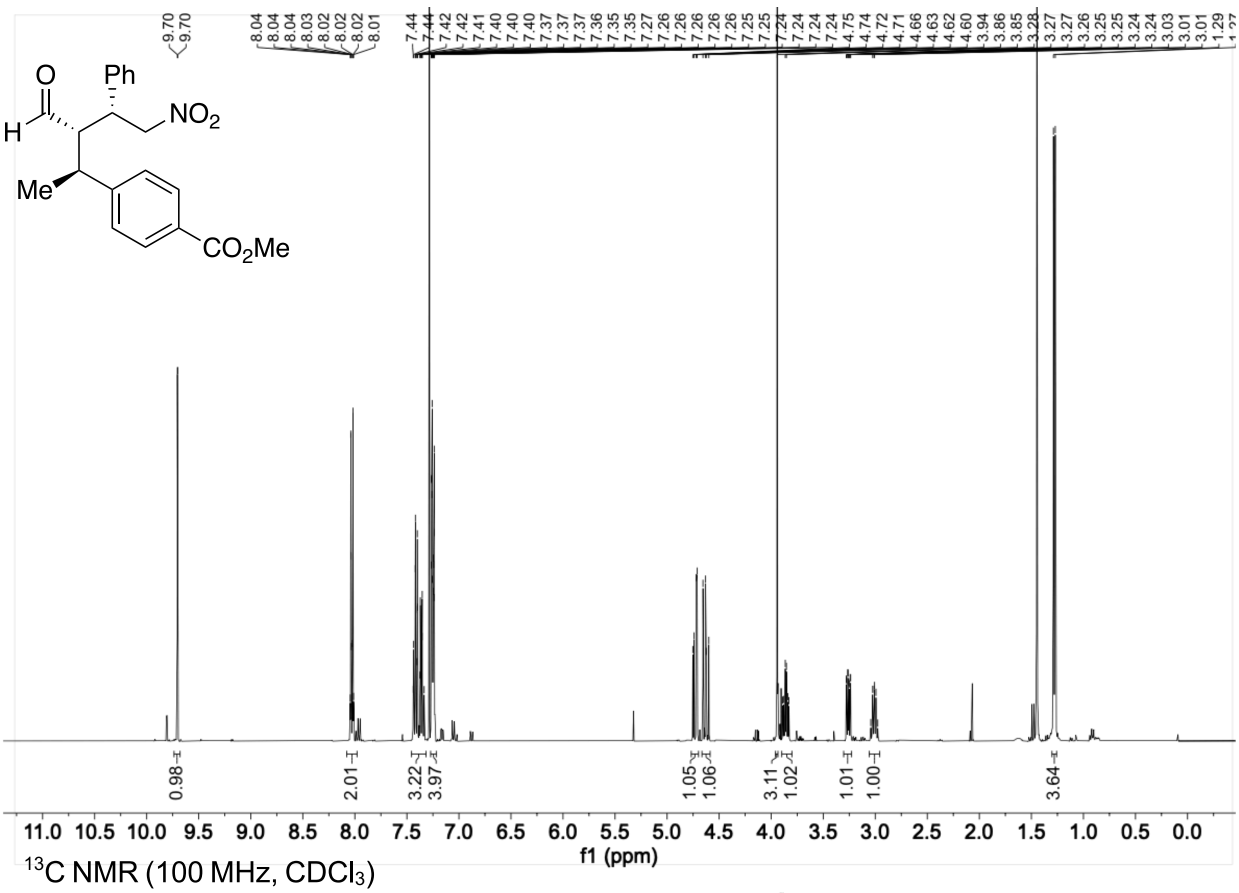

^{13}C NMR (100 MHz, $CDCl_3$)

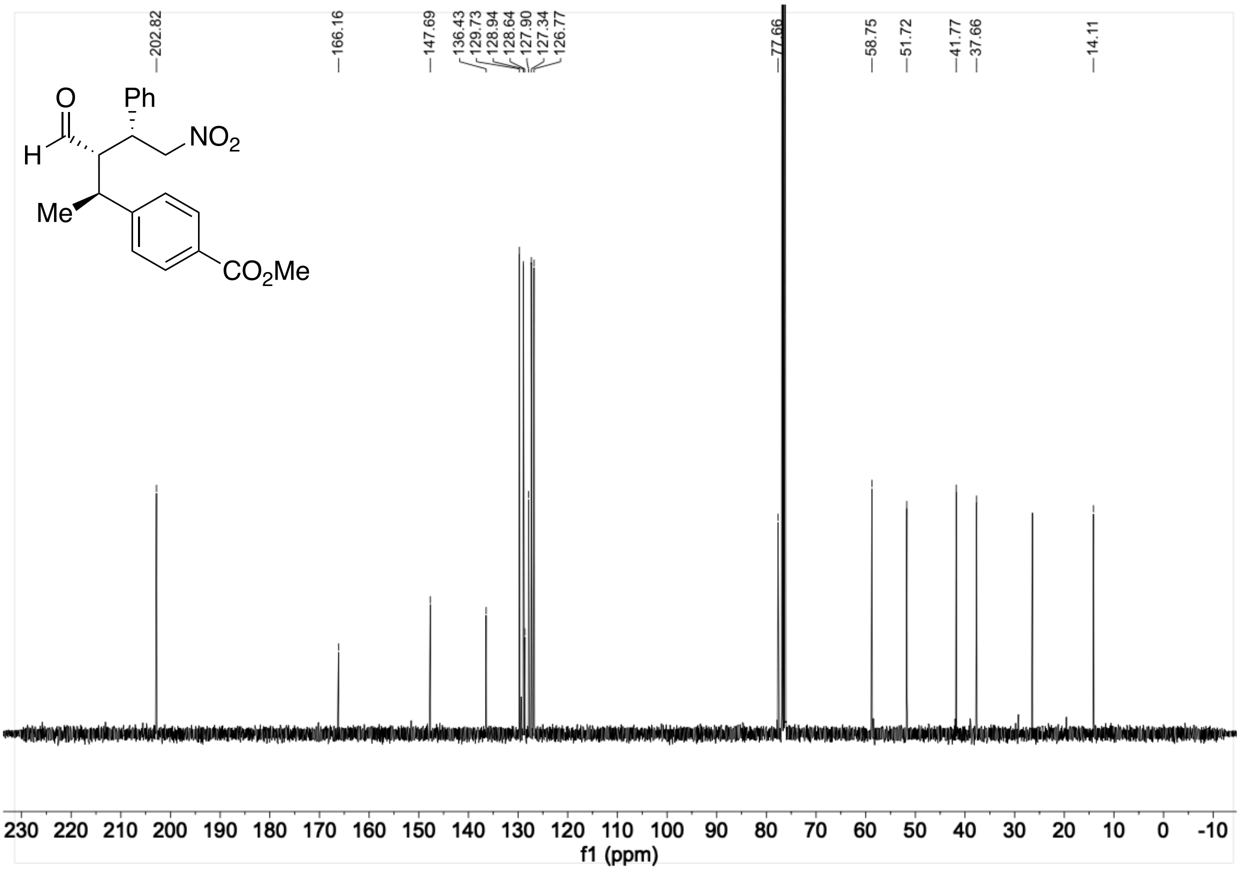

(2*S*,3*R*)-2-((*S*)-1-(4-Fluorophenyl)ethyl)-4-nitro-3-phenylbutanal (3ga)

^{1}H NMR (400 MHz, $CDCl_3$)

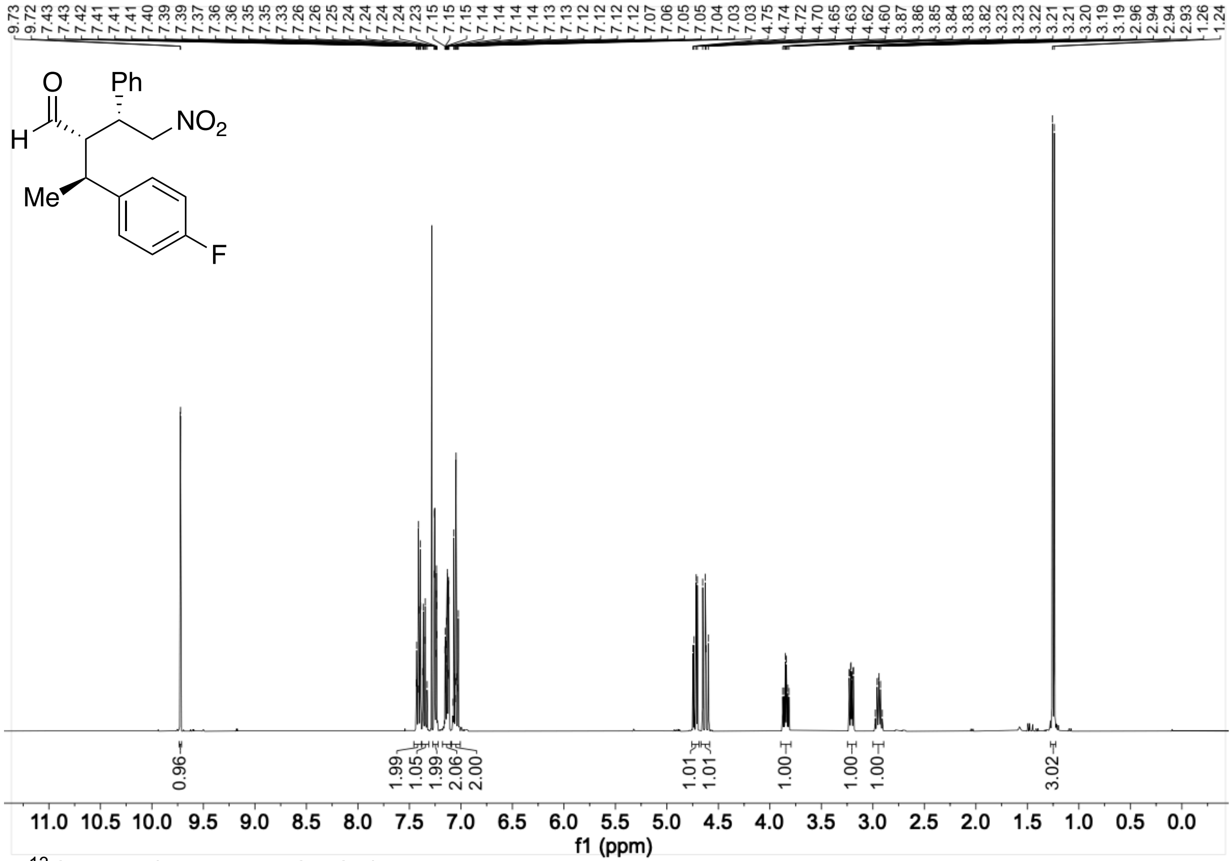

^{13}C NMR (100 MHz, $CDCl_3$)

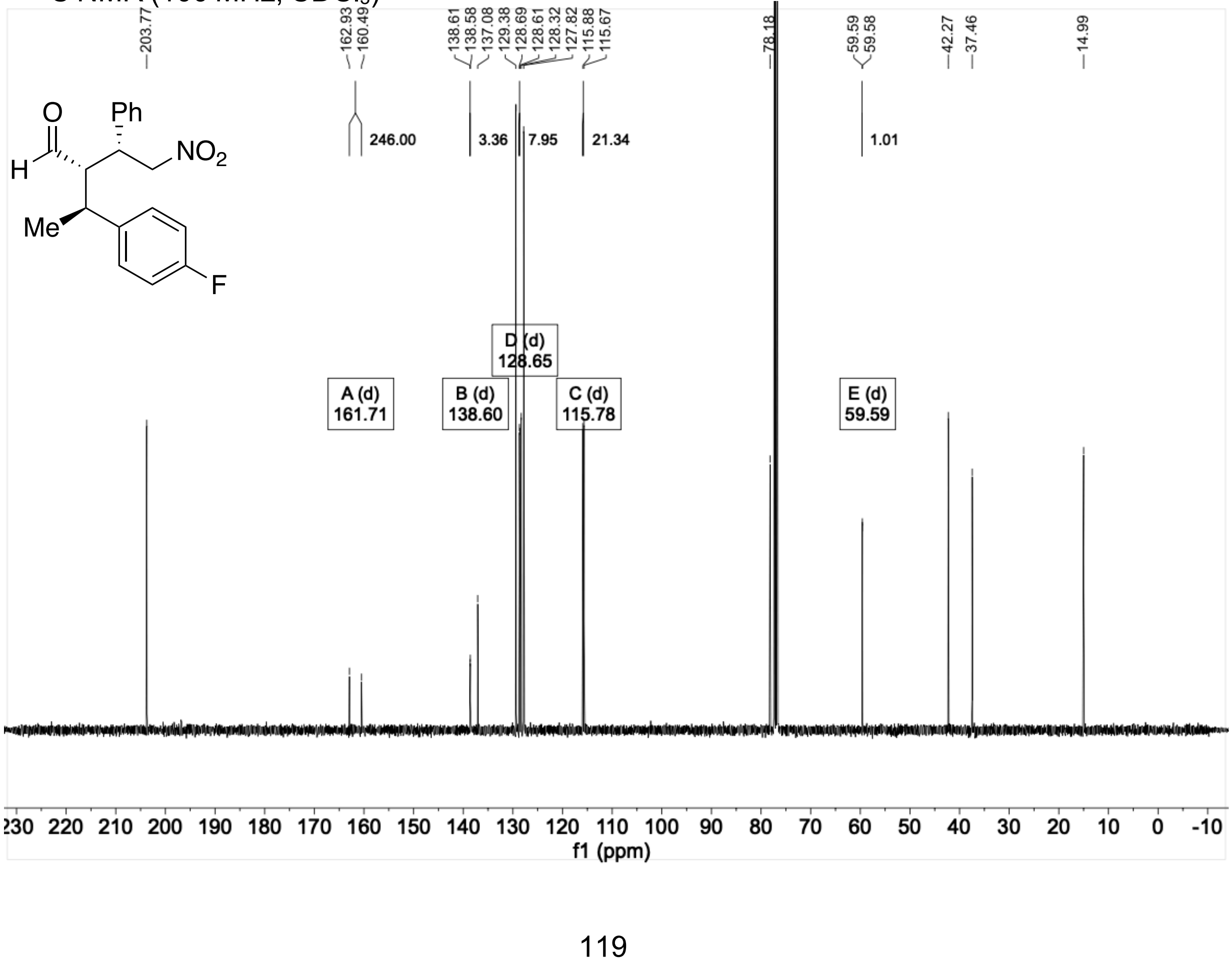

(2*S*,3*R*)-2-((*S*)-1-(3-Fluorophenyl)ethyl)-4-nitro-3-phenylbutanal (3ha)

^{1}H NMR (400 MHz, $CDCl_3$)

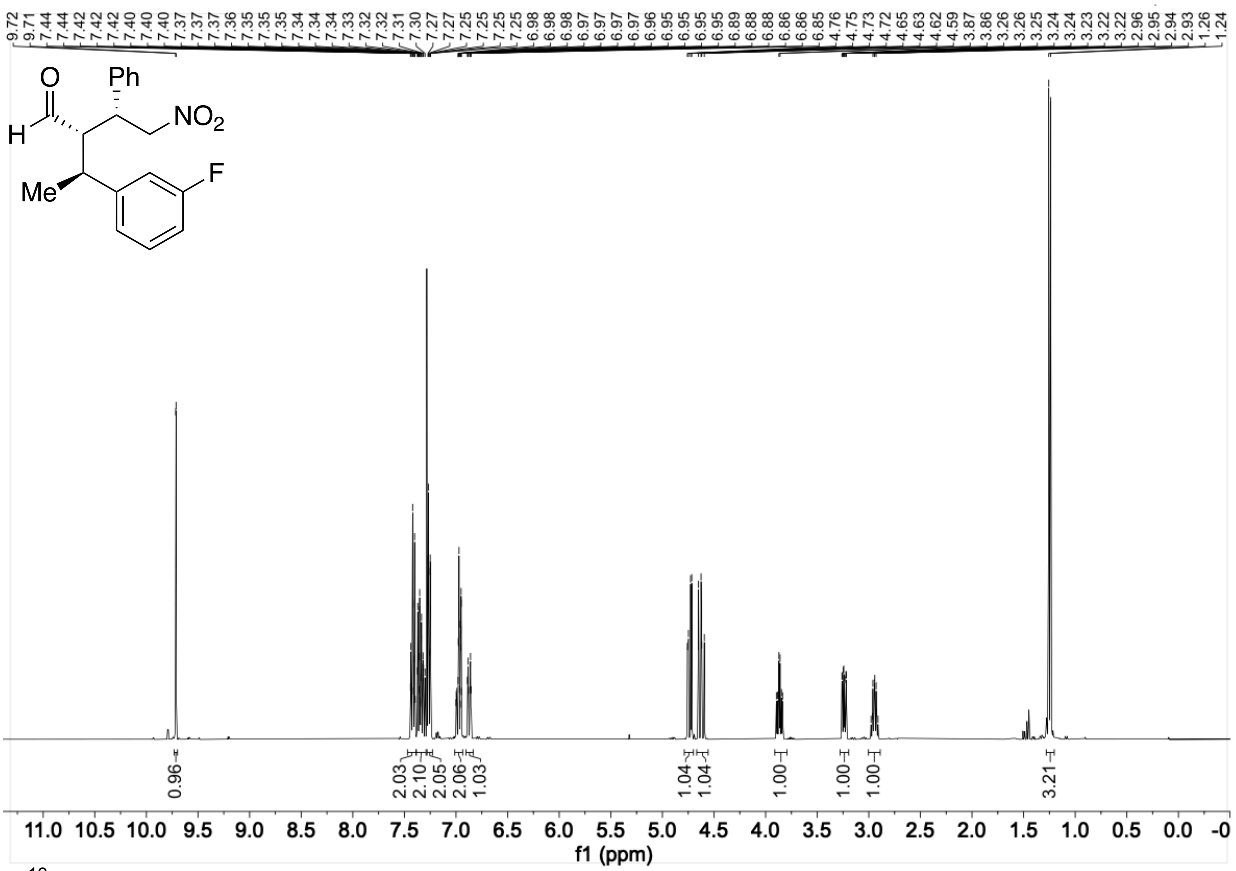

^{13}C NMR (100 MHz, $CDCl_3$)

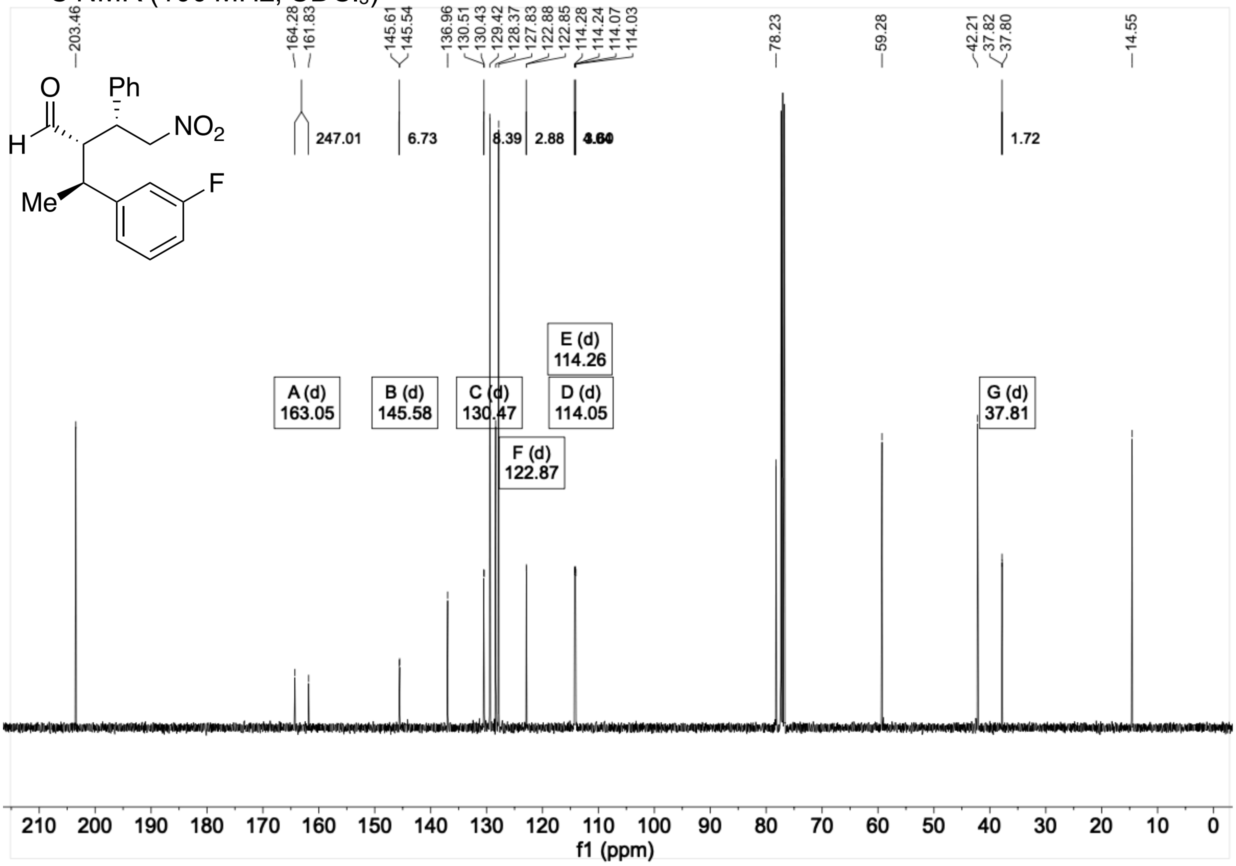

(2S,3R)-2-((S)-1-(2-Fluorophenyl)ethyl)-4-nitro-3-phenylbutanal (3ia)

^{1}H NMR (400 MHz, $CDCl_3$)

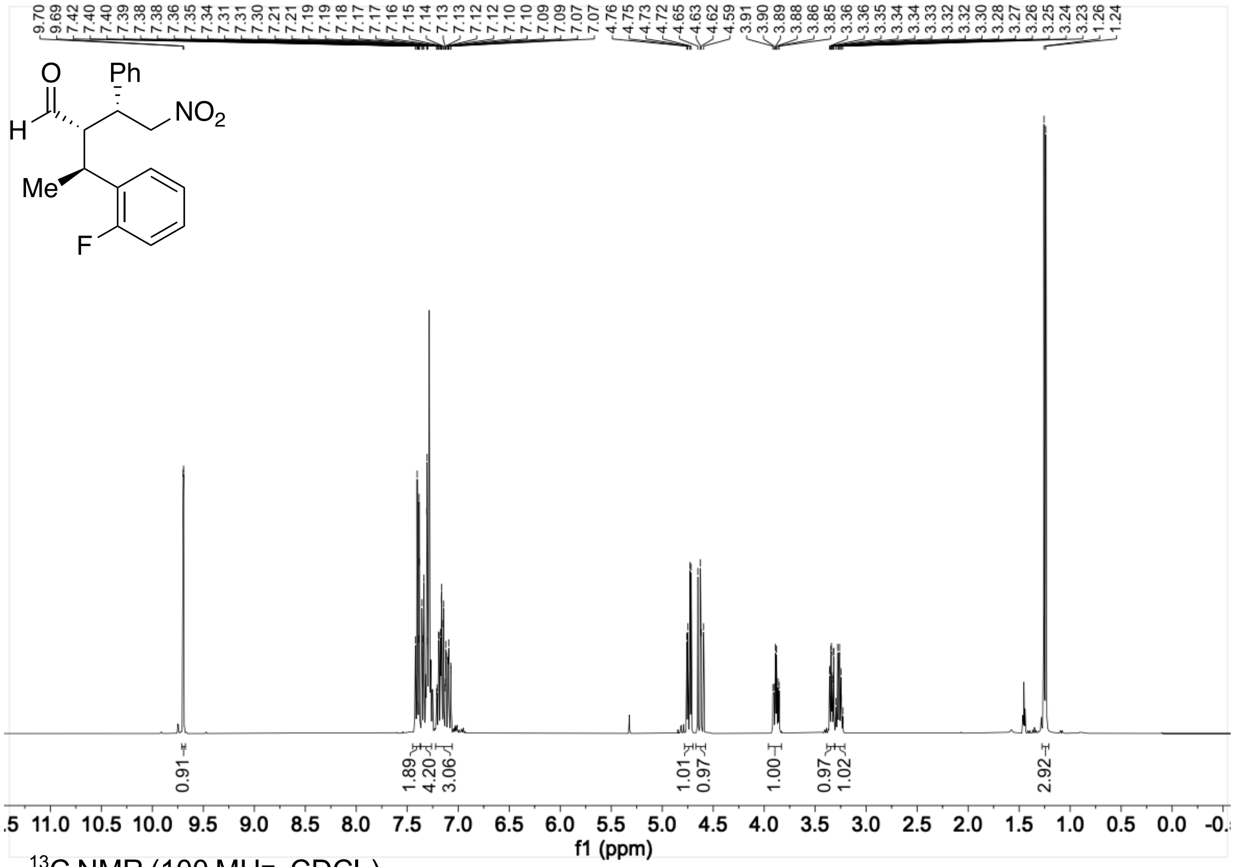

^{13}C NMR (100 MHz, $CDCl_3$)

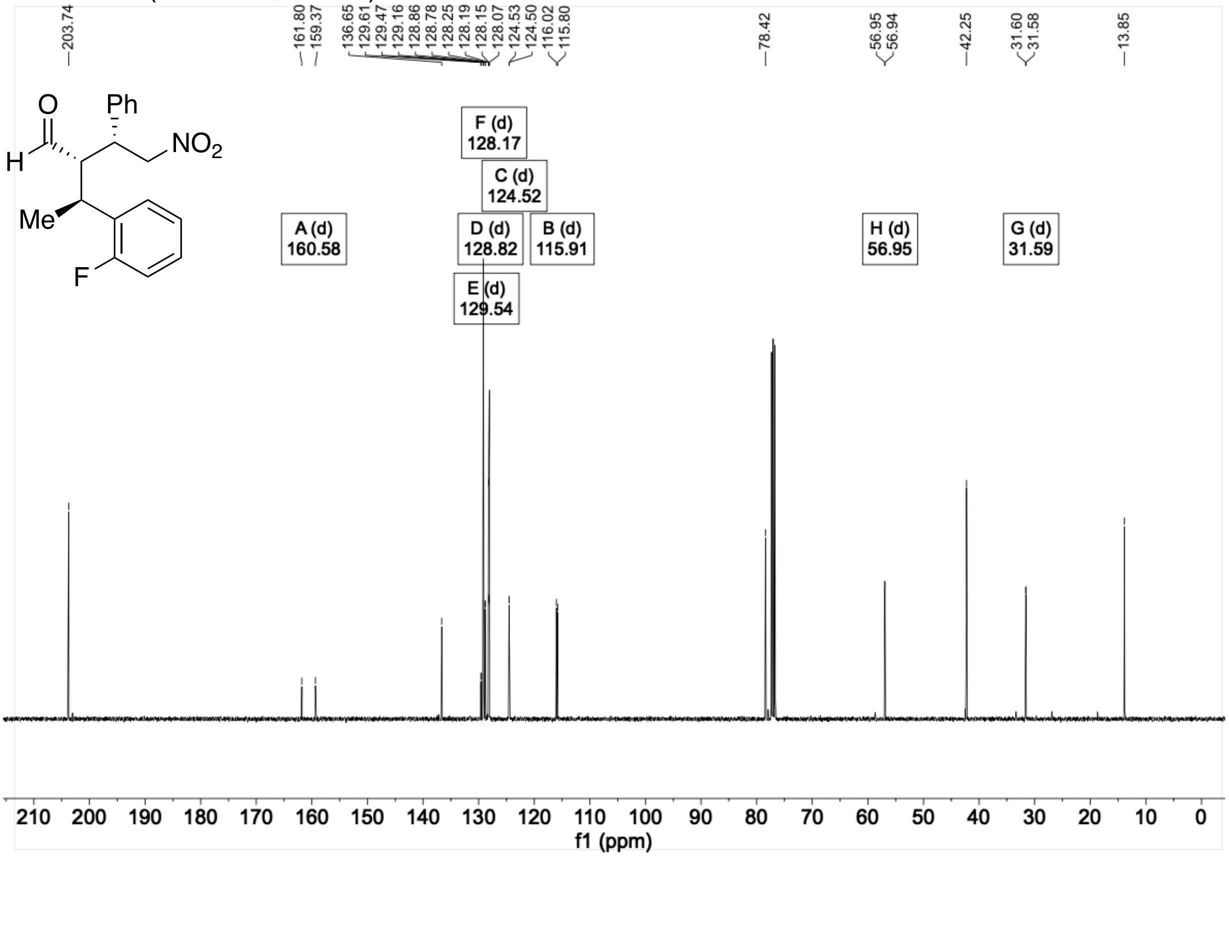

(2*S*,3*S*)-3-(4-Fluorophenyl)-2-((*R*)-2-nitro-1-phenylethyl)pentanal (3ka)

^{1}H NMR (500 MHz, $CDCl_3$)

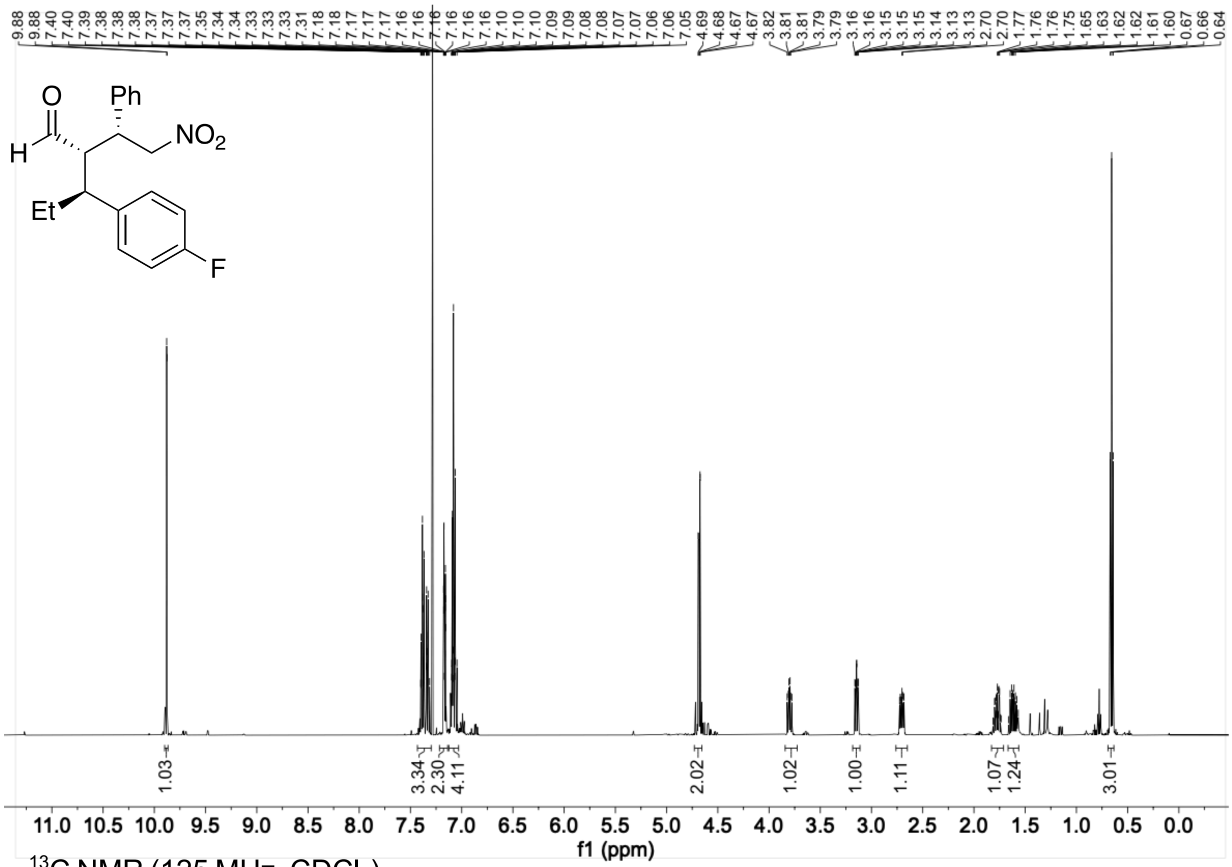

^{13}C NMR (125 MHz, $CDCl_3$)

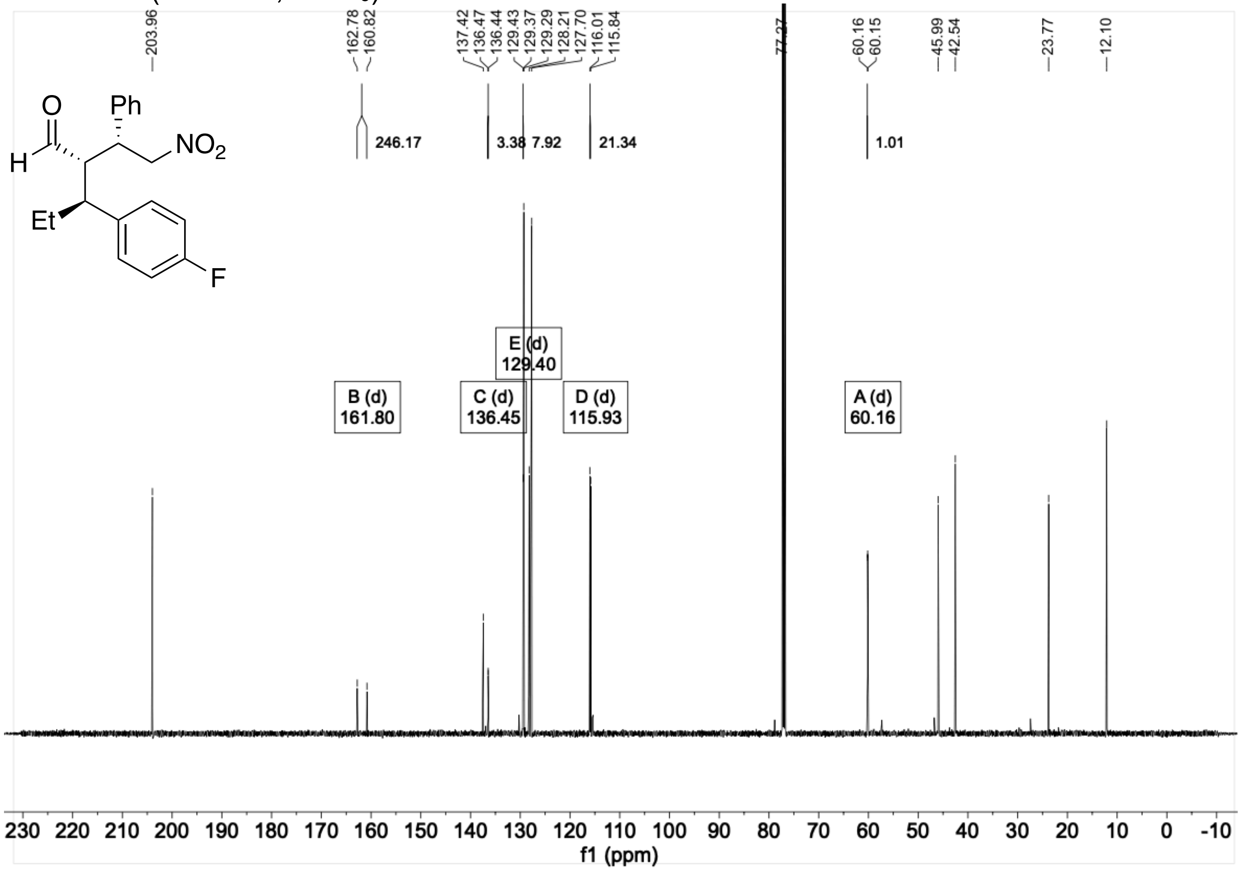

((2*S*,3*S*)-3-(4-Fluorophenyl)-2-((*R*)-2-nitro-1-phenylethyl)hexanal (3Ia)**

^{1}H NMR (400 MHz, $CDCl_3$)

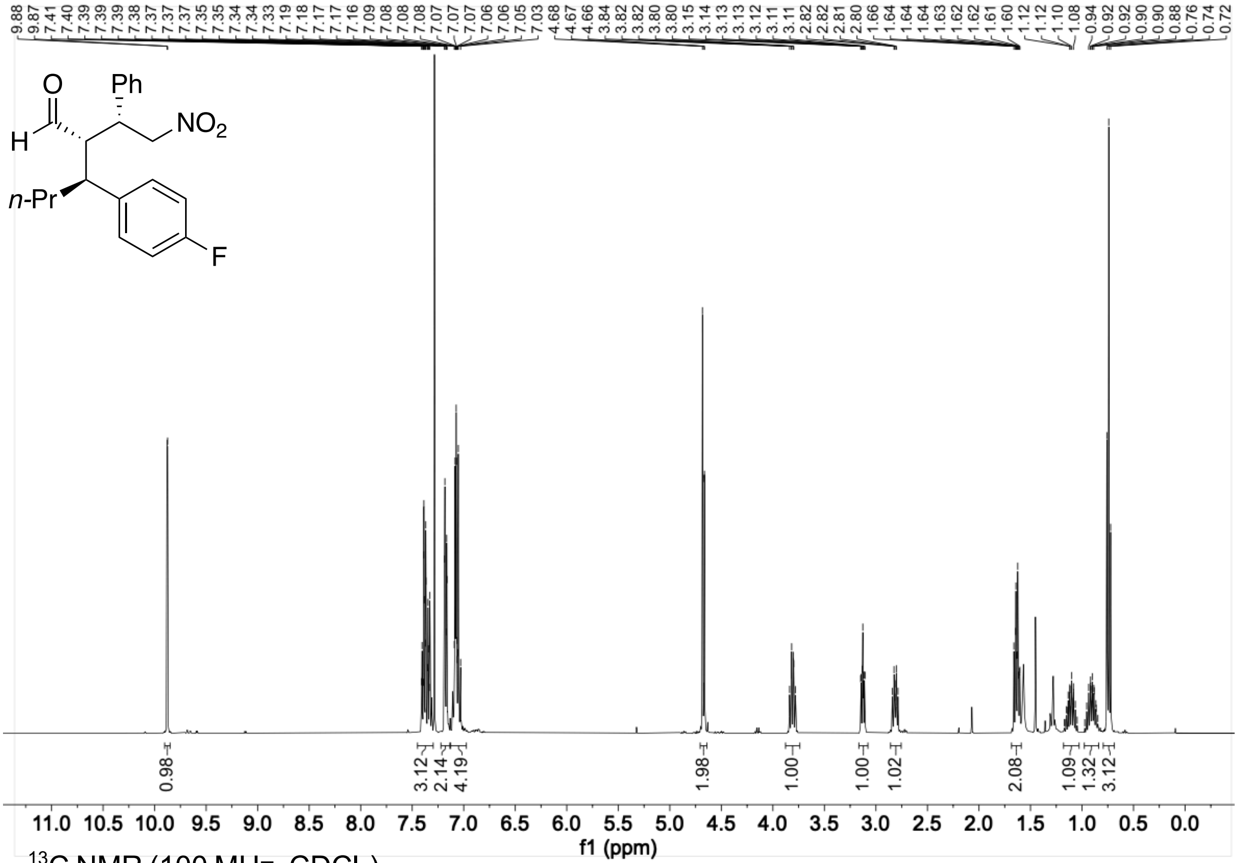

^{13}C NMR (100 MHz, $CDCl_3$)

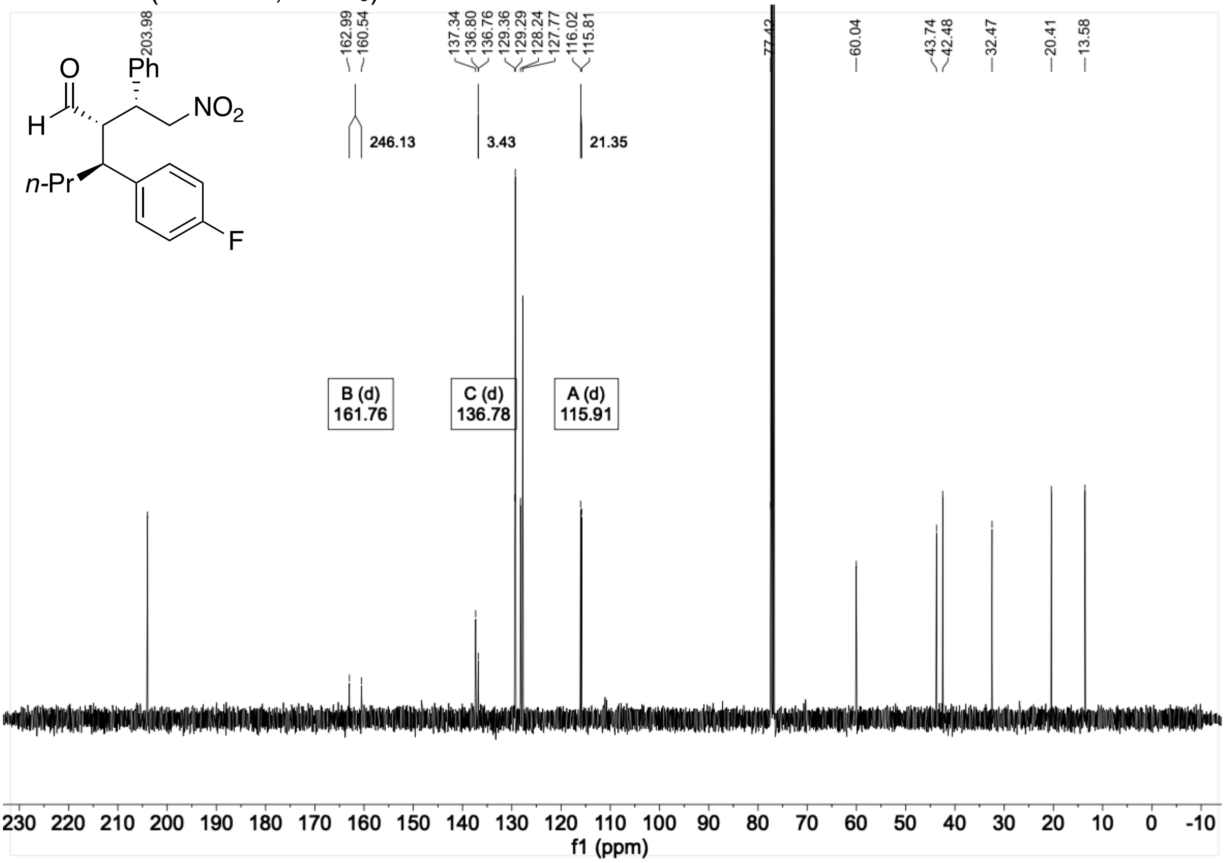

(2*S*,3*S*)-3-(4-Fluorophenyl)-2-((*R*)-2-nitro-1-phenylethyl)dodecanal (3ma)

^{1}H NMR (500 MHz, $CDCl_3$)

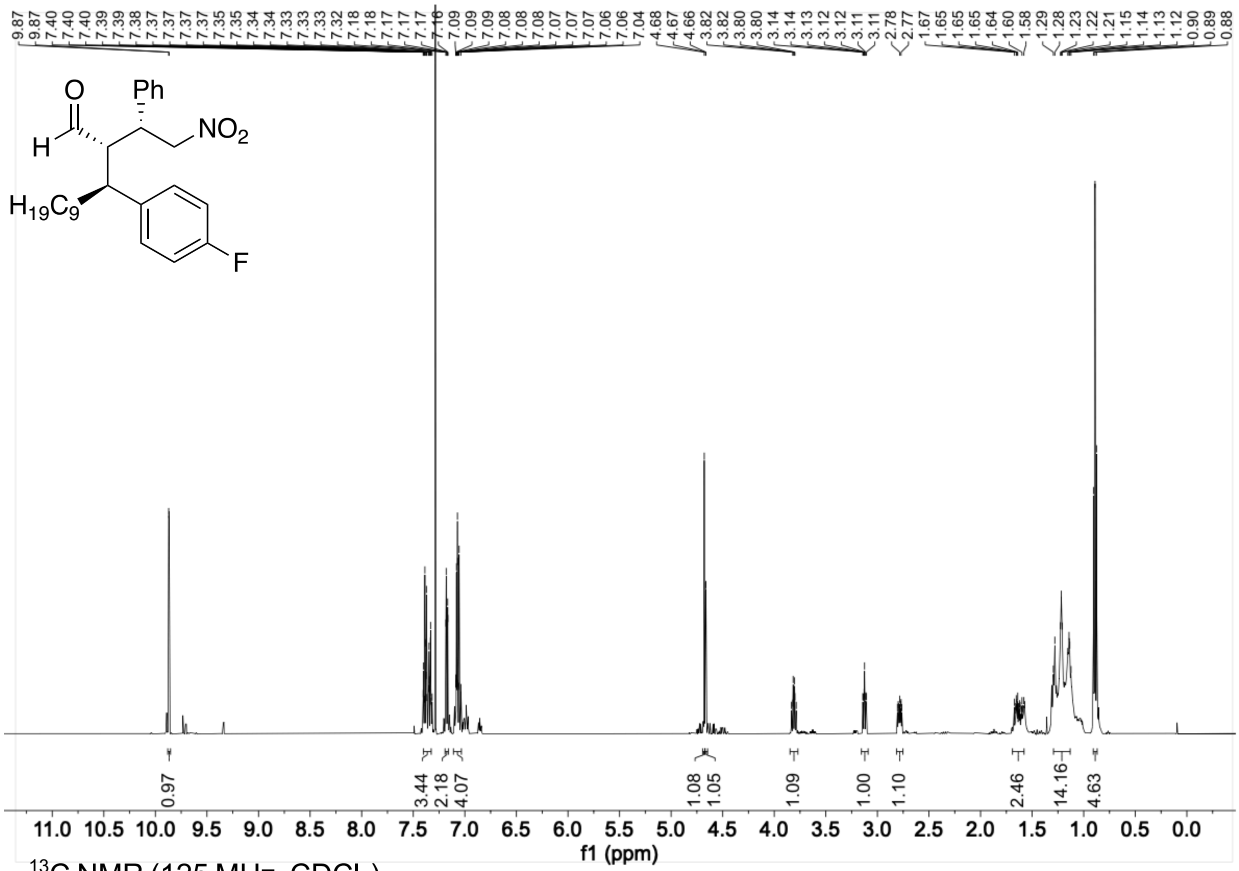

^{13}C NMR (125 MHz, $CDCl_3$)

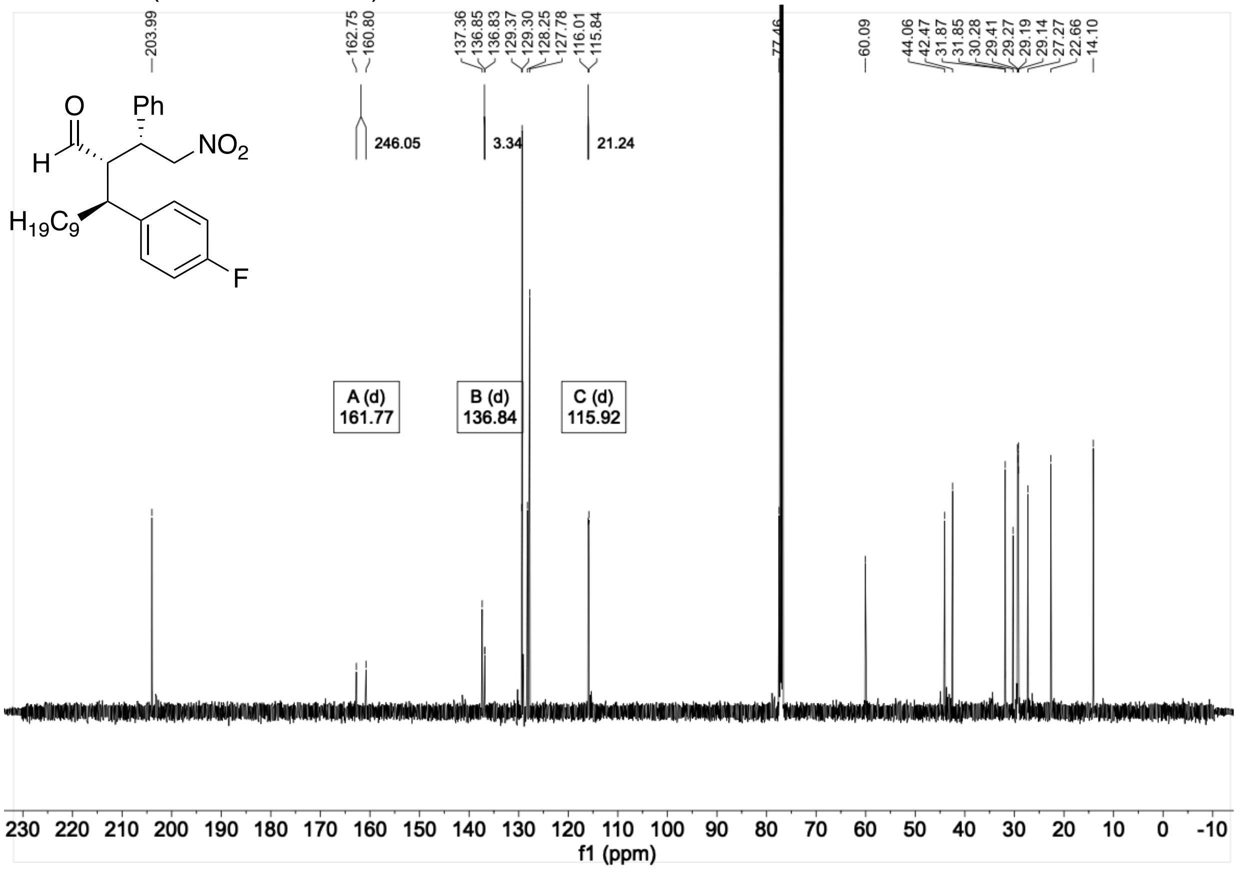

(2*S*,3*S*)-3-(4-Fluorophenyl)-4-methyl-2-((*R*)-2-nitro-1-phenylethyl)pentanal (3na)

^{1}H NMR (500 MHz, $CDCl_3$)

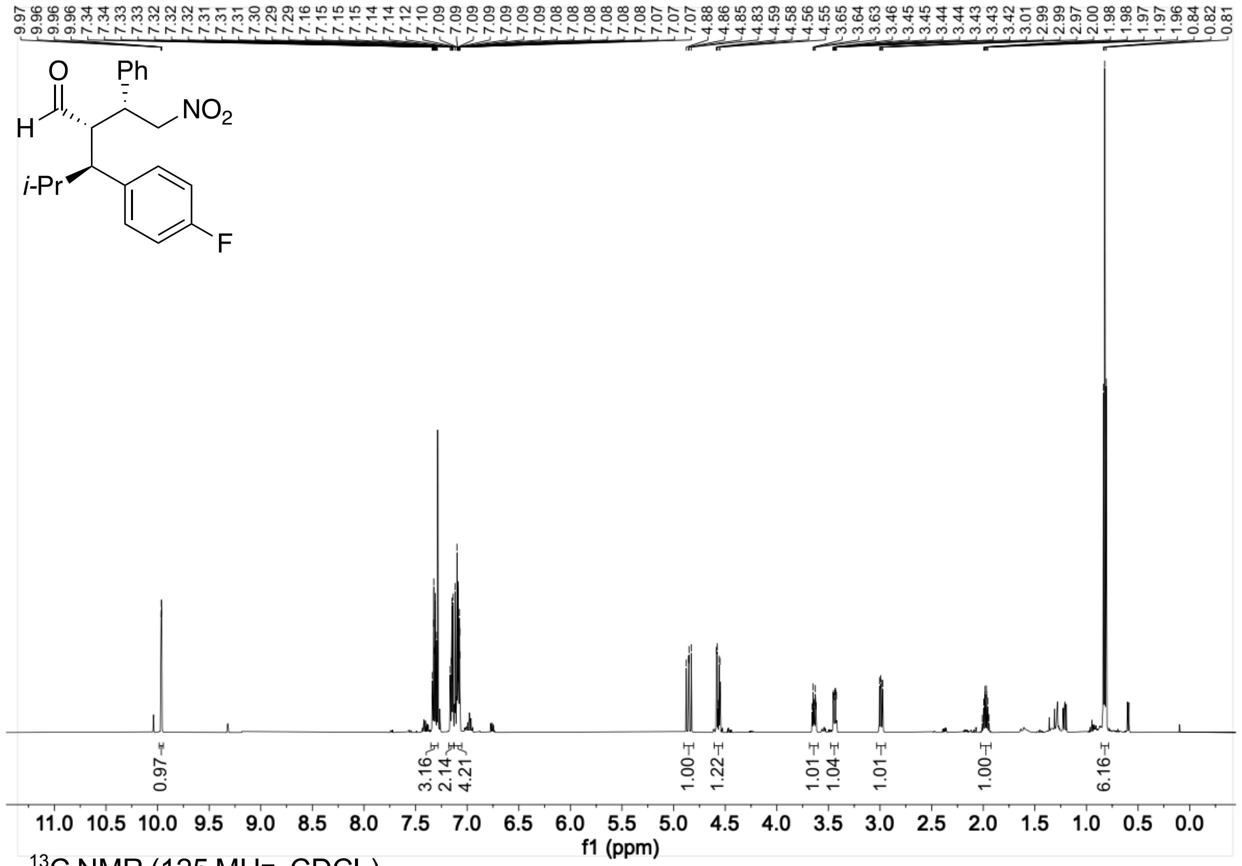

^{13}C NMR (125 MHz, $CDCl_3$)

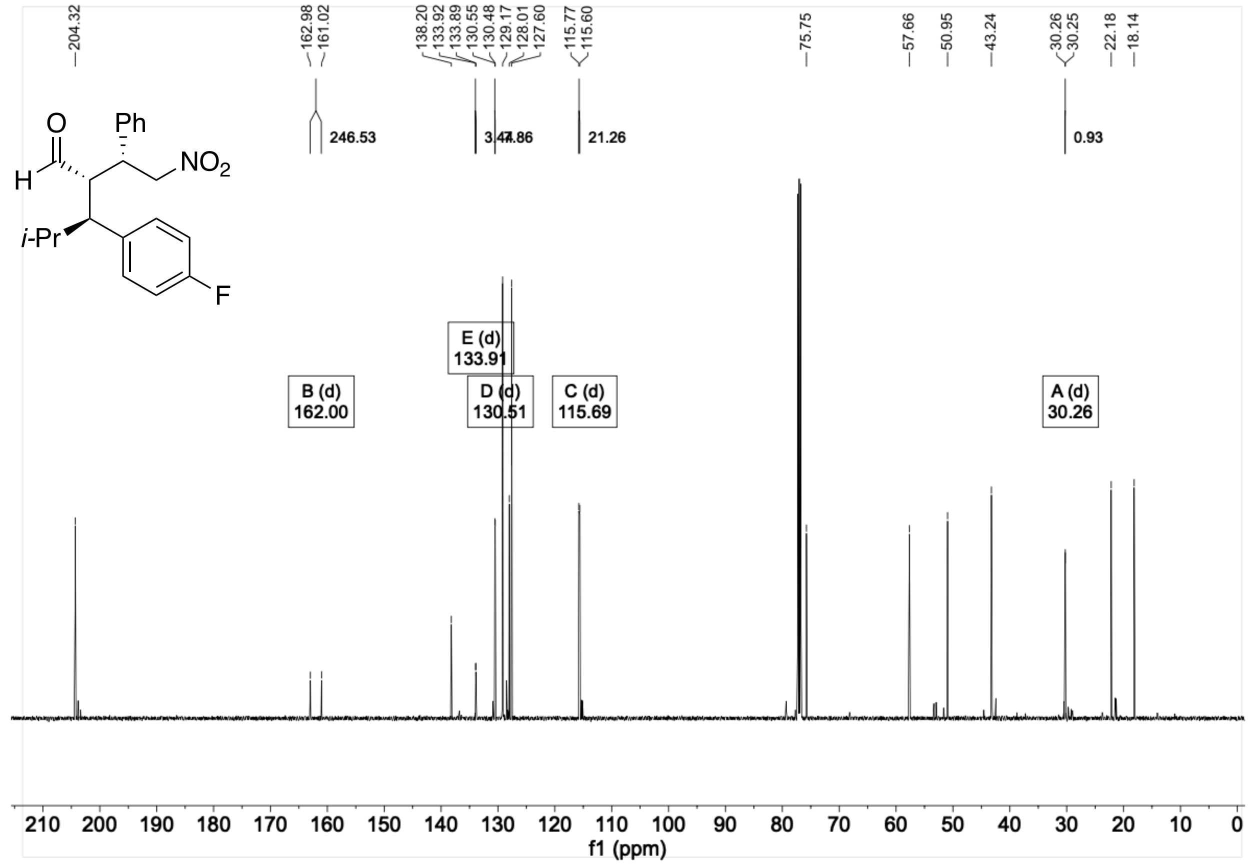

Methyl 4-((3*S*,4*S*,5*R*)-4-formyl-6-nitro-5-phenylhexan-3-yl)benzoate (3oa)

^{1}H NMR (500 MHz, $CDCl_3$)

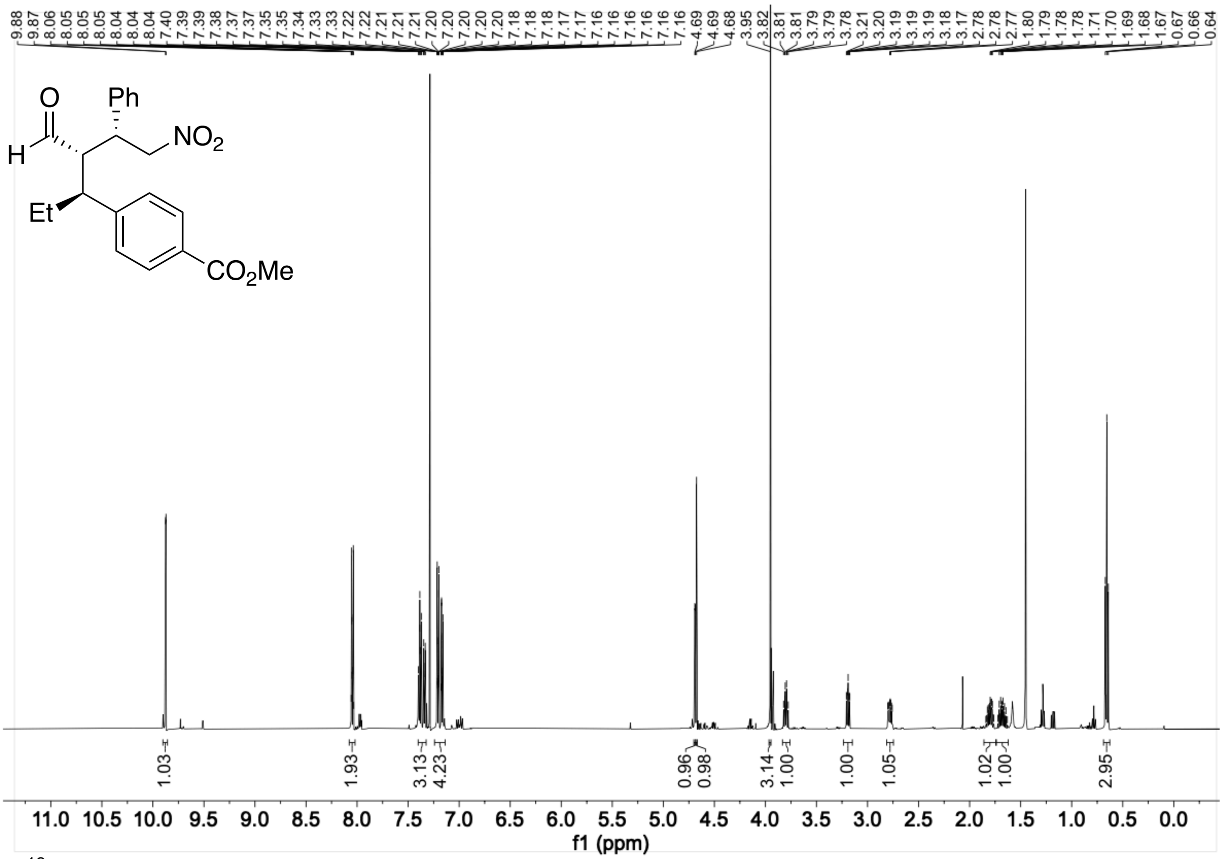

^{13}C NMR (125 MHz, $CDCl_3$)

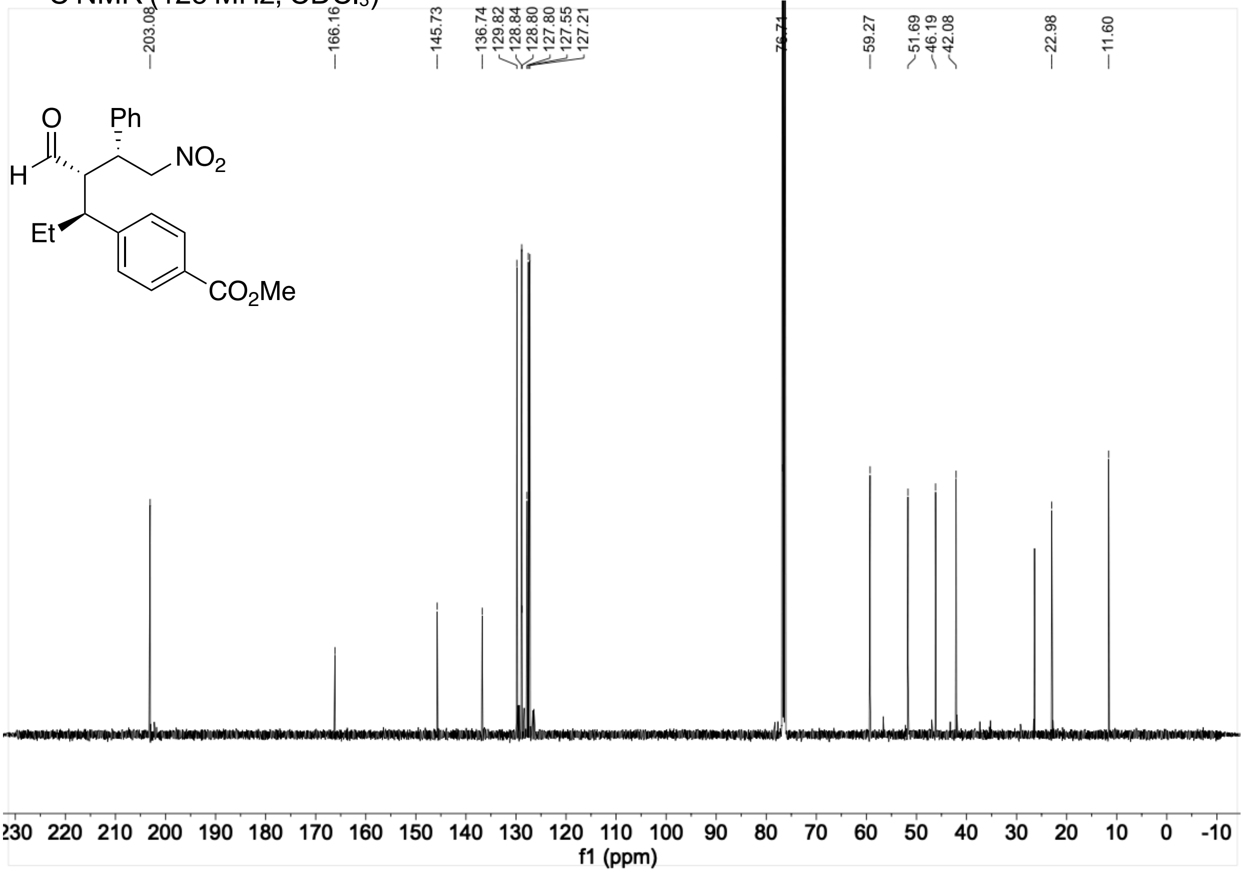

(2*R*,3*R*)-2-((*S*)-1-(5-Methylfuran-2-yl)ethyl)-4-nitro-3-phenylbutanal (3ja-diastA)

^{1}H NMR (500 MHz, $CDCl_3$)

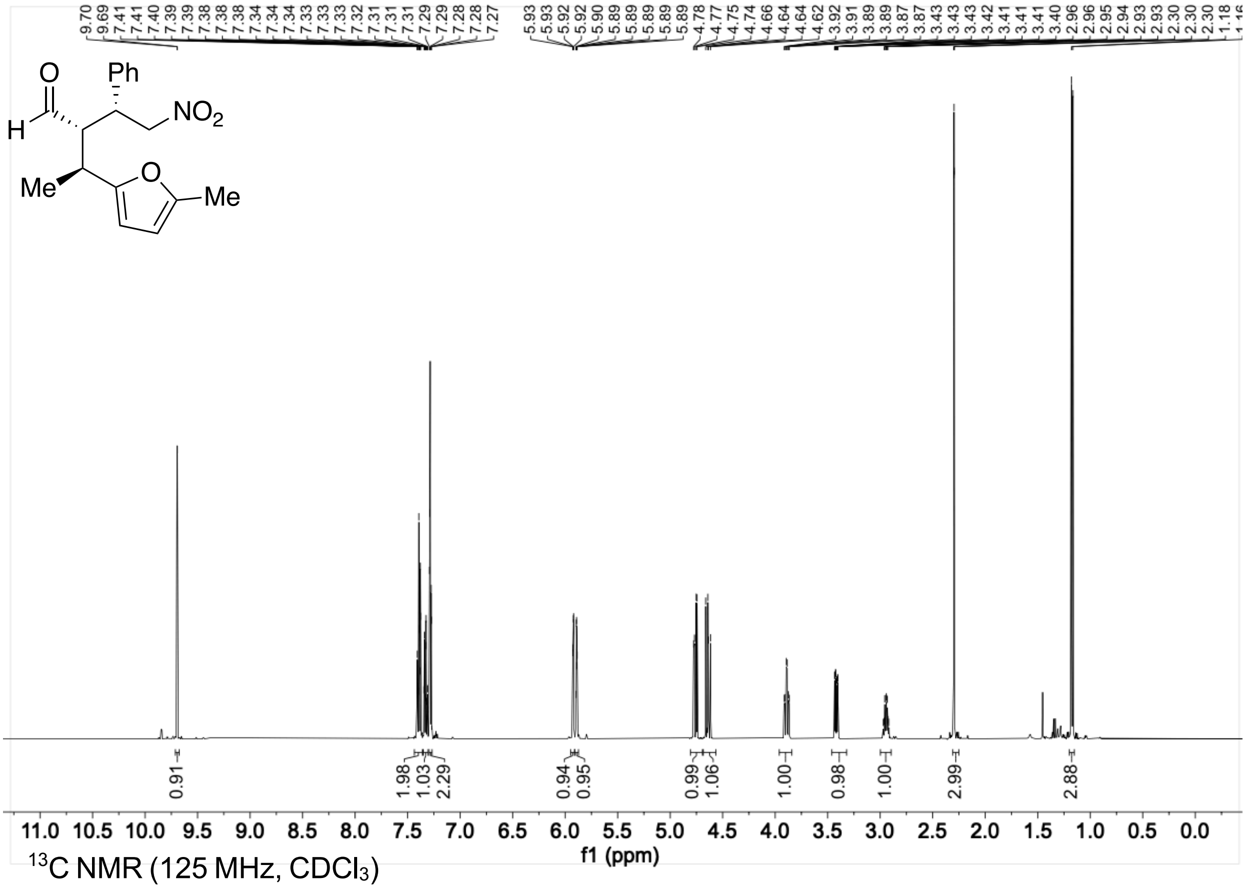

^{13}C NMR (125 MHz, $CDCl_3$)

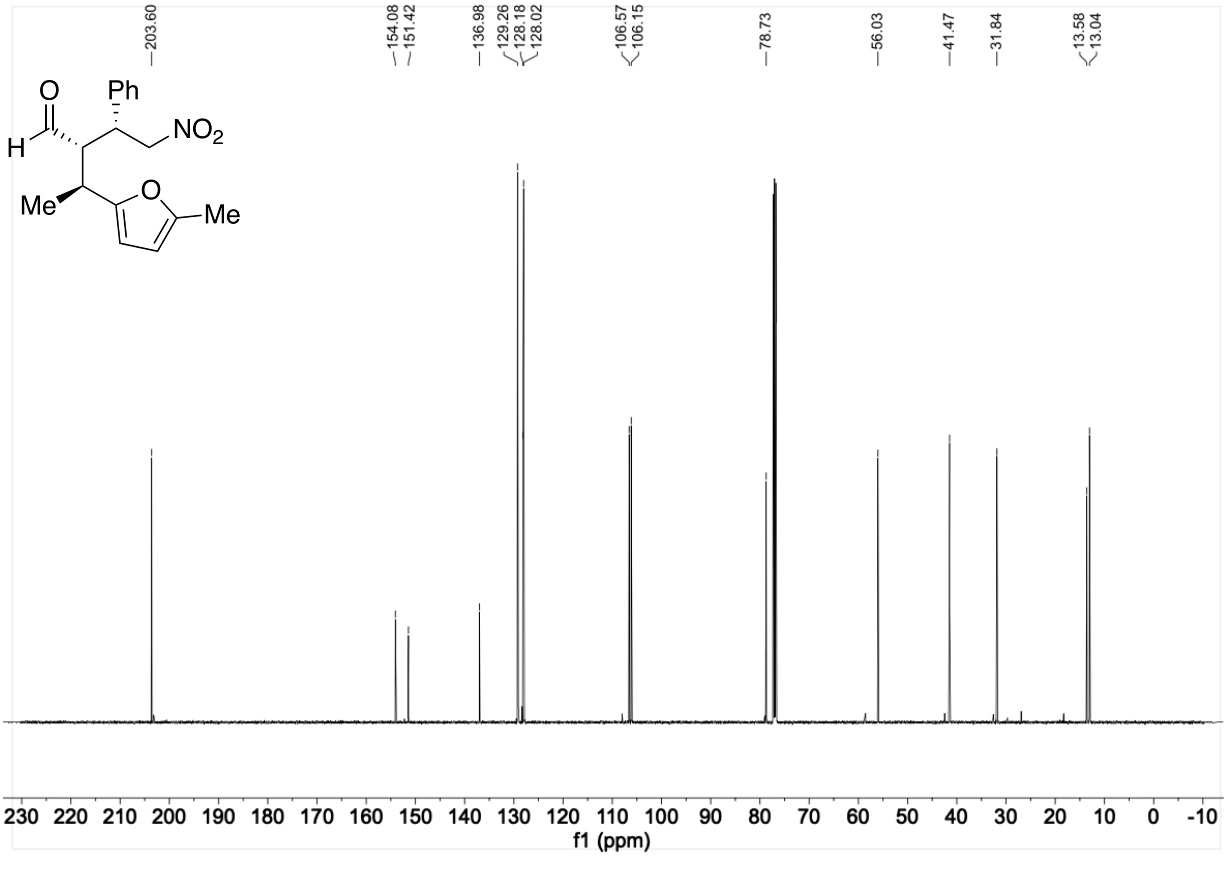

(2*R*,3*R*)-2-((*R*)-1-(5-Methylfuran-2-yl)ethyl)-4-nitro-3-phenylbutanal (3ja-diastB)

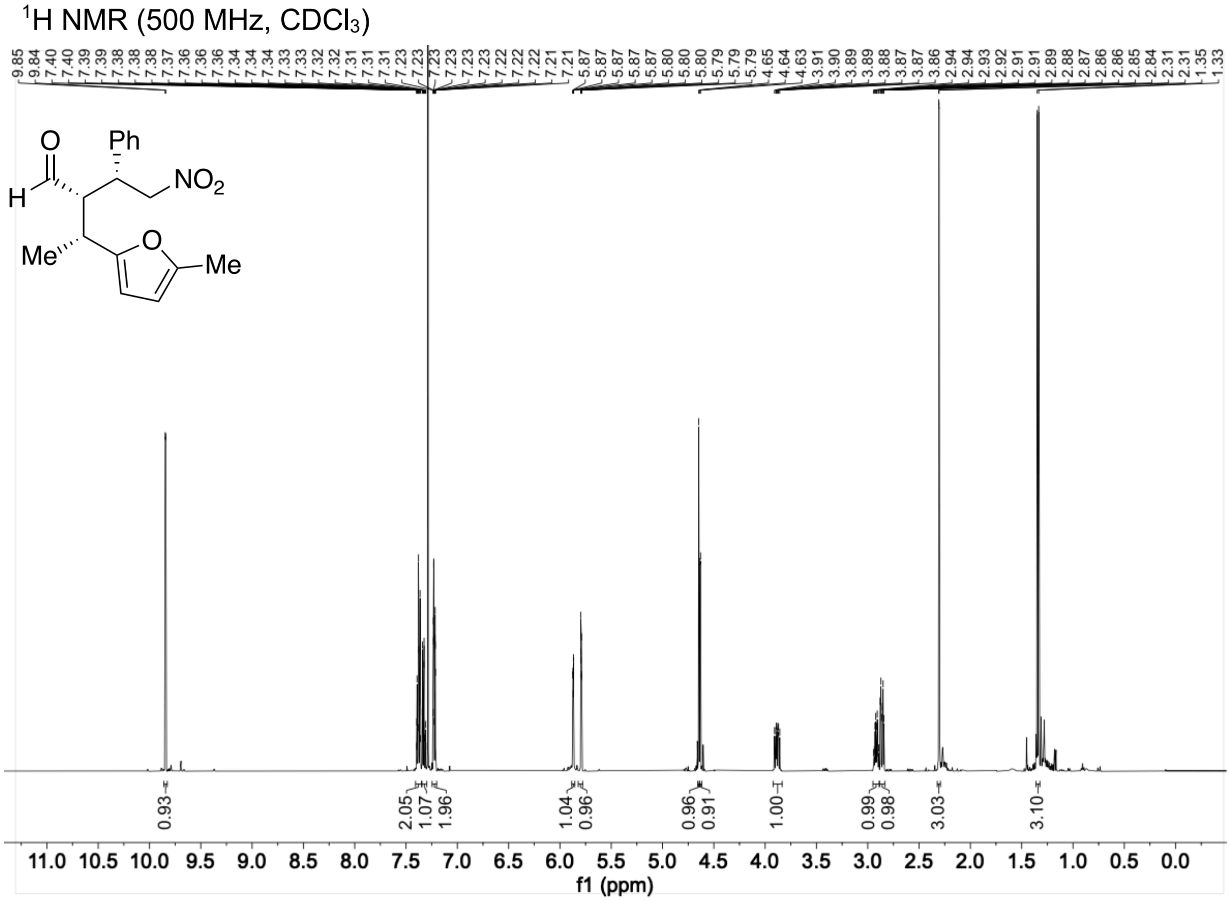

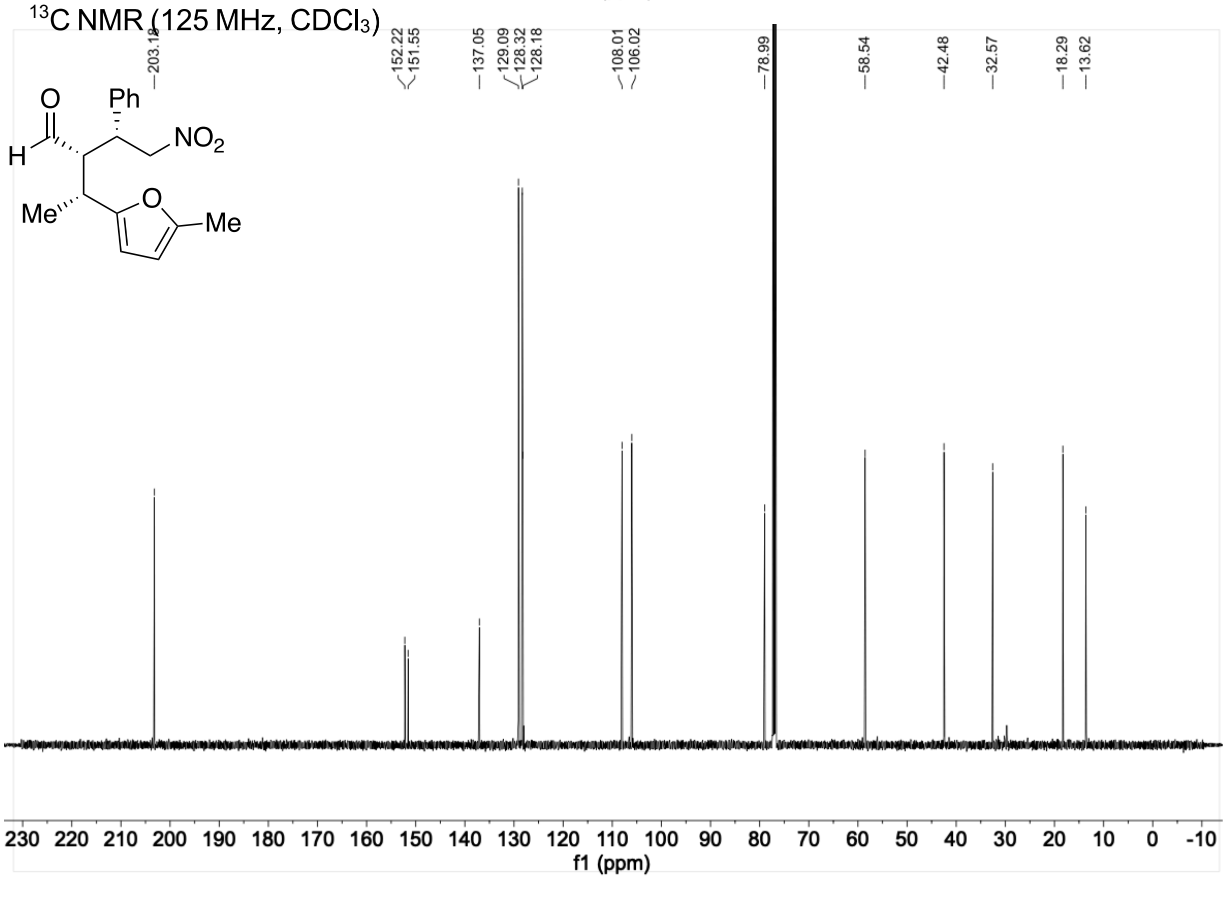

(2*S*,3*R*)-3,5,5-Trimethyl-2-((*R*)-2-nitro-1-phenylethyl)hexanal (3pa)

^{1}H NMR (400 MHz, $CDCl_3$)

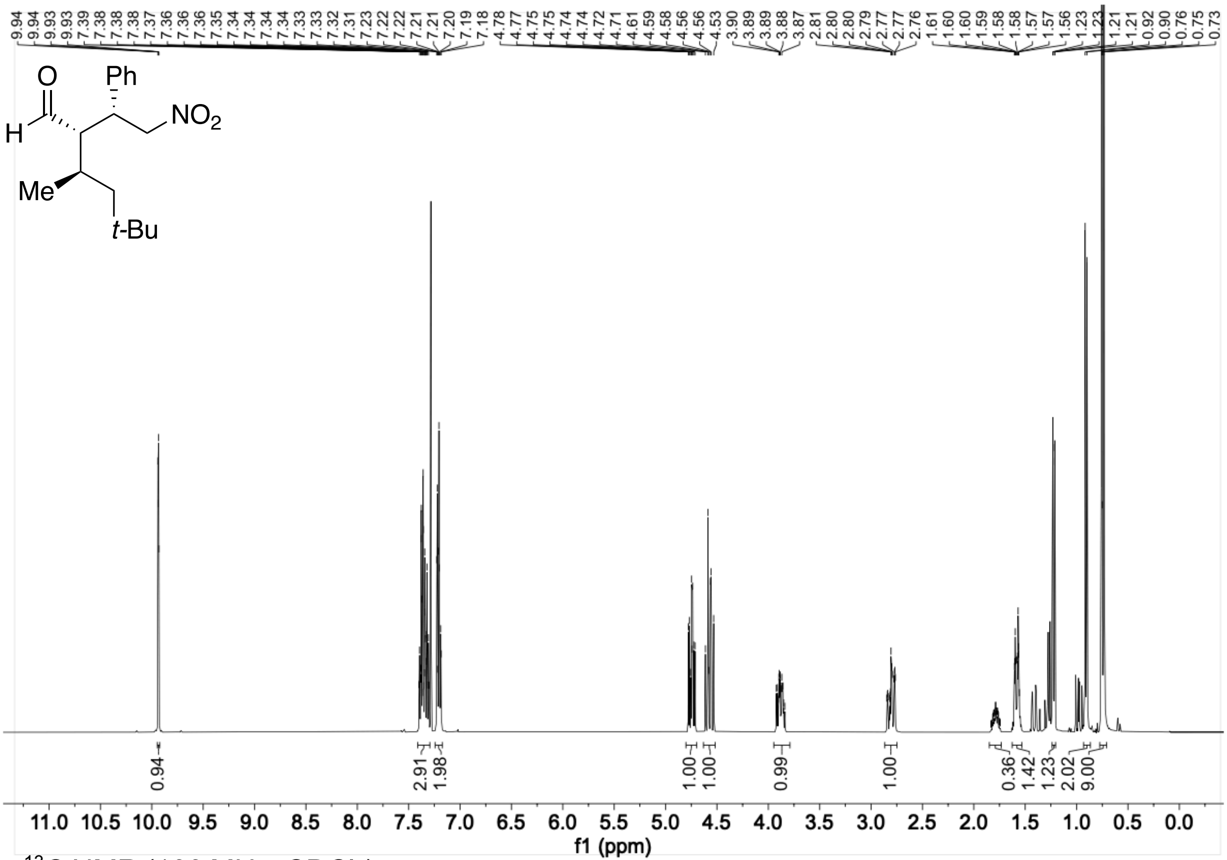

^{13}C NMR (100 MHz, $CDCl_3$)

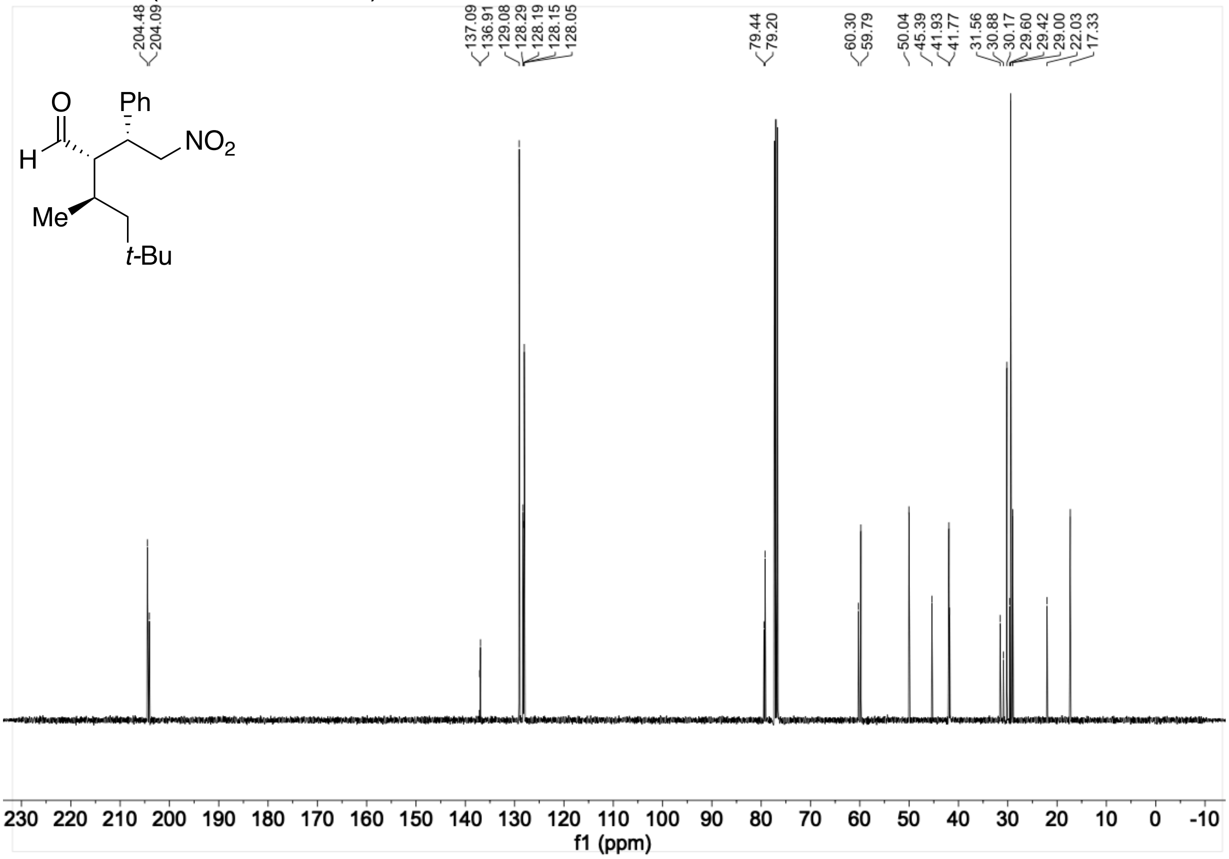

(2*S*,3*R*)-7-Hydroxy-3,7-dimethyl-2-((*R*)-2-nitro-1-phenylethyl)octanal (3qa)

^{1}H NMR (400 MHz, $CDCl_3$)

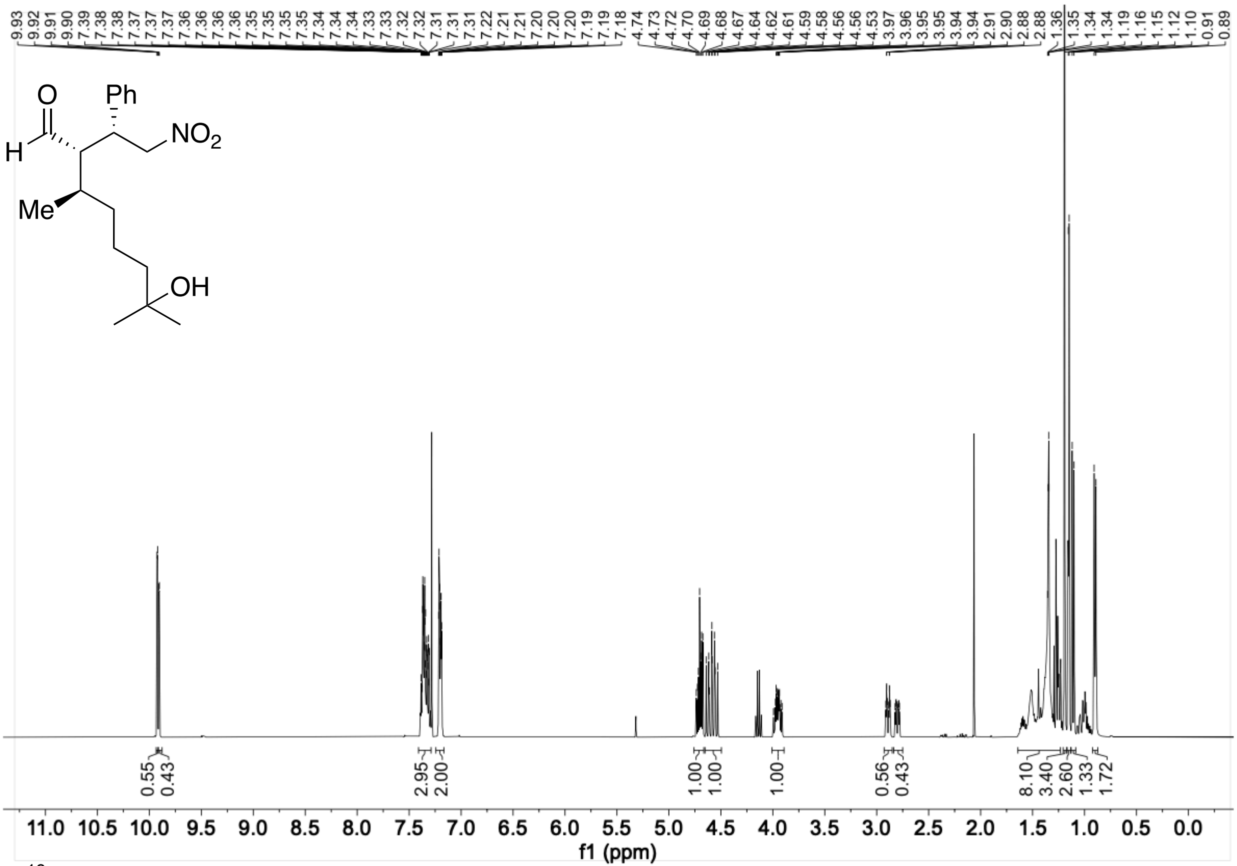

^{13}C NMR (100 MHz, $CDCl_3$)

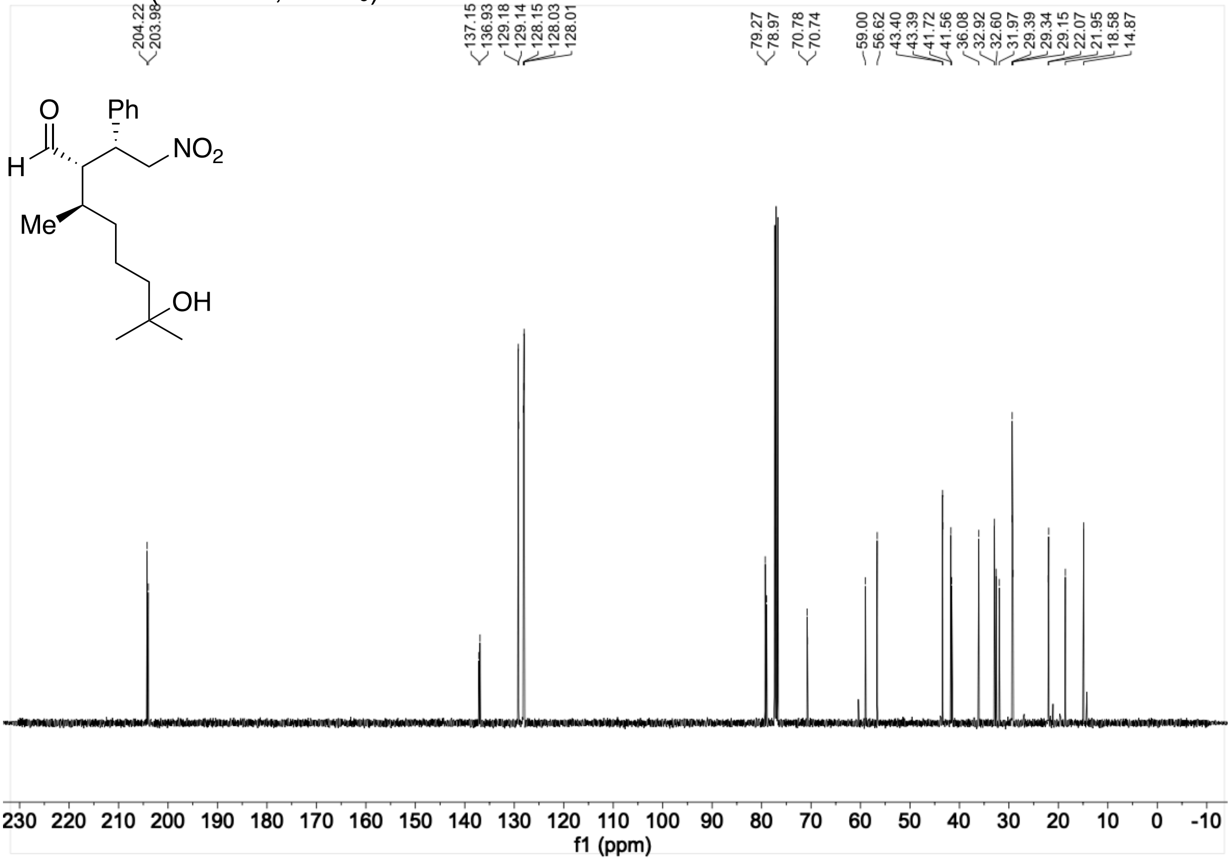

(2*S*,3*R*)-3-(4-fluorophenyl)-4-nitro-2-((*S*)-1-phenylethyl)butanal (3ab)

^{1}H NMR (500 MHz, CDCl$_3$)

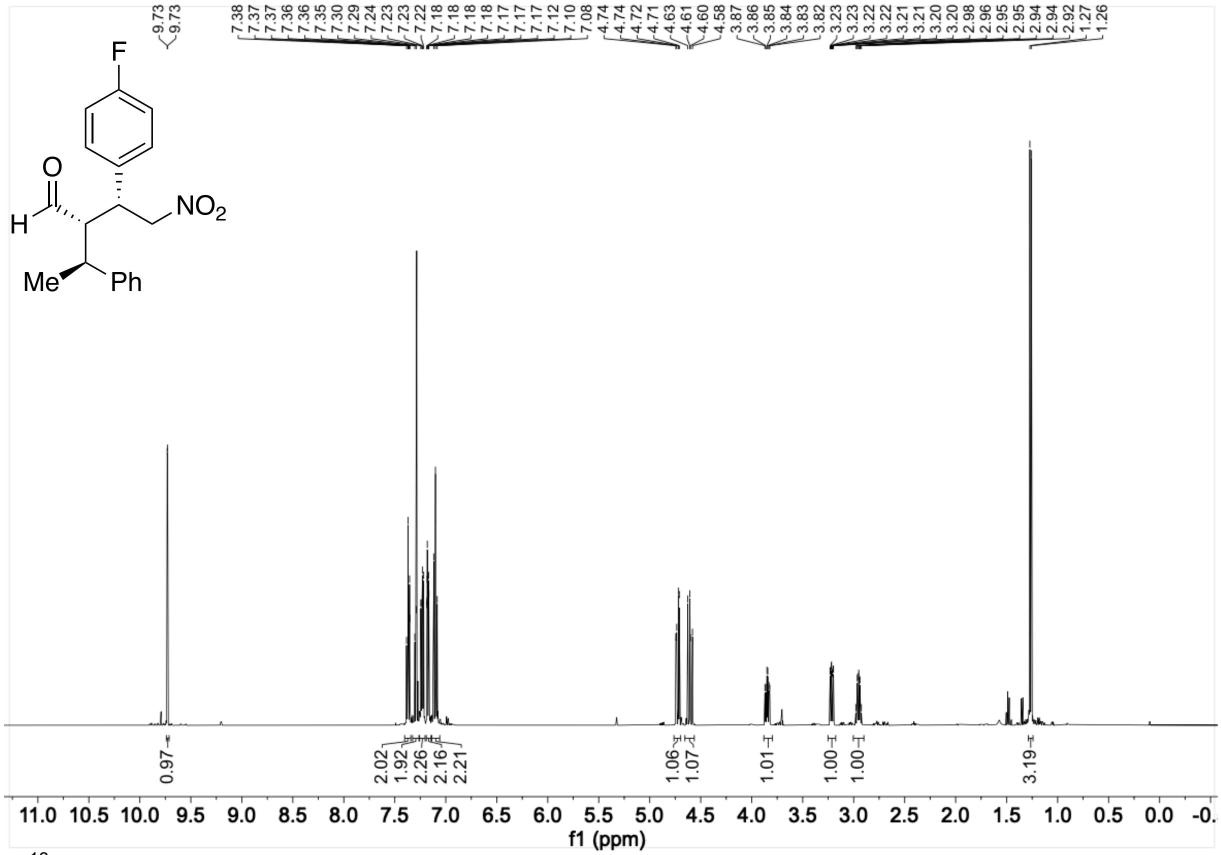

^{13}C NMR (125 MHz, CDCl$_3$)

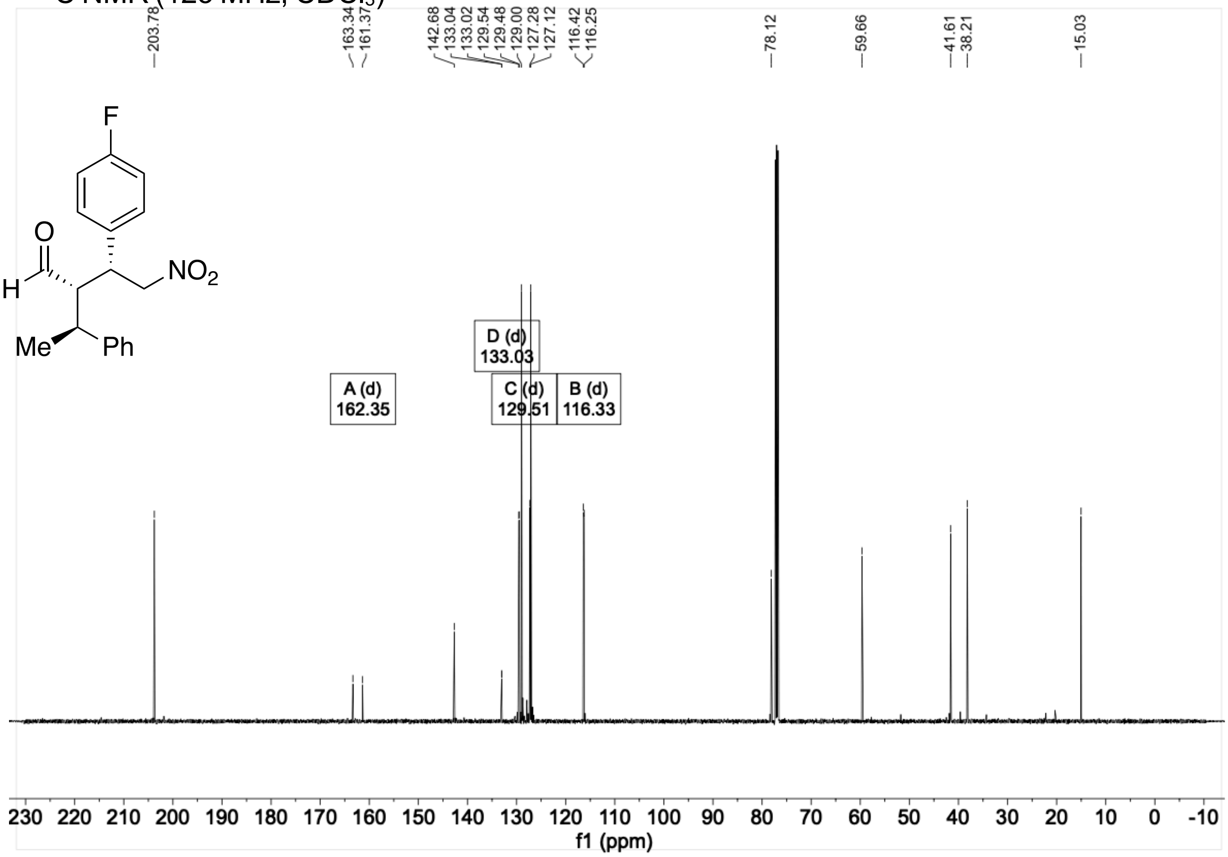

(2*S*,3*R*)-3-(4-Chlorophenyl)-4-nitro-2-((*S*)-1-phenylethyl)butanal (3ac)

^{1}H NMR (500 MHz, $CDCl_3$)

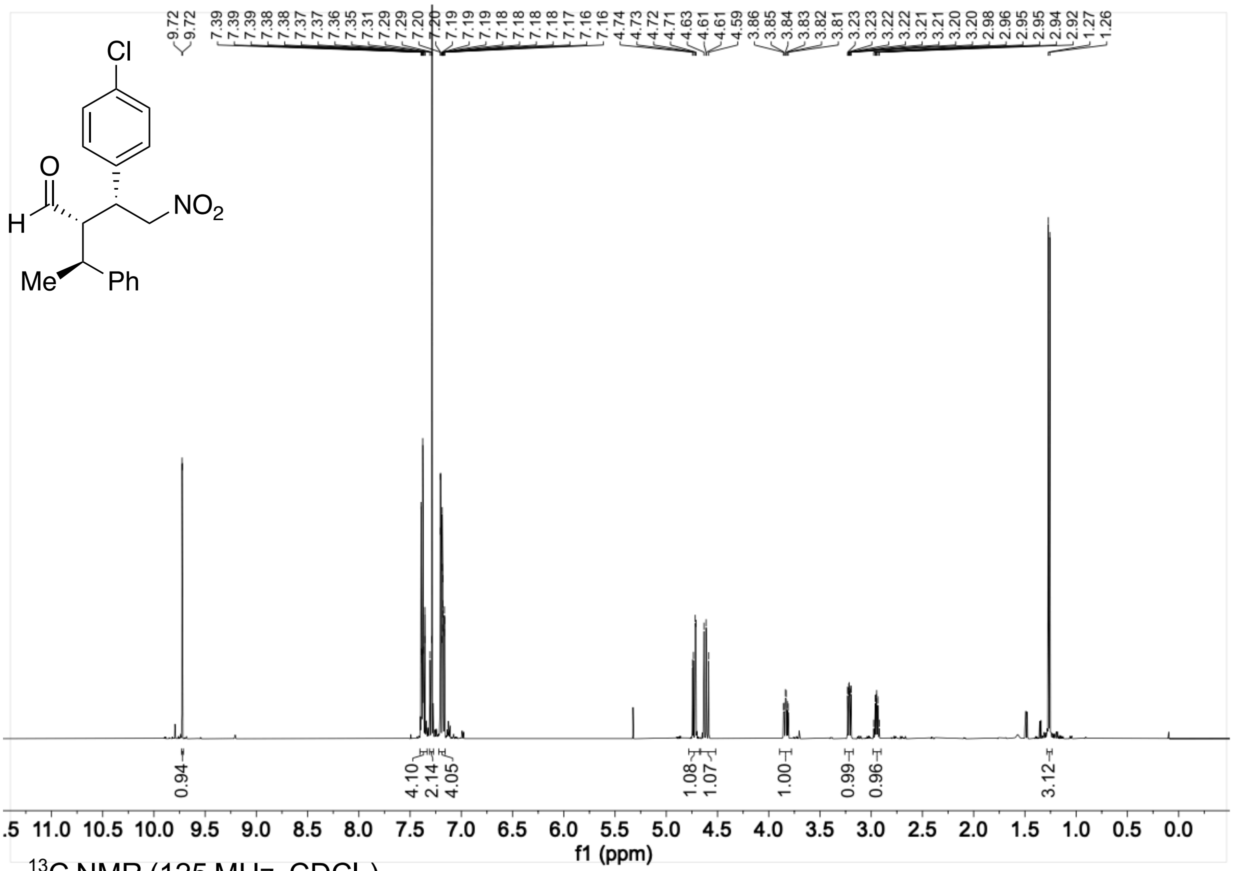

^{13}C NMR (125 MHz, $CDCl_3$)

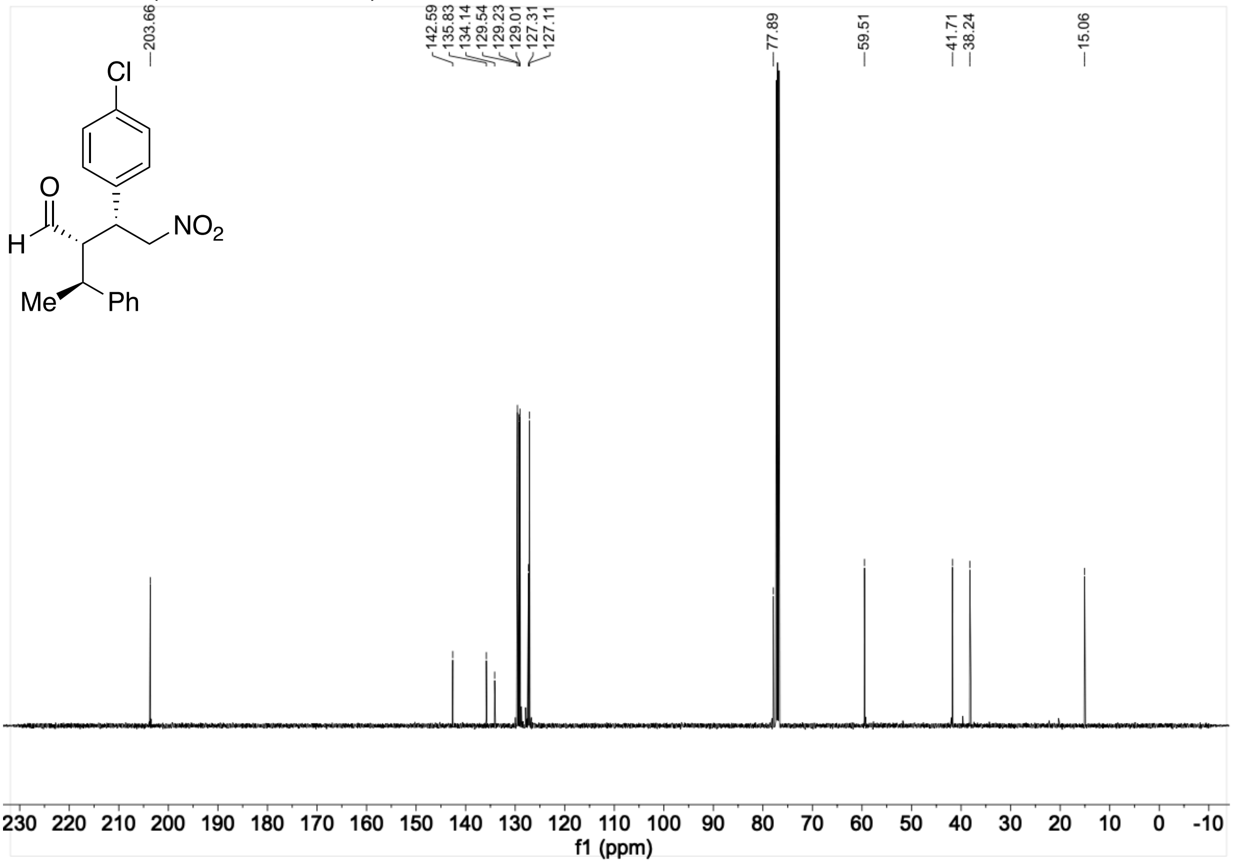

(2*S*,3*R*)-3-(3-Chlorophenyl)-4-nitro-2-((*S*)-1-phenylethyl)butanal (3ad)

^{1}H NMR (500 MHz, $CDCl_3$)

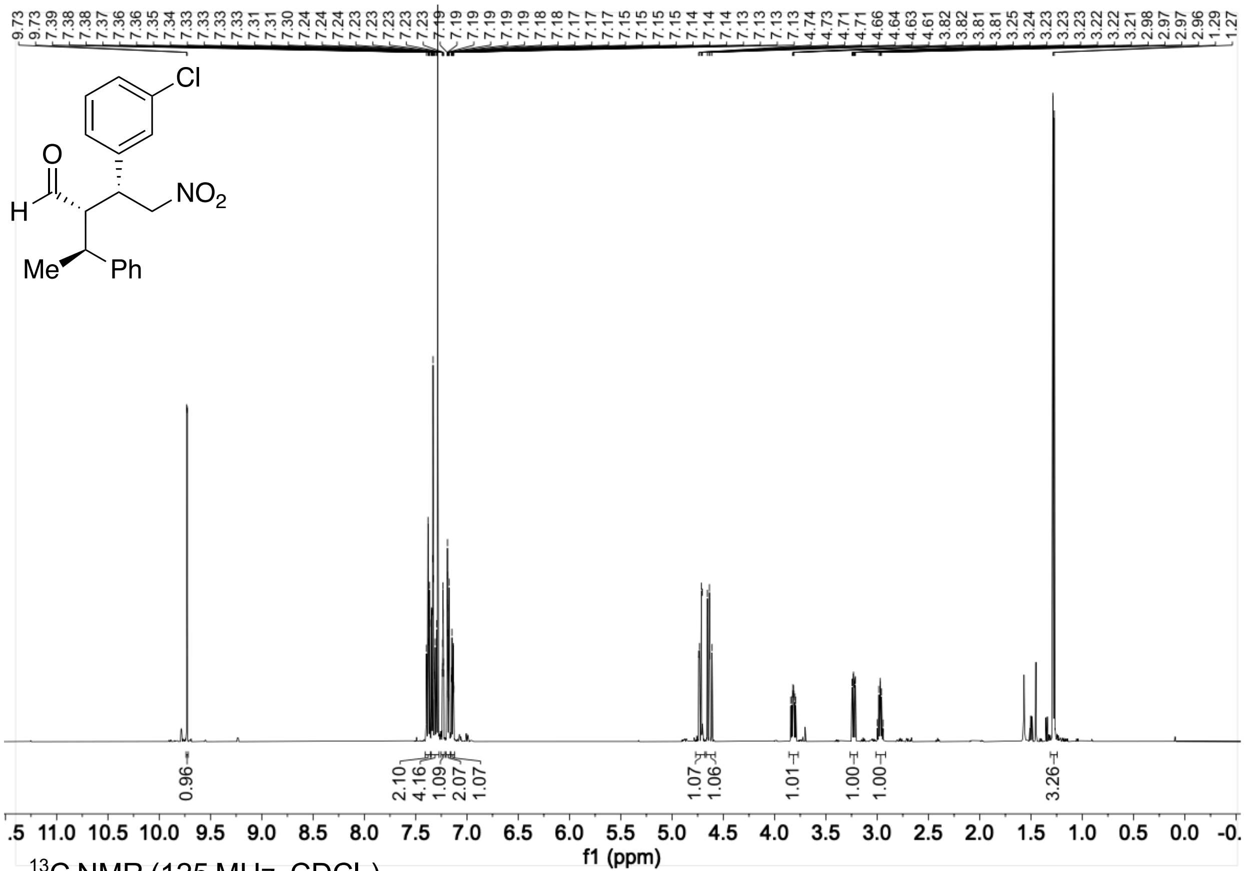

^{13}C NMR (125 MHz, $CDCl_3$)

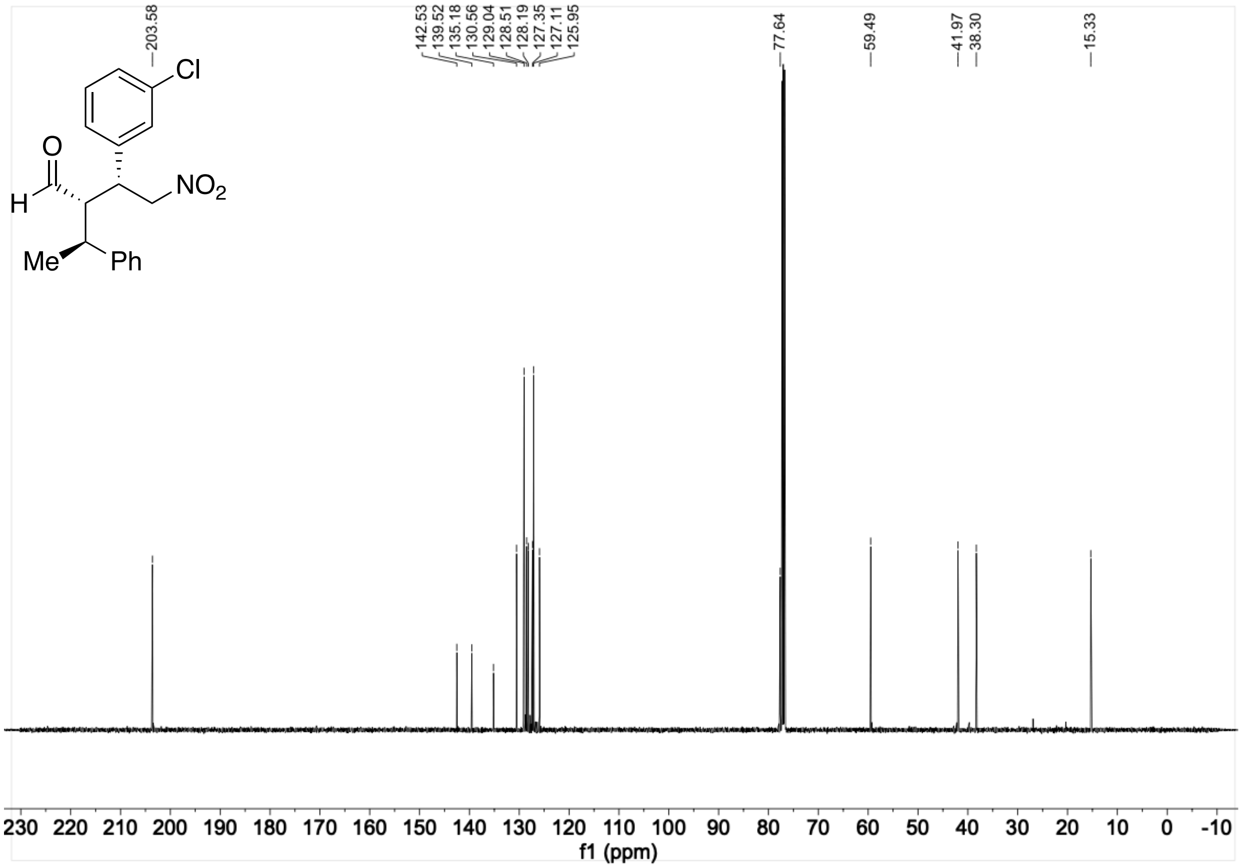

(2*S*,3*R*)-3-(2-Chlorophenyl)-4-nitro-2-((*S*)-1-phenylethyl)butanal (3ae)

^{1}H NMR (500 MHz, $CDCl_3$)

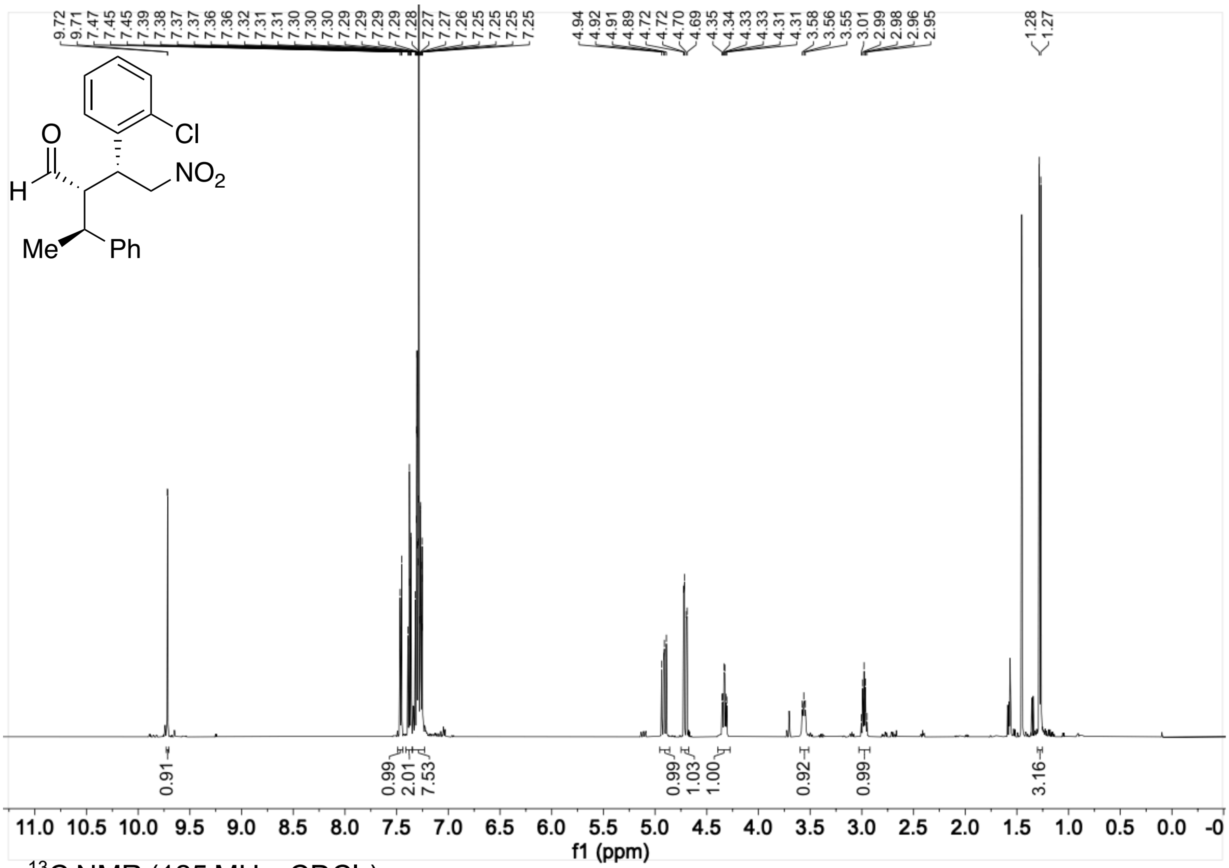

^{13}C NMR (125 MHz, $CDCl_3$)

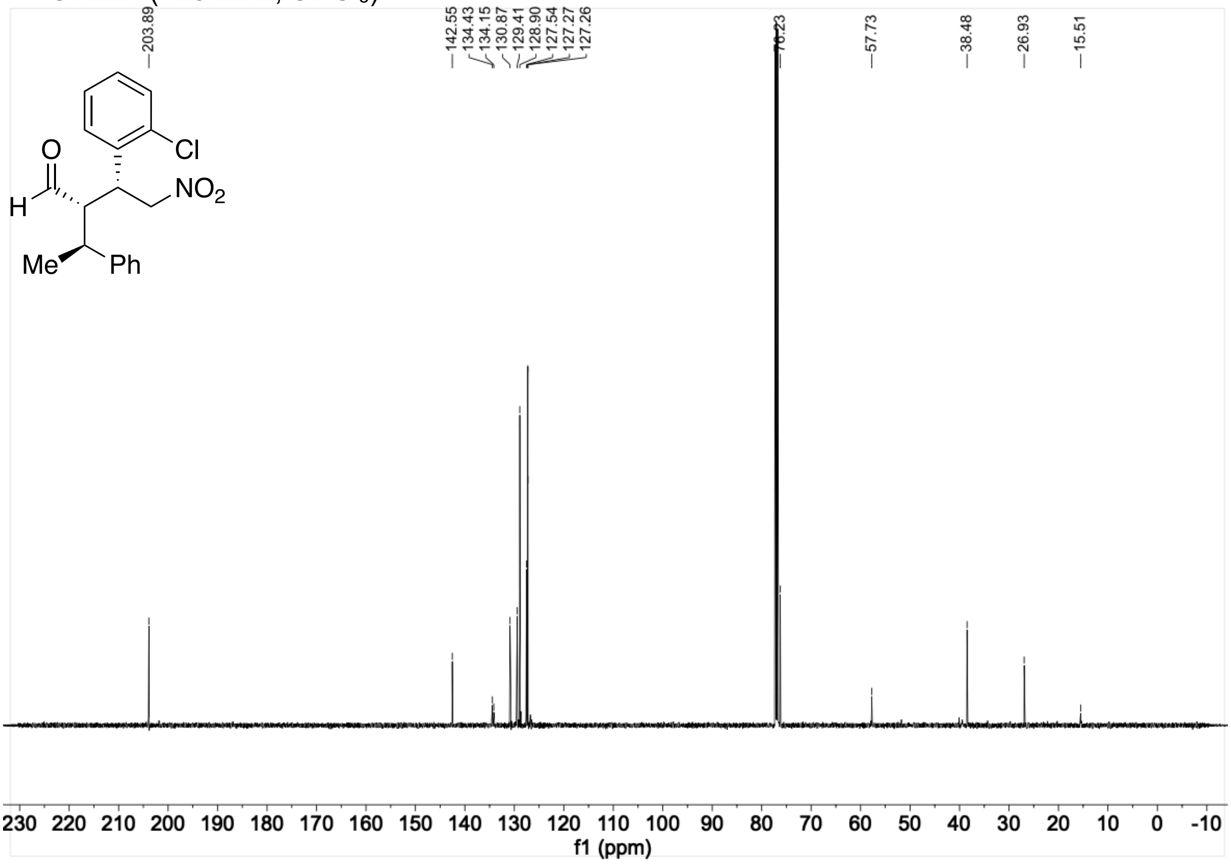

(2*S*,3*R*)-4-Nitro-3-(4-nitrophenyl)-2-((*S*)-1-phenylethyl)butanal (3af)

^{1}H NMR (500 MHz, $CDCl_3$)

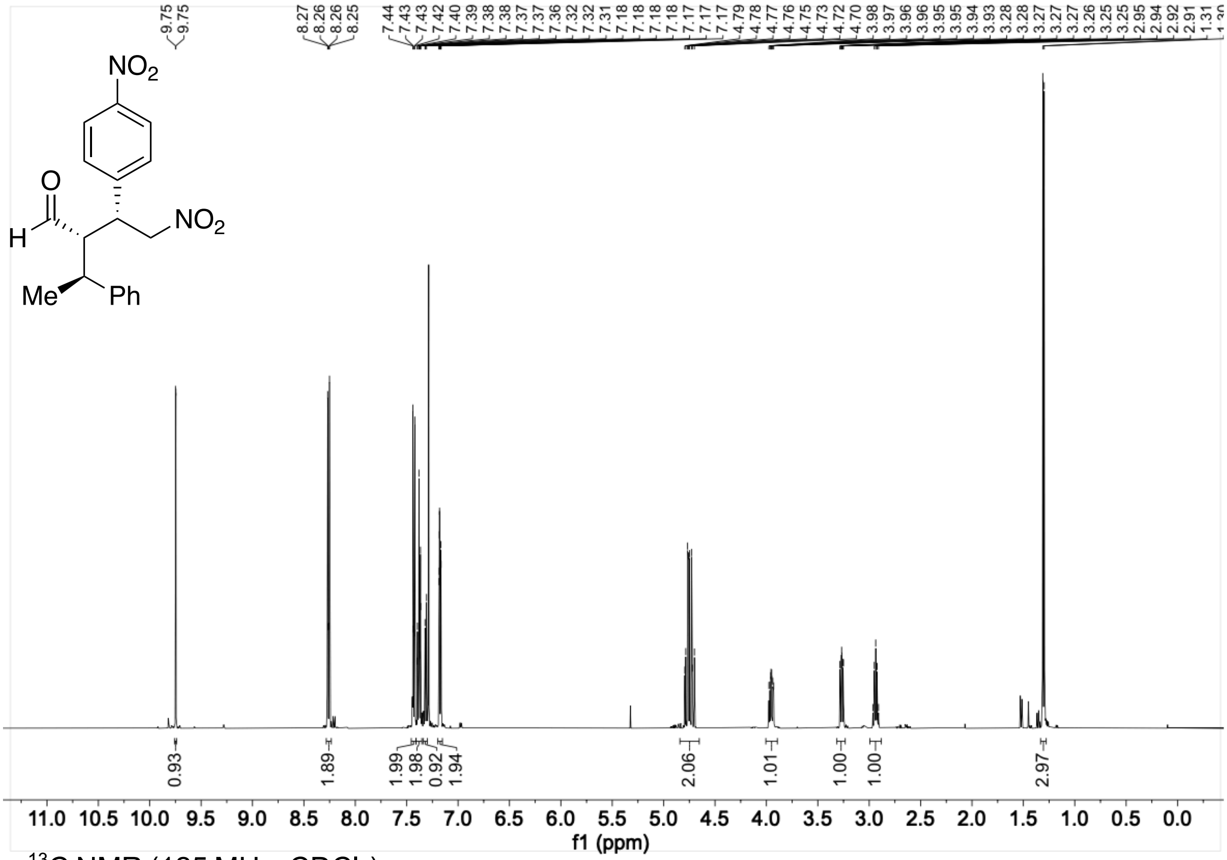

^{13}C NMR (125 MHz, $CDCl_3$)

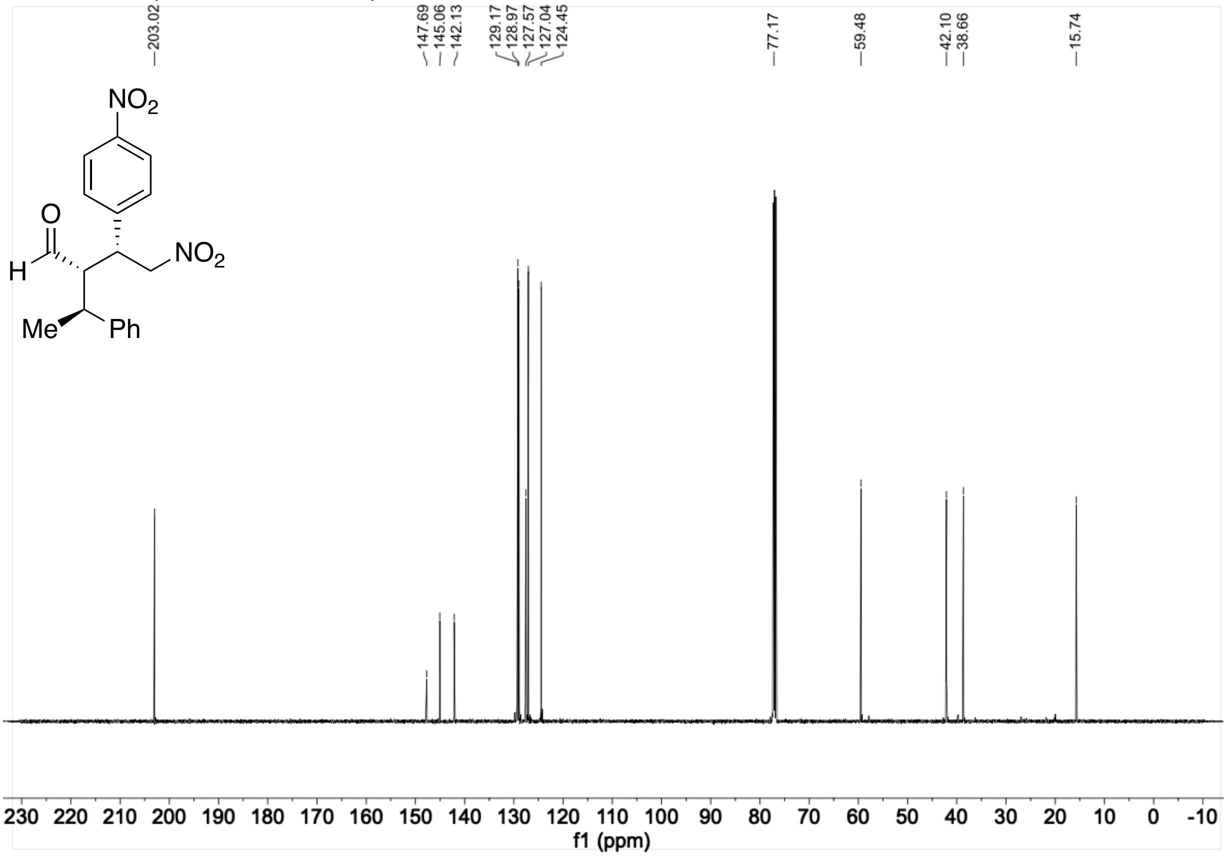

(2*S*,3*R*)-4-nitro-3-(3-nitrophenyl)-2-((*S*)-1-phenylethyl)butanal (3ag)

^{1}H NMR (500 MHz, $CDCl_3$)

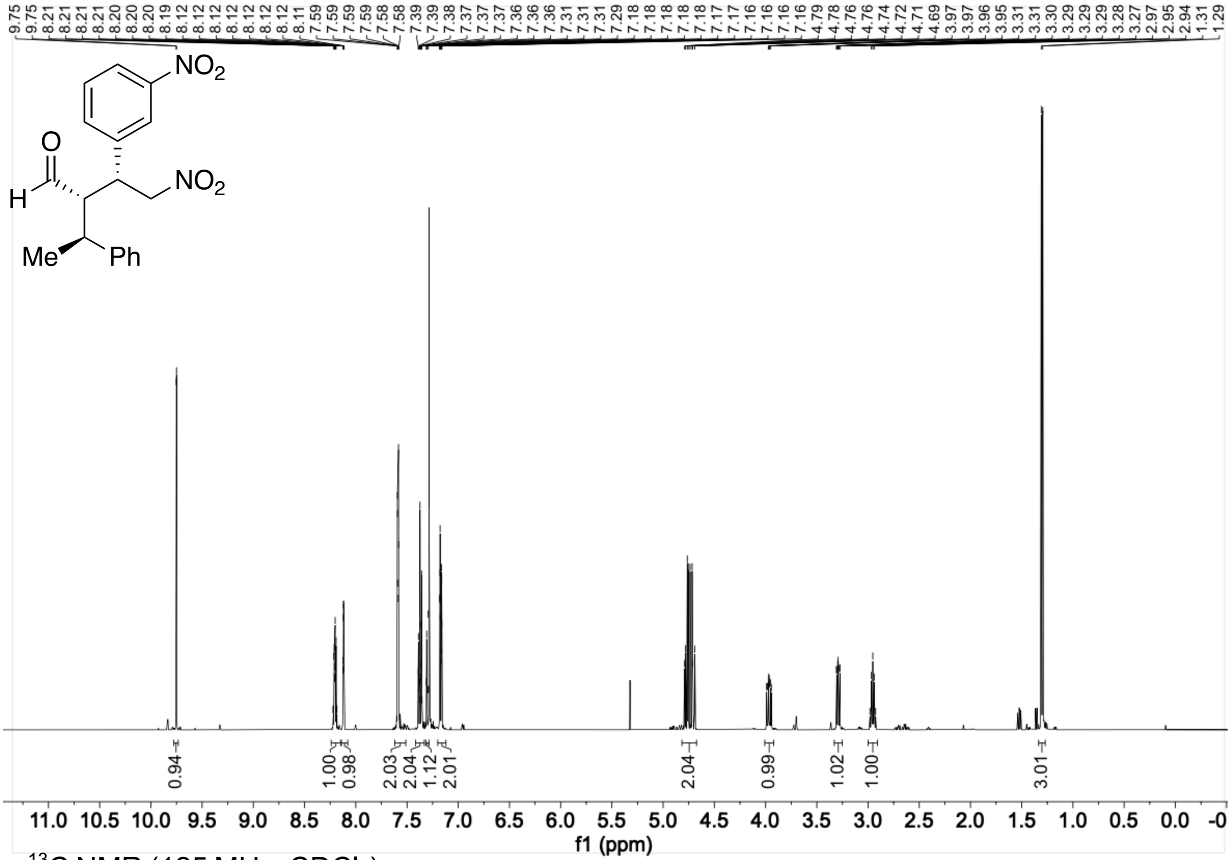

^{13}C NMR (125 MHz, $CDCl_3$)

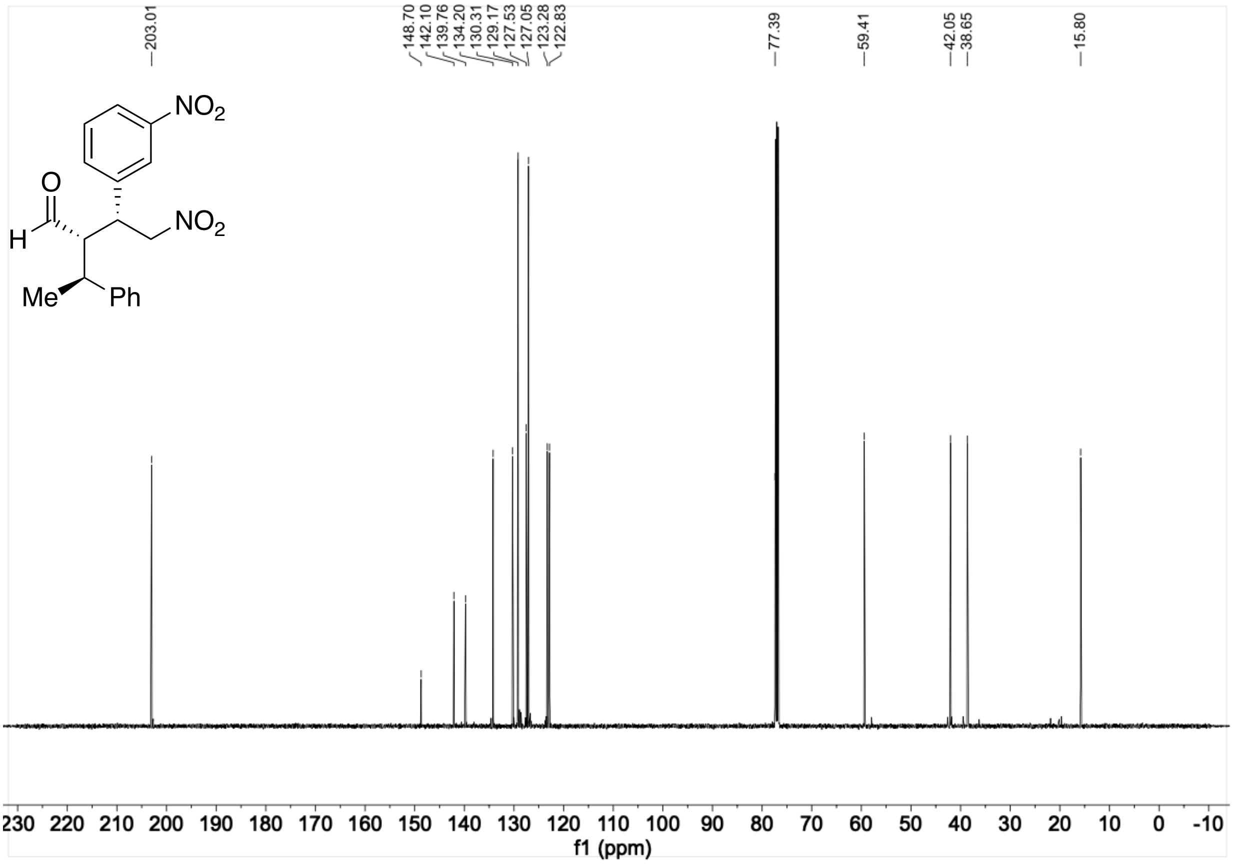

(2*S*,3*R*)-4-nitro-3-(2-nitrophenyl)-2-((*S*)-1-phenylethyl)butanal (3ah)

^{1}H NMR (500 MHz, $CDCl_3$)

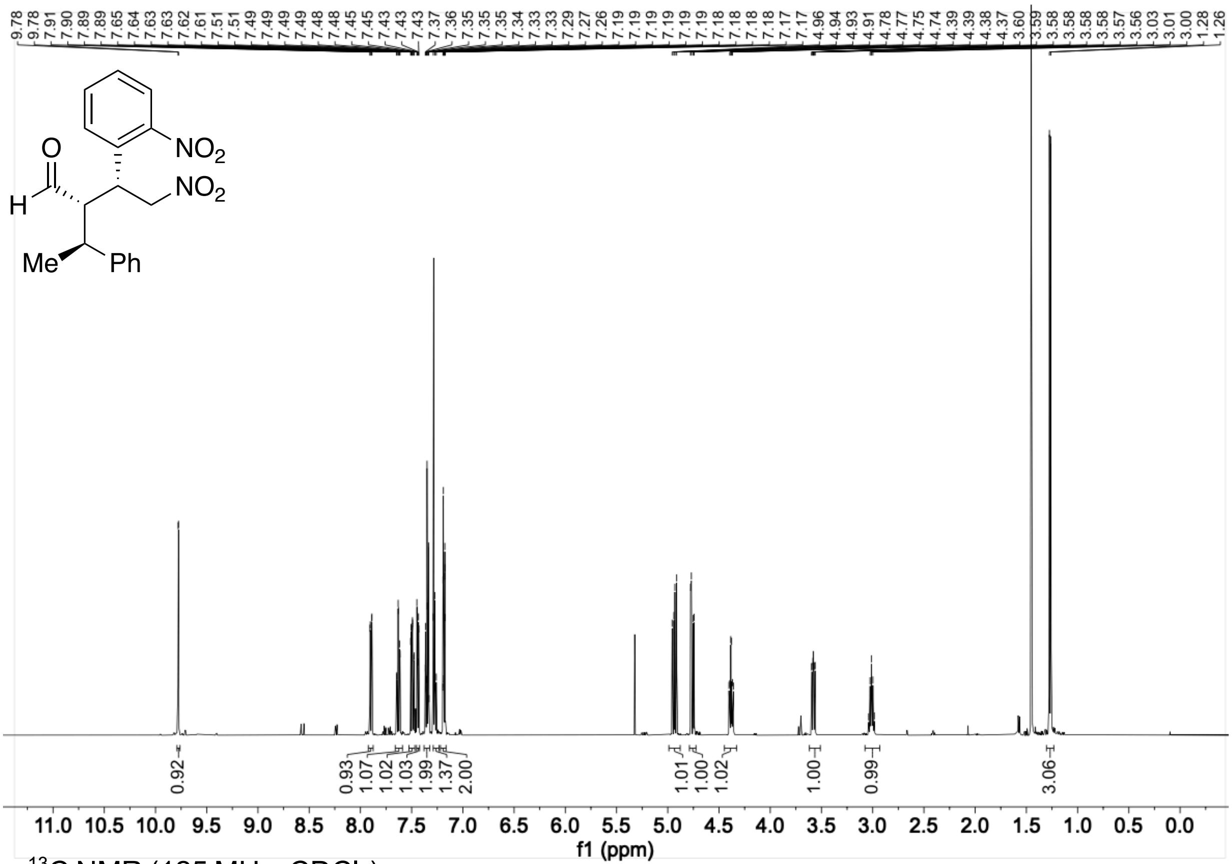

^{13}C NMR (125 MHz, $CDCl_3$)

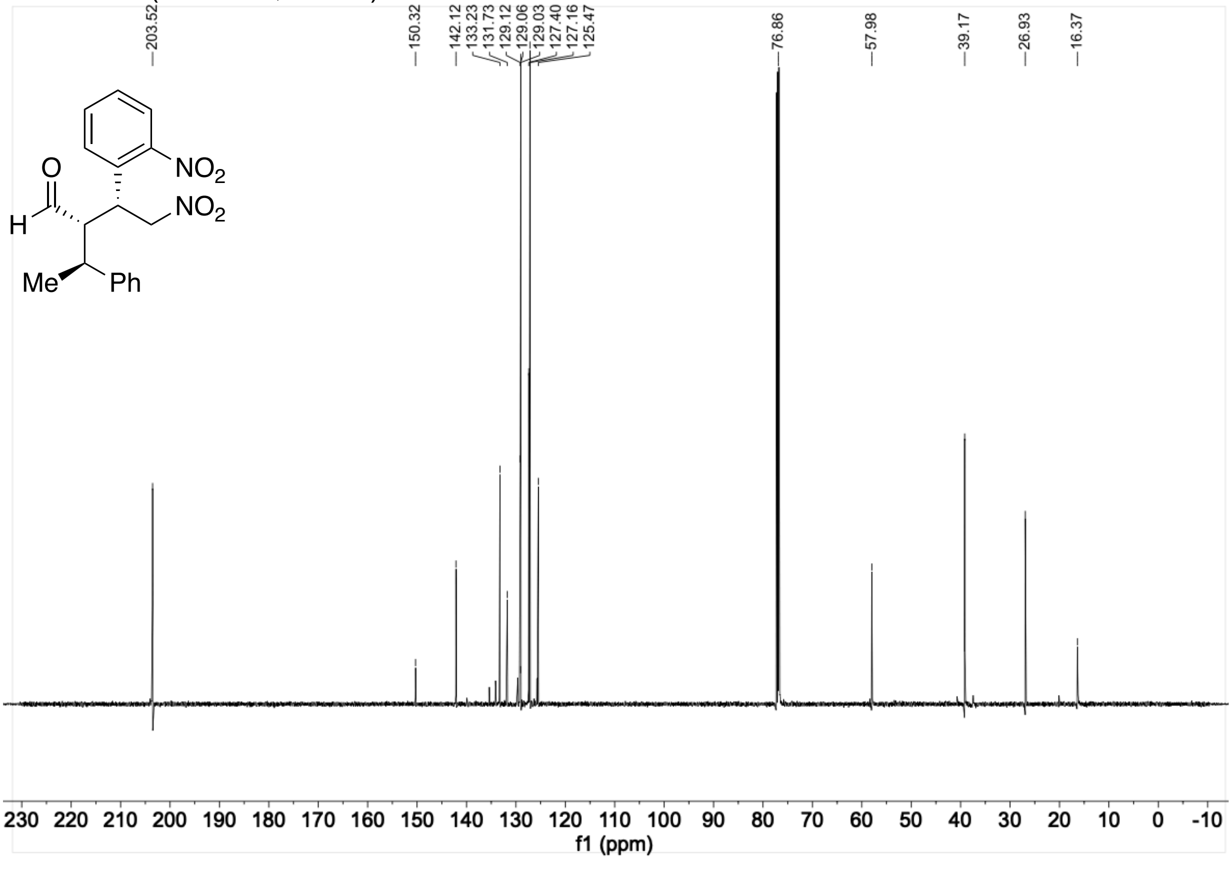

(2*S*,3*R*)-3-(4-Bromophenyl)-4-nitro-2-((*S*)-1-phenylethyl)butanal (3ai)

^{1}H NMR (500 MHz, $CDCl_3$)

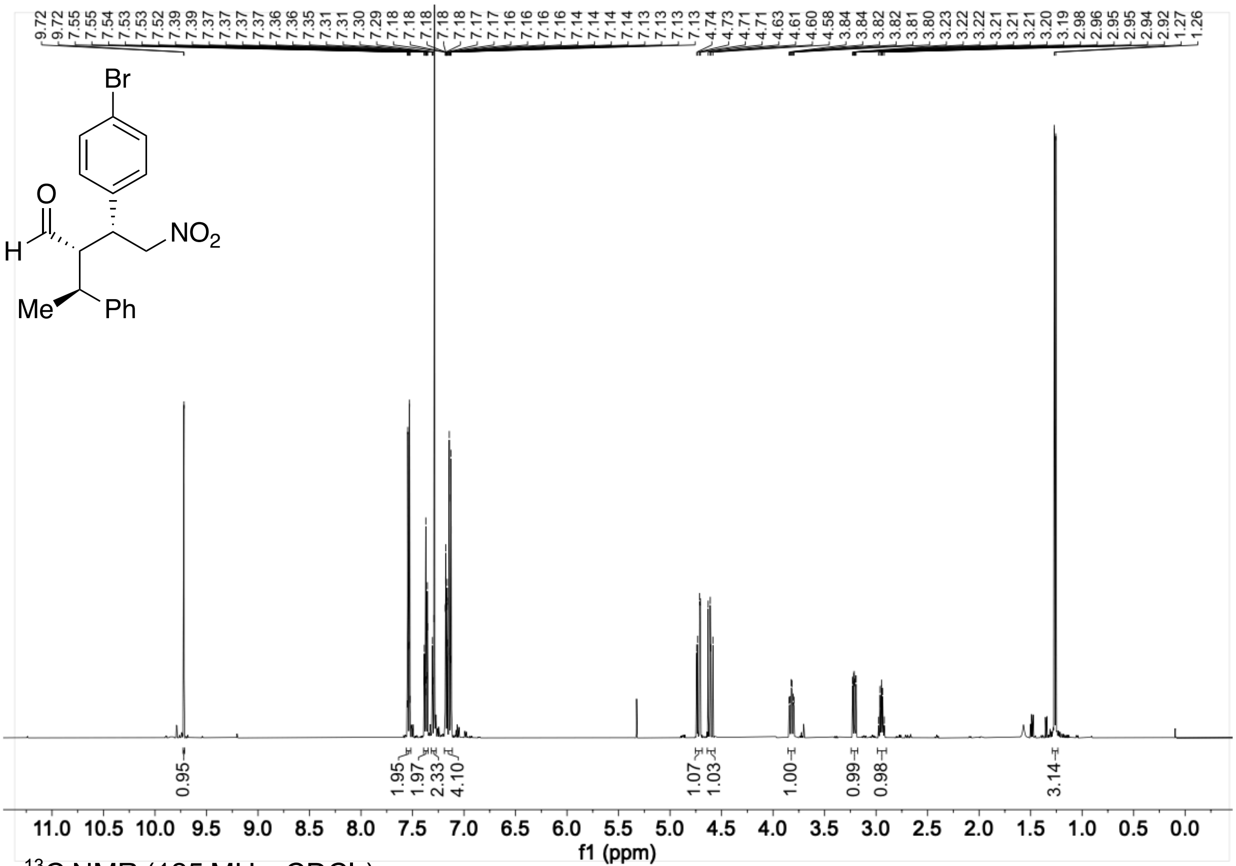

^{13}C NMR (125 MHz, $CDCl_3$)

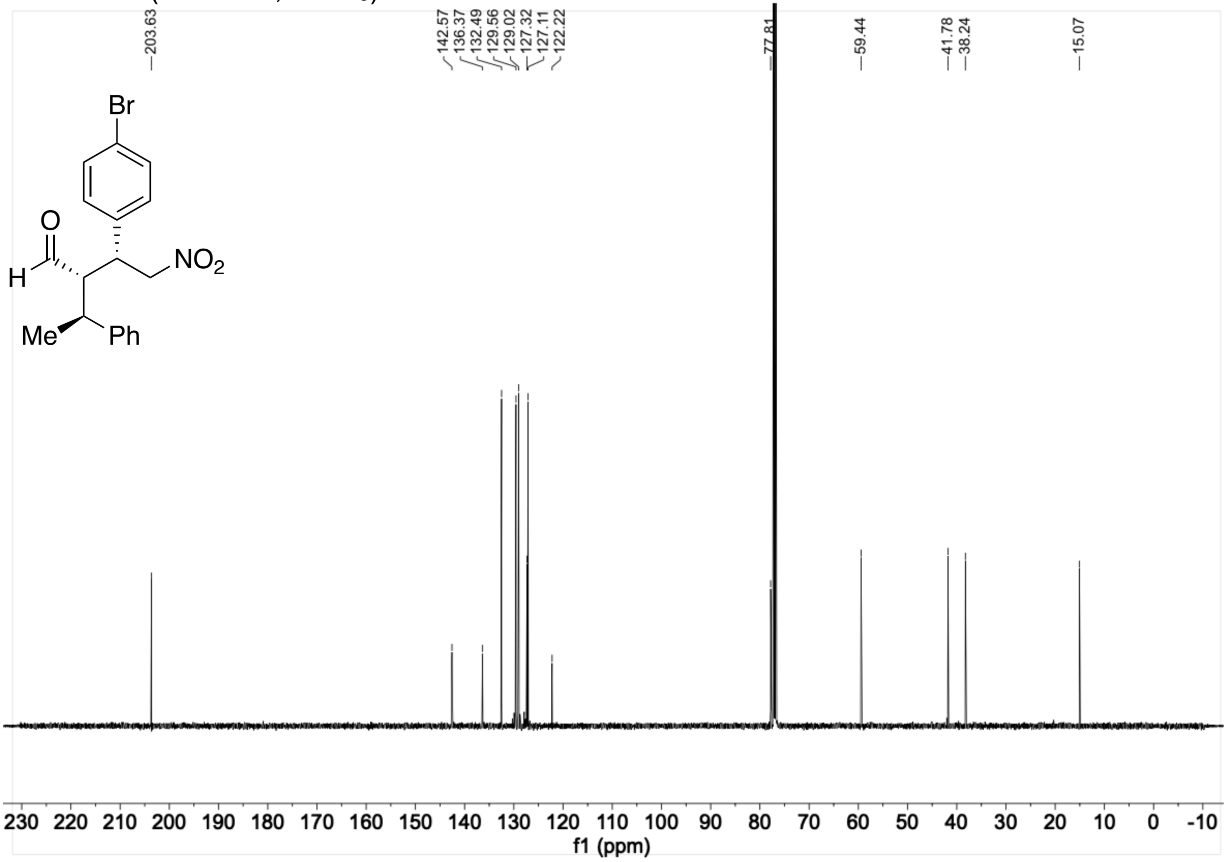

(2*S*,3*R*)-3-(4-Methoxyphenyl)-4-nitro-2-((*S*)-1-phenylethyl)butanal (3aj)

^{1}H NMR (500 MHz, $CDCl_3$)

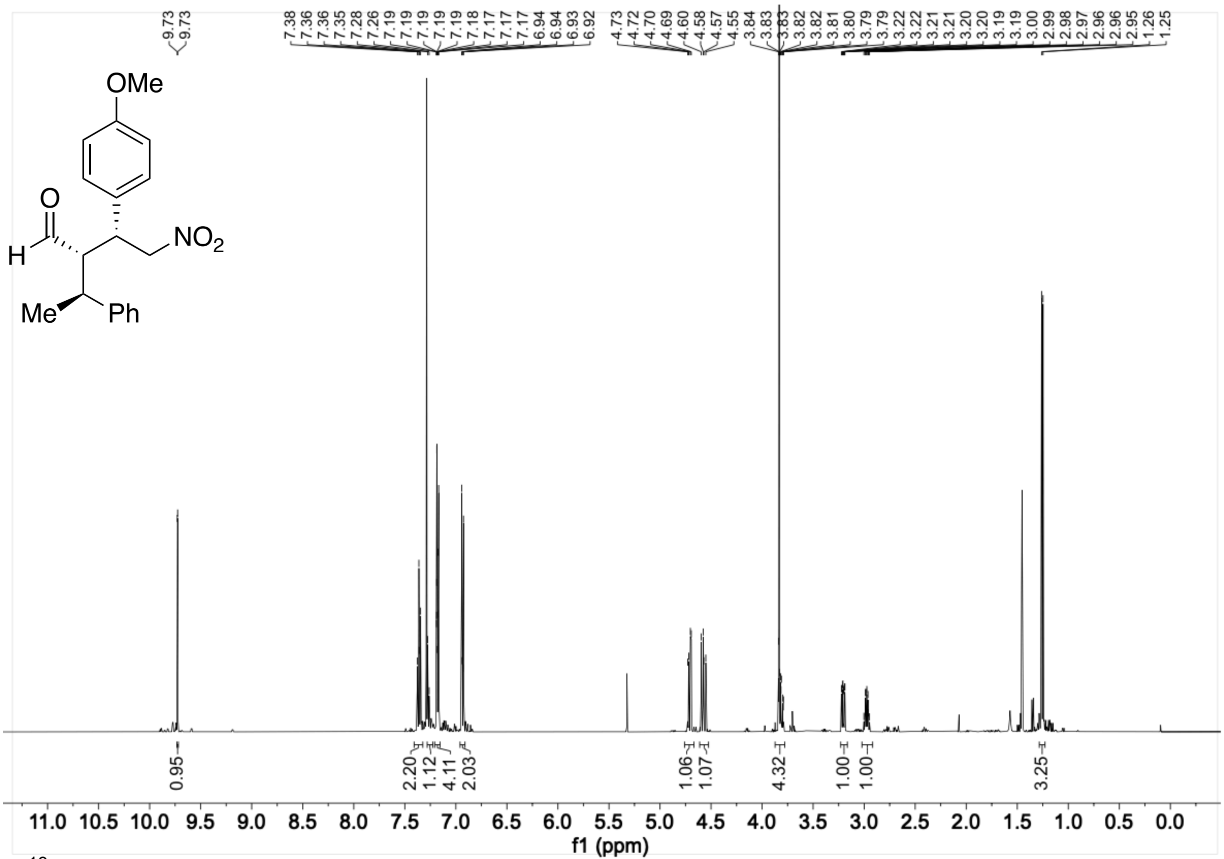

^{13}C NMR (125 MHz, $CDCl_3$)

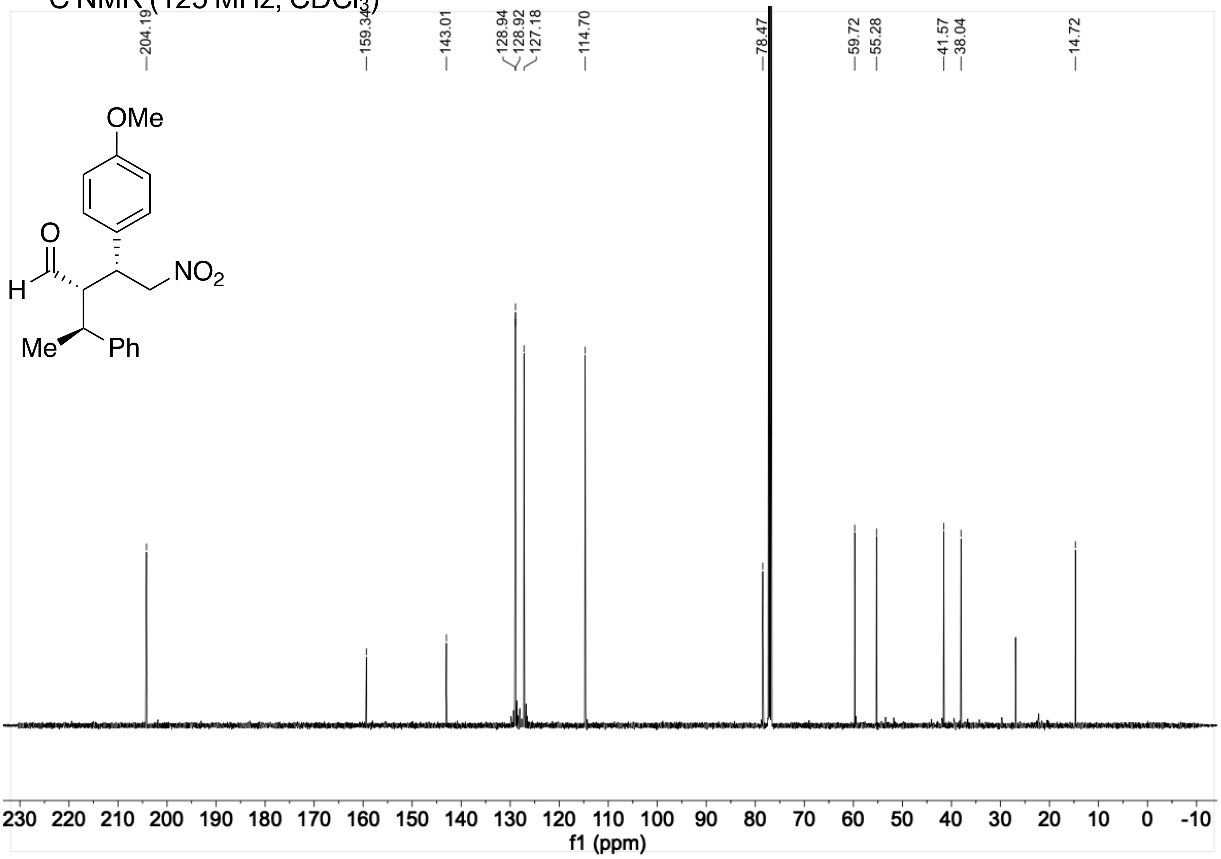

(2*S*,3*R*)-3-(3-Methoxyphenyl)-4-nitro-2-((*S*)-1-phenylethyl)butanal (3ak)

^{1}H NMR (500 MHz, $CDCl_3$)

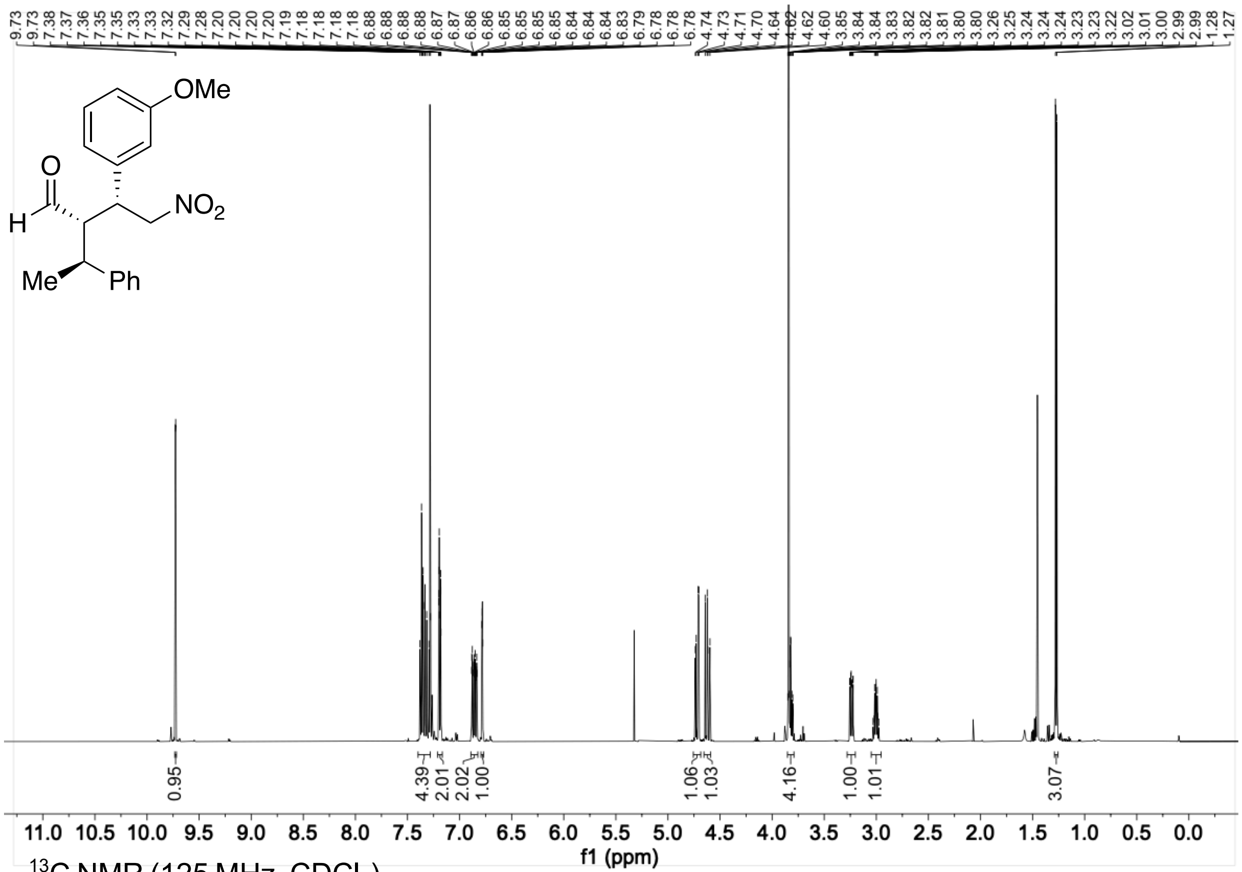

^{13}C NMR (125 MHz, $CDCl_3$)

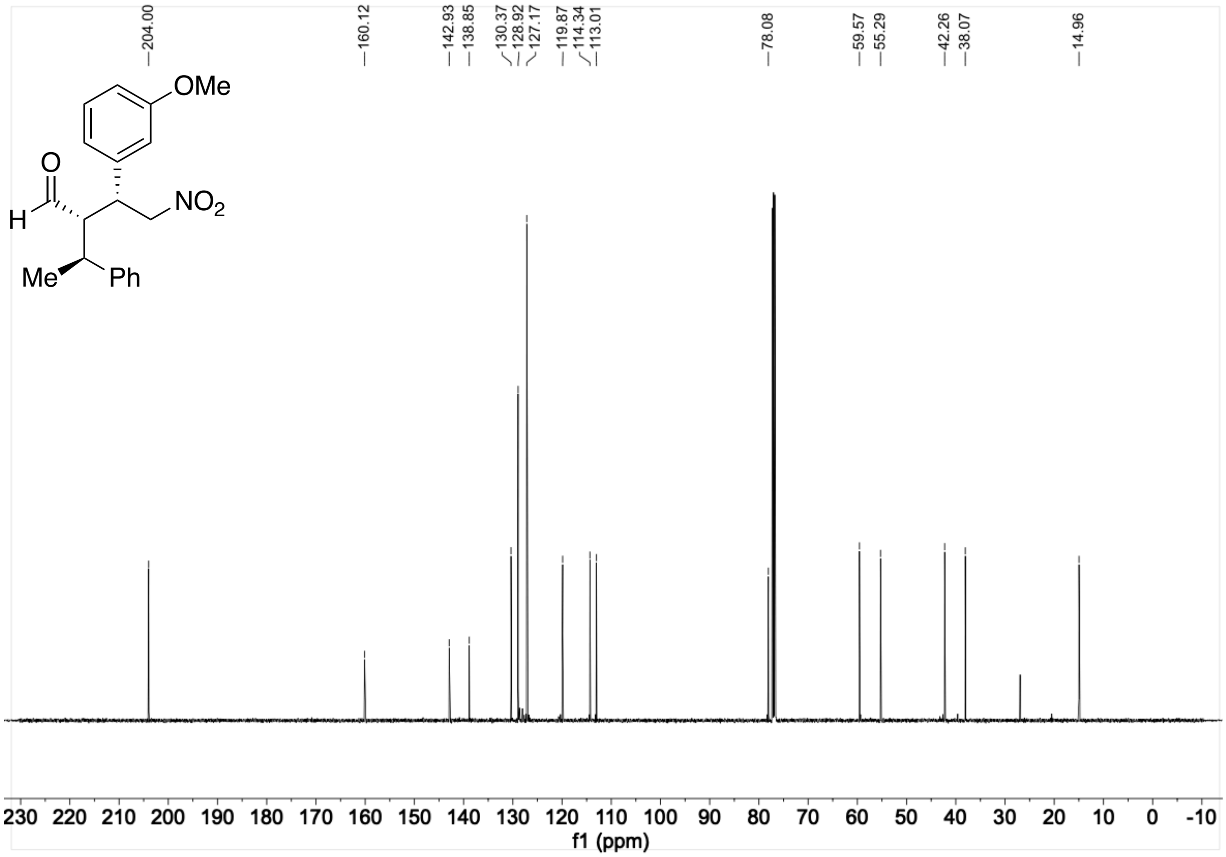

(2*S*,3*R*)-3-(2-Methoxyphenyl)-4-nitro-2-((*S*)-1-phenylethyl)butanal (3al)

^{1}H NMR (500 MHz, $CDCl_3$)

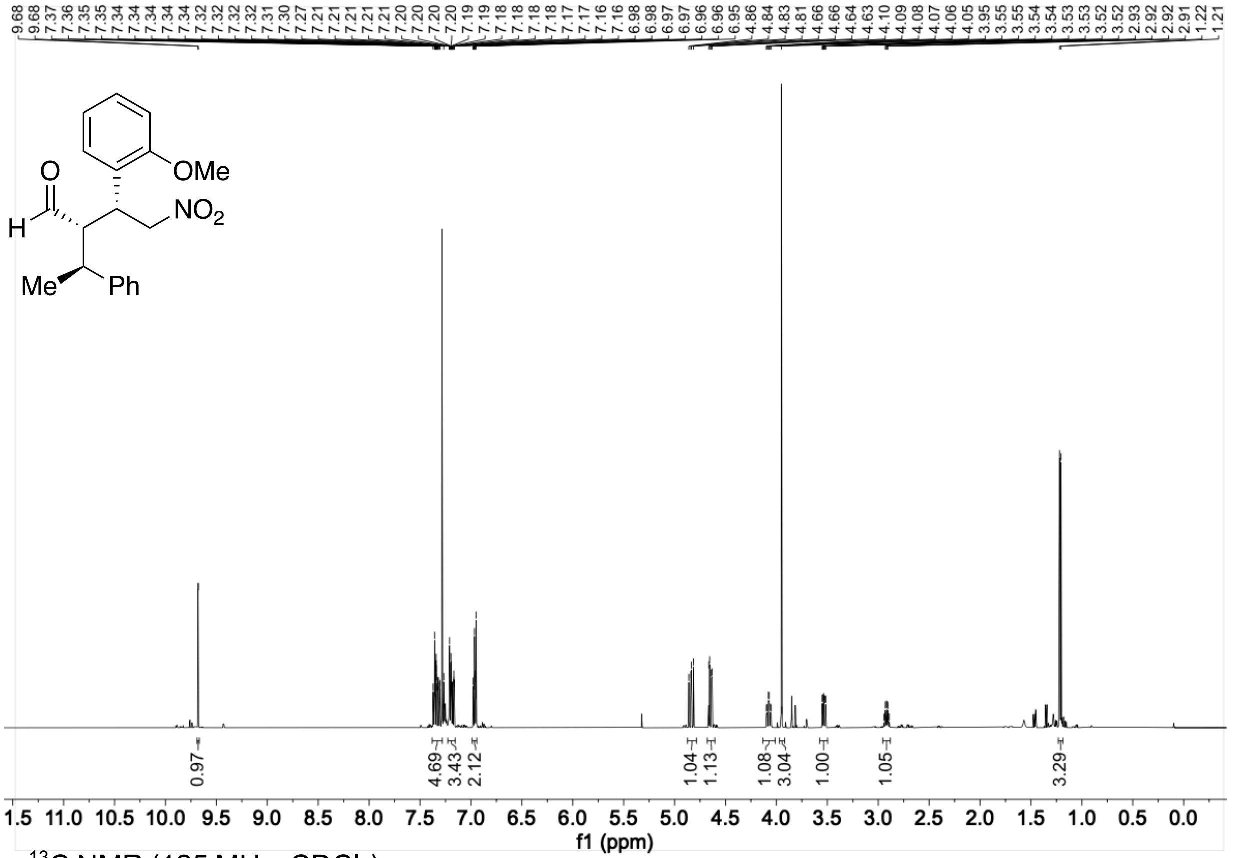

^{13}C NMR (125 MHz, $CDCl_3$)

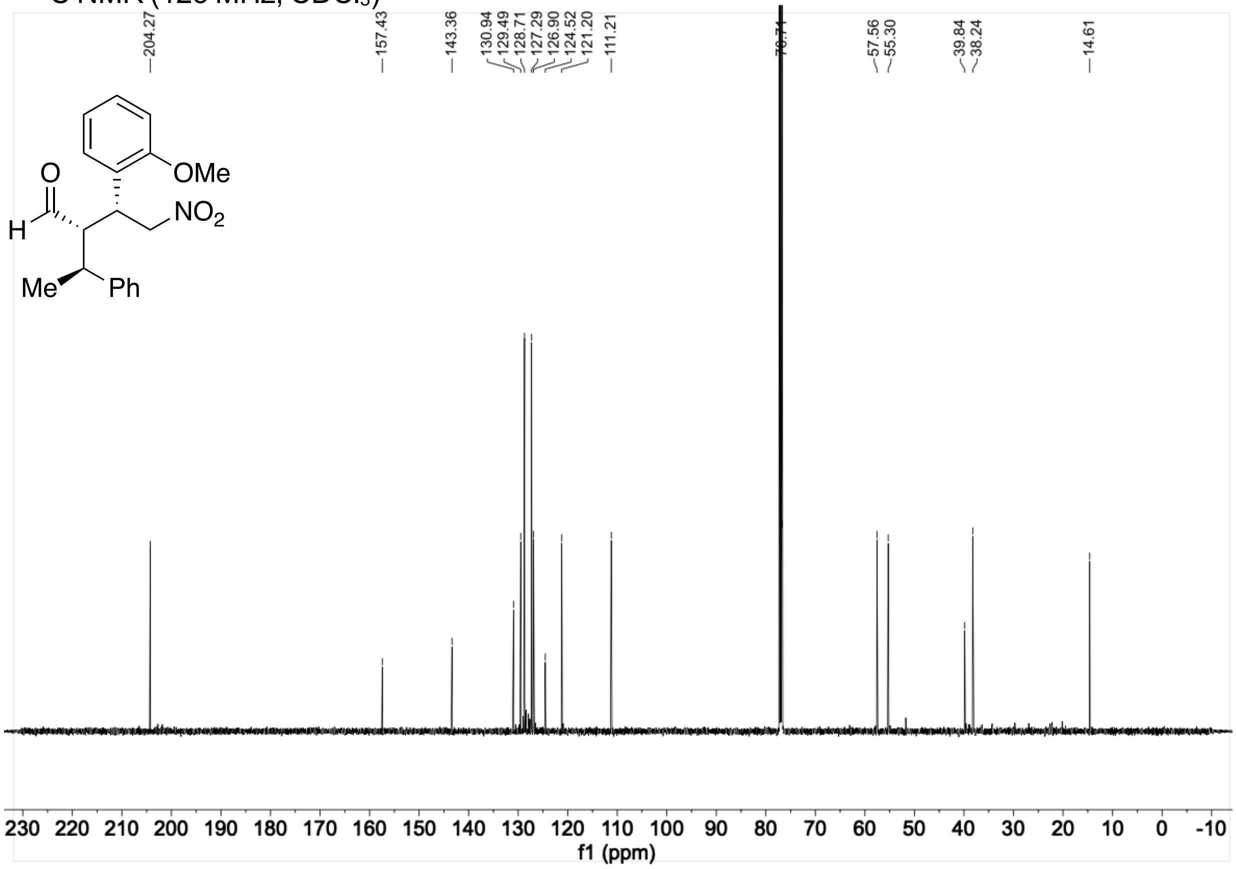

(2*S*,3*R*)-4-Nitro-2-((*S*)-1-phenylethyl)-3-(*p*-tolyl)butanal (3am)

^{1}H NMR (400 MHz, CDCl$_3$)

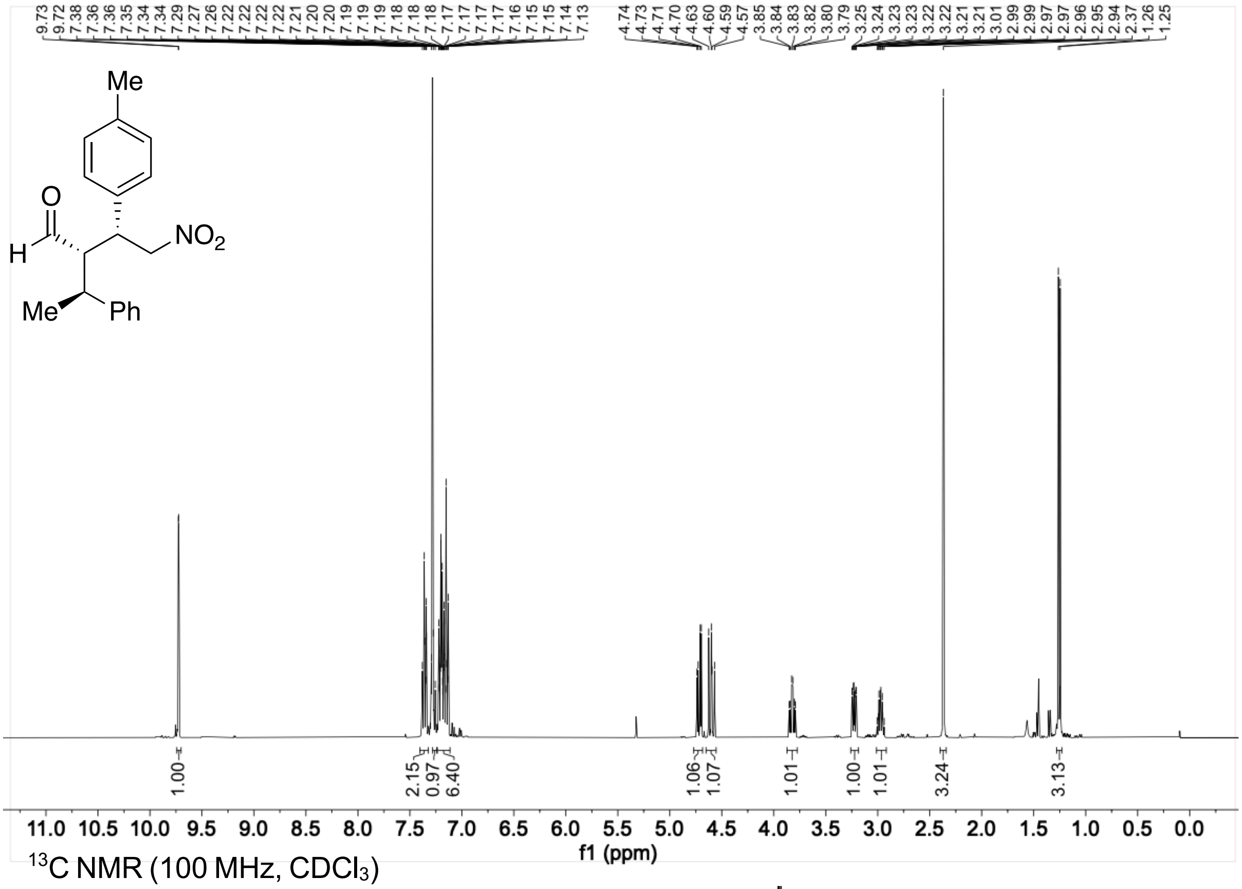

^{13}C NMR (100 MHz, CDCl$_3$)

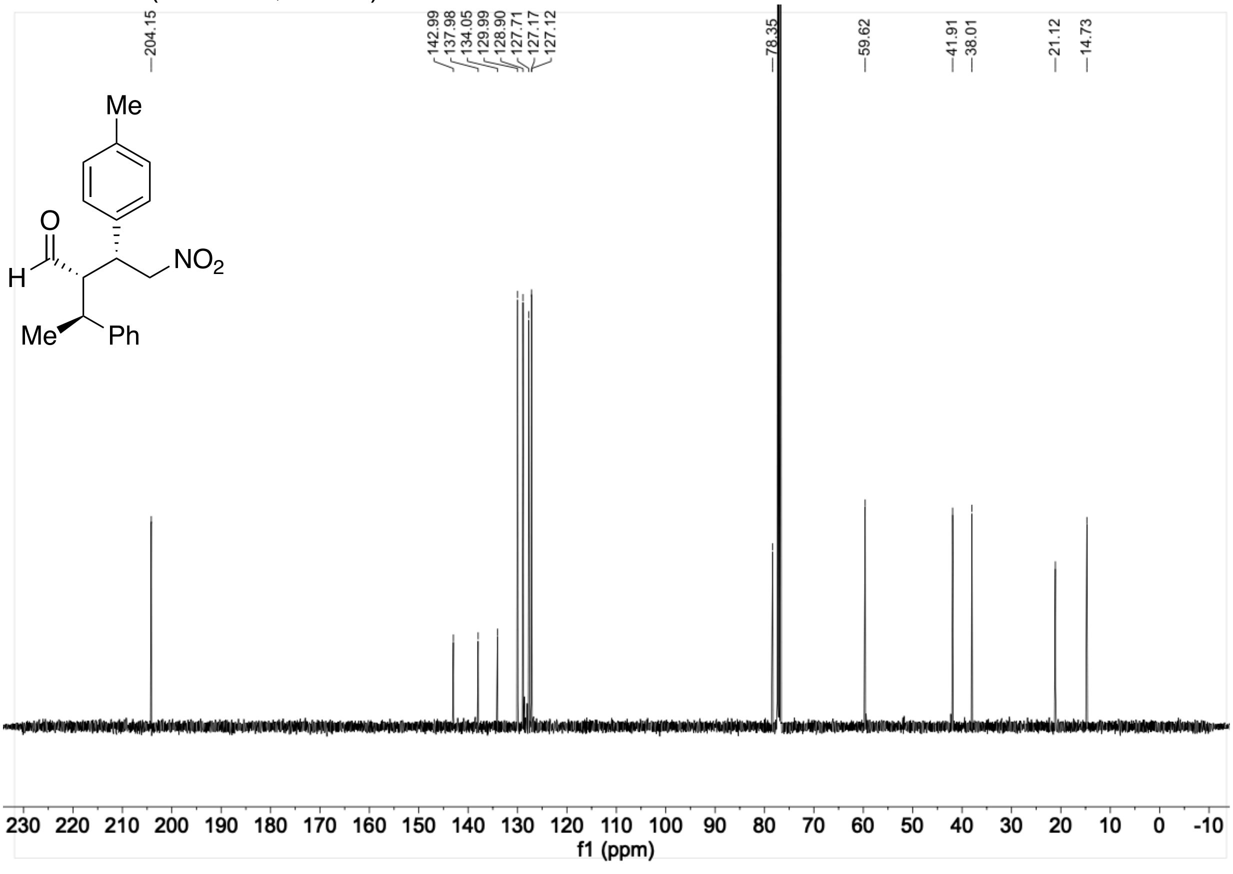

(2*S*,3*S*)-3-(Furan-2-yl)-4-nitro-2-((*S*)-1-phenylethyl)butanal (3an)

^{1}H NMR (400 MHz, $CDCl_3$)

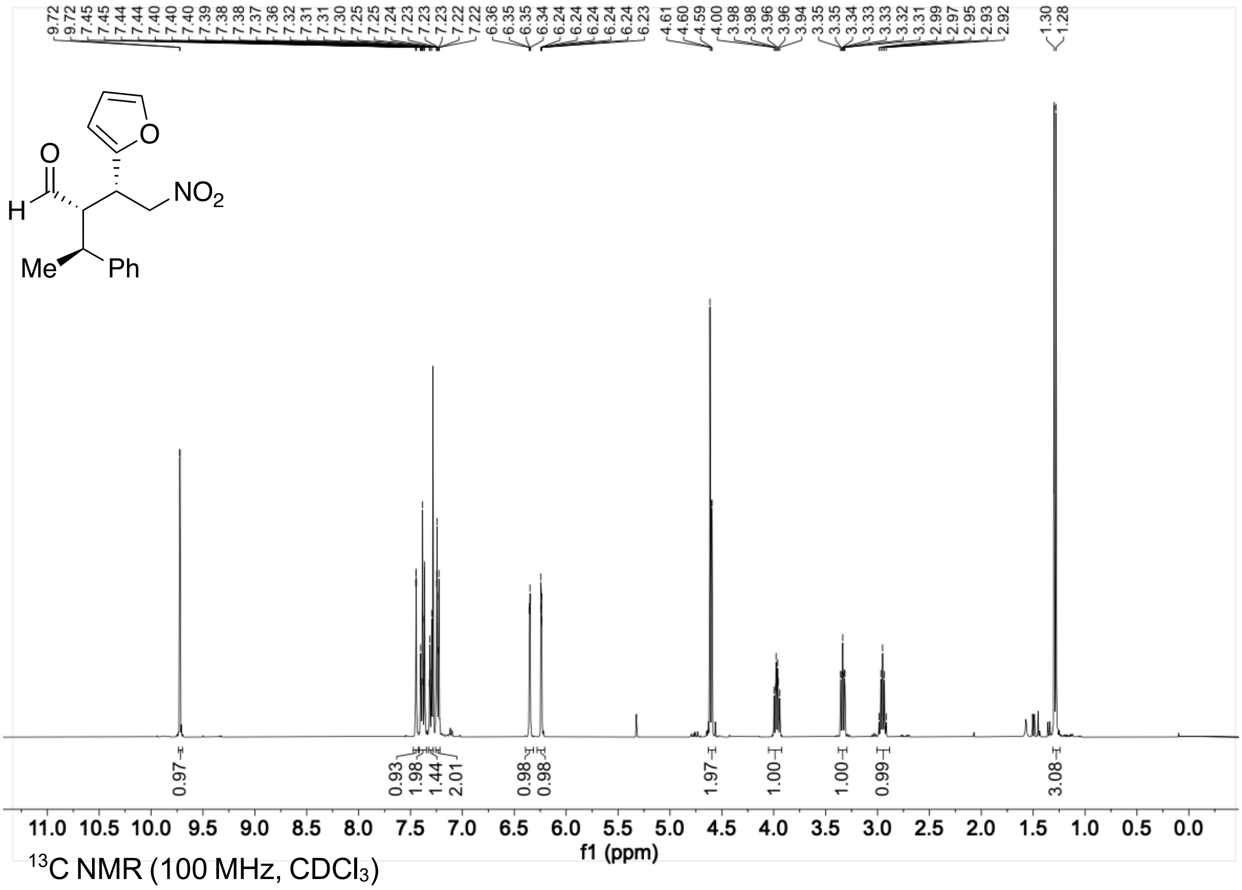

^{13}C NMR (100 MHz, $CDCl_3$)

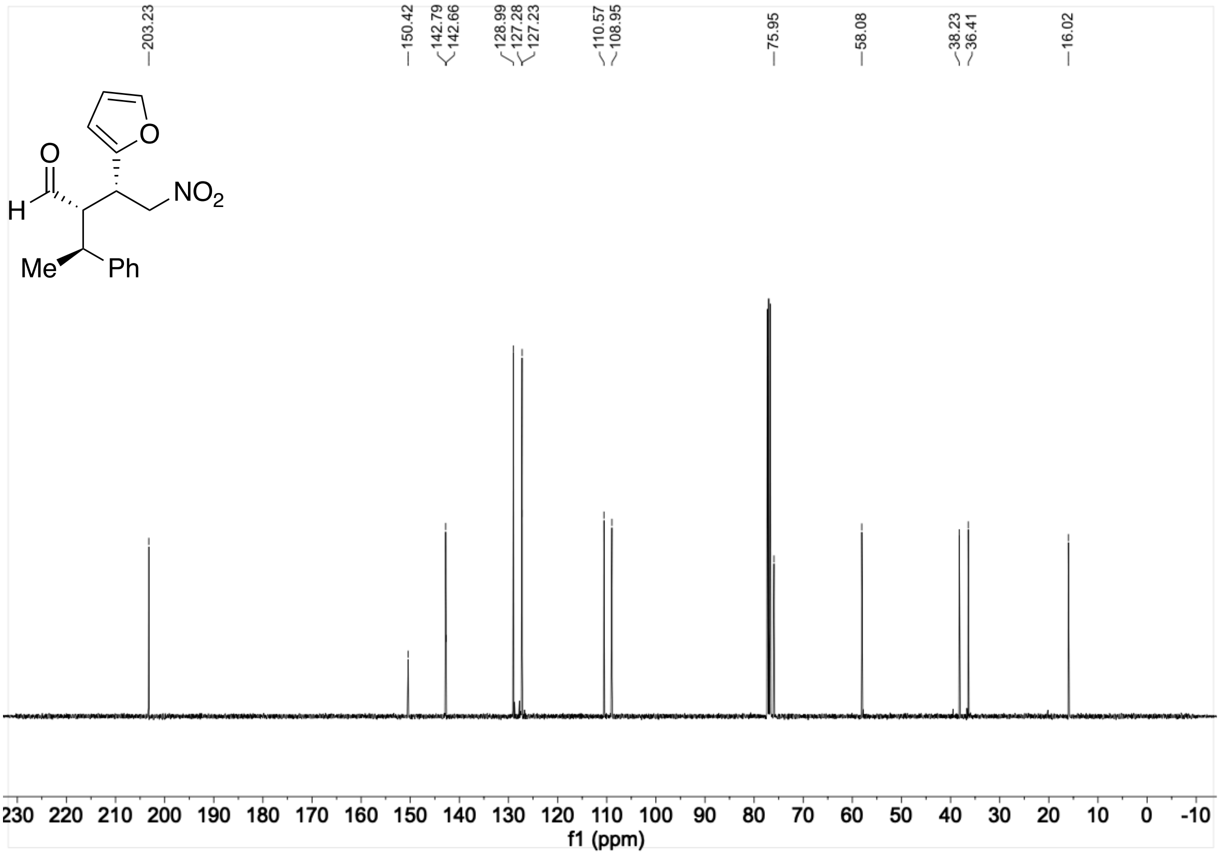

(2*S*,3*S*)-4-Nitro-2-((*S*)-1-phenylethyl)-3-(thiophen-2-yl)butanal (3ao)

^{1}H NMR (400 MHz, $CDCl_3$)

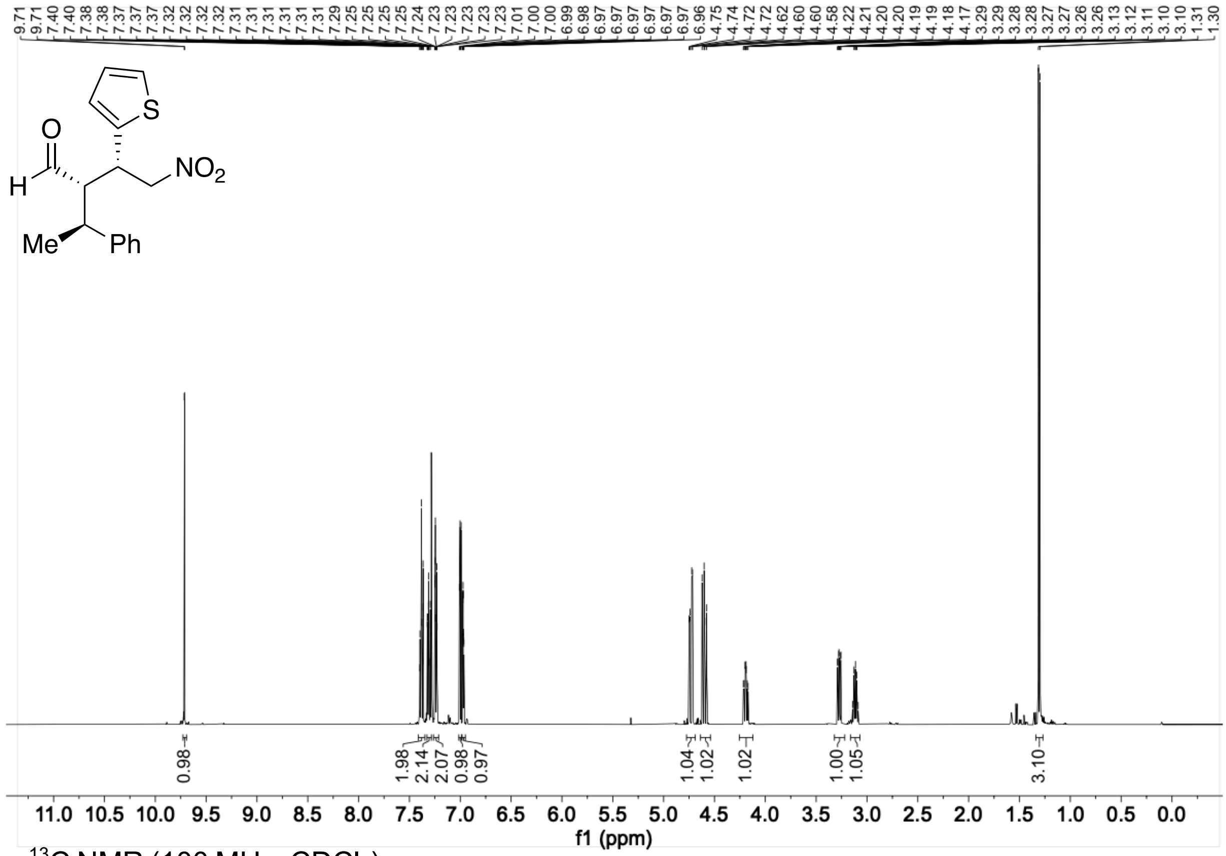

^{13}C NMR (100 MHz, $CDCl_3$)

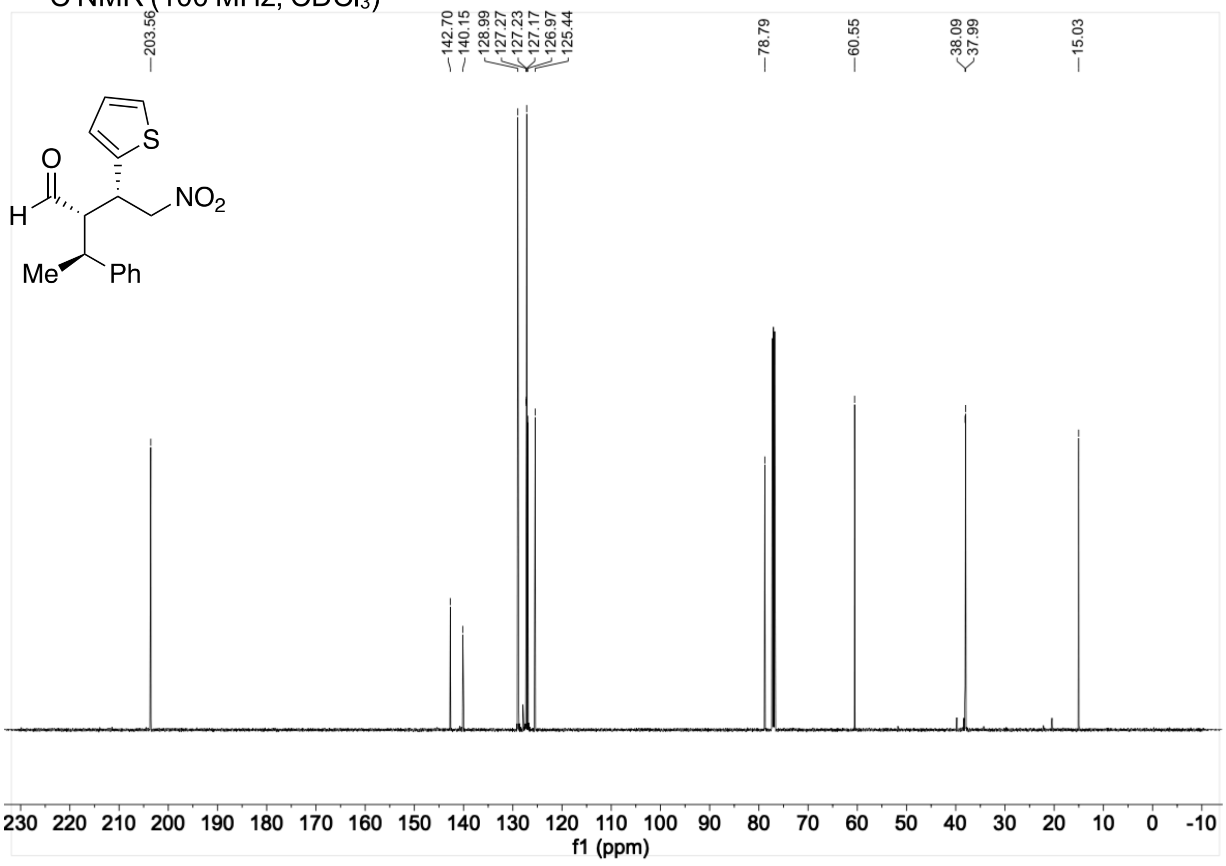

(2*S*,3*R*)-4-Nitro-2-((*S*)-1-phenylethyl)-3-(pyridin-3-yl)butanal (3ap)

^{1}H NMR (400 MHz, $CDCl_3$)

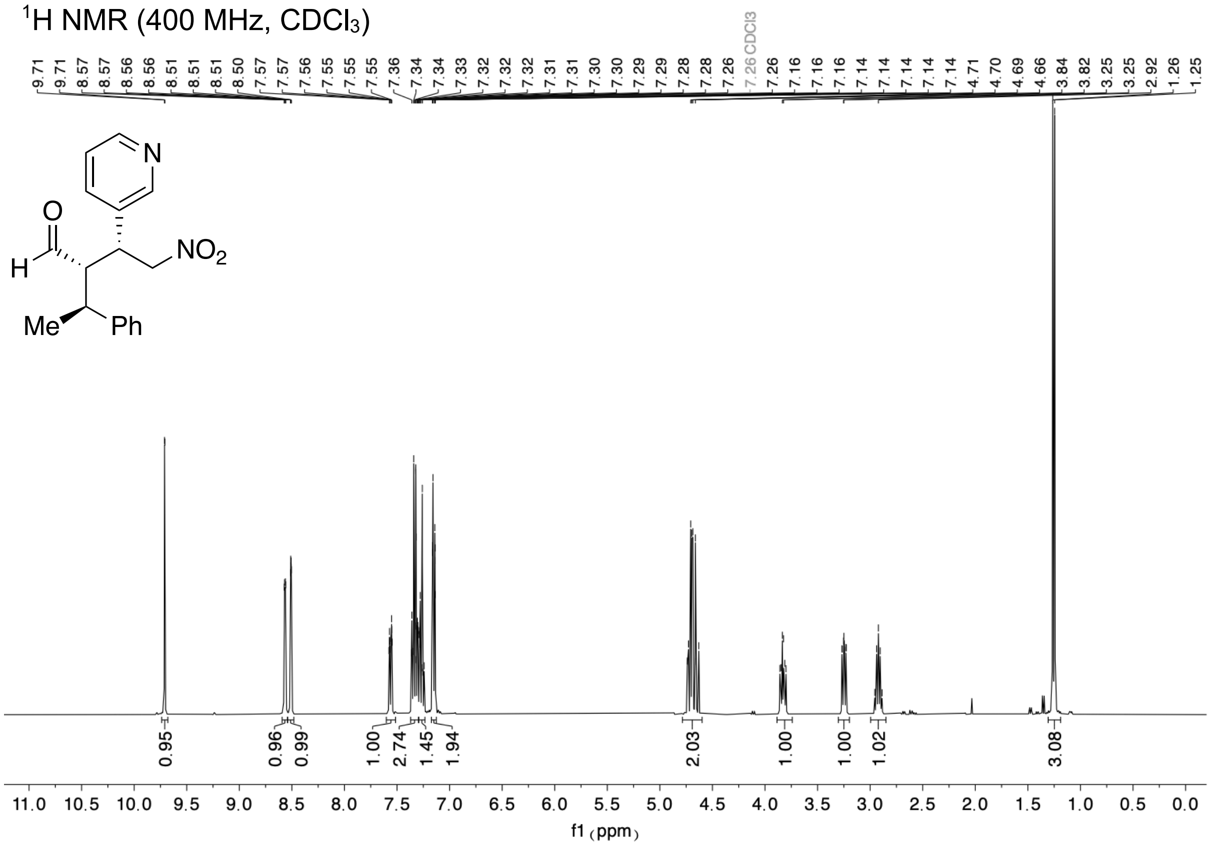

^{13}C NMR (100 MHz, $CDCl_3$)

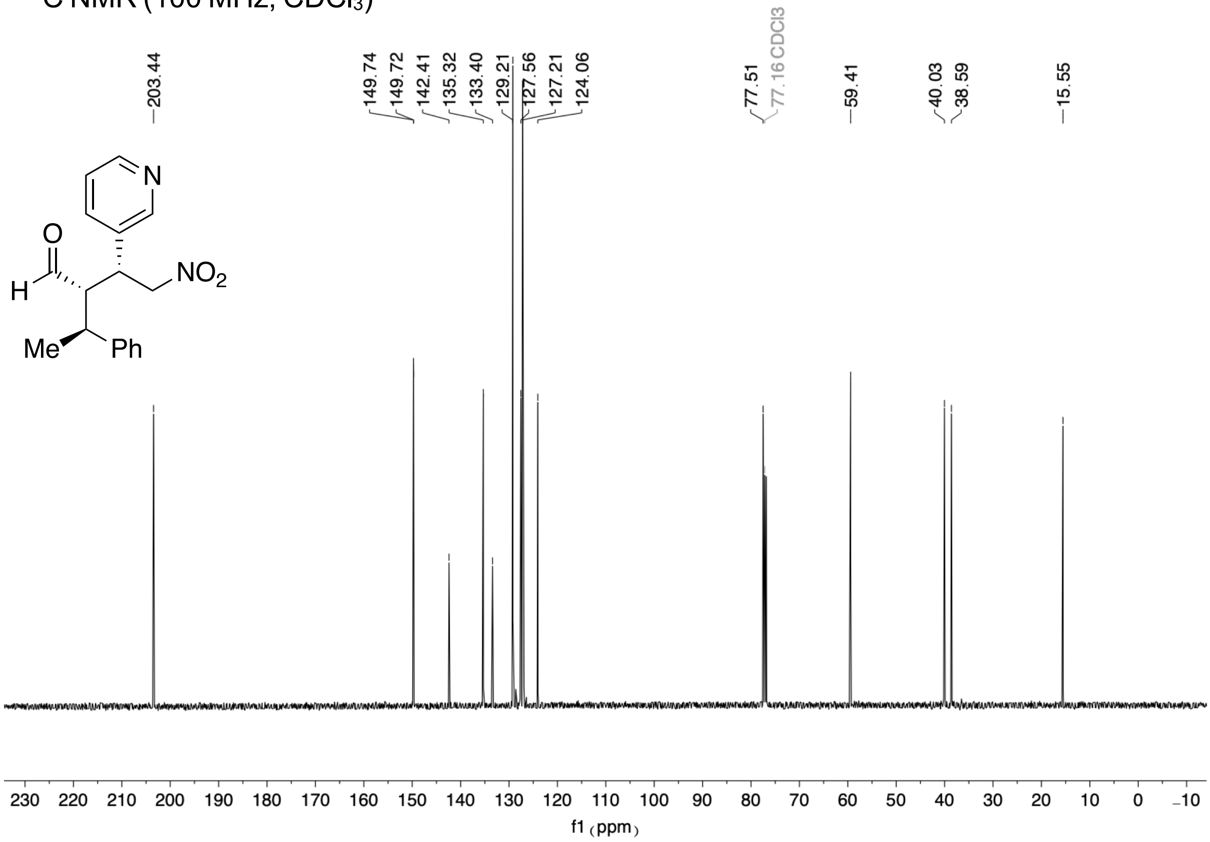

(2*S*,3*S*)-4,4-Dimethoxy-3-(nitromethyl)-2-((*S*)-1-phenylethyl)butanal (3aq)

^{1}H NMR (500 MHz, $CDCl_3$)

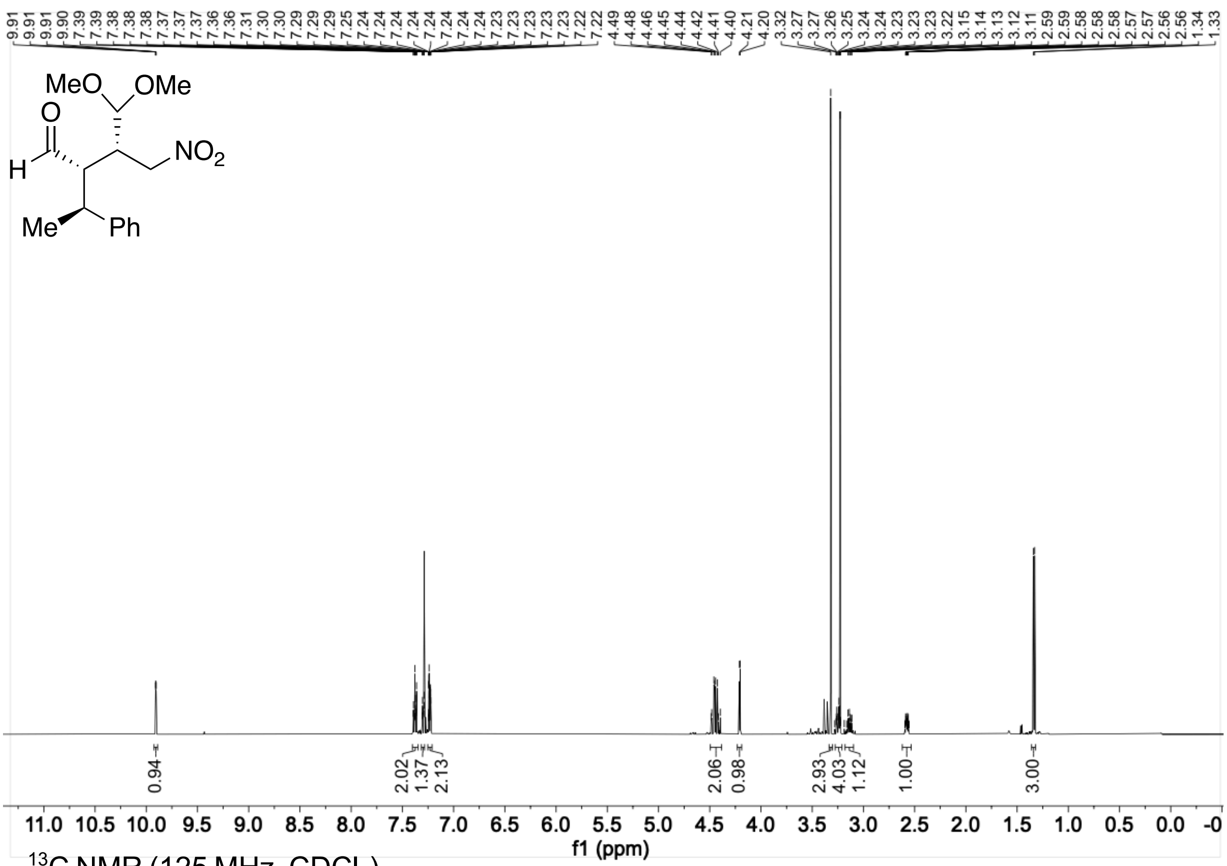

^{13}C NMR (125 MHz, $CDCl_3$)

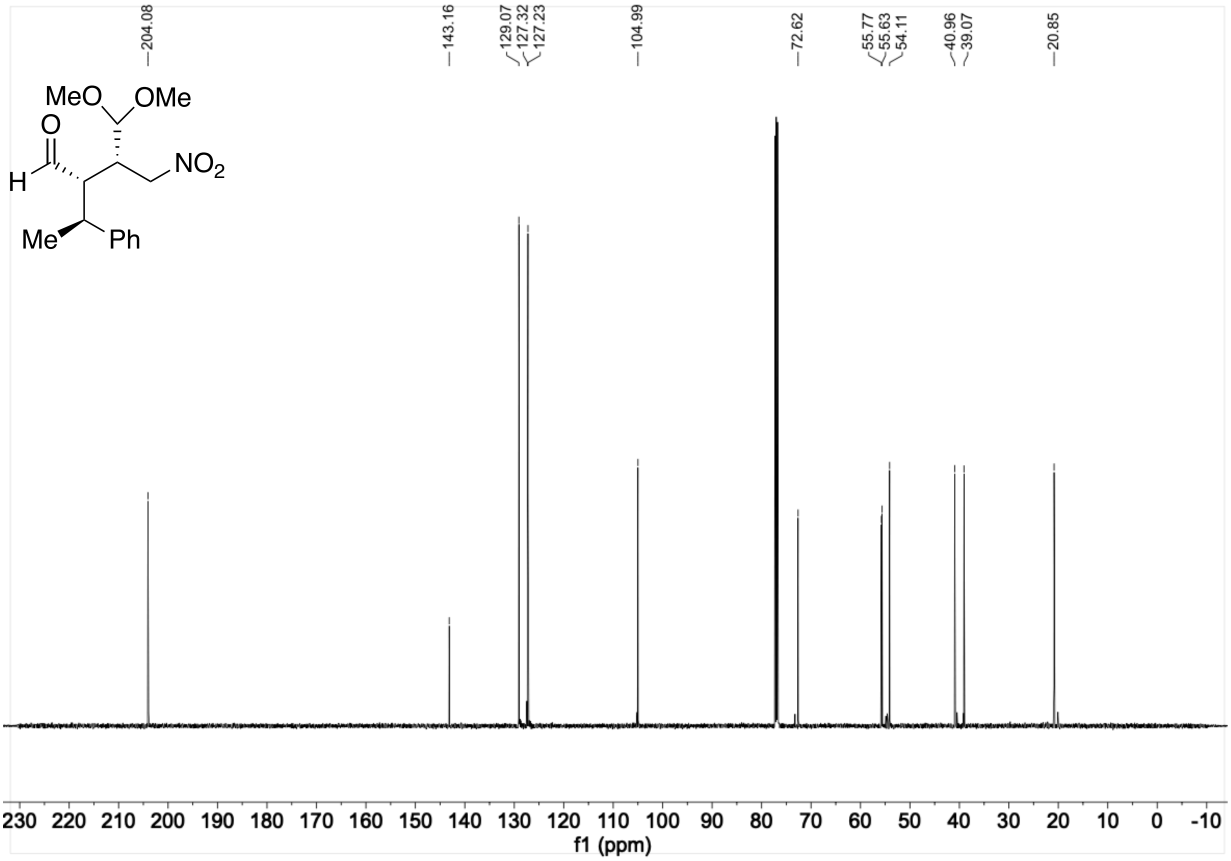

(2*S*,3*S*)-5-Methyl-3-(nitromethyl)-2-((*S*)-1-phenylethyl)hexanal (3ar)

^{1}H NMR (400 MHz, $CDCl_3$)

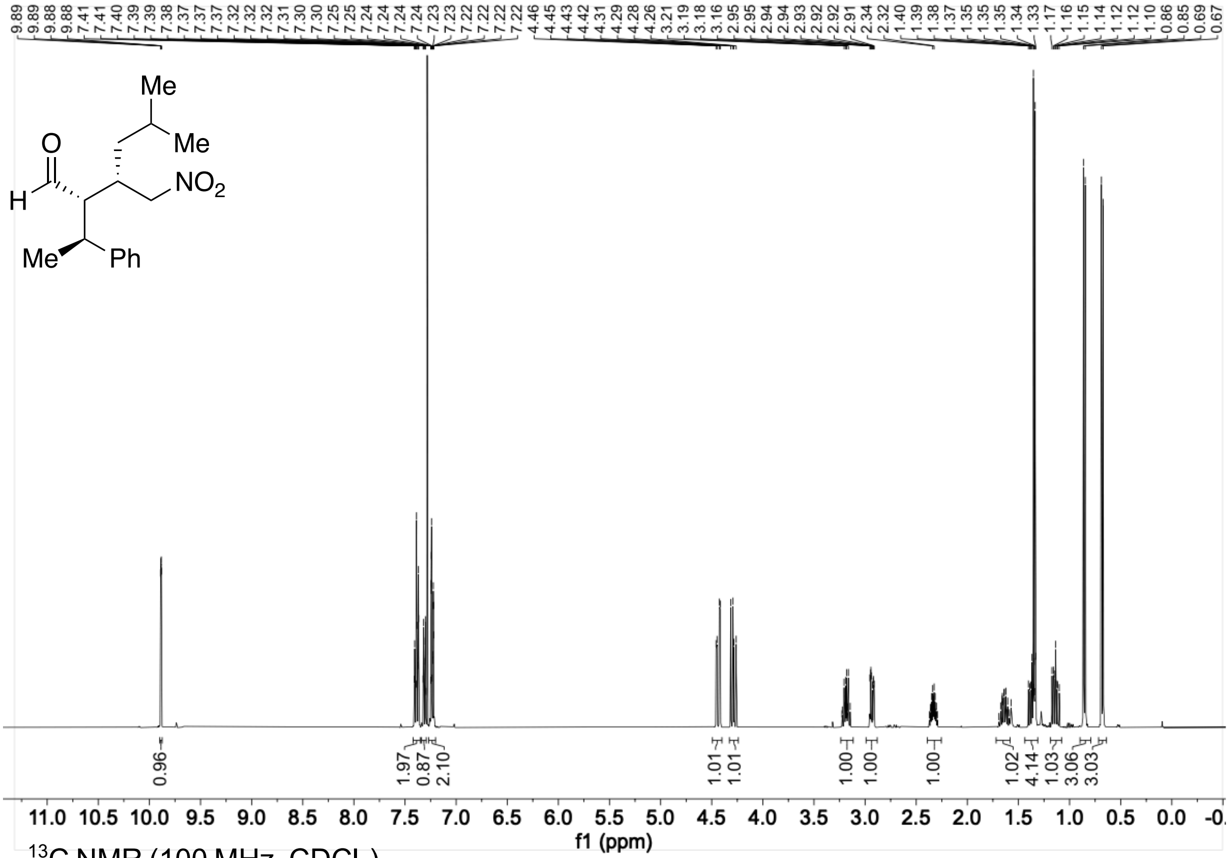

^{13}C NMR (100 MHz, $CDCl_3$)

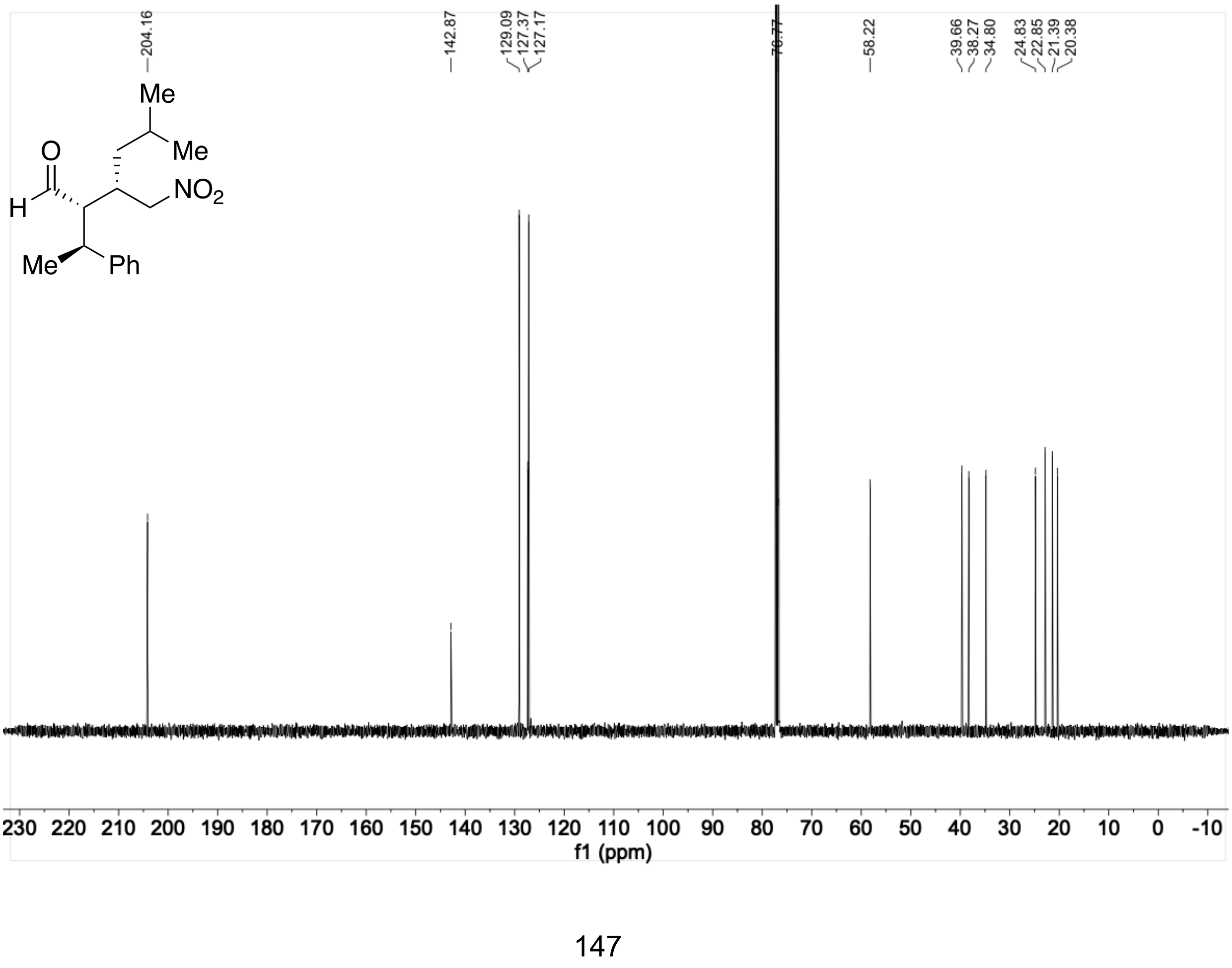

(2*S*,3*S*)-3-Cyclohexyl-4-nitro-2-((*S*)-1-phenylethyl)butanal (3as)

^{1}H NMR (400 MHz, $CDCl_3$)

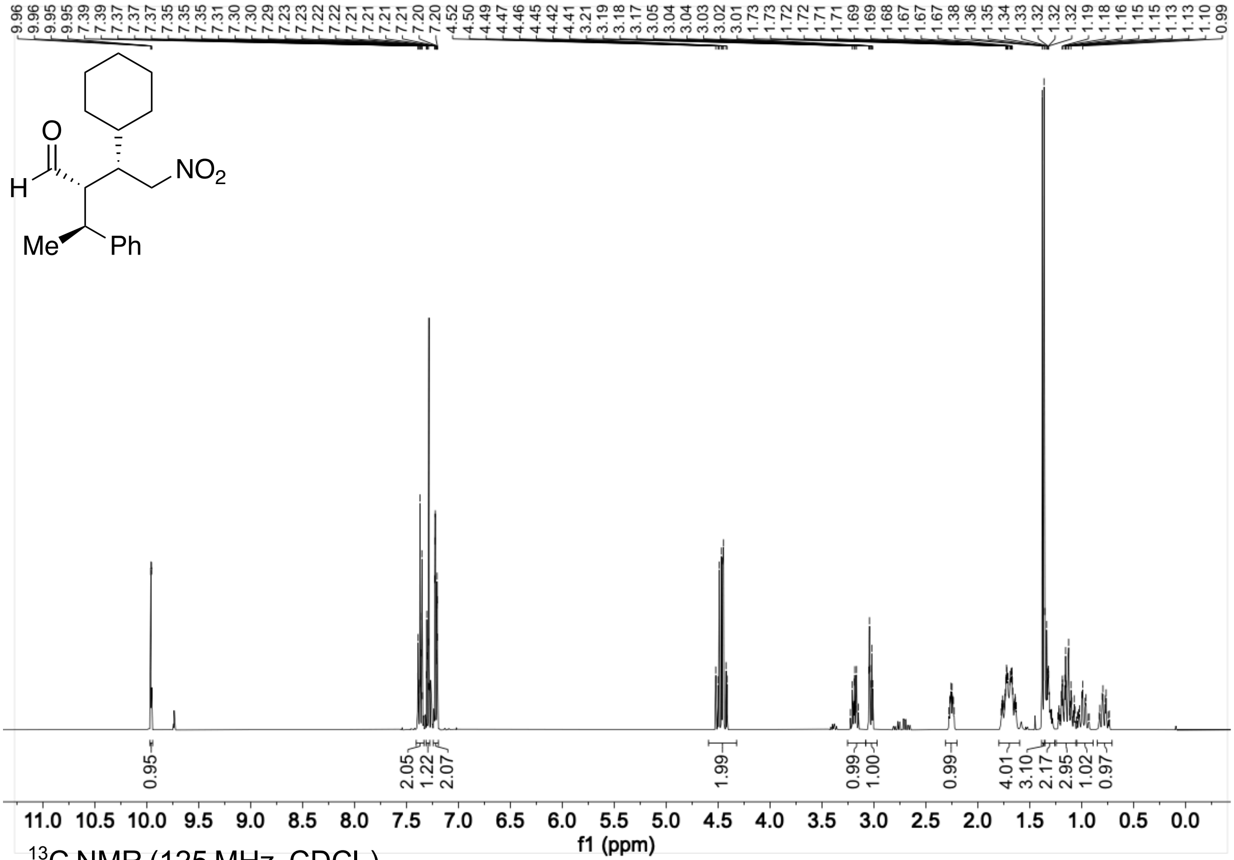

^{13}C NMR (125 MHz, $CDCl_3$)

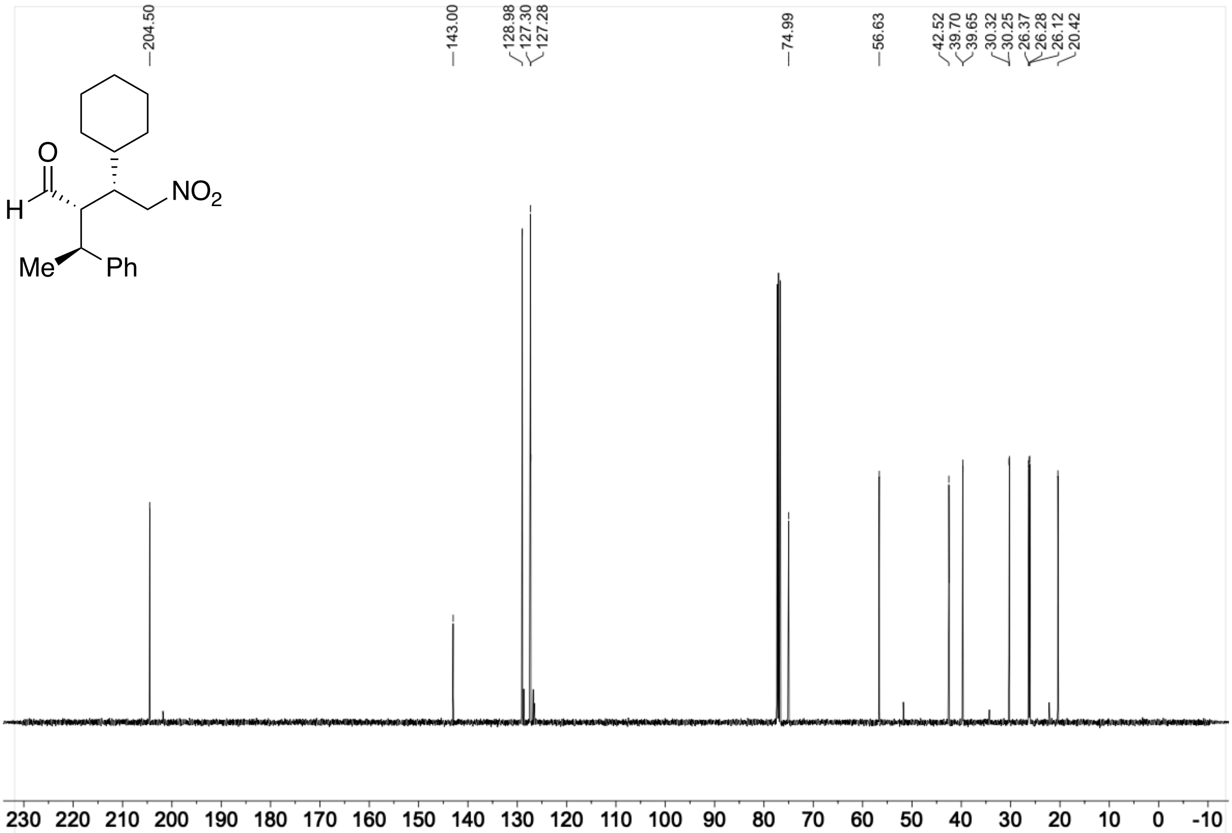

11 NMR Spectroscopic Analysis of Enamine Intermediates

Enantioenriched aldehydes (*R*)-**1a** and (*S*)-**1b** for mechanistic studies were prepared using peptide **B-DLL** or its enantiomer **B-LDD** using the following procedure: A solution of the racemic 3-phenylbutanal (**1a**, 1 equiv., 5 mmol, 0.5 M) in $CHCl_3$/*i*-PrOH 9:1 (10 mL) was cooled to 0 °C. Nitrostyrene **2a** (1.5 equiv., 7.5 mmol), the TFA salt of peptide **B** (3 mol%, 0.15 mmol) and *N*-methylmorpholine (3 mol%, 0.15 mmol) were added and stirred at 0 °C for 24 h. The reaction was allowed to run until ca. 60% conversion of the starting racemic **1a**. The reaction was quenched by the addition of *n*-butanal (2.5 equiv., 12.5 mmol). After full consumption of the remaining nitrostyrene **2a** (note: **2a** has very similar R_f as the aldehyde **1a**, and full consumption of **2a** simplifies purification by column chromatography), the mixture was concentrated *in vacuo* and purified by column chromatography (gradient elution from hexane to 10% EtOAc in hexane). The enantiomeric excess was determined by chiral stationary phase SFC. (*R*)-**1a** (99% ee) was obtained using catalyst **B-DLL** as a colourless liquid in 34% yield. (*S*)-**1a** (99% ee) was obtained using catalyst **B-LDD** as a colourless liquid in 21% yield.

Peptide **B** (1 equiv., 20 µmol), NMM (1 equiv., 20 µmol) and the aldehyde (1.5 equiv., 30 µmol of either *rac*-**1a**, (*S*)-**1a**, or (*R*)-**1a**) were stirred over pre-activated molecular sieve (4 Å) in dry DMSO-d_6 (750 µL) for 30 min. NMR spectra were recorded after filtration.

Enamine (*R*)-En-B

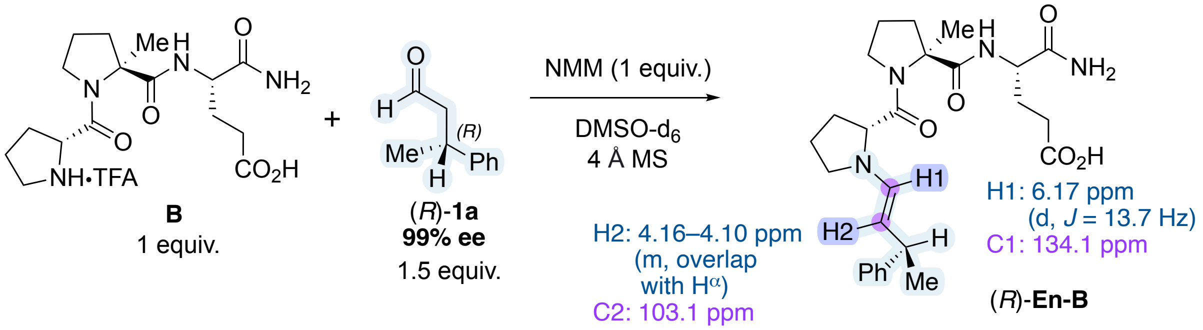

Scheme S2: NMR spectroscopic analysis of the enamine formed by reaction of catalyst **B** with (*R*)-3-phenylbutanal (*R*)-**1a**.

Only the *trans*-conformer of the 3° amide and the (*E*)-isomer of the enamine double bond is visible in the NMR spectra.

^{1}H NMR (500 MHz, DMSO-d6)

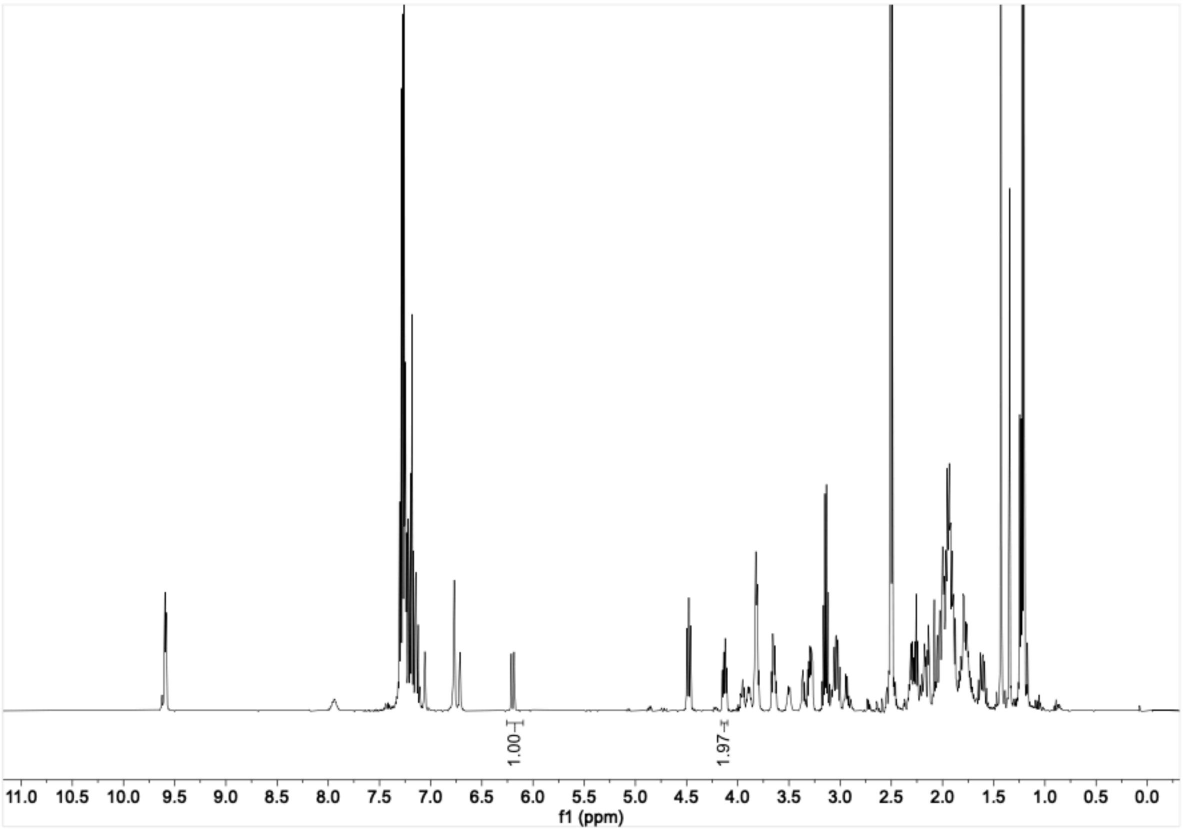

^{13}C NMR (127 MHz, DMSO-d6)

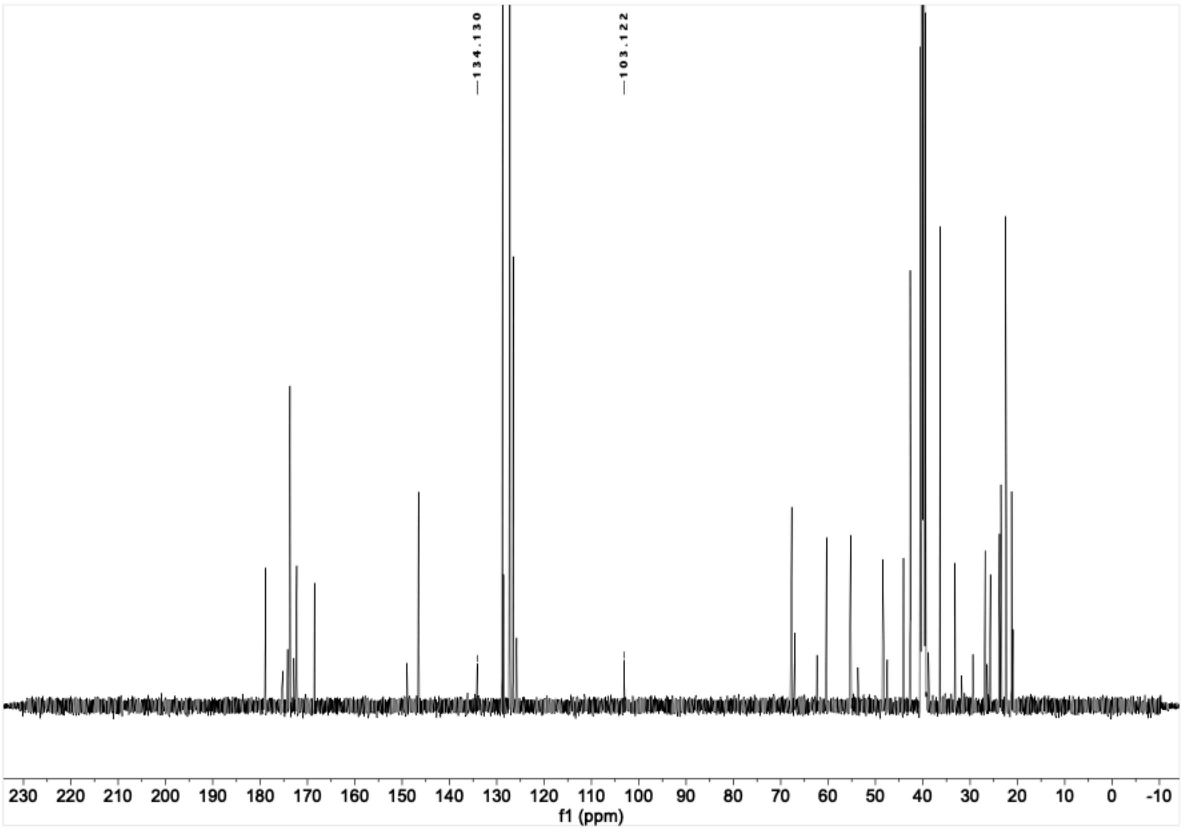

COSY (500 MHz, DMSO-d$_6$)

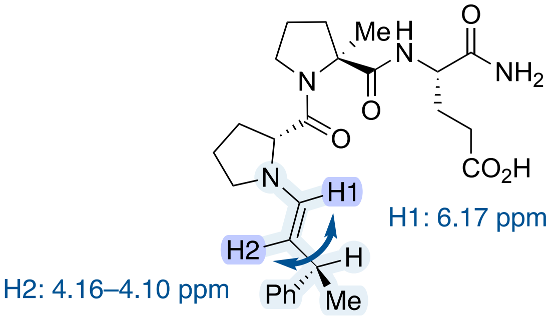

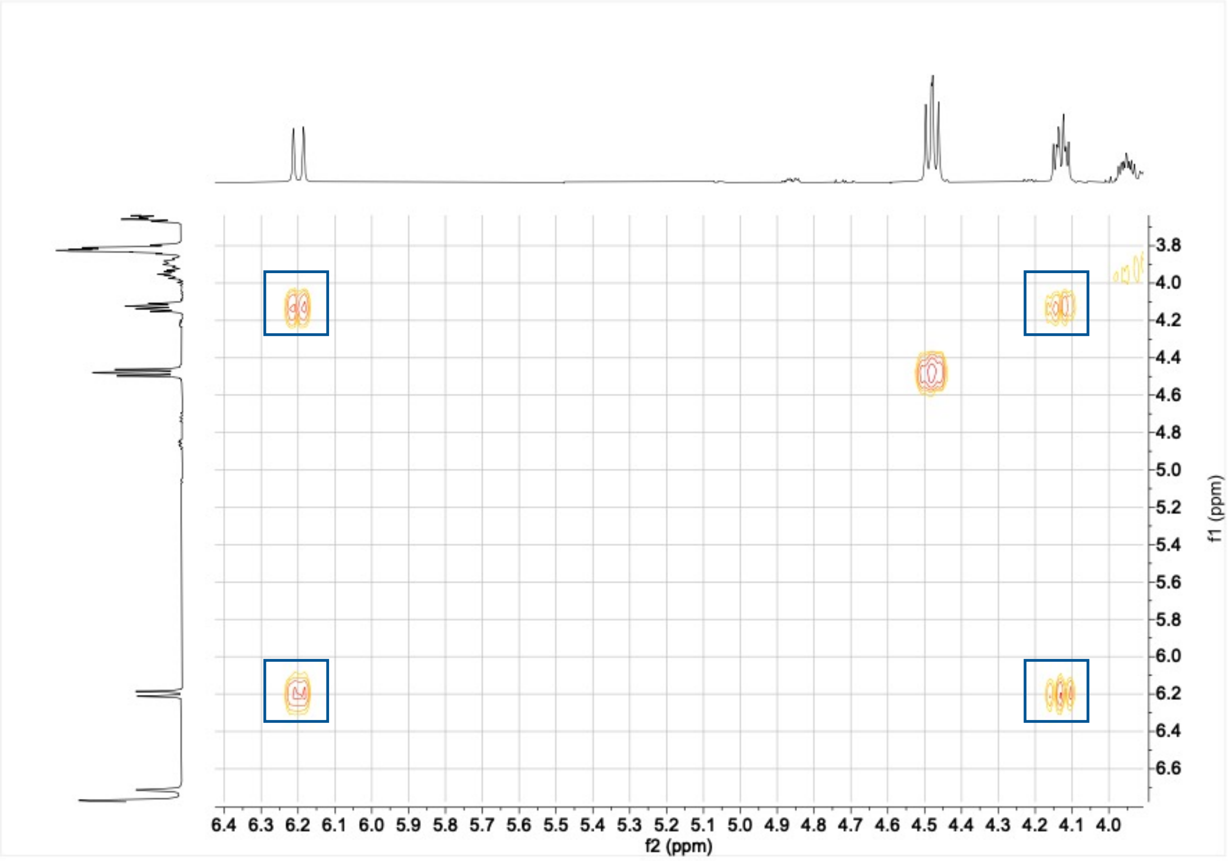

HSQC (500 MHz, DMSO-d6)

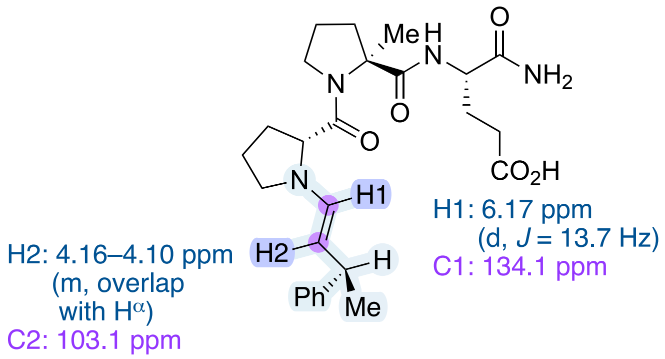

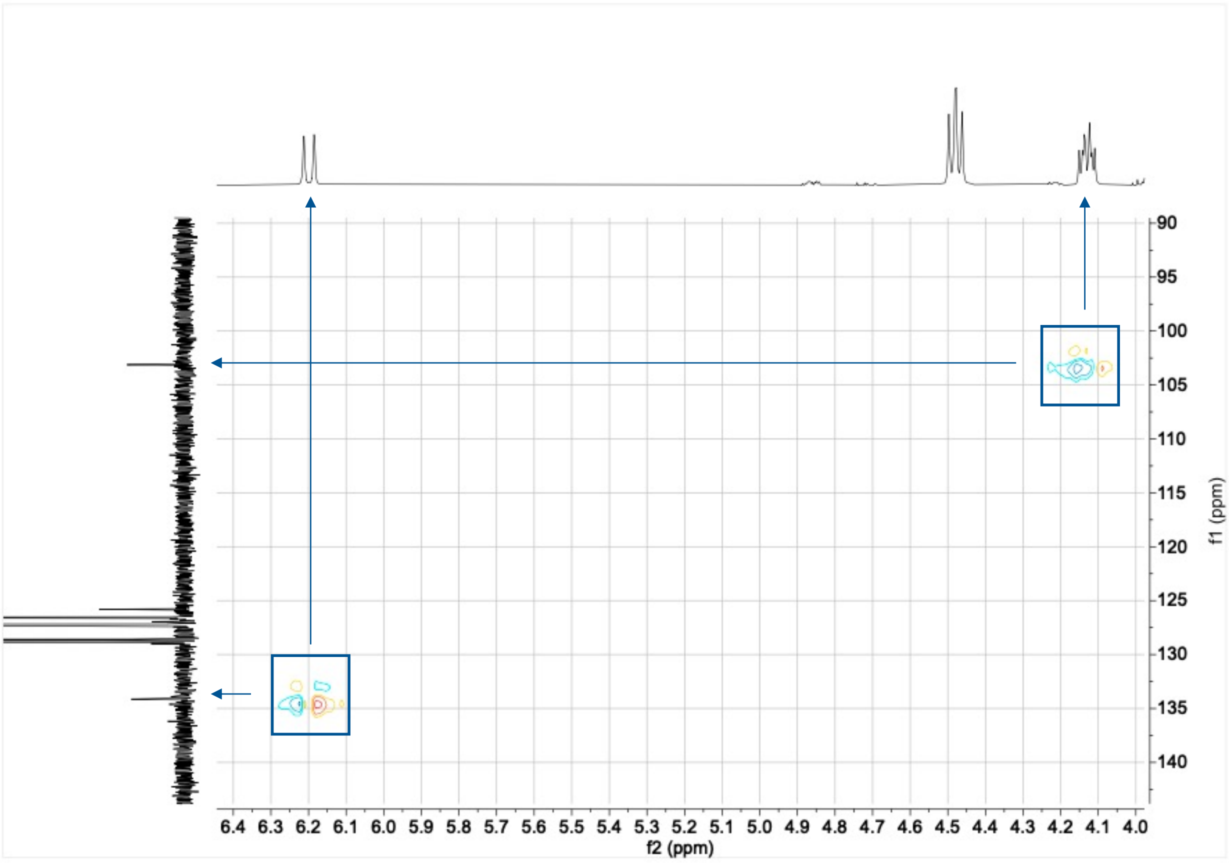

Enamine (*S*)-En-B

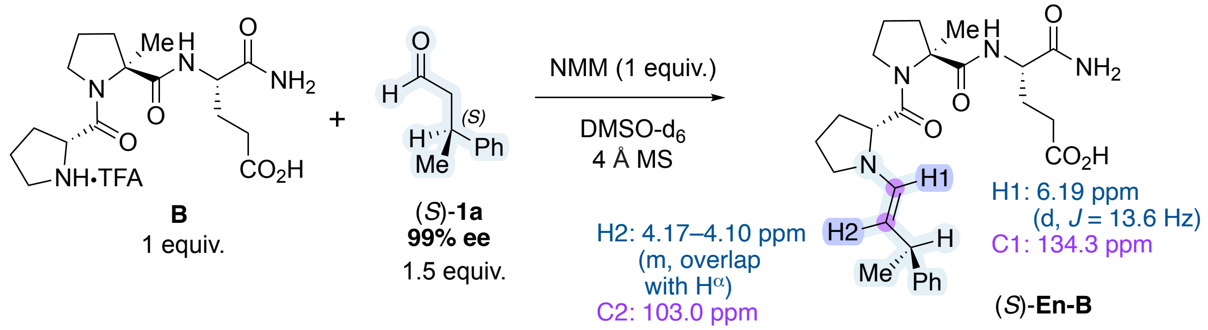

Scheme S3: NMR spectroscopic analysis of the enamine formed by reaction of catalyst **B** with (*S*)-3-phenylbutanal (*S*)-**1a**.

Only the *trans*-conformer of the 3° amide and the (*E*)-isomer of the enamine double bond is visible in the NMR spectra.

^{1}H NMR (500 MHz, DMSO-d$_6$)

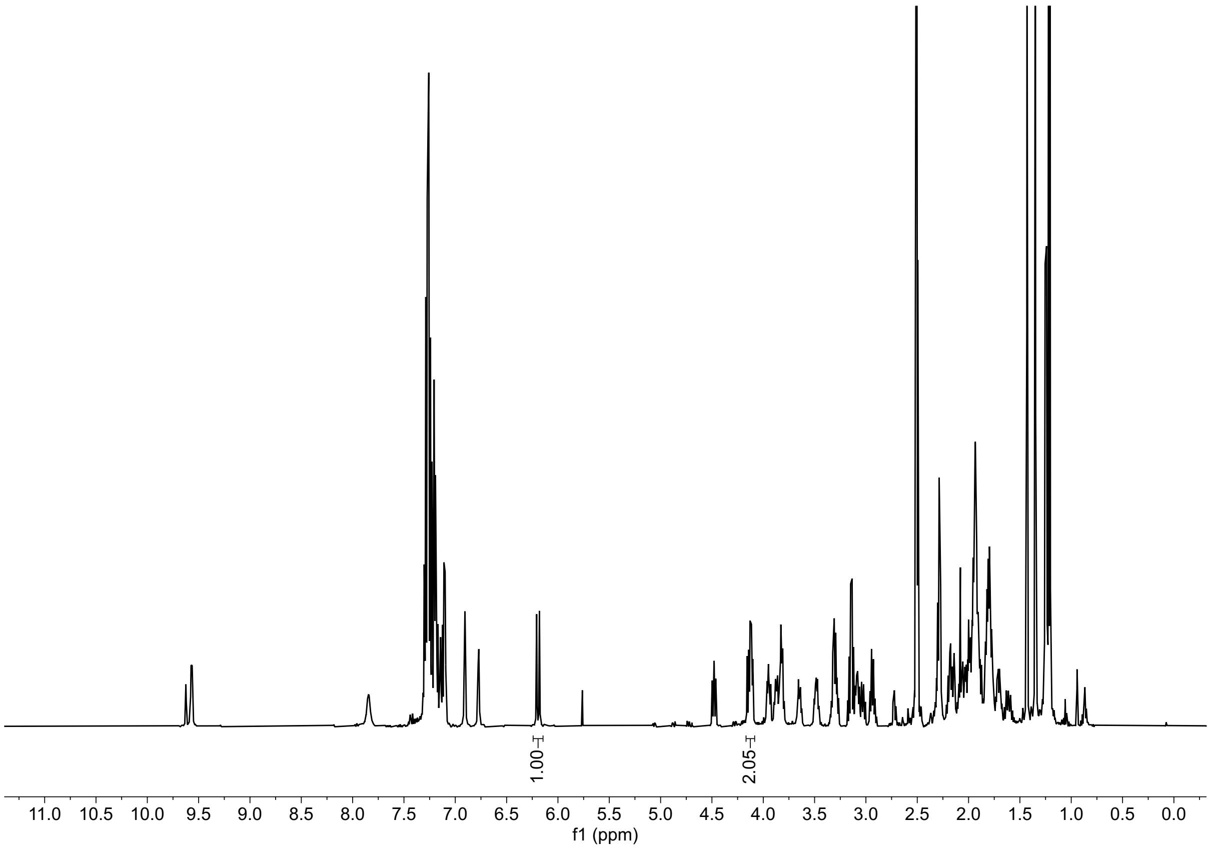

^{13}C NMR (127 MHz, DMSO-d$_6$)

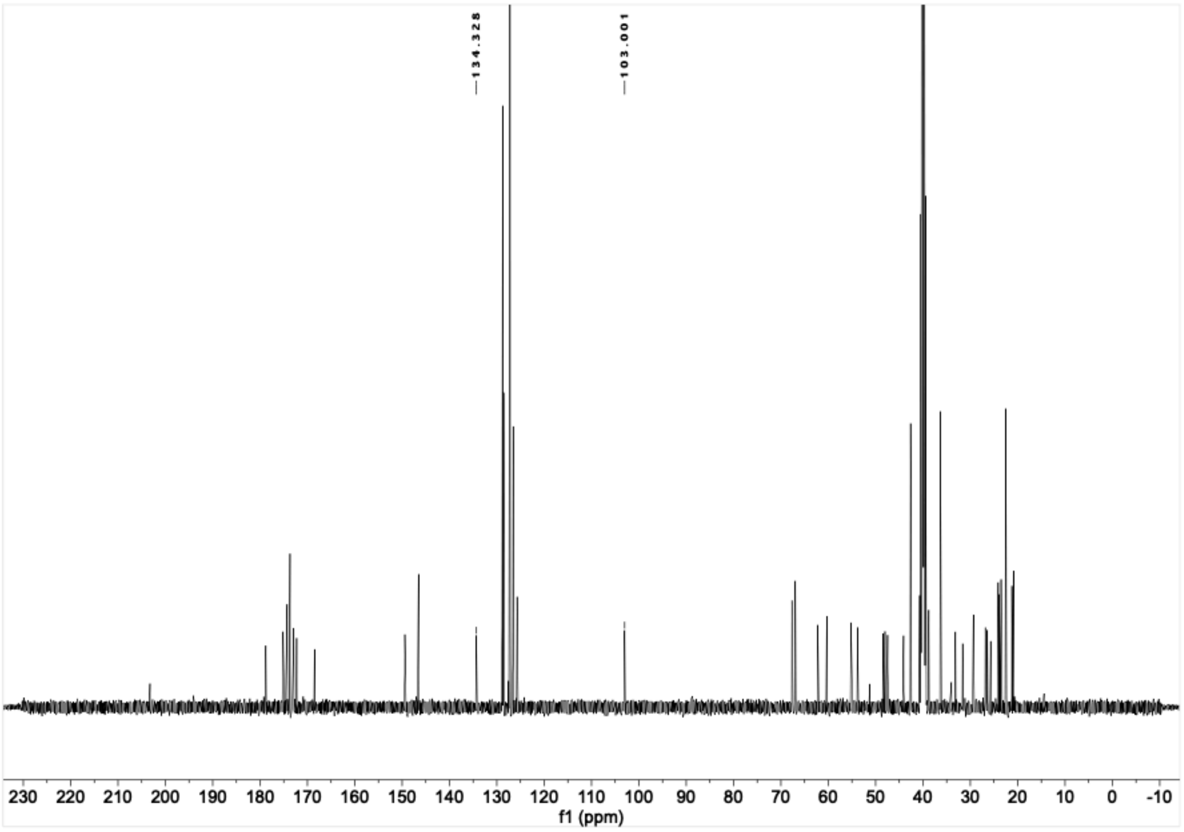

COSY (500 MHz, DMSO-d$_6$)

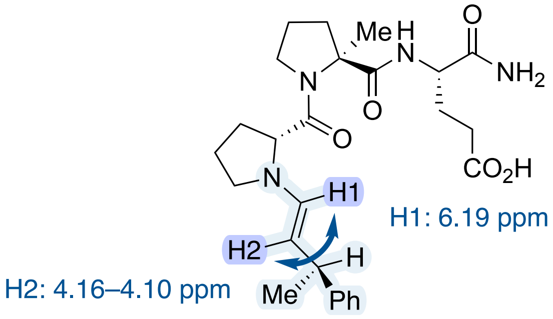

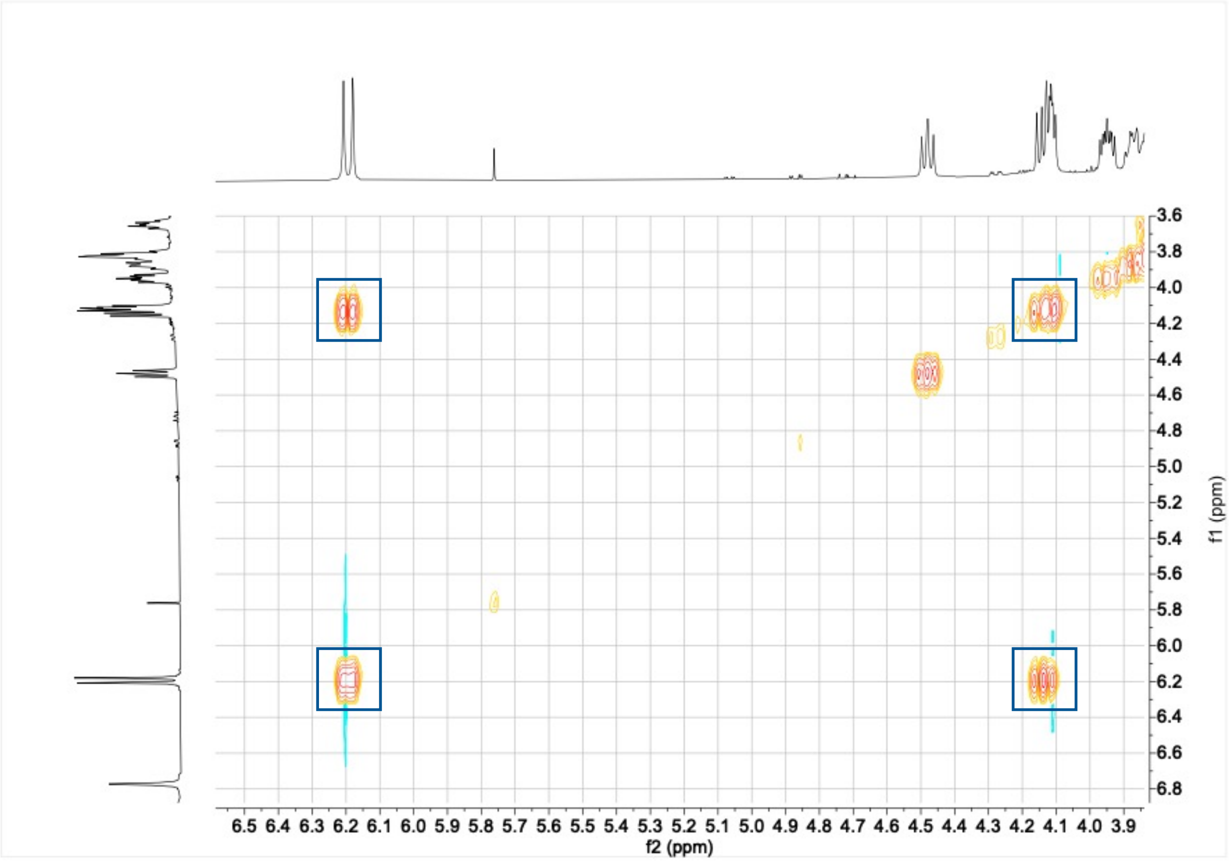

HSQC (500 MHz, DMSO-d$_6$)

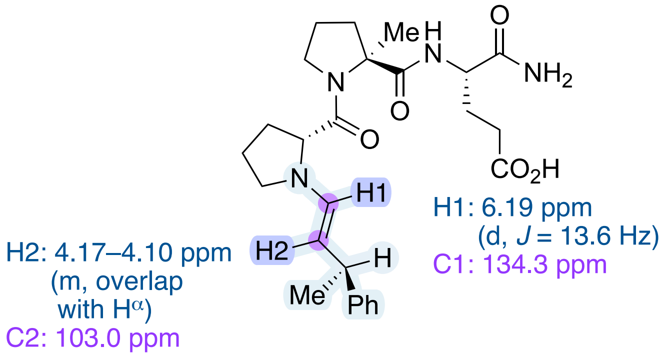

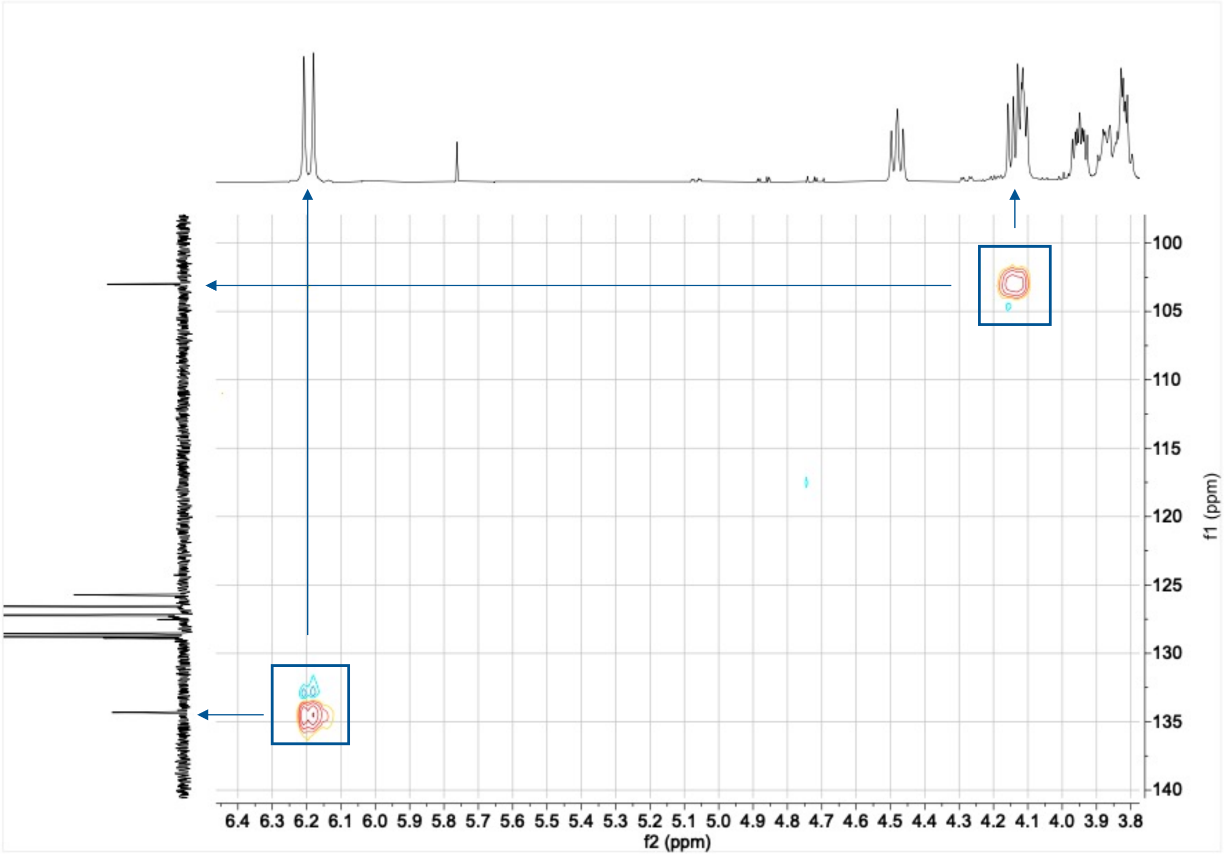

Enamines formed by reaction of catalyst B with *rac*-1a

Since the diastereomeric olefinic proton signals overlap in the ^{1}H NMR spectrum, the diastereomeric ratio was determined using inverse-gated ^{13}C NMR for quantitative measurements by manual integration of the signals corresponding to the olefinic enamine carbons of the two diastereoisomers.

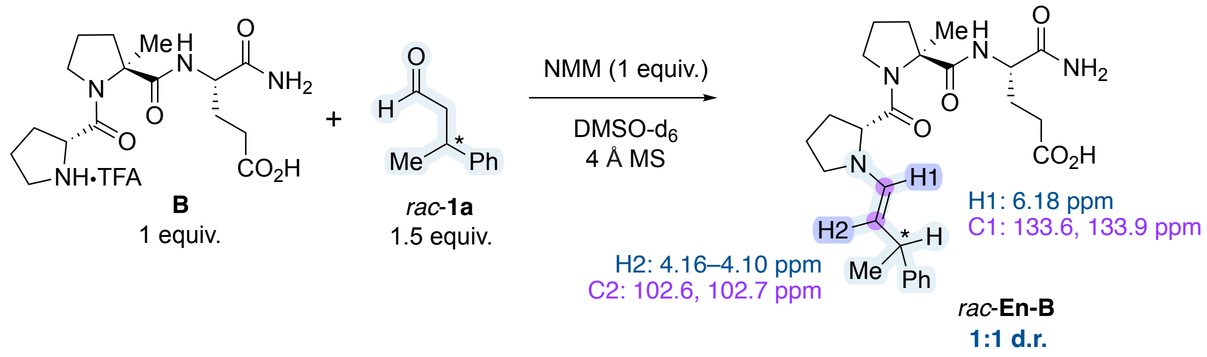

Scheme S4: NMR spectroscopic analysis of enamine formed from a reaction of catalyst **B** and racemic 3-phenylbutanal *rac*-**1a**.

Only the *trans*-conformer of the 3° amide and the (*E*)-isomer of the enamine double bond is visible in the NMR spectra.

^{1}H NMR (600 MHz, DMSO-d$_6$)

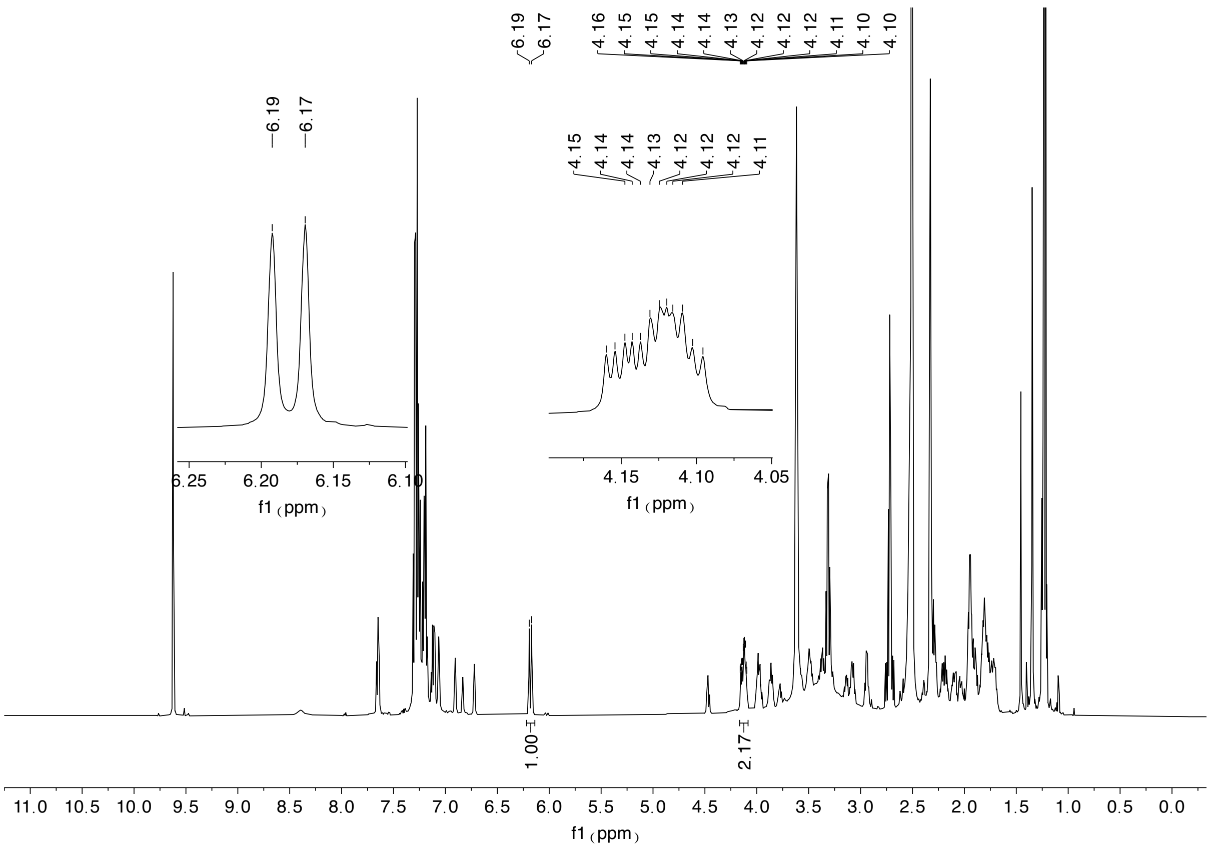

^{13}C NMR (151 MHz, DMSO-d$_6$)

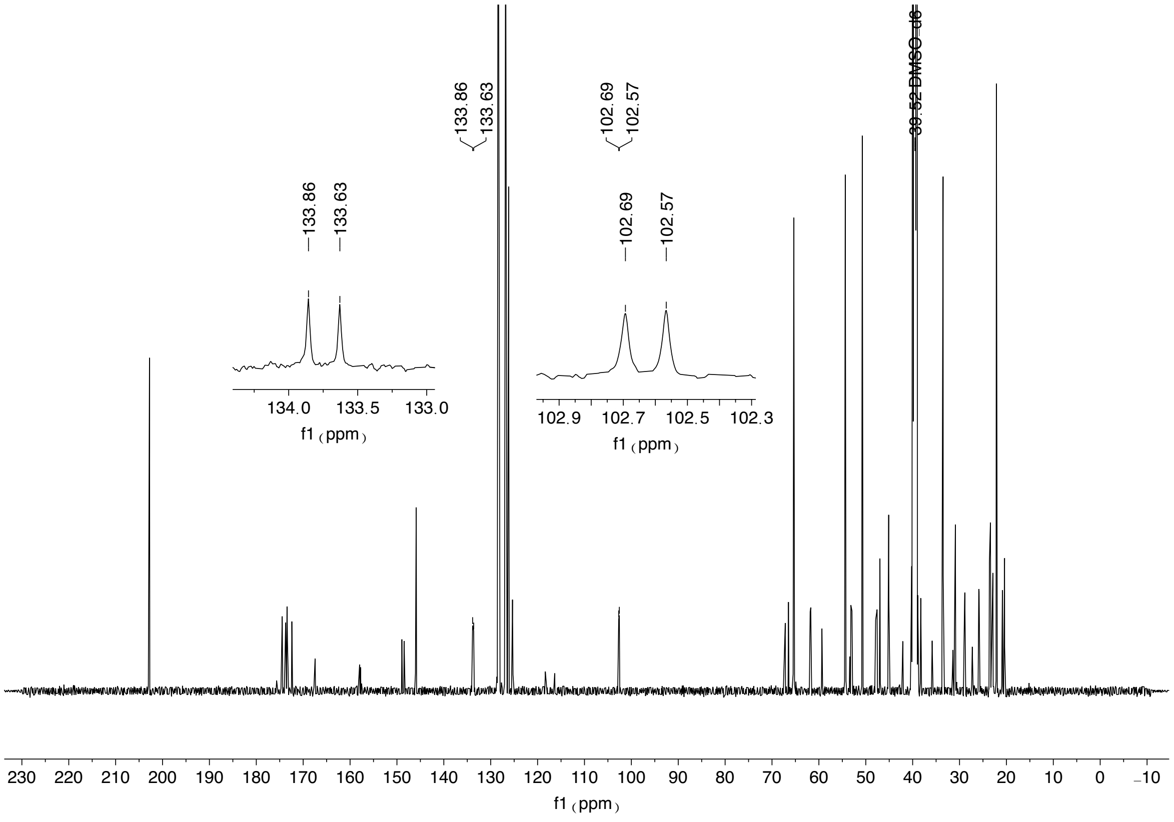

COSY (600 MHz, DMSO-d6)

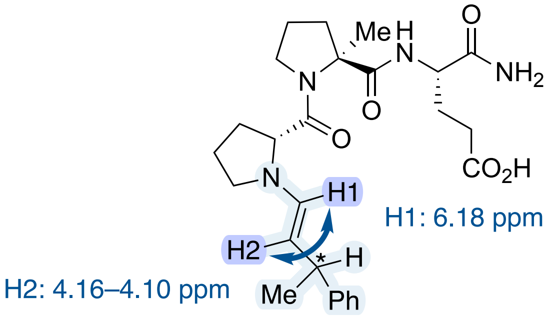

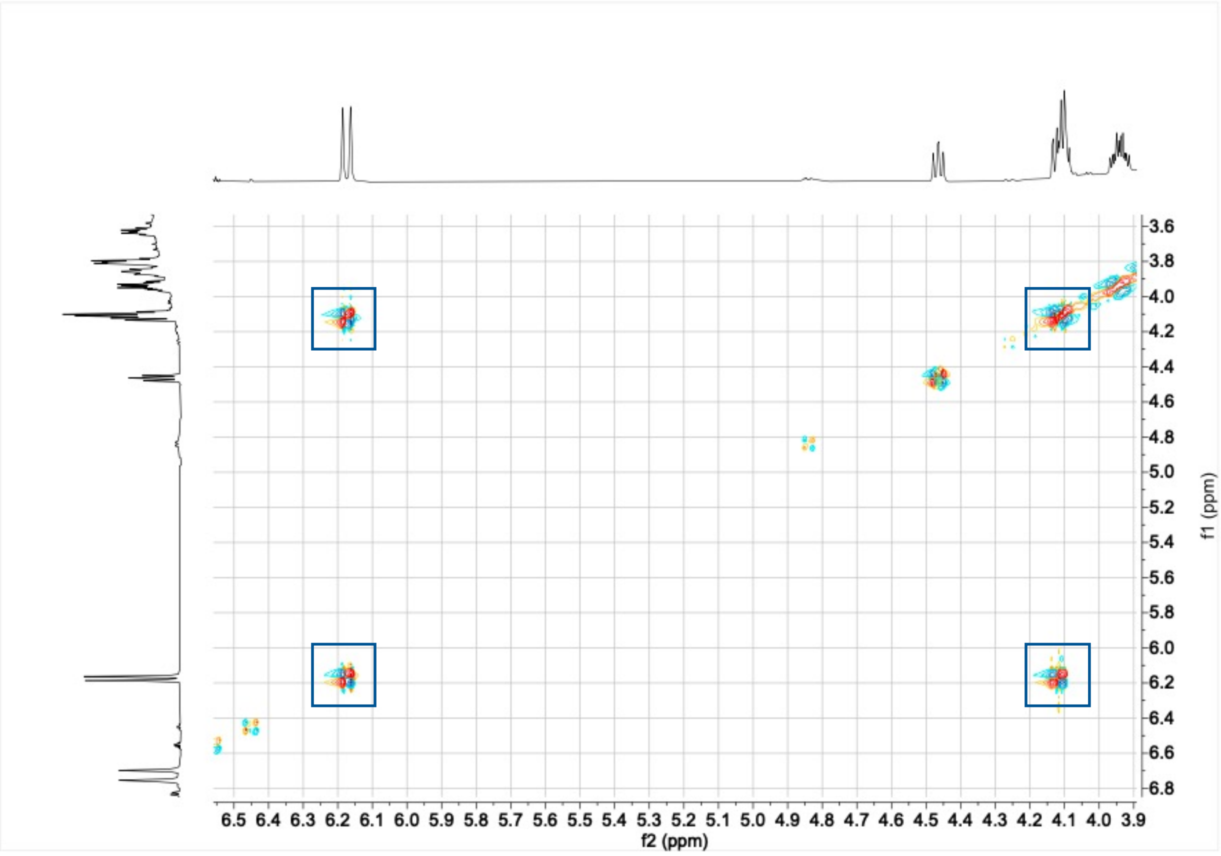

HSQC (600 MHz, DMSO-d_6)

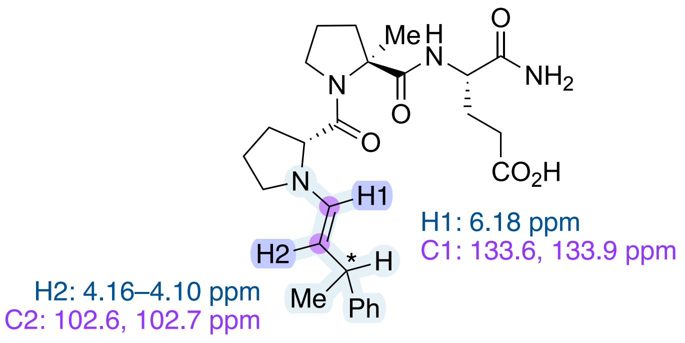

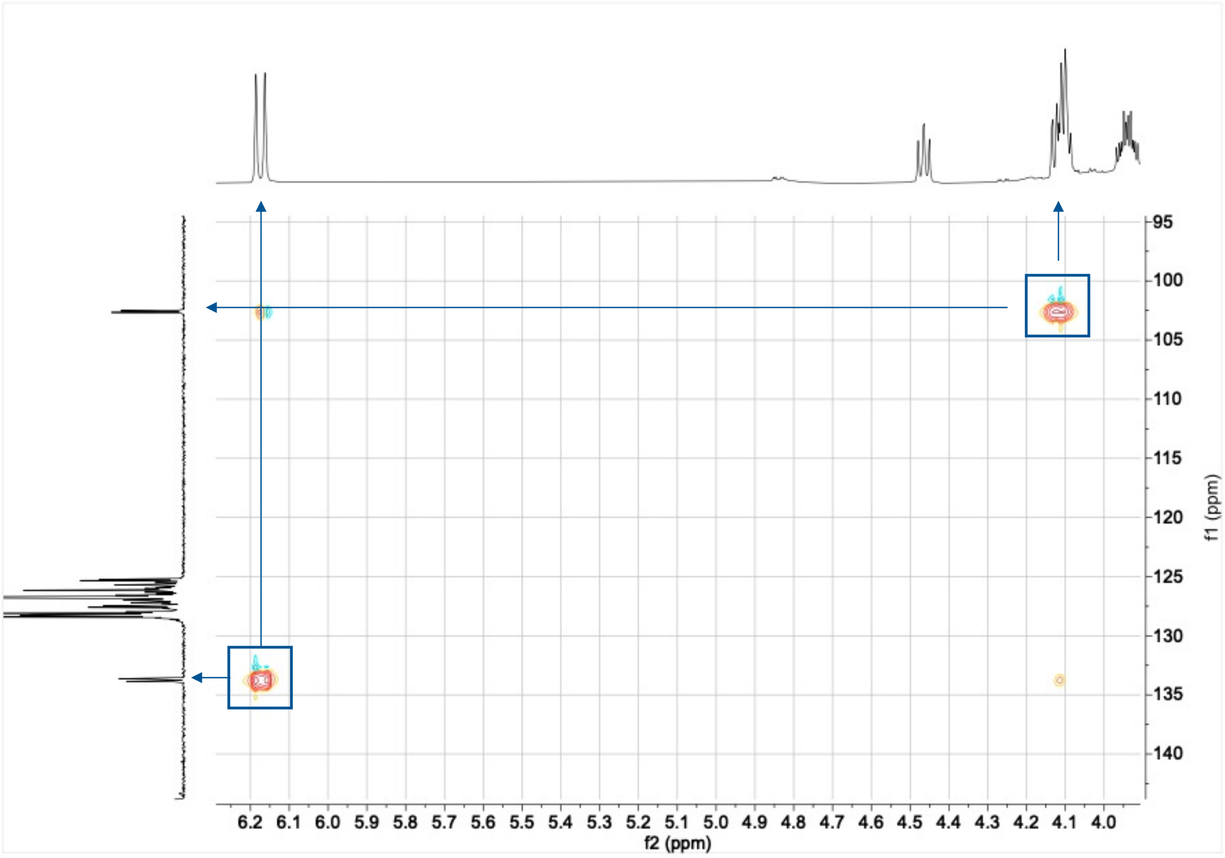

HMBC (600 MHz, DMSO-d6)

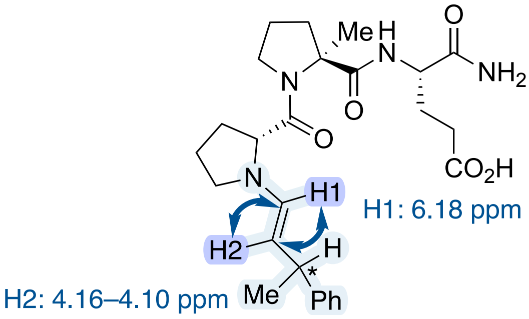

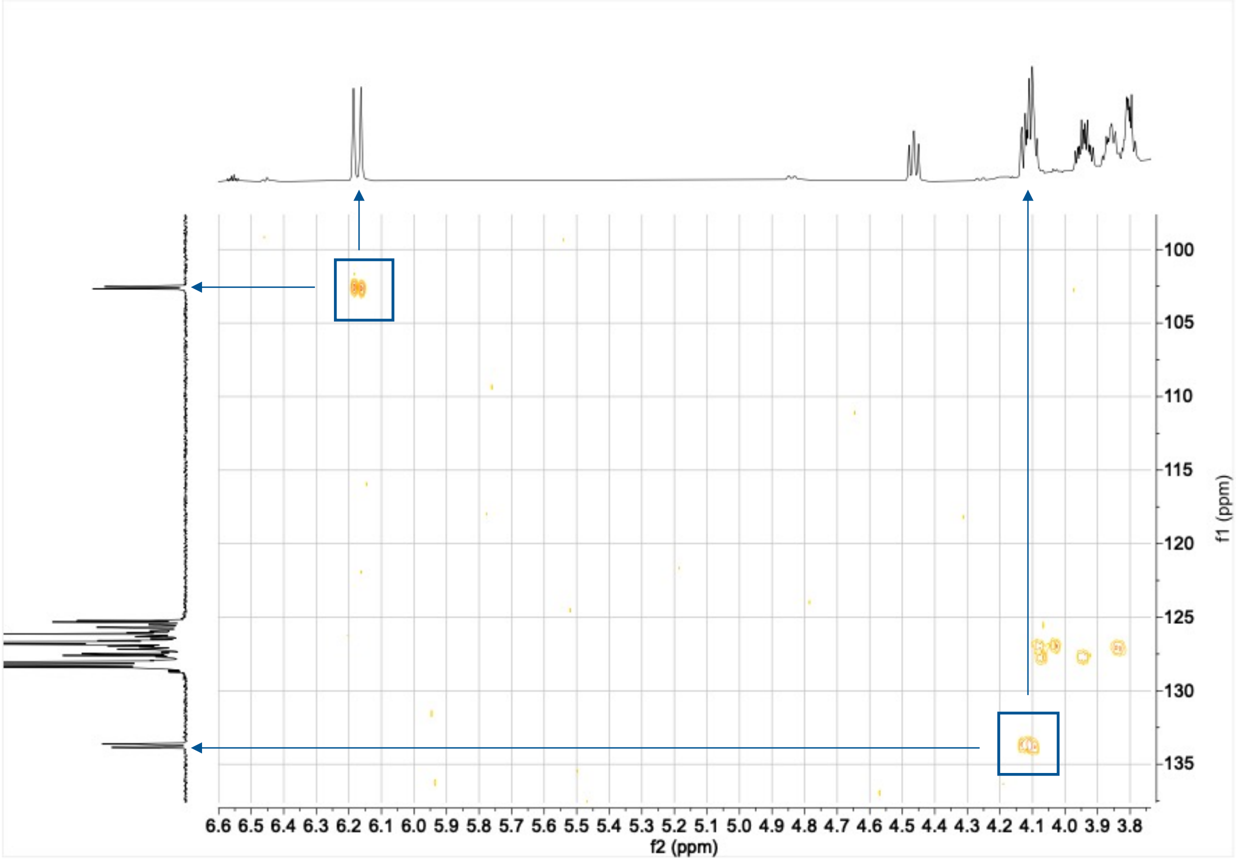

^{13}C NMR (151 MHz, DMSO-d$_6$) *inverse gated for quantitative measurement*

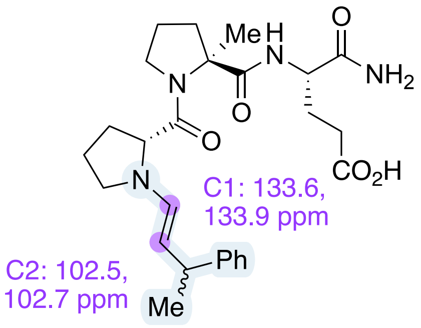

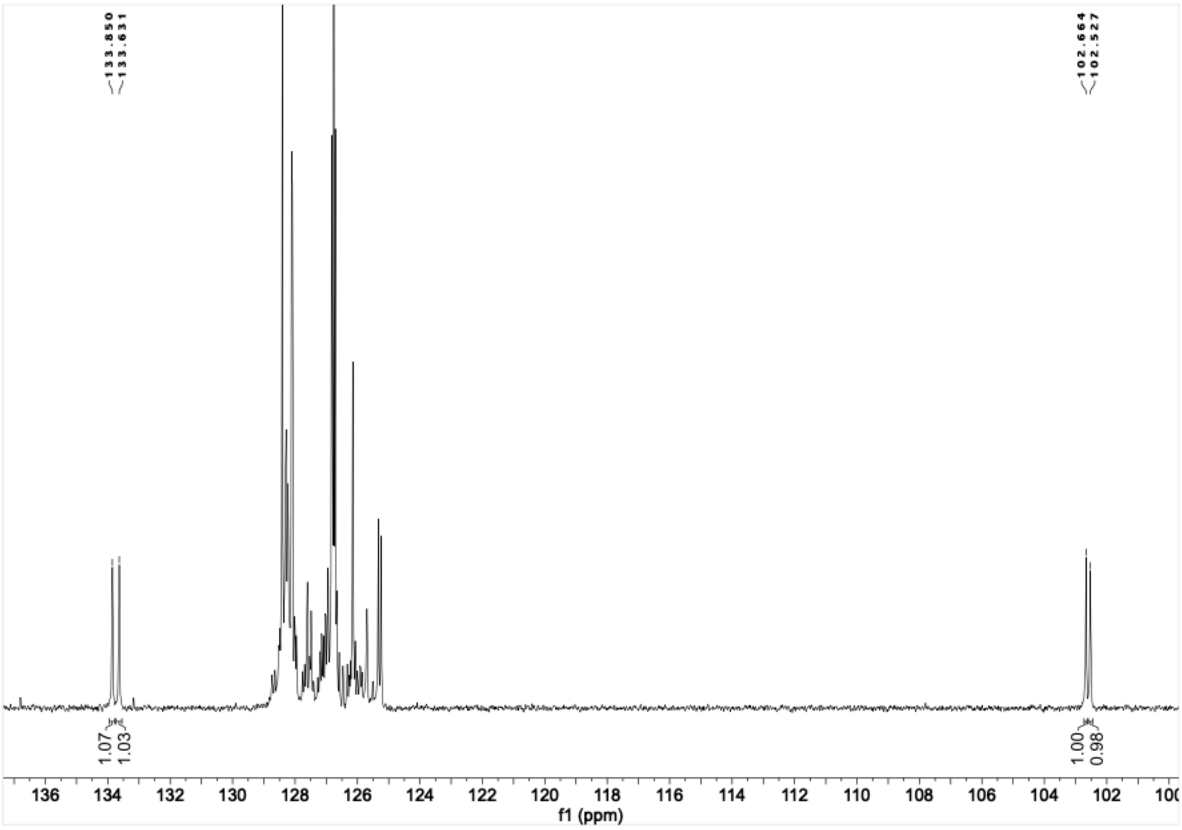

12 Kinetic Experiments

General Procedure for Kinetic Experiments

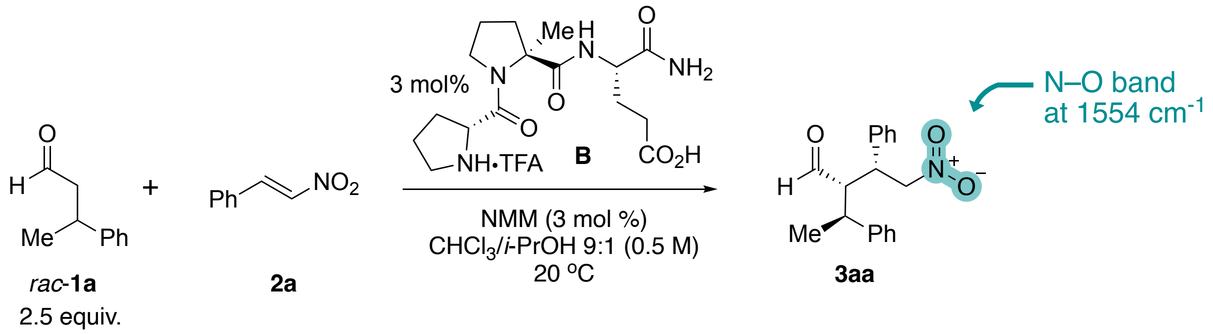

Scheme S5: Reaction set up used to perform kinetic experiments.

An oven dried 10 mL flask was charged with peptide **B** (7.5 μmol, 3 mol%) and nitroolefin **2a** (0.25 mmol, 1 equiv.). $CHCl_3$/*i*-PrOH 9:1 (0.5 M) and *N*-methylmorpholine (7.5 μmol, 3 mol%) were added followed by the addition of aldehyde *rac*-**1a** (0.625 mmol, 2.5 equiv.) and prompt transfer onto the *in-situ* FT-IR reactor. Reaction progress was monitored by FTIR at the N–O stretching-absorbance of the forming product γ-nitroaldehyde at approximately 1554 cm^{-1} (Figure S2).[8] The final conversion of **2a** to **3aa** was determined by ^{1}H NMR spectroscopy.

The same procedure was followed using different equivalents of *rac*-**1a** (2 equiv. or 1 equiv.) and enantioenriched aldehyde (*S*)-**1a** (99% ee, 1.25 equiv.) or (*R*)-**1a** (99% ee, 1.25 equiv.).

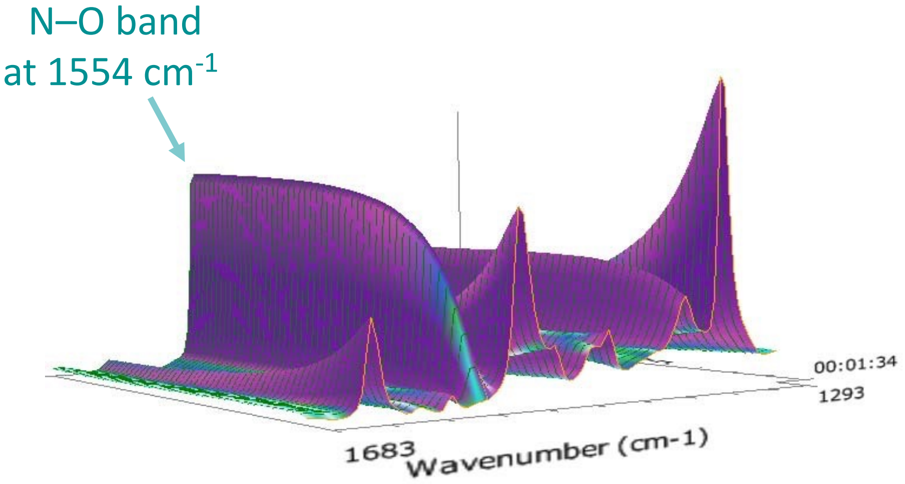

Figure S2: Sample FT-IR spectrum of peptide catalyzed conjugate addition reaction between aldehyde *rac*-**1a** and nitroolefin **2a**.

13 X-Ray Crystal Structures

Crystal structure of 4-nitro-3-phenyl-2-(1-phenylethyl)butanal (3aa)

Single crystals of **3aa** were obtained by slow evaporation from a mixture of *i*-PrOH/*n*-hexane. A suitable crystal was selected and measured on a XtaLAB Synergy, Dualflex, Pilatus 300K diffractometer. The crystal was kept at 100.0(1) K during data collection. Using Olex2,[11] the structure was solved with the ShelXT[12] structure solution program using Intrinsic Phasing and refined with the ShelXL[13] refinement package using Least Squares minimisation.

Crystal Data for $C_{18}H_{19}NO_3$ (M = 297.34 g/mol): orthorhombic, space group $P2_12_12_1$ (no. 19), a = 8.41667(4) Å, b = 10.67333(5) Å, c = 17.27872(11) Å, V = 1552.214(15) Å^3, Z = 4, T = 100.0(1) K, μ(CuKα) = 0.700 mm^{-1}, $Dcalc$ = 1.272 g/cm^3, 47677 reflections measured (9.74° ≤ 2Θ ≤ 159.508°), 3362 unique (R_{int} = 0.0324, R_{sigma} = 0.0117) which were used in all calculations. The final R_1 was 0.0295 (I > 2σ(I)) and wR_2 was 0.0745 (all data).

The crystal structure is deposited in the Cambridge Crystallographic Data Center. The deposition number is CCDC 2152244.

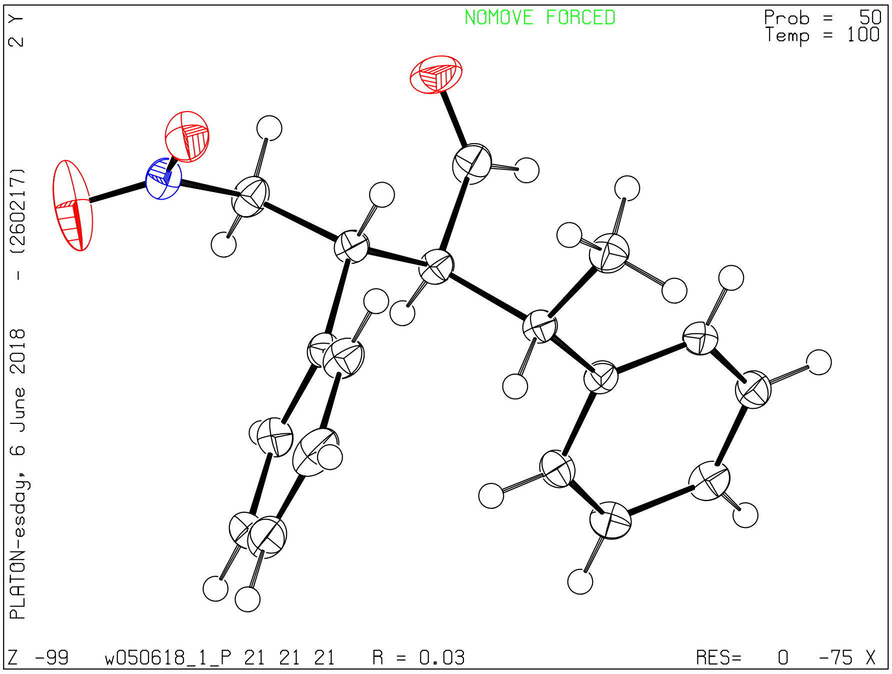

Figure S3: Crystal structure of **3aa** (CCDC 2152244).

Crystal structure of 3-(4-fluorophenyl)-4-nitro-2-(1-phenylethyl)butanal (3ab)

Single crystals of **3ab** were obtained by slow evaporation from a mixture of *i*-PrOH/*n*-hexane. A suitable crystal was selected and measured on a XtaLAB Synergy, Dualflex, Pilatus 300K diffractometer. The crystal was kept at 100.0(1) K during data collection. Using Olex2,[11] the structure was solved with the ShelXT[12] structure solution program using Intrinsic Phasing and refined with the ShelXL[13] refinement package using Least Squares minimisation.

Crystal Data for $C_{18}H_{18}FNO_3$ (M =315.33 g/mol): orthorhombic, space group $P2_12_12_1$ (no. 19), a = 8.45630(10) Å, b = 10.77780(10) Å, c = 17.10180(10) Å, V = 1558.66(3) Å^3, Z = 4, T = 100.0(1) K, μ(CuKα) = 0.825 mm^{-1}, $Dcalc$ = 1.344 g/cm^3, 38198 reflections measured (9.7° ≤ 2Θ ≤ 159.35°), 3374 unique (R_{int} = 0.0392, R_{sigma} = 0.0153) which were used in all calculations. The final R_1 was 0.0268 (I > 2σ(I)) and wR_2 was 0.0673 (all data).

The crystal structure is deposited in the Cambridge Crystallographic Data Center. The deposition number is CCDC 2152245.

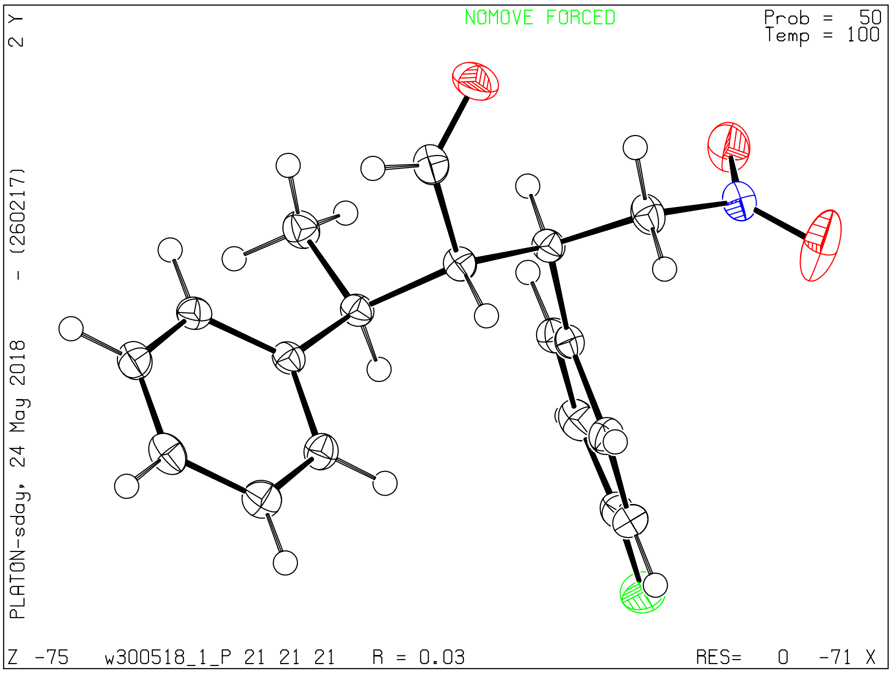

Figure S4: Crystal structure of **3ab** (CCDC 2152245).

Crystal structure of 3-(4-chlorophenyl)-4-nitro-2-(1-phenylethyl)butanal (3ac)

Single crystals of **3ac** were obtained by slow evaporation from a mixture of *i*-PrOH/*n*-hexane. A suitable crystal was selected and measured on a XtaLAB Synergy, Dualflex, Pilatus 300K diffractometer. The crystal was kept at 100.0(1) K during data collection. Using Olex2,[11] the structure was solved with the ShelXT[12] structure solution program using Intrinsic Phasing and refined with the ShelXL[13] refinement package using Least Squares minimisation.

Crystal Data for $C_{18}H_{18}ClNO_3$ (M 331.78 g/mol): orthorhombic, space group $P2_12_12_1$ (no. 19), a = 8.67420(10) Å, b = 10.86610(10) Å, c = 17.03310(10) Å, V = 1605.45(3) Å^3, Z = 4, T = 100.0(1) K, μ(Cu Kα) = 2.231 mm^{-1}, $Dcalc$ = 1.373 g/cm^3, 49424 reflections measured (9.654° ≤ 2Θ ≤ 158.884°), 3439 unique (R_{int} = 0.0310, R_{sigma} = 0.0115) which were used in all calculations. The final R_1 was 0.0234 (I > 2σ(I)) and wR_2 was 0.0595 (all data).

The crystal structure is deposited in the Cambridge Crystallographic Data Center. The deposition number is CCDC 2152234.

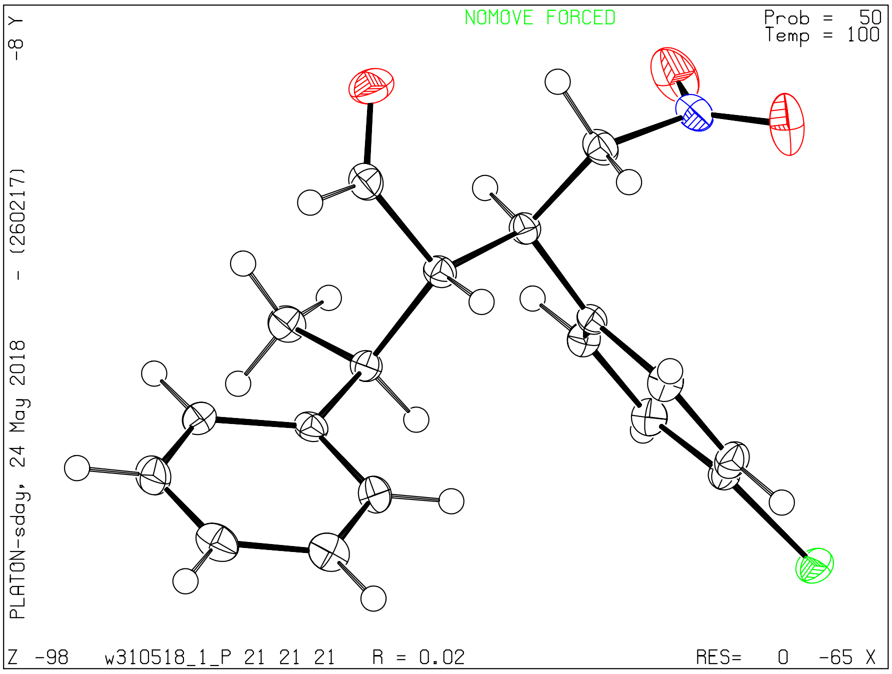

Figure S5: Crystal structure of **3ac** (CCDC 2152234).

Crystal structure of 3-(3-chlorophenyl)-4-nitro-2-(1-phenylethyl)butanal (3ad)

Single crystals of **3ad** were obtained by slow evaporation from a mixture of *i*-PrOH/*n*-hexane. A suitable crystal was selected and measured on a XtaLAB Synergy, Dualflex, Pilatus 300K diffractometer. The crystal was kept at 100.0(1) K during data collection. Using Olex2,[11] the structure was solved with the ShelXT[12] structure solution program using Intrinsic Phasing and refined with the ShelXL[13] refinement package using Least Squares minimisation.

Crystal Data for $C_{18}H_{18}ClNO_3$ (M =331.78 g/mol): orthorhombic, space group $P2_12_12_1$ (no. 19), a = 8.55070(10) Å, b = 11.10360(10) Å, c = 17.0193(2) Å, V = 1615.87(3) Å^3, Z = 4, T = 100.0(1) K, μ(CuKα) = 2.217 mm^{-1}, $Dcalc$ = 1.364 g/cm^3, 39379 reflections measured (9.51° ≤ 2Θ ≤ 159.05°), 3486 unique (R_{int} = 0.0501, R_{sigma} = 0.0192) which were used in all calculations. The final R_1 was 0.0371 (I > 2σ(I)) and wR_2 was 0.1028 (all data).

The crystal structure is deposited in the Cambridge Crystallographic Data Center. The deposition number is CCDC 2152246.

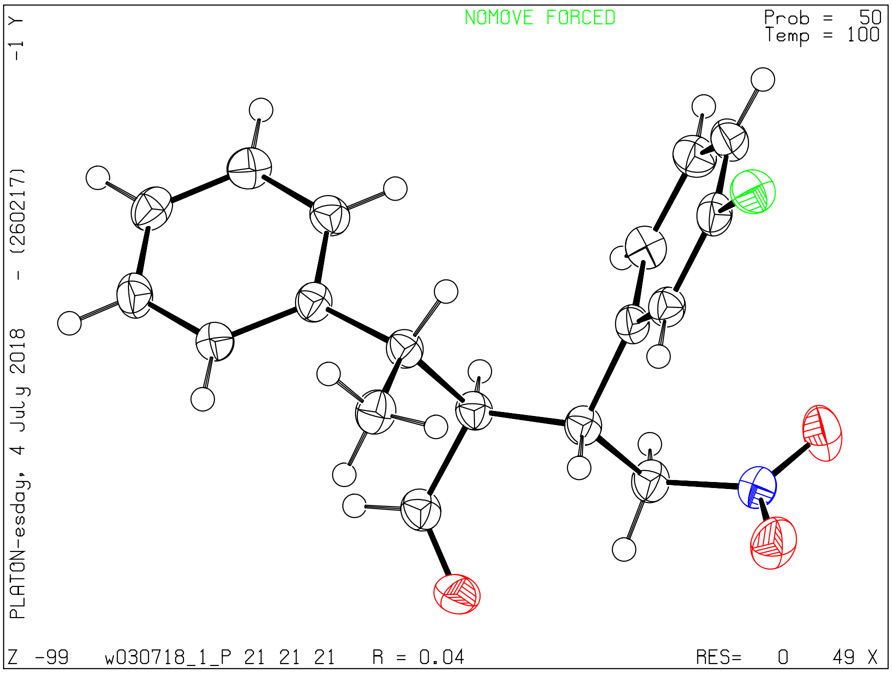

Figure S6: Crystal structure of **3ad** (CCDC 2152246).

Crystal structure of 3-(4-bromophenyl)-4-nitro-2-(1-phenylethyl)butanal (3ai)

Single crystals of **3ai** were obtained by slow evaporation from a mixture of *i*-PrOH/*n*-hexane. A suitable crystal was selected and measured on a XtaLAB Synergy, Dualflex, Pilatus 300K diffractometer. The crystal was kept at 100.0(1) K during data collection. Using Olex2,[11] the structure was solved with the ShelXT[12] structure solution program using Intrinsic Phasing and refined with the ShelXL[13] refinement package using Least Squares minimisation.

Crystal Data for $C_{18}H_{18}BrNO_3$ (M =376.24 g/mol): orthorhombic, space group $P2_12_12_1$ (no. 19), a = 8.90870(5) Å, b = 10.82307(6) Å, c = 17.02379(11) Å, V = 1641.425(16) Å^3, Z = 4, T = 100.0(1) K, μ(CuKα) = 3.537 mm^{-1}, $Dcalc$ = 1.522 g/cm^3, 39703 reflections measured (9.684° ≤ 2Θ ≤ 158.718°), 3534 unique (R_{int} = 0.0376, R_{sigma} = 0.0149) which were used in all calculations. The final R_1 was 0.0219 (I > 2σ(I)) and wR_2 was 0.0543 (all data).

The crystal structure is deposited in the Cambridge Crystallographic Data Center. The deposition number is CCDC 2152236.

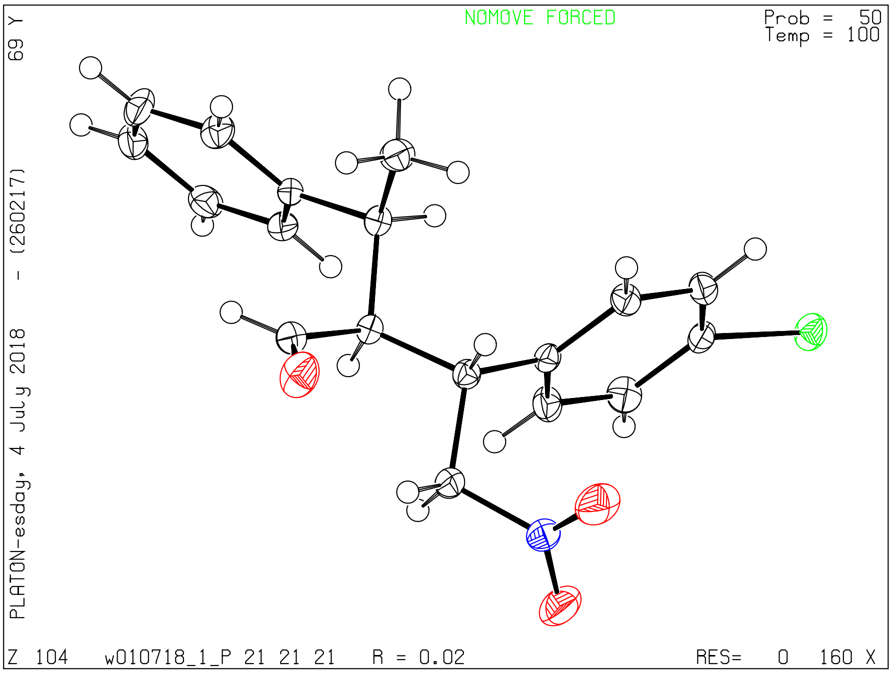

Figure S7: Crystal structure of **3ai** (CCDC 2152236).

Crystal structure of 3-(2-methoxyphenyl)-4-nitro-2-(1-phenylethyl)butanal (3al)

Single crystals of **3al** were obtained by slow evaporation from a mixture of *i*-PrOH/*n*-hexane. A suitable crystal was selected and measured on a XtaLAB Synergy, Dualflex, Pilatus 300K diffractometer. The crystal was kept at 100.0(1) K during data collection. Using Olex2,11 the structure was solved with the ShelXT[12] structure solution program using Intrinsic Phasing and refined with the ShelXL[13] refinement package using Least Squares minimisation.

Crystal Data for $C_{19}H_{21}NO_4$ (M =327.37 g/mol): orthorhombic, space group $P2_12_12_1$ (no. 19), a = 8.32170(10) Å, b = 10.59230(10) Å, c = 19.3949(2) Å, V = 1709.58(3) Å^3, Z = 4, T = 100.0(1) K, μ(CuKα) = 0.728 mm^{-1}, $Dcalc$ = 1.272 g/cm^3, 48268 reflections measured (9.12° ≤ 2Θ ≤ 159.644°), 3713 unique (R_{int} = 0.0425, R_{sigma} = 0.0164) which were used in all calculations. The final R_1 was 0.0291 (I > 2σ(I)) and wR_2 was 0.0754 (all data).

The crystal structure is deposited in the Cambridge Crystallographic Data Center. The deposition number is CCDC 2142247.

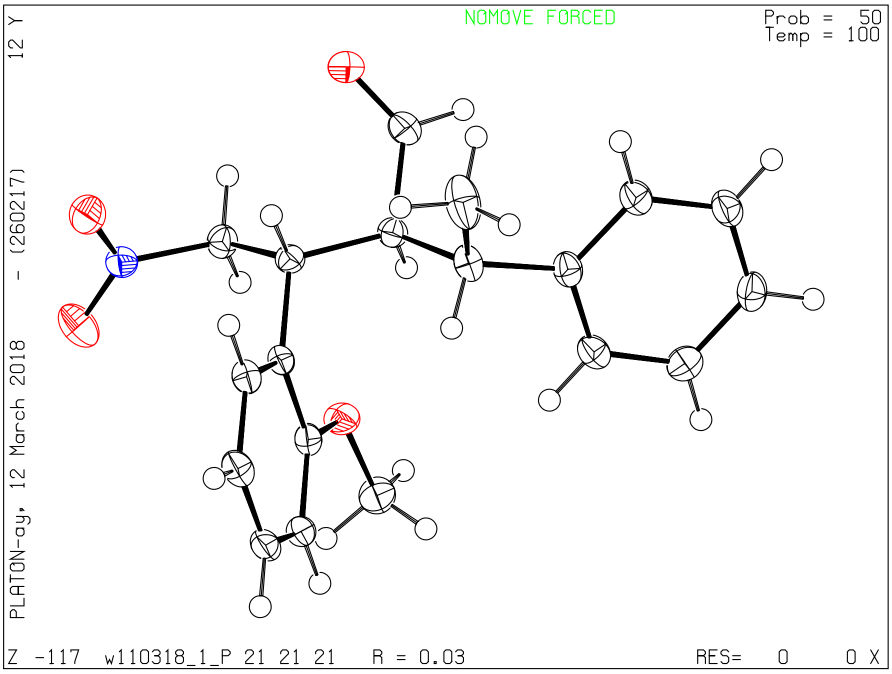

Figure S8: Crystal structure of **3al** (CCDC 2142247).

14 Computational Details

3D structures of the diastereomeric enamines were preoptimized using GFN2-xTB in the gas-phase employing xTB version 6.6.0.[14,15] Conformer-rotamer ensemble sampling at the GFN2-xTB(ALPB($CHCl_3$)) level was performed using CREST version 2.12 through the interface of CREST with xTB.[14–18] Refinement of the conformer-rotamer ensemble was performed by re-ranking to the r^2SCAN-3c/def2-TZVPP(CPCM($CHCl_3$)) level with the calculation of thermostatistical contributions using GFN2-xTB(ALPB($CHCl_3$)).[14,15,18–21] For the re-ranking, CENSO version 1.2.0 was used through the interface of CENSO with xTB and ORCA version 5.0.3.[22,23] The lowest-energy structures of the s-*trans* configured enamines were further optimized at the PBE0-D3BJ/def2-TZVP(CPCM(CHCl3) level of theory using ORCA version 5.0.3.[20,23–25] Cartesian coordinates are provided below.

(*S*)-En-A:

O	-4.04613015599938	-1.82663554330103	-1.23916173953068
C	-3.00782526918793	-1.78654213414861	-0.59488342984793
N	-2.41679643295553	-0.64384134463550	-0.21392320006608
C	-2.86985333751785	0.64698820927728	-0.65691367773465
C	-2.32557705424349	1.75102372585564	0.24382456720240
C	-2.75208138348100	1.62823435233649	1.69509711861119
C	-1.90485634593631	0.71485610525741	2.52995454455192
O	-2.41628465103196	0.54210864939358	3.74940460627431
O	-0.86486780219945	0.19958580577766	2.18868080780091
C	-2.49336302680949	0.96722901274601	-2.10643439337916
N	-1.56255251342273	0.19678690625937	-2.67540048501877
O	-3.02224495100120	1.92072834924584	-2.66430926941464
C	-2.31351890840763	-3.08318289555115	-0.19246598390569
N	-0.97727476017931	-2.89715897443808	0.35640569367705
C	0.03383533497281	-2.53335862273146	-0.45176054088458
C	1.43473845676856	-2.44979745578823	0.14449322674299
C	2.27750672264783	-3.65701582711065	-0.27340071038248
C	2.84227562561476	-3.23996062816948	-1.63113557846445
C	2.99288295861194	-1.72114442558426	-1.53622782855036
N	2.17813128119670	-1.34387041570233	-0.40054640269952
C	1.78725003658350	-0.05438432811875	-0.17391244331493
C	2.19459154970312	1.03944188806715	-0.83282559785707

C	1.73203491366955	2.41765683040967	-0.45618710648074
C	2.87717872613434	3.25257577094108	0.07463941712201
C	2.91448528969598	3.63165149723156	1.41283118992994
C	3.97147469514208	4.37757249996789	1.91888497655401
C	5.01452523263812	4.75839965930218	1.08714945841442
C	4.98972794633603	4.38647971128658	-0.25139974287345
C	3.93109041462468	3.64266923041510	-0.75078338028272
C	1.02341564390318	3.11232663551524	-1.61936567268237
H	1.01361626373533	2.31153297154581	0.36397779504653
O	-0.16488558518319	-2.25949684544652	-1.63584095632493
C	-0.89851006880109	-3.26843372820180	1.77205669920725
C	-2.35952222055106	-3.35089126829265	2.18580986663247
C	-3.05876266757489	-3.82429071891470	0.91809135280304
H	-1.56173321972715	-0.68963104898886	0.32017557648091
H	-3.96258772708840	0.66061450613862	-0.62860577997577
H	-2.68553995586433	2.69820058885300	-0.15728375007297
H	-1.23417701178618	1.76957659584304	0.17865180981231
H	-3.79415558514971	1.30826995924646	1.78778091869493
H	-2.70095910908049	2.60565434962064	2.18682307193789
H	-1.81368382973309	-0.02726781861246	4.25227962340711
H	-1.15285696315214	-0.61010531764493	-2.21910943376352
H	-1.29451269414656	0.39691296345200	-3.62529511328780
H	-2.26168060141798	-3.66901484348209	-1.11225756053327
H	1.37394460154907	-2.36082276821091	1.23013312787911
H	1.70047882737351	-4.58195658284964	-0.31474074602568
H	3.07801128289155	-3.78653596029394	0.45799452978316
H	2.13490705659630	-3.49089119515390	-2.42186133333261
H	3.78686867274237	-3.73750814763509	-1.85260715903392
H	2.64255945901084	-1.22385196016538	-2.44904017108713
H	4.03183700768963	-1.41003349525898	-1.37255858375252
H	1.08603881615459	0.05238662145334	0.65118501865210
H	2.89469153352661	0.96013901849295	-1.66018239606863
H	2.10199857681161	3.33457962277153	2.06915730193488
H	3.97817460824309	4.66253645039619	2.96538207798993
H	5.84150445829685	5.34082073592127	1.47737474695231
H	5.80046418233643	4.67833124690608	-0.91013661490882
H	3.92762142895797	3.35789891104886	-1.79773253196519

H	1.69096803993632	3.21588485932960	-2.47862033601579
H	0.68346162204995	4.11103729891255	-1.33305416414567
H	0.15845386438035	2.52747674026454	-1.93882442760757
H	-0.35039180482789	-2.51896423092800	2.34201466757311
H	-0.39679190543070	-4.23452846841692	1.88330506189721
H	-2.72047604562437	-2.35815205991102	2.46757954779873
H	-2.50875550376377	-4.01939189356856	3.03336133905428
H	-4.12723198688262	-3.61344850177314	0.89877447886029
H	-2.92100405236671	-4.89965883045314	0.78621402199334

(*R*)-En-A:

O	-3.76339756394006	-1.91389194978674	-1.70820967127833
C	-2.87946017212328	-1.81877664314416	-0.86641274581011
N	-2.51230823206827	-0.66189846025817	-0.30039581057305
C	-3.08568528137204	0.59616266213933	-0.69442490168083
C	-3.01058936219045	1.63867415976890	0.42337770709492
C	-1.65598995885401	2.28128285844017	0.69263263271420
C	-0.67565339114520	1.47654681663529	1.48675517641990
O	0.32919905943694	2.22590724156785	1.93528988106687
O	-0.73413273723278	0.28653919838156	1.70987995060328
C	-2.48793439102499	1.17793613747276	-1.97748111432777
N	-1.37116373536199	0.61162083412259	-2.43940845060397
O	-3.02760677167707	2.14812580765076	-2.49620420887829
C	-2.14246526902859	-3.06754744381863	-0.40398328894801
N	-0.88484307179234	-2.80788059201492	0.28275698309072
C	0.17396732656699	-2.35874766212068	-0.41386771998955
C	1.52510502819551	-2.38114636682590	0.28699929954516
C	2.16928630431238	-3.76809099845322	0.08622215824724
C	3.64404196443806	-3.46126245439249	-0.14963857293928
C	3.60614123223026	-2.13743125516874	-0.89437980850005
N	2.47868541936282	-1.46978311758312	-0.29426213027290
C	2.20731560181132	-0.14835143152661	-0.47386297711470
C	2.91183782413340	0.73688925508600	-1.19322207998034
C	2.52003377461354	2.18212340858850	-1.28228185222535
C	1.68373752325358	2.52025332045881	-2.50139083956452
C	1.76647853929822	1.79239378508828	-3.68409212035181

C	0.99071005184392	2.13130976127271	-4.78587530227027
C	0.11251030026091	3.20474259280657	-4.71996026509776
C	0.02254738354737	3.93992430730956	-3.54509098422150
C	0.80335685149500	3.59917159958719	-2.45096027686314
C	3.75207335420793	3.08945659327994	-1.23136931985827
H	1.89811957815965	2.41732815061686	-0.41102563148288
O	0.06249364008308	-1.99609341818158	-1.58280460591460
C	-0.90670899491632	-3.24566354069091	1.67909326164605
C	-2.39252240208217	-3.40566185575333	1.95839553517938
C	-2.94907265340591	-3.87282341227340	0.61937848319656
H	-1.79102615276317	-0.64884573780990	0.41168796726743
H	-4.14052245607057	0.42737653882820	-0.92273847613395
H	-3.40483383170340	1.19015886741168	1.33856020182389
H	-3.69230194425333	2.44170054600409	0.14239793056898
H	-1.79496713599370	3.22575838704584	1.22424592693161
H	-1.14857373076177	2.55448827831929	-0.24084179593972
H	0.95547398916225	1.65162835835570	2.40292512852950
H	-0.96713366847888	-0.21943585286878	-2.02650835307620
H	-0.92977237076571	1.00601098754532	-3.25675776374071
H	-1.95793174152327	-3.63850478861678	-1.31599656533411
H	1.38919909954168	-2.16418235559336	1.35174923095601
H	1.74302703404408	-4.24006035231757	-0.80284047247590
H	1.99159011046181	-4.42643242009549	0.93584788899330
H	4.14870992183819	-4.24869995404243	-0.71011119605489
H	4.16334689180637	-3.33205175716590	0.80329862466581
H	3.46562982412373	-2.28186710728113	-1.97635837272727
H	4.51461212835522	-1.54440719227713	-0.75449863521966
H	1.31802737304383	0.18506054157094	0.05355203755098
H	3.78228875441738	0.41375645407190	-1.75753885598533
H	2.42841815346766	0.93540821597750	-3.73747875651903
H	1.06585185780132	1.54686745397224	-5.69643522779348
H	-0.50426930435202	3.46113345958752	-5.57357956530176
H	-0.67051961008966	4.77089375097359	-3.47646303260466
H	0.71972759710196	4.17211301931735	-1.53180437905078
H	4.41066909993991	2.87962617857935	-2.07897647538254
H	4.31611639980497	2.91811935802111	-0.31123549068777
H	3.47012513208203	4.14383912721822	-1.27628676489809

H	-0.43730251377263	-2.50464189554799	2.32587678524385
H	-0.37863489987666	-4.19760564839323	1.79244823580288
H	-2.82363519575322	-2.43950333324056	2.23282445016391
H	-2.58485017206654	-4.10702425561619	2.77001341413100
H	-4.02089278171137	-3.71215621529487	0.50843569994406
H	-2.74835262609186	-4.93675854491957	0.47591026629570

15 References

(1) Kastl, R.; Wennemers, H. Peptide-Catalyzed Stereoselective Conjugate Addition Reactions Generating All-Carbon Quaternary Stereogenic Centers. *Angew. Chem. Int. Ed.* **2013**, *52*, 7228–7232.

(2) Wiesner, M.; Revell, J. D.; Wennemers, H. Tripeptides as Efficient Asymmetric Catalysts for 1,4-Addition Reactions of Aldehydes to Nitroolefins - A Rational Approach. *Angew. Chem. Int. Ed.* **2008**, *47*, 1871–1874.

(3) Schnitzer, T.; Rackl, J. W.; Wennemers, H. Stereoselective Peptide Catalysis in Complex Environments – from River Water to Cell Lysates. *Chem. Sci.* **2022**, *13*, 8963–8967.

(4) Schnitzer, T.; Wennemers, H. Influence of the *Trans/Cis* Conformer Ratio on the Stereoselectivity of Peptidic Catalysts. *J. Am. Chem. Soc.* **2017**, *139*, 15356–15362.

(5) Grünenfelder, C. E.; Kisunzu, J. K.; Wennemers, H. Peptide-Catalyzed Stereoselective Conjugate Addition Reactions of Aldehydes to Maleimide. *Angew. Chem. Int. Ed.* **2016**, *55*, 8571–8574.

(6) Lu, X.; Lin, S. Pd(II)-Bipyridine Catalyzed Conjugate Addition of Arylboronic Acid to α,β-Unsaturated Carbonyl Compounds. *J. Org. Chem.* **2005**, *70*, 9651–9653.

(7) Kagan, H. B.; Fraud, J. C. Kinetic Resolution. *Top. Stereochem.* **1988**, *18*, 149–330.

(8) Wiesner, M.; Upert, G.; Angelici, G.; Wennemers, H. Enamine Catalysis with Low Catalyst Loadings – High Efficiency via Kinetic Studies. *J. Am. Chem. Soc.* **2010**, *132*, 6–7.

(9) Keith, J. M.; Larrow, J. F., Jacobsen, E. N. Practical Considerations in Kinetic Resolution Reactions. *Adv. Synth. Catal.* **2001**, *343*, 5–26.

(10) Greenhalgh, M. D.; Taylor, J. E.; Smith, A. D. Best Practice Considerations for Using the Selectivity Factor, *s*, as a Metric for the Efficiency of Kinetic Resolutions. *Tetrahedron*, **2018**, *74*, 5554–5560.

(11) Dolomanov, O. V; Bourhis, L. J.; Gildea, R. J.; Howard, J. A. K.; Puschmann, H. OLEX2: A Complete Structure Solution, Refinement and Analysis Program. *J. Appl. Crystallogr.* **2009**, *42*, 339–341.

(12) Sheldrick, G. M. SHELXT – Integrated Space-Group and Crystal-Structure Determination. *Acta Crystallogr. Sect. A* **2015**, *71*, 3–8.

(13) Sheldrick, G. M. Crystal Structure Refinement with SHELXL. *Acta Crystallogr. Sect. C Struct. Chem.* **2015**, *71*, 3–8.

(14) Bannwarth, C.; Ehlert, S.; Grimme, S. GFN2-XTB – An Accurate and Broadly Parametrized Self-Consistent Tight-Binding Quantum Chemical Method with Multipole

Electrostatics and Density-Dependent Dispersion Contributions. *J. Chem. Theory Comput.* **2019**, *15*, 1652–1671.

(15) Bannwarth, C.; Caldeweyher, E.; Ehlert, S.; Hansen, A.; Pracht, P.; Seibert, J.; Spicher, S.; Grimme, S. Extended Tight-Binding Quantum Chemistry Methods. *WIREs Comput. Mol. Sci.* **2021**, *11*, e1493.

(16) Grimme, S. Exploration of Chemical Compound, Conformer, and Reaction Space with Meta-Dynamics Simulations Based on Tight-Binding Quantum Chemical Calculations. *J. Chem. Theory Comput.* **2019**, *15*, 2847–2862.

(17) Pracht, P.; Bohle, F.; Grimme, S. Automated Exploration of the Low-Energy Chemical Space with Fast Quantum Chemical Methods. *Phys. Chem. Chem. Phys.* **2020**, *22*, 7169–7192.

(18) Ehlert, S.; Stahn, M.; Spicher, S.; Grimme, S. Robust and Efficient Implicit Solvation Model for Fast Semiempirical Methods. *J. Chem. Theory Comput.* **2021**, *17*, 4250–4261.

(19) Grimme, S.; Hansen, A.; Ehlert, S.; Mewes, J. M. R2SCAN-3c: A "Swiss Army Knife" Composite Electronic-Structure Method. *J. Chem. Phys.* **2021**, *154*.

(20) Weigend, F.; Ahlrichs, R. Balanced Basis Sets of Split Valence, Triple Zeta Valence and Quadruple Zeta Valence Quality for H to Rn: Design and Assessment of Accuracy. *Phys. Chem. Chem. Phys.* **2005**, *7*, 3297–3305.

(21) Barone, V.; Cossi, M. Quantum Calculation of Molecular Energies and Energy Gradients in Solution by a Conductor Solvent Model. *J. Phys. Chem. A* **1998**, *102*, 1995–2001.

(22) Grimme, S.; Bohle, F.; Hansen, A.; Pracht, P.; Spicher, S.; Stahn, M. Efficient Quantum Chemical Calculation of Structure Ensembles and Free Energies for Nonrigid Molecules. *J. Phys. Chem. A* **2021**, *125*, 4039–4054.

(23) Neese, F.; Wennmohs, F.; Becker, U.; Riplinger, C. The ORCA Quantum Chemistry Program Package. *J. Chem. Phys.* **2020**, *152*, 224108.

(24) Grimme, S.; Antony, J.; Ehrlich, S.; Krieg, H. A Consistent and Accurate Ab Initio Parametrization of Density Functional Dispersion Correction (DFT-D) for the 94 Elements H-Pu. *J. Chem. Phys.* **2010**, *132*, 154104.

(25) Grimme, S.; Ehrlich, S.; Goerigk, L. Effect of the Damping Function in Dispersion Corrected Density Functional Theory. *J. Comput. Chem.* **2011**, *32*, 1456–1465.
